# Supplementary material for: “On-Water” accelerated dearomative cycloaddition via aquaphotocatalysis
Source: Nat Commun. 2024 May 8;15:3876. doi: 10.1038/s41467-024-47861-w (PMC11079013; doi:10.1038/s41467-024-47861-w)
Supplement: Supplementary file 1 — Supplementary Information [file 41467_2024_47861_MOESM1_ESM.pdf]

Supplementary Information

"On-Water" Accelerated Dearomative Cycloaddition via Aquaphotocatalysis

Soo Bok Kim, Dong Hyeon Kim, Han Yong Bae*

Department of Chemistry, Sungkyunkwan University, Suwon, Republic of Korea, 16419
*E-mail: hybae@skku.edu

Supplementary Methods

General information

■ Chemical
Chemicals were purchased from commercial suppliers (e.g., Aldrich, Alfa Aesar, TCI) and used without further purification unless otherwise stated. Anhydrous solvents, NMR solvents, and additional organic solvents were purchased from commercial vendors (e.g., Aldrich, Alfa Aesar, CIL Inc., Merck, and Wako) and used without further distillation or purification. Water was used in the second distilled state.

■ Thin-layer chromatography (TLC)
Pre-coated silica gel plates (Merck, Kieselgel 60 F254 0.25 mm) were used for TLC monitoring with UV.

■ Column chromatographic purification
Purification was carried out using silica gel (Merck, 60 Å, 230–400 mesh, 0.040–0.063 mm).

■ Nuclear magnetic resonance (NMR) spectroscopy
^{1}H NMR (500 MHz, 700 MHz), ^{13}C NMR (126 MHz, 175 MHz), and ^{19}F NMR (470 MHz) spectra were analyzed and obtained by using Bruker Ascend™ 500 and Avance™ III 700 spectrometer at 25 °C. The chemical shifts (δ) were internally referenced to tetramethylsilane (TMS δ = 0.00, ^{1}H NMR) and Chloroform-*d* ($CDCl_3$ δ = 77.160, ^{13}C NMR) for ^{1}H and ^{13}C NMR spectroscopy. Integration data are represented as follows: coupling constant (J = Hz) and multiplicity (s = singlet, bs = broad singlet, d = doublet, dd = double of doublets, ddd = doublet of doublets of doublets, t = triplet, q = quartet, m = multiplet).

■ Mass spectroscopy (MS)
High-resolution mass spectrometry was performed in fast atom bombardment (HR-FAB-MS) and electron ionization (HR-EI-MS) mode using JMS-700, or in positive electrospray ionization time-of-flight mass spectrometry (TOF-MS-ES+) mode using Xevo G2-XS_UPC^2.

■ Melting point determination (mp)
Melting point of products was determined using Büchi® M-560.

■ UV–vis spectroscopy
UV–visible spectra were obtained using a UV-1800 EnG 240 V SOFT spectrometer.

■ Photoluminescence spectroscopy (PL)
PL spectra were obtained using FluoroMate FS-2 fluorescence spectrophotometer (SCINCO, Seoul, Korea).

■ Single-crystal X-ray diffraction (SC-XRD)
The absolute stereochemistry of selective [2+2] products was determined by single-crystal X-ray diffraction.

■ Photoreactor set-up
Intermolecular catalytic [2+2] photocycloadditions were performed under irradiation with a Kessil PR160L-456 LED (maximum 50 W, wavelength maximum 456 nm) at approximately 5 cm from the reaction vessel.

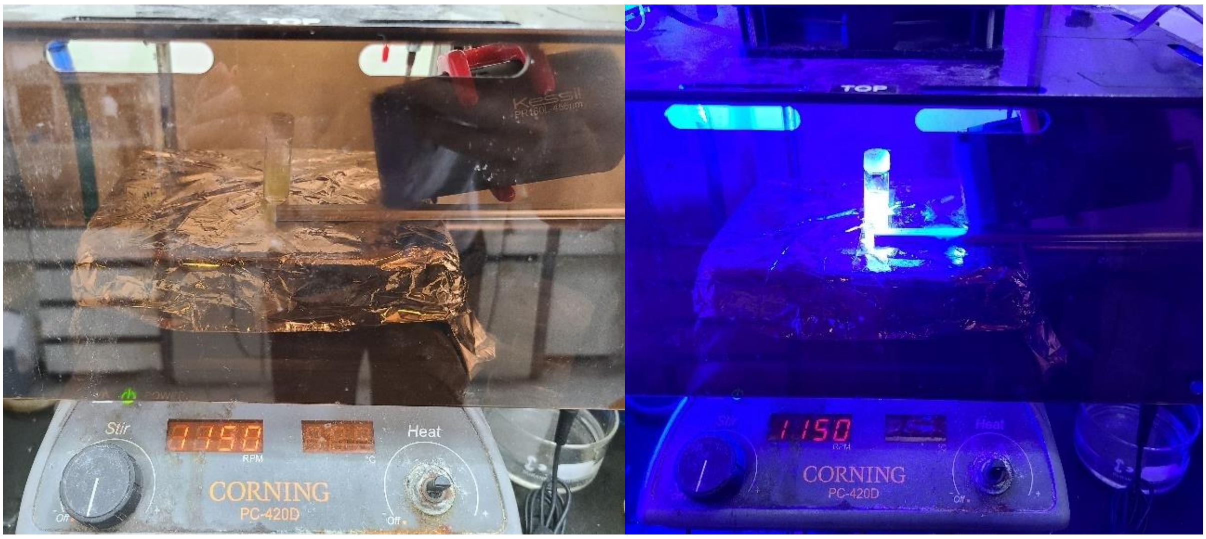

Supplementary Figure 1 Reaction set-up of the intermolecular aquacatalytic [2+2] photocycloadditions.

General procedure for the synthesis of (*E*)-phenyl ethenesulfonyl fluoride (ESF)

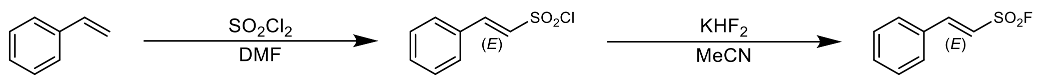

styrene **S-1** **1a**

General procedure for the synthesis of (*E*)-phenyl ethenesulfonyl chloride (S-1)
Following the literature procedure[1] with modification: SO_2Cl_2 (sulfuryl chloride; 60.0 mmol, 2.0 eq.) was slowly added in a reaction vessel filled with DMF (*N*,*N*-dimethylformamide; 2.5 mL) at 0 °C. The solution was warmed to room temperature and styrene (30.0 mmol, 1.0 eq.) was then added. After heating and stirring at 90 °C for 3 h, the reaction mixture was cooled to room temperature. Subsequently, crushed ice was poured and the residue solid was collected by filtration. The collected solid was dissolved by MTBE (methyl *tert*-butyl ether), and crushed ice was poured again. The biphasic mixture was extracted with MTBE (three times)/brine, and the organic layer was dried over anhydrous Na_2SO_4. The filtrate was concentrated under reduced pressure. The obtained crude product was purified for recrystallization by using MTBE to afford the precursor **S-1**.

General procedure for the synthesis of (*E*)-phenyl ESF (1a)
Following the literature procedure[1] with modification: the precursor **S-1** (10.0 mmol, 1.0 eq.) and KHF_2 (potassium hydrogenfluoride; 28 % aq.; 20.0 mmol, 38 mL) were dissolved in MeCN (32 mL). The solution was stirred at room temperature for overnight. After completion of the reaction, the biphasic mixture was extracted with EtOAc (three times)/brine, and the organic layer was dried over anhydrous Na_2SO_4. The residue was concentrated under reduced pressure. After concentration, the obtained crude product was purified for recrystallization by using Et_2O/hexanes to afford the starting material **1a**.

General procedure for the syntheses of (*E*)-β-aryl ESFs

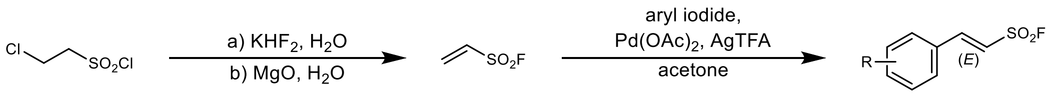

S-2 **S-3** **1b–1q**

General procedure for the synthesis ESF (S-3)
Following the literature procedure[2] with modification: KHF_2 (0.48 mol, 2.4 eq.) was dissolved in a reaction vessel filled with H_2O (100 mL). Sulfonyl chloride **S-2** (0.2 mol, 1.0 eq.) was slowly added to the completely dissolved solution in two portions and vigorously stirred for overnight. When stirring was stopped, the solution was separated into two phases. The upper part is an aqueous phase, and the lower part is pure 2-chloroethanesulfonyl chloride, a precursor of ESF **S-3**. Crushed ice was poured to the biphasic solution. After cooling to approximately 10 °C, MgO (0.1 mol, 0.5 eq.) was added portionwise over 15 min while stirring the biphasic solution and vigorously stirred further for 24 h. Subsequently, the resulting biphasic mixture was extracted with CH_2Cl_2 (three times)/brine, and the organic layer was dried over anhydrous Na_2SO_4. The filtrate was concentrated using a rotary evaporator at low temperature (~18 °C) to afford ESF **S-3**.

General procedure for the synthesis of (*E*)-β-aryl ESFs (1b–1q)
Following the literature procedure[3] with modification: An oven-dried Schlenk tube was charged with AgTFA (silver trifluoroacetate; 12.0 mmol, 1.2 eq.) and $Pd(OAc)_2$ (2.0 mol%) in an argon atmosphere. Subsequently, anhydrous acetone (20 mL), corresponding aryl iodide (10.0 mmol, 1.0 eq.), and ESF **S-3**. (20.0 mmol, 2.0 eq.) were sequentially added into the Schlenk tube. The reaction mixture was then refluxed for 24 h. After checking the reaction was completed by monitoring TLC, the reaction mixture was cooled to room temperature and evaporated under reduced. The obtained crude product was purified by column chromatography to afford starting materials, (*E*)-β-aryl ESFs **(1b–1q)**.

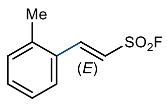

1b

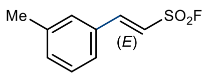

1c

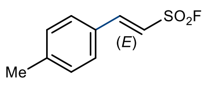

1d

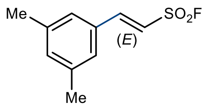

1e

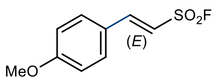

1f

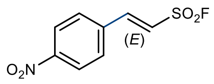

1g

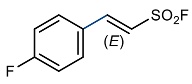

1h

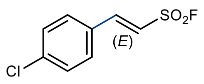

1i

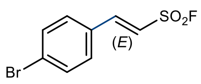

1j

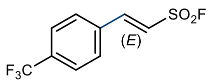

1k

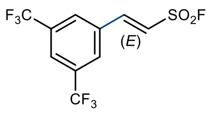

1l

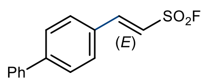

1m

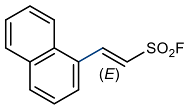

1n

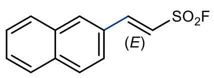

1o

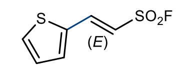

1p

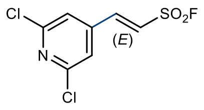

1q

General procedure for the protection of reagents

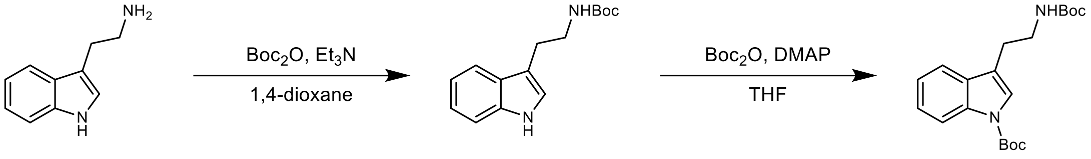

tryptamine **S-4** **2g**

General procedure for the synthesis of *tert*-butyl (2-(*1H*-indol-3-yl)ethyl)carbamate (S-4)
Following the literature procedure with modification[4]: To a solution of tryptamine (30.0 mmol, 1.0 eq.) dissolved in 1,4-dioxane (30.0 mL), Et_3N (60.0 mmol, 2.0 eq.) was added. Then, a solution of Boc_2O (di-*tert*-butyl decarbonate; 33.0 mmol, 1.1 eq.) in 1,4-dioxane (30 mL) was slowly added and stirred at room temperature for 1 h. After the reaction was completed, the reaction mixture was concentrated under reduced pressure. Thereafter, the obtained crude product was purified by column chromatography (EtOAc:hexanes = 10:1 v/v) to afford *tert*-butyl (2-(1*H*-indol-3-yl)ethyl)carbamate (**S-4**).

General procedure for the synthesis of *tert*-butyl 3-(2-((*tert*-butoxycarbonyl)amino)ethyl)-*1H*-indole-1-carboxylate (2g)
Following the literature procedure with modification[5]: To a solution of **S-4** (10.0 mmol, 1.0 eq.) and DMAP (4-(dimethylamino)pyridine; 1.0 mol%) in THF (tetrahydrofuran; 10.0 mL), Et_3N (20.0 mmol, 2.0 eq.) was added. Subsequently, Boc_2O (12.0 mmol) was slowly added and stirred at room temperature for 2 h. After the reaction was completed, the reaction mixture was concentrated under reduced pressure. Thereafter, the obtained crude product was purified by column chromatography (EtOAc:hexanes = 20:1 v/v) to afford *tert*-butyl 3-(2-((*tert*-butoxycarbonyl)amino)ethyl)-*1H*-indole-1-carboxylate (**2g**).

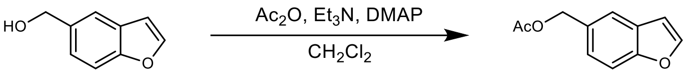

S-5 **2h**

General procedure for the synthesis of benzofuran-5-ylmethyl acetate (2h)
Following the literature procedure with modification[6]: To a cooled (0 °C) solution of 1-benzofuran-5-ylmethanol **S-5** (CAS: 31823-05-9, 10.0 mmol, 1.0 eq.) and DMAP (5.0 mol%) in CH_2Cl_2 (50.0 mL), Et_3N (20.0 mmol, 2.0 eq.) was added. Subsequently, Ac_2O (acetic anhydride; 15.0 mmol, 1.5 eq.) was slowly added and warmed to room temperature. After the reaction mixture was stirred for 12 h, brine was added and extracted with CH_2Cl_2 (three times). The combined organic layer was dried over anhydrous Na_2SO_4, and the residue was concentrated under reduced pressure. Thereafter, the obtained crude product was purified by column chromatography (EtOAc:hexanes = 10:1 v/v) to afford benzofuran-5-ylmethyl acetate (**2h**).

benzofuran-5-ylmethyl acetate (2h)

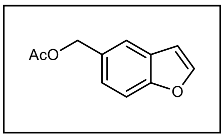

New compound, yellowish liquid.
^{1}H NMR (500 MHz, $CDCl_3$): δ 7.61 – 7.59 (m, 2H), 7.47 (d, *J* = 8.5 Hz, 1H), 7.28 (dd, *J* = 8.5, 1.5 Hz, 1H), 6.73 (d, *J* = 2.0 Hz, 1H), 5.18 (s, 2H), 2.08 (s, 3H).
^{13}C NMR (126 MHz, $CDCl_3$): δ 170.9, 154.8, 145.7, 130.6, 127.7, 125.1, 121.6, 111.5, 106.6, 66.6, 21.1.
HR-MS: *m/z* calcd. [$C_{11}H_{10}O_3$ + Na]: 213.0528; found (TOF MS ES+): 213.0534.

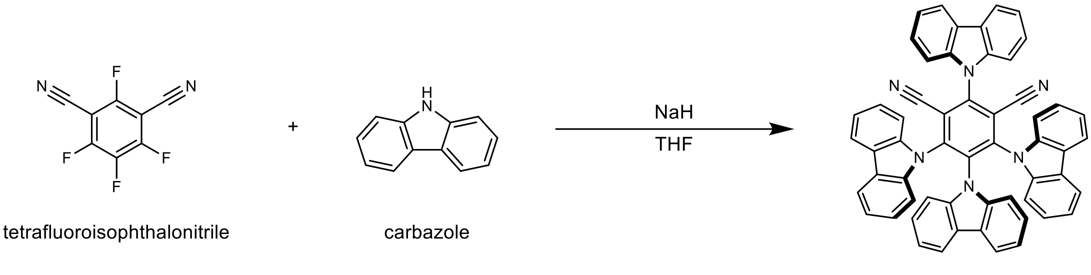

4Cz-IPN

General procedure for the synthesis of 4Cz-IPN

Following the literature procedure[7] with modification: An oven-dried two-neck round-bottom flask was charged in a solution of carbazole (10.0 mmol, 5.0 eq.) dissolved in anhydrous THF (40 mL) under argon atmosphere and cooled to 0 °C. NaH (60% in oil; 15.0 mmol, 7.5 eq.) was slowly added into the solution while flowing argon. The suspension was warmed to room temperature and stirred for 30 min. Subsequently, tetrafluoroisophthalonitrile (2.0 mmol, 1.0 eq.) was added and the reaction mixture was stirred for an additional 12 h. After checking the tetrafluoroisophthalonitrile was fully consumed by monitoring TLC, H_2O (2.0 mL) was slowly poured into the reaction mixture to quench the excess NaH. Thereafter, the resulting residue was yielded after concentration under reduced pressure and washed with H_2O/EtOH. The obtained crude product was then purified for recrystallization by Et_2O/CH_2Cl_2 to afford the desired photocatalyst **4Cz-IPN**.

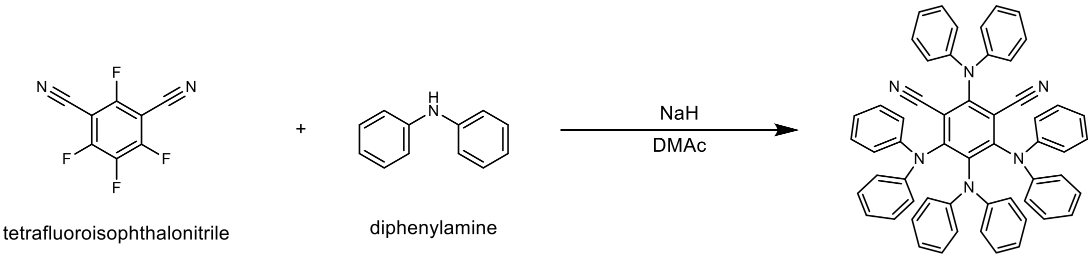

4DP-IPN

General procedure for the synthesis of 4DP-IPN

Following the literature procedure[7] with modification: An oven-dried two-neck round-bottom flask was charged in a solution of diphenylamine (9.0 mmol, 4.5 eq.) dissolved in anhydrous DMAc (*N*,*N*-dimethylacetamide; 5 mL) under argon atmosphere and cooled to 0 °C. NaH (60% in oil; 12.0 mmol, 6.0 eq.) was slowly added in the solution while flowing argon. The suspension was warmed to room temperature and stirred for 30 min. A solution of tetrafluoroisophthalonitrile (1.0 mmol, 1.0 eq.) dissolved in DMAc (5 mL) was added and the reaction mixture was stirred at 100 °C for an additional 10 h. After checking the tetrafluoroisophthalonitrile was fully consumed by monitoring TLC, H_2O (2.0 mL) was slowly poured into reaction mixture to quench the remaining excess amount of NaH. Thereafter, MeOH was added to the resulting mixture to precipitate the crude product the precipitate was collected through filtration and purified by column chromatography (CH_2Cl_2:hexanes = 2:3 v/v) to afford the desired photocatalyst **4DP-IPN**.

General procedure for dearomative [2+2] cycloaddition

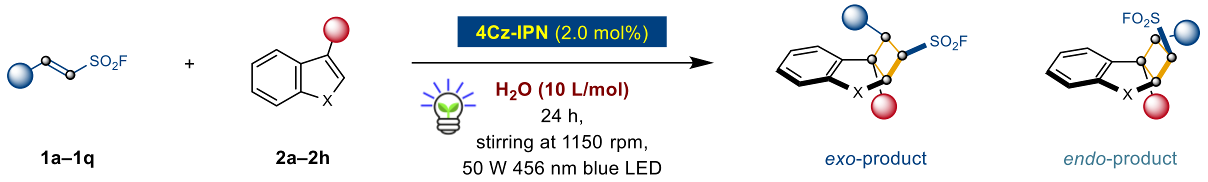

1a–1q (0.4 mmol, 1.0 eq.) and 4Cz-IPN (2.0 mol%) were added in a vial without further drying with multiple magnetic stirring bars. Afterwards, corresponding **2a–2h** (2.0 mmol, 5.0 eq.) and H_2O (deionized, 10 L/mol, 4.0 mL) were sequentially added to the reaction vial without further degassing. The suspension was then stirred vigorously (rpm > 1000) under irradiation with a 50 W 456 nm blue LED for 24 h. The distance between the light source and the reaction vial was approximately 5 cm, and an electric fan (PR160 Rig w/ Fan Kit) was used to decrease the temperature of the reaction vial. Subsequently, the crude mixture was extracted with EtOAc (three times)/brine. The organic layer was then dried over anhydrous Na_2SO_4 and concentrated under reduced pressure. The resulting residue was purified by column chromatography to afford the desired [2+2] cycloadducts, and further purification was performed by recrystallization by Et_2O/pentane. *Exo-*/*endo*-ratio was determined by ^1H NMR of the crude mixture.

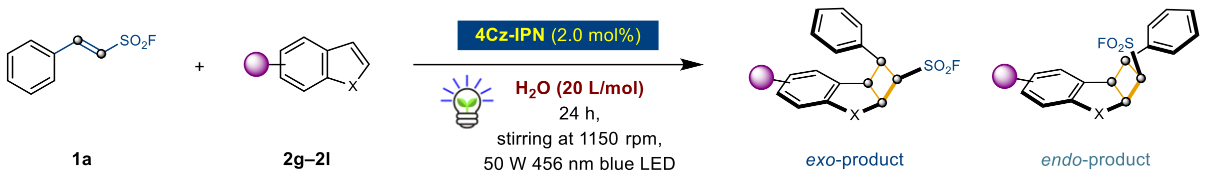

1a (0.4 mmol, 1.0 eq.) and 4Cz-IPN (2.0 mol%) were added in a vial without further drying with multiple magnetic stirring bars. Afterwards, corresponding **2g–2l** (2.0 mmol, 5.0 eq.) and H_2O (deionized, 20 L/mol, 8.0 mL) were sequentially added to the reaction vial without further degassing. The suspension was then stirred vigorously (rpm > 1000) under irradiation with a 50 W 456 nm blue LED for 24 h. The distance between the light source and the reaction vial was approximately 5 cm, and an electric fan (PR160 Rig w/ Fan Kit) was used to decrease the temperature of the reaction vial. Subsequently, the crude mixture was extracted with EtOAc (three times)/ brine. The organic layer was then dried over anhydrous Na_2SO_4 and concentrated under reduced pressure. The resulting residue was purified by column chromatography to afford the desired [2+2] cycloadducts, and further purification was performed by recrystallization by Et_2O/pentane. *Exo-*/*endo*-ratio was determined by ^1H NMR of the crude mixture.

Characterization data of the desired [2+2] cycloadducts

1-phenyl-1,2,2a,7b-tetrahydrocyclobuta[*b*]benzofuran-2-sulfonyl fluoride (**3aa**-*exo*)

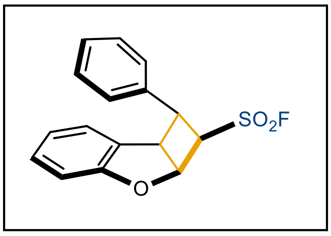

New compound, white crystal, 94.1 mg, 77% yield.
Purification conditions for column chromatography: hexanes:Et_2O 100:0 to 50:1
Purification conditions for recrystallization: pentane/Et_2O
R$_f$ = 0.53 (acetone:hexanes = 1:6 v/v)
mp: 92 °C.
^{1}H NMR (500 MHz, $CDCl_3$): δ 7.30 – 7.25 (m, 3H), 7.18 – 7.15 (m, 1H), 7.00 – 6.99 (m, 2H), 6.93 (d, *J* = 8.1 Hz, 1H), 6.75 – 6.72 (m, 1H), 6.52 (d, *J* = 7.5 Hz, 1H), 5.60 (dd, *J* = 7.1, 4.6 Hz, 1H), 4.58 – 4.51 (m, 2H), 4.43 – 4.39 (m, 1H).
^{13}C NMR (126 MHz, $CDCl_3$): δ 160.1, 134.4, 129.9, 128.7, 128.1, 128.0, 127.3, 123.9, 122.0, 111.5, 77.4, 63.8 (d, $J_{C\text{-}F}$ = 14.1 Hz), 48.3 (d, $J_{C\text{-}F}$ = 1.6 Hz), 42.6.
^{19}F NMR (471 MHz, $CDCl_3$): δ 50.5 (s, 1F).
HR-MS: *m/z* calcd. $[C_{16}H_{13}FO_3S]^+$: 304.0569; found (EI+): 304.0567.

1-phenyl-1,2,2a,7b-tetrahydrocyclobuta[*b*]benzofuran-2-sulfonyl fluoride (**3aa**-*endo*)

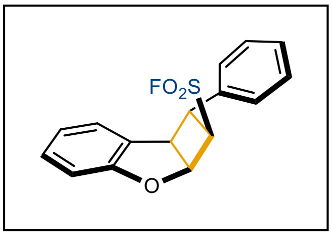

New compound, white crystal, 26.0 mg, 21% yield.
Purification conditions for column chromatography: hexanes:Et_2O 50:1 to 20:1
Purification conditions for recrystallization: pentane/Et_2O
R$_f$ = 0.50 (acetone:hexanes = 1:6 v/v)
mp: 96 °C.
^{1}H NMR (500 MHz, $CDCl_3$): δ 7.45 – 7.42 (m, 2H), 7.37 – 7.33 (m, 3H), 7.26 – 7.22 (m, 2H), 7.02 (d, *J* = 8.1 Hz, 1H), 6.98 – 6.95 (m, 1H), 5.66 – 5.63 (m, 1H), 4.57 – 4.54 (m, 1H), 4.30 – 4.23 (m, 2H).
^{13}C NMR (126 MHz, $CDCl_3$): δ 161.6, 138.7, 129.7, 129.5, 128.4, 128.2, 126.4, 124.1, 122.3, 111.0, 78.2, 64.1 (d, $J_{C\text{-}F}$ = 13.3 Hz), 51.0, 48.5 (d, $J_{C\text{-}F}$ = 1.1 Hz).
^{19}F NMR (471 MHz, $CDCl_3$): δ 57.8 (s, 1F).
HR-MS: *m/z* calcd. $[C_{16}H_{13}FO_3S]^+$: 304.0569; found (EI+): 304.0571.

1-(*o*-tolyl)-1,2,2a,7b-tetrahydrocyclobuta[*b*]benzofuran-2-sulfonyl fluoride (**3ba**-*exo*)

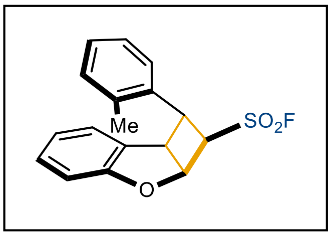

New compound, white crystal, 93.9 mg, 74% yield.
Purification conditions for column chromatography: hexanes:Et_2O 100:0 to 50:1
Purification conditions for recrystallization: pentane/Et_2O
R$_f$ = 0.54 (acetone:hexanes = 1:6 v/v)
mp: 106 °C.
^{1}H NMR (500 MHz, $CDCl_3$): δ 7.25 – 7.24 (m, 1H), 7.20 – 7.17 (m, 1H), 7.15 – 7.12 (m, 1H), 7.07 – 7.04 (m, 1H), 6.91 (d, *J* = 8.1 Hz, 1H), 6.73 (d, *J* = 7.7 Hz, 1H), 6.69 – 6.66 (m, 1H), 6.38 (d, *J* = 7.5 Hz, 1H), 5.64 (dd, *J* = 7.1, 4.6 Hz, 1H), 4.69 – 4.61 (m, 2H), 4.52 – 4.48 (m, 1H), 2.47 (s, 3H).
^{13}C NMR (126 MHz, $CDCl_3$): δ 160.0, 135.5, 132.6, 130.7, 129.9, 127.9, 127.0, 126.4, 125.8, 124.0, 122.2, 111.5 ,77.5, 62.6 (d, $J_{C\text{-}F}$ = 14.4 Hz), 47.2 (d, $J_{C\text{-}F}$ = 1.8 Hz), 40.6, 19.9.
^{19}F NMR (471 MHz, $CDCl_3$): δ 50.9 (s, 1F).
HR-MS: *m/z* calcd. $[C_{17}H_{15}FO_3S + Na]$: 341.0624; found (TOF MS ES+): 341.0628.

1-(*o*-tolyl)-1,2,2a,7b-tetrahydrocyclobuta[*b*]benzofuran-2-sulfonyl fluoride (**3ba**-*endo*)

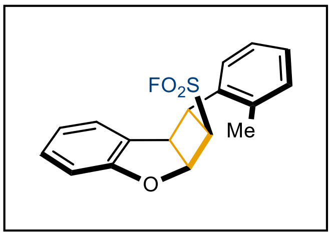

New compound, white crystal, 21.5 mg, 17% yield.
Purification conditions for column chromatography: hexanes:Et_2O 50:1 to 20:1
Purification conditions for recrystallization: pentane/Et_2O
R$_f$ = 0.50 (acetone:hexanes = 1:6 v/v)
mp: 109 °C.
^{1}H NMR (500 MHz, $CDCl_3$): δ 7.38 – 7.37 (m, 1H), 7.35 – 7.32 (m, 1H), 7.28 – 7.23 (m, 4H), 7.20 (d, *J* = 7.3 Hz, 1H), 7.03 (d, *J* = 8.1 Hz, 1H), 6.97 – 6.94 (m, 1H), 5.71 – 5.68 (m, 1H), 4.65 – 4.62 (m, 1H), 4.45 (dd, *J* = 8.0, 5.6 Hz, 1H), 4.19 – 4.16 (m, 1H), 2.33 (s, 3H).
^{13}C NMR (126 MHz, $CDCl_3$): δ 161.7, 136.9, 136.3, 131.2, 129.8, 128.4, 128.2, 127.1, 125.2, 124.4, 122.2, 111.0, 78.1, 63.5 (d, J_{C-F} = 13.3 Hz), 49.6 (d, J_{C-F} = 1.4 Hz), 47.7, 20.2.
^{19}F NMR (471 MHz, $CDCl_3$): δ 58.3 (s, 1F).
HR-MS: *m/z* calcd. $[C_{17}H_{15}FO_3S]^+$: 318.0726; found (EI+): 318.0722.

1-(*m*-tolyl)-1,2,2a,7b-tetrahydrocyclobuta[*b*]benzofuran-2-sulfonyl fluoride (**3ca**-*exo*)

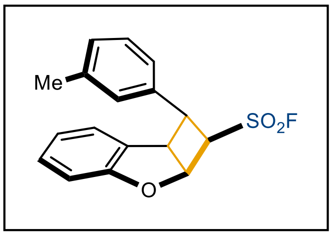

New compound, white solid, 95.2 mg, 75% yield.
Purification conditions for column chromatography: hexanes:Et_2O 100:0 to 50:1
Purification conditions for recrystallization: pentane/Et_2O
R$_f$ = 0.53 (acetone:hexanes = 1:6 v/v)
mp: 95 °C.
^{1}H NMR (500 MHz, $CDCl_3$): δ 7.20 – 7.15 (m, 2H), 7.07 (d, *J* = 7.6 Hz, 1H), 6.94 (d, *J* = 8.1 Hz, 1H), 6.80 – 6.74 (m, 3H), 6.54 (d, *J* = 7.5 Hz, 1H), 5.61 (dd, *J* = 7.1, 4.6 Hz, 1H), 4.57 – 4.51 (m, 2H), 4.40 – 4.36 (m, 1H), 2.28 (s, 3H).
^{13}C NMR (126 MHz, $CDCl_3$): δ 160.1, 138.4, 134.3, 129.9, 128.8, 128.6, 128.2, 128.1, 124.4, 124.0, 122.0 ,115.5, 77.4, 64.0 (d, J_{C-F} = 13.9 Hz), 48.4 (d, J_{C-F} = 1.7 Hz), 42.7, 21.5.
^{19}F NMR (471 MHz, $CDCl_3$): δ 50.4 (s, 1F).
HR-MS: *m/z* calcd. $[C_{17}H_{15}FO_3S]^+$: 318.0726; found (EI+): 318.0722.
§ **Note**: The peak at 77.4 ppm overlaps the $CDCl_3$ peak.

1-(*m*-tolyl)-1,2,2a,7b-tetrahydrocyclobuta[*b*]benzofuran-2-sulfonyl fluoride (**3ca**-*endo*)

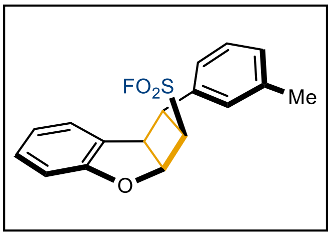

New compound, white crystal, 27.3 mg, 21% yield.
Purification conditions for column chromatography: hexanes:Et_2O 50:1 to 20:1
Purification conditions for recrystallization: pentane/Et_2O
R$_f$ = 0.50 (acetone:hexanes = 1:6 v/v)
mp: 98 °C.
^{1}H NMR (500 MHz, $CDCl_3$): δ 7.34 – 7.30 (m, 1H), 7.25 – 7.21 (m, 2H), 7.16 (d, *J* = 7.6 Hz, 1H), 7.14 – 7.12 (m, 2H), 7.01 (d, *J* = 8.1 Hz, 1H), 6.98 – 6.95 (m, 1H), 5.65 – 5.62 (m, 1H), 4.56 – 4.53 (m, 1H), 4.28 – 4.25 (m, 1H), 4.20 (dd, *J* = 7.9, 5.8 Hz, 1H), 2.40 (s, 3H).
^{13}C NMR (126 MHz, $CDCl_3$): δ 161.6, 139.3, 138.7, 129.7, 129.3, 129.0, 128.5, 127.1, 124.1, 123.4, 122.3, 111.0, 78.2, 64.1 (d, J_{C-F} = 13.2 Hz), 51.0, 48.6 (d, J_{C-F} = 0.8 Hz), 21.6.
^{19}F NMR (471 MHz, $CDCl_3$): δ 57.8 (s, 1F).
HR-MS: *m/z* calcd. for $[C_{17}H_{15}FO_3S]^+$: 318.0726; found (EI+): 318.0728.

1-(*p*-tolyl)-1,2,2a,7b-tetrahydrocyclobuta[*b*]benzofuran-2-sulfonyl fluoride (**3da**-*exo*)

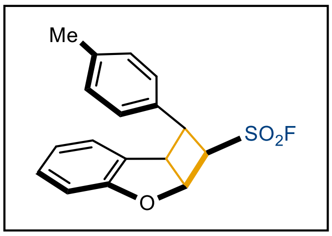

New compound, white crystal, 94.2 mg, 74% yield.
Purification conditions for column chromatography: hexanes:Et_2O 100:0 to 50:1
Purification conditions for recrystallization: pentane/Et_2O
R_f = 0.54 (acetone:hexanes = 1:6 v/v)
mp: 125 °C.
^{1}H NMR (500 MHz, $CDCl_3$): δ 7.21 – 7.17 (m, 1H), 7.08 (d, J = 7.9 Hz, 2H), 6.94 (d, J = 8.1 Hz, 1H), 6.88 (d, J = 8.0 Hz, 2H), 6.78 – 6.75 (m, 1H), 6.56 (d, J = 7.5 Hz, 1H), 5.60 (dd, J = 6.9, 4.9 Hz, 1H), 4.54 – 4.51 (m, 2H), 4.39 – 4.35 (m, 1H), 2.31 (s, 3H).
^{13}C NMR (126 MHz, $CDCl_3$): δ 160.1, 137.9, 131.4, 129.9, 129.4, 128.1, 127.4, 124.0, 122.0, 115.5, 77.4, 64.2 (d, $J_{C\text{-}F}$ = 13.7 Hz), 48.4 (d, $J_{C\text{-}F}$ = 1.7 Hz), 42.5, 21.3.
^{19}F NMR (471 MHz, $CDCl_3$): δ 50.5 (s, 1F).
HR-MS: *m/z* calcd. $[C_{17}H_{15}FO_3S]^+$: 318.0726; found (EI+): 318.0723.

1-(*p*-tolyl)-1,2,2a,7b-tetrahydrocyclobuta[*b*]benzofuran-2-sulfonyl fluoride (**3da**-*endo*)

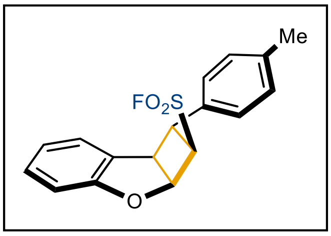

New compound, white crystal, 26.7 mg, 21% yield.
Purification conditions for column chromatography: hexanes:Et_2O 50:1 to 20:1
Purification conditions for recrystallization: pentane/Et_2O
R_f = 0.51 (acetone:hexanes = 1:6 v/v)
mp: 94 °C.
^{1}H NMR (500 MHz, $CDCl_3$): δ 7.26 – 7.20 (m, 8H), 7.01 (d, J = 8.1 Hz, 1H), 6.97 – 6.94 (m, 1H), 5.65 – 5.62 (m, 1H), 4.53 – 4.51 (m, 1H), 4.26 – 4.24 (m, 1H), 4.20 (dd, J = 7.8, 5.9 Hz, 1H), 2.38 (s, 3H).
^{13}C NMR (126 MHz, $CDCl_3$): δ 161.6, 138.1, 135.7, 130.1, 129.7, 128.5, 126.3, 124.1, 122.2, 111.0, 78.2, 64.3 (d, $J_{C\text{-}F}$ = 13.2 Hz), 50.8, 48.7, 21.2.
^{19}F NMR (471 MHz, $CDCl_3$): δ 57.8 (s, 1F).
HR-MS: *m/z* calcd. $[C_{17}H_{15}FO_3S]^+$: 318.0726; found (EI+): 318.0729.

1-(3,5-dimethylphenyl)-1,2,2a,7b-tetrahydrocyclobuta[*b*]benzofuran-2-sulfonyl fluoride (**3ea**-*exo*)

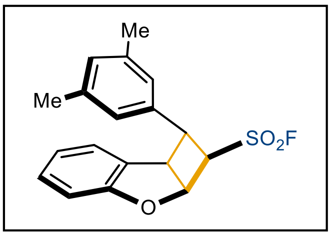

New compound, white crystal, 109.7 mg, 83% yield.
Purification conditions for column chromatography: hexanes:Et_2O 100:0 to 55:1
Purification conditions for recrystallization: pentane/Et_2O
R_f = 0.57 (acetone:hexanes = 1:6 v/v)
mp: 125 °C.
^{1}H NMR (500 MHz, $CDCl_3$): δ 7.20 – 7.17 (m, 1H), 6.94 (d, J = 8.1 Hz, 1H), 6.90 (s, 1H), 6.77 – 6.74 (m, 1H), 6.57 – 6.54 (m, 3H), 5.59 (dd, J = 7.1, 4.7 Hz, 1H), 4.56 – 4.49 (m, 2H), 4.35 – 4.31 (m, 1H), 2.23 (s, 6H).
^{13}C NMR (126 MHz, $CDCl_3$): δ 160.1, 138.2, 134.2, 129.8, 129.7, 128.2, 125.3, 124.1, 121.9, 111.5, 77.4, 64.1 (d, $J_{C\text{-}F}$ = 13.7 Hz), 48.3 (d, $J_{C\text{-}F}$ = 1.6 Hz), 42.6, 21.4.
^{19}F NMR (471 MHz, $CDCl_3$): δ 50.4 (s, 1F).
HR-MS: *m/z* calcd. $[C_{18}H_{17}FO_3S]^+$: 332.0882; found (EI+): 332.0880
§ **Note**: The peak at 77.4 ppm overlaps the $CDCl_3$ peak.

1-(3,5-dimethylphenyl)-1,2,2a,7b-tetrahydrocyclobuta[*b*]benzofuran-2-sulfonyl fluoride (**3ea**-*endo*)

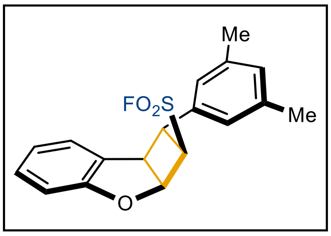

New compound, white solid, 21.5 mg, 16% yield.
Purification conditions for column chromatography: hexanes:Et_2O 55:1 to 25:1
Purification conditions for recrystallization: pentane/Et_2O
R_f = 0.55 (acetone:hexanes = 1:6 v/v)
mp: 140 °C.
^{1}H NMR (500 MHz, $CDCl_3$): δ 7.26 – 7.20 (m, 2H), 7.01 – 6.92 (m, 5H), 5.65 – 5.62 (m, 1H), 4.55 – 4.53 (m, 1H), 4.26 – 4.24 (m, 1H), 4.15 (dd, J = 7.9, 5.7 Hz, 1H), 2.35 (s, 6H).
^{13}C NMR (126 MHz, $CDCl_3$): δ 161.6, 139.2, 138.7, 129.8, 129.6, 128.6, 124.2, 124.1, 122.2, 110.9, 78.2, 64.1 (d, $J_{C\text{-}F}$ = 13.2 Hz), 51.0, 48.7, 21.5.
^{19}F NMR (471 MHz, $CDCl_3$): δ 57.9 (s, 1F).
HR-MS: *m/z* calcd. $[C_{18}H_{17}FO_3S]^+$: 332.0882; found (EI+): 332.0885.

1-(4-methoxyphenyl)-1,2,2a,7b-tetrahydrocyclobuta[*b*]benzofuran-2-sulfonyl fluoride (**3fa**-*exo*)

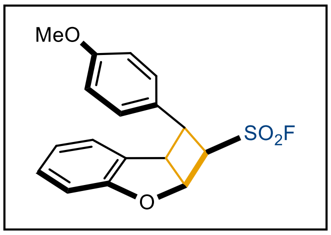

New compound, white solid, 108.8 mg, 81% yield.
Purification conditions for column chromatography: hexanes:Et_2O 100:1 to 20:1
Purification conditions for recrystallization: pentane/Et_2O
R_f = 0.52 (acetone:hexanes = 1:4 v/v)
mp: 99 °C.
^{1}H NMR (500 MHz, $CDCl_3$): δ 7.22 – 7.19 (m, 1H), 6.95 (d, J = 8.1 Hz, 1H), 6.91 – 6.89 (m, 2H), 6.82 – 6.76 (m, 3H), 6.57 (d, J = 7.5 Hz, 1H), 5.59 (dd, J = 7.1, 4.6 Hz, 1H), 4.53 – 4.48 (m, 2H), 4.37 – 4.33 (m, 1H), 3.78 (s, 3H).
^{13}C NMR (126 MHz, $CDCl_3$): δ 160.2, 159.4, 129.9, 128.7, 128.2, 126.5, 124.0, 122.1, 114.1, 111.6, 77.3, 64.5 (d, $J_{C\text{-}F}$ = 13.5 Hz), 55.4, 48.6 (d, $J_{C\text{-}F}$ = 1.2 Hz), 42.2.
^{19}F NMR (471 MHz, $CDCl_3$): δ 50.5 (s, 1F).
HR-MS: *m/z* calcd. $[C_{17}H_{15}FO_4S]^+$: 334.0675; found (EI+): 334.0672.

1-(4-methoxyphenyl)-1,2,2a,7b-tetrahydrocyclobuta[*b*]benzofuran-2-sulfonyl fluoride (**3fa**-*endo*)

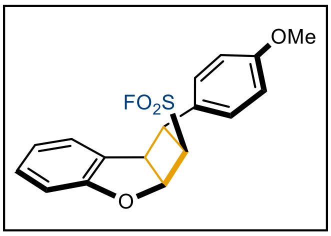

New compound, white solid, 22.1 mg, 17% yield.
Purification conditions for column chromatography: hexanes:Et_2O 20:1 to 8:1
Purification conditions for recrystallization: pentane/Et_2O
R_f = 0.48 (acetone:hexanes = 1:4 v/v)
mp: 94 °C.
^{1}H NMR (500 MHz, $CDCl_3$): δ 7.26 – 7.19 (m, 4H), 7.01 (d, J = 8.1 Hz, 1H) 6.96 – 6.94 (m, 3H), 5.65 – 5.62 (m, 1H), 4.50 – 4.47 (m, 1H), 4.25 – 4.22 (m, 1H), 4.18 (dd, J = 7.9, 5.8 Hz, 1H), 3.83 (s, 3H).
^{13}C NMR (126 MHz, $CDCl_3$): δ 161.6, 159.5, 130.8, 129.7, 127.6, 124.1, 122.2, 114.8, 111.0, 78.2, 64.5 (d, $J_{C\text{-}F}$ = 12.9 Hz), 55.6, 50.6, 48.7 (d, $J_{C\text{-}F}$ = 1.0 Hz).
^{19}F NMR (471 MHz, $CDCl_3$): δ 57.7 (s, 1F).
HR-MS: *m/z* calcd. $[C_{17}H_{15}FO_4S]^+$: 334.0675; found (EI+): 334.0678.

1-(4-nitrophenyl)-1,2,2a,7b-tetrahydrocyclobuta[*b*]benzofuran-2-sulfonyl fluoride (**3ga**-*exo*)

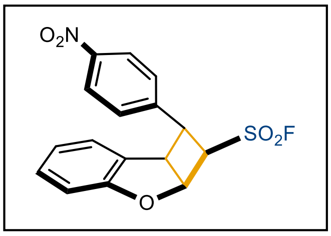

New compound, yellowish solid, 38.0 mg, 27% yield.
Purification conditions for column chromatography: hexanes:Et_2O 50:1 to 10:1
Purification conditions for recrystallization: pentane/Et_2O
R_f = 0.41 (acetone:hexanes = 1:4 v/v)
mp: 68 °C.
^{1}H NMR (500 MHz, $CDCl_3$): δ 8.19 – 8.17 (m, 2H), 7.24 – 7.21 (m, 3H), 6.98 (d, J = 8.1 Hz, 1H), 6.79 – 6.76 (m, 1H), 6.52 (d, J = 7.5 Hz, 1H), 5.67 (dd, J = 7.1, 4.5 Hz, 1H), 4.66 – 4.62 (m, 1H), 4.60 – 4.57 (m, 1H), 4.54 – 4.50 (m, 1H).
^{13}C NMR (126 MHz, $CDCl_3$): δ 160.1, 147.8, 141.7, 130.5, 128.5, 127.6, 124.1, 123.0, 122.5, 112.0, 77.3, 63.7 (d, $J_{C\text{-}F}$ = 15.3 Hz), 48.3 (d, $J_{C\text{-}F}$ = 1.4 Hz), 42.4.
^{19}F NMR (471 MHz, $CDCl_3$): δ 50.5 (s, 1F).
HR-MS: *m/z* calcd. $[C_{16}H_{12}FNO_5S]^+$: 349.0420; found (EI+): 349.0419.

1-(4-nitrophenyl)-1,2,2a,7b-tetrahydrocyclobuta[*b*]benzofuran-2-sulfonyl fluoride (**3ga**-*endo*)

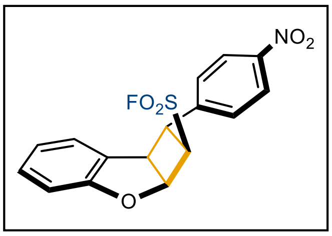

New compound, yellowish solid, 32.7 mg, 23% yield.
Purification conditions for column chromatography: hexanes:Et_2O 10:1 to 4:1
Purification conditions for recrystallization: pentane/Et_2O
R_f = 0.38 (acetone:hexanes = 1:4 v/v)
mp: 202 °C.
^{1}H NMR (500 MHz, $CDCl_3$): δ 8.32 – 8.30 (m, 2H), 7.54 – 7.52 (m, 2H), 7.29 – 7.23 (m, 2H), 7.29 – 7.23 (m, 1H), 7.04 (d, J = 8.1 Hz, 1H), 7.01 – 6.98 (m, 1H), 5.69 – 5.66 (m, 1H), 4.61 – 4.58 (m, 1H), 4.38 – 4.31 (m, 2H).
^{13}C NMR (126 MHz, $CDCl_3$): δ 161.6, 147.8, 145.4, 130.2, 127.7, 127.4, 124.7, 124.0, 122.5, 111.3, 78.2, 63.3 (d, $J_{C\text{-}F}$ = 14.4 Hz), 50.6, 48.4.
^{19}F NMR (471 MHz, $CDCl_3$): δ 57.8 (s, 1F).
HR-MS: *m/z* calcd. $[C_{16}H_{12}FNO_5S + H]^+$: 350.0493; found (FAB+): 350.0495.

1-(4-fluorophenyl)-1,2,2a,7b-tetrahydrocyclobuta[*b*]benzofuran-2-sulfonyl fluoride (**3ha**-*exo*)

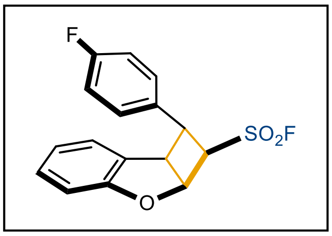

New compound, white crystal, 100.4 mg, 78% yield.
Purification conditions for column chromatography: hexanes:Et_2O 100:0 to 50:1
Purification conditions for recrystallization: pentane/Et_2O
R_f = 0.54 (acetone:hexanes = 1:6 v/v)
mp: 97 °C.
^{1}H NMR (500 MHz, $CDCl_3$): δ 7.22 – 7.19 (m, 1H), 7.00 – 6.94 (m, 5H), 6.79 – 6.76 (m, 1H), 6.54 (d, J = 7.5 Hz, 1H), 5.60 (dd, J = 7.1, 4.6 Hz, 1H), 4.55 – 4.49 (m, 2H), 4.41 – 4.37 (m, 1H).
^{13}C NMR (126 MHz, $CDCl_3$): δ 162.5 (d, $J_{C\text{-}F}$ = 247.3 Hz), 160.1, 130.3 (d, $J_{C\text{-}F}$ = 3.3 Hz), 130.1, 129.2 (d, $J_{C\text{-}F}$ = 8.2 Hz), 128.0, 123.6, 122.2, 115.8 (d, $J_{C\text{-}F}$ = 21.7 Hz), 111.7, 77.3, 64.3 (d, $J_{C\text{-}F}$ = 14.1 Hz), 48.5, 42.1.
^{19}F NMR (471 MHz, $CDCl_3$): δ 50.4 (s, 1F), -113.8 (s, 1F).
HR-MS: *m/z* calcd. $[C_{16}H_{12}F_2O_3S]^+$: 322.0475; found (EI+): 322.0471.

1-(4-fluorophenyl)-1,2,2a,7b-tetrahydrocyclobuta[*b*]benzofuran-2-sulfonyl fluoride (**3ha**-*endo*)

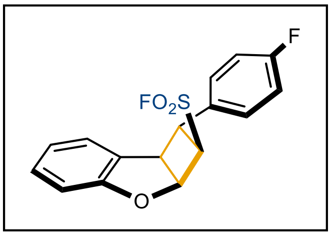

New compound, white solid, 26.7 mg, 21% yield.
Purification conditions for column chromatography: hexanes:Et_2O 50:1 to 20:1
Purification conditions for recrystallization: pentane/Et_2O
R_f = 0.51 (acetone:hexanes = 1:6 v/v)
mp: 140 °C.
^{1}H NMR (500 MHz, $CDCl_3$): δ 7.32 – 7.30 (m, 2H), 7.26 – 7.20 (m, 2H), 7.14 – 7.11 (m, 2H), 7.02 (d, J = 8.1 Hz, 1H), 6.98 – 6.95 (m, 1H), 5.65 – 5.62 (m, 1H), 4.52 – 4.49 (m, 1H), 4.26 – 4.20 (m, 2H).
^{13}C NMR (126 MHz, $CDCl_3$): δ 162.5 (d, J_{C-F} = 247.5 Hz), 161.5, 134.4 (d, J_{C-F} = 3.1 Hz), 129.8, 128.3, 128.1 (d, J_{C-F} = 8.2 Hz), 124.1, 122.3, 116.4 (d, J_{C-F} = 21.7 Hz), 111.0, 78.2, 64.2 (d, J_{C-F} = 13.4 Hz), 50.5, 48.6.
^{19}F NMR (471 MHz, $CDCl_3$): δ 57.7 (s, 1F), -113.6 (s, 1F).
HR-MS: *m/z* calcd. $[C_{16}H_{12}F_2O_3S]^+$: 322.0475; found (EI+): 322.0476.

1-(4-chlorophenyl)-1,2,2a,7b-tetrahydrocyclobuta[*b*]benzofuran-2-sulfonyl fluoride (**3ia**-*exo*)

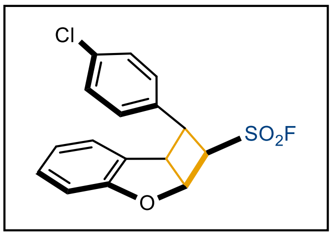

New compound, white solid, 110.3 mg, 81% yield.
Purification conditions for column chromatography: hexanes:Et_2O 100:0 to 50:1
Purification conditions for recrystallization: pentane/Et_2O
R_f = 0.54 (acetone:hexanes = 1:6 v/v)
mp: 111 °C.
^{1}H NMR (500 MHz, $CDCl_3$): δ 7.27 (d, J = 8.5 Hz, 2H), 7.23 – 7.19 (m, 1H), 6.96 – 6.93 (m, 3H), 6.80 – 6.77 (m, 1H), 6.55 (d, J = 7.4 Hz, 1H), 5.61 (dd, J = 7.0, 4.7 Hz, 1H), 4.56 – 4.49 (m, 2H), 4.40 – 4.36 (m, 1H).
^{13}C NMR (126 MHz, $CDCl_3$): δ 160.1, 134.2, 133.0, 130.1, 129.0, 128.9, 128.0, 123.5, 122.3, 111.7, 77.3, 64.1 (d, J_{C-F} = 14.4 Hz), 48.4 (d, J_{C-F} = 1.4 Hz), 42.2.
^{19}F NMR (471 MHz, $CDCl_3$): δ 50.5 (s, 1F).
HR-MS: *m/z* calcd. $[C_{16}H_{12}ClFO_3S]^+$: 338.0180; found (EI+): 338.0182.

1-(4-chlorophenyl)-1,2,2a,7b-tetrahydrocyclobuta[*b*]benzofuran-2-sulfonyl fluoride (**3ia**-*endo*)

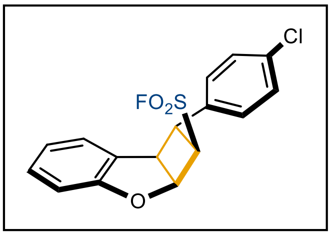

New compound, white solid, 24.8 mg, 18% yield.
R_f = 0.51 (acetone:hexanes = 1:6 v/v)
Purification conditions for column chromatography: hexanes:Et_2O 50:1 to 20:1
Purification conditions for recrystallization: pentane/Et_2O
mp: 129 °C.
^{1}H NMR (500 MHz, $CDCl_3$): δ 7.41 (d, J = 8.4 Hz, 2H), 7.28 – 7.20 (m, 4H), 7.02 (d, J = 8.1 Hz, 1H), 6.98 – 6.95 (m, 1H), 5.65 – 5.62 (m, 1H), 4.52 – 4.49 (m, 1H), 4.26 – 4.20 (m, 2H).
^{13}C NMR (126 MHz, $CDCl_3$): δ 161.6, 137.0, 134.2, 129.9, 129.6, 128.2, 127.8, 124.1, 122.3, 111.1, 78.2, 63.9 (d, J_{C-F} = 13.5 Hz), 50.5, 48.5.
^{19}F NMR (471 MHz, $CDCl_3$): δ 57.8 (s, 1F).
HR-MS: *m/z* calcd. $[C_{16}H_{12}ClFO_3S]^+$: 338.0180; found (EI+): 338.0177.

1-(4-bromophenyl)-1,2,2a,7b-tetrahydrocyclobuta[*b*]benzofuran-2-sulfonyl fluoride (**3ja**-*exo*)

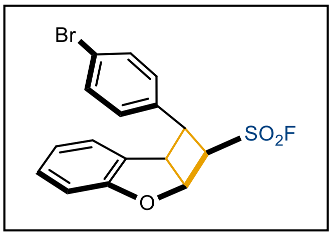

New compound, white solid, 123.1 mg, 80% yield.
Purification conditions for column chromatography: hexanes:Et_2O 100:0 to 50:1
Purification conditions for recrystallization: pentane/Et_2O
R$_f$ = 0.55 (acetone:hexanes = 1:6 v/v)
mp: 128 °C.
^{1}H NMR (500 MHz, $CDCl_3$): δ 7.42 (d, *J* = 8.4 Hz, 2H), 7.22 – 7.19 (m, 1H), 6.95 (d, *J* = 8.1 Hz, 1H), 6.87 (d, *J* = 8.4 Hz, 2H), 6.81 – 6.78 (m, 1H), 6.56 (d, *J* = 7.5 Hz, 1H), 5.61 (dd, *J* = 7.1, 4.6 Hz, 1H), 4.56 – 4.52 (m, 1H), 4.51 – 4.48 (m, 1H), 4.38 – 4.34 (m, 1H).
^{13}C NMR (126 MHz, $CDCl_3$): δ 160.1, 133.5, 131.9, 130.1, 129.2, 128.0, 123.5, 122.27, 122.26, 111.7, 77.3, 64.0 (d, $J_{C\text{-}F}$ = 14.4 Hz), 48.3 (d, $J_{C\text{-}F}$ = 1.5 Hz), 42.2.
^{19}F NMR (471 MHz, $CDCl_3$): δ 50.5 (s, 1F).
HR-MS: *m/z* calcd. $[C_{16}H_{12}BrFO_3S]^+$: 381.9675; found (EI+): 381.9676.

1-(4-bromophenyl)-1,2,2a,7b-tetrahydrocyclobuta[*b*]benzofuran-2-sulfonyl fluoride (**3ja**-*endo*)

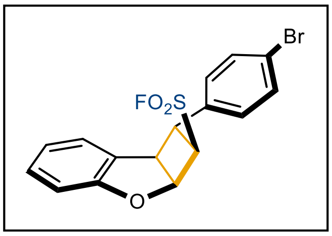

New compound, white solid, 27.8 mg, 18% yield.
Purification conditions for column chromatography: hexanes:Et_2O 50:1 to 20:1
Purification conditions for recrystallization: pentane/Et_2O
R$_f$ = 0.52 (acetone:hexanes = 1:6 v/v)
mp: 118 °C.
^{1}H NMR (500 MHz, $CDCl_3$): δ 7.56 (d, *J* = 8.4 Hz, 2H), 7.26 – 7.20 (m, 4H), 7.02 (d, *J* = 8.1 Hz, 1H), 6.98 – 6.95 (m, 1H), 5.65 – 5.62 (m, 1H), 4.52 – 4.49 (m, 1H), 4.25 – 4.18 (m, 2H).
^{13}C NMR (126 MHz, $CDCl_3$): δ 161.6, 137.6, 132.6, 129.9, 128.13, 128.10, 124.0, 122.4, 122.3, 111.1, 78.2, 63.8 (d, $J_{C\text{-}F}$ = 13.7 Hz), 50.6, 48.4.
^{19}F NMR (471 MHz, $CDCl_3$): δ 57.8 (s, 1F).
HR-MS: *m/z* calcd. $[C_{16}H_{12}BrFO_3S]^+$: 381.9675; found (EI+): 381.9677.

1-(4-(trifluoromethyl)phenyl)-1,2,2a,7b-tetrahydrocyclobuta[*b*]benzofuran-2-sulfonyl fluoride (**3ka**-*exo*)

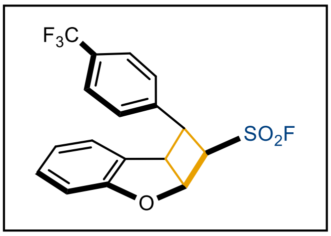

New compound, white crystal, 110.8 mg, 74% yield.
Purification conditions for column chromatography: hexanes:Et_2O 100:0 to 50:1
Purification conditions for recrystallization: pentane/Et_2O
R$_f$ = 0.52 (acetone:hexanes = 1:6 v/v)
mp: 102 °C.
^{1}H NMR (500 MHz, $CDCl_3$): δ 7.56 (d, *J* = 8.1 Hz, 2H), 7.23 – 7.20 (m, 1H), 7.14 (d, *J* = 8.1 Hz, 2H), 6.96 (d, *J* = 8.1 Hz, 1H), 6.79 – 6.76 (m, 1H), 6.53 (d, *J* = 7.5 Hz, 1H), 5.65 (dd, *J* = 7.0, 4.6 Hz, 1H), 4.61 – 4.55 (m, 2H), 4.49 – 4.45 (m, 1H).
^{13}C NMR (126 MHz, $CDCl_3$): δ 160.1, 138.5, 130.5 (d, $J_{C\text{-}F}$ = 32.8 Hz), 130.3, 127.9, 127.8, 125.8 (q, $J_{C\text{-}F}$ = 3.7 Hz), 124.0 (d, $J_{C\text{-}F}$ = 272.7 Hz), 123.3, 122.3, 111.8, 77.3, 63.8 (d, $J_{C\text{-}F}$ = 14.8 Hz), 48.3, 42.4.
^{19}F NMR (471 MHz, $CDCl_3$): δ 50.5 (s, 1F), -62.7 (s, 3F).
HR-MS: *m/z* calcd. $[C_{17}H_{12}F_4O_3S]^+$: 372.0443; found (EI+): 372.0444.

1-(4-(trifluoromethyl)phenyl)-1,2,2a,7b-tetrahydrocyclobuta[*b*]benzofuran-2-sulfonyl fluoride (**3ka**-*endo*)

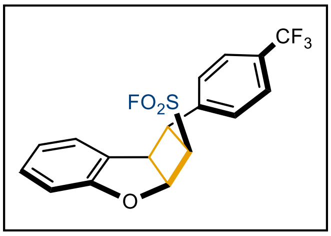

New compound, white crystal, 35.7 mg, 24% yield.
Purification conditions for column chromatography: hexanes:Et_2O 50:1 to 20:1
Purification conditions for recrystallization: pentane/Et_2O
R$_f$ = 0.49 (acetone:hexanes = 1:6 v/v)
mp: 113 °C.
^{1}H NMR (500 MHz, $CDCl_3$): δ 7.71 (d, *J* = 8.0 Hz, 2H), 7.47 (d, *J* = 8.0 Hz, 2H), 7.28 – 7.22 (m, 2H), 7.03 (d, *J* = 8.1 Hz, 1H), 7.00 – 6.97 (m, 1H), 5.67 – 5.65 (m, 1H), 4.58 – 4.55 (m, 1H), 4.32 – 4.28 (m, 2H).
^{13}C NMR (126 MHz, $CDCl_3$): δ 161.6, 142.4, 130.6 (d, $J_{C\text{-}F}$ = 32.7 Hz), 130.0, 128.0, 126.8, 126.5 (q, $J_{C\text{-}F}$ = 3.7 Hz), 124.1, 123.6 (d, $J_{C\text{-}F}$ = 144.3 Hz), 122.4, 111.2, 78.2, 63.6 (d, $J_{C\text{-}F}$ = 13.9 Hz), 50.7, 48.4.
^{19}F NMR (471 MHz, $CDCl_3$): δ 57.8 (s, 1F), -62.7 (s, 3F).
HR-MS: *m/z* calcd. $[C_{17}H_{12}F_4O_3S]^+$: 372.0443; found (EI+): 372.0441.

1-(3,5-bis(trifluoromethyl)phenyl)-1,2,2a,7b-tetrahydrocyclobuta[*b*]benzofuran-2-sulfonyl fluoride (**3la**-*exo*)

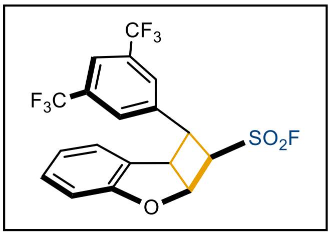

New compound, white crystal, 113.1 mg, 64% yield.
Purification conditions for column chromatography: hexanes:Et_2O 100:0 to 50:1
Purification conditions for recrystallization: pentane/Et_2O
R$_f$ = 0.54 (acetone:hexanes = 1:6 v/v)
mp: 105 °C.
^{1}H NMR (500 MHz, $CDCl_3$): δ 7.82 (s, 1H), 7.42 (s, 2H), 7.28 – 7.24 (m, 1H), 7.01 (d, *J* = 8.1 Hz, 1H), 6.82 – 6.79 (m, 1H), 6.46 (d, *J* = 7.5 Hz, 1H), 5.68 (dd, *J* = 7.1, 4.3 Hz, 1H), 4.67 – 4.64 (m, 1H), 4.57 – 4.49 (m, 2H).
^{13}C NMR (126 MHz, $CDCl_3$): δ 160.1, 137.3, 132.2 (q, $J_{C\text{-}F}$ = 33.6 Hz), 130.7, 128.0 (d, $J_{C\text{-}F}$ = 2.8 Hz), 127.7, 123.0 (d, $J_{C\text{-}F}$ = 272.9 Hz), 122.6, 122.5, 122.3 (dt, $J_{C\text{-}F}$ = 7.4, 3.7 Hz), 112.2, 77.3, 64.2 (d, $J_{C\text{-}F}$ = 15.4 Hz), 48.4, 42.4.
^{19}F NMR (471 MHz, $CDCl_3$): δ 50.3 (s, 1F), -63.0 (s, 6F).
HR-MS: *m/z* calcd. $[C_{18}H_{11}F_7O_3S]^+$: 440.0317; found (EI+): 440.0314.

1-(3,5-bis(trifluoromethyl)phenyl)-1,2,2a,7b-tetrahydrocyclobuta[*b*]benzofuran-2-sulfonyl fluoride (**3la**-*endo*)

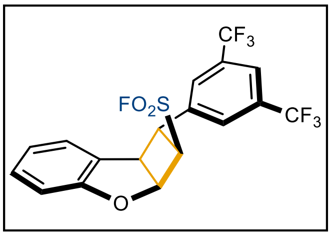

New compound, white solid, 33.6 mg, 19% yield.
Purification conditions for column chromatography: hexanes:Et_2O 50:1 to 20:1
Purification conditions for recrystallization: pentane/Et_2O
R$_f$ = 0.51 (acetone:hexanes = 1:6 v/v)
mp: 168 °C.
^{1}H NMR (500 MHz, $CDCl_3$): δ 7.90 (s, 1H), 7.78 (s, 2H), 7.30 – 7.27 (m, 1H), 7.24 – 7.23 (m, 1H), 7.05 (d, *J* = 8.1 Hz, 1H), 7.02 – 6.99 (m, 1H), 5.71 – 5.69 (m, 1H), 4.59 – 4.56 (m, 1H), 4.38 – 4.31 (m, 2H).
^{13}C NMR (126 MHz, $CDCl_3$): δ 161.6, 140.9, 133.1 (q, $J_{C\text{-}F}$ = 35.1 Hz), 130.3, 127.4, 126.7 (d, $J_{C\text{-}F}$ = 2.0 Hz), 124.1, 123.1 (d, $J_{C\text{-}F}$ = 273.0 Hz), 122.6, 122.5 (td, $J_{C\text{-}F}$ = 4.2, 1.3 Hz), 111.3, 78.1, 63.4 (d, $J_{C\text{-}F}$ = 14.4 Hz), 50.5, 48.4.
^{19}F NMR (471 MHz, $CDCl_3$): δ 57.9 (s, 1F), -62.9 (s, 6F).
HR-MS: *m/z* calcd. $[C_{18}H_{11}F_7O_3S]^+$: 440.0317; found (EI+): 440.0317.

1-([1,1'-biphenyl]-4-yl)-1,2,2a,7b-tetrahydrocyclobuta[*b*]benzofuran-2-sulfonyl fluoride (**3ma**-*exo*)

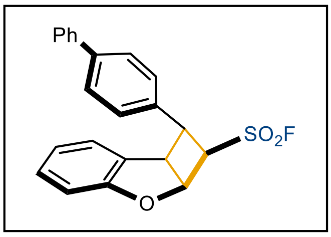

New compound, white solid, 133.5 mg, 88% yield.
Purification conditions for column chromatography: hexanes:Et_2O 100:0 to 55:1
Purification conditions for recrystallization: pentane/Et_2O
R$_f$ = 0.57 (acetone:hexanes = 1:6 v/v)
mp: 160 °C.
^{1}H NMR (500 MHz, $CDCl_3$): δ 7.56 (d, *J* = 7.4 Hz, 2H), 7.52 (d, *J* = 8.2 Hz, 2H), 7.44 – 7.41 (m, 2H), 7.36 – 7.33 (m, 1H), 7.21 – 7.18 (m, 1H), 7.07 (d, *J* = 8.1 Hz, 2H), 6.96 (d, *J* = 8.1 Hz, 1H), 6.78 – 6.75 (m, 1H), 6.60 (d, *J* = 7.5 Hz, 1H), 5.64 (dd, *J* = 7.0, 4.7 Hz, 1H), 4.61 – 4.56 (m, 2H), 4.47 – 4.43 (m, 1H).
^{13}C NMR (126 MHz, $CDCl_3$): δ 160.1, 140.9, 140.4, 133.4, 130.0, 129.0, 128.1, 127.9, 127.7, 127.4, 127.1, 123.9, 122.1, 111.6, 77.4, 64.1 (d, J_{C-F} = 14.0 Hz), 48.5 (d, J_{C-F} = 1.4 Hz), 42.5.
^{19}F NMR (471 MHz, $CDCl_3$): δ 50.5 (s, 1F).
HR-MS: *m/z* calcd. $[C_{22}H_{17}FO_3S]^+$: 380.0882; found (EI+): 380.0885.

1-(naphthalen-1-yl)-1,2,2a,7b-tetrahydrocyclobuta[*b*]benzofuran-2-sulfonyl fluoride (**3na**-*exo*)

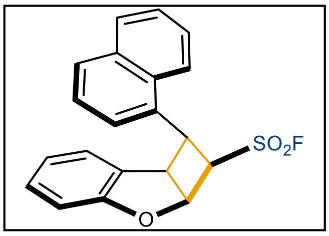

New compound, white solid, 92.6 mg, 65% yield.
Purification conditions for column chromatography: hexanes:Et_2O 100:0 to 40:1
Purification conditions for recrystallization: pentane/Et_2O
R$_f$ = 0.51 (acetone:hexanes = 1:6 v/v)
mp: 134 °C.
^{1}H NMR (500 MHz, $CDCl_3$): δ 8.04 (d, *J* = 8.4 Hz, 1H), 7.94 (d, *J* = 8.0 Hz, 1H), 7.78 (d, *J* = 8.3 Hz, 1H), 7.69 – 7.65 (m, 1H), 7.62 – 7.59 (m, 1H), 7.34 – 7.31 (m, 1H), 7.07 – 7.03 (m, 1H), 7.01 (d, *J* = 7.2 Hz, 1H), 6.88 (d, *J* = 8.1 Hz, 1H), 6.45 – 6.42 (m, 1H), 5.82 (d, *J* = 7.5 Hz, 1H), 5.76 (dd, *J* = 7.1, 4.6 Hz, 1H), 5.06 – 5.03 (m, 1H), 4.93 – 4.90 (m, 1H), 4.85 – 4.82 (m, 1H).
^{13}C NMR (126 MHz, $CDCl_3$): δ 159.9, 133.9, 130.9, 133.5, 129.8, 129.5, 128.6, 127.1, 126.8, 126.4, 125.4, 123.91, 123.87, 123.1, 122.0, 111.4, 77.7, 62.6 (d, J_{C-F} = 14.9 Hz), 48.7 (d, J_{C-F} = 1.4 Hz), 40.9.
^{19}F NMR (471 MHz, $CDCl_3$): δ 51.1 (s, 1F).
HR-MS: *m/z* calcd. $[C_{20}H_{15}FO_3S]^+$: 354.0726; found (EI+): 354.0727.

1-(naphthalen-2-yl)-1,2,2a,7b-tetrahydrocyclobuta[*b*]benzofuran-2-sulfonyl fluoride (**3oa**-*exo*)

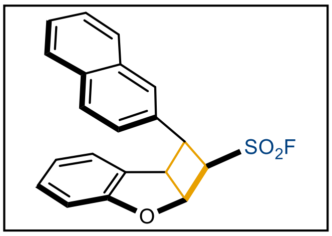

New compound, white solid, 109.4 mg, 77% yield.
Purification conditions for column chromatography: hexanes:Et_2O 100:0 to 40:1
Purification conditions for recrystallization: pentane/Et_2O
R$_f$ = 0.50 (acetone:hexanes = 1:6 v/v)
mp: 100 °C.
^{1}H NMR (500 MHz, $CDCl_3$): δ 7.82 – 7.78 (m, 2H), 7.73 – 7.70 (m, 1H), 7.50 – 7.44 (m, 3H), 7.18 – 7.13 (m, 2H), 6.96 (d, *J* = 8.1 Hz, 1H), 6.68 – 6.65 (m, 1H), 6.52 (d, *J* = 7.5 Hz, 1H), 5.68 (dd, *J* = 7.0, 4.6 Hz, 1H), 4.72 – 4.69 (m, 1H), 4.66 – 4.62 (m, 1H), 4.60 – 4.56 (m, 1H).
^{13}C NMR (126 MHz, $CDCl_3$): δ 160.1, 133.2, 132.9, 132.0, 129.9, 128.6, 128.04, 128.02, 127.8, 126.7, 126.5, 126.4, 125.2, 123.9, 122.1, 111.6, 77.5, 64.0 (d, J_{C-F} = 14.1 Hz), 48.5 (d, J_{C-F} = 1.6 Hz), 42.9.
^{19}F NMR (471 MHz, $CDCl_3$): δ 50.6 (s, 1F).
HR-MS: *m/z* calcd. $[C_{20}H_{15}FO_3S]^+$: 354.0726; found (EI+): 354.0723.

1-(thiophen-2-yl)-1,2,2a,7b-tetrahydrocyclobuta[*b*]benzofuran-2-sulfonyl fluoride (3pa-*exo*)

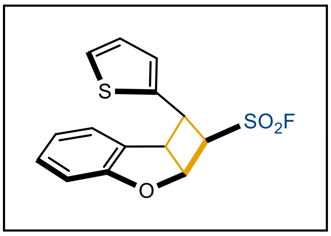

New compound, yellowish solid, 73.2 mg, 59% yield.
Purification conditions for column chromatography: hexanes:Et_2O 100:0 to 50:1
Purification conditions for recrystallization: pentane/Et_2O
R$_f$ = 0.56 (acetone:hexanes = 1:6 v/v)
mp: 84 °C.
^{1}H NMR (500 MHz, $CDCl_3$): δ 7.25 – 7.20 (m, 2H), 6.96 (d, J = 8.1 Hz, 1H), 6.91 (dd, J = 5.1, 3.6 Hz, 1H), 6.85 – 6.82 (m, 1H), 6.76 (d, J = 7.5 Hz, 1H), 6.67 (d, J = 3.5 Hz, 1H), 5.58 (dd, J = 7.0, 4.6 Hz, 1H), 4.62 – 4.59 (m, 1H), 4.57 – 4.53 (m, 1H), 4.47 – 4.44 (m, 1H).
^{13}C NMR (126 MHz, $CDCl_3$): δ 160.2, 137.6, 130.2, 128.1, 127.2, 126.2, 125.6, 123.6 122.2, 111.6, 77.2, 66.6 (d, J_{C-F} = 14.0 Hz), 49.3 (d, J_{C-F} = 1.8 Hz), 38.8.
^{19}F NMR (471 MHz, $CDCl_3$): δ 50.7 (s, 1F).
HR-MS: *m/z* calcd. $[C_{14}H_{11}FO_3S_2]^+$: 310.0134; found (EI+): 310.0137.

1-(thiophen-2-yl)-1,2,2a,7b-tetrahydrocyclobuta[*b*]benzofuran-2-sulfonyl fluoride (3pa-*endo*)

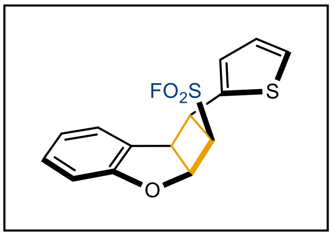

New compound, yellowish solid, 30.0 mg, 24% yield.
Purification conditions for column chromatography: hexanes:Et_2O 50:1 to 20:1
Purification conditions for recrystallization: pentane/Et_2O
R$_f$ = 0.53 (acetone:hexanes = 1:6 v/v)
mp: 82 °C.
^{1}H NMR (500 MHz, $CDCl_3$): δ 7.32 (dd, J = 5.0, 1.0 Hz, 1H), 7.25 – 7.22 (m, 2H), 7.05 – 7.00 (m, 3H), 6.98 – 6.95 (m, 1H), 5.67 – 5.64 (m, 1H), 4.55 – 4.53 (m, 1H), 4.44 – 4.41 (m, 1H), 4.31 – 4.29 (m, 1H).
^{13}C NMR (126 MHz, $CDCl_3$): δ 161.5, 141.9, 129.9, 127.84, 127.78, 125.6, 125.2, 124.2, 122.3, 111.0, 78.1, 65.3 (d, J_{C-F} = 13.6 Hz), 50.3 (d, J_{C-F} = 1.2 Hz), 46.7.
^{19}F NMR (471 MHz, $CDCl_3$): δ 58.4 (s, 1F).
HR-MS: *m/z* calcd. $[C_{14}H_{11}FO_3S_2]^+$: 310.0134; found (EI+): 310.0132.

1-(2,6-dichloropyridin-4-yl)-1,2,2a,7b-tetrahydrocyclobuta[*b*]benzofuran-2-sulfonyl fluoride (3qa-*exo*)

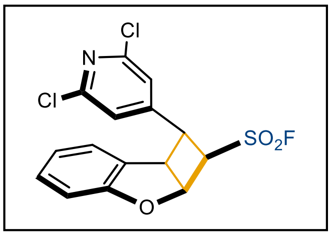

New compound, yellowish solid, 56.6 mg, 38% yield.
R$_f$ = 0.44 (acetone:hexanes = 1:4 v/v)
Purification conditions for column chromatography: hexanes:Et_2O 50:1 to 10:1
Purification conditions for recrystallization: pentane/Et_2O
mp: 143 °C.
^{1}H NMR (500 MHz, $CDCl_3$): δ 7.29 – 7.26 (m, 1H), 6.99 (d, J = 8.1 Hz, 1H), 6.94 (s, 2H), 6.89 – 6.86 (m, 1H), 6.65 (d, J = 7.5 Hz, 1H), 5.65 (dd, J = 7.1, 4.6 Hz, 1H), 4.63 – 4.60 (m, 1H), 4.50 – 4.47 (m, 1H), 4.37 – 4.33 (m, 1H).
^{13}C NMR (126 MHz, $CDCl_3$): δ 160.0, 151.4, 149.3, 130.9, 127.6, 122.7, 122.4, 121.7, 112.2, 77.3, 63.0 (d, J_{C-F} = 16.3 Hz), 47.9 (d, J_{C-F} = 1.2 Hz), 41.4.
^{19}F NMR (471 MHz, $CDCl_3$): δ 50.6 (s, 1F).
HR-MS: *m/z* calcd. $[C_{15}H_{10}Cl_2FNO_3S]^+$: 372.9742; found (EI+): 372.9741.

1-(2,6-dichloropyridin-4-yl)-1,2,2a,7b-tetrahydrocyclobuta[*b*]benzofuran-2-sulfonyl fluoride (**3qa**-*endo*)

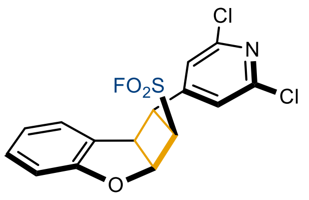

New compound, yellowish solid, 54.7 mg, 37% yield.
Purification conditions for column chromatography: hexanes:Et_2O 10:1 to 5:1
Purification conditions for recrystallization: pentane/Et_2O
R$_f$ = 0.42 (acetone:hexanes = 1:4 v/v)
mp: 190 °C.
^{1}H NMR (500 MHz, $CDCl_3$): δ 7.30 – 7.23 (m, 4H), 7.04 – 6.99 (m, 2H), 5.66 – 5.63 (m, 1H), 4.57 – 4.54 (m, 1H), 4.30 – 4.27 (m, 1H), 4.21 – 4.19 (m, 1H).
^{13}C NMR (126 MHz, $CDCl_3$): δ 161.5, 152.6, 152.0, 130.4, 127.0, 124.1, 122.7, 120.6, 111.3, 78.1, 111.2, 78.0, 62.4 (d, $J_{C\text{-}F}$ = 15.1 Hz), 49.3, 47.8.
^{19}F NMR (471 MHz, $CDCl_3$): δ 57.9 (s, 1F).
HR-MS: *m/z* calcd. [$C_{15}H_{10}Cl_2FNO_3S$ + Na]: 395.9640; found (EI+): 395.9639.

1-phenyl-1,2,2a,7b-tetrahydrobenzo[*b*]cyclobuta[*d*]thiophene-2-sulfonyl fluoride (**3ab**-*exo*)

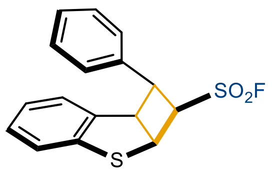

New compound, pinkish solid, 100.4 mg, 78% yield.
Purification conditions for column chromatography: hexanes:Et_2O 100:0 to 50:1
Purification conditions for recrystallization: pentane/Et_2O
R$_f$ = 0.54 (acetone:hexanes = 1:6 v/v)
mp: 82 °C.
^{1}H NMR (500 MHz, $CDCl_3$): δ 7.25 – 7.22 (m, 4H), 7.16 – 7.13 (m, 1H), 6.96 – 6.94 (m, 2H), 6.79 – 6.76 (m, 1H), 6.22 (d, *J* = 7.6 Hz, 1H), 4.75 – 4.73 (m, 2H), 4.66 – 4.56 (m, 2H).
^{13}C NMR (126 MHz, $CDCl_3$): δ 141.5, 135.0, 134.0, 129.1, 128.7, 128.2, 127.93, 127.88, 124.8, 123.1, 66.2 (d, $J_{C\text{-}F}$ = 12.2 Hz), 54.2 (d, $J_{C\text{-}F}$ = 1.6 Hz), 47.3, 44.4.
^{19}F NMR (471 MHz, $CDCl_3$): δ 49.5 (s, 1F).
HR-MS: *m/z* calcd. [$C_{16}H_{13}FO_2S_2$]$^+$: 320.0341; found (EI+): 320.0341.

1-phenyl-1,2,2a,7b-tetrahydrocyclobuta[*b*]benzofuran-2-sulfonyl fluoride (**3ab**-*endo*)

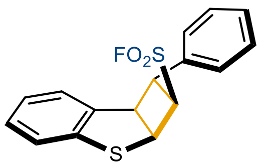

New compound, pinkish solid, 17.6 mg, 14% yield.
Purification conditions for column chromatography: hexanes:Et_2O 50:1 to 20:1
Purification conditions for recrystallization: pentane/Et_2O
R$_f$ = 0.51 (acetone:hexanes = 1:6 v/v)
mp: 114 °C.
^{1}H NMR (500 MHz, $CDCl_3$): δ 7.50 (d, *J* = 7.7 Hz, 1H), 7.42 – 7.39 (m, 2H), 7.36 – 7.27 (m, 5H), 7.20 – 7.17 (m, 1H), 4.92 – 4.88 (m, 1H), 4.69 – 4.64 (m, 1H), 4.38 – 4.33 (m, 2H).
^{13}C NMR (126 MHz, $CDCl_3$): δ 143.4, 139.6, 137.9, 129.4, 128.9, 128.3, 126.4, 125.2, 124.0, 122.4, 64.2 (d, $J_{C\text{-}F}$ = 10.9 Hz), 53.6, 51.1, 45.4 (d, $J_{C\text{-}F}$ = 1.3 Hz).
^{19}F NMR (471 MHz, $CDCl_3$): δ 57.8 (s, 1F).
HR-MS: *m/z* calcd. [$C_{16}H_{13}FO_2S_2$ + Na]: 343.0239; found (TOF MS ES+): 343.0242.

***tert*-butyl-2-(fluorosulfonyl)-1-phenyl-1,2,2a,7b-tetrahydro-3*H*-cyclobuta[*b*]indole-3-carboxylate** (**3ac**-*exo*)

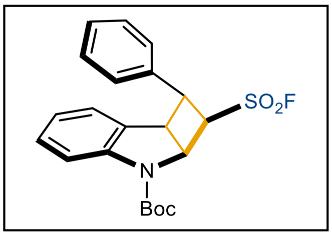

New compound, white solid, 84.2 mg, 52% yield.
Purification conditions for column chromatography: hexanes:Et_2O 100:0 to 40:1
Purification conditions for recrystallization: pentane/Et_2O
R$_f$ = 0.51 (acetone:hexanes = 1:6 v/v)
mp: 157 °C.
^{1}H NMR (500 MHz, $CDCl_3$): δ 7.92 (br s, 1H), 7.27 – 7.21 (m, 4H), 6.97 – 6.95 (m, 2H), 6.78 – 6.75 (m, 1H), 6.39 (d, J = 7.5 Hz, 1H), 5.43 (br s, 1H), 4.50 – 4.43 (m, 3H), 1.59 (s, 9H).
^{13}C NMR (126 MHz, $CDCl_3$): δ 151.1, 143.6, 134.5, 129.3, 128.7, 128.2, 127.78, 127.75, 127.2, 123.1, 116.0, 82.7, 64.5 (d, $J_{C\text{-}F}$ = 12.0 Hz), 57.2, 45.8, 43.9, 28.3.
^{19}F NMR (471 MHz, $CDCl_3$): δ 51.0 (s, 1F).
HR-MS: *m/z* calcd. $[C_{21}H_{22}FNO_4S]^+$: 403.1254; found (EI+): 403.1251.

***tert*-butyl-2-(fluorosulfonyl)-1-phenyl-1,2,2a,7b-tetrahydro-3*H*-cyclobuta[*b*]indole-3-carboxylate** (**3ac**-*endo*)

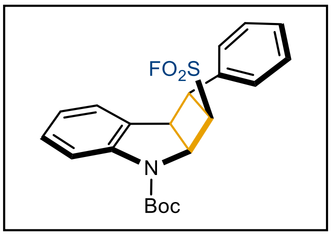

New compound, white solid, 37.3 mg, 23% yield.
Purification conditions for column chromatography: hexanes:Et_2O 40:1 to 18:1
Purification conditions for recrystallization: pentane/Et_2O
R$_f$ = 0.47 (acetone:hexanes = 1:6 v/v)
mp: 133 °C.
^{1}H NMR (500 MHz, $CDCl_3$): δ 8.02 (br s, 1H), 7.45 – 7.42 (m, 2H), 7.37 – 7.35 (m, 3H), 7.30 – 7.26 (m, 1H), 7.14 (d, J = 7.2 Hz, 1H), 7.03 – 7.00 (m, 1H), 5.42 (br s, 1H), 4.57 – 4.53 (m, 1H), 4.24 – 4.22 (m, 1H), 4.06 – 4.03 (m, 3H), 1.59 (s, 9H).
^{13}C NMR (126 MHz, $CDCl_3$): δ 151.7, 144.0, 138.9, 131.7, 129.4, 129.2, 128.2, 126.6, 123.7, 123.5, 116.1, 82.7, 63.6 (d, $J_{C\text{-}F}$ = 12.4 Hz), 57.6, 50.5, 46.6, 28.2.
^{19}F NMR (471 MHz, $CDCl_3$): δ 57.0 (s, 1F).
HR-MS: *m/z* calcd. $[C_{21}H_{22}FNO_4S]^+$: 403.1254; found (EI+): 403.1256.

7b-methyl-1-phenyl-1,2,2a,7b-tetrahydrocyclobuta[*b*]benzofuran-2-sulfonyl fluoride (**3ad**-*exo*)

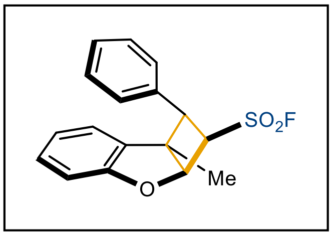

New compound, white crystal, 19.0 mg, 15% yield.
Purification conditions for column chromatography: hexanes:Et_2O 100:0 to 55:1
Purification conditions for recrystallization: pentane/Et_2O
R$_f$ = 0.55 (acetone:hexanes = 1:6 v/v)
mp: 115 °C.
^{1}H NMR (500 MHz, $CDCl_3$): δ 7.30 – 7.28 (m, 3H), 7.20 – 7.16 (m, 1H), 6.98 – 6.92 (m, 3H), 6.75 – 6.71 (m, 1H), 6.34 (dd, J = 7.5, 0.9 Hz, 1H), 5.24 (d, J = 4.6 Hz, 1H), 4.37 (dd, J = 10.0, 4.6 Hz, 1H), 4.04 (d, J = 10.0 Hz, 1H) 1.67 (s, 3H).
^{13}C NMR (126 MHz, $CDCl_3$): δ 159.3, 134.2, 129.8, 128.8, 128.4, 128.2, 127.2, 126.6, 122.1, 111.6, 82.6, 61.0 (d, $J_{C\text{-}F}$ = 14.1 Hz), 55.7 (d, $J_{C\text{-}F}$ = 1.7 Hz), 49.4, 24.8.
^{19}F NMR (471 MHz, $CDCl_3$): δ 50.5 (s, 1F).
HR-MS: *m/z* calcd. $[C_{17}H_{15}FO_3S]^+$: 318.0726; found (EI+): 318.0722.

7b-methyl-1-phenyl-1,2,2a,7b-tetrahydrocyclobuta[*b*]benzofuran-2-sulfonyl fluoride (**3ad**-*endo*)

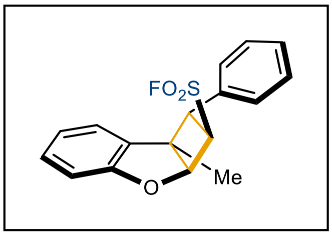

New compound, white crystal, 106.6 mg, 84% yield.
Purification conditions for column chromatography: hexanes:Et_2O 55:1 to 22:1
Purification conditions for recrystallization: pentane/Et_2O
R_f = 0.52 (acetone:hexanes = 1:6 v/v)
mp: 157 °C.
^{1}H NMR (500 MHz, $CDCl_3$): δ 7.44 – 7.41 (m, 2H), 7.36 – 7.33 (m, 1H), 7.25 – 7.23 (m, 1H), 7.19 – 7.14 (m, 3H), 7.02 – 6.99 (m, 2H), 5.16 (d, J = 6.1 Hz, 1H), 4.83 (dd, J = 9.6, 6.3 Hz, 1H), 4.43 (d, J = 9.8 Hz, 1H) 1.19 (s, 3H).
^{13}C NMR (126 MHz, $CDCl_3$): δ 161.4, 134.8, 134.1, 129.5, 129.2, 128.1, 127.0, 122.3, 121.8, 111.1, 84.0, 58.5 (d, $J_{C\text{-}F}$ = 13.3 Hz), 53.3, 52.4 (d, $J_{C\text{-}F}$ = 1.6 Hz), 17.1.
^{19}F NMR (471 MHz, $CDCl_3$): δ 58.9 (s, 1F).
HR-MS: *m/z* calcd. $[C_{17}H_{15}FO_3S]^+$: 318.0726; found (EI+): 318.0729.

7b-methyl-1-phenyl-1,2,2a,7b-tetrahydrobenzo[*b*]cyclobuta[*d*]thiophene-2-sulfonyl fluoride (**3ae**-*exo*)

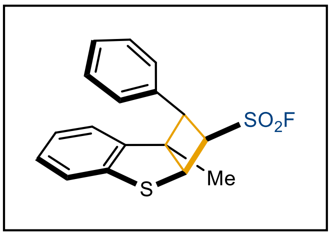

New compound, white solid, 25.2 mg, 19% yield.
Purification conditions for column chromatography: hexanes:Et_2O 100:0 to 55:1
Purification conditions for recrystallization: pentane/Et_2O
R_f = 0.55 (acetone:hexanes = 1:6 v/v)
mp: 109 °C.
^{1}H NMR (500 MHz, $CDCl_3$): δ 7.28 – 7.26 (m, 3H), 7.21 (d, J = 7.8 Hz, 1H), 7.16 – 7.13 (m, 1H), 6.95 – 6.94 (m, 2H), 6.78 – 6.75 (m, 3H), 6.03 (d, J = 7.7 Hz, 1H), 4.41 (dd, J = 10.1, 7.3 Hz, 1H), 4.31 (d, J = 7.1 Hz, 1H), 4.25 (d, J = 10.3 Hz, 1H), 1.67 (s, 3H).
^{13}C NMR (126 MHz, $CDCl_3$): δ 140.6, 139.3, 134.0, 129.2, 128.7, 128.3, 127.6, 127.1, 124.9, 123.2, 63.1 (d, $J_{C\text{-}F}$ = 12.4 Hz), 61.9 (d, $J_{C\text{-}F}$ = 2.0 Hz), 54.6, 51.0, 26.5.
^{19}F NMR (471 MHz, $CDCl_3$): δ 49.9 (s, 1F).
HR-MS: *m/z* calcd. $[C_{17}H_{15}FO_2S_2 + Na]$: 357.0395; found (TOF MS ES+): 357.0395.

7b-methyl-1-phenyl-1,2,2a,7b-tetrahydrobenzo[*b*]cyclobuta[*d*]thiophene-2-sulfonyl fluoride (**3ae**-*endo*)

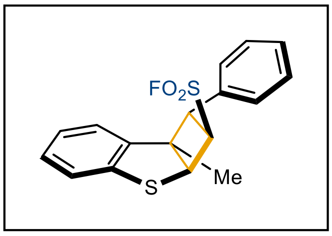

New compound, white crystal, 96.5 mg, 72% yield.
Purification conditions for column chromatography: hexanes:Et_2O 55:1 to 22:1
Purification conditions for recrystallization: pentane/Et_2O
R_f = 0.53 (acetone:hexanes = 1:6 v/v)
mp: 173 °C.
^{1}H NMR (500 MHz, $CDCl_3$): δ 7.41 – 7.39 (m, 2H), 7.35 – 7.32 (m, 1H), 7.27 – 7.21 (m, 2H), 7.16 – 7.11 (m, 3H), 6.97 (d, J = 7.5 Hz, 1H), 4.98 (dd, J = 10.2, 7.7 Hz, 1H), 4.57 (d, J = 10.4 Hz, 1H), 4.44 (d, J = 7.4 Hz, 1H), 1.25 (s, 3H).
^{13}C NMR (126 MHz, $CDCl_3$): δ 144.4, 142.9, 134.5, 129.1, 128.8, 128.1, 126.9, 125.3, 122.7, 122.4, 59.7 (d, $J_{C\text{-}F}$ = 11.3 Hz), 57.5 (d, $J_{C\text{-}F}$ = 2.5 Hz), 52.7, 51.7 (d, $J_{C\text{-}F}$ = 1.0 Hz), 19.5.
^{19}F NMR (471 MHz, $CDCl_3$): δ 60.7 (s, 1F).
HR-MS: *m/z* calcd. $[C_{17}H_{15}FO_2S_2 + Na]$: 357.0395; found (TOF MS ES+): 357.0393.

6-phenyl-2-oxabicyclo[3.2.0]hept-3-ene-7-sulfonyl fluoride (**3af**-*exo*)

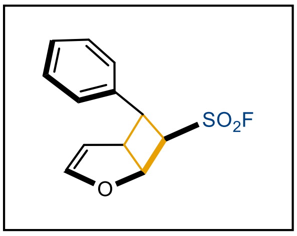

New compound, white crystal, 30.7 mg, 30% yield.
Purification conditions for column chromatography: hexanes:Et_2O 100:0 to 50:1
Purification conditions for recrystallization: pentane/Et_2O
R$_f$ = 0.54 (acetone:hexanes = 1:6 v/v)
mp: 101 °C.
^{1}H NMR (500 MHz, $CDCl_3$): δ 7.39 – 7.36 (m, 2H), 7.32 – 7.25 (m, 3H), 6.42 – 6.41 (m, 1H), 5.41 (dd, J = 7.4, 4.6 Hz, 1H), 4.98 – 4.97 (m, 1H), 4.65 (ddd, J = 9.5, 4.6, 0.9 Hz, 1H), 4.30 – 4.27 (m, 1H), 4.18 – 4.14 (m, 1H).
^{13}C NMR (126 MHz, $CDCl_3$): δ 148.2, 135.8, 128.9, 127.8, 126.9, 102.7, 76.6, 64.9 (d, $J_{C\text{-}F}$ = 14.0 Hz), 49.0 (d, $J_{C\text{-}F}$ = 1.7 Hz), 43.0.
^{19}F NMR (471 MHz, $CDCl_3$): δ 49.8 (s, 1F).
HR-MS: *m/z* calcd. [$C_{12}H_{11}FO_3S$ + Na]: 277.0311; found (TOF MS ES+): 277.0314.

6-phenyl-2-oxabicyclo[3.2.0]hept-3-ene-7-sulfonyl fluoride (**3af**-*endo*)

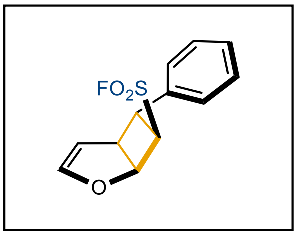

New compound, white solid, 14.7 mg, 14% yield.
Purification conditions for column chromatography: hexanes:Et_2O 50:1 to 20:1
Purification conditions for recrystallization: pentane/Et_2O
R$_f$ = 0.51 (acetone:hexanes = 1:6 v/v)
mp: 81 °C.
^{1}H NMR (500 MHz, $CDCl_3$): δ 7.42 – 7.39 (m, 2H), 7.33 – 7.28 (m, 3H), 6.622 – 6.619 (m, 1H), 5.44 – 5.41 (m, 1H), 5.37 – 5.36 (m, 1H), 4.50 – 4.47 (m, 1H), 4.15 – 4.12 (m, 1H), 3.83 – 3.80 (m, 1H).
^{13}C NMR (126 MHz, $CDCl_3$): δ 149.7, 139.2, 129.3, 128.0, 126.4, 104.0, 76.2, 65.9 (d, $J_{C\text{-}F}$ = 12.6 Hz), 51.6, 48.9.
^{19}F NMR (471 MHz, $CDCl_3$): δ 57.7 (s, 1F).
HR-MS: *m/z* calcd. [$C_{12}H_{11}FO_3S$ + Na]: 277.0311; found (TOF MS ES+): 277.0313.

***tert*-butyl 7b-(2-((tert-butoxycarbonyl)amino)ethyl)-2-(fluorosulfonyl)-1-phenyl-1,2,2a,7b-tetrahydro-3*H*-cyclobuta[*b*]indole-3-carboxylate** (**3ag**-*exo*)

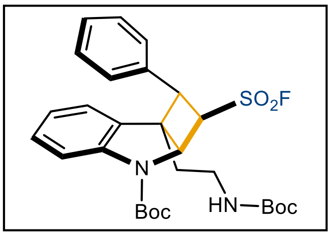

New compound, yellowish solid, 42.7 mg, 28% yield.
Purification conditions for column chromatography: hexanes:Et_2O 10:1 to 5:1
Purification conditions for recrystallization: pentane/Et_2O
R$_f$ = 0.45 (acetone:hexanes = 1:2 v/v)
mp: 123 °C.
^{1}H NMR (500 MHz, $CDCl_3$): δ 7.93 (bs, 1H), 7.29 – 7.23 (m, 4H), 6.91 (d, J = 6.8 Hz, 2H), 6.77 (t, J = 7.4 Hz, 1H), 6.19 (d, J = 7.4 Hz, 1H), 5.15 (bs, 1H), 4.38 – 4.30 (m, 2H), 4.12 – 4.10 (m, 1H), 2.93 (bs, 2H), 2.35 – 2.31 (m, 1H), 2.17 – 2.13 (m, 1H), 1.60 (s, 9H), 1.42 (s, 9H).
^{13}C NMR (126 MHz, $CDCl_3$): δ 155.9, 151.0, 143.3, 133.9, 129.7, 129.1, 128.7, 128.3, 127.8, 126.6, 123.3, 116.1, 83.0, 79.6, 61.8 (d, $J_{C\text{-}F}$ = 11.5 Hz), 61.5, 55.2, 50.0, 38.8, 36.7, 28.5, 28.3.
^{19}F NMR (471 MHz, $CDCl_3$): δ 51.2 (s, 1F).
HR-MS: *m/z* calcd. [$C_{28}H_{35}FN_2O_6S$ + Na]: 569.2098; found (TOF MS ES+): 569.2097.

***tert*-butyl 7b-(2-((tert-butoxycarbonyl)amino)ethyl)-2-(fluorosulfonyl)-1-phenyl-1,2,2a,7b-tetrahydro-3*H*-cyclobuta[*b*]indole-3-carboxylate** (**3ag**-*endo*)

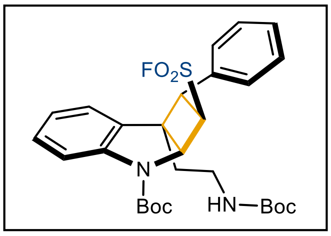

New compound, yellowish solid, 42.7 mg, 28% yield.
Purification conditions for column chromatography: hexanes:Et_2O 10:1 to 5:1
Purification conditions for recrystallization: pentane/Et_2O
R$_f$ = 0.39 (acetone:hexanes = 1:2 v/v)
mp: 110 °C.
^{1}H NMR (500 MHz, $CDCl_3$): δ 8.06 (bs, 1H), 7.41 – 7.39 (m, 2H), 7.35 – 7.33 (m, 1H), 7.30 – 7.28 (m, 1H), 7.20 – 7.19 (m, 2H), 7.07 – 7.05 (m, 2H), 5.10 – 4.89 (m, 2H), 4.34 (bs, 1H), 4.25 – 4.23 (m, 1H), 2.99 – 2.81 (m, 2H), 1.77 – 1.61 (m, 11H), 1.37 (s, 9H).
^{13}C NMR (126 MHz, $CDCl_3$): δ 155.8, 151.6, 144.5, 134.1, 130.5, 129.3, 129.1, 128.3, 127.4, 125.6, 123.6, 116.4, 83.2, 79.6, 61.6, 58.2 (d, $J_{C\text{-}F}$ = 11.0 Hz), 53.4, 36.7, 34.2, 30.8, 28.5, 28.2.
^{19}F NMR (471 MHz, $CDCl_3$): δ 58.9 (s, 1F).
HR-MS: *m/z* calcd. [$C_{28}H_{35}FN_2O_6S$ + Na]: 569.2098; found (TOF MS ES+): 569.2102.

2-(fluorosulfonyl)-1-phenyl-1,2,2a,7b-tetrahydrocyclobuta[*b*]benzofuran-6-yl)methyl acetate (**3ah**-*exo*)

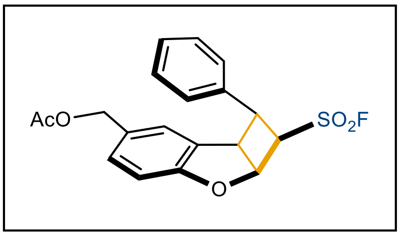

New compound, yellowish solid, 105.4 mg, 70% yield.
Purification conditions for column chromatography: hexanes:Et_2O 50:1 to 10:1
Purification conditions for recrystallization: pentane/Et_2O
R$_f$ = 0.25 (acetone:hexanes = 1:4 v/v)
mp: 115 °C.
^{1}H NMR (500 MHz, $CDCl_3$): δ 7.29 – 7.25 (m, 3H), 7.18 (d, *J* = 7.9 Hz, 1H), 7.00 – 6.98 (m, 2H), 6.92 – 6.90 (d, *J* = 7.9 Hz, 1H), 6.52 (s, 1H), 5.63 (dd, *J* = 6.9, 4.7 Hz, 1H), 4.81 (dd, *J* = 34.1, 12.1 Hz, 2H), 4.57 – 4.52 (m, 2H), 4.45 – 4.41 (m, 1H) 1.98 (s, 3H).
^{13}C NMR (126 MHz, $CDCl_3$): δ 170.7, 160.1, 134.2, 130.6, 130.0, 128.7, 128.5, 128.1, 127.3, 124.5, 111.4, 77.8, 65.8, 63.8 (d, $J_{C\text{-}F}$ = 14.3 Hz), 48.1 (d, $J_{C\text{-}F}$ = 1.1 Hz), 42.6, 21.1.
^{19}F NMR (471 MHz, $CDCl_3$): δ 50.5 (s, 1F).
HR-MS: *m/z* calcd. [$C_{19}H_{17}FO_5S$ + Na]: 399.0678; found (TOF MS ES+): 399.0682.

2-(fluorosulfonyl)-1-phenyl-1,2,2a,7b-tetrahydrocyclobuta[*b*]benzofuran-6-yl)methyl acetate (**3ah**-*endo*)

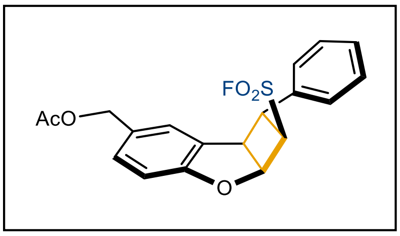

New compound, yellowish solid, 42.7 mg, 28% yield.
Purification conditions for column chromatography: hexanes:Et_2O 10:1 to 5:1
Purification conditions for recrystallization: pentane/Et_2O
R$_f$ = 0.22 (acetone:hexanes = 1:4 v/v)
mp: 104 °C.
^{1}H NMR (500 MHz, $CDCl_3$): δ 7.46 – 7.43 (m, 2H), 7.38 – 7.33 (m, 3H), 7.26 – 7.24 (m, 2H), 7.00 – 6.98 (m, 1H), 5.67 – 5.64 (m, 1H), 5.04 (dd, *J* = 26.6, 12.1 Hz, 2H), 4.57 – 4.54 (m, 1H), 4.29 – 4.26 (m, 2H), 2.08 (s, 3H).
^{13}C NMR (126 MHz, $CDCl_3$): δ 171.0, 161.7, 138.5, 130.6, 130.1, 129.5, 128.9, 128.3, 126.4, 124.8, 110.9, 78.7, 66.2, 64.1 (d, $J_{C\text{-}F}$ = 13.5 Hz), 50.9, 48.2 (d, $J_{C\text{-}F}$ = 0.6 Hz), 21.2.
^{19}F NMR (471 MHz, $CDCl_3$): δ 57.9 (s, 1F).
HR-MS: *m/z* calcd. [$C_{19}H_{17}FO_5S$ + Na]: 399.0678; found (TOF MS ES+): 399.0684.

methyl-2-(fluorosulfonyl)-1-phenyl-1,2,2a,7b-tetrahydrocyclobuta[*b*]benzofuran-6-carboxylate (**3ai**-*exo*)

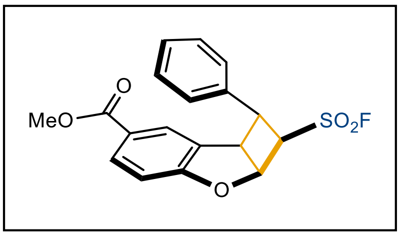

New compound, white solid, 30.6 mg, 21% yield.
Purification conditions for column chromatography: hexanes:Et_2O 50:1 to 20:1
Purification conditions for recrystallization: pentane/Et_2O
R$_f$ = 0.32 (acetone:hexanes = 1:6 v/v)
mp: 115 °C.
^{1}H NMR (500 MHz, $CDCl_3$): δ 7.93 (d, J = 8.4 Hz, 1H) 7.32 – 7.26 (m, 4H), 7.01 (d, J = 6.7 Hz, 2H), 6.97 (d, J = 8.5 Hz, 1H), 5.70 (dd, J = 6.8, 4.7 Hz, 1H), 4.59 – 4.55 (m, 2H), 4.50 – 4.46 (m, 1H), 3.79 (s, 3H).
^{13}C NMR (126 MHz, $CDCl_3$): δ 166.3, 164.0, 133.8, 132.5, 129.9, 129.0, 128.4, 127.2, 124.6, 124.56, 111.2, 78.4, 63.9 (d, J_{C-F} = 14.6 Hz), 52.1, 47.7 (d, J_{C-F} = 1.3 Hz), 42.7.
^{19}F NMR (471 MHz, $CDCl_3$): δ 50.5 (s, 1F).
HR-MS: *m/z* calcd. [$C_{18}H_{15}FO_5S$ + Na]: 385.0522; found (TOF MS ES+): 385.0523.

methyl-2-(fluorosulfonyl)-1-phenyl-1,2,2a,7b-tetrahydrocyclobuta[*b*]benzofuran-5-carboxylate (**3ai**-*exo*)

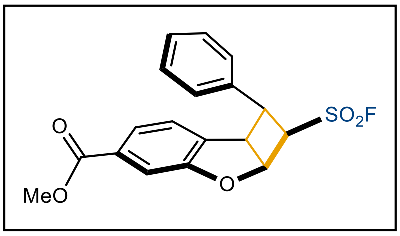

New compound, white solid, 92.8 mg, 64% yield.
Purification conditions for column chromatography: hexanes:Et_2O 50:1 to 20:1
Purification conditions for recrystallization: pentane/Et_2O
R$_f$ = 0.36 (acetone:hexanes = 1:6 v/v)
mp: 68 °C.
^{1}H NMR (500 MHz, $CDCl_3$): δ 7.58 (s, 1H), 7.45 (d, J = 7.8 Hz, 1H), 7.29 – 7.26 (m, 3H), 7.00 – 6.99 (m, 2H), 6.58 (d, J = 7.8 Hz, 1H), 5.68 (dd, J = 6.7, 4.8 Hz, 1H), 4.60 – 4.57 (m, 2H), 4.50 – 4.46 (m, 1H), 3.89 (s, 3H).
^{13}C NMR (126 MHz, $CDCl_3$): δ 166.4, 160.4, 133.9, 132.2, 129.4, 128.9, 128.3, 127.7, 127.2, 123.7, 112.4, 77.9, 63.7 (d, J_{C-F} = 14.5 Hz), 52.5, 48.2 (d, J_{C-F} = 1.2 Hz), 42.7.
^{19}F NMR (471 MHz, $CDCl_3$): δ 50.5 (s, 1F).
HR-MS: *m/z* calcd. [$C_{18}H_{15}FO_5S$ + Na]: 385.0522; found (TOF MS ES+): 385.0526.

methyl-2-(fluorosulfonyl)-1-phenyl-1,2,2a,7b-tetrahydrocyclobuta[*b*]benzofuran-5-carboxylate (**3aj**-*endo*)

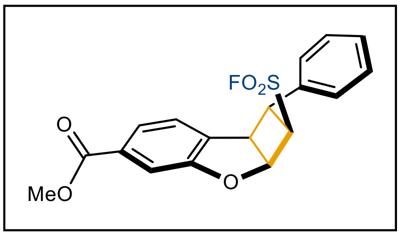

New compound, yellowish solid, 31.2 mg, 22% yield.
Purification conditions for column chromatography: hexanes:Et_2O 20:1 to 10:1
Purification conditions for recrystallization: pentane/Et_2O
R$_f$ = 0.32 (acetone:hexanes = 1:6 v/v)
mp: 151 °C.
^{1}H NMR (500 MHz, $CDCl_3$): δ 7.70 (dd, J = 7.7, 1.0 Hz, 1H), 7.65 (s, 1H), 7.46 – 7.43 (m, 2H), 7.38 – 7.33 (m, 3H), 7.28 (d, J = 7.7 Hz, 1H), 5.72 – 5.69 (m, 1H), 4.59 – 4.56 (m, 1H), 4.34 – 4.31 (m, 1H), 4.26 – 4.23 (m, 1H), 3.92 (s, 3H).
^{13}C NMR (126 MHz, $CDCl_3$): δ 166.5, 161.8, 138.3, 133.6, 132.0, 129.5, 128.4, 126.3, 124.3, 123.9, 111.9, 78.7, 64.1 (d, J_{C-F} = 13.7 Hz), 52.4, 50.8, 48.3.
^{19}F NMR (471 MHz, $CDCl_3$): δ 58.0 (s, 1F).
HR-MS: *m/z* calcd. [$C_{18}H_{15}FO_5S$ + Na]: 385.0522; found (TOF MS ES+): 385.0529.

6-methyl-1-phenyl-1,2,2a,7b-tetrahydrobenzo[*b*]cyclobuta[*d*]thiophene-2-sulfonyl fluoride (**3ak**-*exo*)

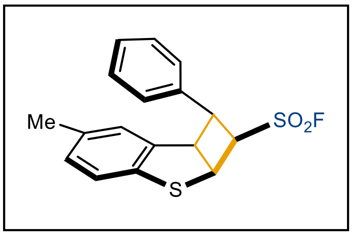

New compound, white solid, 92.8 mg, 69% yield.
Purification conditions for column chromatography: hexanes:Et_2O 100:0 to 50:1
Purification conditions for recrystallization: pentane/Et_2O
R_f = 0.53 (acetone:hexanes = 1:6 v/v)
mp: 105 °C.
^{1}H NMR (500 MHz, $CDCl_3$): δ 7.26 – 7.23 (m, 3H), 7.10 (d, J = 8.0 Hz, 1H), 6.97 – 6.93 (m, 3H), 6.00 (s, 1H), 4.75 – 4.67 (m, 2H), 4.62 – 4.55 (m, 2H), 1.99 (s, 3H).
^{13}C NMR (126 MHz, $CDCl_3$): δ 137.8, 135.1, 134.6, 134.2, 129.9, 128.8, 128.5, 128.2, 128.0, 122.8, 66.2 (d, J_{C-F} = 12.1 Hz), 54.1 (d, J_{C-F} = 1.7 Hz), 47.2, 44.7, 20.8.
^{19}F NMR (471 MHz, $CDCl_3$): δ 49.4 (s, 1F).
HR-MS: *m/z* calcd. [$C_{17}H_{15}FO_2S_2$ + Na]: 357.0395; found (TOF MS ES+): 357.0396.

6-bromo-1-phenyl-1,2,2a,7b-tetrahydrobenzo[*b*]cyclobuta[*d*]thiophene-2-sulfonyl fluoride (**3al**-*exo*)

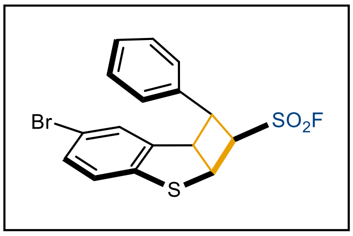

New compound, light brown solid, 107.3 mg, 67% yield.
Purification conditions for column chromatography: hexanes:Et_2O 100:0 to 50:1
Purification conditions for recrystallization: pentane/Et_2O
R_f = 0.53 (acetone:hexanes = 1:6 v/v)
mp: 96 °C.
^{1}H NMR (500 MHz, $CDCl_3$): δ 7.32 – 7.30 (m, 3H), 7.27 – 7.25 (m, 1H), 7.10 – 7.08 (m, 1H), 6.97 – 6.94 (m, 2H), 6.29 (s, 1H), 4.78 – 4.75 (m, 1H), 4.73 – 4.69 (m, 1H), 4.65 – 4.58 (m, 2H).
^{13}C NMR (126 MHz, $CDCl_3$): δ 140.8, 137.5, 133.5, 132.1, 130.9, 128.8, 128.6, 127.7, 124.3, 118.2, 65.9 (d, J_{C-F} = 12.8 Hz), 54.0 (d, J_{C-F} = 1.7 Hz), 47.3, 44.9.
^{19}F NMR (471 MHz, $CDCl_3$): δ 49.5 (s, 1F).
HR-MS: *m/z* calcd. [$C_{16}H_{12}BrF_2O_2S_2$]$^+$: 397.9446; found (EI+): 397.9449.

1D-NOESY NMR experiments to determine the *exo-/endo*-configurations

1D-NOESY spectra of 3aa-*exo*

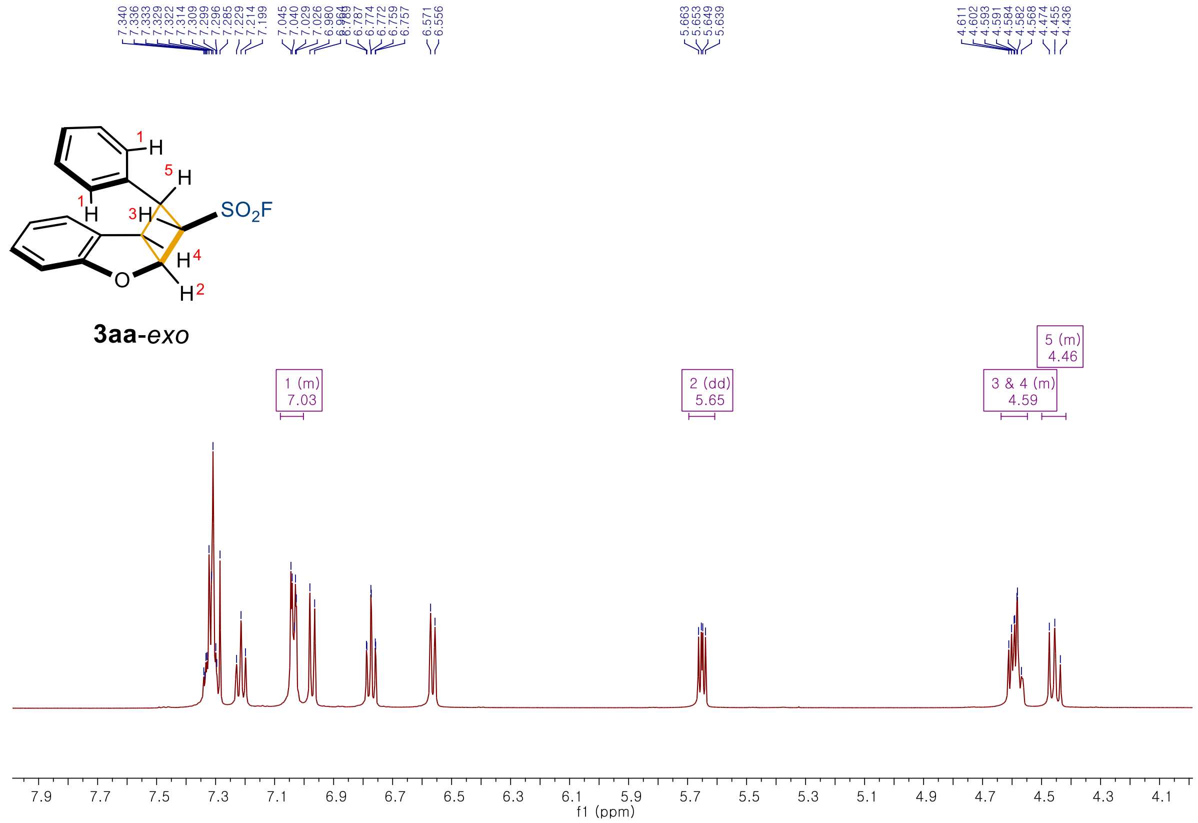

Supplementary Figure 2 ^{1}H NMR spectrum of **3aa**-*exo* (500 MHz, $CDCl_3$, 25 °C).

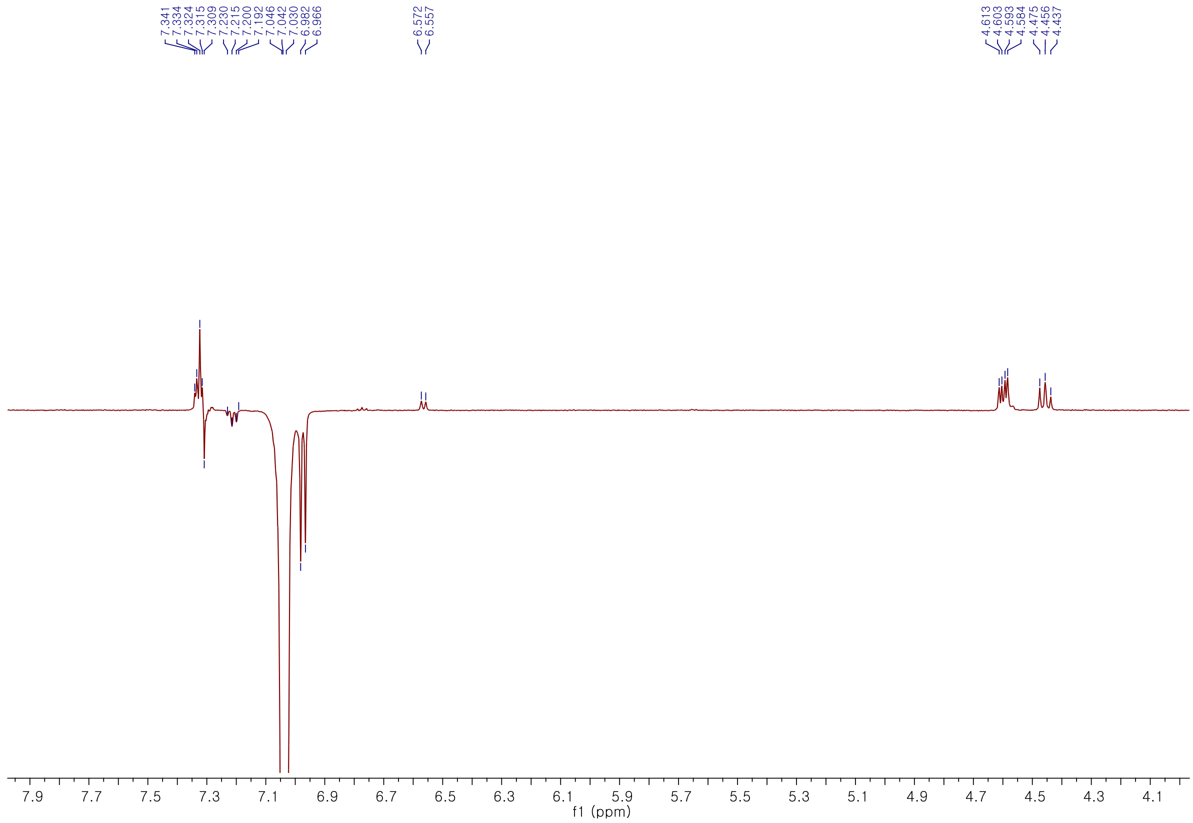

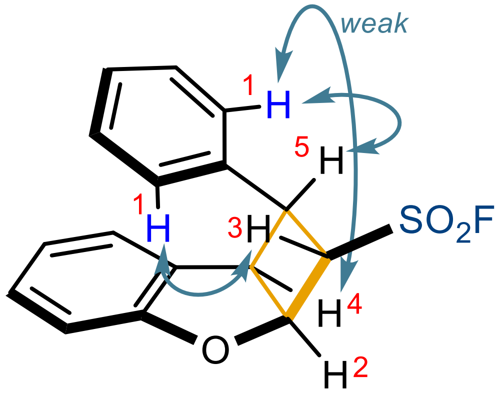

Supplementary Figure 3 1D-NOESY NMR analysis for proton 1 of **3aa**-*exo* (500 MHz, $CDCl_3$, 25 °C).

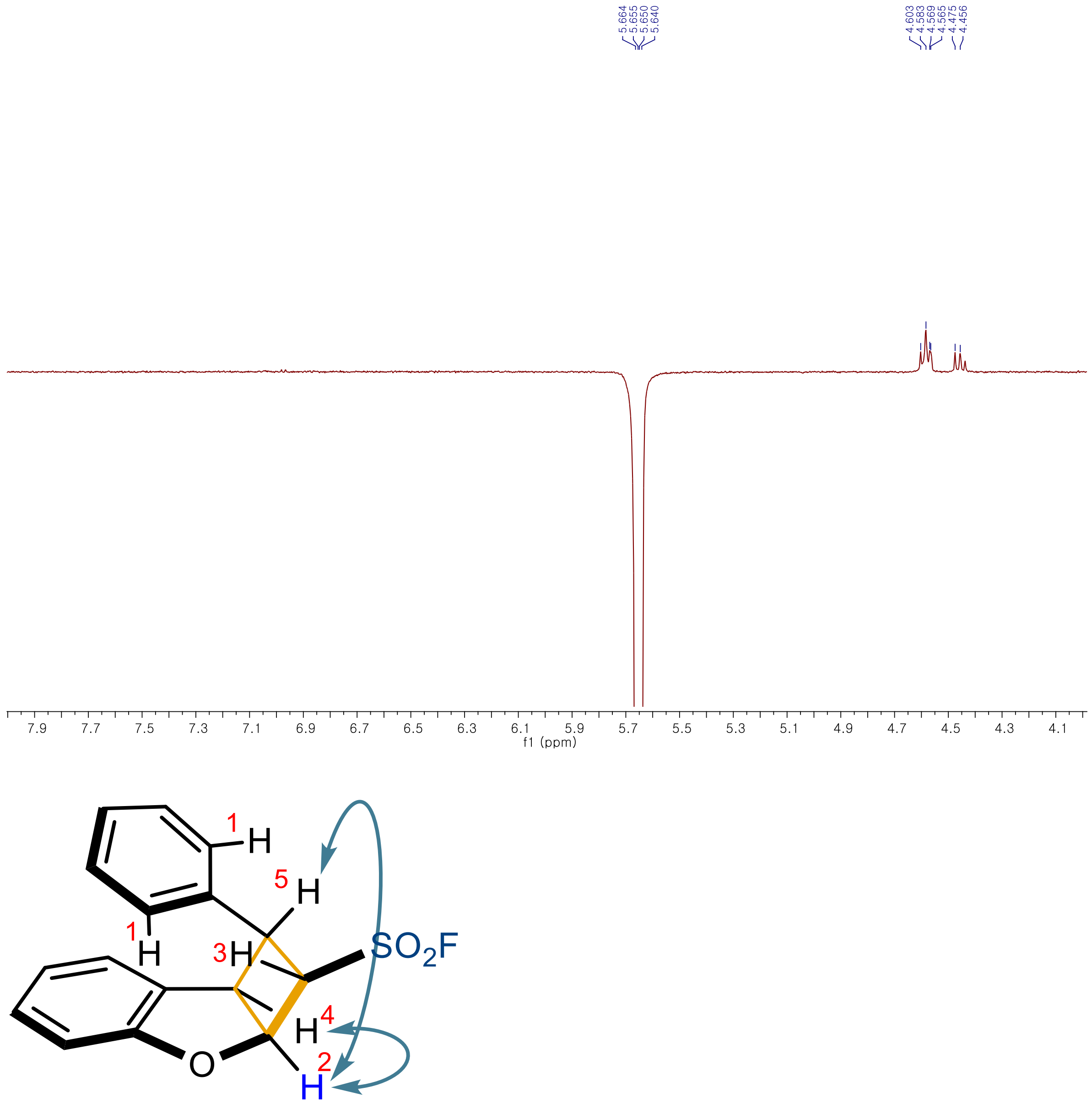

Supplementary Figure 4 1D-NOESY NMR analysis for proton 2 of **3aa**-*exo* (500 MHz, $CDCl_3$, 25 °C).

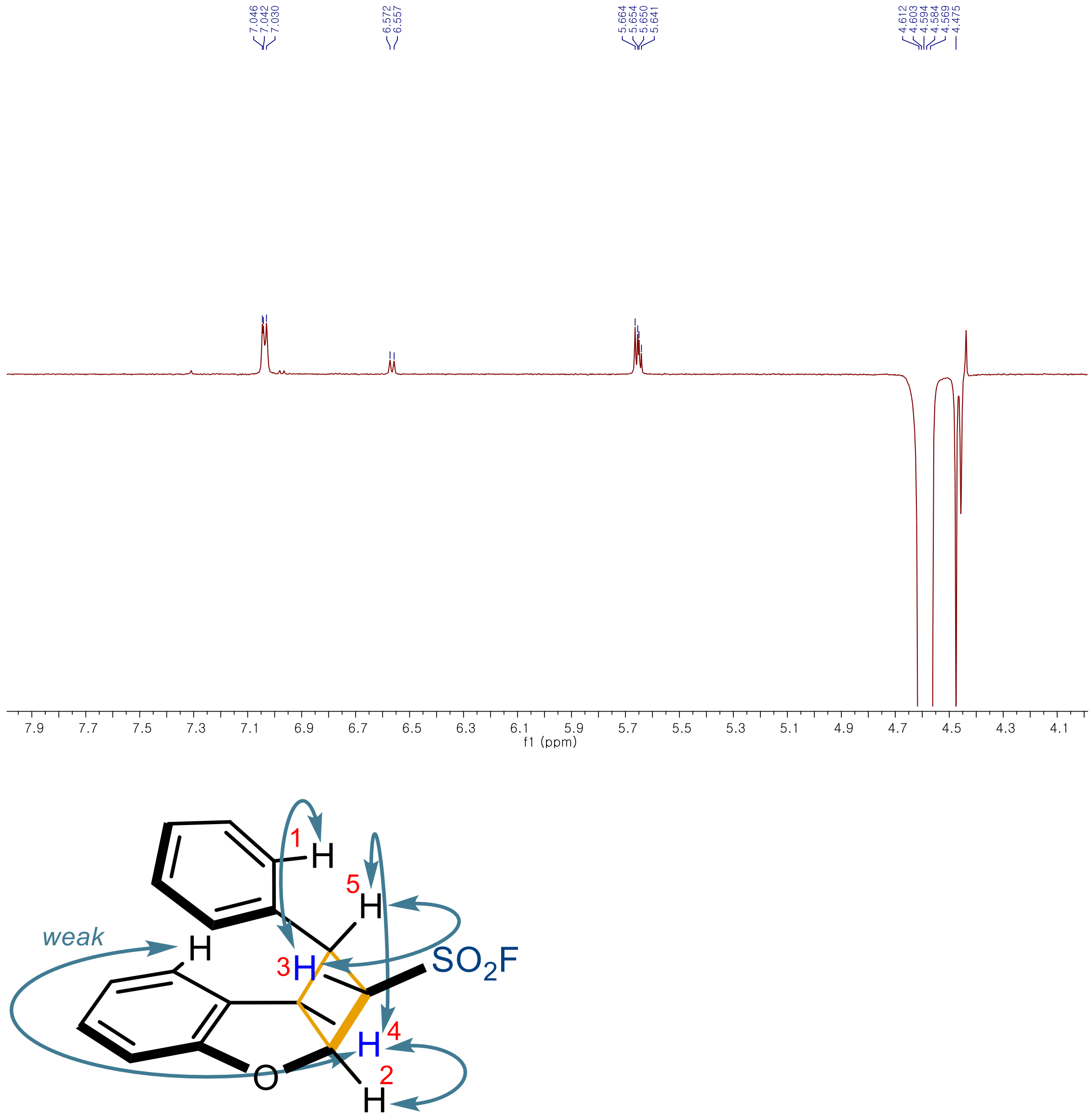

Supplementary Figure 5 1D-NOESY NMR analysis for proton 3 and 4 of **3aa**-*exo* (500 MHz, $CDCl_3$, 25 °C).

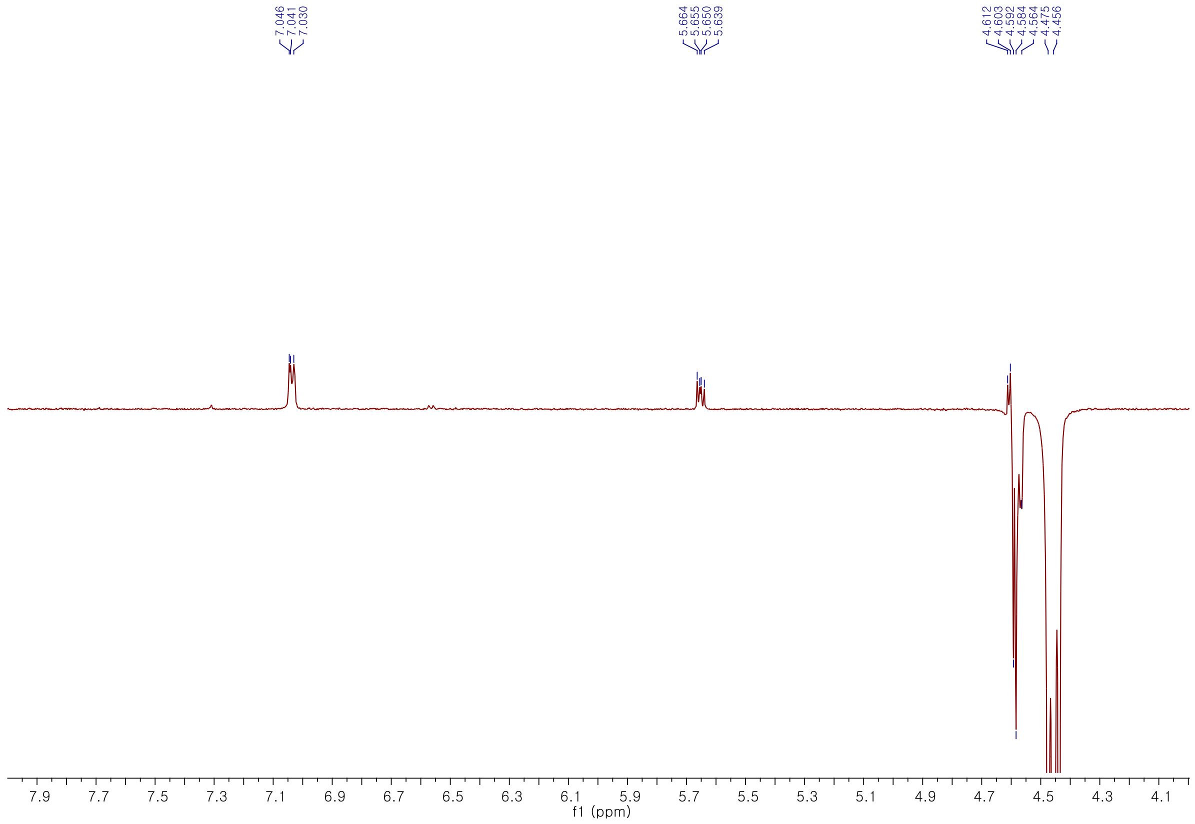

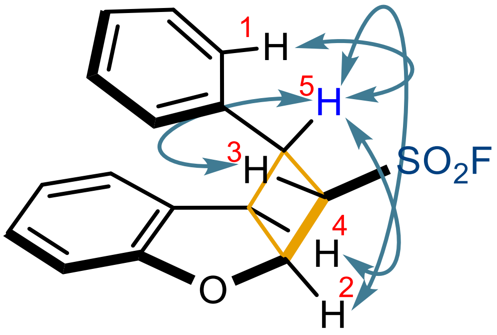

Supplementary Figure 6 1D-NOESY NMR analysis for proton 5 of **3aa**-*exo* (500 MHz, $CDCl_3$, 25 °C).

Comment: Based on the 1D-NOESY NMR analysis, it is evident that the proton 2 does not influence the peak around δ = 4.16 ppm, corresponding to the proton 3 of the cyclobutane moiety. Additionally, proton 5 affected protons 2, 3, and 4. If this product was *endo*-configuration, proton 5 would not interact with proton 2 due to its relatively far distance. These results strongly suggests that the proton 2 and the proton 3 are oriented in the opposite direction, and proton 5 is also oriented in the opposite direction with benzofuran moiety. Consequently, the product is determined as *exo*-configuration (further matched with single crystal X-ray analysis).

1D-NOESY spectra of 3aa-*endo*

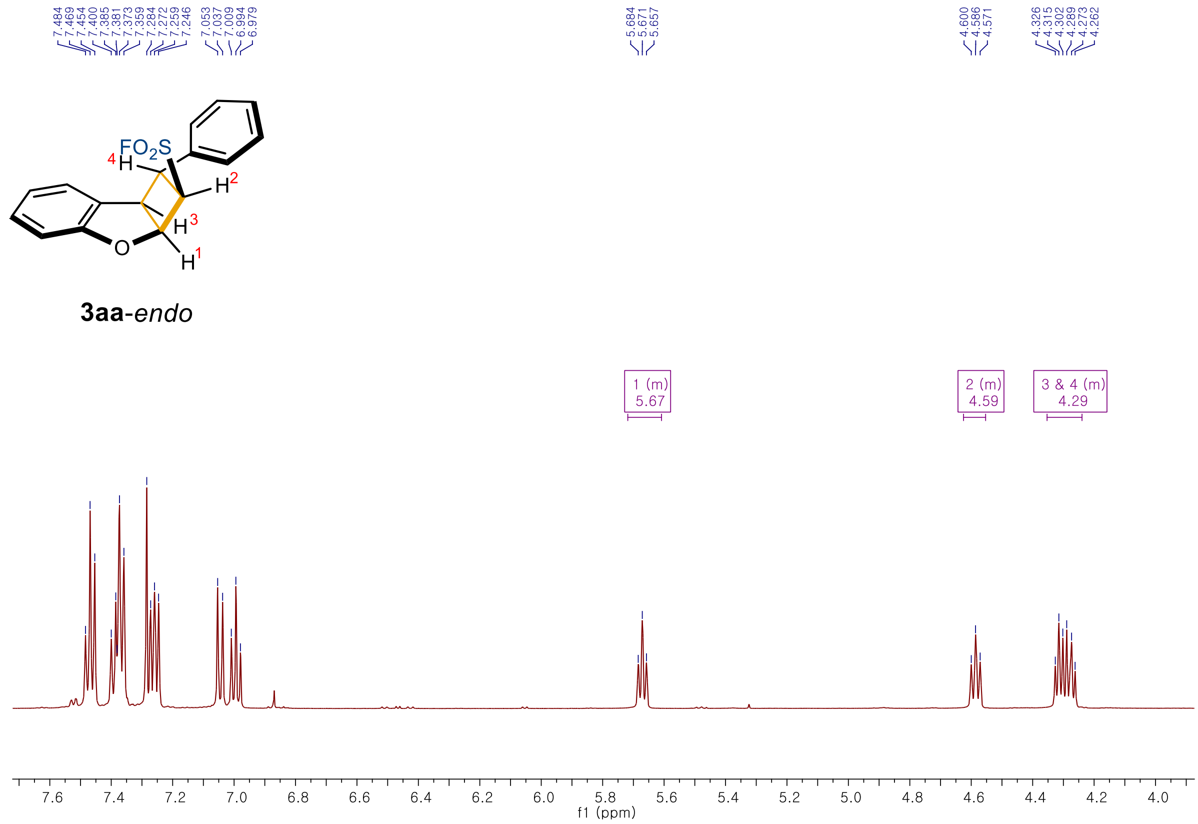

Supplementary Figure 7 ^{1}H NMR spectrum of **3aa**-*endo* (500 MHz, $CDCl_3$, 25 °C).

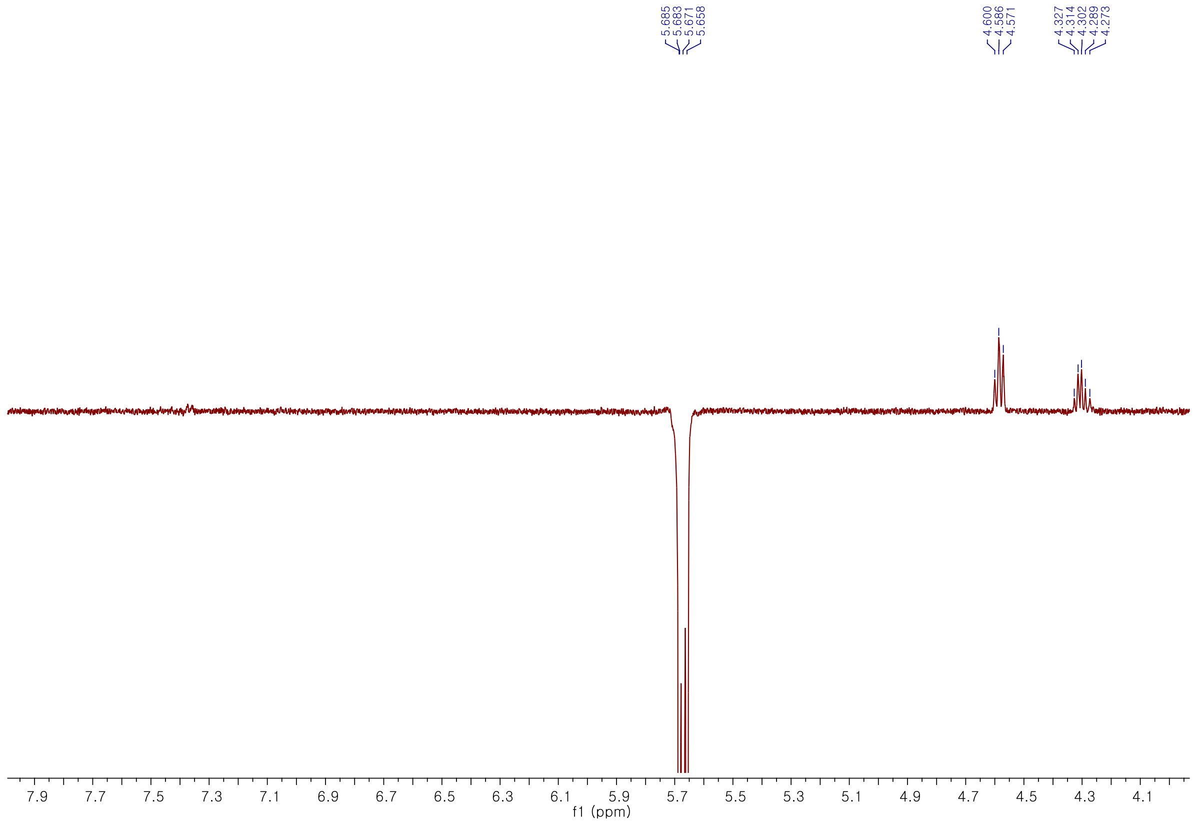

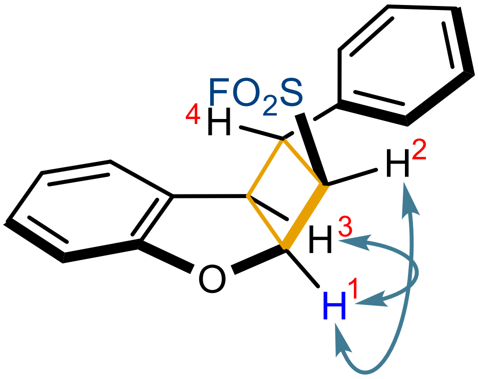

Supplementary Figure 8 1D-NOESY analysis for proton 1 of **3aa**-*endo* (500 MHz, $CDCl_3$, 25 °C).

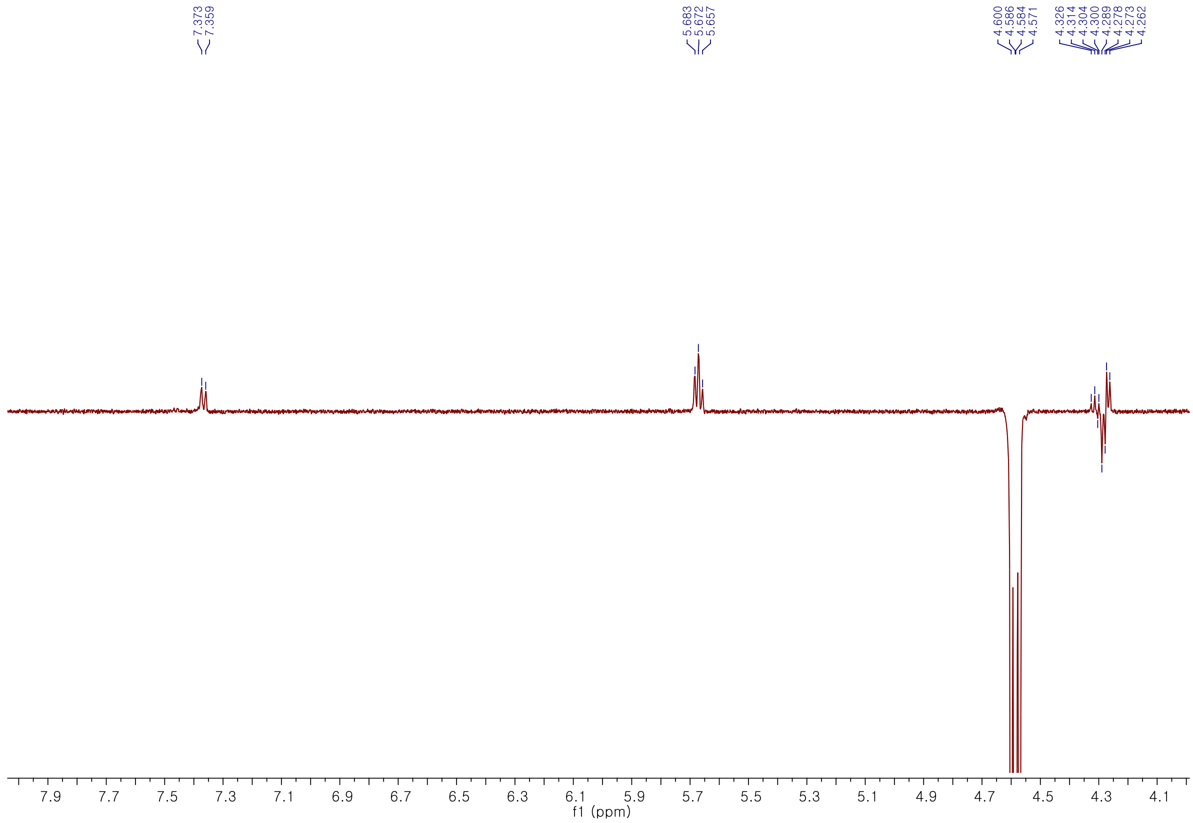

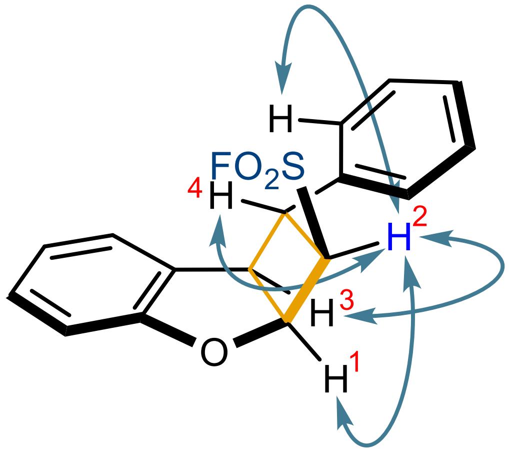

Supplementary Figure 9 1D-NOESY analysis for proton 2 of **3aa**-*endo* (500 MHz, $CDCl_3$, 25 °C).

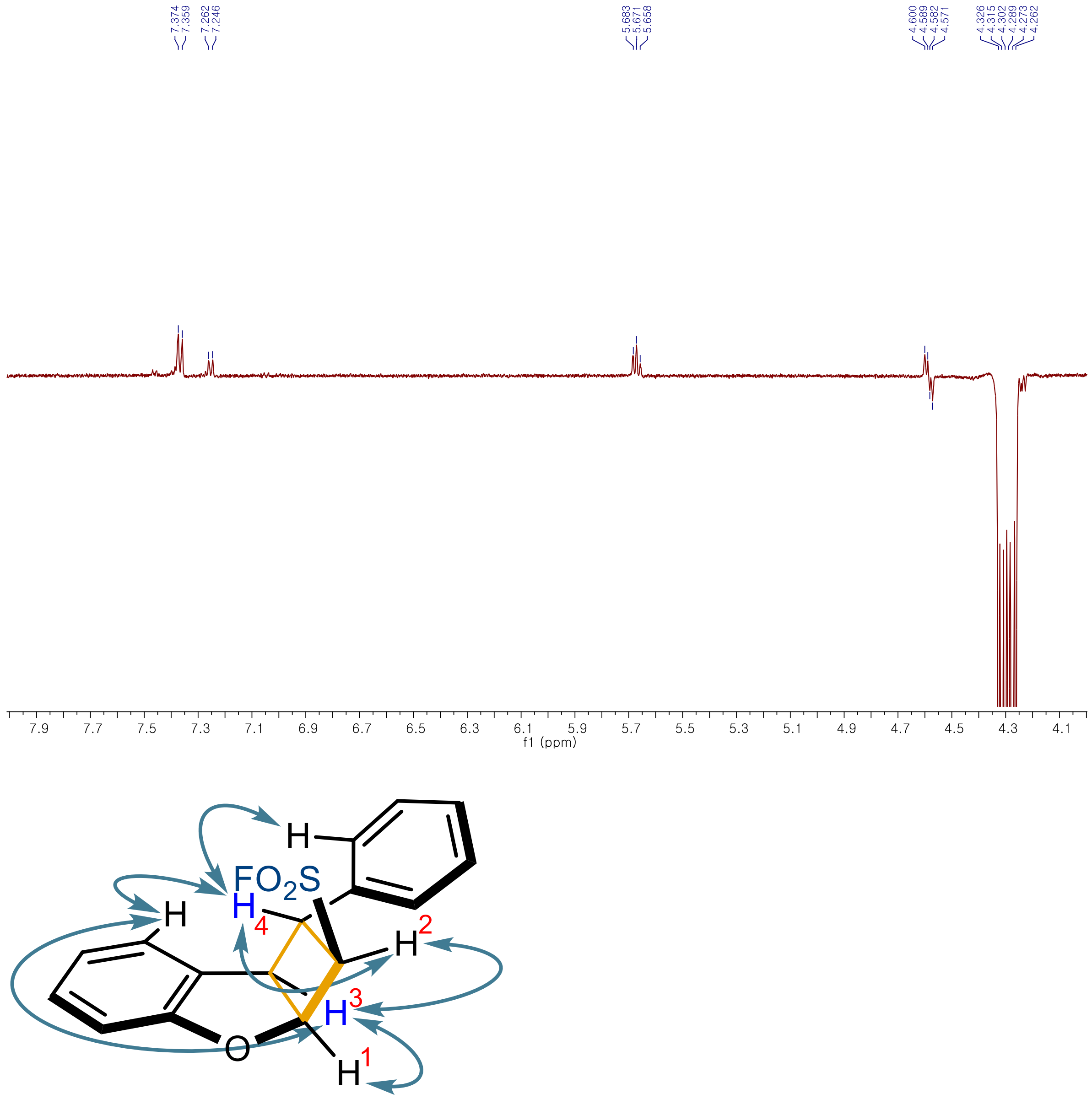

Supplementary Figure 10 1D-NOESY analysis for proton 3 and 4 of **3aa**-*endo* (500 MHz, $CDCl_3$, 25 °C).

Comment: Based on the 1D-NOESY NMR analysis, proton 1 has no effect on the proton 4 because it is relatively far away. Moreover, the proton 1 is interacted with the proton 2 unlike the *exo*-structure. These results strongly suggests that the proton 1 is oriented in the same direction with proton 2, and the proton 4 is oriented in the opposite direction with proton 1. Additionally, **Supplementary Figure 10** shows that the proton 4 is close enough to affect the proton peak around $\delta = 7.25$ ppm, a C(sp2)–H of benzofuran moiety. Therefore, the product's structure is determined to be *endo*-configuration, in which proton 4 is in the same direction as the oxygen atom (ether moiety). However, the proton 2 is in the opposite direction to the oxygen atom (further matched with single crystal X-ray analysis).

1D-NOESY spectra of 3ma-*exo*

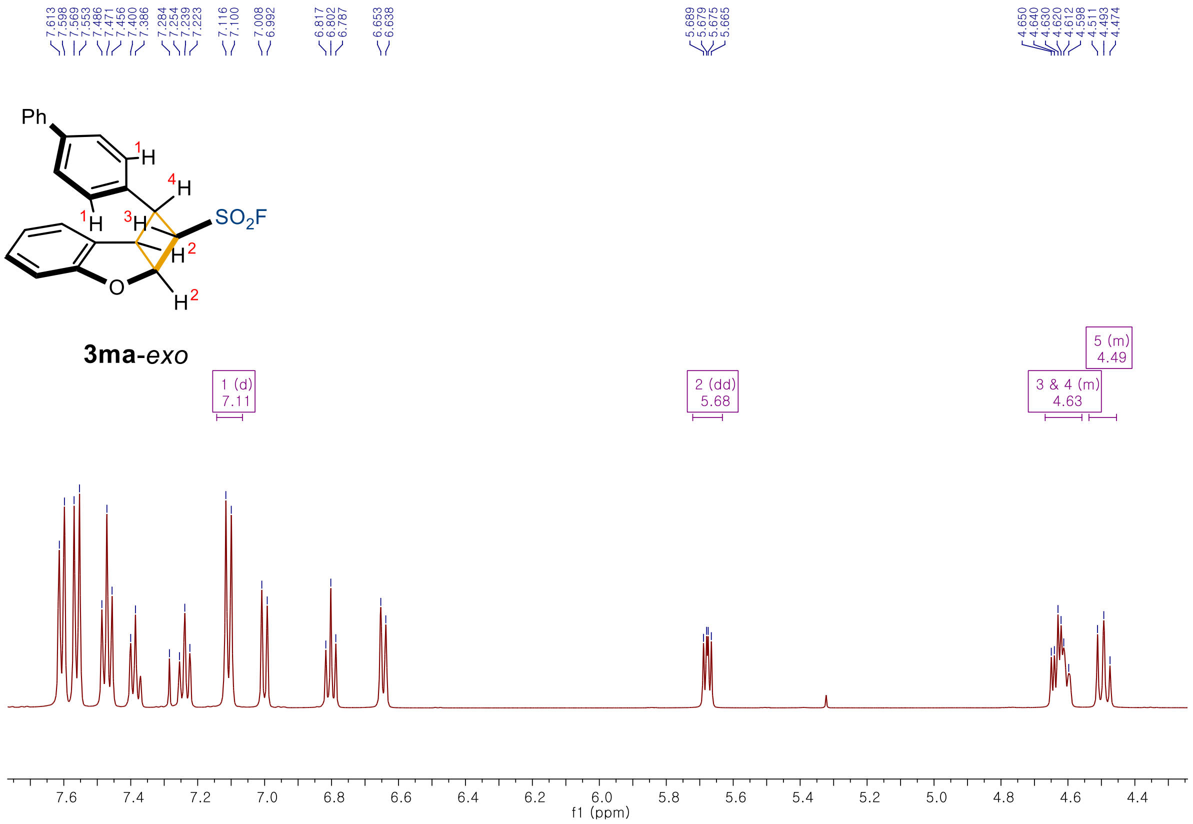

Supplementary Figure 11 ^{1}H NMR spectrum of **3ma**-*exo* (500 MHz, $CDCl_3$, 25 °C).

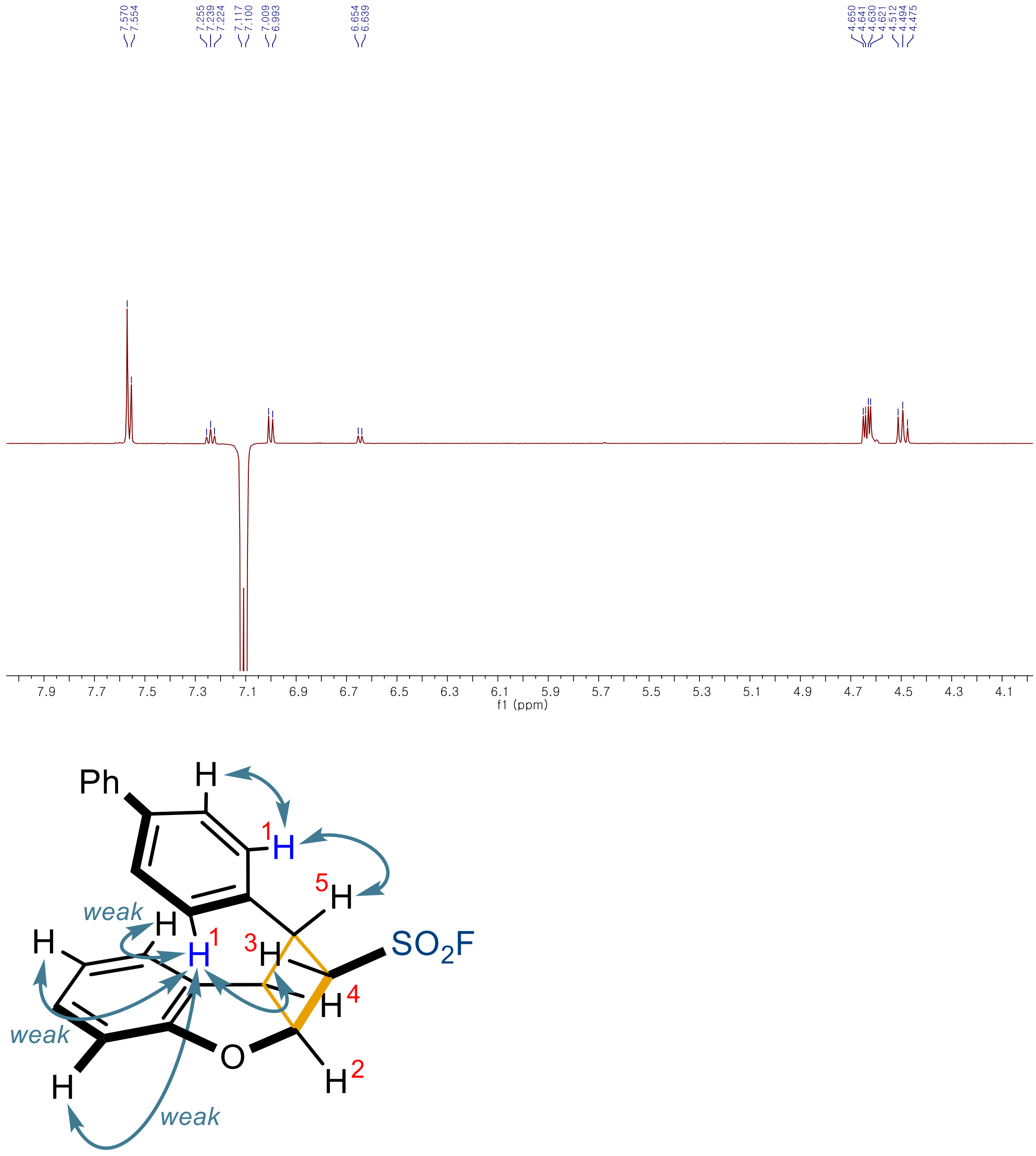

Supplementary Figure 12 1D-NOESY analysis for proton 1 of **3ma**-*exo* (500 MHz, $CDCl_3$, 25 °C).

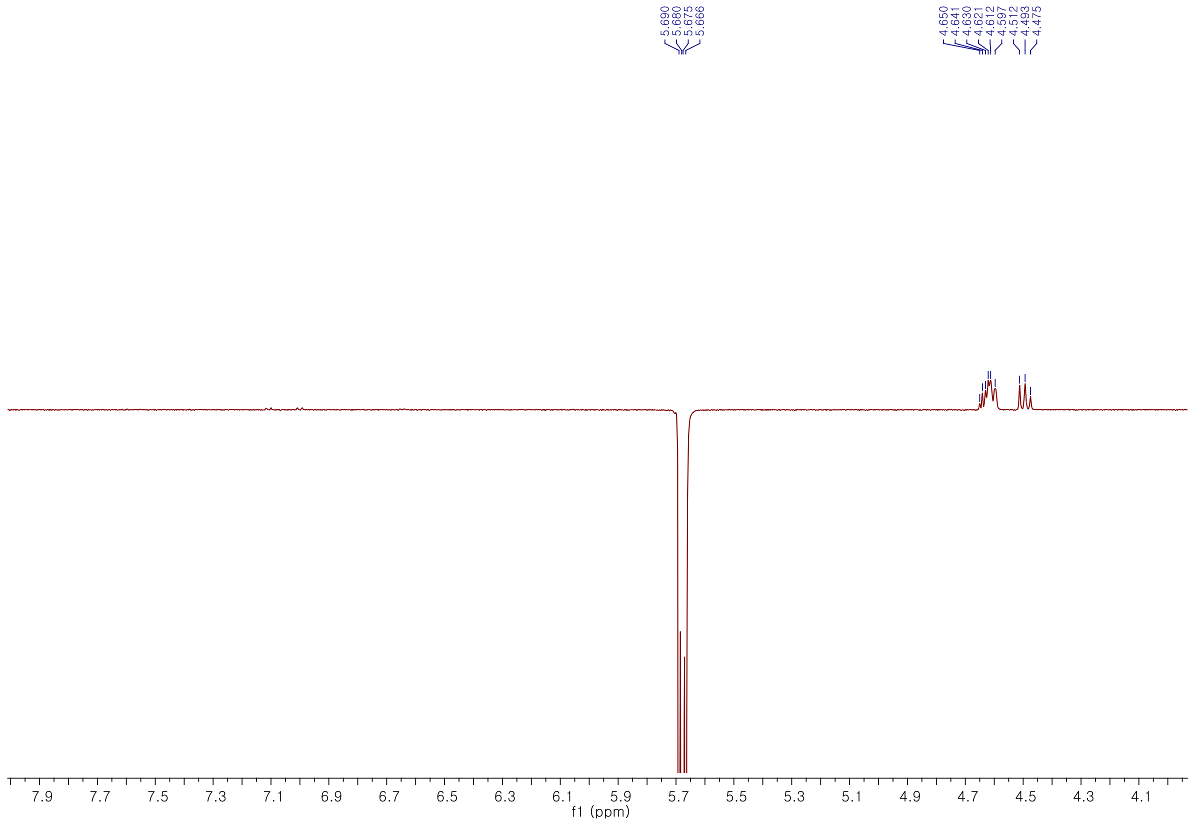

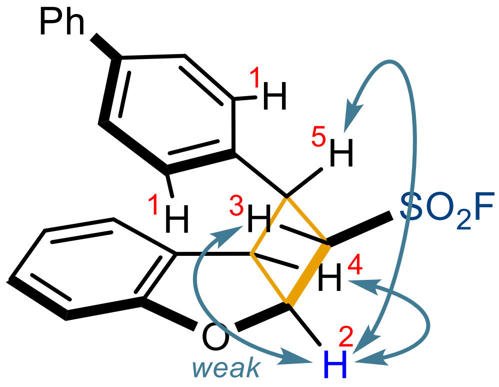

Supplementary Figure 13 1D-NOESY analysis for proton 2 of **3ma**-*exo* (500 MHz, $CDCl_3$, 25 °C).

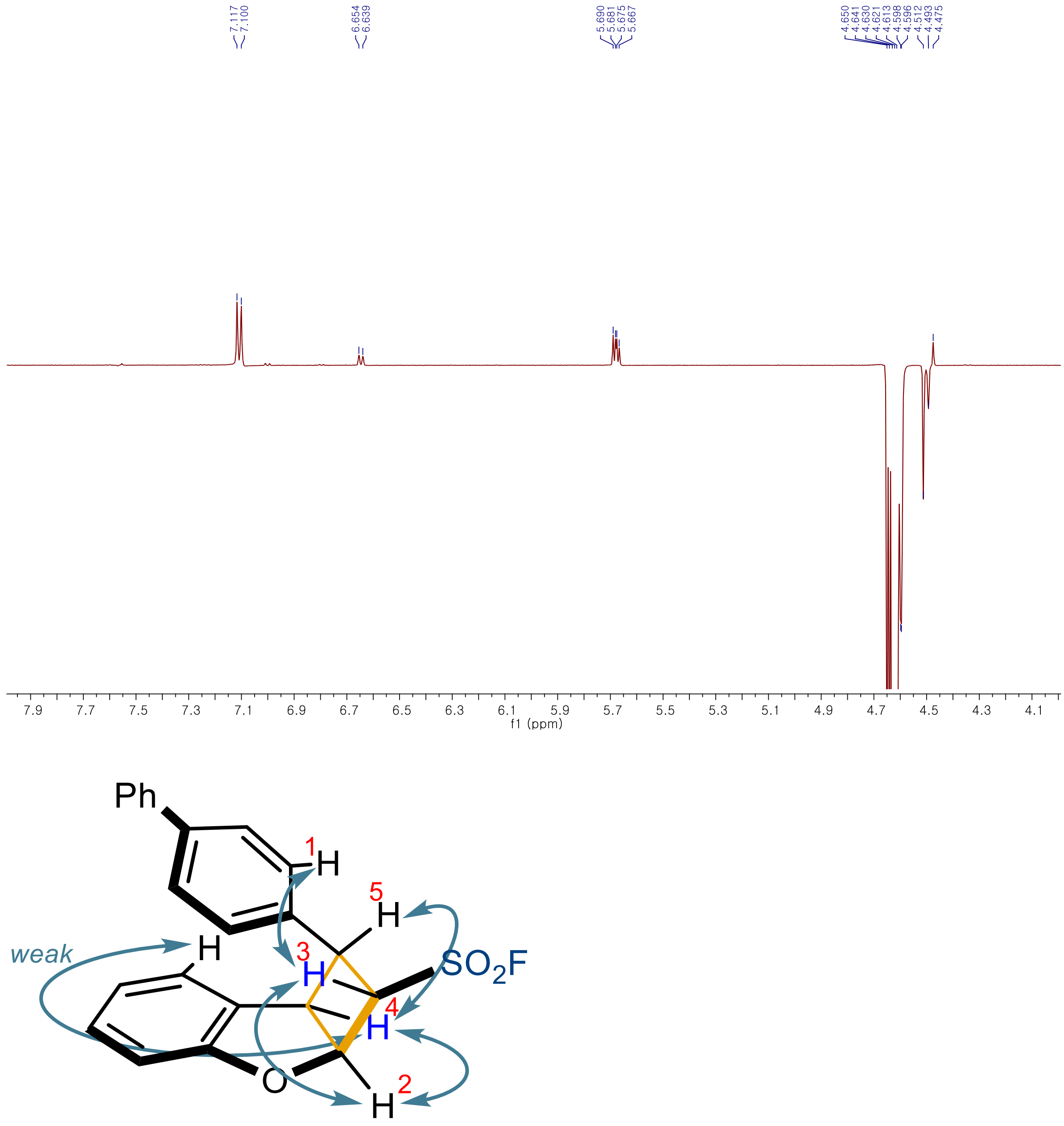

Supplementary Figure 14 1D-NOESY analysis for proton 3 and 4 of **3ma**-*exo* (500 MHz, $CDCl_3$, 25 °C).

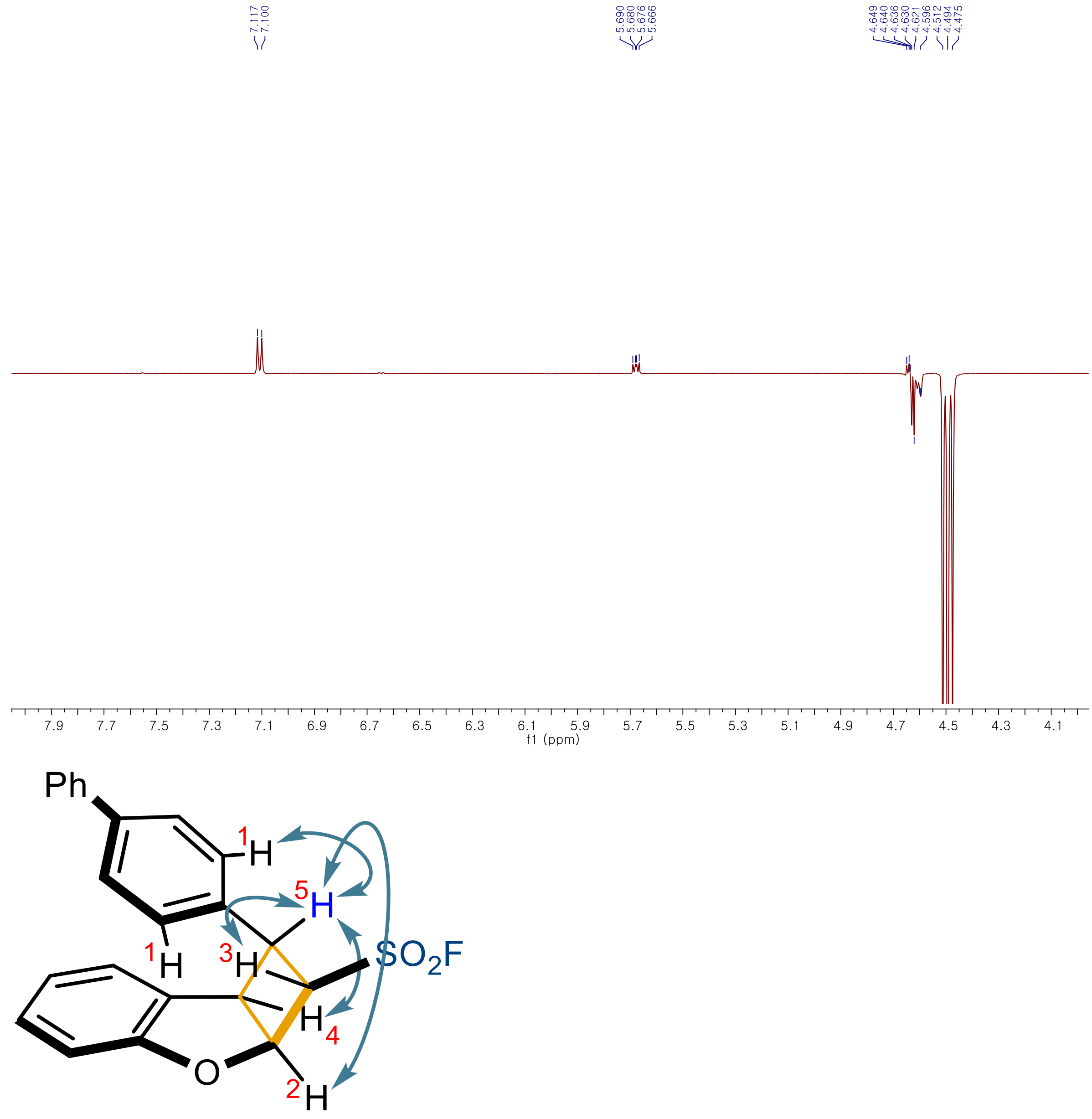

Supplementary Figure 15 1D-NOESY analysis for proton 5 of **3ma**-*exo* (500 MHz, $CDCl_3$, 25 °C).

1D-NOESY spectra of 3oa-*exo*

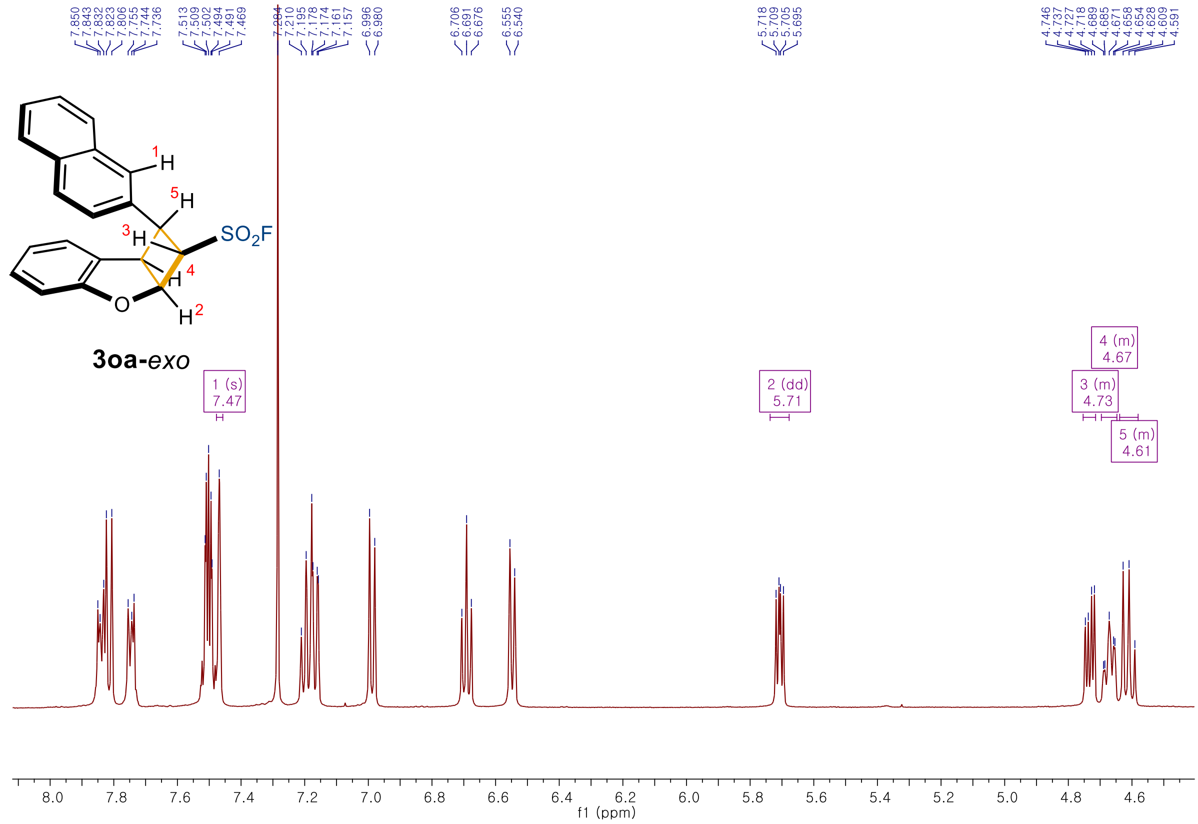

Supplementary Figure 16 ^{1}H NMR spectrum of **3oa**-*exo* (500 MHz, $CDCl_3$, 25 °C).

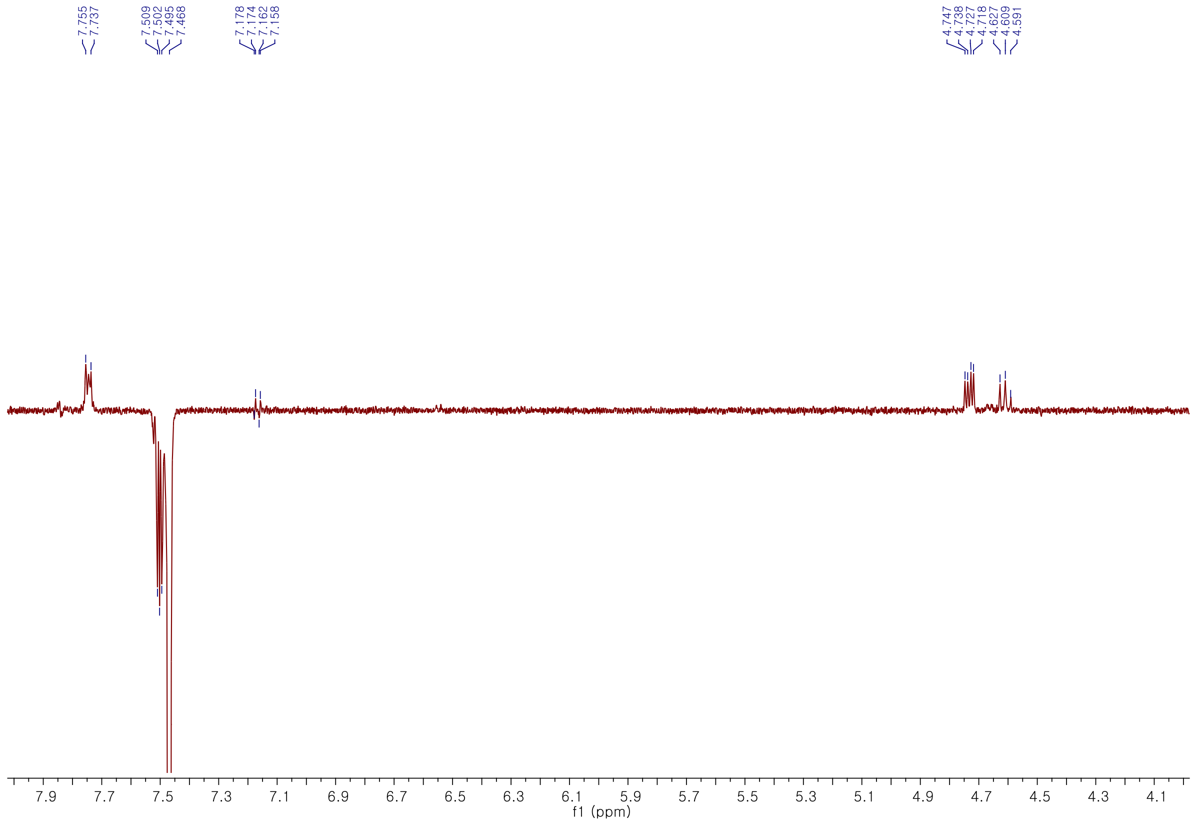

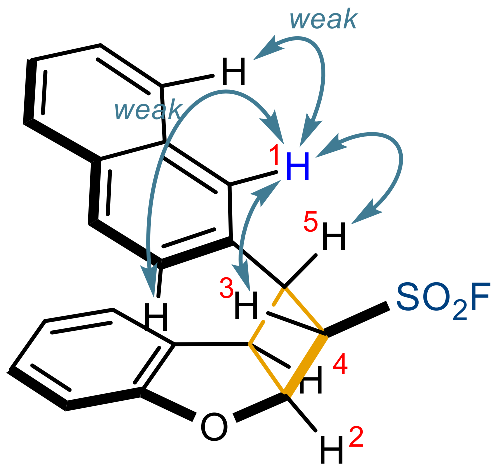

Supplementary Figure 17 1D-NOESY analysis for proton 1 of **3oa**-*exo* (500 MHz, $CDCl_3$, 25 °C).

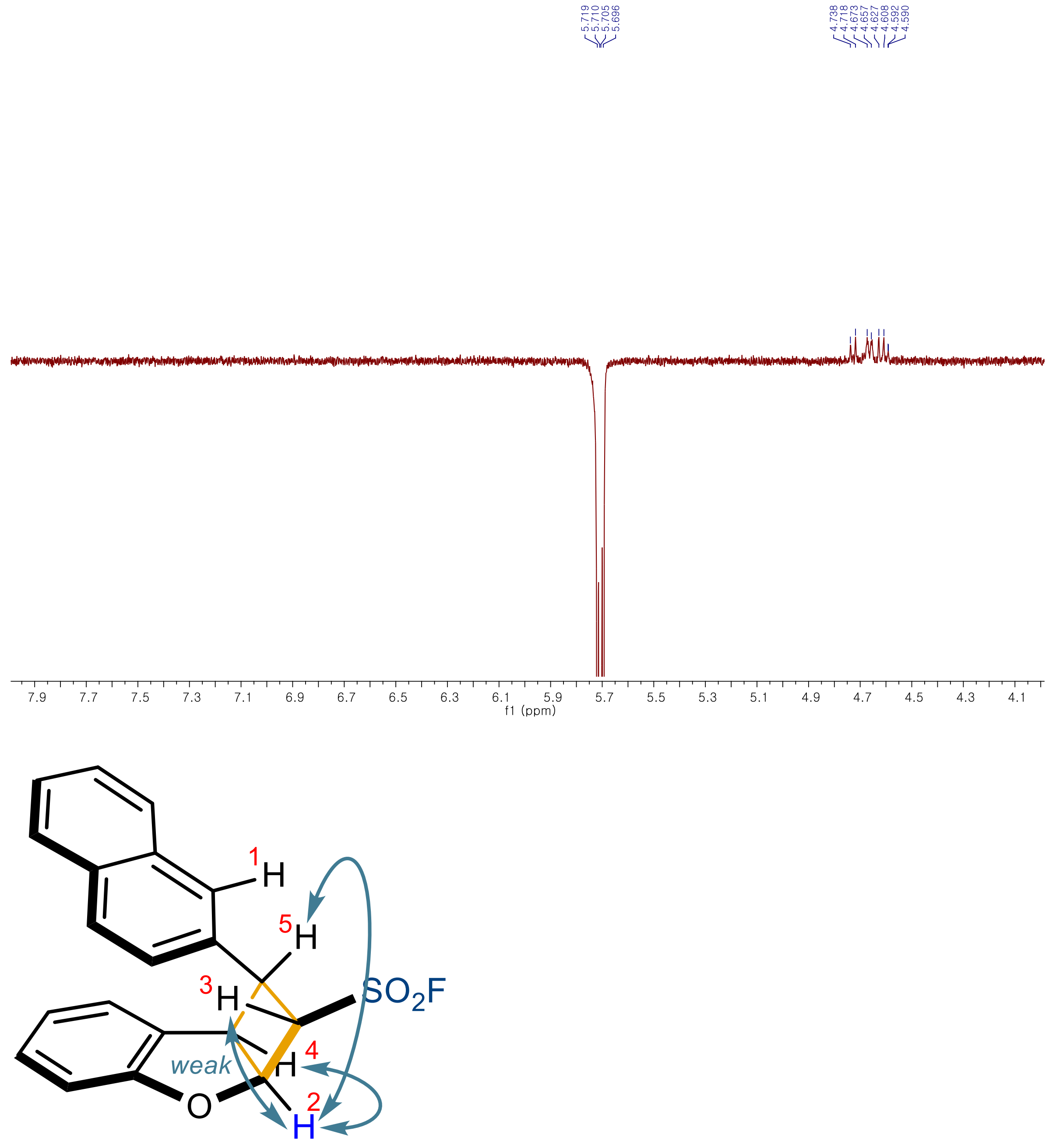

Supplementary Figure 18 1D-NOESY analysis for proton 2 of **3oa**-*exo* (500 MHz, $CDCl_3$, 25 °C).

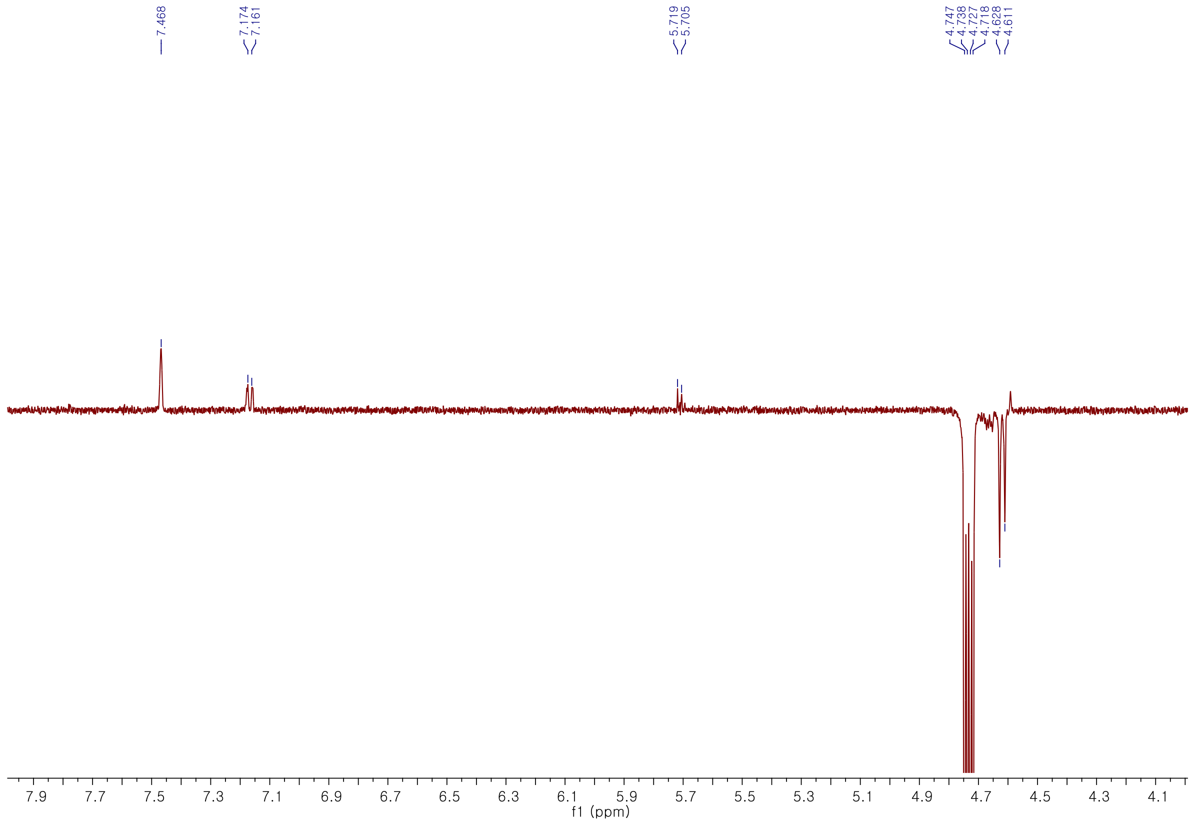

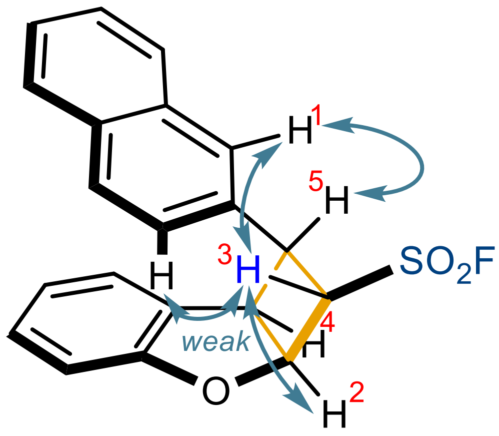

Supplementary Figure 19 1D-NOESY analysis for proton 3 of **3oa**-*exo* (500 MHz, $CDCl_3$, 25 °C).

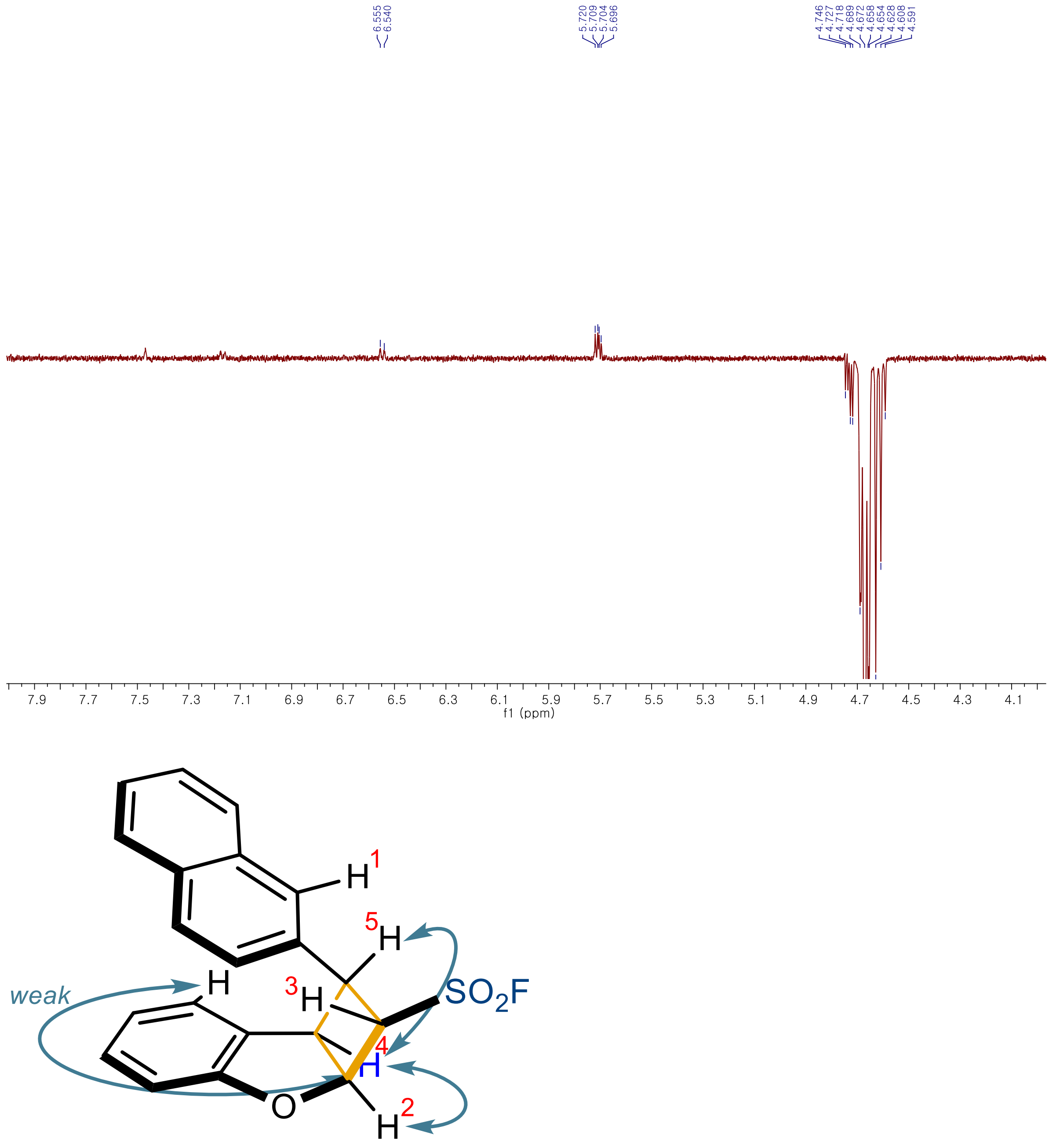

Supplementary Figure 20 1D-NOESY analysis for proton 4 of **3oa**-*exo* (500 MHz, $CDCl_3$, 25 °C).

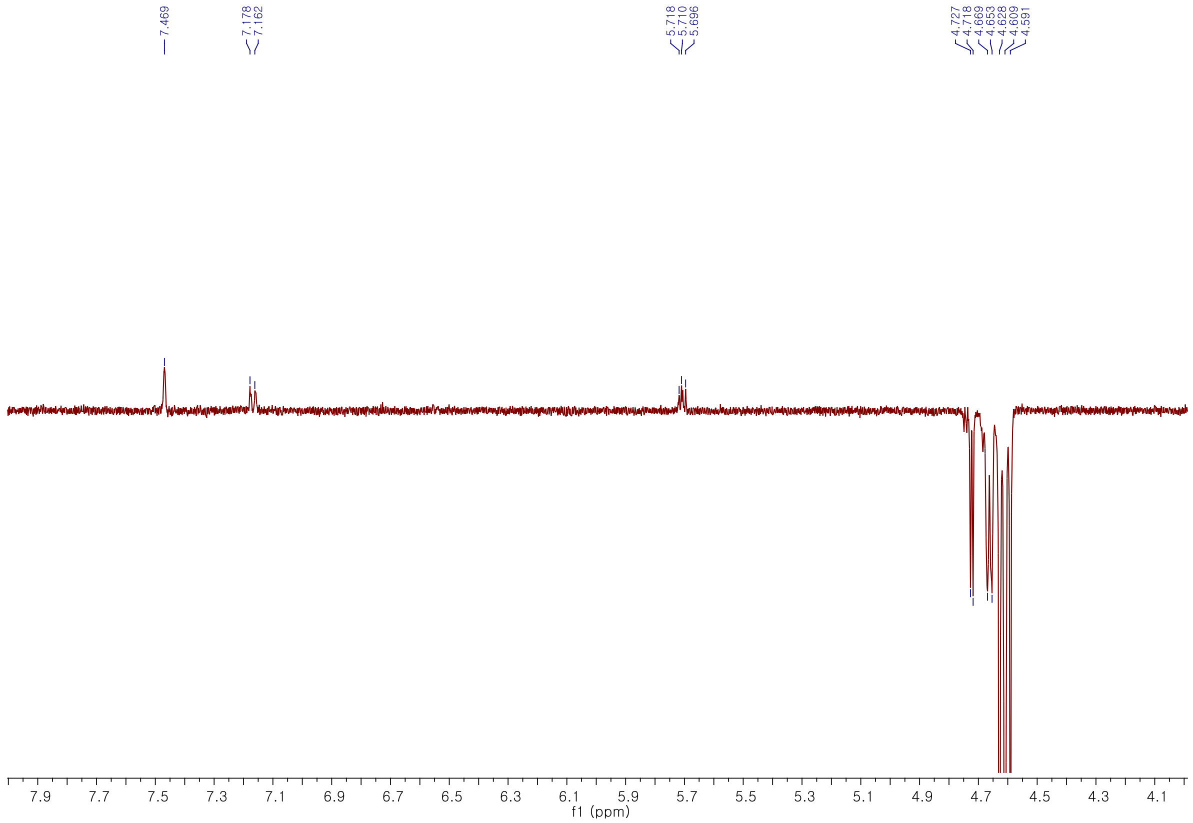

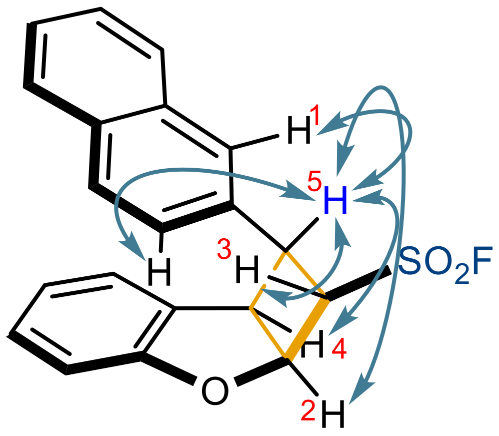

Supplementary Figure 21 1D-NOESY analysis for proton 5 of **3oa**-*exo* (500 MHz, $CDCl_3$, 25 °C).

1D-NOESY spectra of 3pa-*exo*

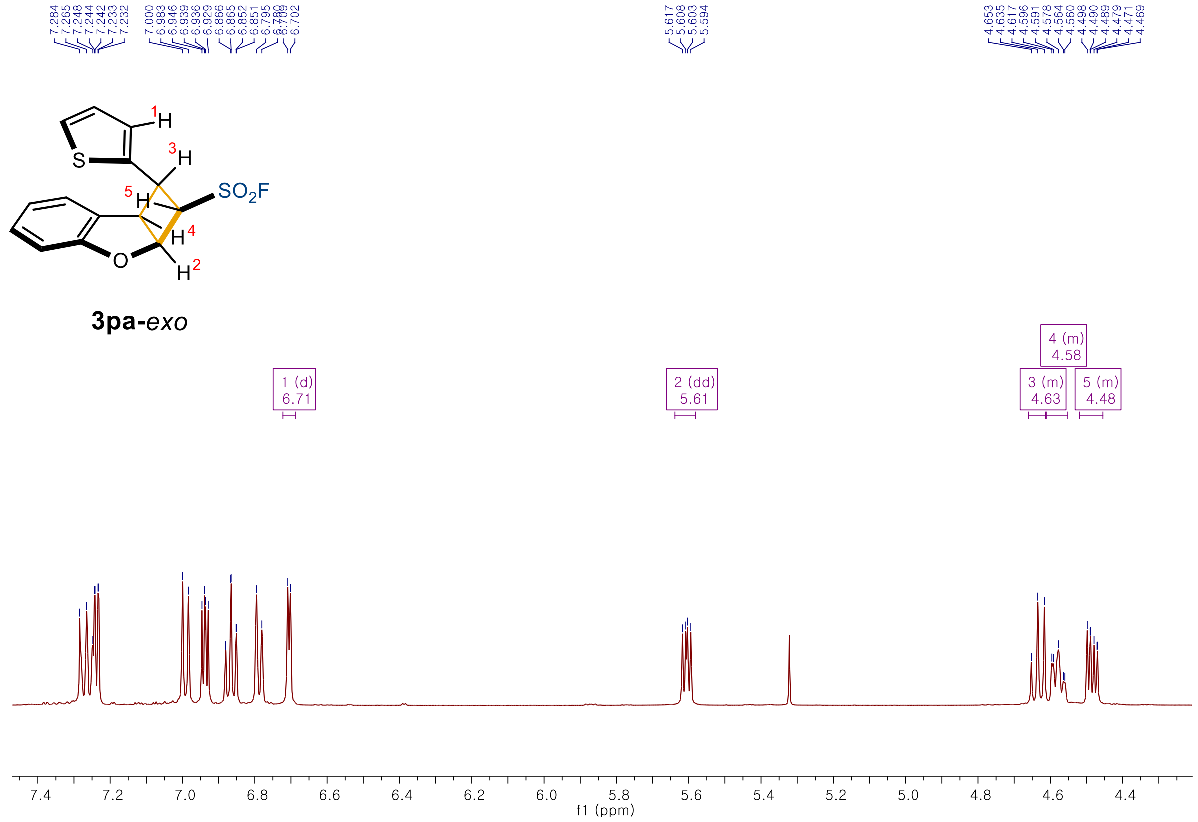

Supplementary Figure 22 ^{1}H NMR spectrum of **3pa**-*exo* (500 MHz, $CDCl_3$, 25 °C).

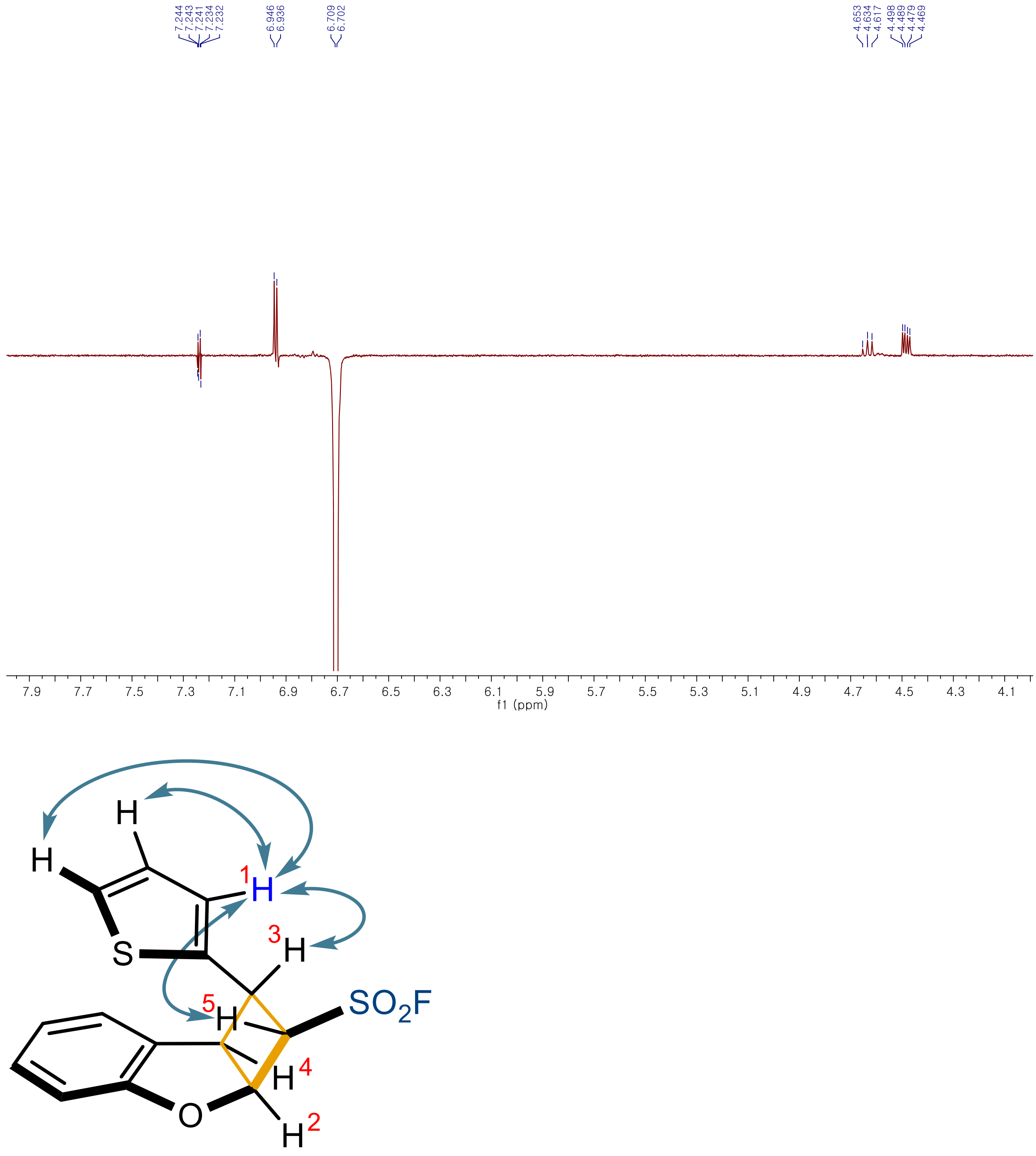

Supplementary Figure 23 1D-NOESY analysis for proton 1 of **3pa**-*exo* (500 MHz, $CDCl_3$, 25 °C).

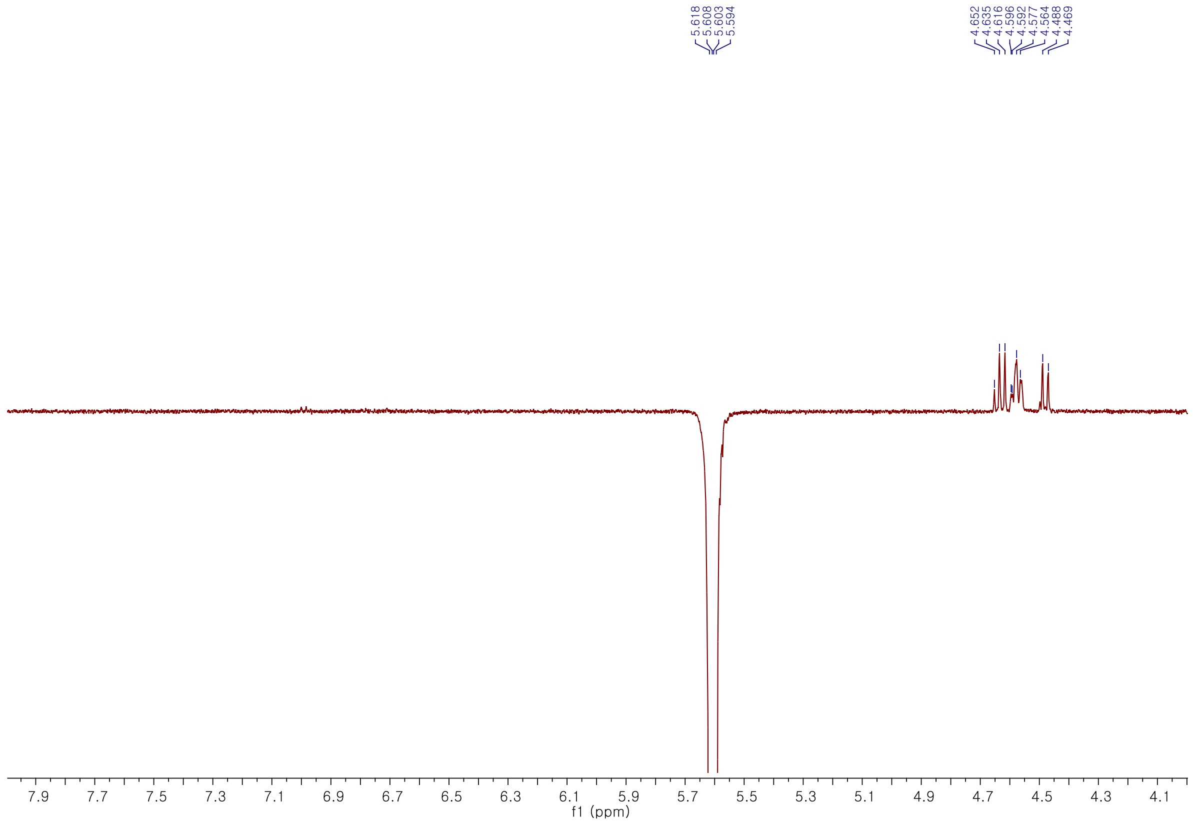

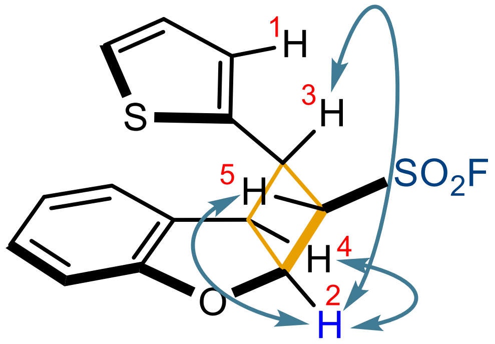

Supplementary Figure 24 1D-NOESY analysis for proton 2 of **3pa**-*exo* (500 MHz, $CDCl_3$, 25 °C).

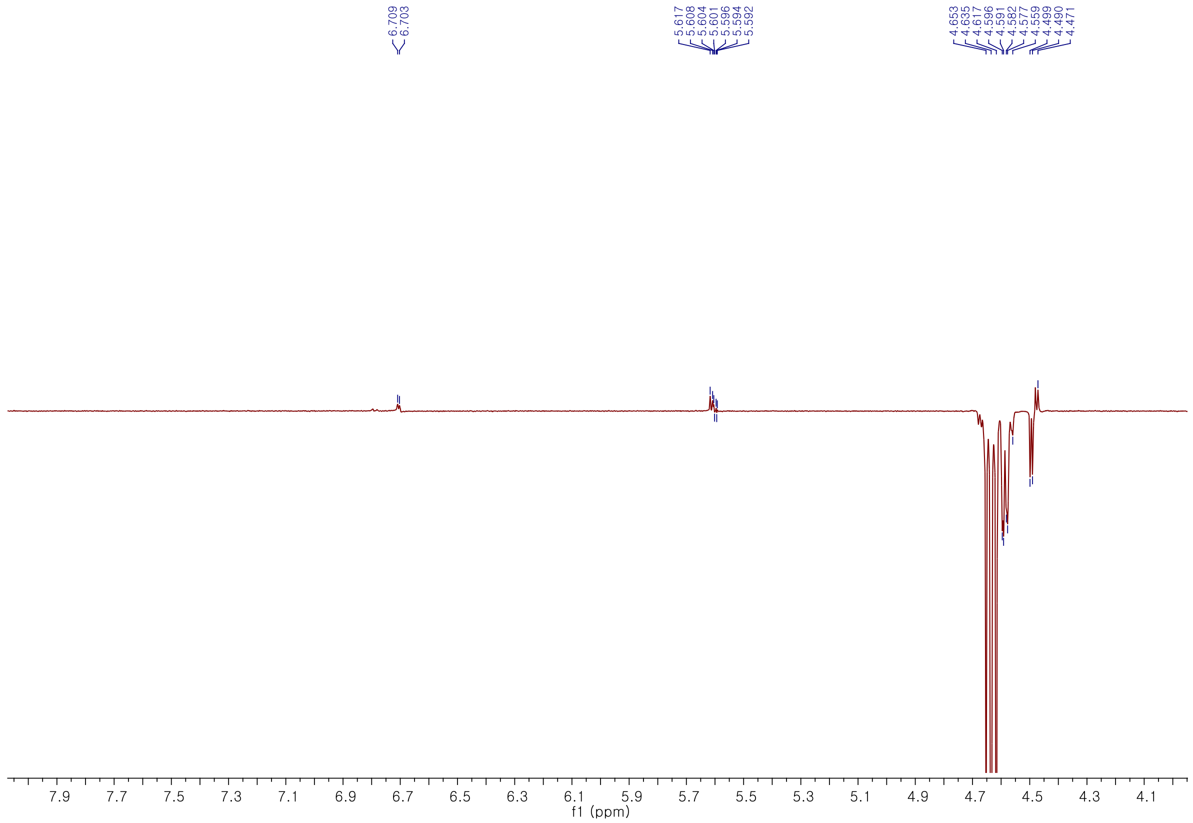

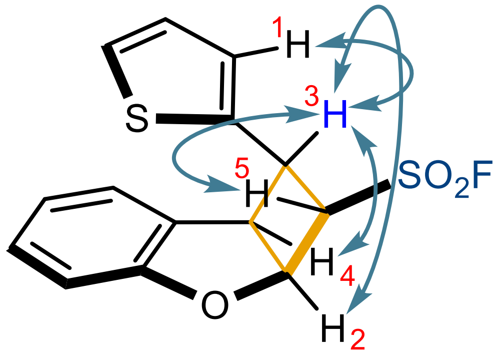

Supplementary Figure 25 1D-NOESY analysis for proton 3 of **3pa**-*exo* (500 MHz, $CDCl_3$, 25 °C).

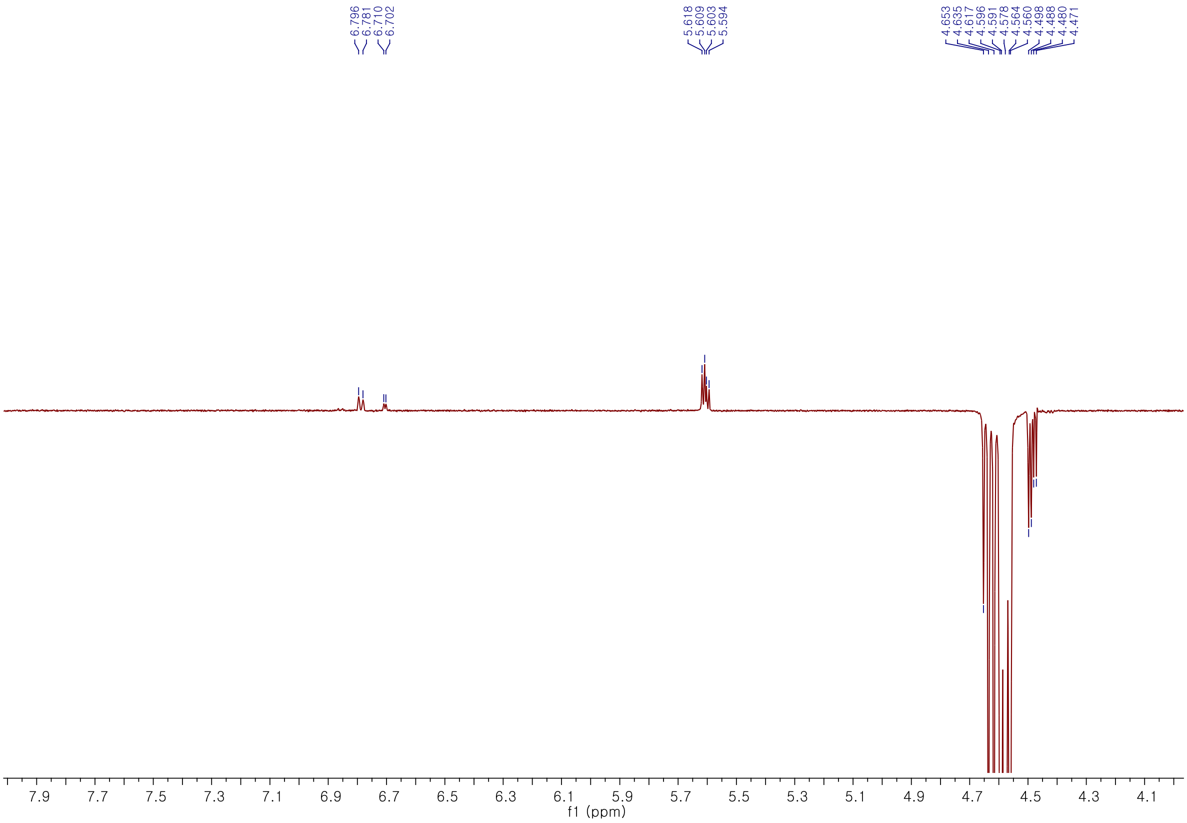

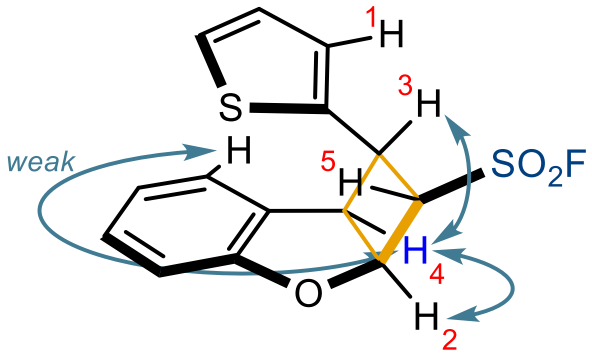

Supplementary Figure 26 1D-NOESY analysis for proton 4 of **3pa**-*exo* (500 MHz, $CDCl_3$, 25 °C).

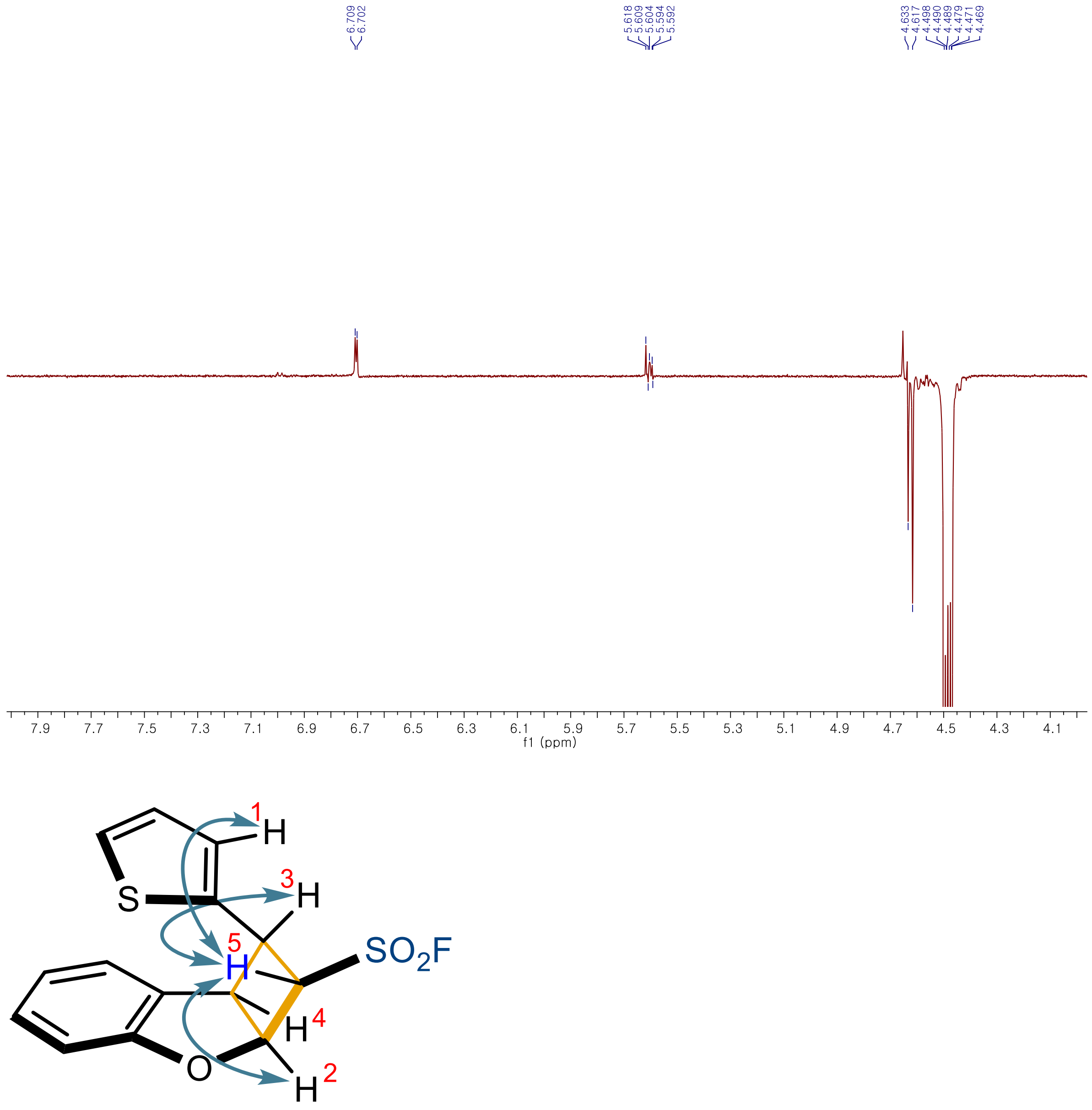

Supplementary Figure 27 1D-NOESY analysis for proton 5 of **3pa**-*exo* (500 MHz, $CDCl_3$, 25 °C).

Comment: Although the sequence of peaks on ^1H NMR changed due to thiophene group, the coupling pattern of the peaks did not change. Therefore, there was no problem with numbering. According to **Supplementary Figure 26**, when the C–H peak at δ = 4.58 ppm (multiplet) was selectively inverted, 4-position proton of benzofuran moiety was affected. This result suggests that C–H peak at δ = 4.58 ppm (multiplet) is proton 4. Based on the 1D-NOESY NMR analysis, the proton 5 does not affect proton 4, and the proton 3 does not interact with the 4-position of benzofuran moiety. In conclusion, proton 3 is in the opposite direction to the benzofuran moiety, and protons 4 is oriented in the opposite direction with proton 5. Therefore, obtained product was determined to be an *exo*-configuration as a major form (like other products) although it has the thiophene heterocycle.

1D-NOESY spectra of 3pa-*endo*

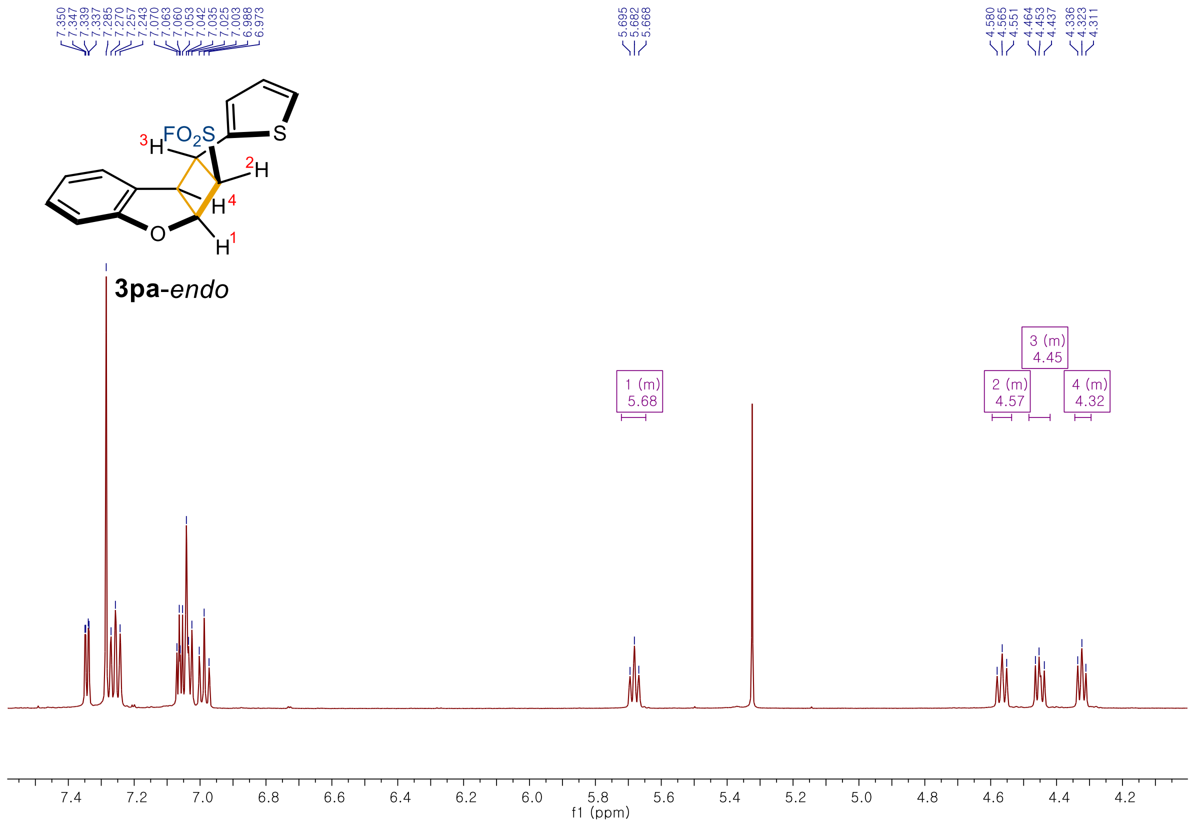

Supplementary Figure 28 ^{1}H NMR spectrum of **3pa**-*endo* (500 MHz, $CDCl_3$, 25 °C).

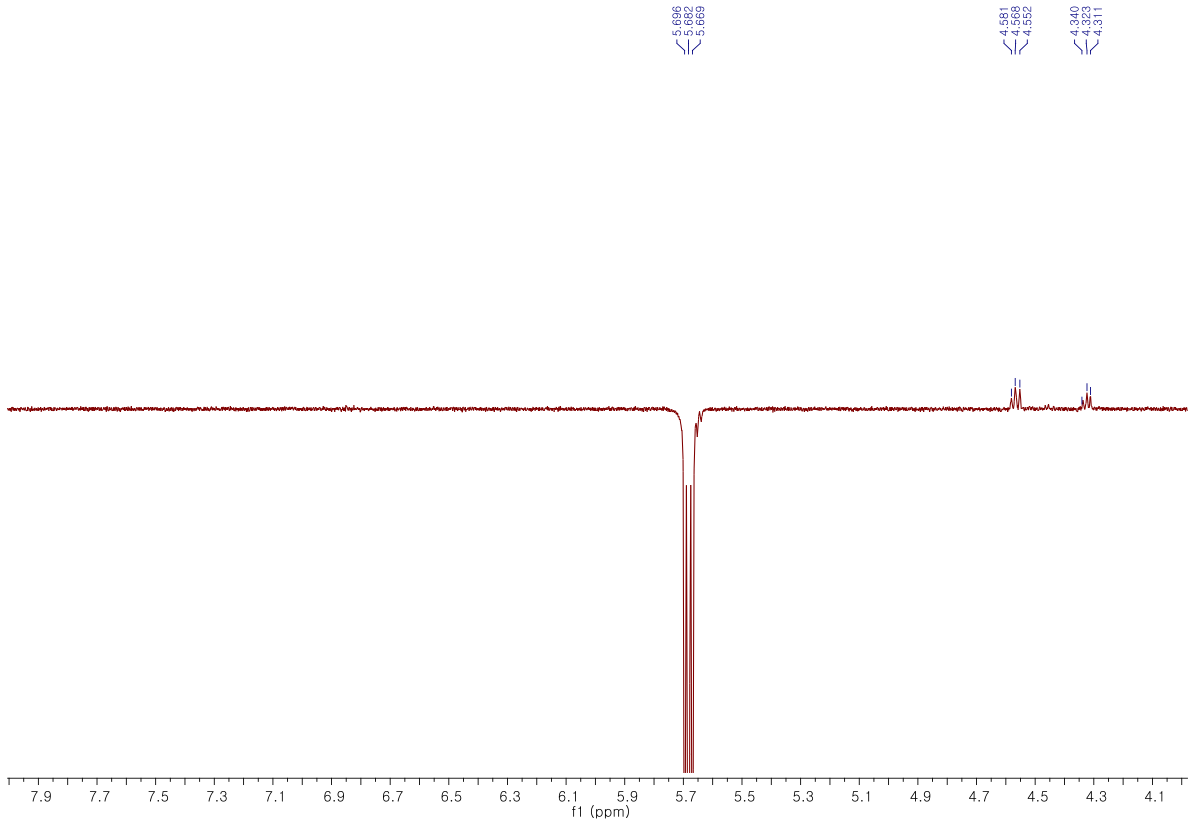

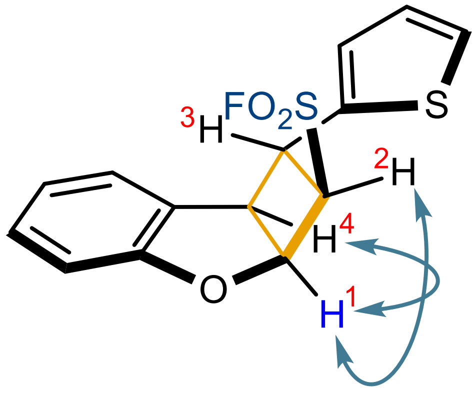

Supplementary Figure 29 1D-NOESY analysis for proton 1 of **3pa**-*endo* (500 MHz, $CDCl_3$, 25 °C).

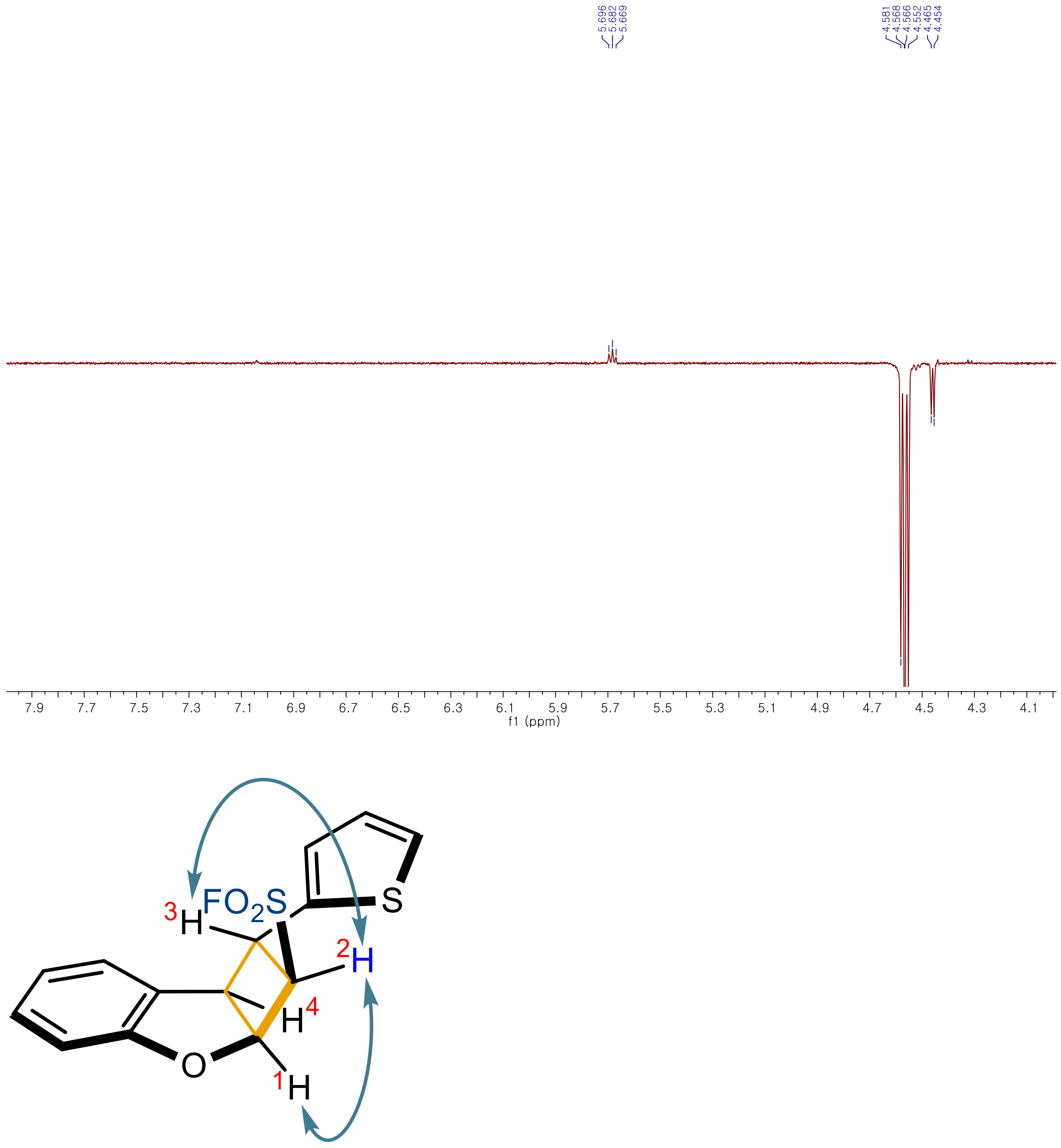

Supplementary Figure 30 1D-NOESY analysis for proton 2 of **3pa**-*endo* (500 MHz, $CDCl_3$, 25 °C).

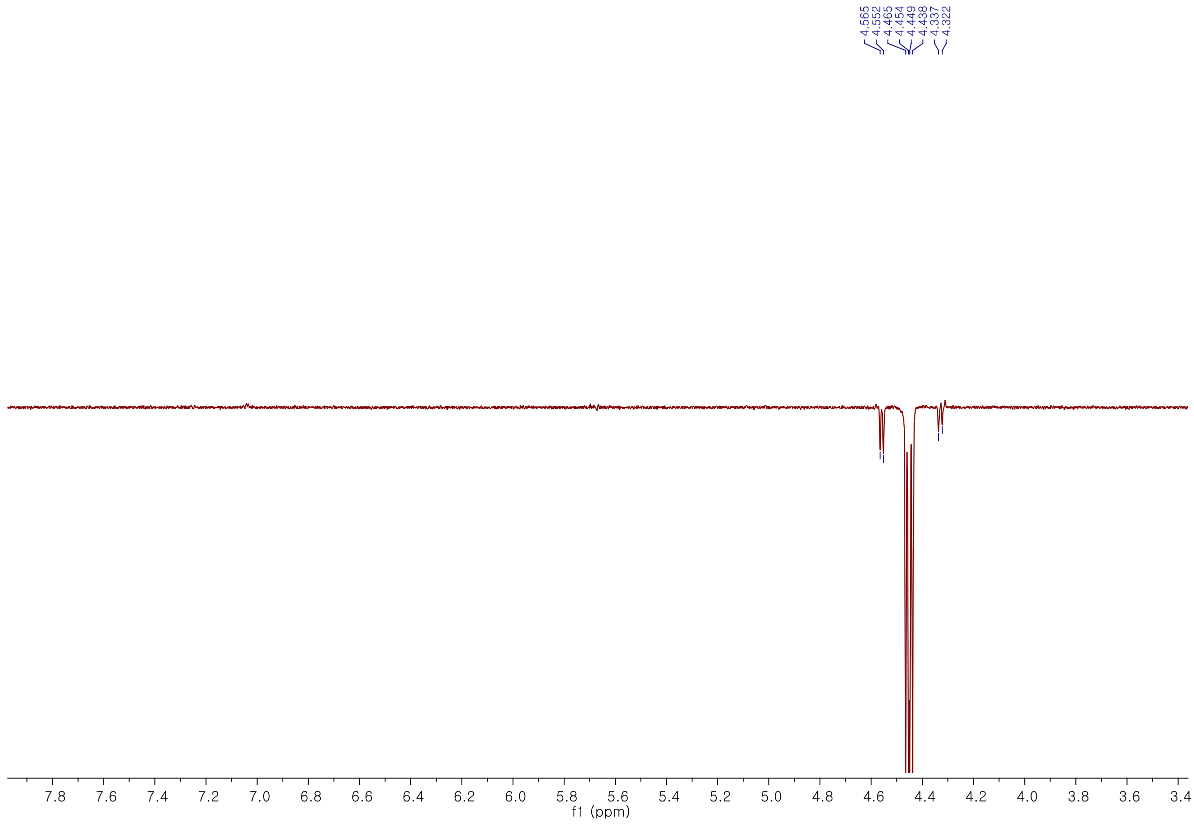

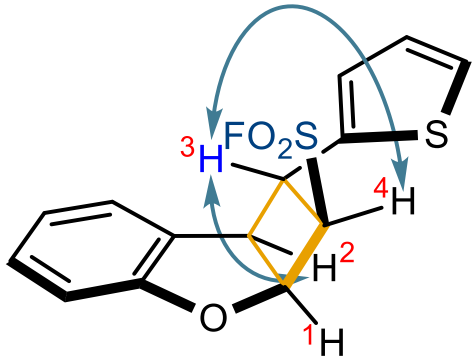

Supplementary Figure 31 1D-NOESY analysis for proton 3of **3pa**-*endo* (500 MHz, $CDCl_3$, 25 °C).

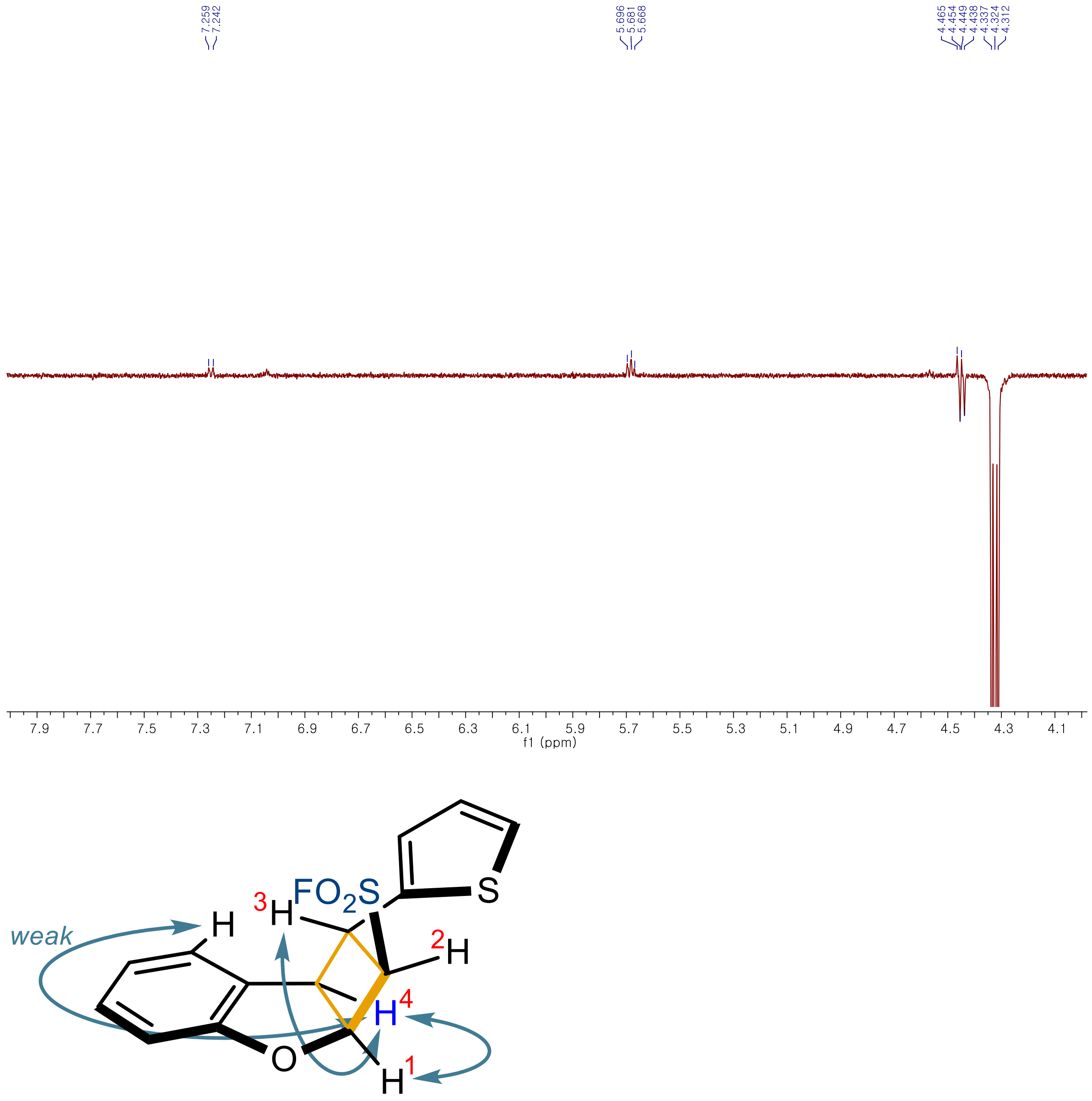

Supplementary Figure 32 1D-NOESY analysis for proton 4 of **3pa**-*endo* (500 MHz, $CDCl_3$, 25 °C).

1D-NOESY spectra of 3qa-*exo*

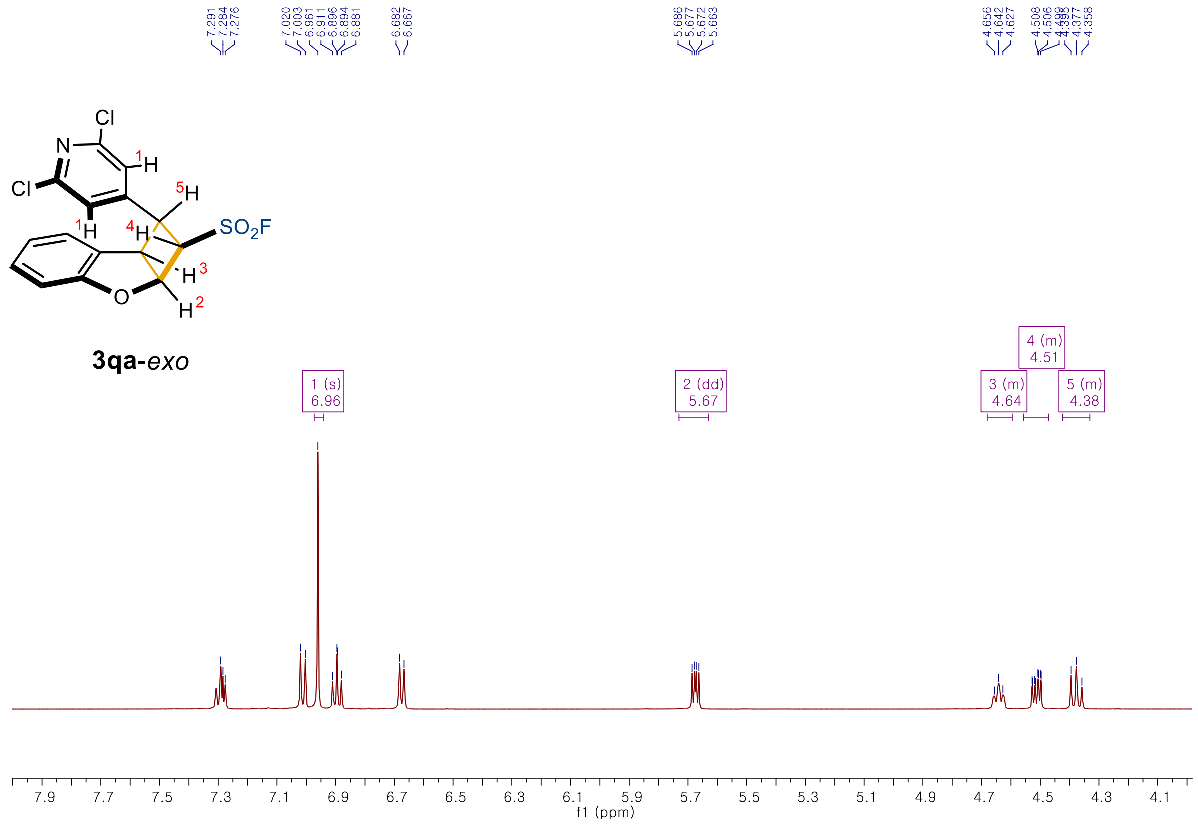

Supplementary Figure 33 ^{1}H NMR spectrum of **3qa**-*exo* (500 MHz, $CDCl_3$, 25 °C).

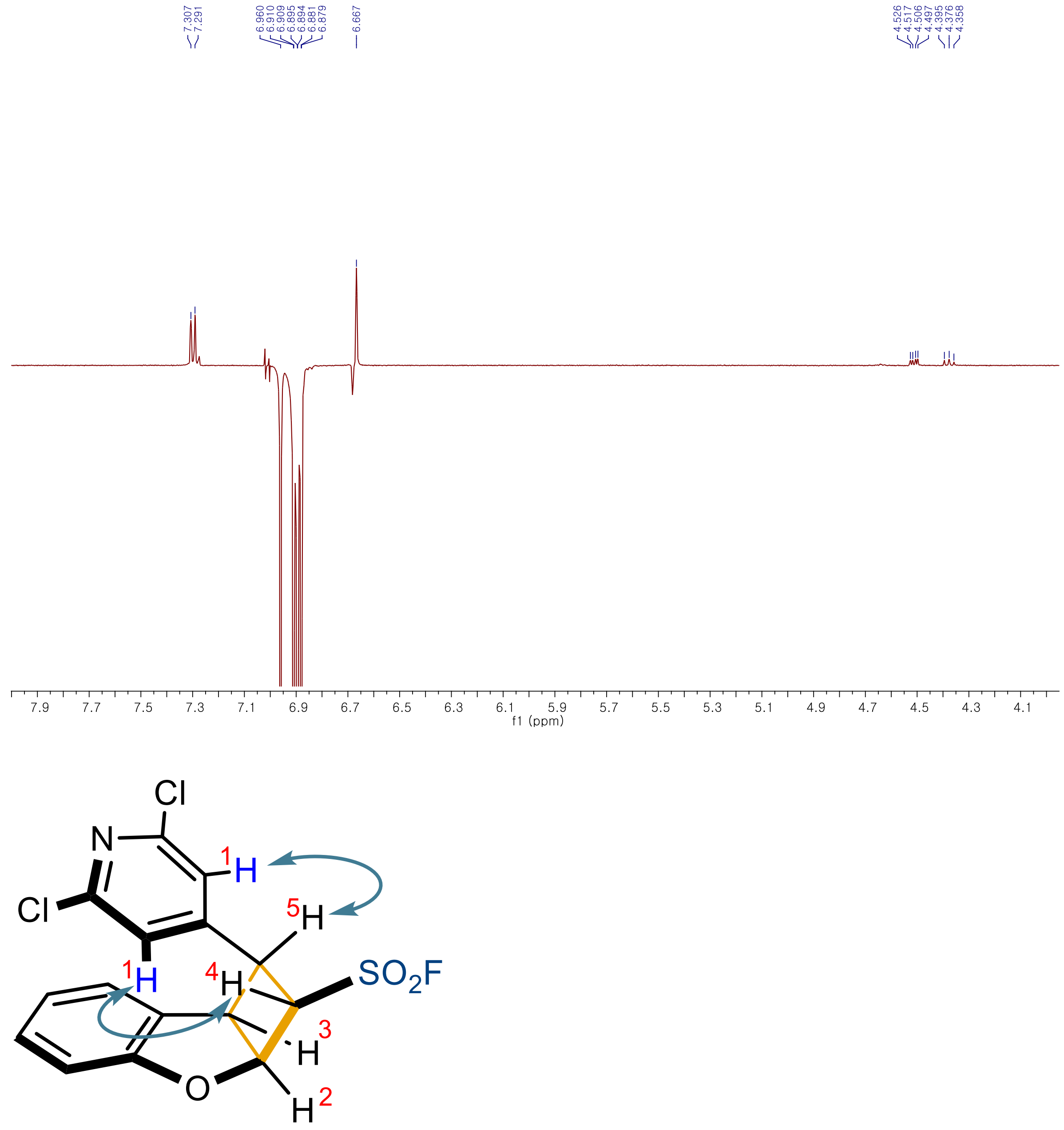

Supplementary Figure 34 1D-NOESY analysis for proton 1 of **3qa**-*exo* (500 MHz, $CDCl_3$, 25 °C).

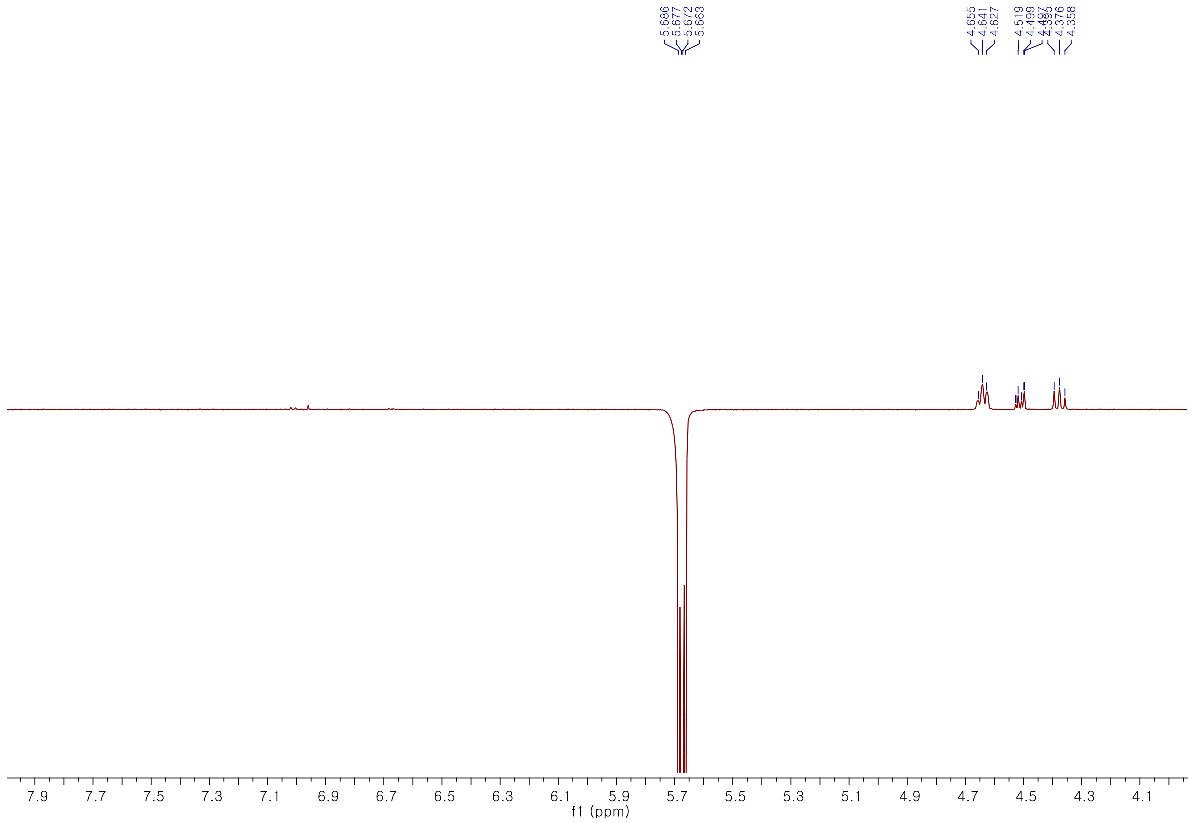

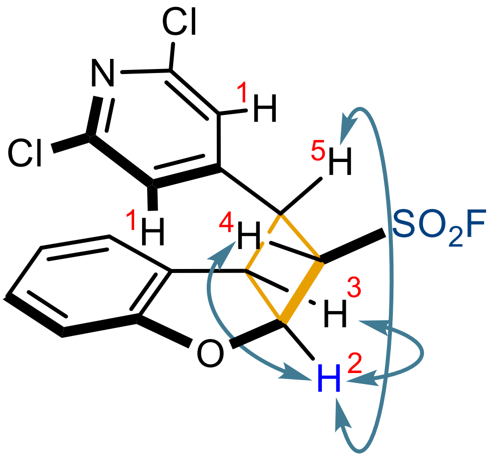

Supplementary Figure 35 1D-NOESY analysis for proton 2 of **3qa**-*exo* (500 MHz, $CDCl_3$, 25 °C).

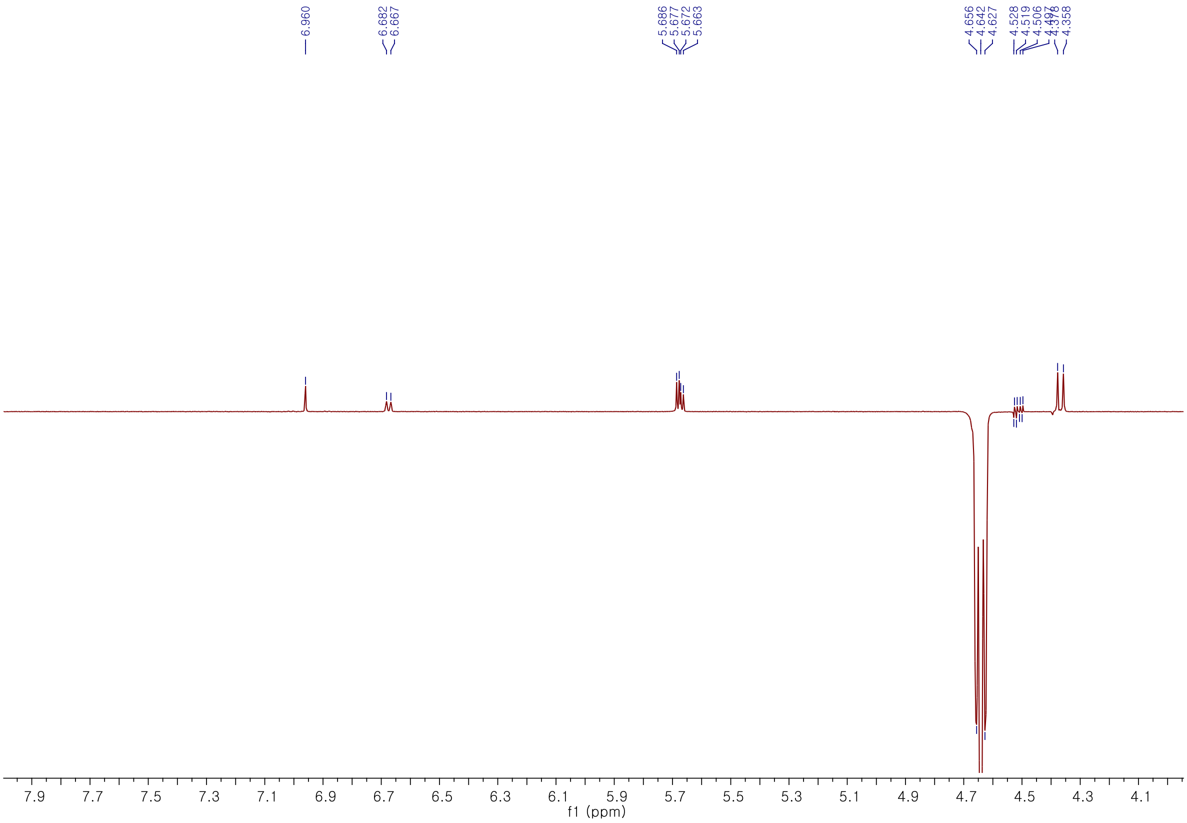

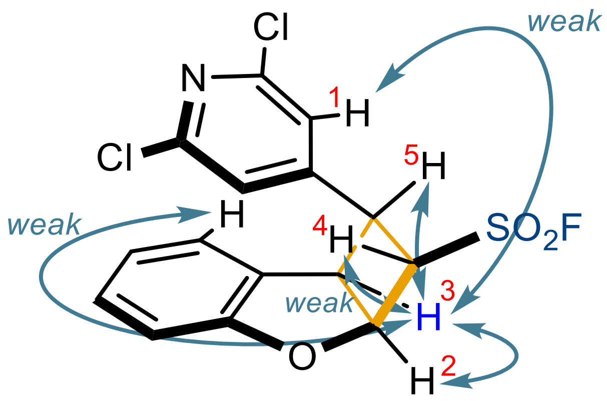

Supplementary Figure 36 1D-NOESY analysis for proton 3 of **3qa**-*exo* (500 MHz, $CDCl_3$, 25 °C).

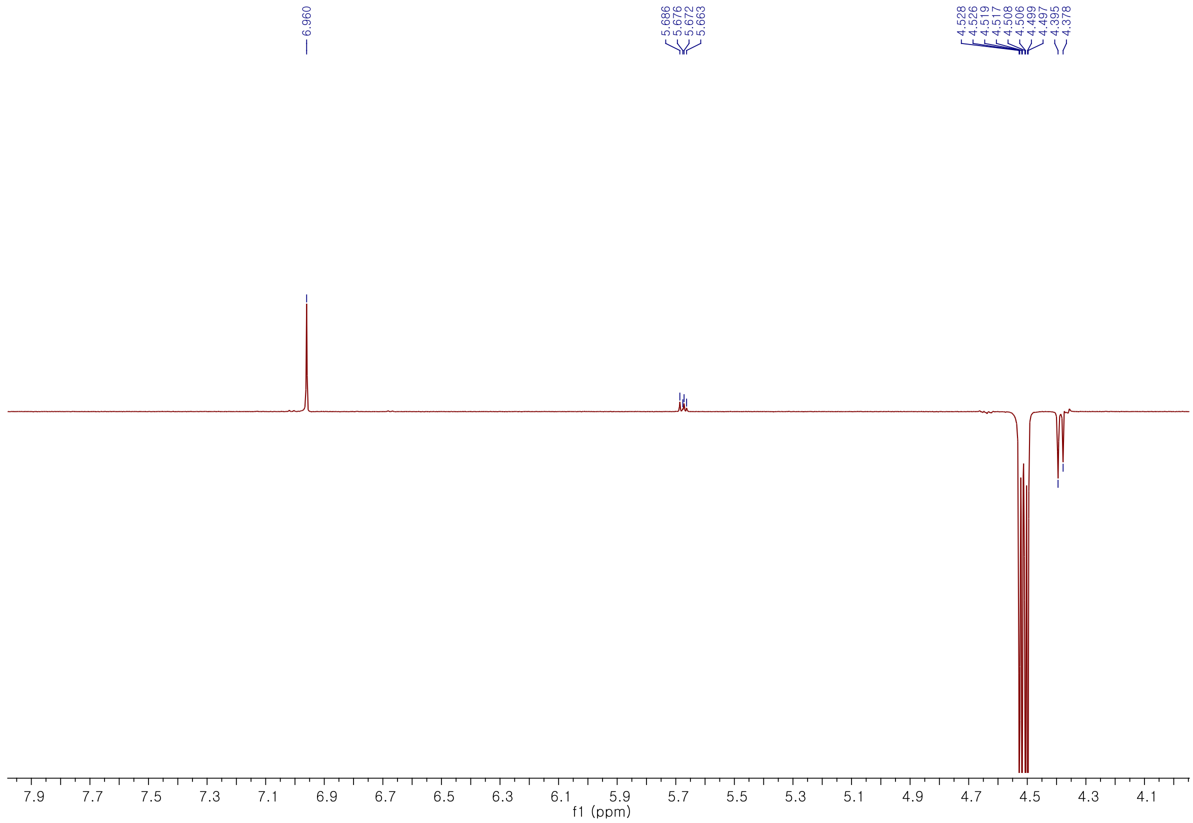

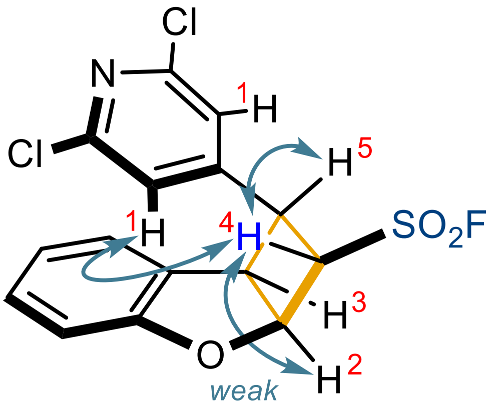

Supplementary Figure 37 1D-NOESY analysis for proton 4 of **3qa**-*exo* (500 MHz, $CDCl_3$, 25 °C).

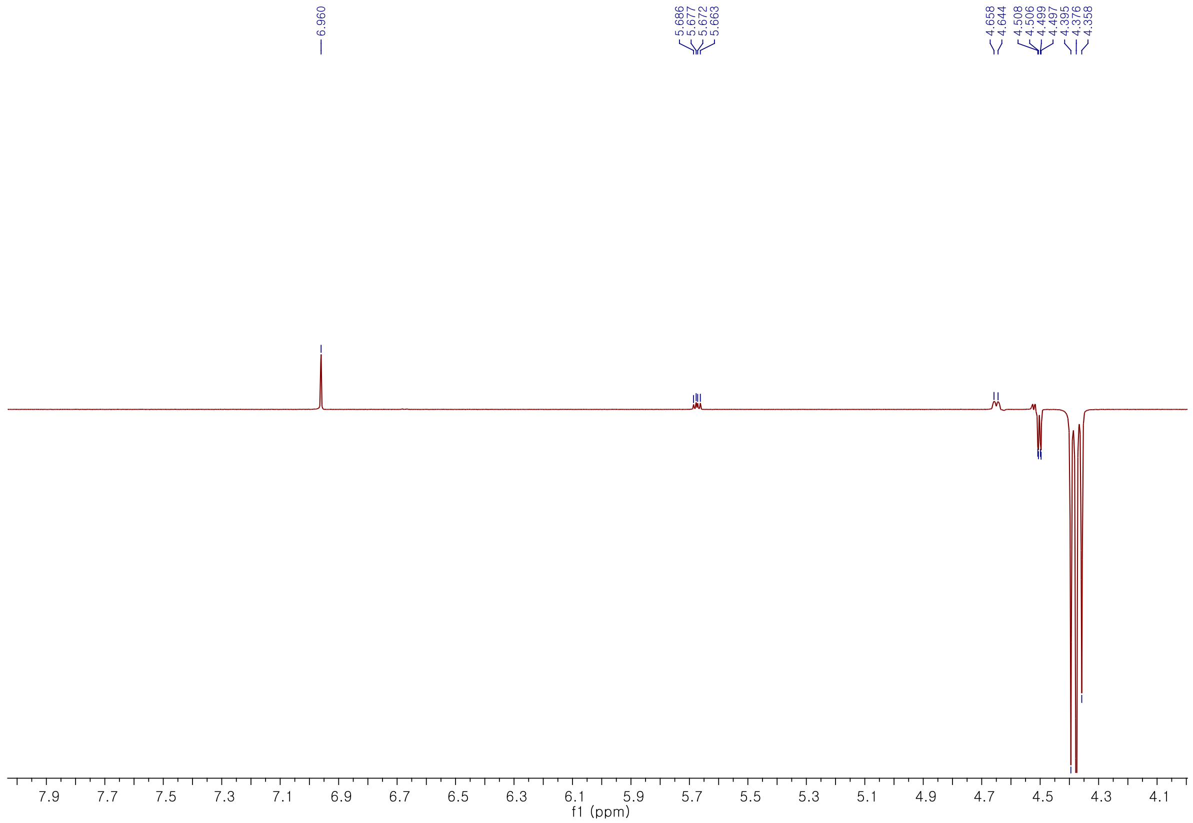

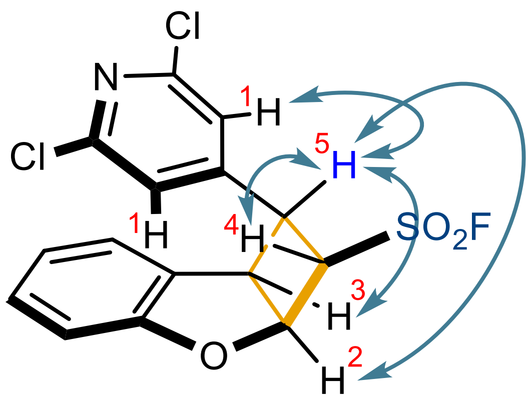

Supplementary Figure 38 1D-NOESY analysis for proton 5 of **3qa**-*exo* (500 MHz, $CDCl_3$, 25 °C).

Comment: Similar to the case of **3pa**-*exo*, the sequence of peaks on ^1H NMR changed but the coupling pattern of the peaks did not change. Therefore, there was no problem with numbering. The ^1H NMR peak of proton 4 (δ = 4.51 ppm), which is close to the sulfonyl fluoride group due to the 2,6-dichloropyridyl moiety, more upfield shifted than proton 3. When this proton 4 is selectively inverted, proton 3 was not affected. And when proton 1 was selectively inverted, proton 3 was also unaffected. As a result, the product is determined to be an *exo*-configuration in which proton 4 is in the same orientation as the oxygen (ether moiety). Proton 5 is in the opposite orientation to the oxygen of benzofuran moiety.

1D-NOESY spectra of 3qa-*endo*

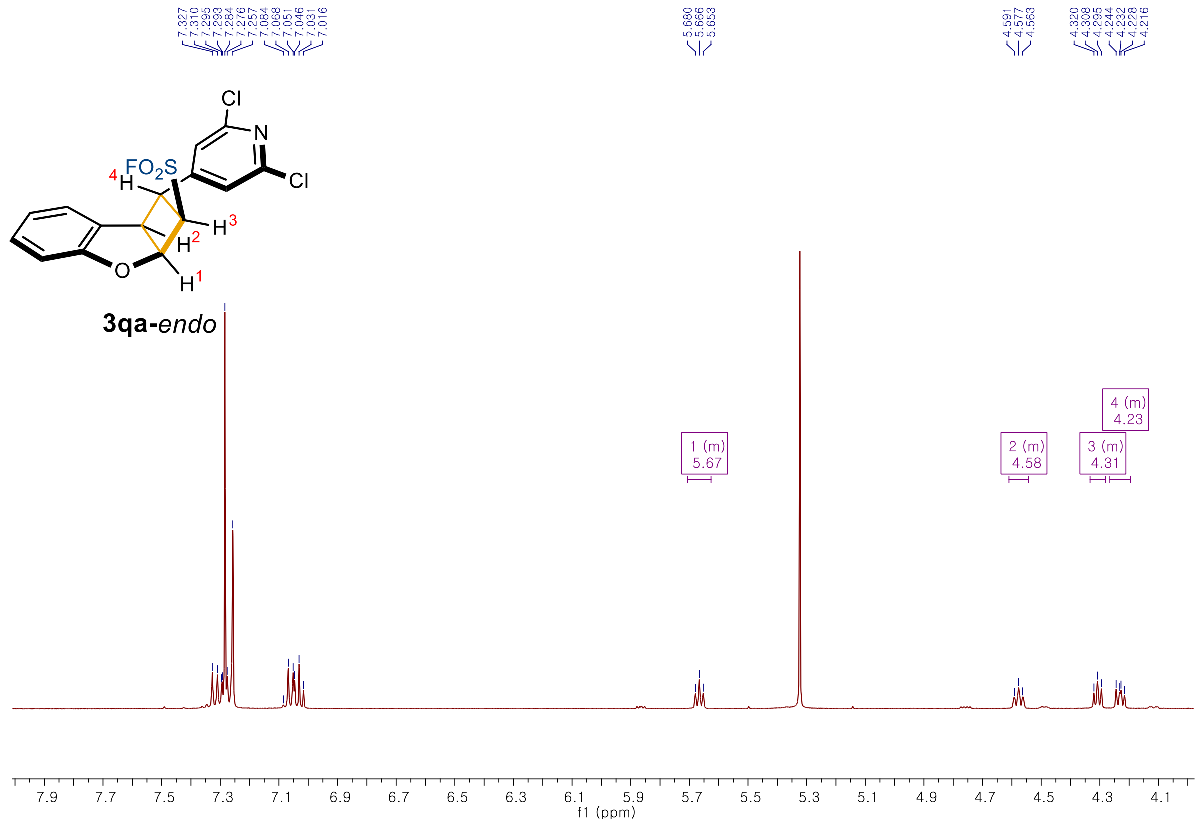

Supplementary Figure 39 ^{1}H NMR spectrum of **3qa**-*endo* (500 MHz, $CDCl_3$, 25 °C).

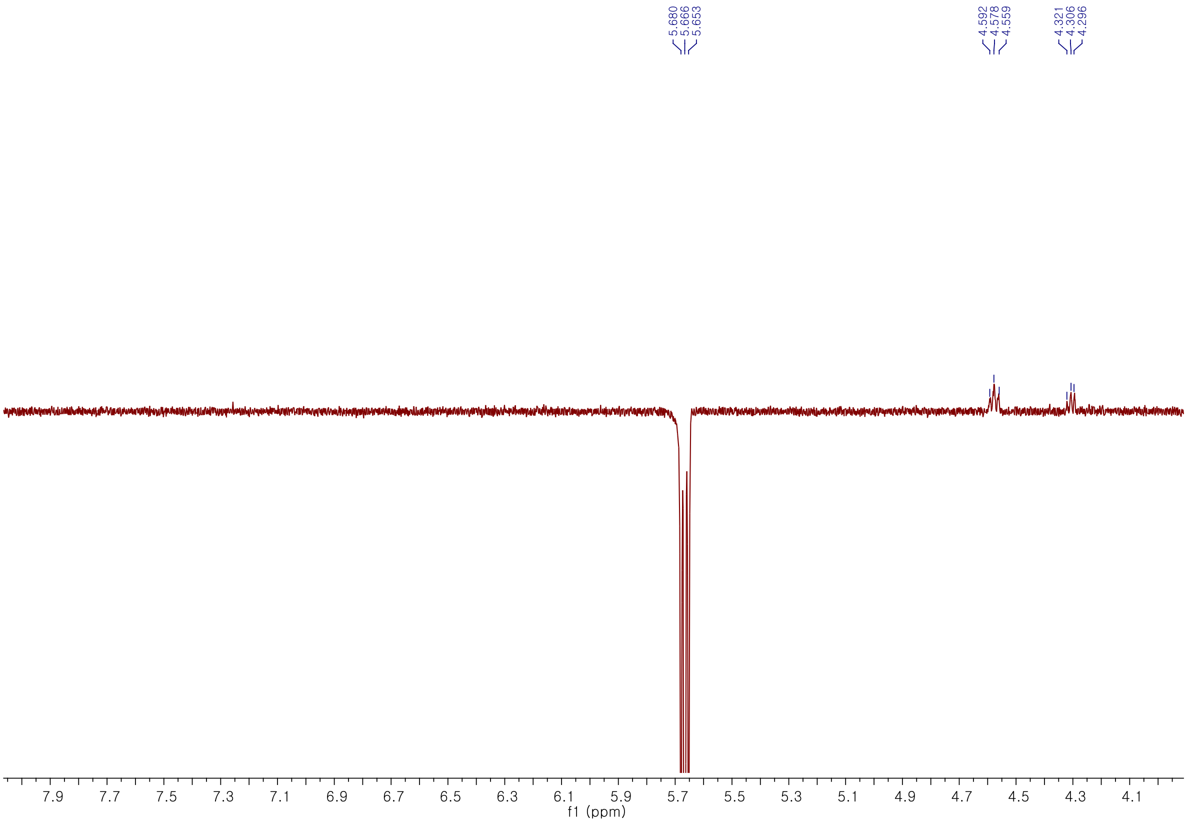

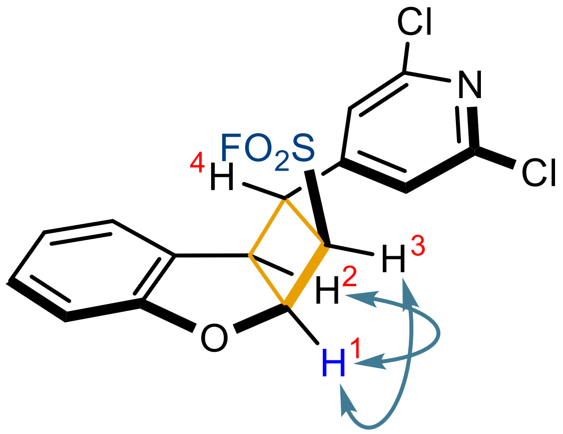

Supplementary Figure 40 1D-NOESY analysis for proton 1 of **3qa**-*endo* (500 MHz, $CDCl_3$, 25 °C).

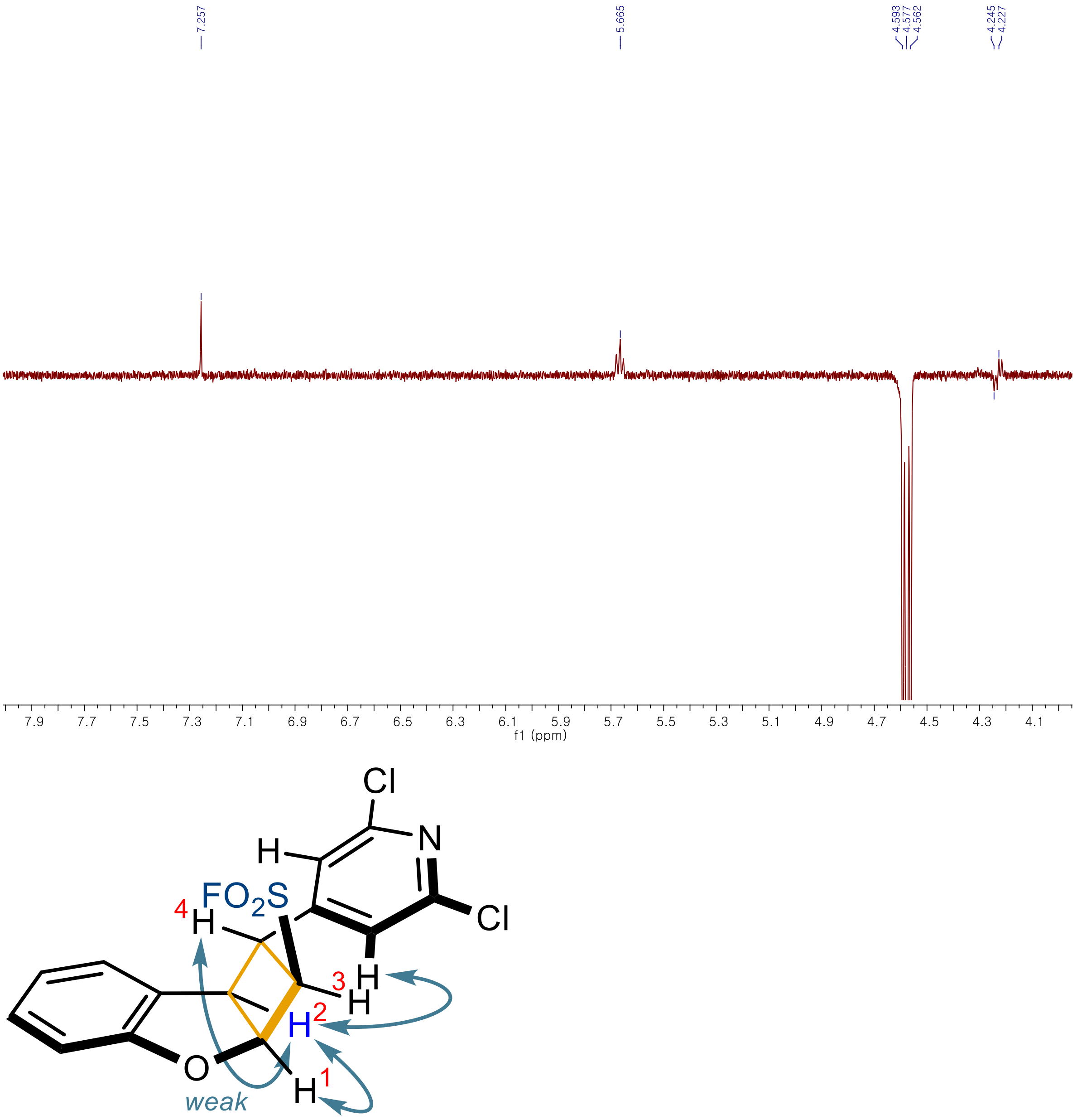

Supplementary Figure 41 1D-NOESY analysis for proton 2 of **3qa**-*endo* (500 MHz, $CDCl_3$, 25 °C).

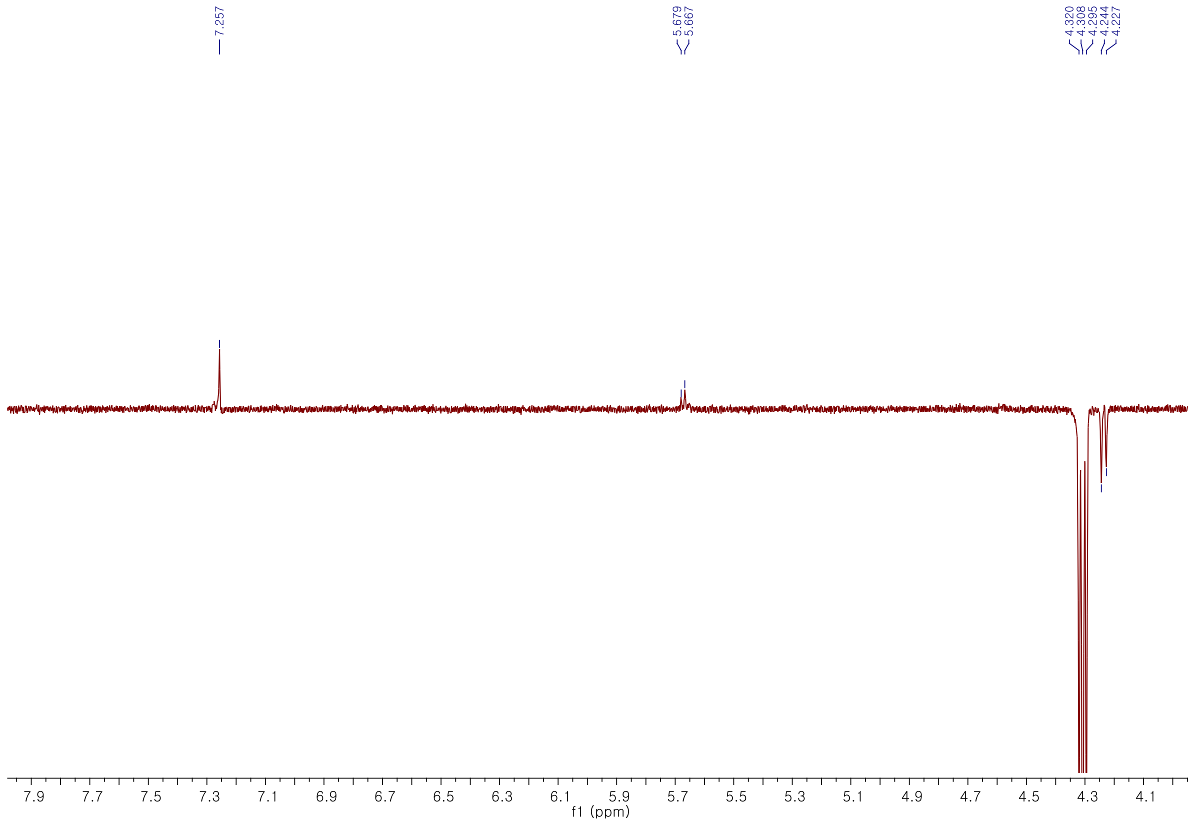

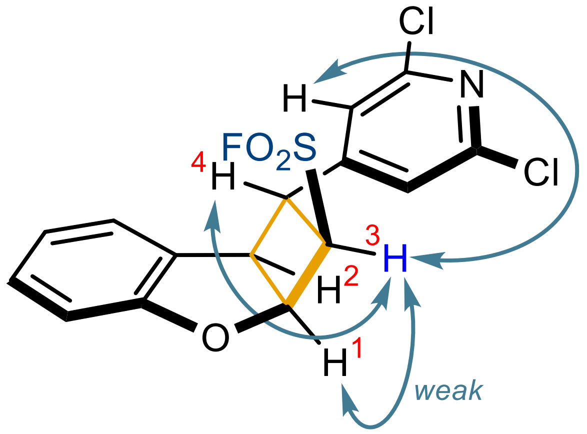

Supplementary Figure 42 1D-NOESY analysis for proton 3 of **3qa**-*endo* (500 MHz, $CDCl_3$, 25 °C).

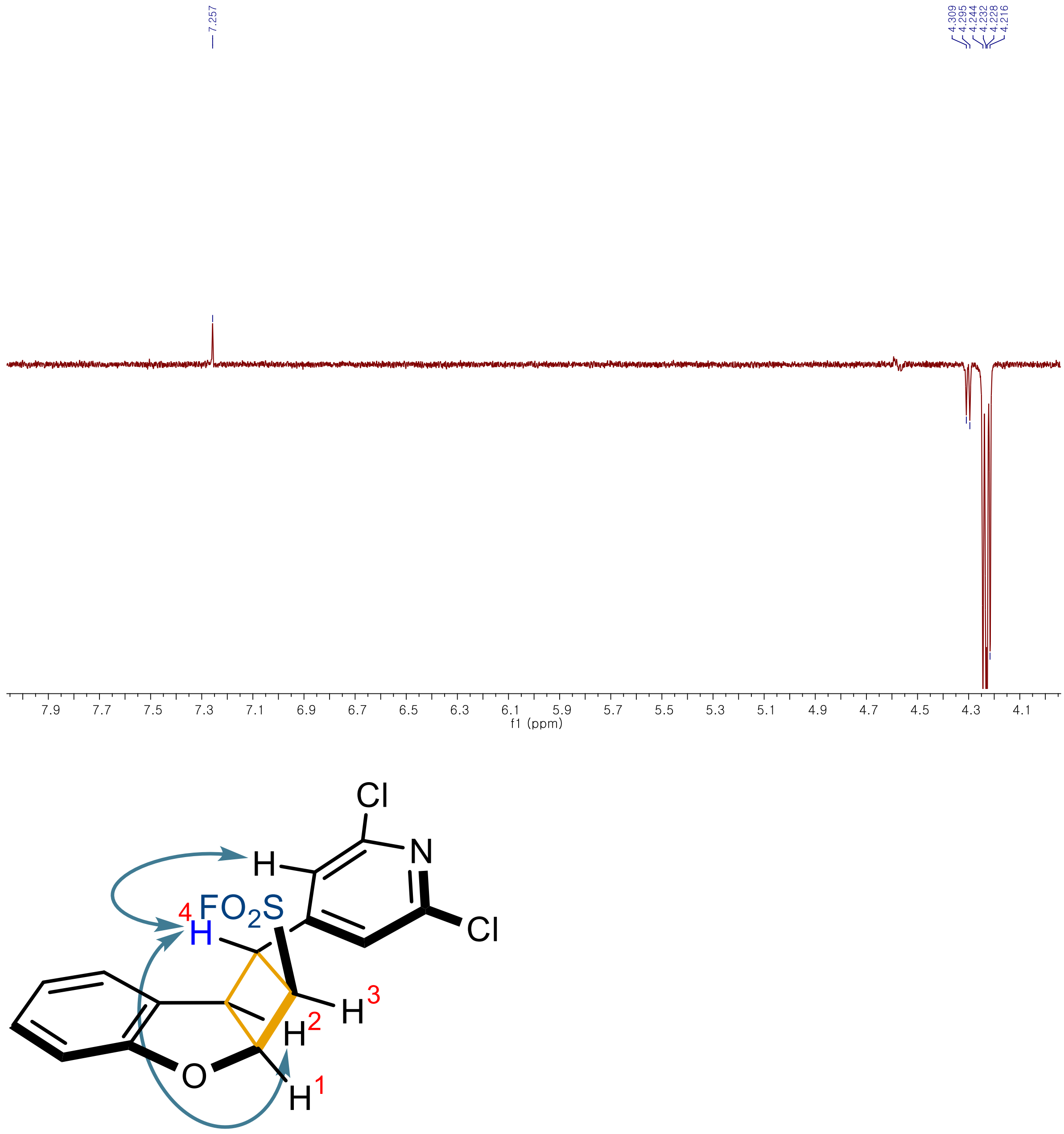

Supplementary Figure 43 1D-NOESY analysis for proton 4 of **3qa**-*endo* (500 MHz, $CDCl_3$, 25 °C).

1D-NOESY spectra of 3ad-*exo*

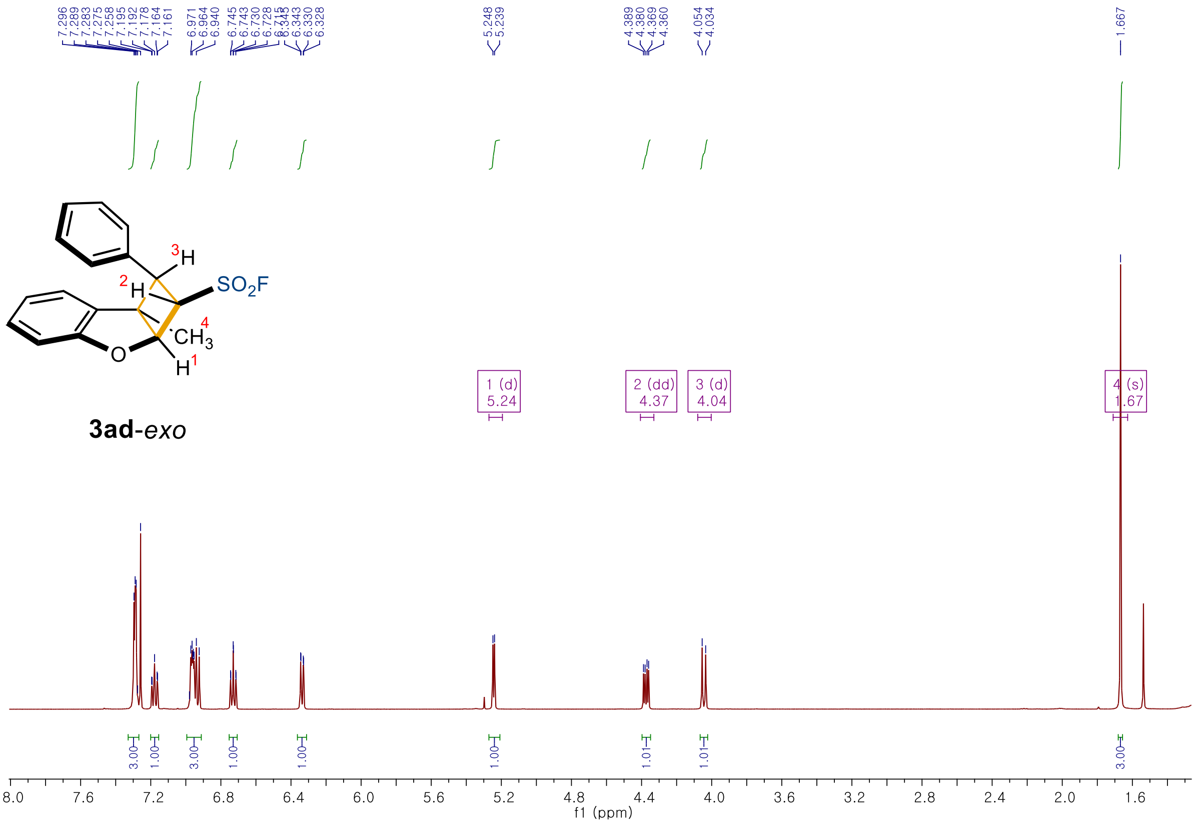

Supplementary Figure 44 ^{1}H NMR spectrum of **3ad**-*exo* (500 MHz, $CDCl_3$, 25 °C).

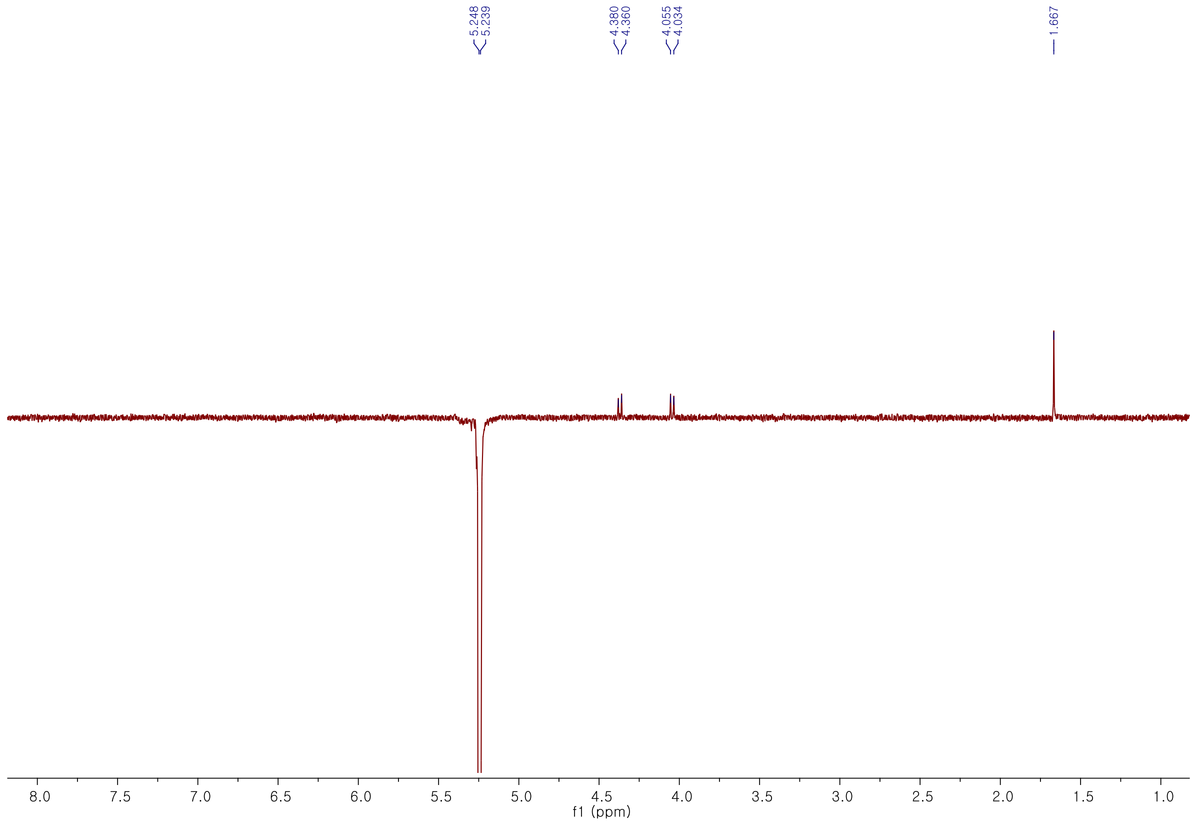

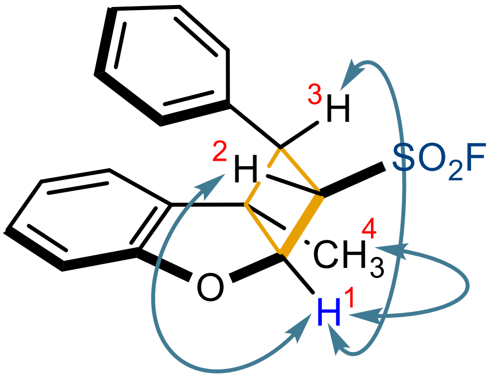

Supplementary Figure 45 1D-NOESY analysis for proton 1 of **3ad**-*exo* (500 MHz, $CDCl_3$, 25 °C).

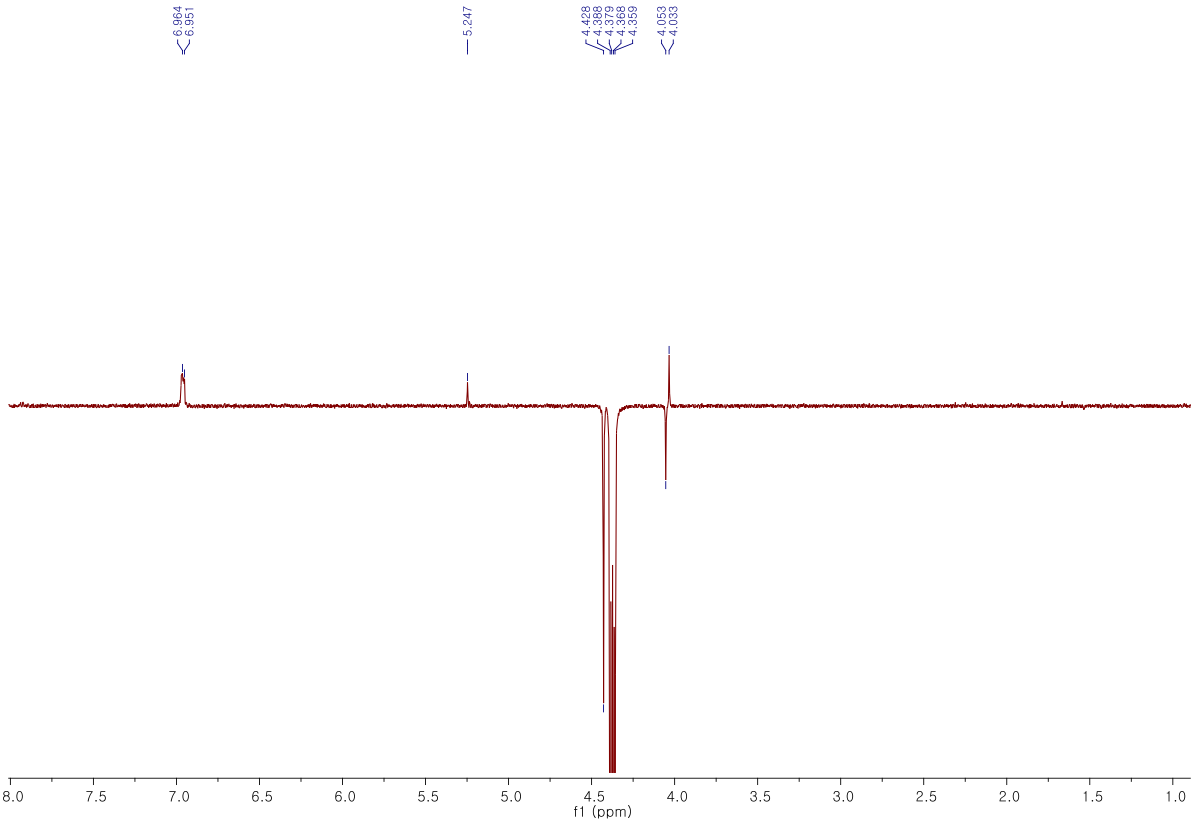

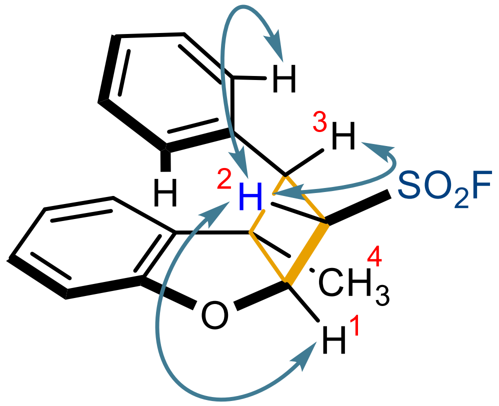

Supplementary Figure 46. 1D-NOESY analysis for proton 2 of **3ad**-*exo* (500 MHz, $CDCl_3$, 25 °C).

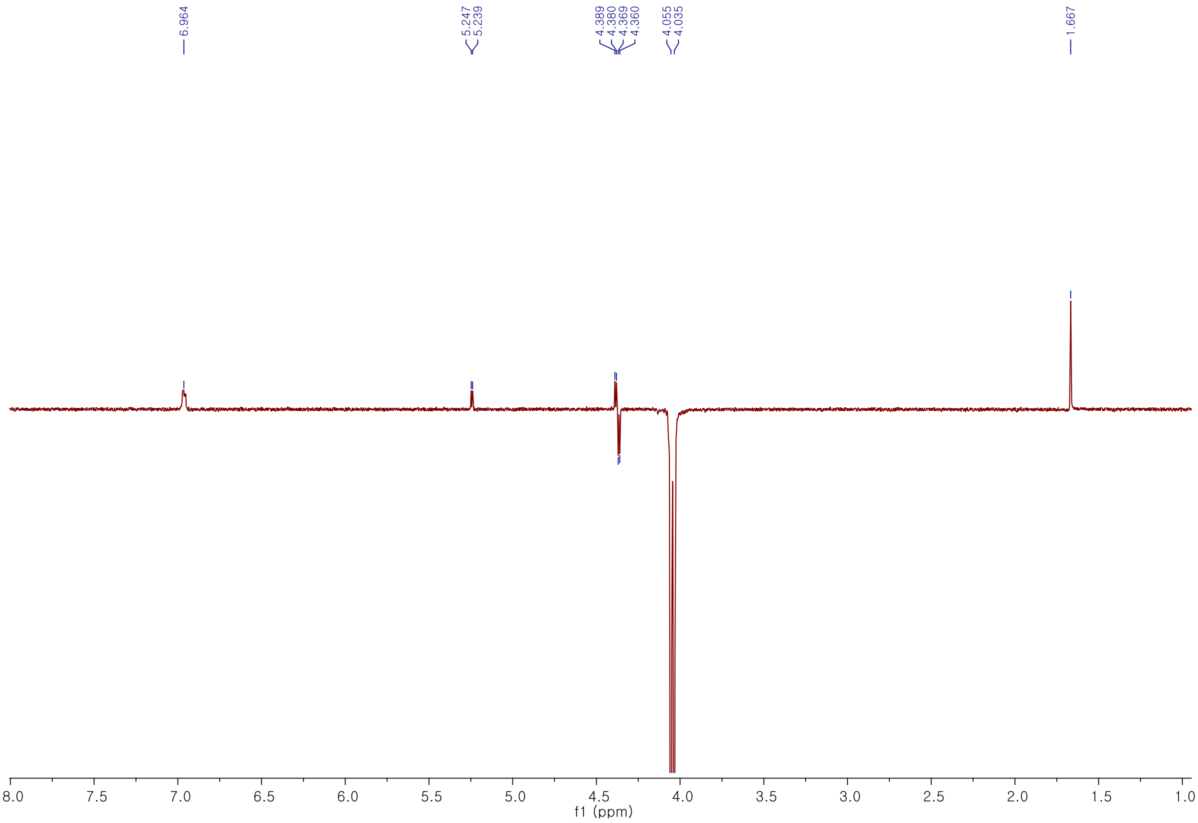

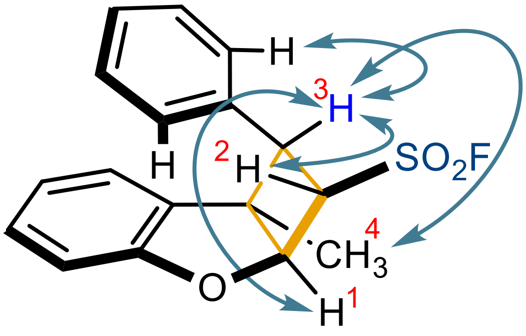

Supplementary Figure 47 1D-NOESY analysis for proton 3 of **3ad**-*exo* (500 MHz, $CDCl_3$, 25 °C).

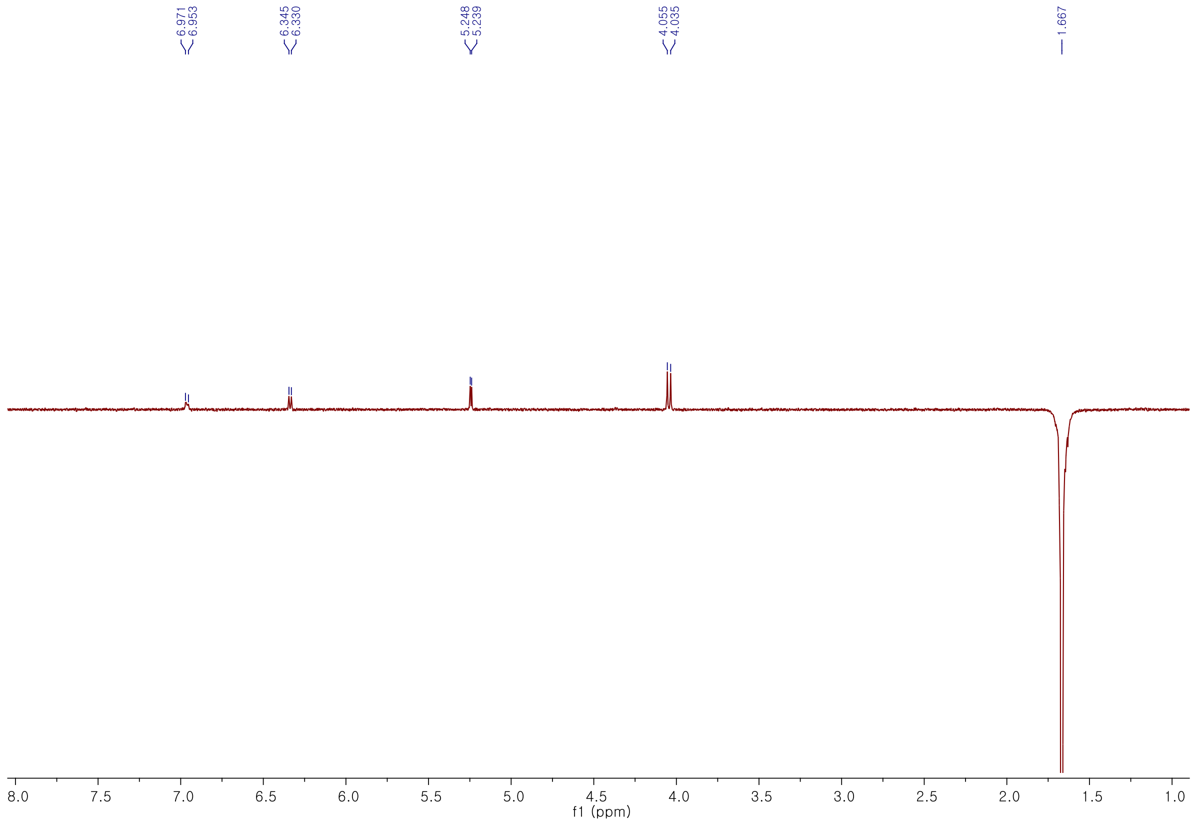

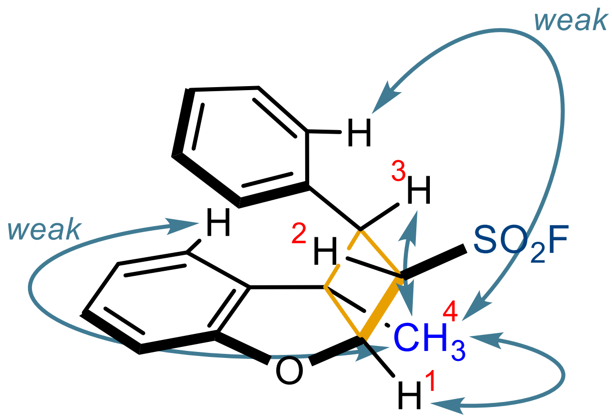

Supplementary Figure 48 1D-NOESY analysis for proton 4 of **3ad**-*exo* (500 MHz, $CDCl_3$, 25 °C).

Comment: Proton 2, close to the sulfonyl fluoride, was observed at δ = 4.37 ppm in ^{1}H NMR. When proton 4 was selectively inverted, protons 1 and 3 were affected, but proton 2 was not. Based on these 1D-NOESY spectra, it was confirmed that proton 2 is close enough to protons 1 and 3, but proton 4 of the methyl group is relatively far away. Therefore, the minor product of **3ad** was determined to be an *exo*-configuration.

1D-NOESY spectra of 3ad-*endo*

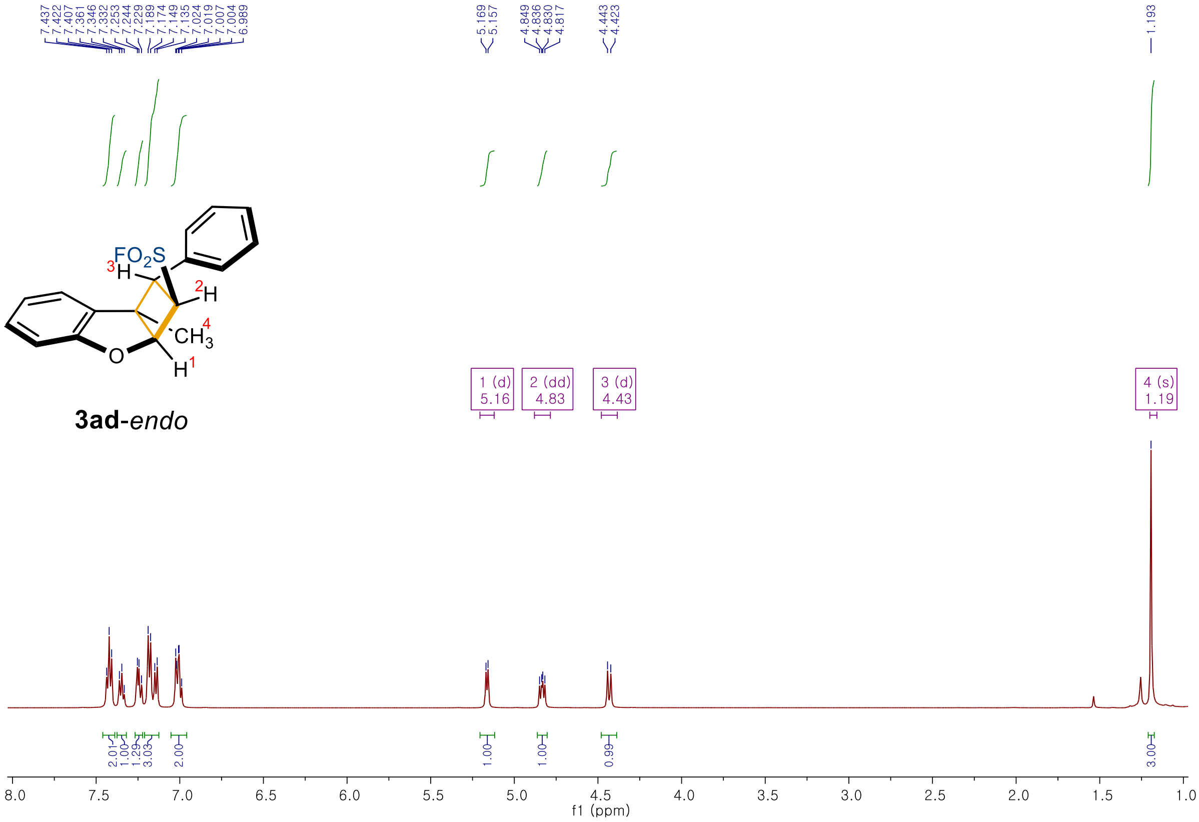

Supplementary Figure 49 ^{1}H NMR spectrum of **3ad**-*endo* (500 MHz, $CDCl_3$, 25 °C).

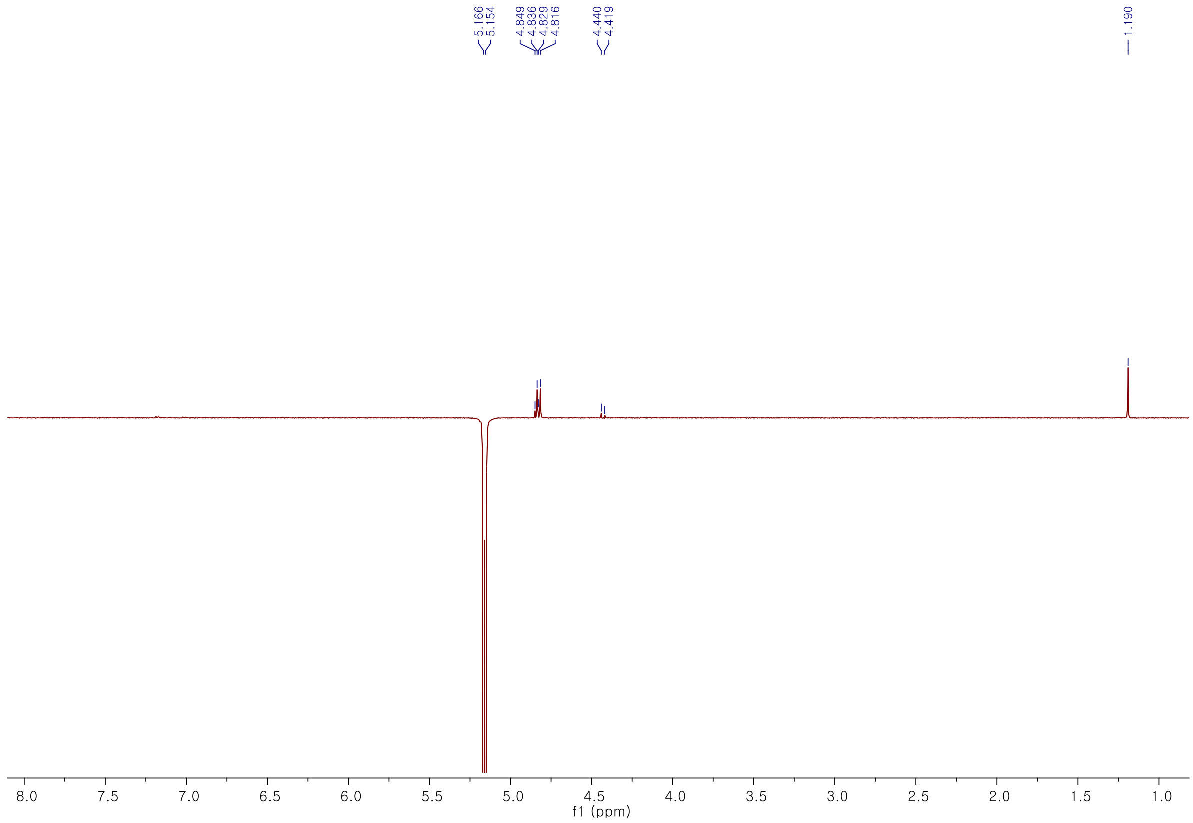

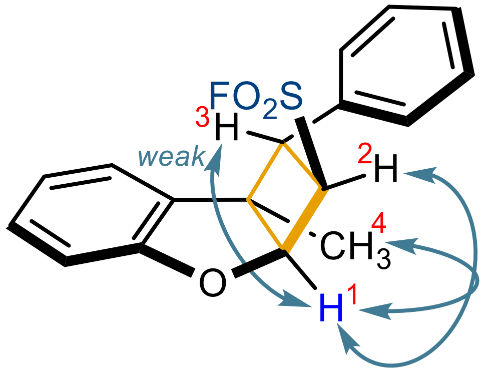

Supplementary Figure 50 1D-NOESY analysis for proton 1 of **3ad**-*endo* (500 MHz, $CDCl_3$, 25 °C).

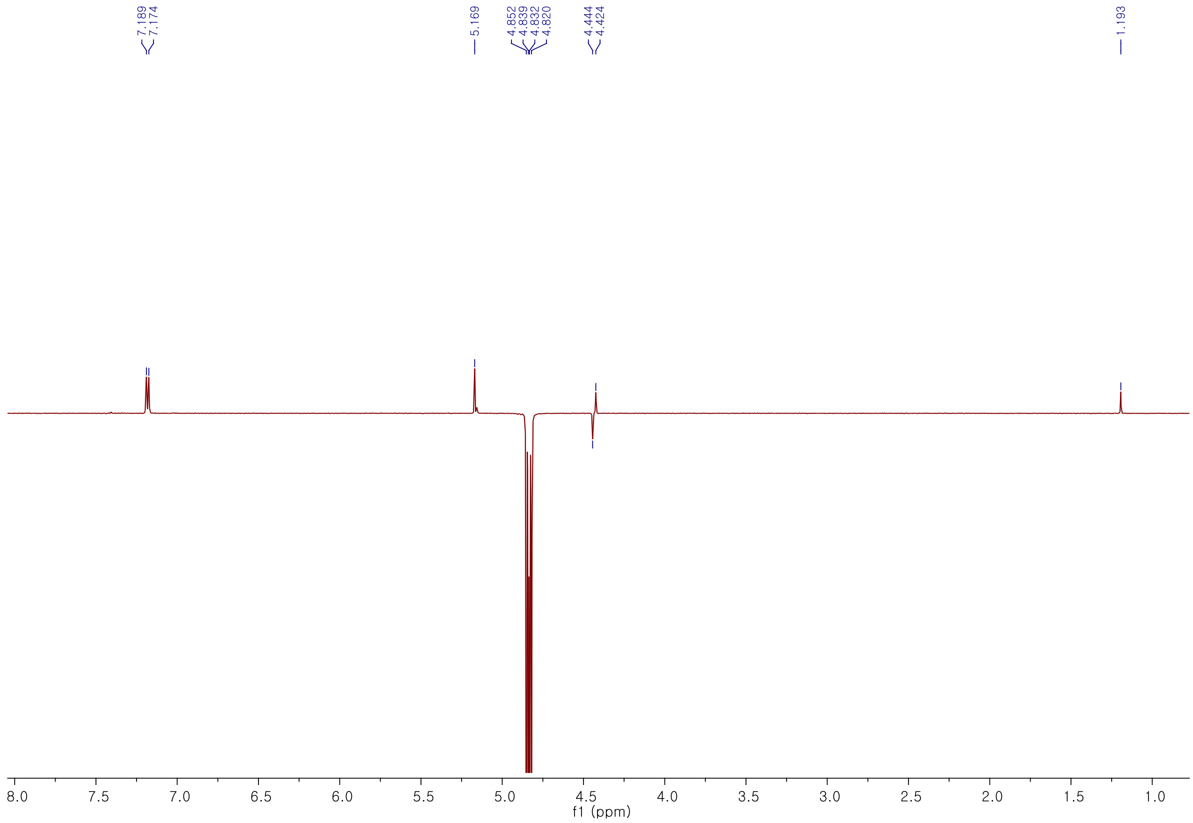

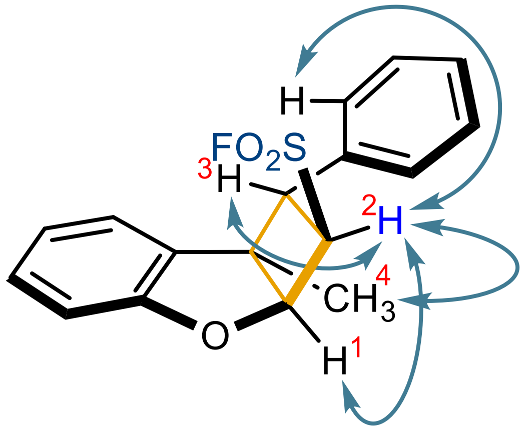

Supplementary Figure 51 1D-NOESY analysis for proton 2 of **3ad**-*endo* (500 MHz, $CDCl_3$, 25 °C).

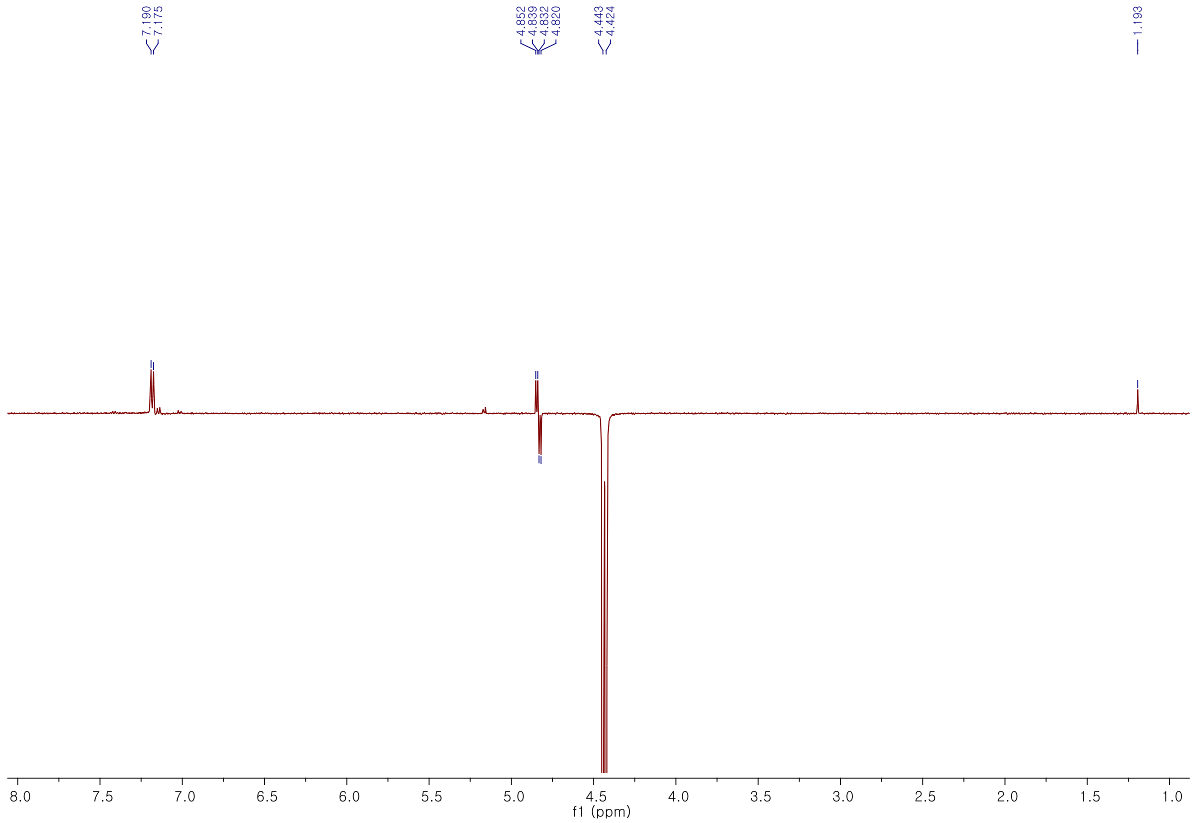

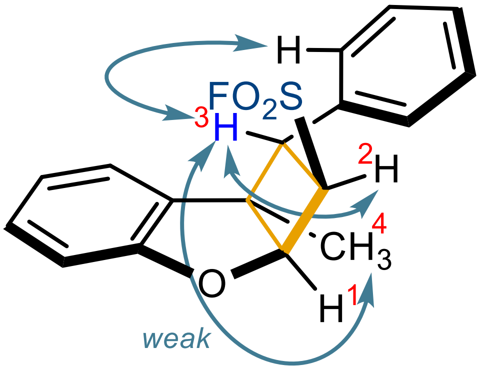

Supplementary Figure 52 1D-NOESY analysis for proton 3 of **3ad**-*endo* (500 MHz, $CDCl_3$, 25 °C).

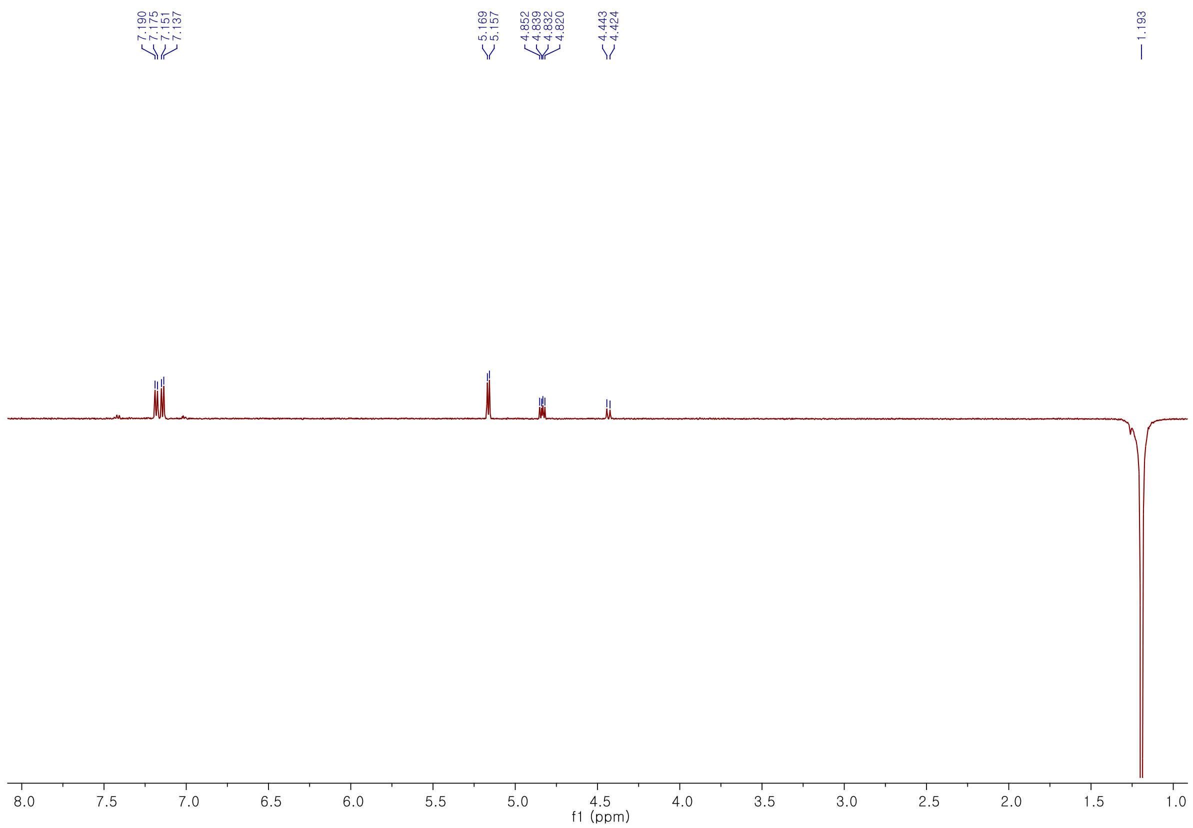

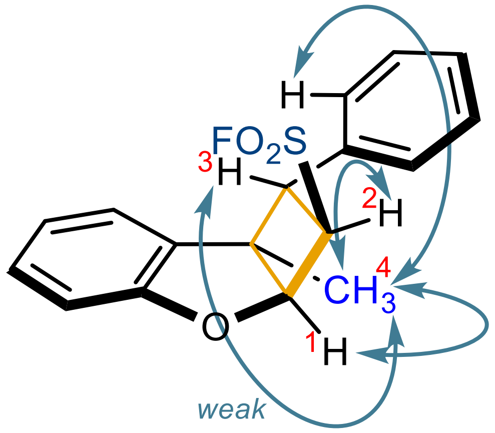

Supplementary Figure 53 1D-NOESY analysis for proton 4 of **3ad**-*endo* (500 MHz, $CDCl_3$, 25 °C).

Comment: When proton 1 was selectively inverted, the peak of proton 3 was observed weakly. This fact means the distance between proton 1 and proton 3 is relatively far. Moreover, when proton 2 was selectively inverted, the peak of proton 4 of the methyl group was observed, unlike the minor product. This is a valid difference from the minor product (*exo*-configuration), and also means that the methyl group of the major product is oriented in the same direction as proton 2. Therefore, the major product of **3ad** is determined to be an *endo*-configuration.

1D-NOESY spectra of 3aj-*exo*

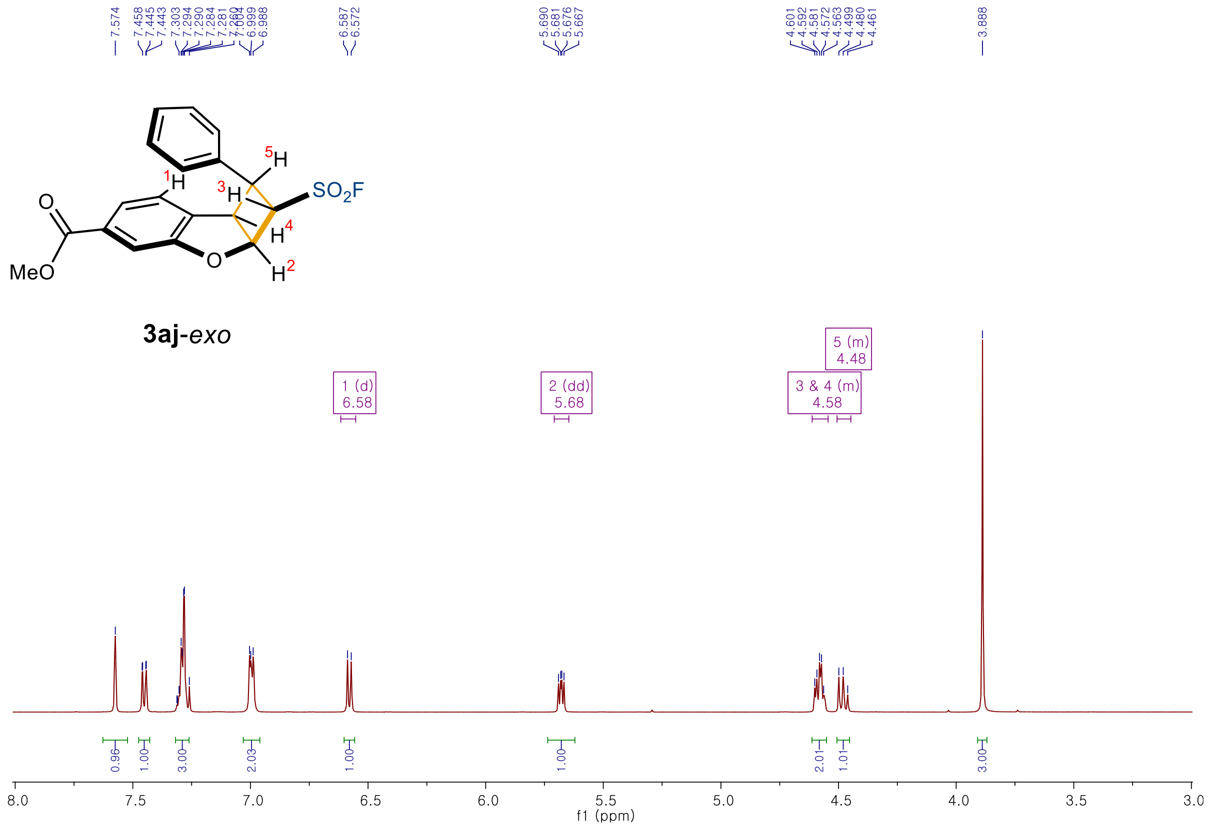

Supplementary Figure 54 ^{1}H NMR spectrum of **3aj**-*exo* (500 MHz, $CDCl_3$, 25 °C).

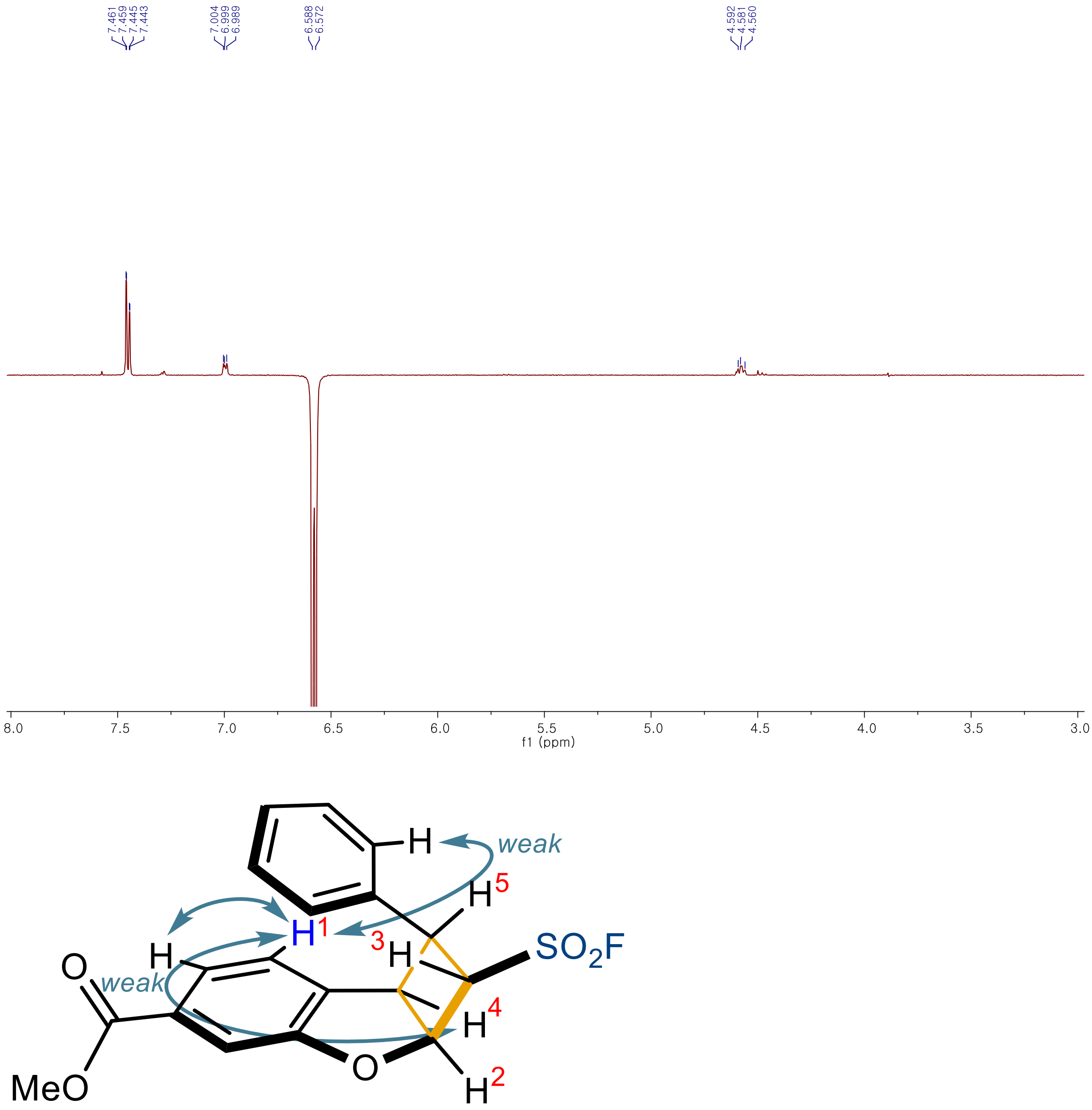

Supplementary Figure 55 1D-NOESY analysis for proton 1 of **3aj**-*exo* (500 MHz, $CDCl_3$, 25 °C).

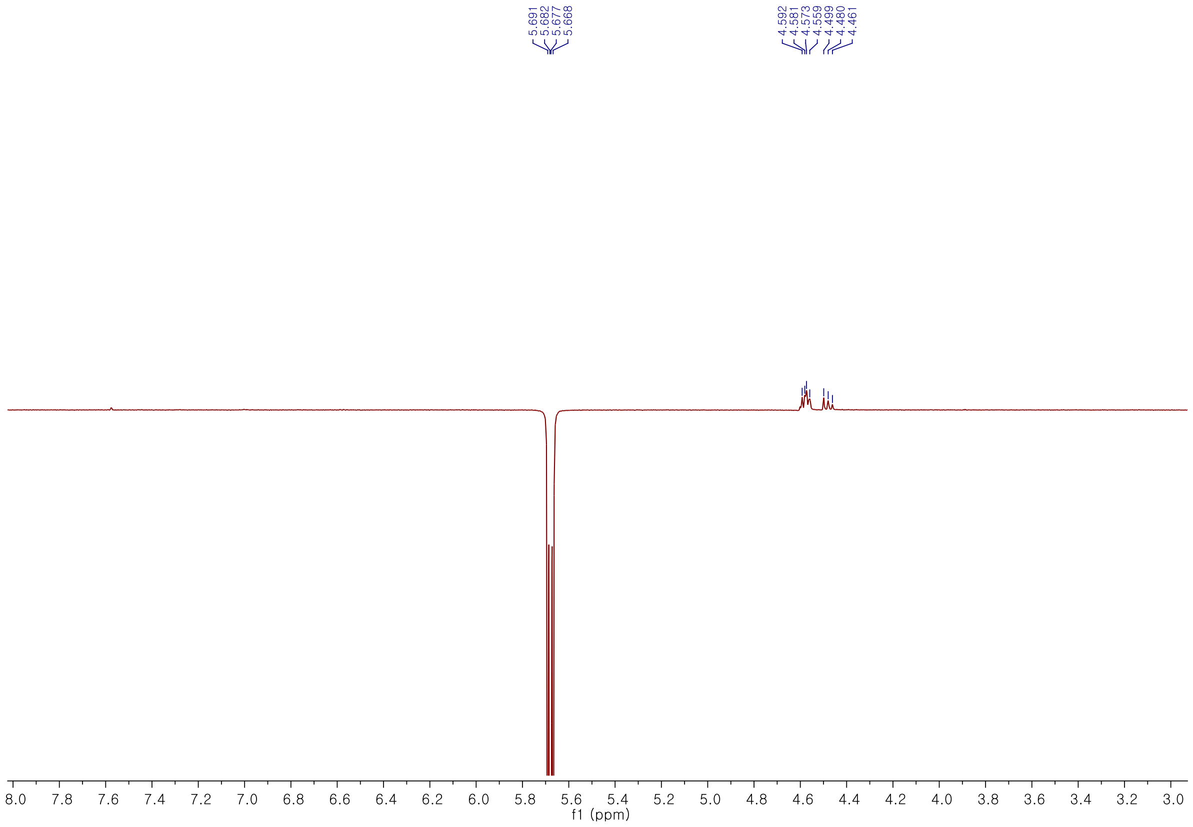

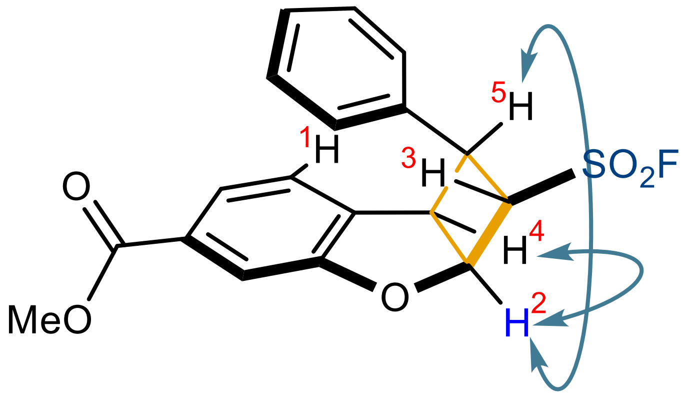

Supplementary Figure 56 1D-NOESY analysis for proton 2 of **3aj**-*exo* (500 MHz, $CDCl_3$, 25 °C).

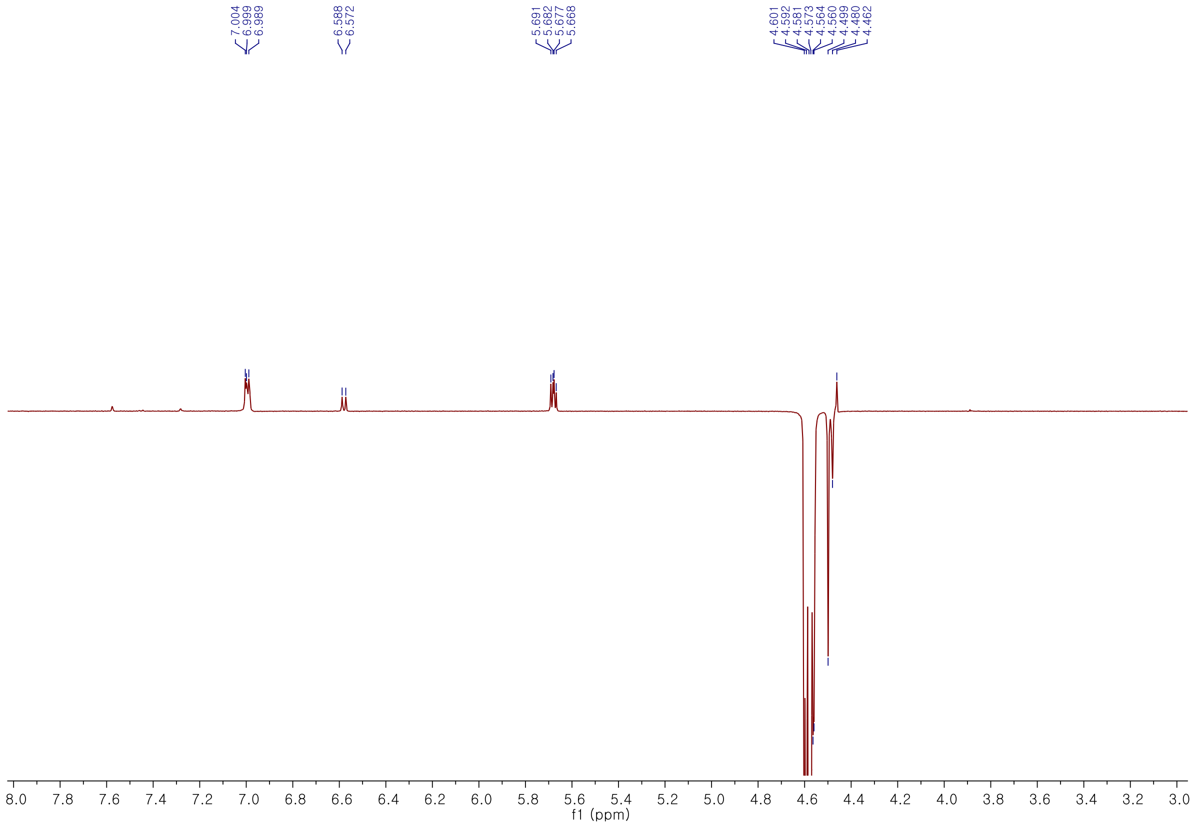

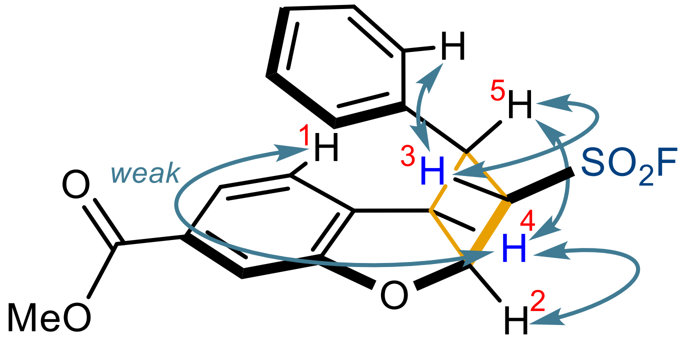

Supplementary Figure 57 1D-NOESY analysis for proton 3 and 4 of **3aj**-*exo* (500 MHz, $CDCl_3$, 25 °C).

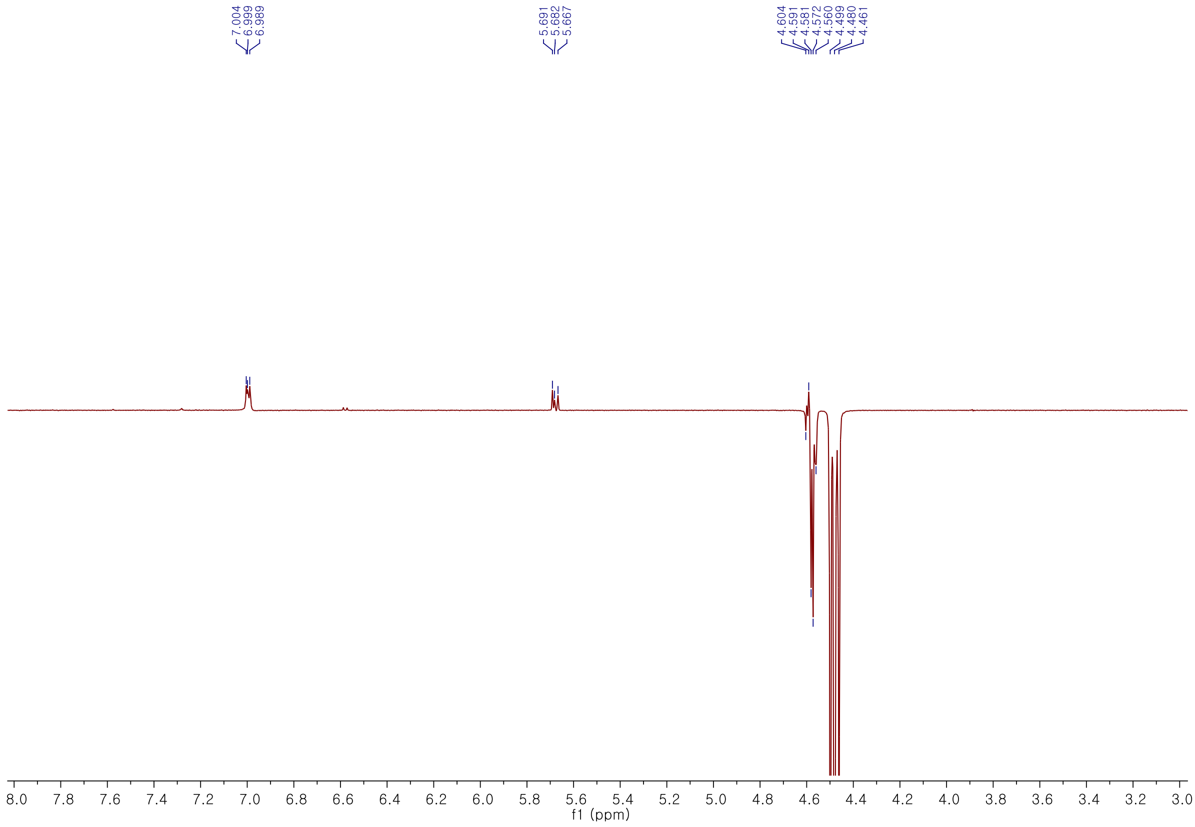

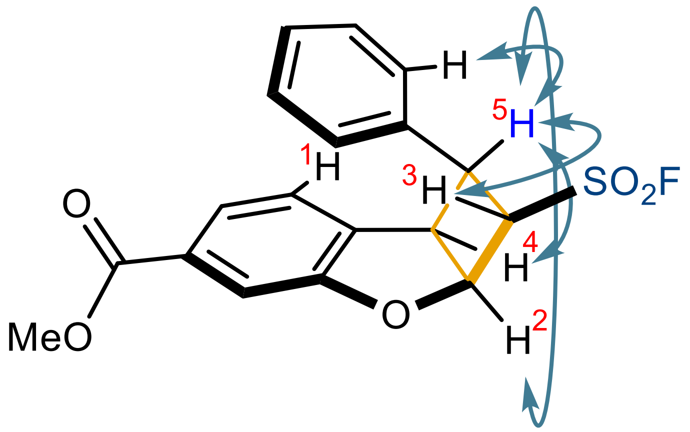

Supplementary Figure 58 1D-NOESY analysis for proton 5 of **3aj**-*exo* (500 MHz, $CDCl_3$, 25 °C).

Comment: Although the benzofuran at position 6 was substituted, specific change for protons present in the cyclobutane moiety was not observed in 1D-NOESY analysis. Therefore, the determination for *exo-/endo*-configuration is similar to **3aa**-*exo* and **3aa**-e*ndo*. When proton 2 was selectively inverted, the peak of proton 3 was not observed. This fact means the distance between proton 2 and proton 3 is relatively far. Moreover, when proton 5 was selectively inverted, protons 2, 3 and 4 of the cyclobutane moiety are all affected. Therefore, proton 2 is oriented in the opposite direction with proton 3, and proton 5 is oriented in the same direction with proton 2 and 4. Therefore, obtained product is determined to be an *exo*-configuration.

1D-NOESY spectra of 3aj-*endo*

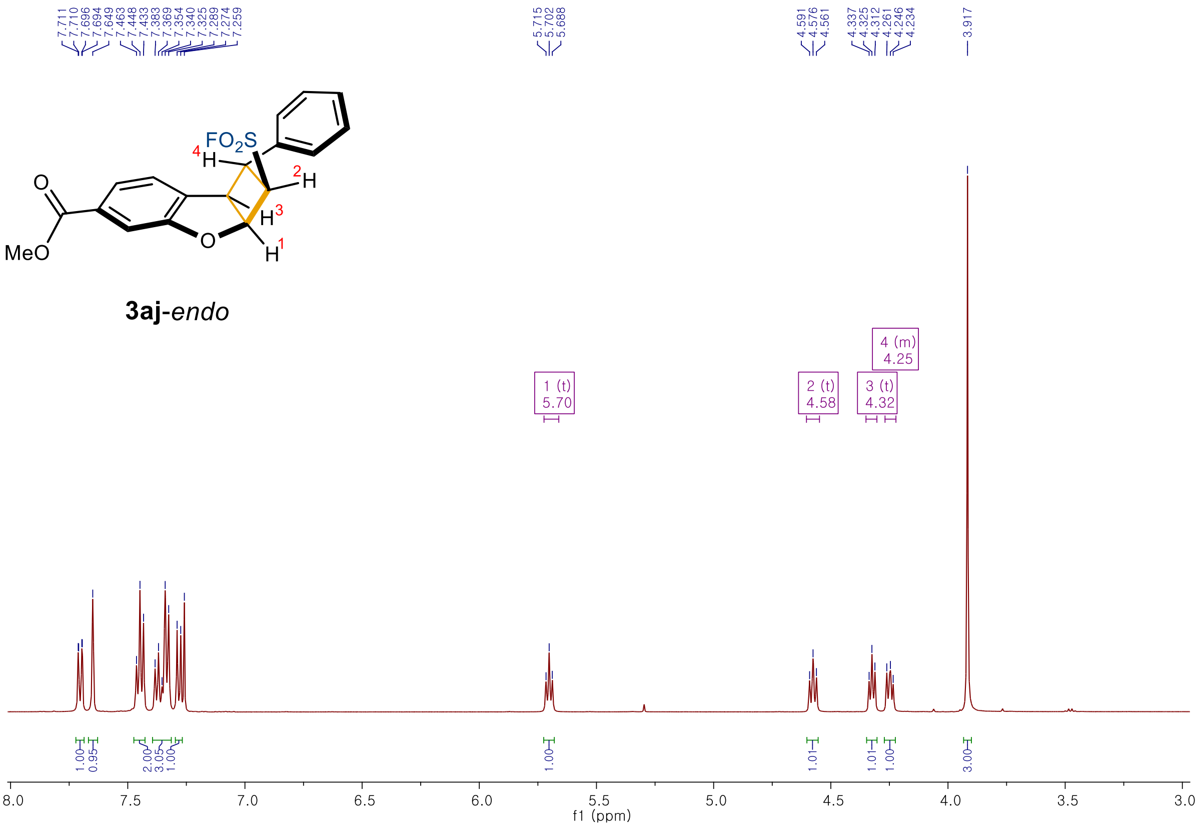

Supplementary Figure 59 ^{1}H NMR spectrum of **3aj**-*endo* (500 MHz, $CDCl_3$, 25 °C).

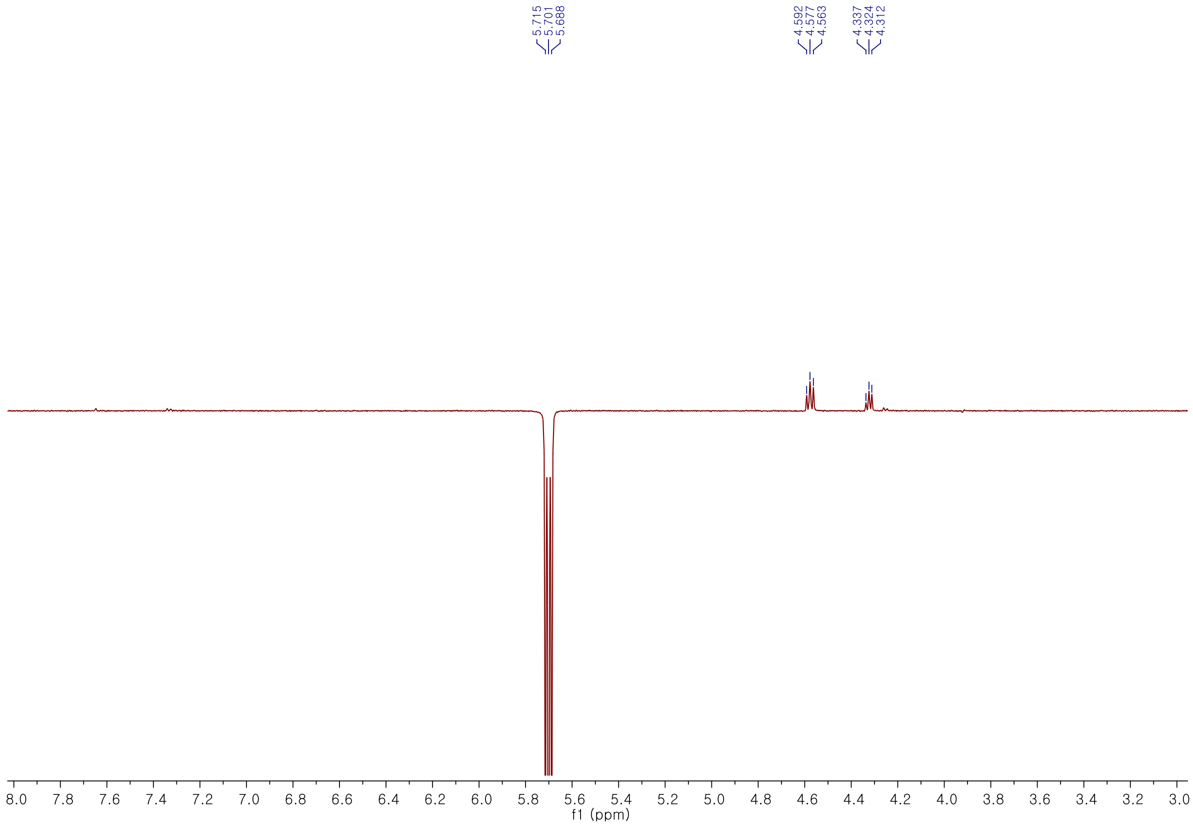

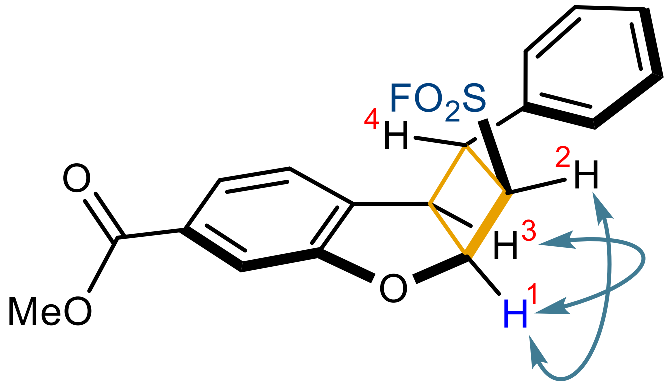

Supplementary Figure 60 1D-NOESY analysis for proton 1 of **3aj**-*endo* (500 MHz, $CDCl_3$, 25 °C).

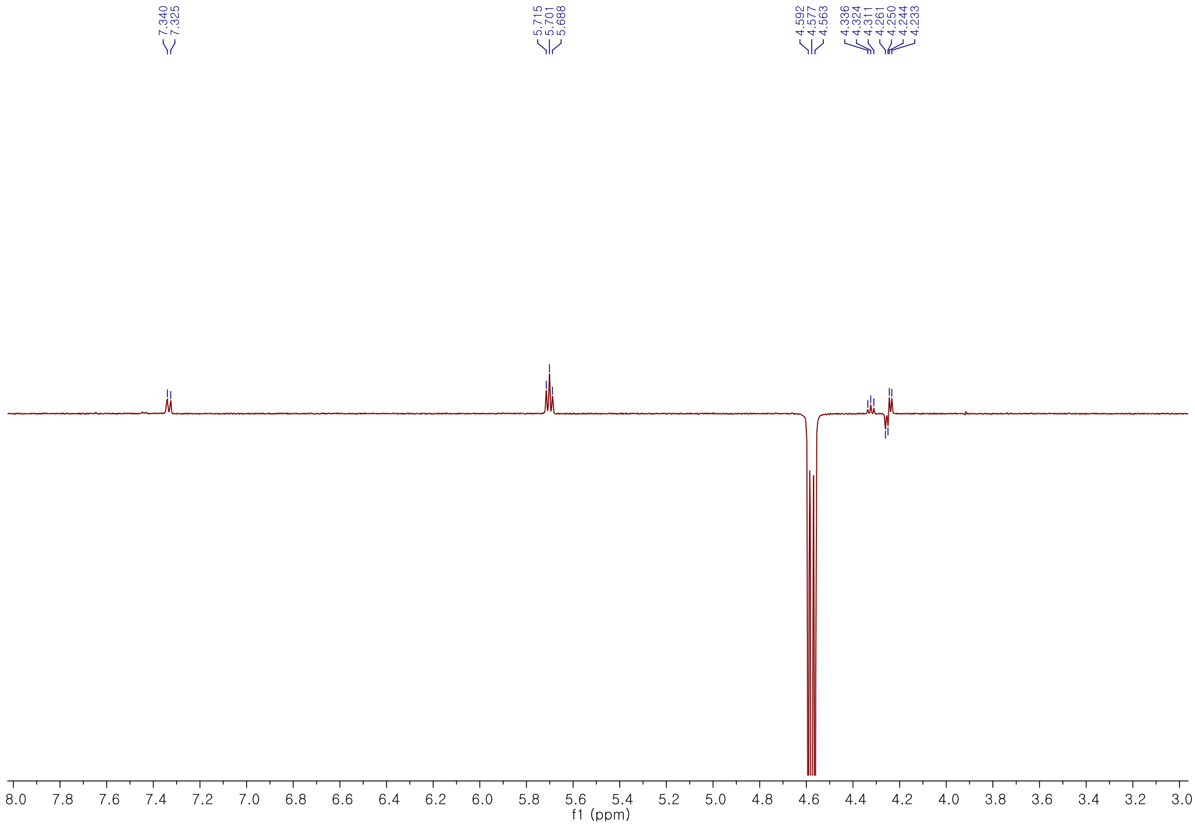

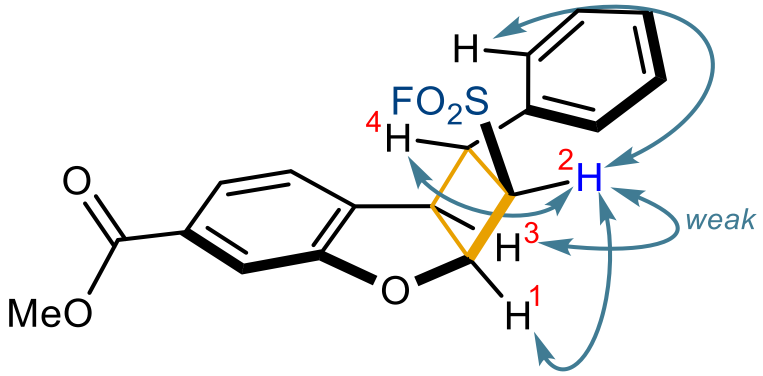

Supplementary Figure 61 1D-NOESY analysis for proton 2 of **3aj**-*endo* (500 MHz, $CDCl_3$, 25 °C).

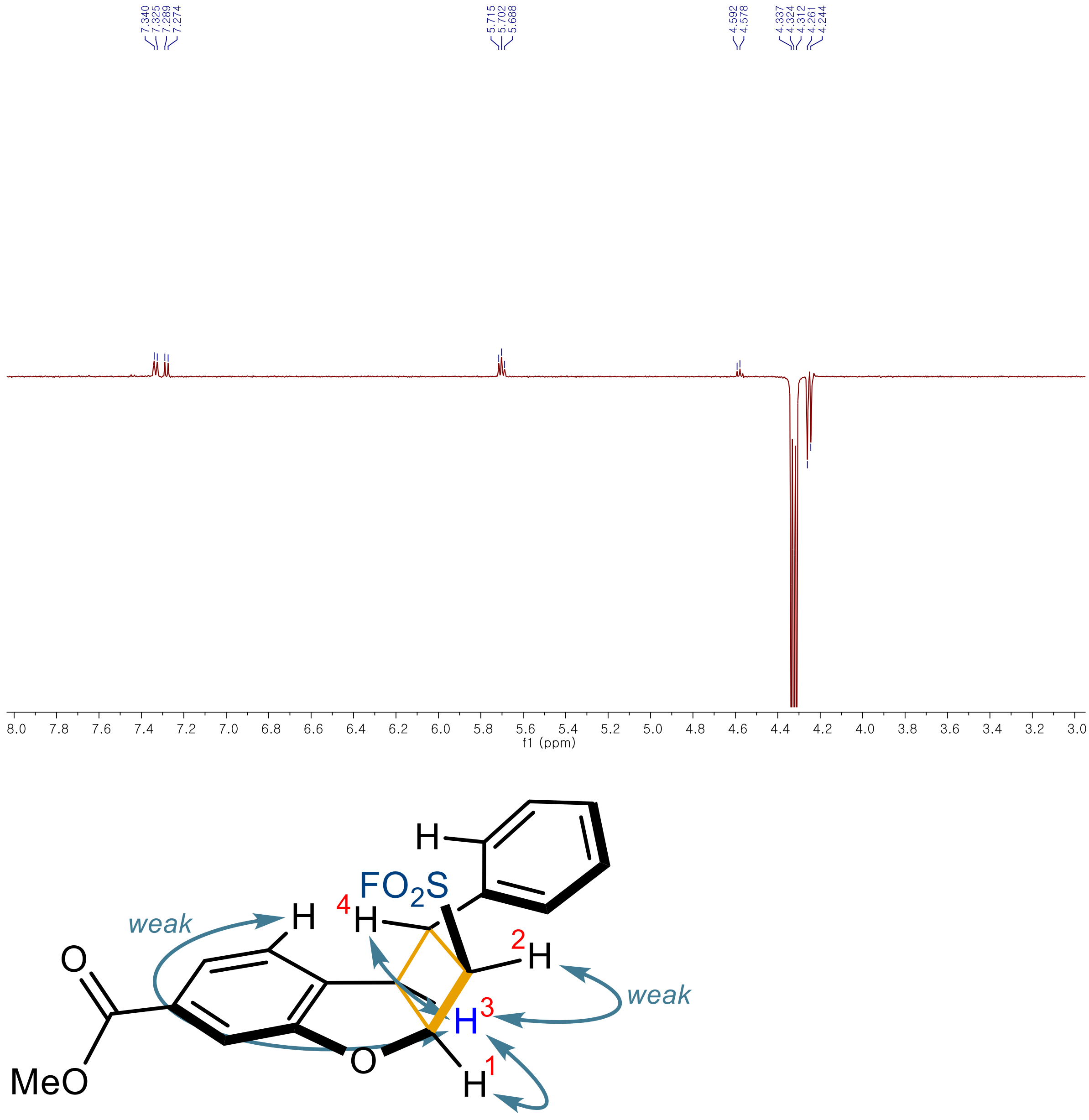

Supplementary Figure 62 1D-NOESY analysis for proton 3 of **3aj**-*endo* (500 MHz, $CDCl_3$, 25 °C).

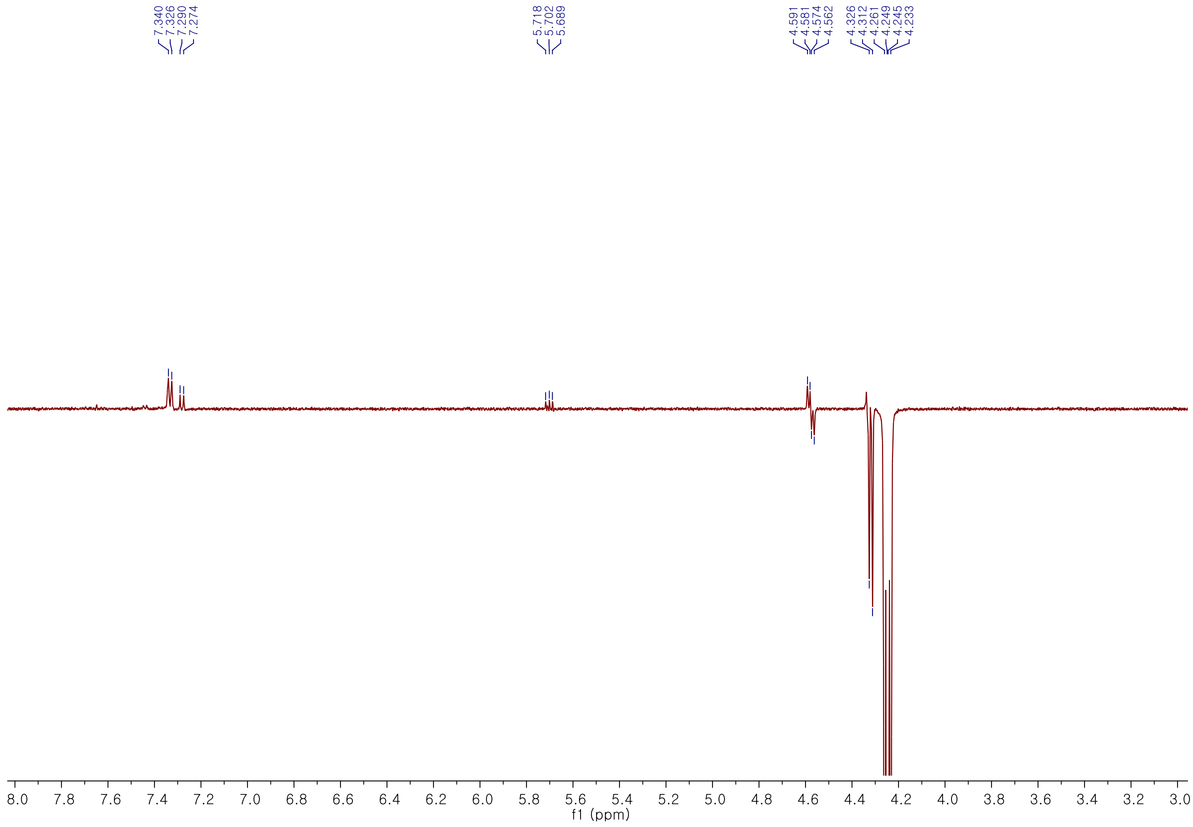

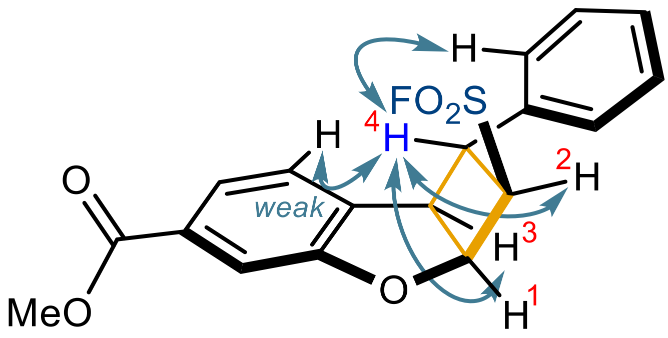

Supplementary Figure 63 1D-NOESY analysis for proton 4 of **3aj**-*endo* (500 MHz, $CDCl_3$, 25 °C).

Structural analysis of Single crystal X-ray

X-ray crystal structure of 3aa-*exo* (CCDC-2279317)

Crystal data and structure refinement for **3aa**-*exo*.

Identification code	3a_exo_a	
Empirical formula	C16 H13 F O3 S	
Formula weight	304.32	
Temperature	223(2) K	
Wavelength	0.71073 Å	
Crystal system	Triclinic	
Space group	P-1	
Unit cell dimensions	a = 9.309(4) Å	a= 116.248(14)°.
	b = 15.733(8) Å	b= 91.149(13)°.
	c = 16.748(8) Å	g = 98.665(14)°.
Volume	2164.6(18) Å^3	
Z	6	
Density (calculated)	1.401 Mg/m^3	
Absorption coefficient	0.242 mm^{-1}	
F(000)	948	
Crystal size	0.146 x 0.114 x 0.040 mm^3	
Theta range for data collection	2.410 to 26.102°.	
Index ranges	-11<=h<=11, -19<=k<=19, -20<=l<=20	
Reflections collected	58614	
Independent reflections	8559 [R(int) = 0.1342]	
Completeness to theta = 25.242°	99.9 %	
Absorption correction	Semi-empirical from equivalents	
Max. and min. transmission	0.7453 and 0.6820	
Refinement method	Full-matrix least-squares on F^2	
Data / restraints / parameters	8559 / 0 / 570	
Goodness-of-fit on F^2	0.994	
Final R indices [I>2sigma(I)]	R1 = 0.0643, wR2 = 0.1640	
R indices (all data)	R1 = 0.1571, wR2 = 0.2217	
Extinction coefficient	n/a	
Largest diff. peak and hole	0.369 and -0.443 e.Å^{-3}	

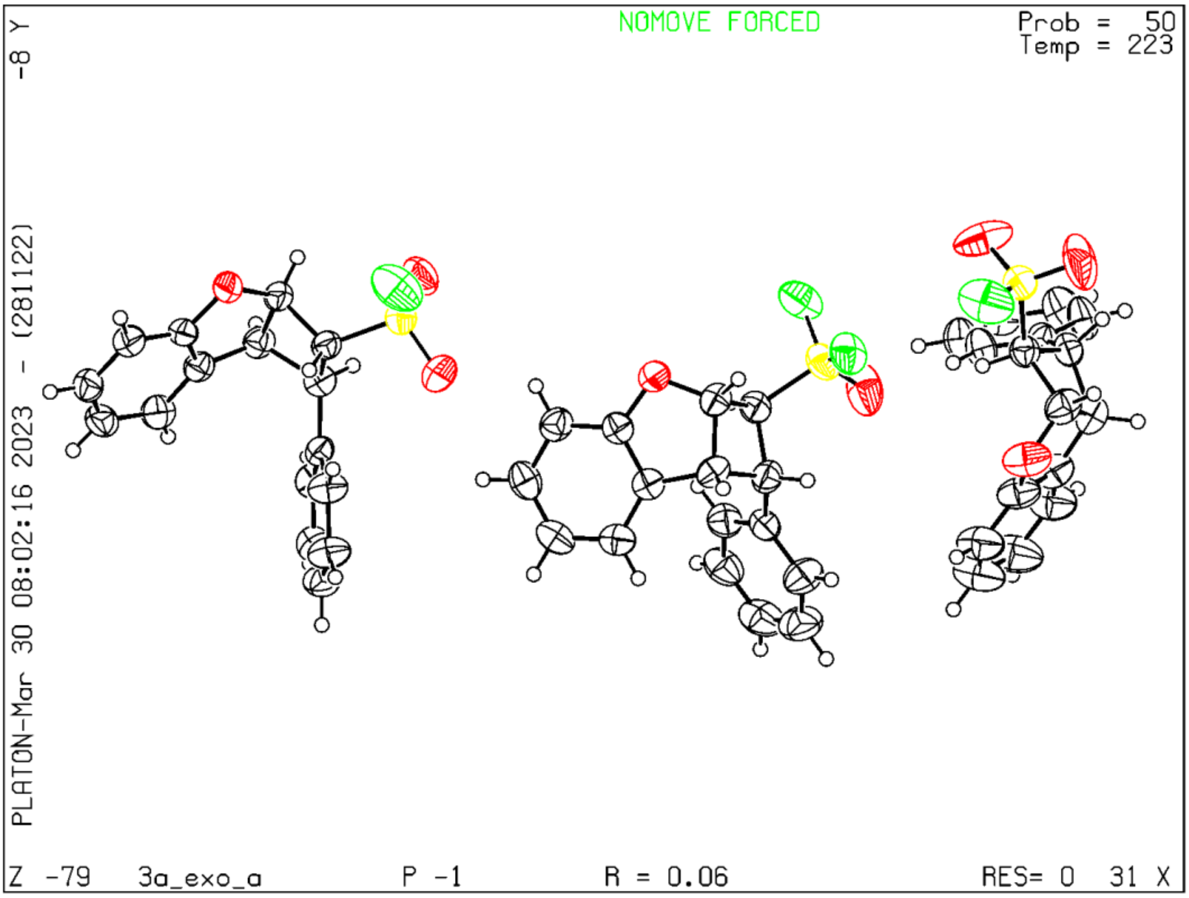

Supplementary Figure 64 ORTEP structure of **3aa**-*exo*.

X-ray crystal structure of 3aa-*endo* (CCDC-2279318)

Crystal data and structure refinement for **3aa**-*endo*.

Identification code	3a_endo_a	
Empirical formula	C16 H13 F O3 S	
Formula weight	304.32	
Temperature	223(2) K	
Wavelength	0.71073 Å	
Crystal system	Monoclinic	
Space group	$P2_1/c$	
Unit cell dimensions	a = 10.2953(6) Å	a= 90°.
	b = 15.5574(8) Å	b= 117.242(2)°.
	c = 9.6117(5) Å	g = 90°.
Volume	1368.73(13) $Å^3$	
Z	4	
Density (calculated)	1.477 Mg/m^3	
Absorption coefficient	0.255 mm^{-1}	
F(000)	632	
Crystal size	0.110 x 0.100 x 0.093 mm^3	
Theta range for data collection	2.225 to 28.381°.	
Index ranges	-13<=h<=13, -20<=k<=20, -10<=l<=12	
Reflections collected	26857	
Independent reflections	3415 [R(int) = 0.0512]	
Completeness to theta = 25.242°	99.9 %	
Absorption correction	Semi-empirical from equivalents	
Max. and min. transmission	0.7457 and 0.6578	
Refinement method	Full-matrix least-squares on F^2	
Data / restraints / parameters	3415 / 0 / 191	
Goodness-of-fit on F^2	1.041	
Final R indices [I>2sigma(I)]	R1 = 0.0404, wR2 = 0.0944	
R indices (all data)	R1 = 0.0689, wR2 = 0.1118	
Extinction coefficient	n/a	
Largest diff. peak and hole	0.283 and -0.315 e.$Å^{-3}$	

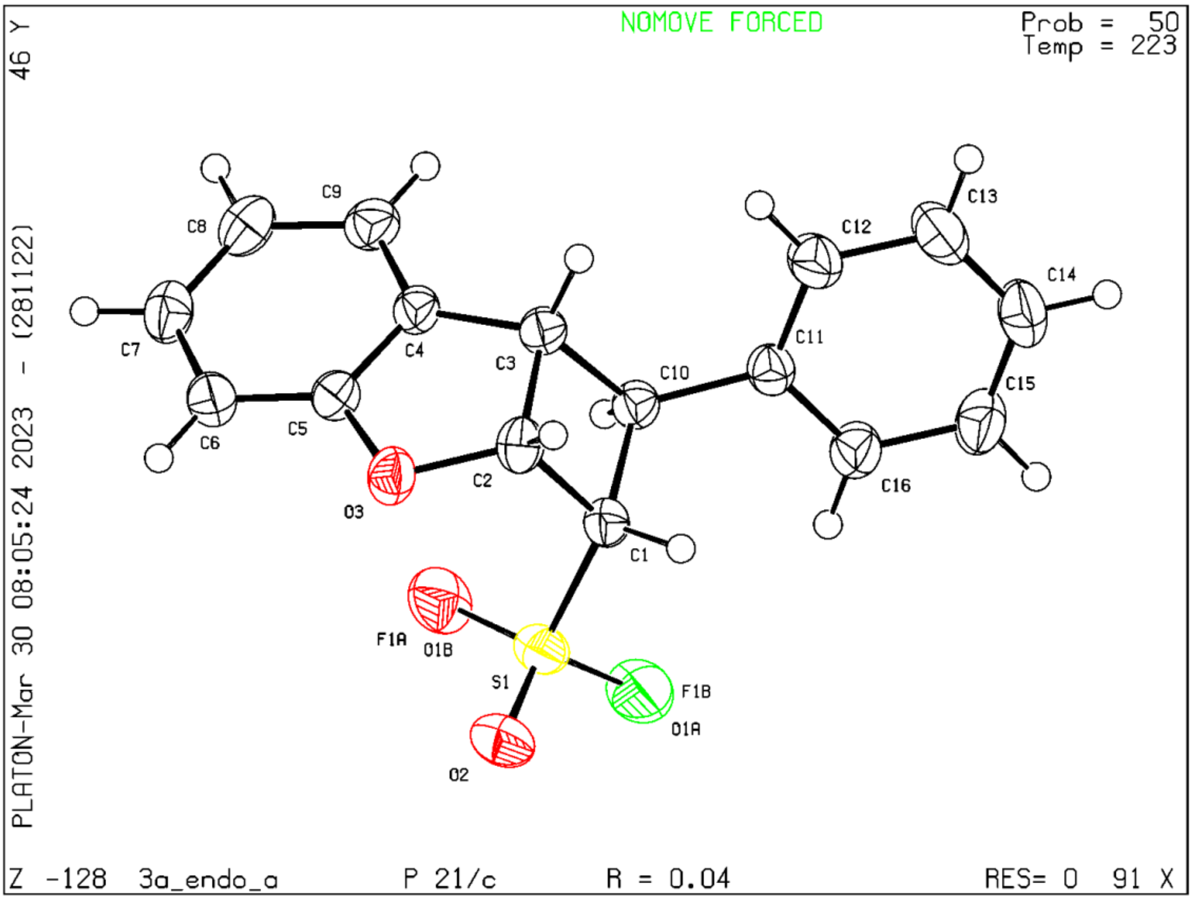

Supplementary Figure 65 ORTEP structure of **3aa**-*endo*.

X-ray crystal structure of 3da-*exo* (CCDC-2279322)

Crystal data and structure refinement for **3da**-*exo*.

Identification code	sample1_a	
Empirical formula	C17 H15 F O3 S	
Formula weight	318.35	
Temperature	223(2) K	
Wavelength	0.71073 Å	
Crystal system	Monoclinic	
Space group	$P2_1/c$	
Unit cell dimensions	a = 5.2299(5) Å	a= 90°.
	b = 18.610(2) Å	b= 98.254(3)°.
	c = 15.7094(17) Å	g = 90°.
Volume	1513.1(3) $Å^3$	
Z	4	
Density (calculated)	1.397 Mg/m^3	
Absorption coefficient	0.234 mm^{-1}	
F(000)	664	
Crystal size	0.390 x 0.054 x 0.036 mm^3	
Theta range for data collection	2.189 to 28.313°.	
Index ranges	-6<=h<=5, -24<=k<=24, -20<=l<=20	
Reflections collected	28319	
Independent reflections	3747 [R(int) = 0.0745]	
Completeness to theta = 25.242°	100.0 %	
Absorption correction	Semi-empirical from equivalents	
Max. and min. transmission	0.7457 and 0.6836	
Refinement method	Full-matrix least-squares on F^2	
Data / restraints / parameters	3747 / 6 / 200	
Goodness-of-fit on F^2	1.051	
Final R indices [I>2sigma(I)]	R1 = 0.0477, wR2 = 0.1090	
R indices (all data)	R1 = 0.1061, wR2 = 0.1402	
Extinction coefficient	n/a	
Largest diff. peak and hole	0.246 and -0.348 e.$Å^{-3}$	

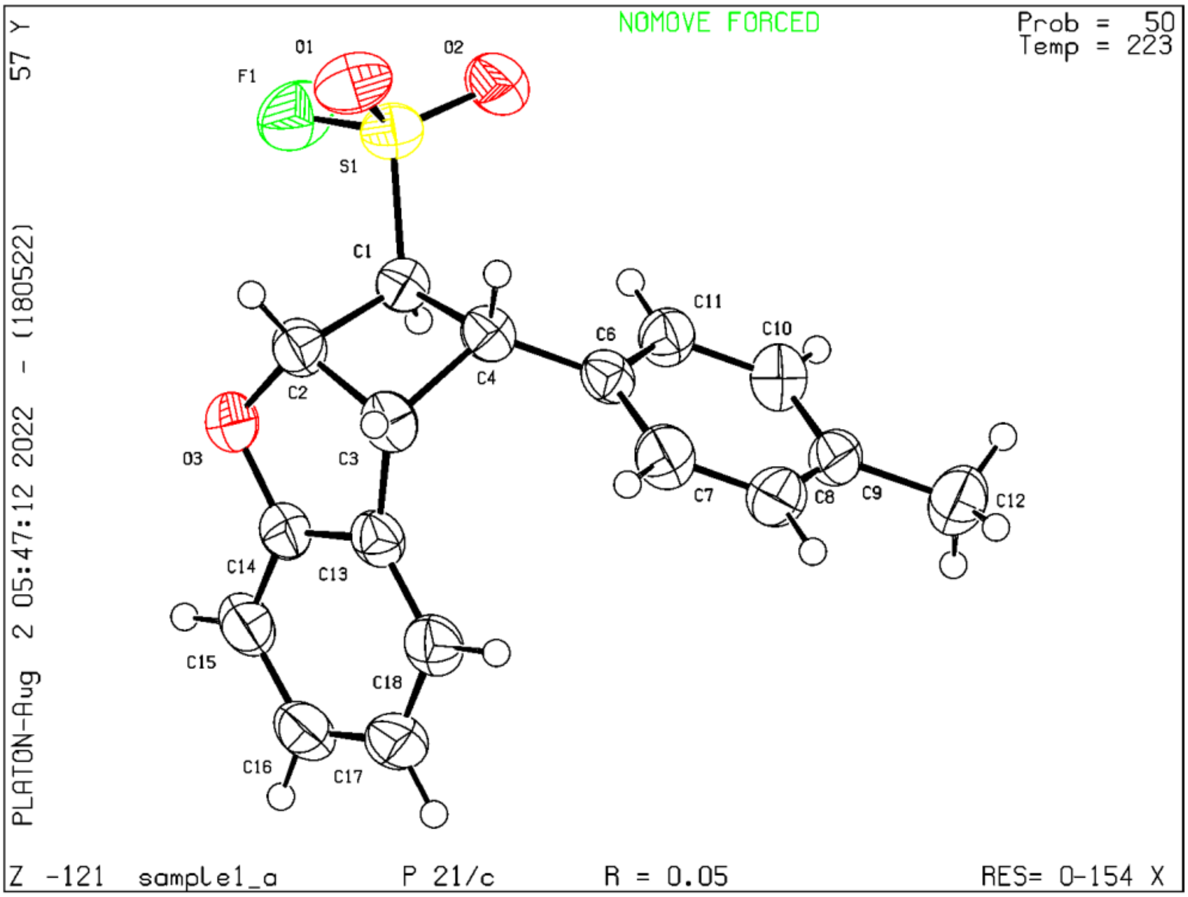

Supplementary Figure 66 ORTEP structure of **3da**-*exo*.

X-ray crystal structure of 3ma-*exo* (CCDC-2279321)

Crystal data and structure refinement for **3ma**-*exo*.

Identification code	22cycloadduct_a	
Empirical formula	C22 H17 F O3 S	
Formula weight	380.41	
Temperature	223(2) K	
Wavelength	0.71073 Å	
Crystal system	Orthorhombic	
Space group	$Pna2_1$	
Unit cell dimensions	a = 16.6920(9) Å	a= 90°.
	b = 18.5028(10) Å	b= 90°.
	c = 5.7219(3) Å	g = 90°.
Volume	1767.20(16) $Å^3$	
Z	4	
Density (calculated)	1.430 Mg/m^3	
Absorption coefficient	0.214 mm^{-1}	
F(000)	792	
Crystal size	0.400 x 0.033 x 0.030 mm^3	
Theta range for data collection	2.201 to 28.445°.	
Index ranges	-22<=h<=22, -24<=k<=24, -7<=l<=5	
Reflections collected	34535	
Independent reflections	3898 [R(int) = 0.0913]	
Completeness to theta = 25.242°	99.9 %	
Absorption correction	Semi-empirical from equivalents	
Max. and min. transmission	0.7457 and 0.6904	
Refinement method	Full-matrix least-squares on F^2	
Data / restraints / parameters	3898 / 1 / 245	
Goodness-of-fit on F^2	1.084	
Final R indices [I>2sigma(I)]	R1 = 0.0452, wR2 = 0.0986	
R indices (all data)	R1 = 0.0889, wR2 = 0.1190	
Absolute structure parameter	0.03(6)	
Extinction coefficient	0.0067(13)	
Largest diff. peak and hole	0.219 and -0.252 $e.Å^{-3}$	

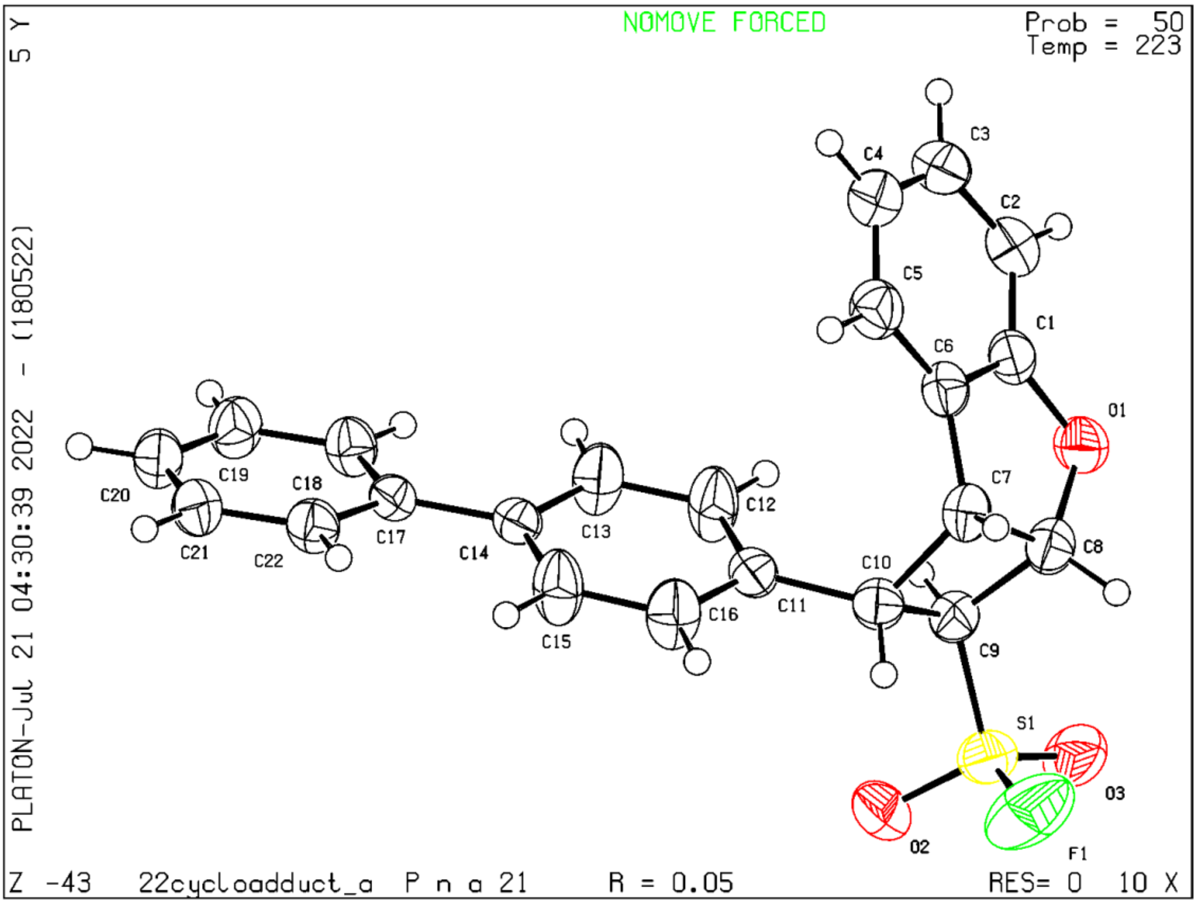

Supplementary Figure 67 ORTEP structure of **3ma**-*exo*.

X-ray crystal structure of 3ab-*exo* (CCDC-2295179)

Crystal data and structure refinement for **3ab**-*exo*.

Identification code	3ab_exo_2_a_pl	
Empirical formula	C16 H13 F O2 S2	
Formula weight	320.38	
Temperature	223(2) K	
Wavelength	0.71073 Å	
Crystal system	Monoclinic	
Space group	$P2_1/c$	
Unit cell dimensions	a = 15.123(5) Å	a= 90°.
	b = 5.9695(15) Å	b= 110.939(11)°.
	c = 16.931(6) Å	g = 90°.
Volume	1427.5(8) Å^3	
Z	4	
Density (calculated)	1.491 Mg/m^3	
Absorption coefficient	0.385 mm^{-1}	
F(000)	664	
Crystal size	0.227 x 0.079 x 0.018 mm^3	
Theta range for data collection	2.576 to 26.304°.	
Index ranges	-18<=h<=18, -7<=k<=7, -20<=l<=20	
Reflections collected	14202	
Independent reflections	2830 [R(int) = 0.0812]	
Completeness to theta = 25.242°	99.4 %	
Absorption correction	Nulti-scan	
Max. and min. transmission	0.7453 and 0.6373	
Refinement method	Full-matrix least-squares on F^2	
Data / restraints / parameters	2830 / 0 / 190	
Goodness-of-fit on F^2	1.039	
Final R indices [I>2sigma(I)]	R1 = 0.0506, wR2 = 0.1073	
R indices (all data)	R1 = 0.0885, wR2 = 0.1251	
Extinction coefficient	n/a	
Largest diff. peak and hole	0.262 and -0.505 e.Å^{-3}	

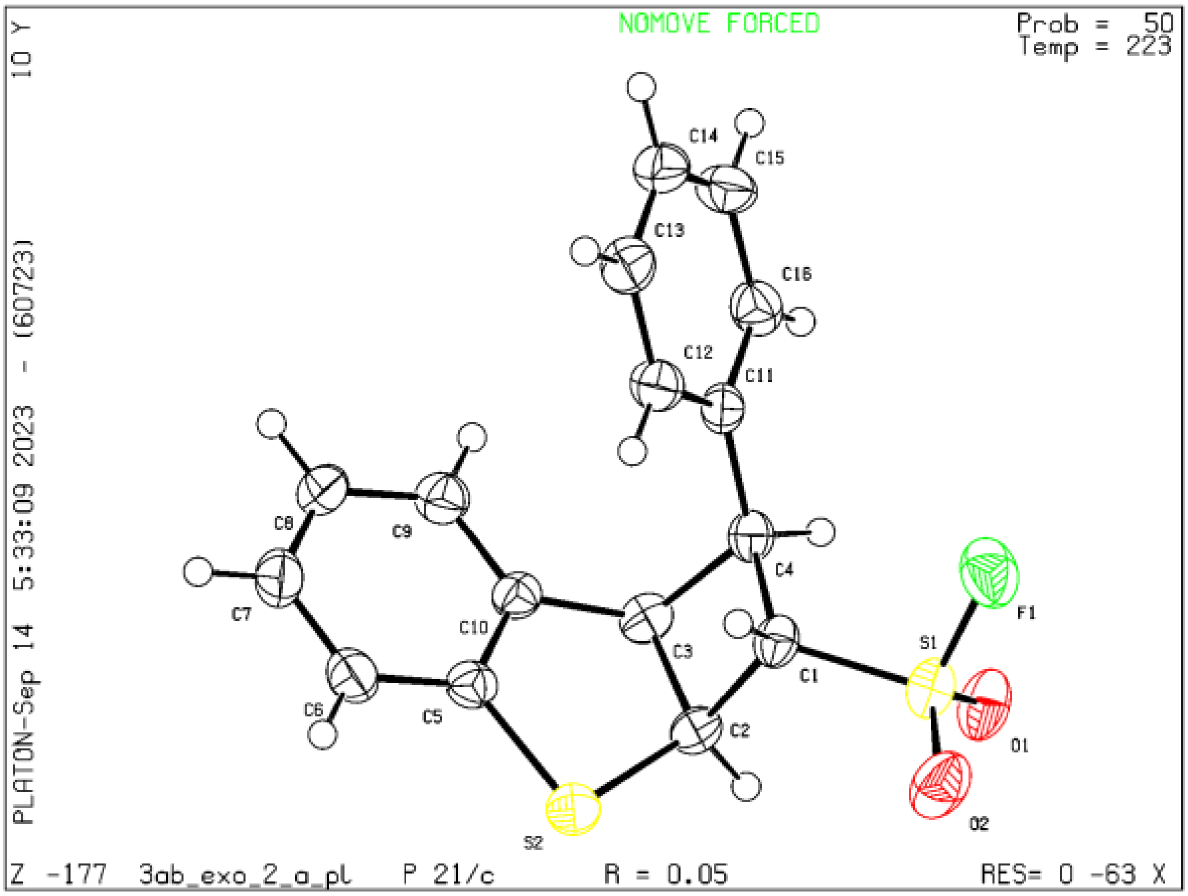

Supplementary Figure 68 ORTEP structure of **3ab**-*exo*.

X-ray crystal structure of 3ac-*exo* (CCDC-2295180)

Crystal data and structure refinement for **3ac**-*exo*.

Identification code	3ac_exo_a	
Empirical formula	C21 H22 F N O4 S	
Formula weight	403.45	
Temperature	223(2) K	
Wavelength	0.71073 Å	
Crystal system	Monoclinic	
Space group	$P2_1/n$	
Unit cell dimensions	a = 10.4641(18) Å	a= 90°.
	b = 10.5500(19) Å	b= 90.467(6)°.
	c = 18.306(3) Å	g = 90°.
Volume	2020.9(6) $Å^3$	
Z	4	
Density (calculated)	1.326 Mg/m^3	
Absorption coefficient	0.196 mm^{-1}	
F(000)	848	
Crystal size	0.158 x 0.100 x 0.085 mm^3	
Theta range for data collection	2.225 to 28.318°.	
Index ranges	-13<=h<=9, -14<=k<=14, -24<=l<=24	
Reflections collected	34500	
Independent reflections	5010 [R(int) = 0.0563]	
Completeness to theta = 25.242°	99.9 %	
Absorption correction	Semi-empirical from equivalents	
Max. and min. transmission	0.7457 and 0.7075	
Refinement method	Full-matrix least-squares on F^2	
Data / restraints / parameters	5010 / 0 / 257	
Goodness-of-fit on F^2	1.043	
Final R indices [I>2sigma(I)]	R1 = 0.0439, wR2 = 0.0996	
R indices (all data)	R1 = 0.0878, wR2 = 0.1240	
Extinction coefficient	n/a	
Largest diff. peak and hole	0.192 and -0.327 e.$Å^{-3}$	

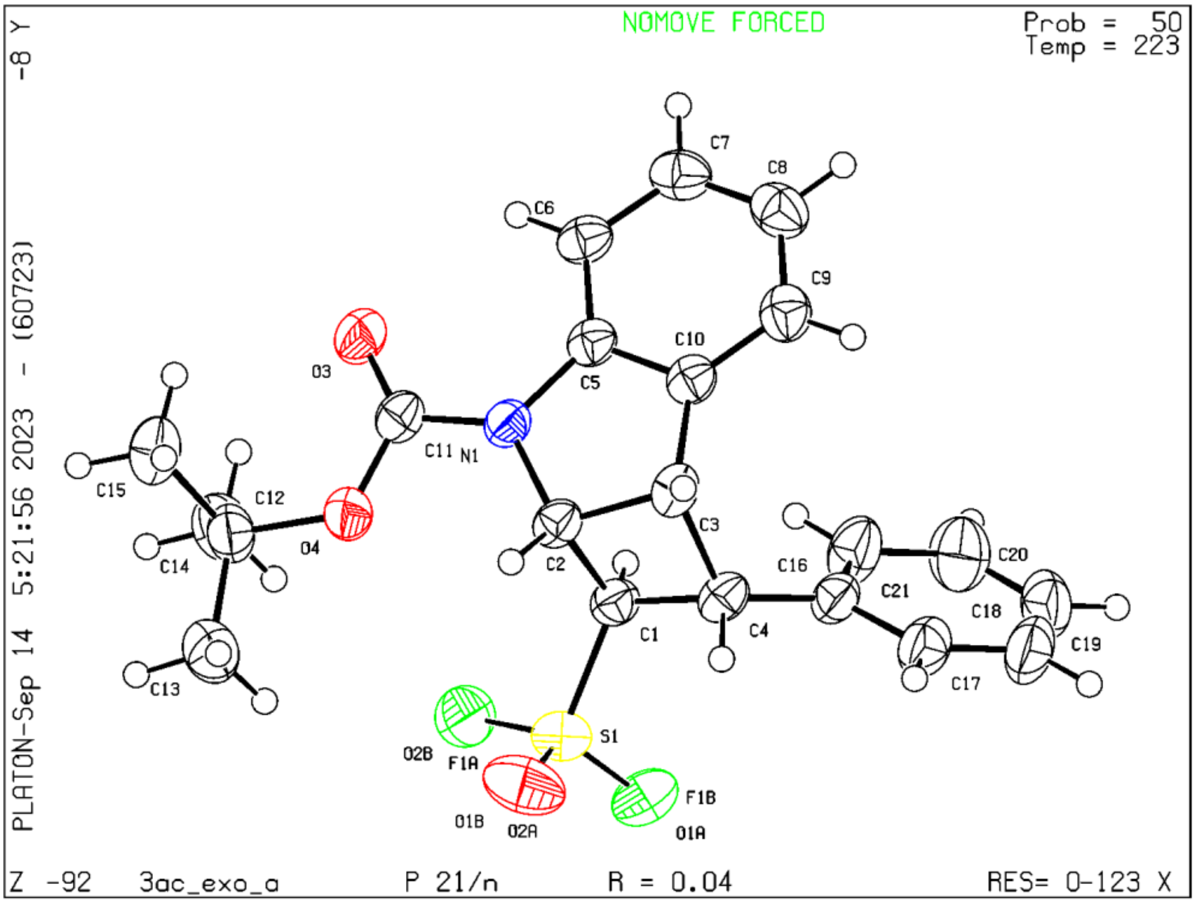

Supplementary Figure 69 ORTEP structure of **3ac**-*exo*.

X-ray crystal structure of 3ae-*endo* (CCDC-2295183)

Crystal data and structure refinement for **3ae**-*endo*.

Identification code	3ae_endo_a	
Empirical formula	C17 H15 F O2 S2	
Formula weight	334.41	
Temperature	223(2) K	
Wavelength	0.71073 Å	
Crystal system	Orthorhombic	
Space group	$P2_12_12_1$	
Unit cell dimensions	a = 9.1428(19) Å	a= 90°.
	b = 10.136(3) Å	b= 90°.
	c = 16.533(3) Å	g = 90°.
Volume	1532.2(6) $Å^3$	
Z	4	
Density (calculated)	1.450 Mg/m^3	
Absorption coefficient	0.362 mm^{-1}	
F(000)	696	
Crystal size	0.223 x 0.190 x 0.095 mm^3	
Theta range for data collection	2.357 to 28.376°.	
Index ranges	-12<=h<=10, -13<=k<=8, -22<=l<=22	
Reflections collected	11799	
Independent reflections	3787 [R(int) = 0.0447]	
Completeness to theta = 25.242°	99.8 %	
Absorption correction	Semi-empirical from equivalents	
Max. and min. transmission	0.7457 and 0.6609	
Refinement method	Full-matrix least-squares on F^2	
Data / restraints / parameters	3787 / 0 / 200	
Goodness-of-fit on F^2	1.051	
Final R indices [I>2sigma(I)]	R1 = 0.0391, wR2 = 0.0794	
R indices (all data)	R1 = 0.0537, wR2 = 0.0871	
Absolute structure parameter	0.11(5)	
Extinction coefficient	n/a	
Largest diff. peak and hole	0.199 and -0.275 e.$Å^{-3}$	

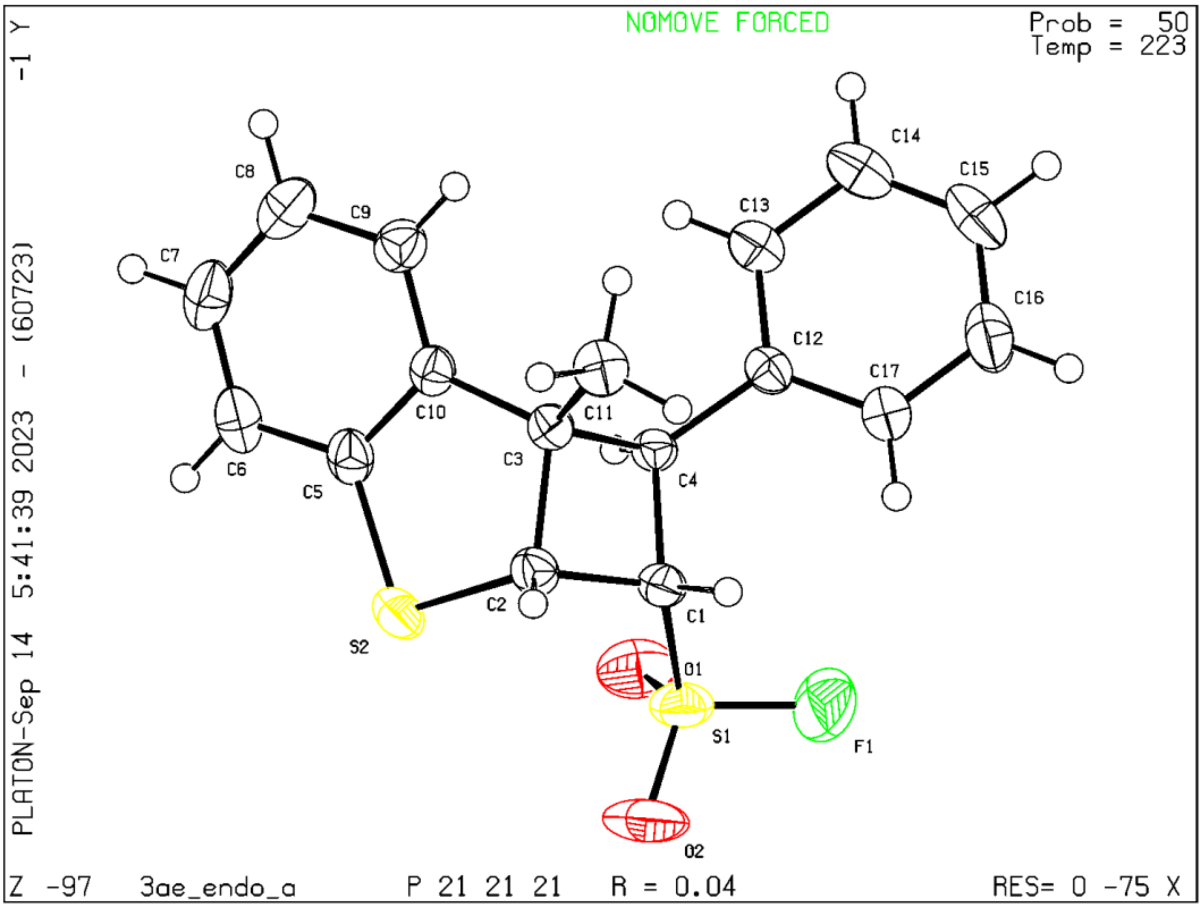

Supplementary Figure 70 ORTEP structure of **3ae**-*endo*.

Gram-scale procedure for the desired [2+2] cycloadduct

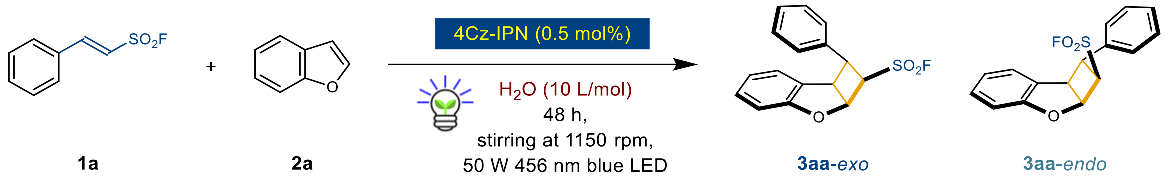

1a (1.00 g, 1.0 eq.), **2a** (5.0 eq.), and 4Cz-IPN (0.5 mol%) were added in a round-bottom flask. Afterwards, H_2O (deionized, 10 L/mol) was added in the reaction mixture without further degassing, and then stirred vigorously (rpm > 1000) under irradiation with a 50 W 456 nm blue LED for 48 h. When the reaction was completed, the crude mixture was transferred to a separation funnel, washed with EtOAc (50 mL) and water (50 mL). Thereafter, the mixture was extracted with EtOAc (50 mL × 3) and brine (50 mL). Subsequently, the organic layer was dried over anhydrous Na_2SO_4 and concentrated under reduced pressure. The resulting mixture was purified by column chromatography (eluent condition of **3aa**-*exo*; hexanes: Et_2O 100:0 to 50:1, eluent condition of **3aa**-*endo*; hexanes: Et_2O 50:0 to 20:1) to afford the desired [2+2] cycloadducts **3aa**-*exo* (1.21 g, 74% yield) and **3aa**-*endo* (0.34 g, 21% yield), respectively.

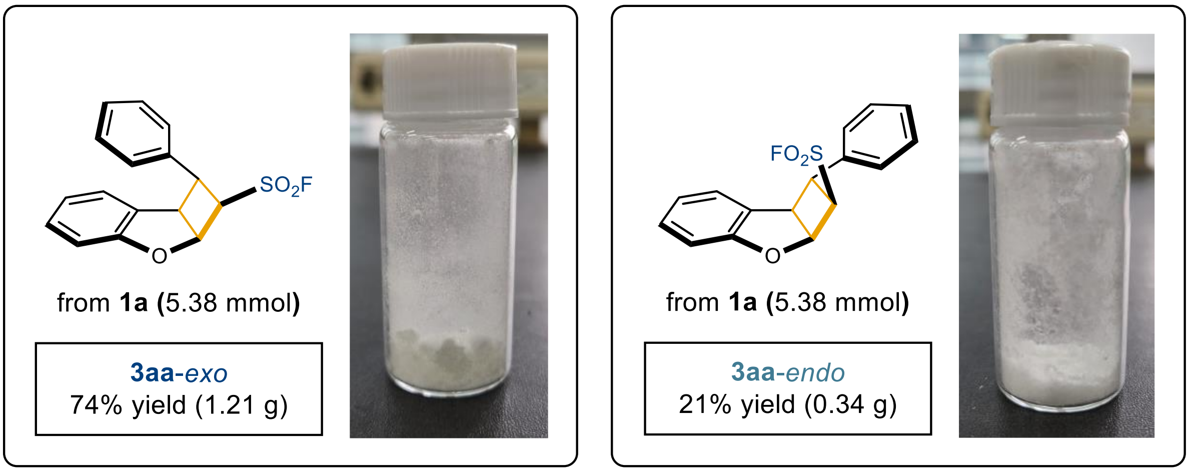

Synthetic Applications

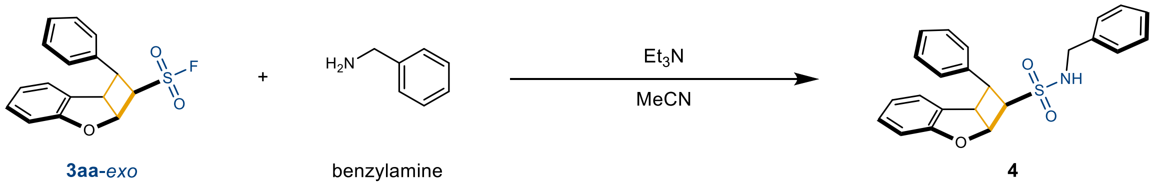

General procedure for SuFEx click reaction between 3aa-*exo* and benzylamine

The reaction was following the literature procedure[8] with modification: **3aa**-*exo* (0.2 mmol, 1.0 eq.) was added to anhydrous MeCN (0.5 mL) filled in an oven-dried vial. Benzylamine (0.4 mmol, 2.0 eq.) and Et_3N (0.4 mmol, 2.0 eq.) were added to the solution under an argon atmosphere, and the reaction mixture was then stirred at 60 °C for 24 h. After checking the reaction was completed by monitoring TLC, the crude mixture was quenched with brine and extracted with EtOAc (three times). Subsequently, the organic layer was dried over anhydrous Na_2SO_4 and concentrated under reduced pressure. The resulting residue was purified by column chromatography to afford the sulfonamide SuFEx product **4** in 61% yield.

N-benzyl-1-phenyl-1,2,2a,7b-tetrahydrocyclobuta[b]benzofuran-2-sulfonamide (4)

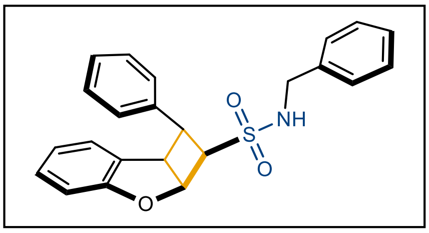

New compound, white solid, 94.9 mg, 61% yield.
Purification conditions for column chromatography: hexanes:EtOAc 50:1 to 8:1
Rf = 0.42 (acetone:hexanes = 1:4 v/v).
mp: 120 °C.
^{1}H NMR (500 MHz, $CDCl_3$): δ 7.27 – 7.25 (m, 6H), 7.18 – 7.14 (m, 1H), 7.12 – 7.11 (m, 2H), 6.97 – 6.95 (m, 2H), 6.92 (d, *J* = 8.1 Hz, 1H), 6.73 – 6.70 (m, 1H), 6.49 (d, *J* = 7.5 Hz, 1H), 5.53 (dd, *J* = 7.1, 4.5 Hz, 1H), 4.44 – 4.38 (m, 2H), 4.31 – 4.27 (m, 1H), 4.24 – 4.16 (m, 2H), 4.13 – 4.09 (m, 1H).
^{13}C NMR (126 MHz, $CDCl_3$): δ 160.6, 136.3, 136.1, 129.4, 128.9, 128.6, 128.25, 128.23, 128.1, 127.9, 127.7, 125.0, 121.5, 111.3, 78.0, 65.8, 47.51, 47.47, 42.7.
HR-MS: *m/z* calcd. [$C_{23}H_{21}NO_3S$ + Na]: 414.1140; found (TOF MS ES+): 414.1134.

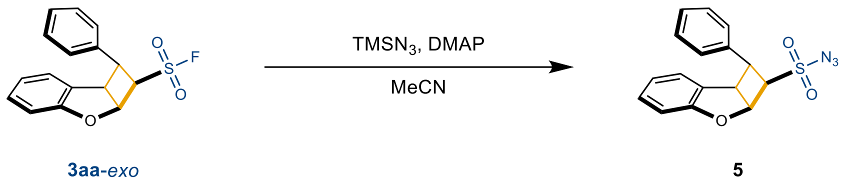

General procedure for SuFEx-CuAAC double click reactions
The reaction was following the literature procedure[8] with modification: **3aa**-*exo* (0.2 mmol, 1.0 eq.) and DMAP (0.3 mmol, 1.5 eq.) were added to anhydrous MeCN (0.4 mL) filled in an oven-dried vial. $TMSN_3$ (trimethylsilyl azide; 0.3 mmol, 1.5 eq.) was added to the solution under an argon atmosphere, and the reaction mixture was then stirred at 60 °C for overnight. After checking the reaction was completed by monitoring TLC, the crude mixture was quenched with brine and extracted with EtOAc (three times). Subsequently, the organic layer was dried over anhydrous Na_2SO_4 and concentrated under reduced pressure. The resulting residue was purified by column chromatography to afford the sulfonyl azide-SuFEx product **5** in 81% yield.

1-phenyl-1,2,2a,7b-tetrahydrocyclobuta[b]benzofuran-2-sulfonyl azide (5)

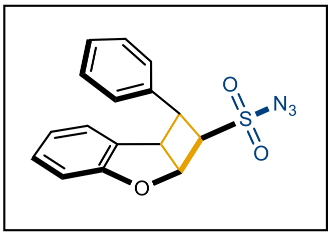

New compound, cloudy white solid, 53.2 mg, 81% yield.
Purification conditions for column chromatography: hexanes:Et_2O 50:1 to 20:1
Rf = 0.50 (acetone:hexanes = 1:6 v/v)
mp: 79 °C.
^{1}H NMR (500 MHz, $CDCl_3$): δ 7.31 – 7.26 (m, 3H), 7.20 – 7.17 (m, 1H), 7.02 – 7.00 (m, 2H), 6.94 (d, *J* = 8.1 Hz, 1H), 6.76 – 6.72 (m, 1H), 6.52 (d, *J* = 7.5 Hz, 1H), 5.59 (dd, *J* = 7.1, 4.6 Hz, 1H), 4.55 – 4.49 (m, 2H), 4.40 – 4.37 (m, 1H).
^{13}C NMR (126 MHz, $CDCl_3$): δ 160.3, 134.8, 132.1, 129.8, 128.8, 128.0, 127.5, 124.2, 121.9, 111.5, 77.4, 68.5, 48.1, 42.6.
HR-MS: *m/z* calcd. [$C_{21}H_{22}FNO_4S$]$^+$: 327.0678; found (EI+): 327.0675.

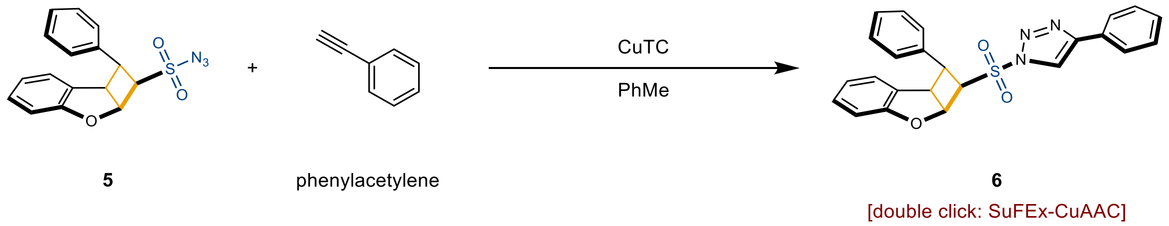

The reaction was following the literature procedure[8] with modification: The azide-SuFEx product **5** (0.2 mmol, 1.0 eq.) and CuTC (copper(I) thiophene-2-carboxylate; 10 mol%) were added to anhydrous PhMe (4.0 mL) filled in an oven-dried vial. Phenylacetylene (0.22 mmol, 1.1 eq.) was added under an argon atmosphere, and the

reaction mixture was stirred at room temperature for 24 h. After checking the reaction was completed by monitoring TLC, the reaction mixture was quenched with brine and extracted with EtOAc (three tmies). Subsequently, the organic layer was dried over anhydrous Na_2SO_4, and concentrated under reduced pressure. The resulting residue was purified by column chromatography to afford the SuFEx-CuAAC product **6** in 90% yield.

4-phenyl-1-((1-phenyl-1,2,2a,7b-tetrahydrocyclobuta[*b*]benzofuran-2-yl)sulfonyl)-*1H*-1,2,3-triazole (6)

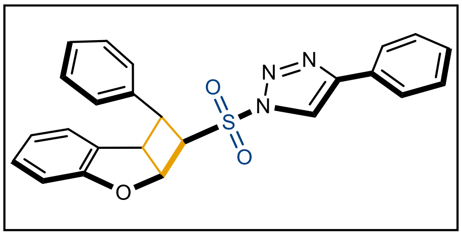

New compound, pale brown solid, 77.4 mg, 90% yield.
Purification conditions for column chromatography: hexanes:Et_2O 50:1 to 20:1
R_f = 0.52 (acetone:hexanes = 1:6 v/v)
mp: 141 °C.
^{1}H NMR (500 MHz, $CDCl_3$): δ 8.14 (s, 1H), 7.73 – 7.71 (m, 2H), 7.44 – 7.36 (m, 3H), 7.17 – 7.13 (m, 4H), 6.89 (d, J = 8.1 Hz, 1H) 6.84 – 6.82 (m, 2H), 6.72 – 6.69 (m, 1H), 6.47 (d, J = 7.4 Hz, 1H), 5.74 (dd, J = 7.1, 4.7 Hz, 1H), 4.77 – 4.74 (m, 1H), 4.53 – 4.50 (m, 1H), 4.42 – 4.39 (m, 1H).
^{13}C NMR (126 MHz, $CDCl_3$): δ 160.1, 147.8, 134.0, 129.9, 129.4, 129.1, 128.7, 128.6, 128.1, 128.0, 127.4, 126.4, 123.8, 122.0, 119.7, 111.7, 77.0, 68.0, 48.2, 42.4.
HR-MS: *m/z* calcd. $[C_{24}H_{20}N_3O_3S + H]^+$: 430.1225; found (FAB+): 430.1223.

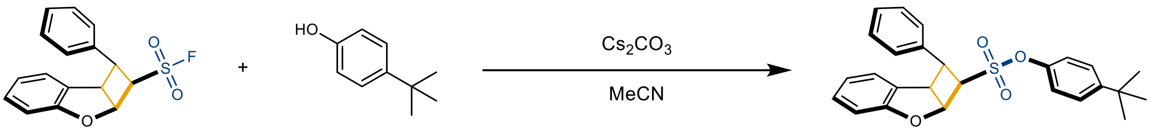

3aa-*exo* 4-*tert*-butylphenol 7

General procedure for SuFEx click reaction between 3aa-*exo* and 4-*tert*-butylphenol
The reaction was following the literature procedure[8] with modification: **3aa**-*exo* (0.2 mmol, 1.0 eq.), 4-*tert*-butylphenol (0.24 mmol, 1.2 eq.), and Cs_2CO_3 (0.4 mmol, 2.0 eq.) were added to anhydrous MeCN (1.0 mL) filled in an oven-dried vial under an argon atmosphere. The reaction mixture was then stirred at room temperature for 12 h. After checking the reaction was completed by monitoring TLC, the crude mixture was quenched with brine and extracted with EtOAc (three times). Subsequently, the organic layer was dried over anhydrous Na_2SO_4 and concentrated under reduced pressure. The resulting residue was purified by column chromatography to afford the sulfonic ester-SuFEx product **7** in 86% yield.

4-(*tert*-butyl)phenyl-1-phenyl-1,2,2a,7b-tetrahydrocyclobuta[*b*]benzofuran-2-sulfonate (7)

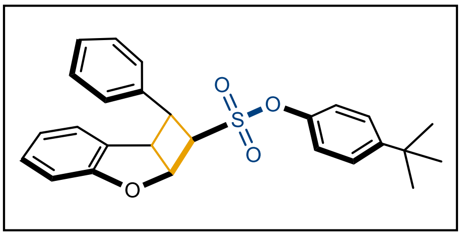

New compound, colorless oil, 74.9 mg, 86% yield.
Purification conditions for column chromatography: hexanes:Et_2O 50:1 to 20:1
R_f = 0.51 (acetone:hexanes = 1:6 v/v)
^{1}H NMR (500 MHz, $CDCl_3$): δ 7.32 – 7.29 (m, 2H), 7.25 – 7.23 (m, 3H), 7.19 – 7.15 (m, 1H), 7.05 – 7.02 (m, 2H), 6.98 – 6.94 (m, 3H), 6.74 – 6.71 (m, 1H), 6.54 (d, J = 7.4 Hz, 1H), 5.67 – 5.64 (m, 1H), 4.54 – 4.44 (m, 3H), 1.29 (s, 9H).
^{13}C NMR (126 MHz, $CDCl_3$): δ 160.5, 150.5, 146.9, 135.5, 129.6, 128.5, 128.1, 127.83, 127.76, 126.9, 124.7, 122.8, 121.7, 111.3, 77.9, 63.6, 48.0, 42.7, 34.7, 31.5.
HR-MS: *m/z* calcd. $[C_{26}H_{26}O_4S]^+$: 434.1552; found (EI+): 434.1545.

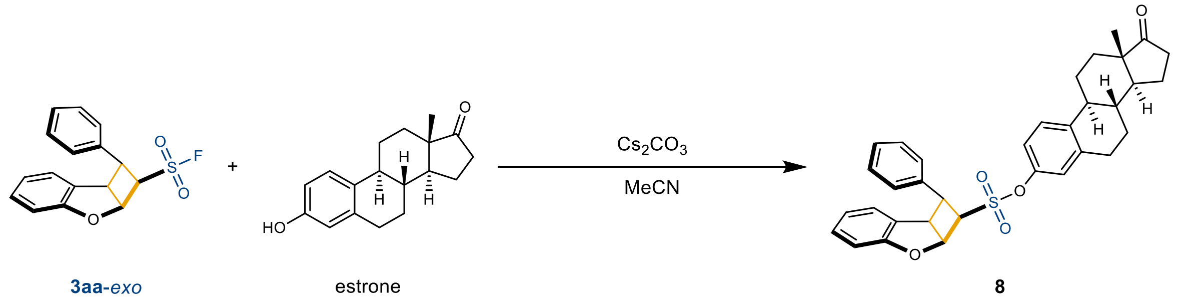

General procedure for SuFEx click reaction between 3aa-*exo* and estrone

The reaction was following the literature procedure[8] with modification: **3aa**-*exo* (0.2 mmol, 1.0 eq.), estrone (0.24 mmol, 1.2 eq.), and Cs_2CO_3 (0.4 mmol, 2.0 eq.) were added to anhydrous MeCN (1.0 mL) filled in an oven-dried vial under an argon atmosphere. The reaction mixture was then stirred at room temperature for 24 h. Thereafter, Cs_2CO_3 (0.1 mmol, 0.5 eq.) was further added and stirred for an additional 24 h. The crude mixture was quenched with brine and extracted with EtOAc (three times) after checking the reaction was completed by monitoring TLC. Subsequently, the organic layer was dried over anhydrous Na_2SO_4 and concentrated under reduced pressure. The resulting residue was purified by column chromatography to afford the estrone-SuFEx product **8** in 64% yield.

(*8R*,*9S*,*13S*,*14S*)-13-methyl-17-oxo-7,8,9,11,12,13,14,15,16,17-decahydro-*6H*-cyclopenta[*a*]phenanthren-2-yl-1-phenyl-1,2,2a,7b-tetrahydrocyclobuta[*b*]benzofuran-2-sulfonate (8)

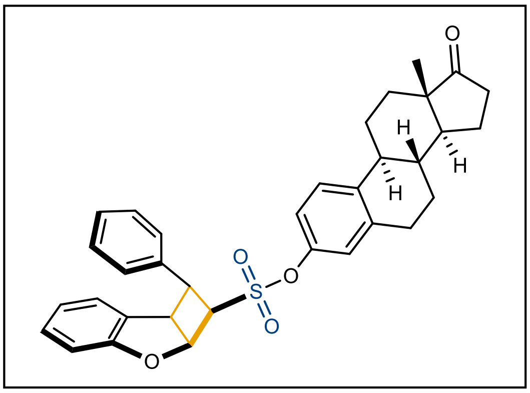

New compound, white solid, 142.3 mg, 64% yield.
Purification conditions for column chromatography: hexanes:EtOAc 20:1 to 4:1
R$_f$ = 0.38 (acetone:hexanes = 1:2 v/v)
mp: 197 °C.
^{1}H NMR (500 MHz, $CDCl_3$): δ 7.26 – 7.15 (m, 5H), 6.98 – 6.91 (m, 4H), 6.83 – 6.79 (m, 1H), 6.74 – 6.71 (m, 1H), 6.55 – 6.54 (m, 1H), 5.66 – 5.64 (m, 1H), 4.54 – 4.42 (m, 3H), 2.86 – 2.78 (m, 2H), 2.53 – 2.48 (m, 1H), 2.39 – 2.35 (m, 1H), 2.27 – 2.22 (m, 1H), 2.18 – 2.11 (m, 1H), 2.08 – 1.93 (m, 3H), 1.66 – 1.39 (m, 6H), 0.90 (s, 3H).
^{13}C NMR (126 MHz, $CDCl_3$): δ 220.8, 160.4, 147.1, 139.1, 138.7 (d, *J* = 2.9 Hz), 135.5 (d, *J* = 1.4 Hz), 129.6, 128.5, 128.2, 127.84 (d, *J* = 3.8 Hz), 127.75 (d, *J* = 1.4 Hz), 126.9 (d, *J* = 0.7 Hz), 124.7, 122.3 (d, *J* = 3.3 Hz), 121.7, 119.4 (d, *J* = 2.9 Hz), 111.2, 77.8, 63.4 (d, *J* = 10.1 Hz), 50.5, 48.02, 47.95, 44.2 (d, *J* = 1.5 Hz), 42.7 (d, *J* = 5.7 Hz), 38.0, 36.0, 31.6, 29.4 (d, *J* = 4.1 Hz), 26.3, 25.8, 21.7, 14.0.
HR-MS: *m/z* calcd. $[C_{34}H_{34}O_5S + H]^+$: 555.2205; found (FAB+): 555.2201.

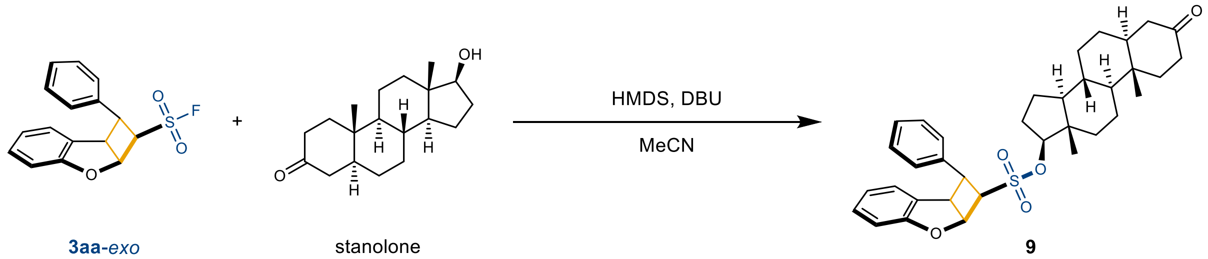

General procedure for SuFEx click reaction between 3aa-*exo* and stanolone

The reaction was following the literature procedure[9] with modification: HMDS (hexamethyldisilane; 0.2 mmol, 1.0 eq.) and DBU (1,8-diazabicyclo[5.4.0]undec-7-ene; 0.2 mmol, 1.0 eq.) were added to a solution of **3aa**-*exo* (0.2 mmol, 1.0 eq.) and stanolone (0.24 mmol, 1.2 eq.) in anhydrous MeCN (0.5 mL) under an argon atmosphere. The reaction mixture was then stirred at 60 °C for 24 h. When the reaction was complete, the solvent was removed. The obtained crude product was purified by column chromatography to afford the stanolone-SuFEx product **9** in 31% yield.

(*5S*,*8R*,*9S*,*10S*,*13S*,*14S*,*17S*)-10,13-dimethyl-3-oxohexadecahydro-*1H*-cyclopenta[*a*]phenanthren-17-yl-1-phenyl-1,2,2a,7b-tetrahydrocyclobuta[*b*]benzofuran-2-sulfonate (9)

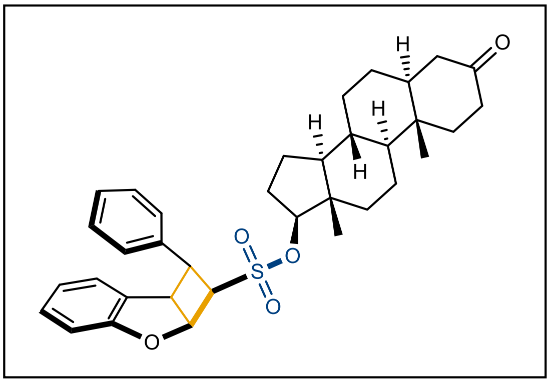

New compound, white solid, 35.9 mg, 31% yield.
Purification conditions for column chromatography: hexanes:EtOAc 10:1 to 5:1
R_f = 0.42 (acetone:hexanes = 1:2 v/v)
mp: 220 °C.
^{1}H NMR (700 MHz, $CDCl_3$): δ 7.26 – 7.21 (m, 3H), 7.16 – 7.14 (m, 1H), 7.00 – 6.99 (m, 2H), 6.90 (dd, *J* = 7.9, 4.6 Hz, 1H), 6.71 (td, *J* = 7.3, 1.5 Hz, 1H), 6.53 (dd, *J* = 7.2, 4.1 Hz, 1H), 5.55 – 5.52 (m, 1H), 4.47 – 4.43 (m, 2H), 4.35 – 4.31 (m, 1H), 4.29 – 4.27 (m, 1H), 2.40 – 2.35 (m, 1H), 2.31 – 2.29 (m, 1H), 2.25 (t, *J* = 14.4 Hz, 1H), 2.10 – 1.99 (m, 3H), 1.81 – 1.28 (m, 14H), 1.10 – 1.02 (m, 1H), 0.99 (s, 3H), 0.96 – 0.92 (m, 1H), 0.71 (s, 3H).
^{13}C NMR (175 MHz, $CDCl_3$): δ 211.8, 160.5 (d, *J* = 1.0 Hz),135.8, 129.5, 128.44 (d, *J* = 1.6 Hz), 128.1 (d, *J* = 3.2 Hz), 127.8 (d, *J* = 10.7 Hz), 127.6 (d, *J* = 5.3 Hz), 124.9 (d, *J* = 2.7 Hz), 121.6, 111.2 (d, *J* = 2.9 Hz), 90.1 (d, *J* = 16.5 Hz), 77.9, 64.1, 53.8, 49.9, 47.8 (d, *J* = 1.9 Hz), 46.7, 44.8, 43.3 (d, *J* = 7.7 Hz), 42.8 (d, *J* = 11.9 Hz), 38.6 (d, *J* = 1.2 Hz), 38.2, 36.2 (d, *J* = 7.4 Hz), 35.8, 35.3 (d, *J* = 2.3 Hz), 31.2 (d, *J* = 1.3 Hz), 28.8, 28.0 (d, *J* = 1.7 Hz), 23.5 (d, *J* = 7.4 Hz), 20.9, 11.8 (d, *J* = 6.1 Hz), 11.6.
HR-MS: *m/z* calcd. [$C_{35}H_{42}O_5S$ + Na]: 597.2651; found (TOF MS ES+): 597.2654.

Supplementary Discussion

Reaction in the presence of TEMPO as a radical scavenger

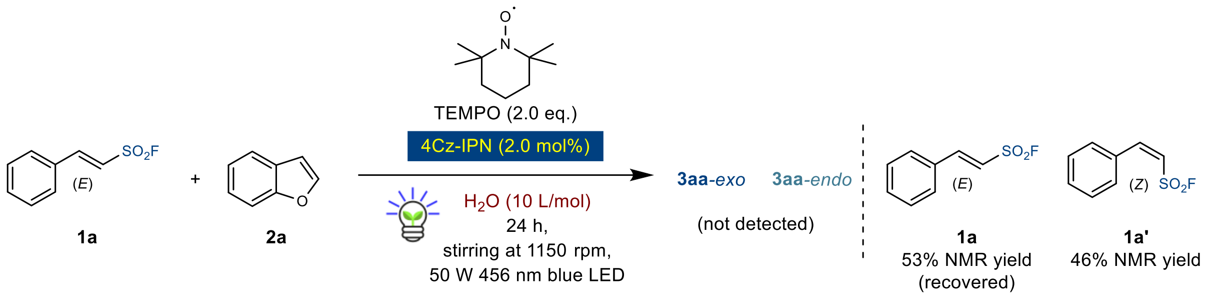

1a (0.2 mmol, 1.0 eq.), 4Cz-IPN (2.0 mol%), and TEMPO ((2,2,6,6-tetramethylpiperidin-1-yl)oxyl; 0.4 mmol, 2.0 eq.) were added in a vial without further drying equipped with multiple magnetic stirring bars. Subsequently, 2,3-benzofuran (1.0 mmol, 5.0 eq.) and H_2O (deionized, 10 L/mol, 2.0 mL) were added in the reaction mixture without further degassing, and then stirred vigorously (rpm > 1000) under irradiation with a 50 W 456 nm blue LED for 24 h. As a result, desired products were not detected on the crude ^{1}H NMR analysis. Remained **1a** (53%) was observed with isomerized **1a'** (46%). In this case, 1,4-dimethoxybenzene (0.2 mmol, 1.0 eq.) was used as a standard for ^{1}H NMR analysis.

Crude ^{19}F NMR analyses of reaction mixture

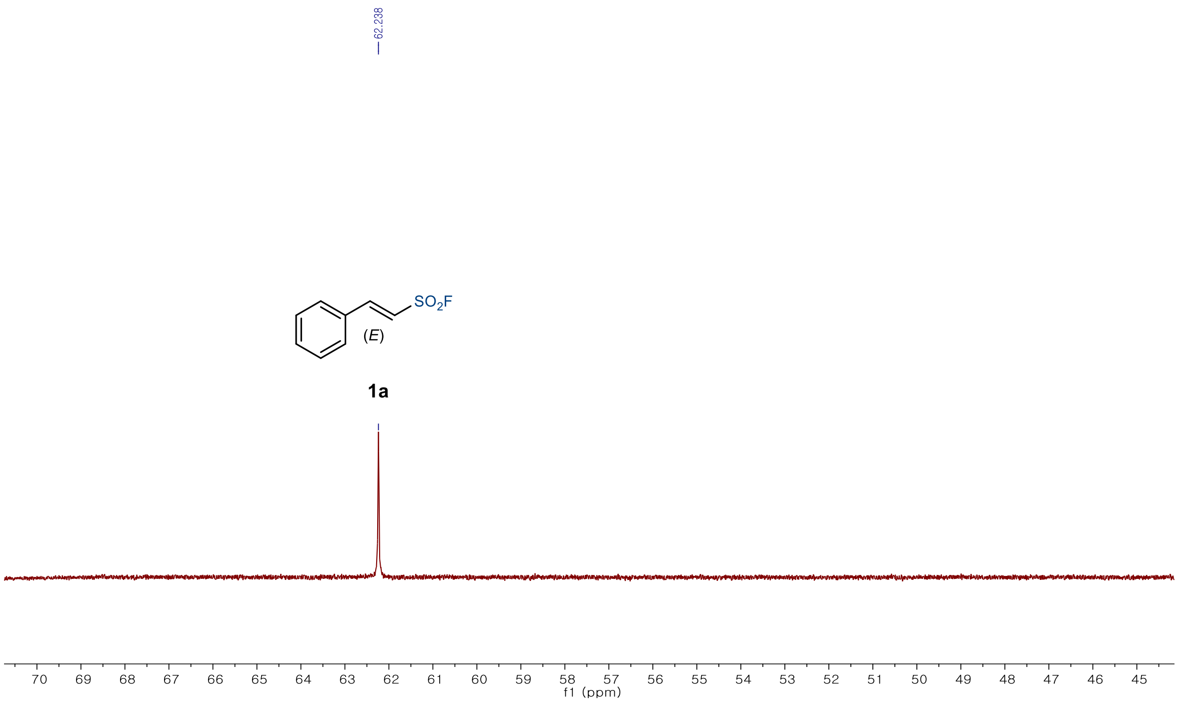

Supplementary Figure 71 ^{19}F NMR of reaction mixture before the reaction (500 MHz, DMSO-d_6, 25 °C)

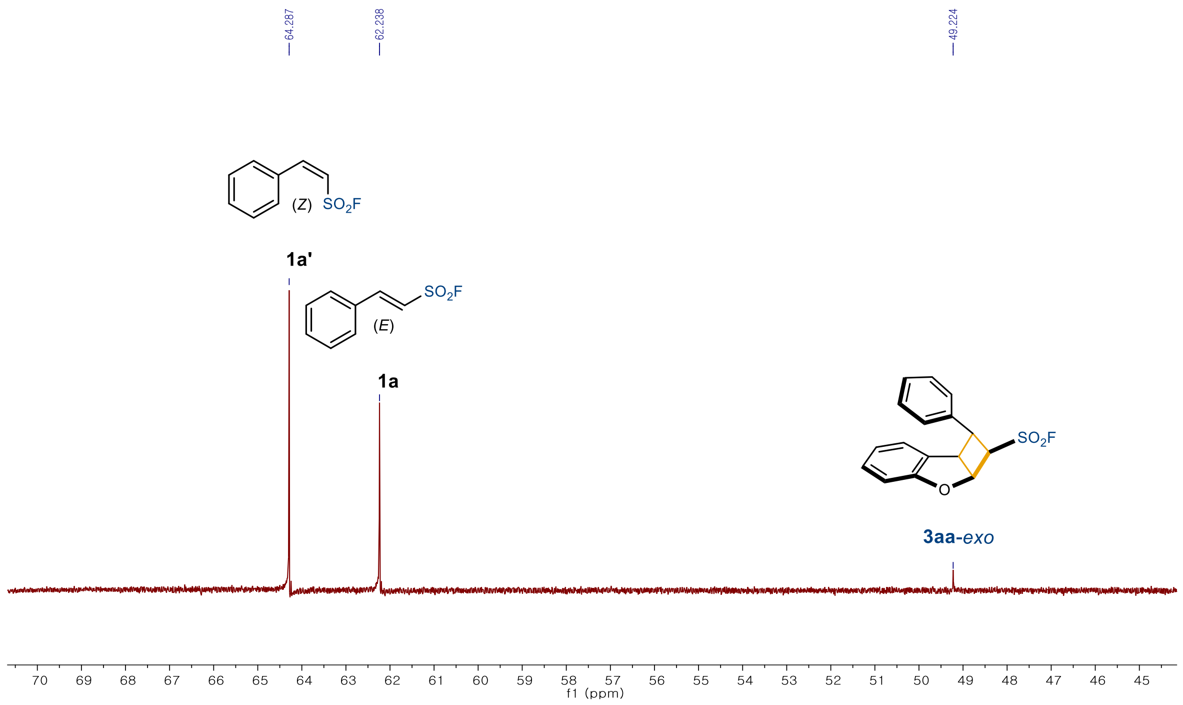

Supplementary Figure 72 ^{19}F NMR of reaction mixture after 10 min (500 MHz, DMSO-d_6, 25 °C)

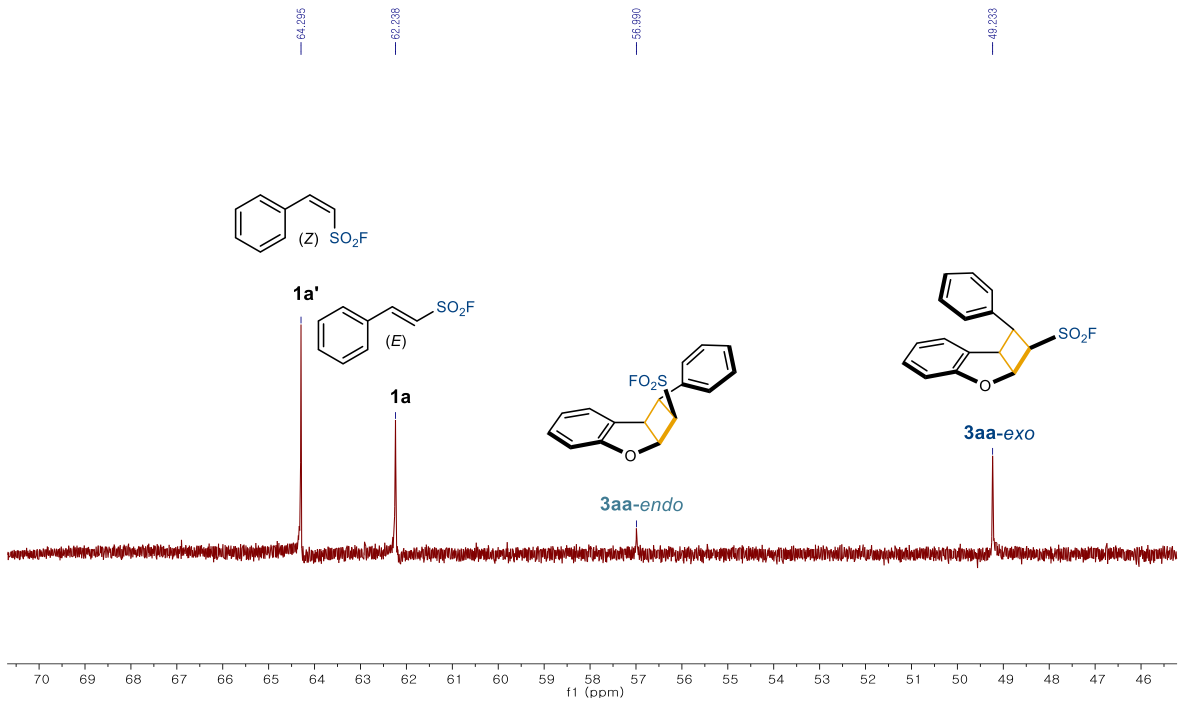

Supplementary Figure 73 ^{19}F NMR of reaction mixture after 60 min (500 MHz, DMSO-d_6, 25 °C)

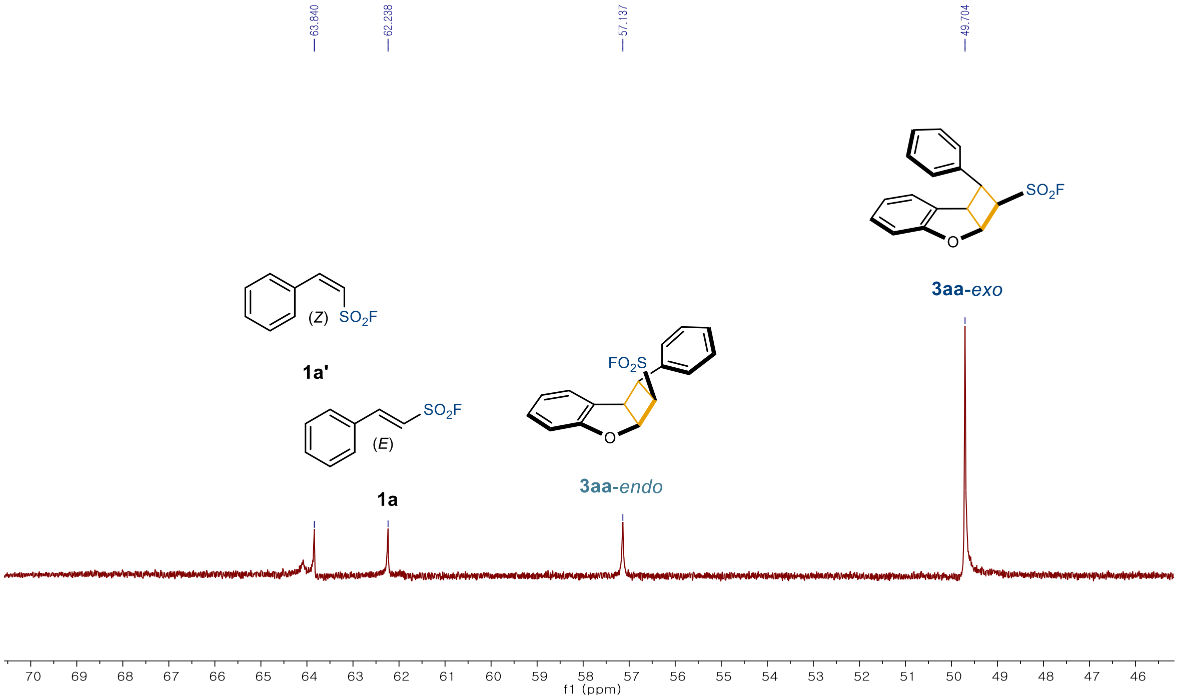

Supplementary Figure 74 ^{19}F NMR of reaction mixture after 120 min (500 MHz, DMSO-d_6, 25 °C)

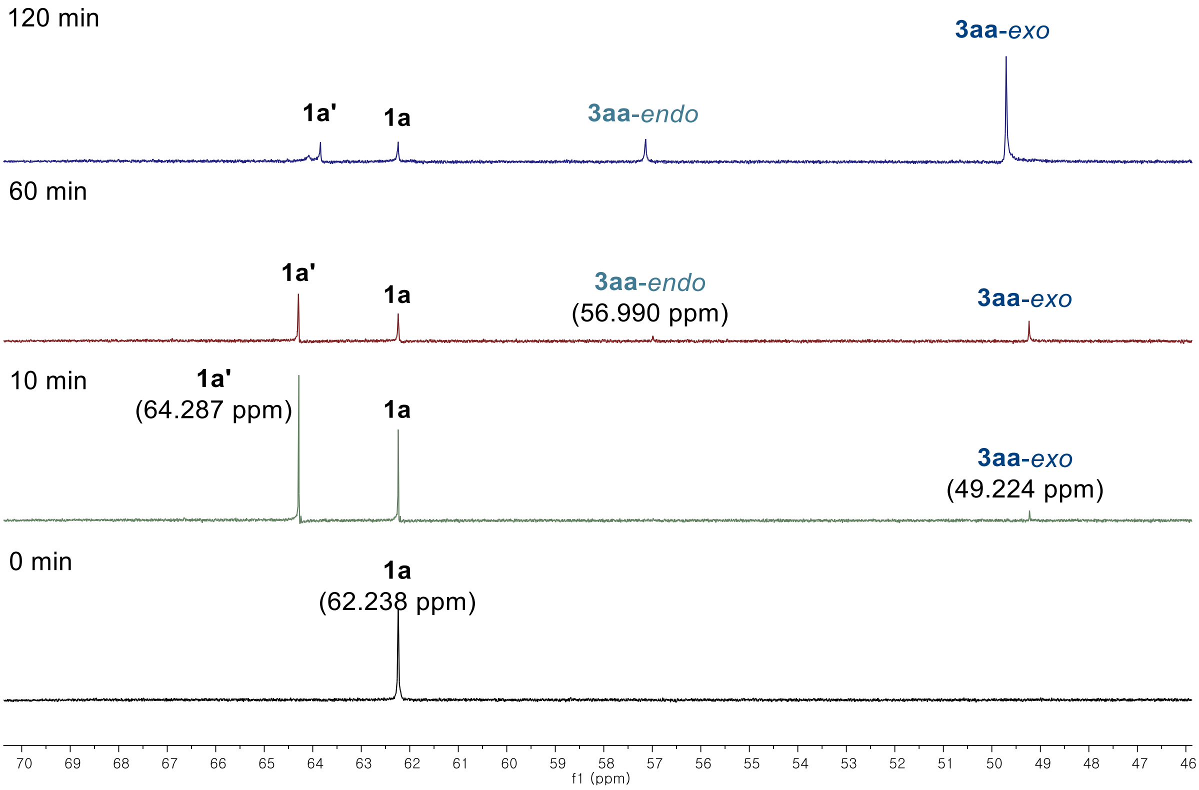

Supplementary Figure 75 Stacked ^{19}F NMR spectra (500 MHz, DMSO-d_6, 25 °C)

Comment: The E to Z isomerization process is faster than the intermolecular [2+2] cycloaddition. The product **3aa**-*exo* is generated more rapidly than the product **3aa**-*endo*.

Cyclic voltammetry

Cyclic voltammetry experiments were conducted according to the reported procedure[10]: the experiments were performed in MeCN with analyte (0.01 M) and $TBAPF_6$ (0.1 M) using a glassing carbon working electrode, an $Ag/AgNO_3$ MeCN reference electrode, and a scan rate of 100 mV/s, and potential referred to SCE at room temperature. using a platinum wire as counter electrode. In acetonitrile condition, the redox potential of **4Cz-IPN** was reported to E_{red} = −1.24 V (*vs.* SCE.) and E_{ox} = +1.49 V (*vs.* SCE), respectively[11].

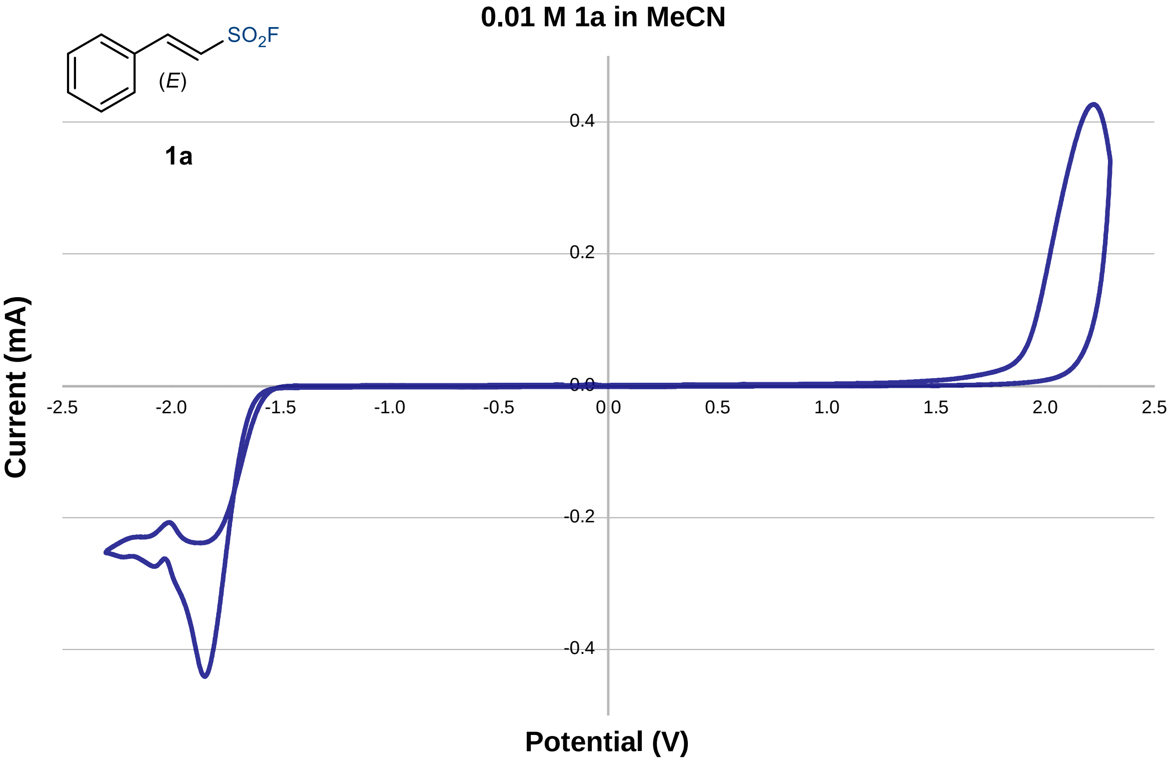

Supplementary Figure 76 Cyclic voltammogram (100 mV/s) of **1a** in acetonitrile with $TBAPF_6$ (0.1 M) as the supporting electrolyte

E_{ox} **1a** = +2.22 V *vs.* SCE
E_{red} **1a** = −1.85 V *vs.* SCE

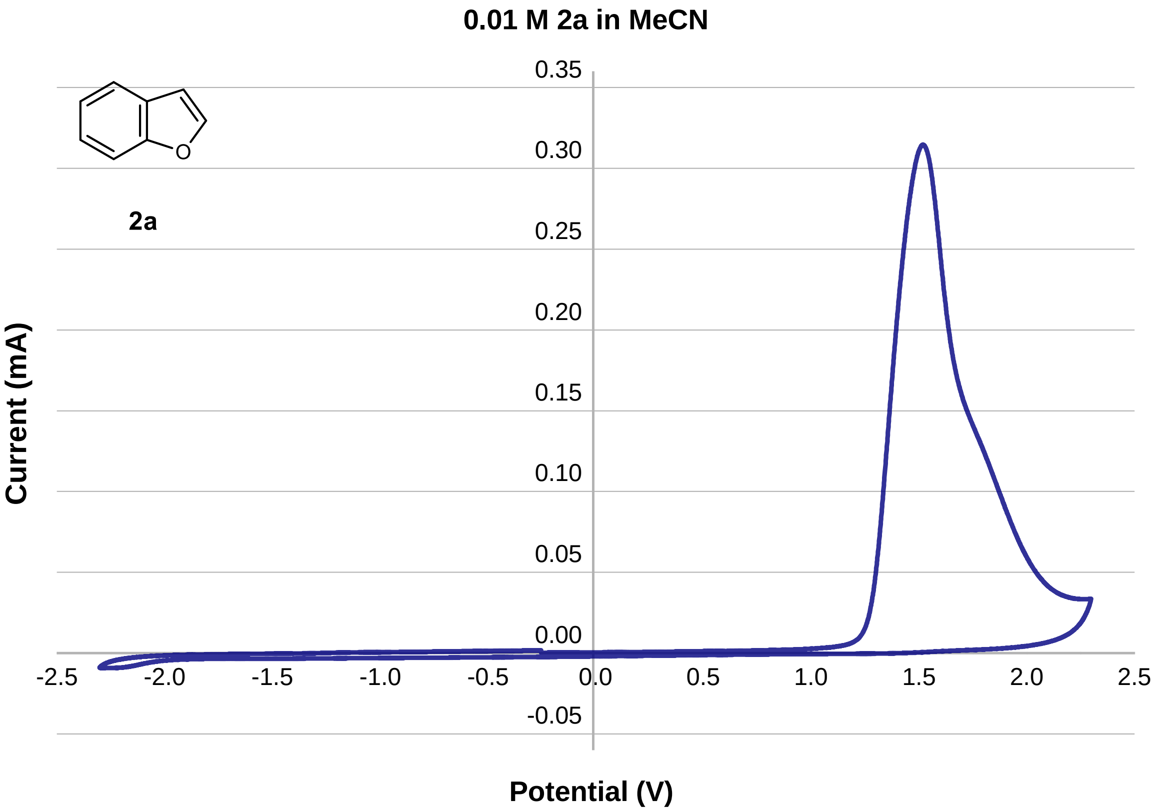

Supplementary Figure 77 Cyclic voltammogram (100 mV/s) of **2a** in acetonitrile with $TBAPF_6$ (0.1 M) as the supporting electrolyte

E_{ox} **2a** = +1.52 V *vs*. SCE
E_{red} **2a** = No reduction wave

Spectrochemical control experiments

a

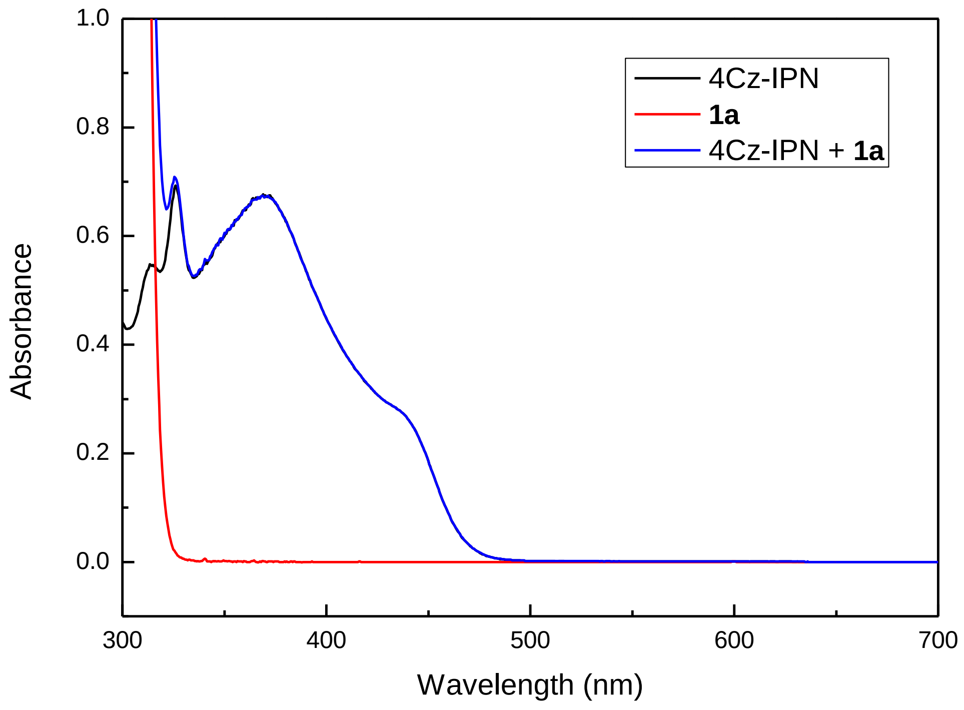

b

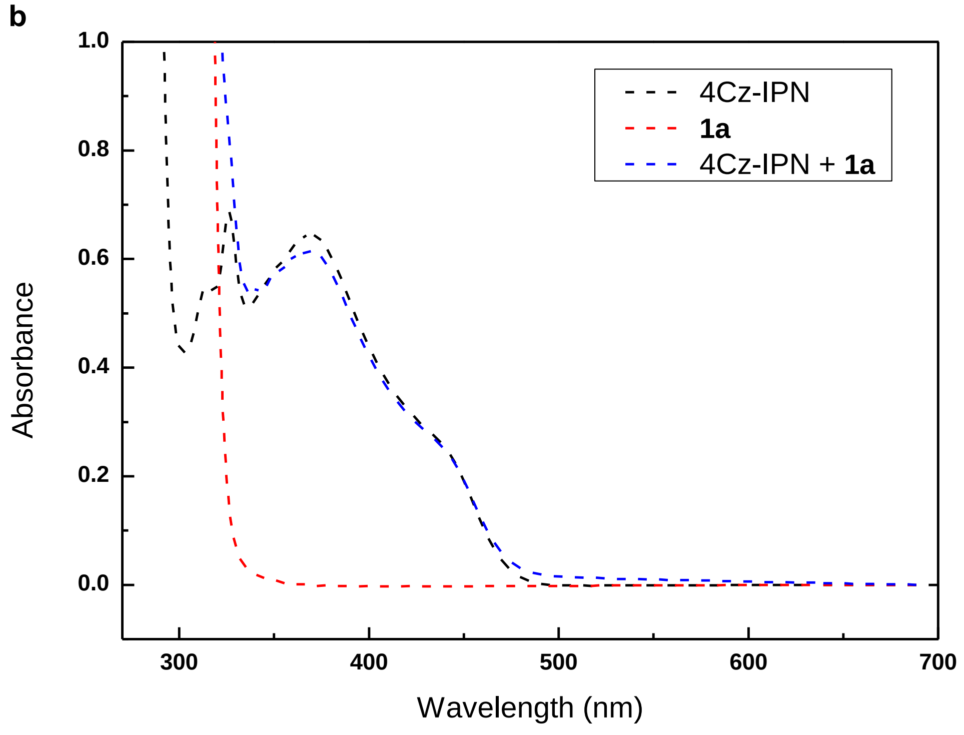

c

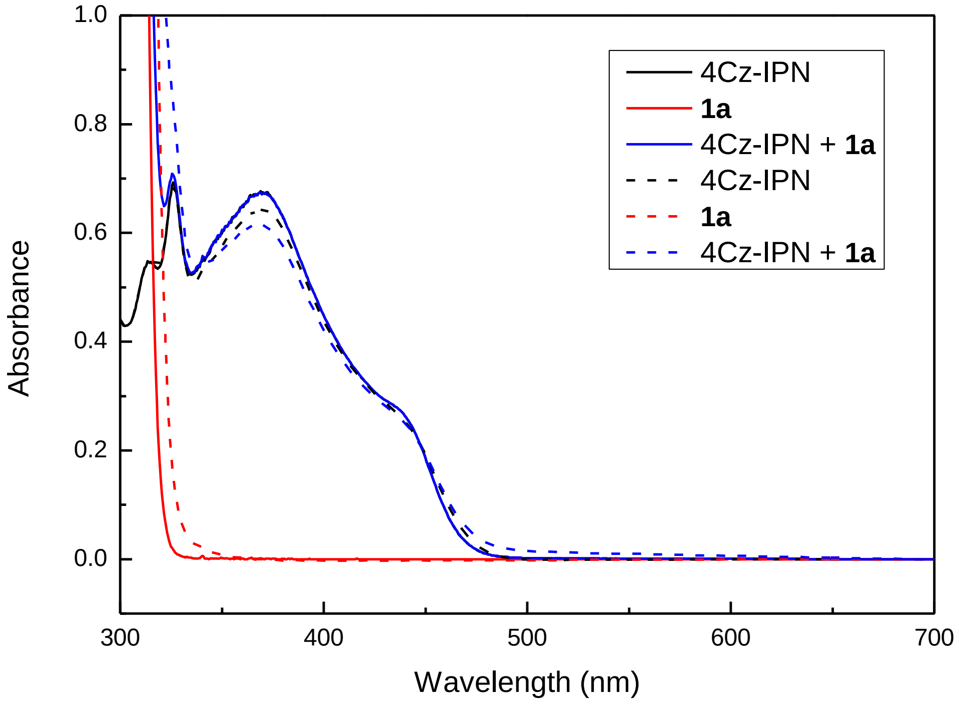

Supplementary Figure 78 UV–vis spectra

- **a.** Components of the parent reaction in THF solution; [4Cz-IPN] = 40 μM, [**1a**] = 1 mM, and [4Cz-IPN + **1a**] = 40 μM of 4Cz-IPN with 1 mM of **1a**.
- **b.** Components of the parent reaction in co-solution of THF and H_2O (1:1); [4Cz-IPN] = 40 μM, [**1a**] = 1 mM, and [4Cz-IPN + **1a**] = 40 μM of 4Cz-IPN with 1 mM of **1a**.
- **c.** Overlayed UV–vis spectra of the components of the parent reaction for THF solution (solid line) and THF 10 mL + H_2O 10 mL co-solution (dashed line).

Comment: Because of the limited solubility of the organic analytes in pure water, the initial measurement attempt was unsuccessful. Consequently, a co-solution of water/THF was employed for UV-Vis measurements. The experimental data revealed a negligible variance in absorption wavelength between the use of THF alone and the water/THF mixture, which could be attributed to experimental error.

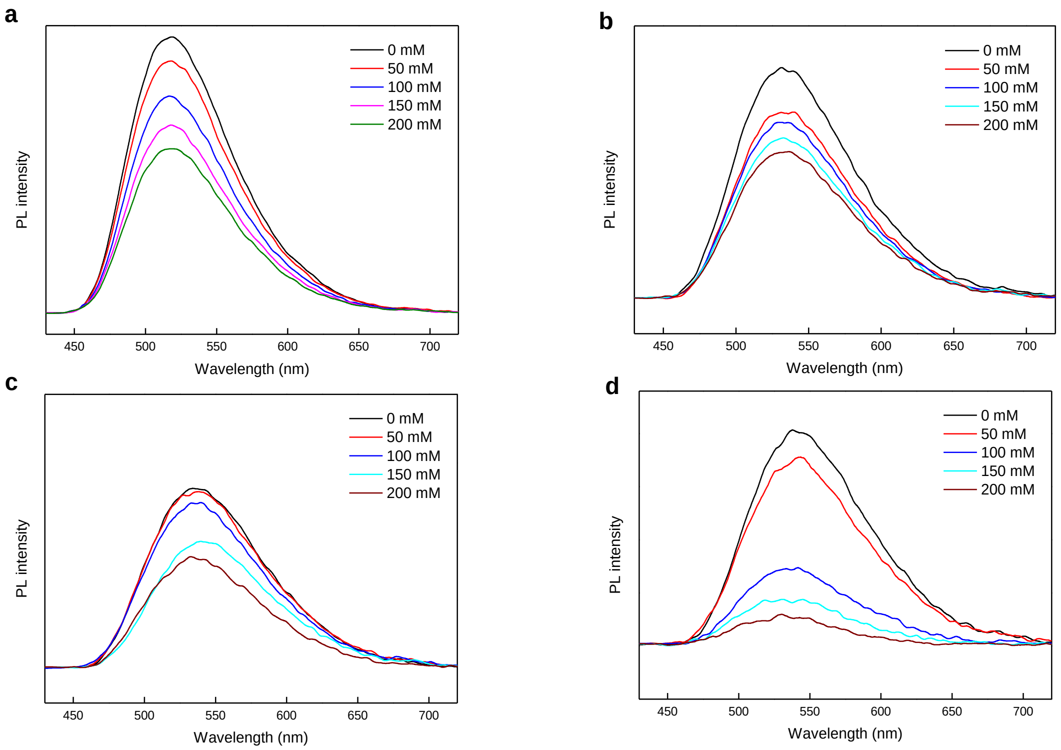

Supplementary Figure 79 PL spectra of the reaction components

[4Cz-IPN + **1a**] by **2a** in the various ratio of the co-solutions (THF + H_2O);
The solutions were prepared by dissolving 10 mM of **1a** and 40 µM of 4Cz-IPN in 20 mL of dried THF only (**a**), 16 mL dried THF + 4 mL distilled H_2O (**b**), 13 mL dried THF + 7 mL distilled H_2O (**c**), and 10 mL dried THF + 10 mL distilled H_2O (**d**).

Each of the five curves is shown below:
Black solid curve: [4Cz-IPN + **1a**] solution only
Red solid curve: [4Cz-IPN + **1a**] with 50 mM of **2a**
Bule solid curve: [4Cz-IPN + **1a**] with 100 mM of **2a**
Magenta solid curve: [4Cz-IPN + **1a**] with 150 mM of **2a**
Olive solid curve: [4Cz-IPN + **1a**] with 200 mM of **2a**

Comment: Due to the limited solubility of the organic analytes in pure water, co-solutions of water/THF mixture were employed for measurements.

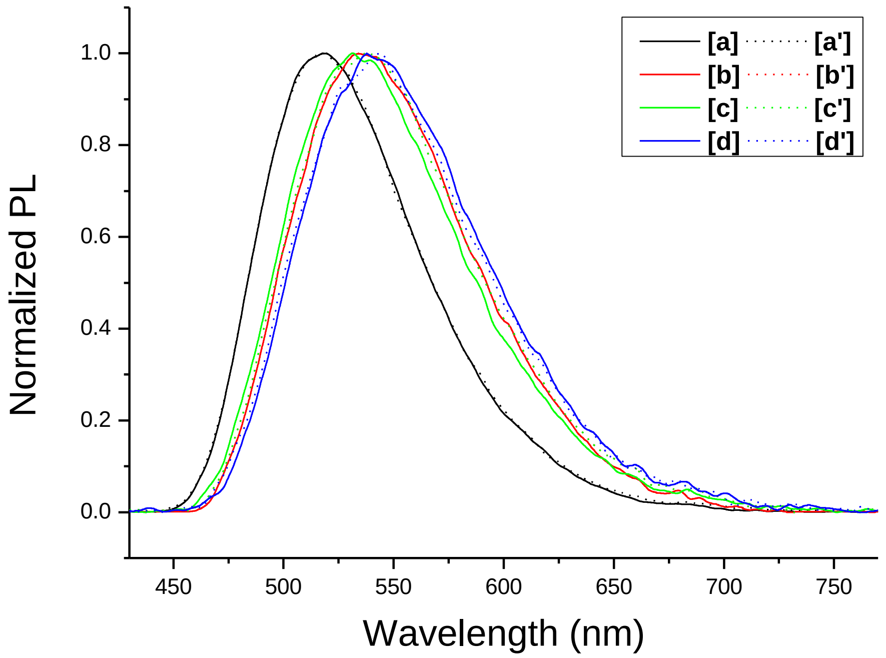

Supplementary Figure 80 Normalized PL spectra

Solutions were prepared by dissolving 10 mM of **1a** and 40 µM of 4Cz-IPN;
[a] in 20 mL of dried THF only (black solid curve).
[a'] in 20 mL of dried THF with 50 mM of **2a** (black dashed curve).
[b] in 16 mL of dried THF + 4 mL of distilled H_2O (red solid curve).
[b'] in 16 mL of dried THF + 4 mL of distilled H_2O with 50 mM of **2a** (red dashed curve).
[c] in 13 mL of dried THF + 7 mL of distilled H_2O (green solid curve).
[c'] in 13 mL of dried THF + 7 mL of distilled H_2O with 50 mM of **2a** (green dashed curve).
[d] in 10 mL of dried THF + 10 mL of distilled H_2O (blue solid curve).
[d'] in 10 mL of dried THF + 10 mL of distilled H_2O (blue dashed curve).

Comment: As the water content in the co-solution increases, a noticeable red shift in the maximum emission wavelength becomes evident in the normalized PL spectrum. This shift signifies that an increase in the water proportion fosters the formation of an exciplex due to the composition approaching high-pressure-like conditions.

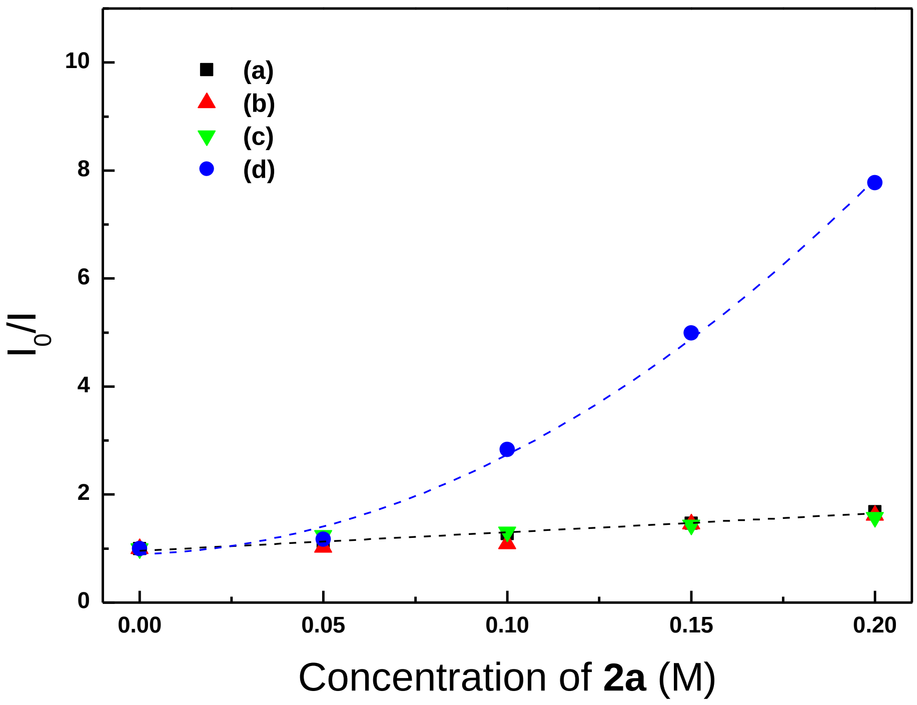

Supplementary Figure 81 Stern–Volmer plot

Comment: A Stern–Volmer plot was displayed, revealing a linear rise in the PL intensity ratio with the concentration of the quencher [**2a**] within the homogeneous solution (represented by the black dashed line). In sharp contrast, an exponential increment in the PL intensity was observed as the solution heterogeneity increased (indicated by the blue dashed line).

Control experiments using THF/H_2O mixed solvent (1:1 v/v)

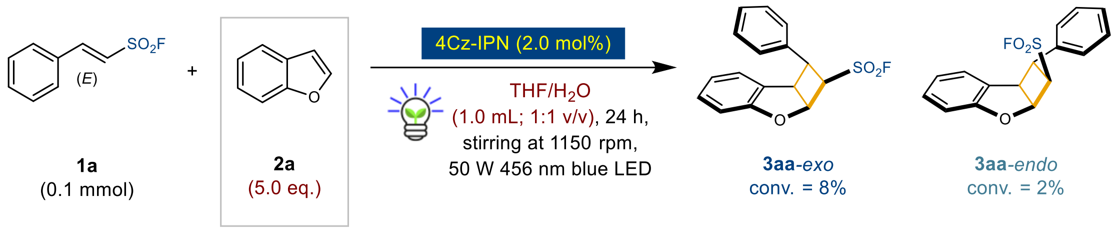

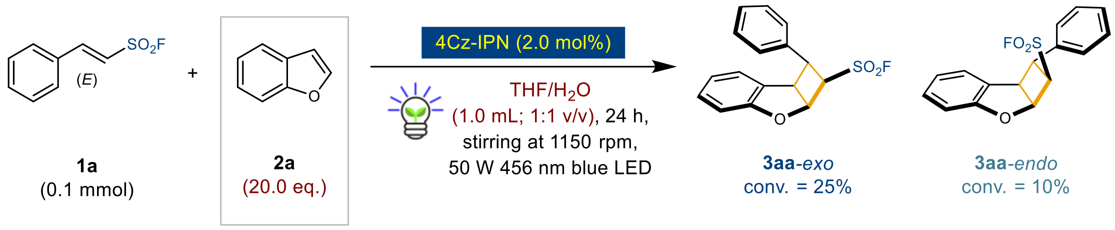

1a (0.1 mmol, 1.0 eq.) and 4Cz-IPN (2.0 mol%) were added in a vial without further drying with multiple magnetic stirring bars. Subsequently, **2a** (0.5 mmol: 5.0 eq. or 2.0 mmol: 20.0 eq.) was added with H_2O (deionized, 0.5 mL) and dried THF (0.5 mL) in the reaction mixture without further degassing, and then stirred vigorously (rpm > 1000) under irradiation with a 50 W 456 nm blue LED for 24 h. In this case, 1,4-dimethoxybenzene (0.1 mmol, 1.0 eq.) was used as a standard on the crude ^{1}H NMR analysis to determine conversion (conv.) and *exo*-/*endo*-ratio.

Comment: The reaction was conducted using a mixed solvent ratio of THF : H_2O = 1 : 1 (v/v). As the equivalent of **2a** was raised, the heterogeneity between the organic and water phases increased (the mixed solvent exhibited higher heterogeneity). Consequently, a slight enhancement in reactivity was observed, in line with the Stern-Volmer plot results that indicated a more efficient quenching effect when employing 20 equivalents (instead of 5 equivalents).

Reaction kinetics

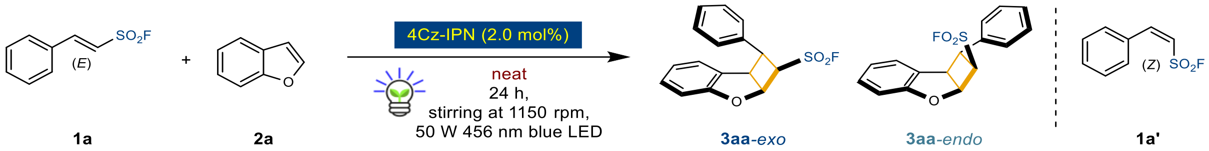

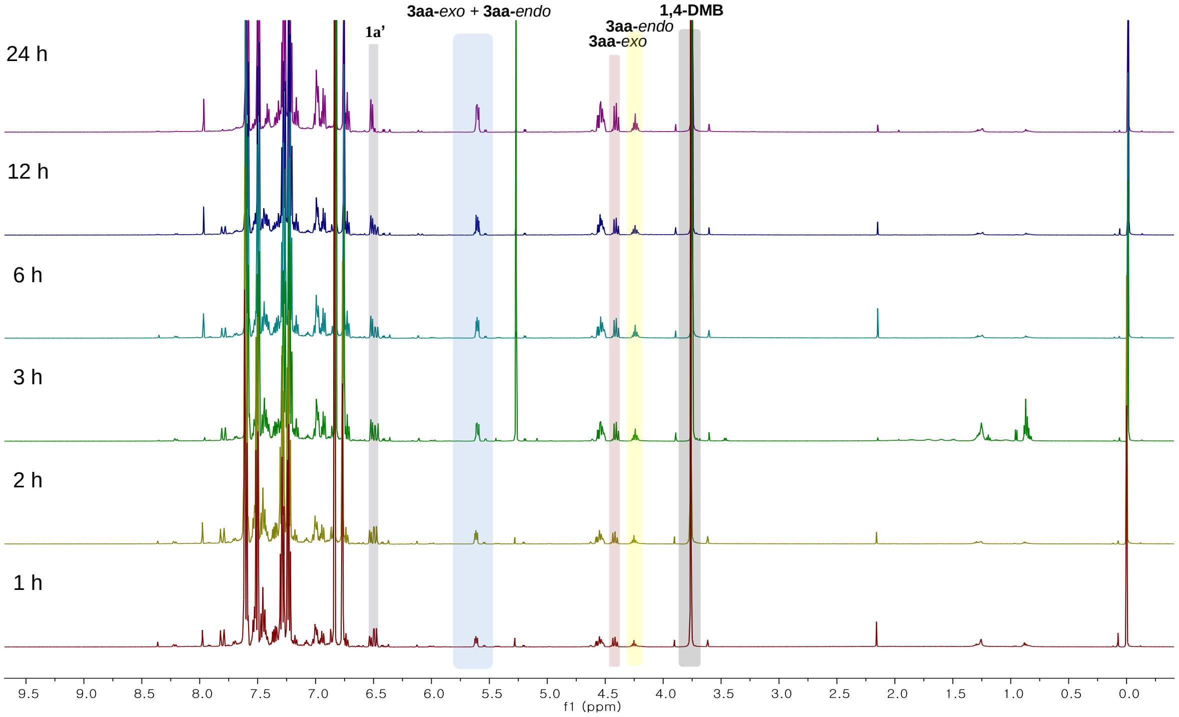

Supplementary Figure 82 Stacked ^{1}H NMR spectra of the reaction performed under neat condition (500 MHz, $CDCl_3$, 25 °C)

1a (0.1 mmol, 1.0 eq.) and 4Cz-IPN (2.0 mol%) were added in a vial without further drying with multiple magnetic stirring bars. Subsequently, **2a** (0.5 mmol, 5.0 eq.) was added with H_2O (deionized, 10 L/mol, 1.0 mL) in the reaction mixture without further degassing, and then stirred vigorously (rpm > 1000) under irradiation with a 50 W 456 nm blue LED. In this case, 1,4-dimethoxybenzene (1,4-DMB; 0.1 mmol, 1.0 eq.) was used as a standard on crude ^{1}H NMR analysis to confirm conversion and *exo*-/*endo*-ratio depending on each time scale.

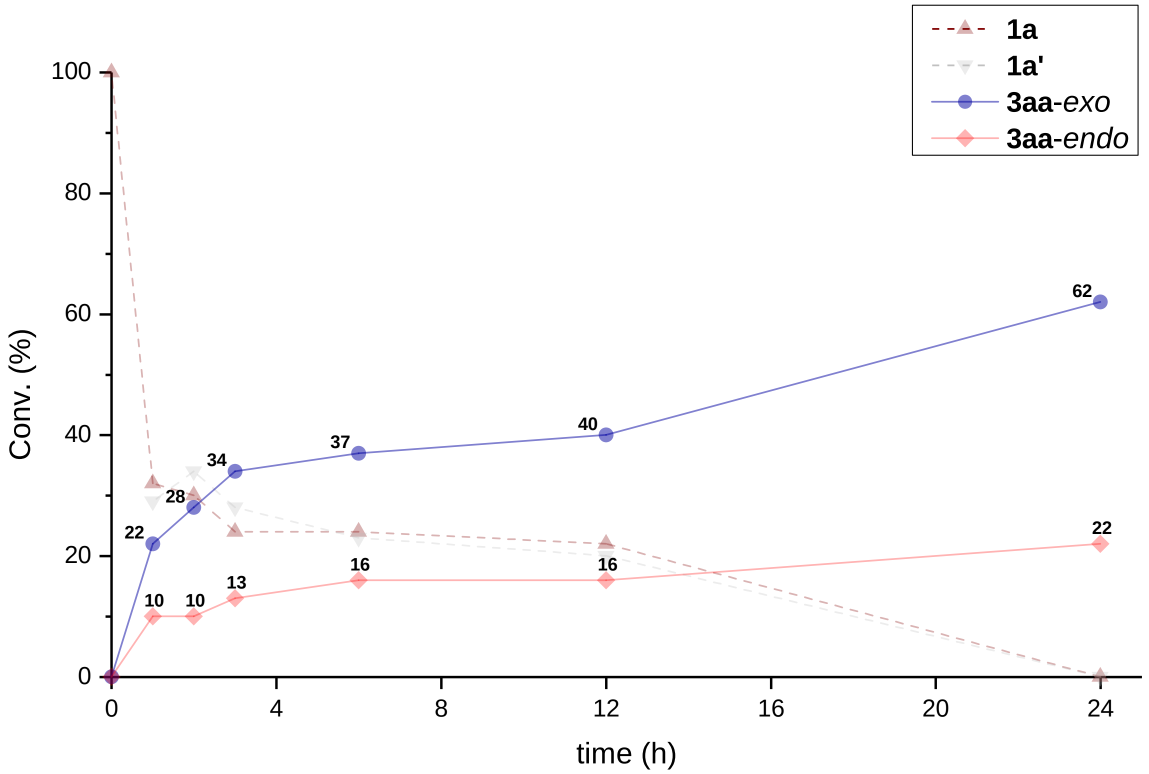

Supplementary Figure 83 Kinetic profile of the reaction progress under neat condition.

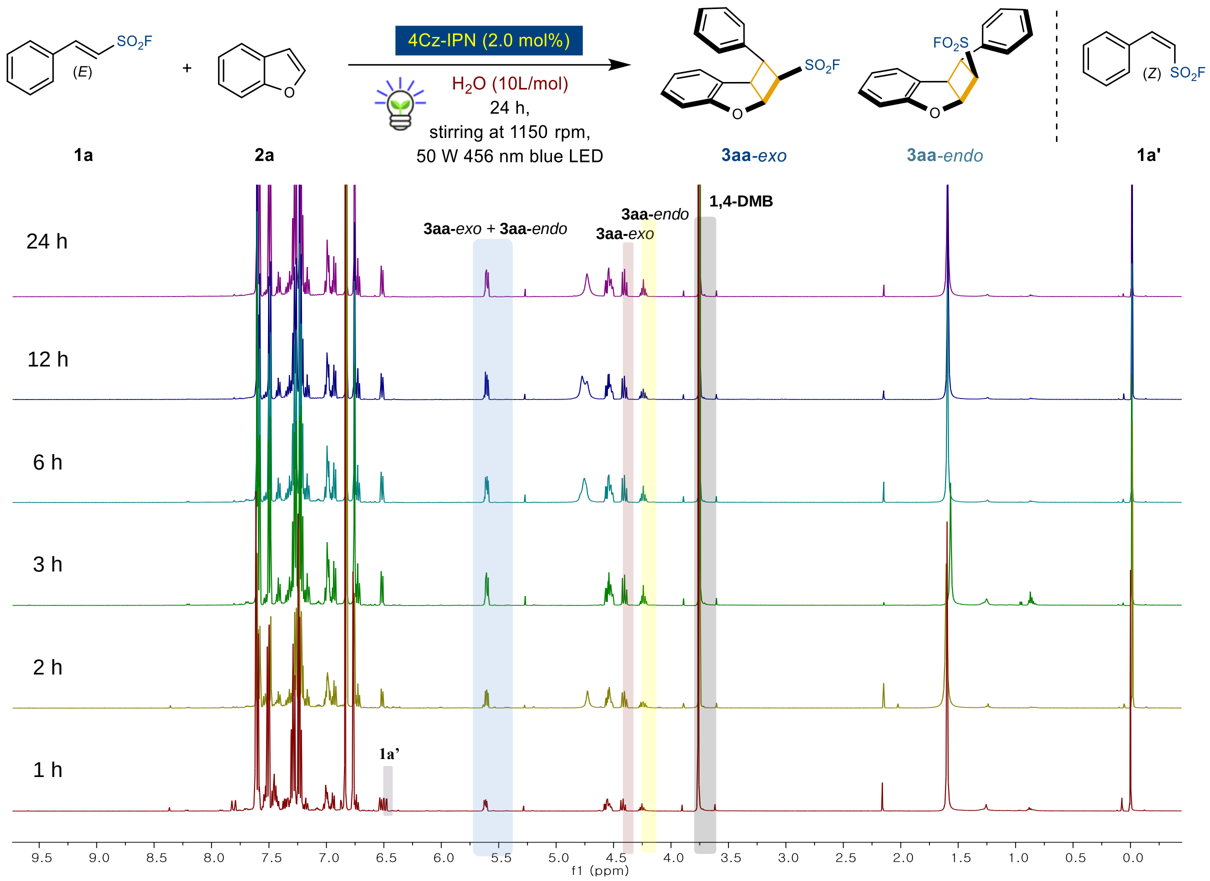

Supplementary Figure 84 Stacked ^{1}H NMR spectra of the reaction performed to the on-water condition (500 MHz, $CDCl_3$, 25 °C).

1a (0.1 mmol, 1.0 eq.) and 4Cz-IPN (2.0 mol%) were added in a vial without further drying with multiple magnetic stirring bars. Subsequently, **2a** (0.5 mmol, 5.0 eq.) was added with H_2O (deionized, 10 L/mol, 1.0 mL) in the reaction mixture without further degassing, and then stirred vigorously (rpm > 1000) under irradiation with a 50 W 456 nm blue LED. In this case, 1,4-dimethoxybenzene (1,4-DMB; 0.1 mmol, 1.0 eq.) was used as a standard on the crude ^{1}H NMR analysis to confirm conversion and *exo-*/*endo*-ratio depending on each time scale.

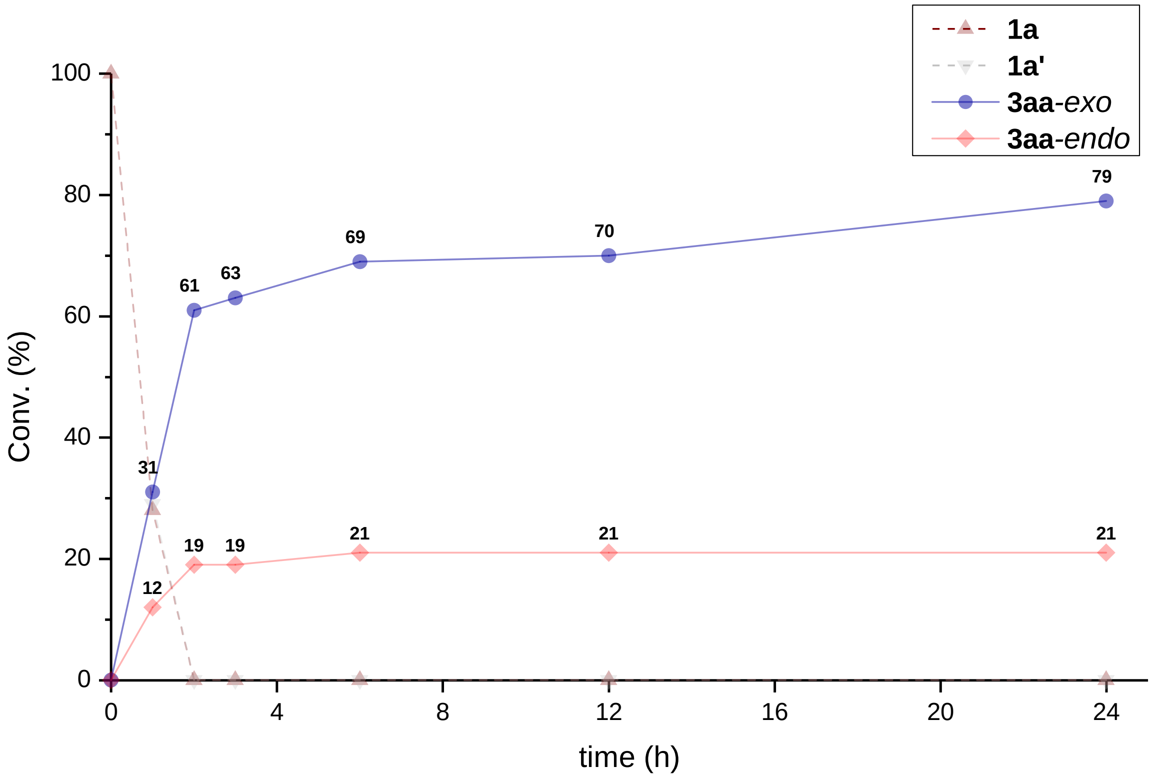

Supplementary Figure 85 Kinetic profile for the reaction progress of the on-water condition.

Study on the stirring speed

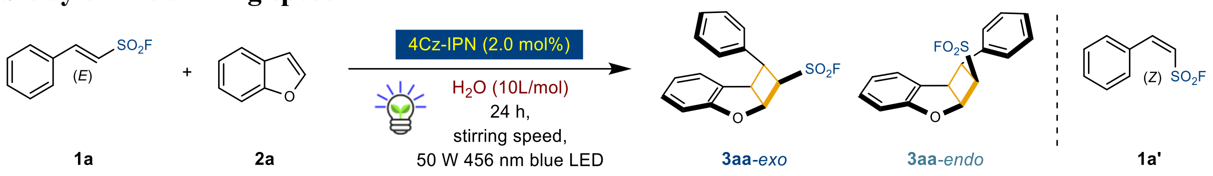

1a (0.1 mmol, 1.0 eq.) and 4Cz-IPN (2.0 mol%) were added in a vial without further drying with multiple magnetic stirring bars. Subsequently, **2a** (0.5 mmol, 5.0 eq.) was added with H_2O (deionized, 10 L/mol, 1.0 mL) in the reaction mixture without further degassing, and then stirred under irradiation with a 50 W 456 nm blue LED. In this case, 1,4-dimethoxybenzene (0.1 mmol, 1.0 eq.) was used as a standard on the crude ^{1}H NMR analysis to confirm conversion and *exo-*/*endo*-ratio depending on each stirring speed.

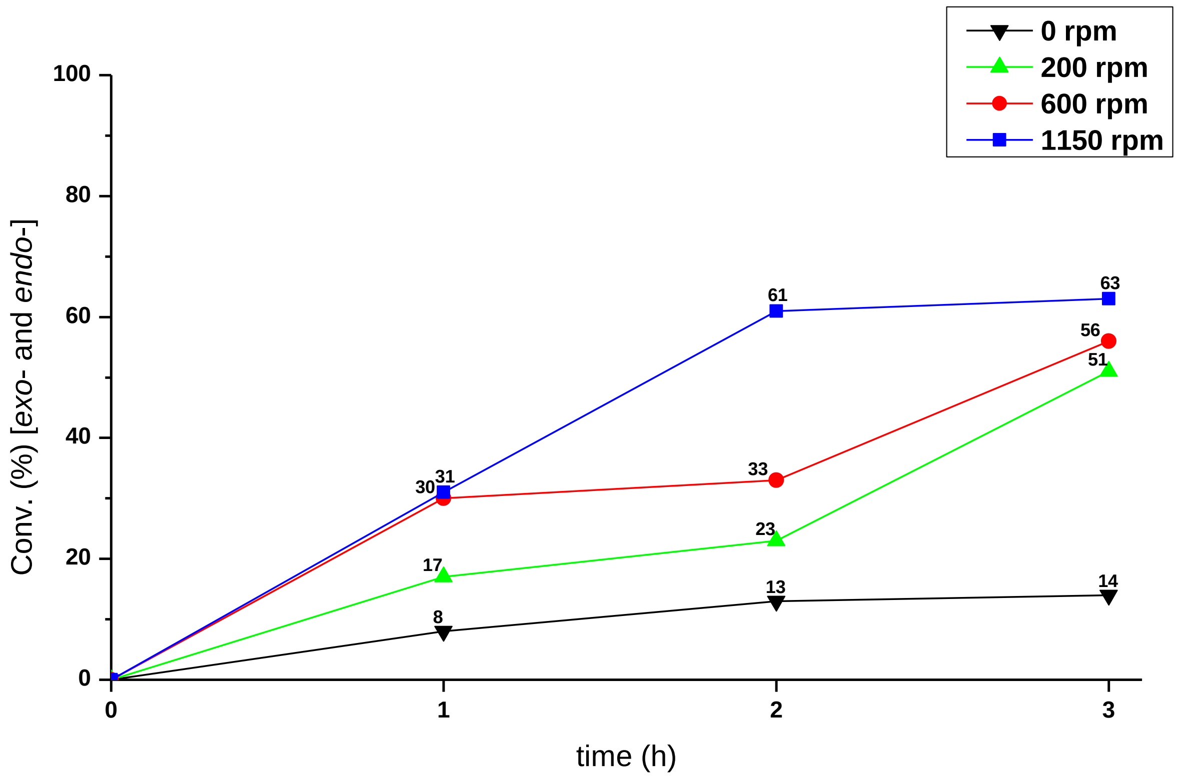

Supplementary Figure 86 Conv. (%) of desired cycloadducts depending on the stirring speed. Conv. (%) = conversion.

Supplementary Note

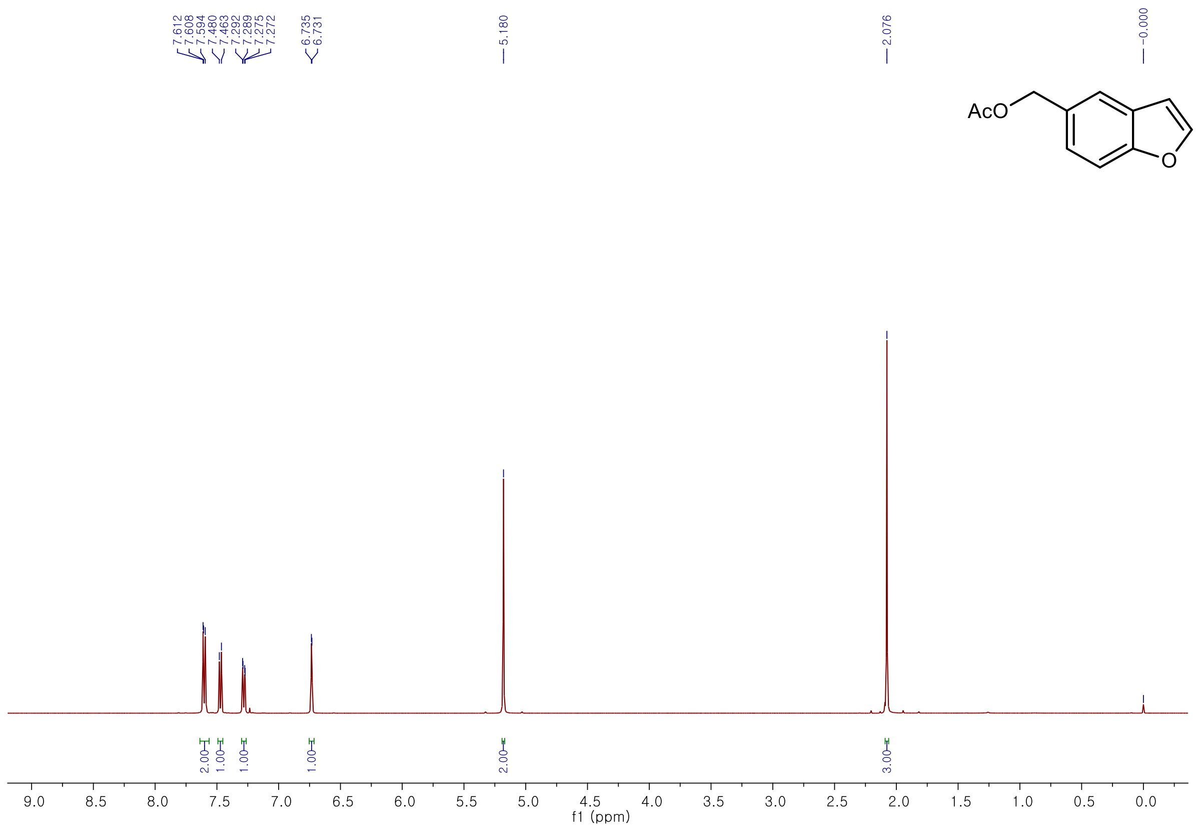

^{1}H NMR spectrum (**2h**)

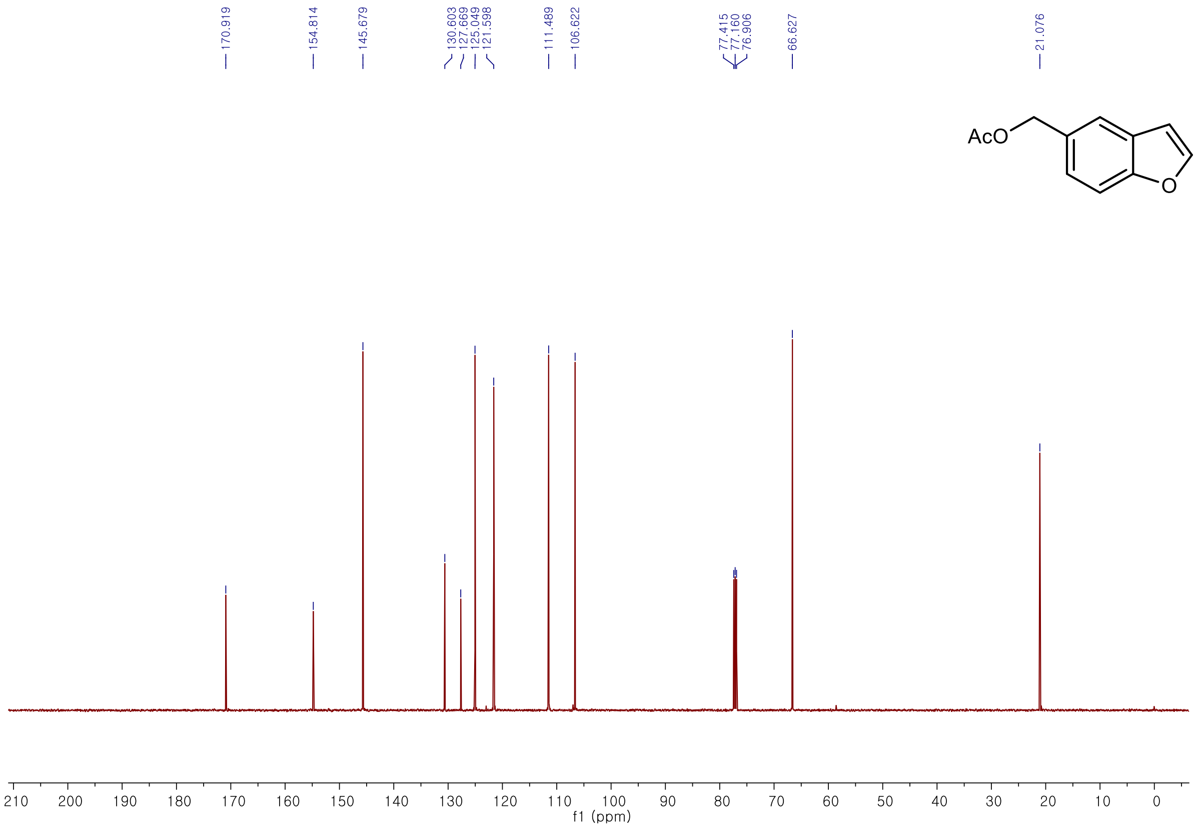

^{13}C NMR spectrum (**2h**)

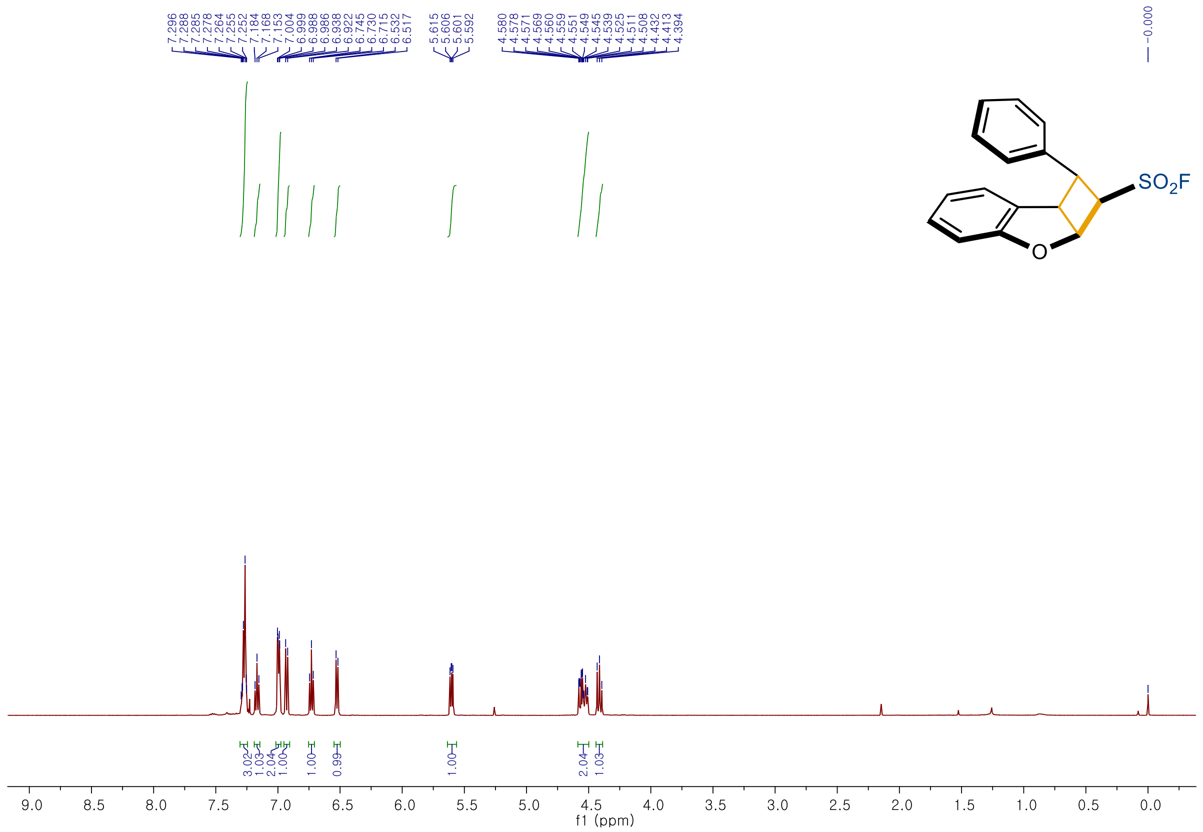

^{1}H NMR spectrum (**3aa**-*exo*)

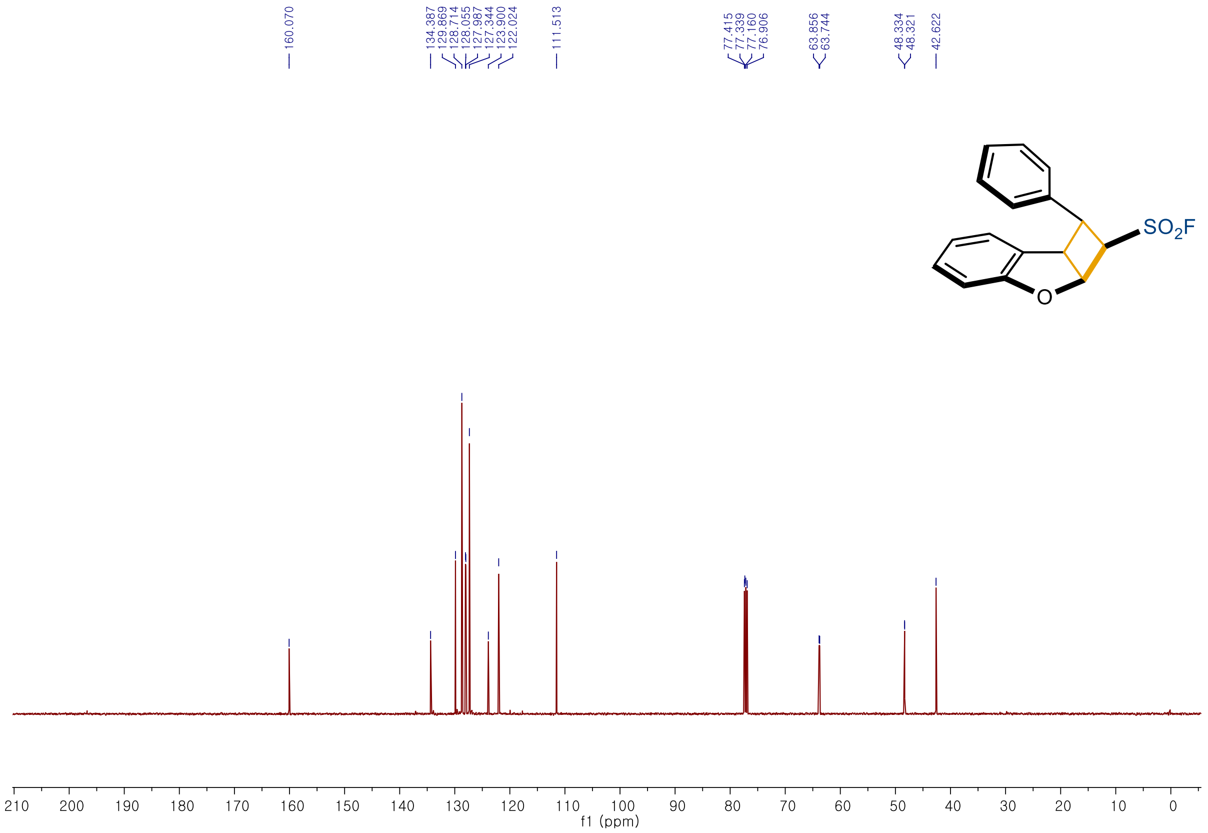

^{13}C NMR spectrum (**3aa**-*exo*)

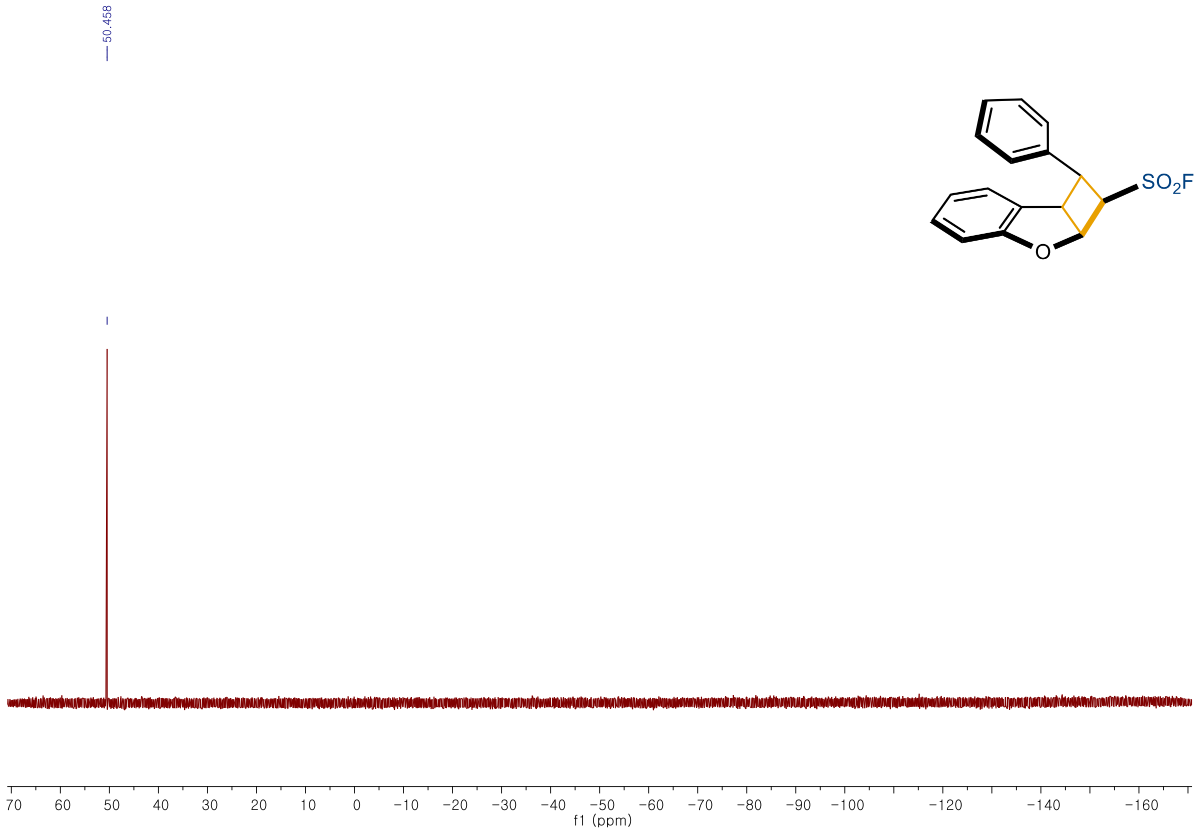

^{19}F NMR spectrum (**3aa**-*exo*)

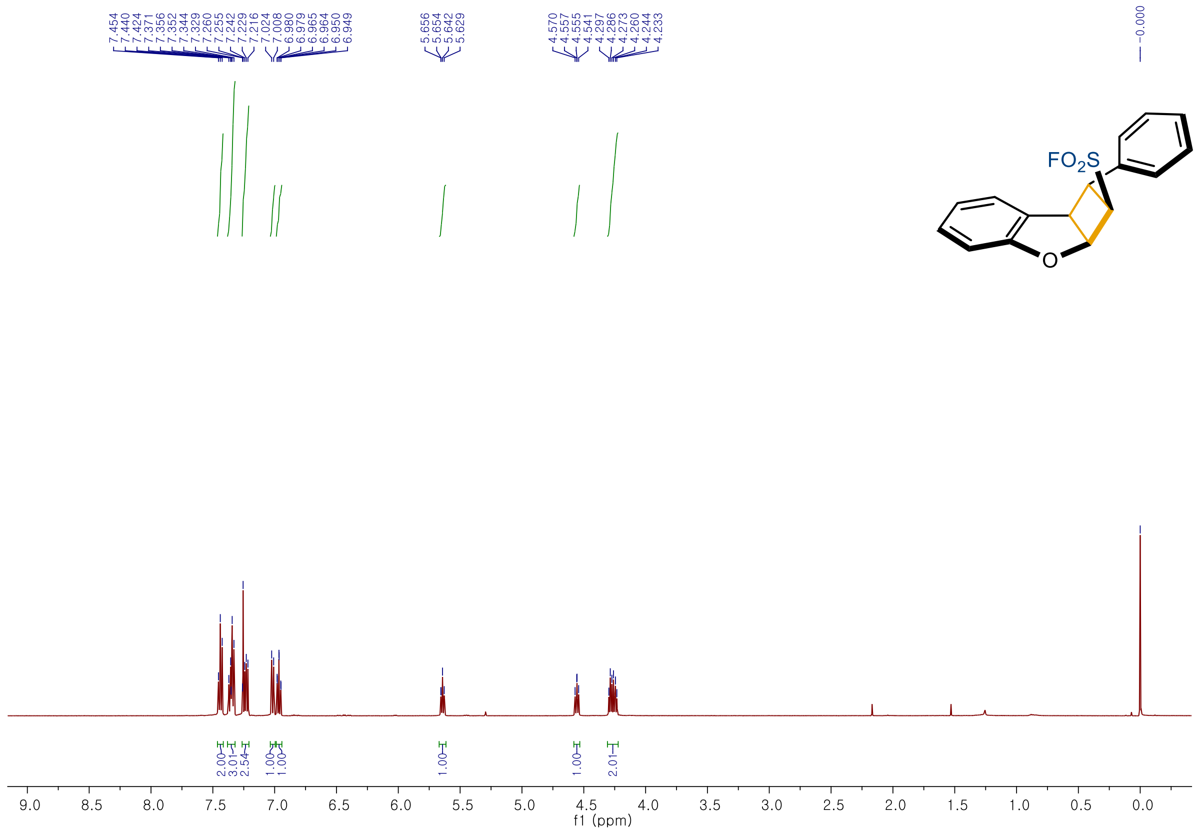

^{1}H NMR spectrum (**3aa**-*endo*)

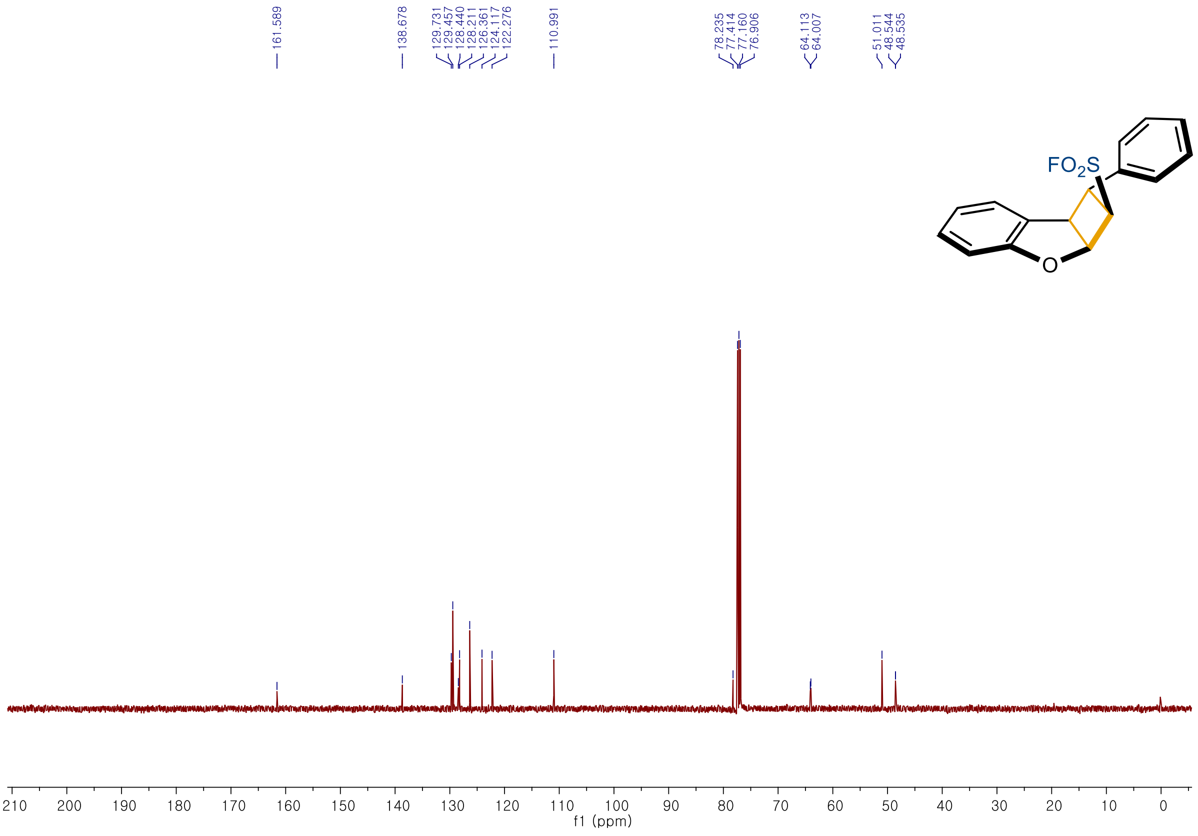

^{13}C NMR spectrum (**3aa**-*endo*)

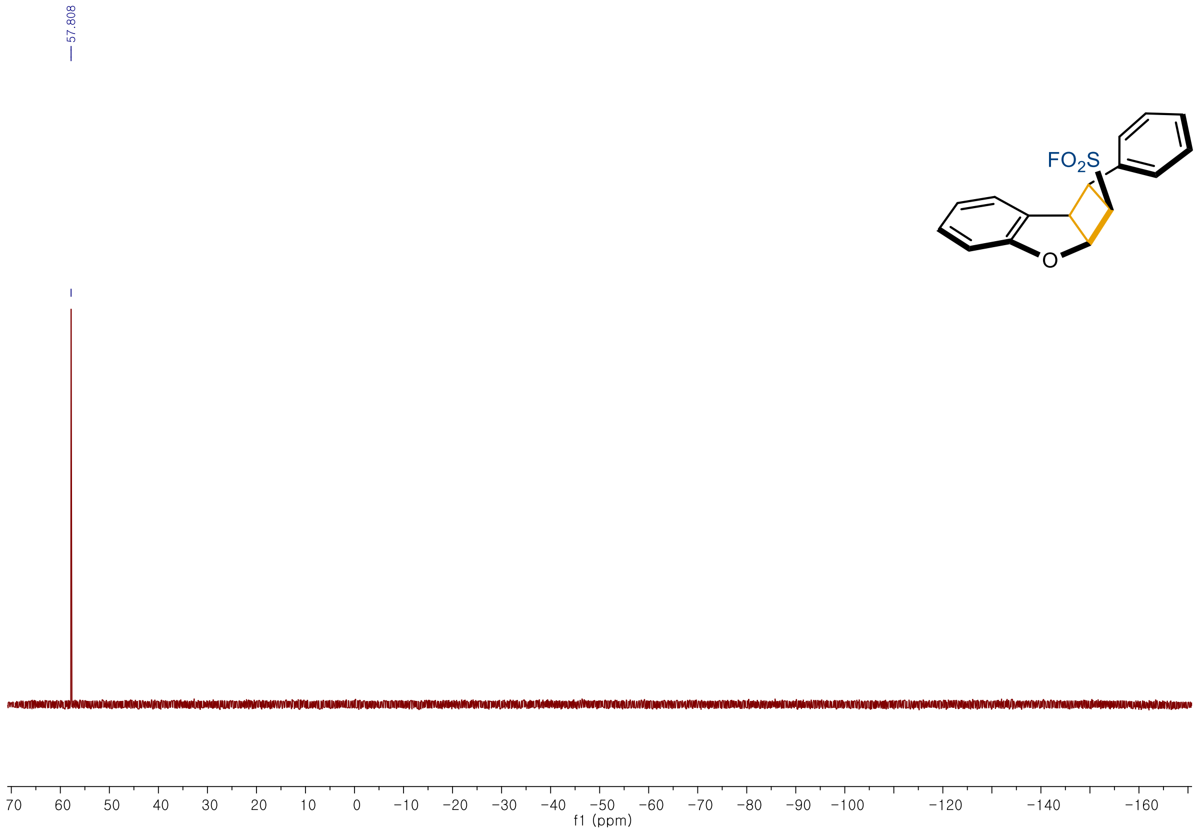

^{19}F NMR spectrum (**3aa**-*endo*)

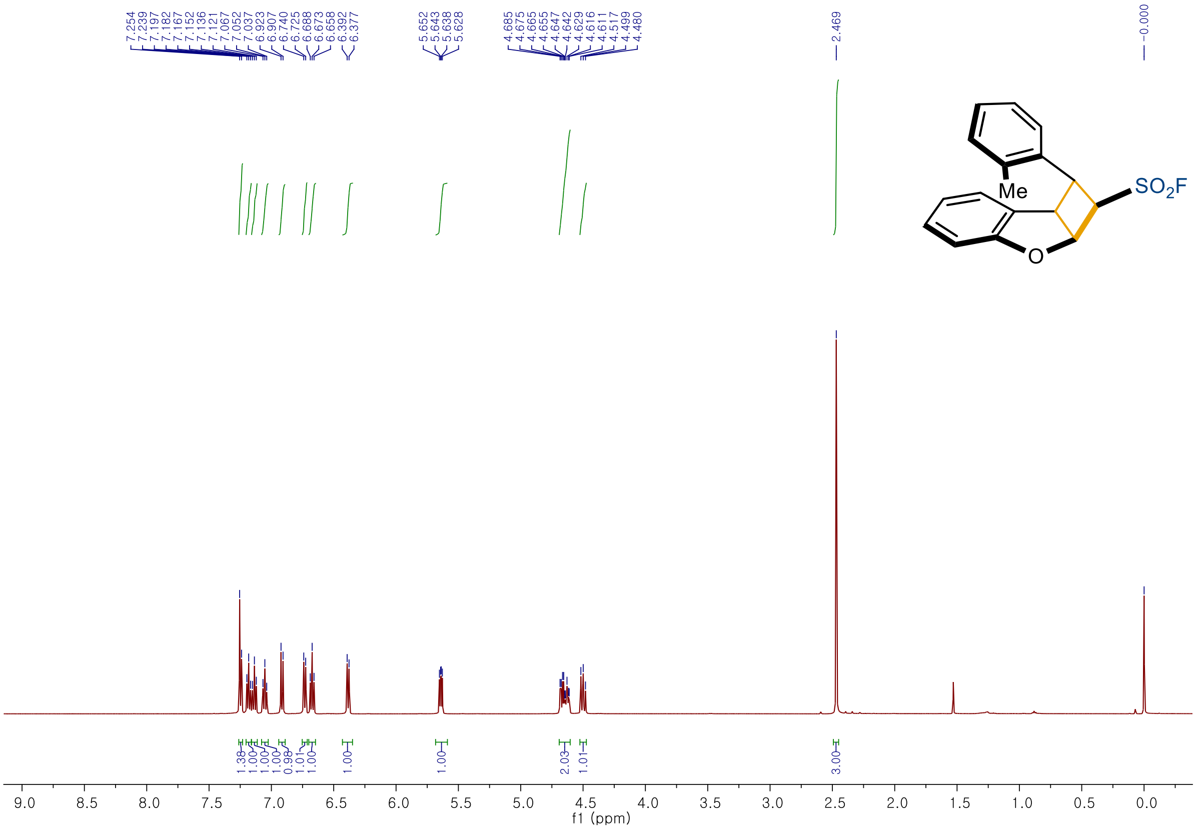

^{1}H NMR spectrum (**3ba**-*exo*)

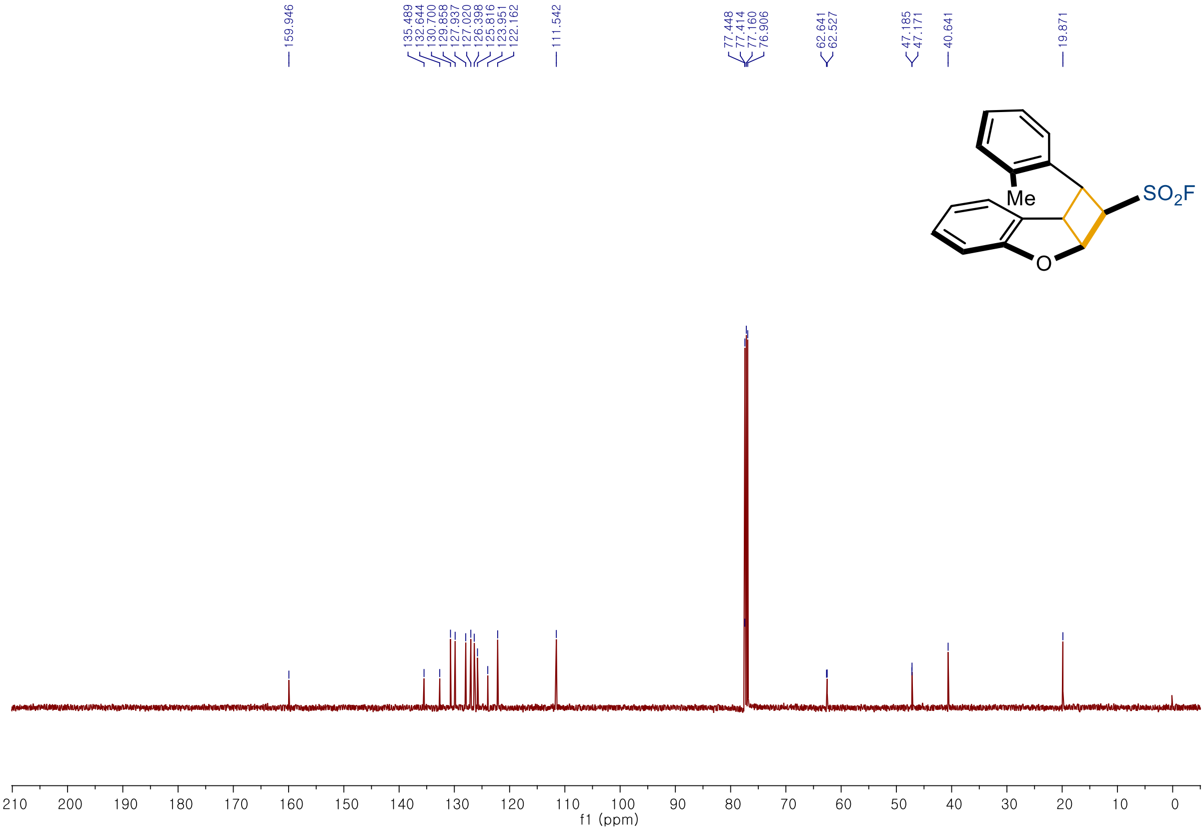

^{13}C NMR spectrum (**3ba**-*exo*)

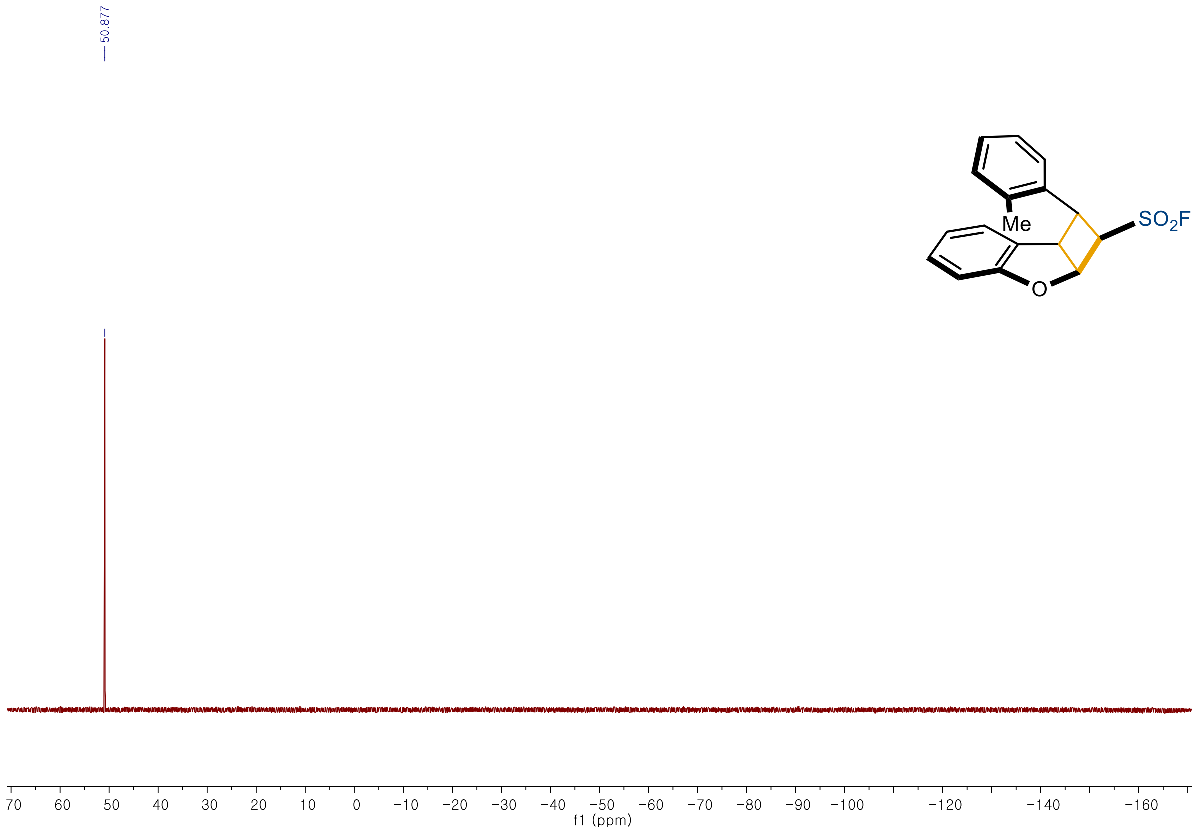

^{19}F NMR spectrum (**3ba**-*exo*)

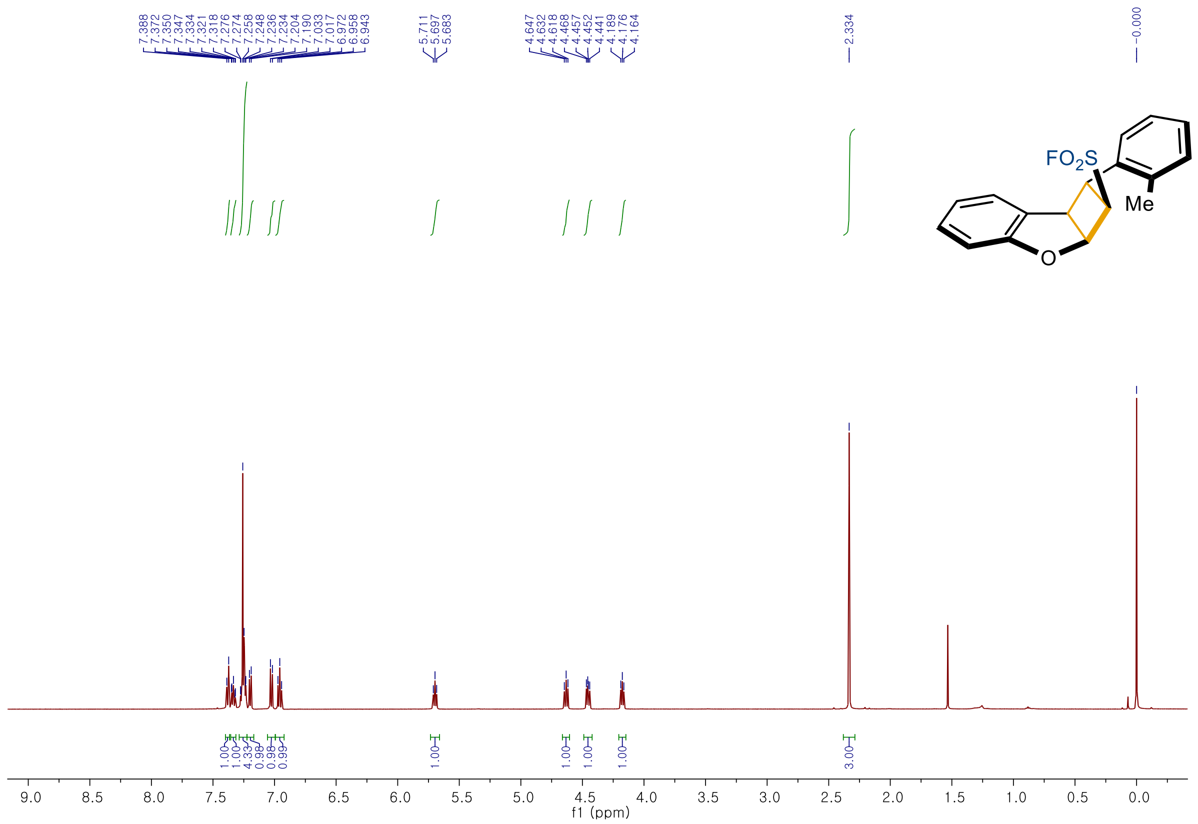

^{1}H NMR spectrum (**3ba**-*endo*)

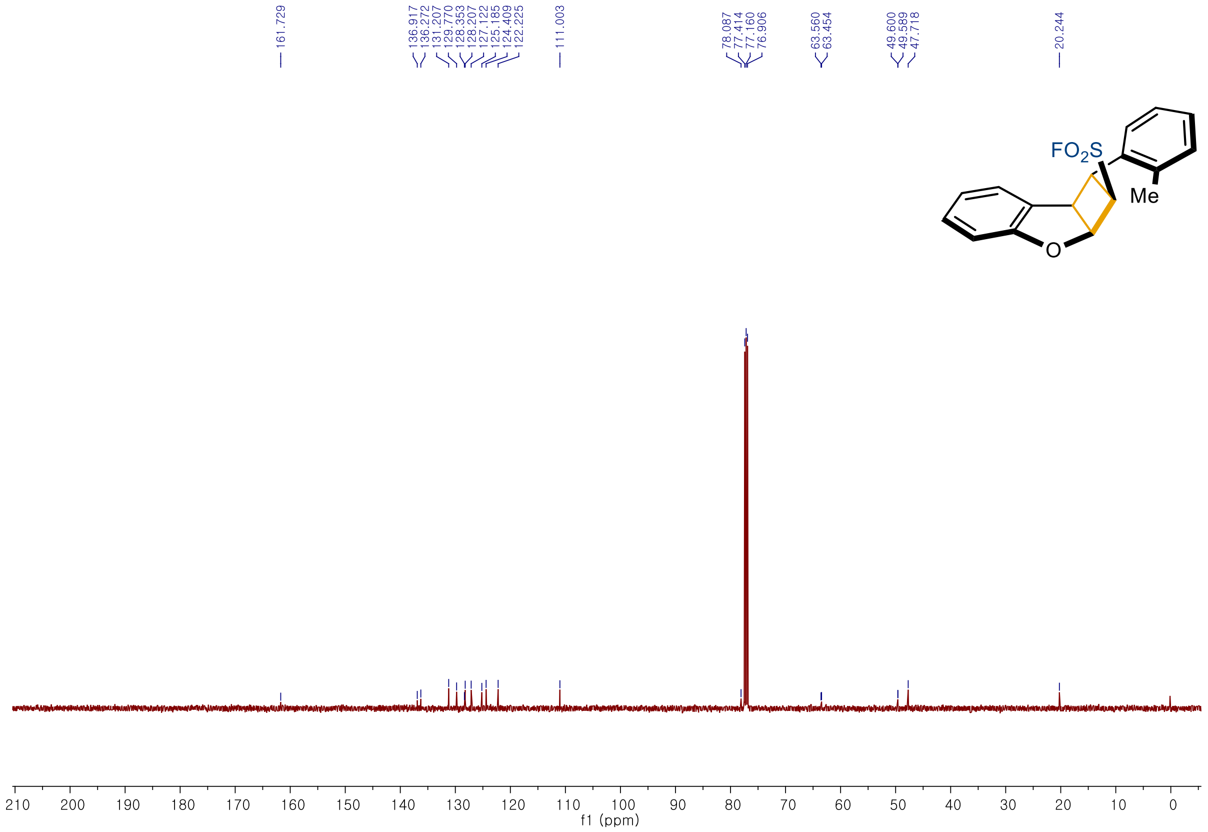

^{13}C NMR spectrum (**3ba**-*endo*)

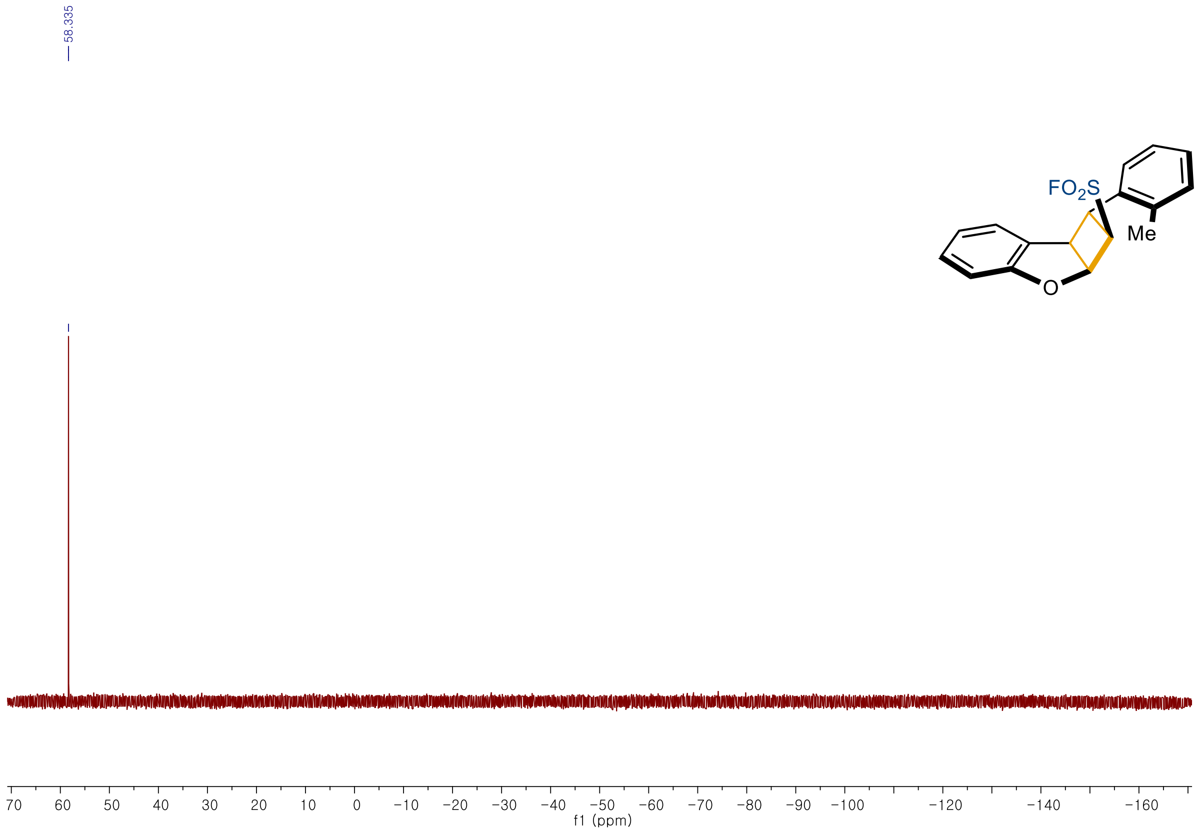

^{19}F NMR spectrum (**3ba**-*endo*)

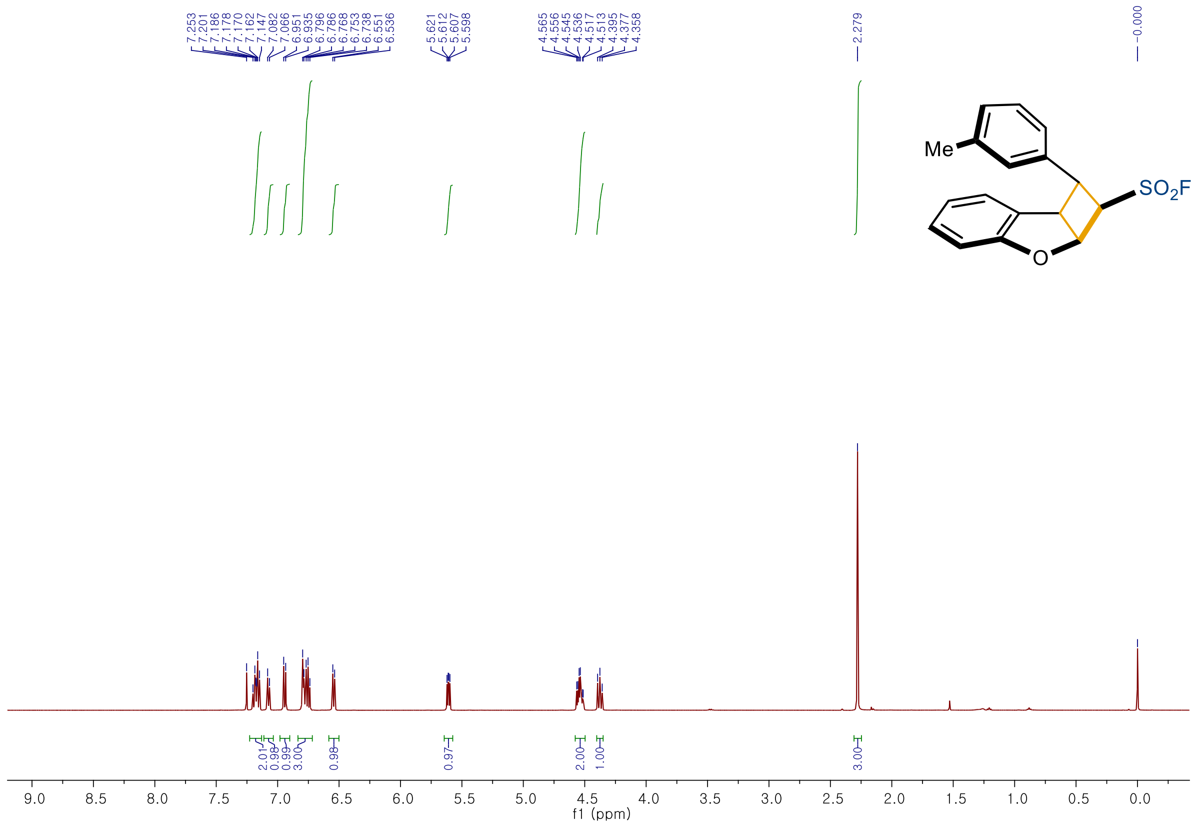

^{1}H NMR spectrum (**3ca**-*exo*)

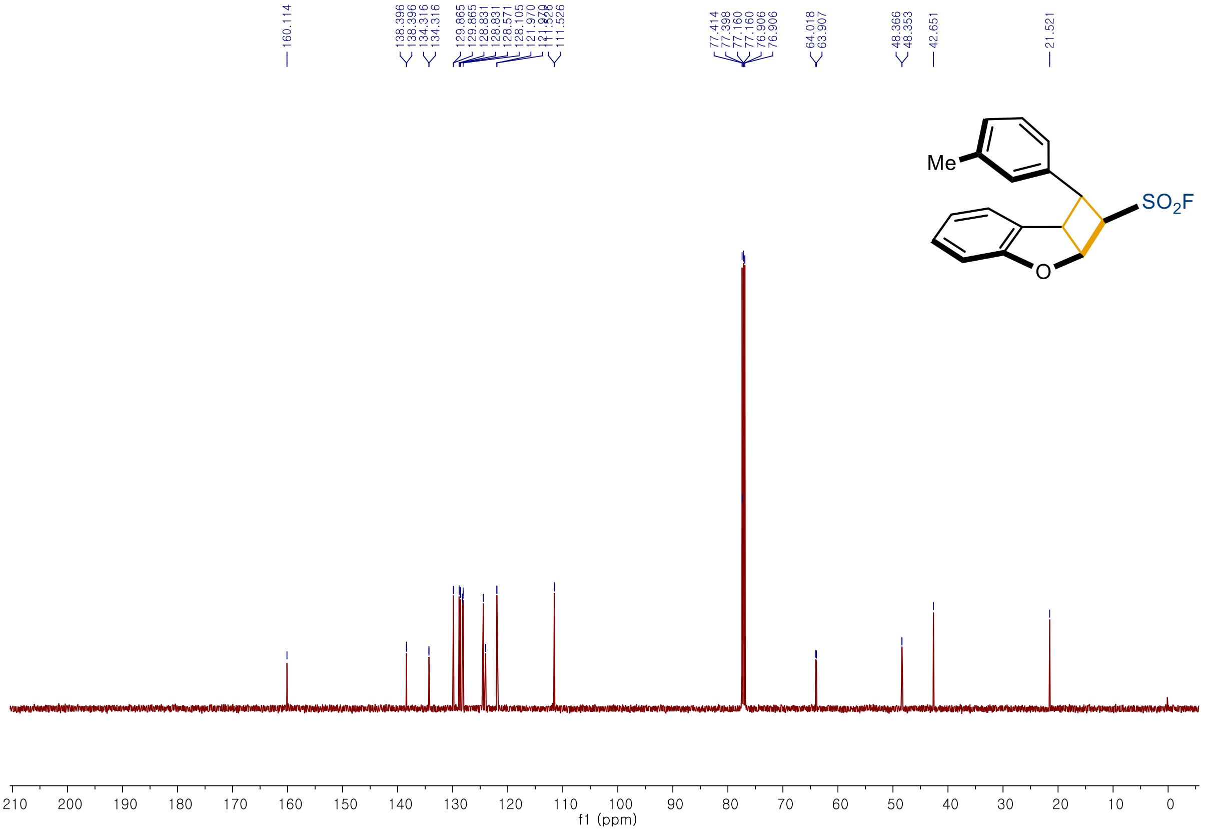

^{13}C NMR spectrum (**3ca**-*exo*)

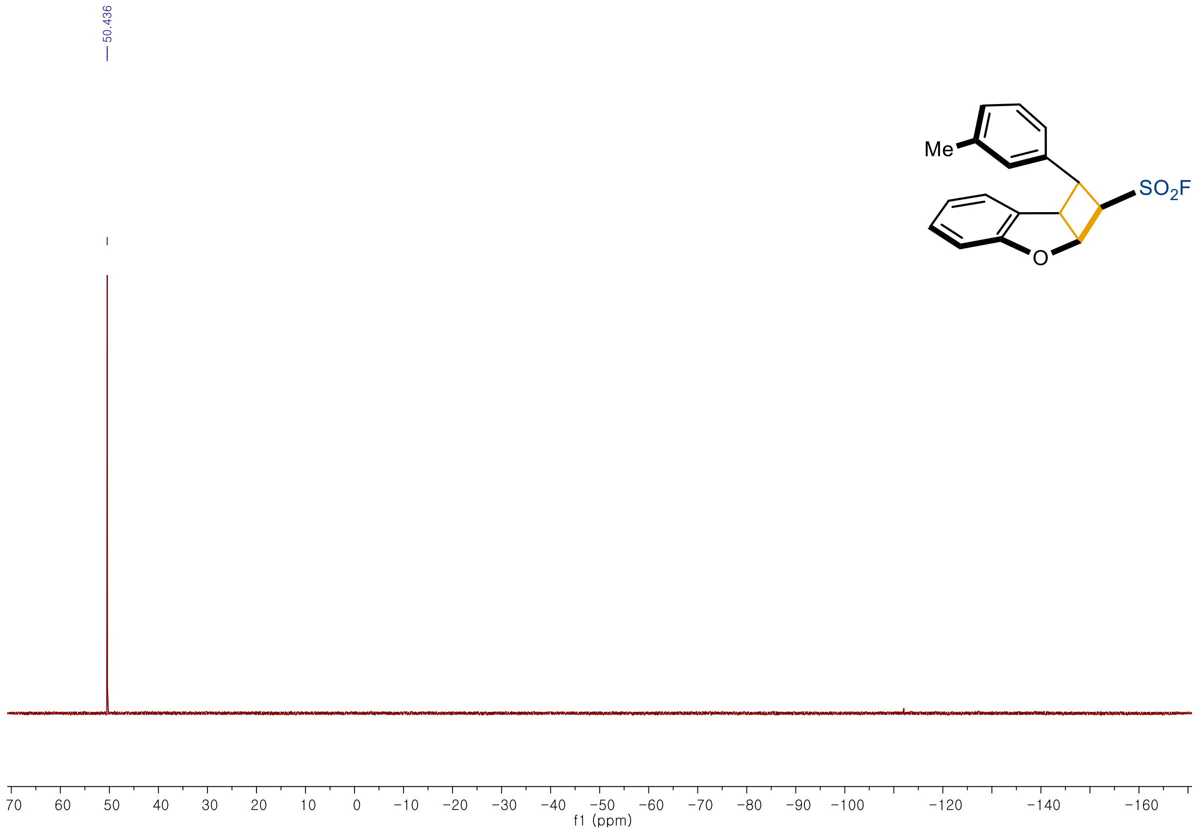

^{19}F NMR spectrum (**3ca**-*exo*)

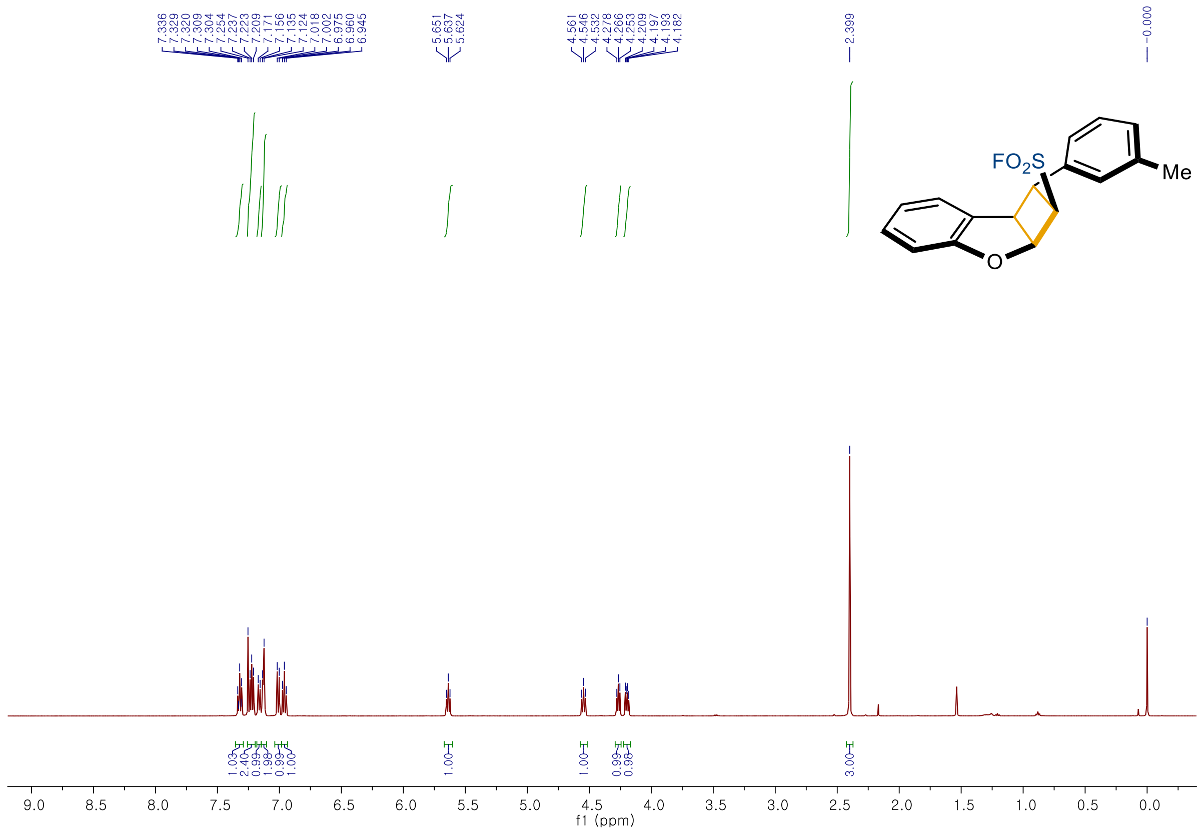

^{1}H NMR spectrum (**3ca**-*endo*)

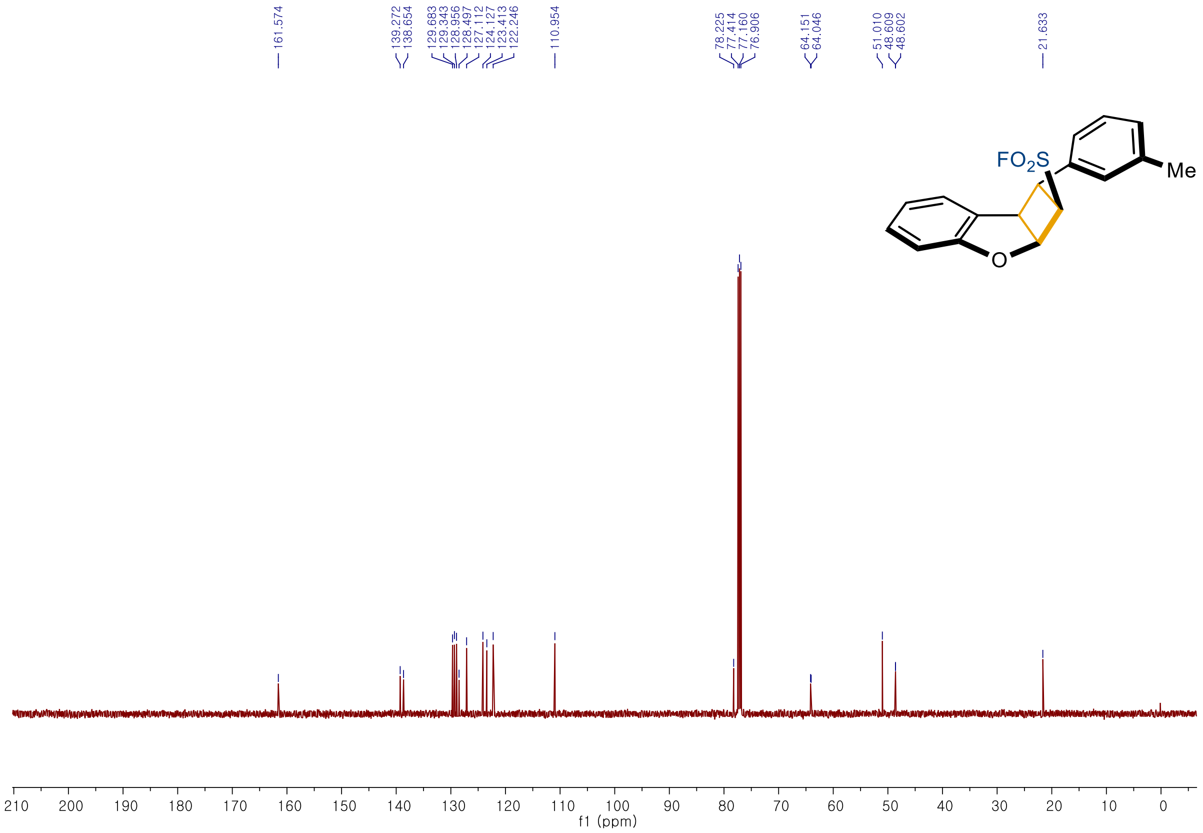

^{13}C NMR spectrum (**3ca**-*endo*)

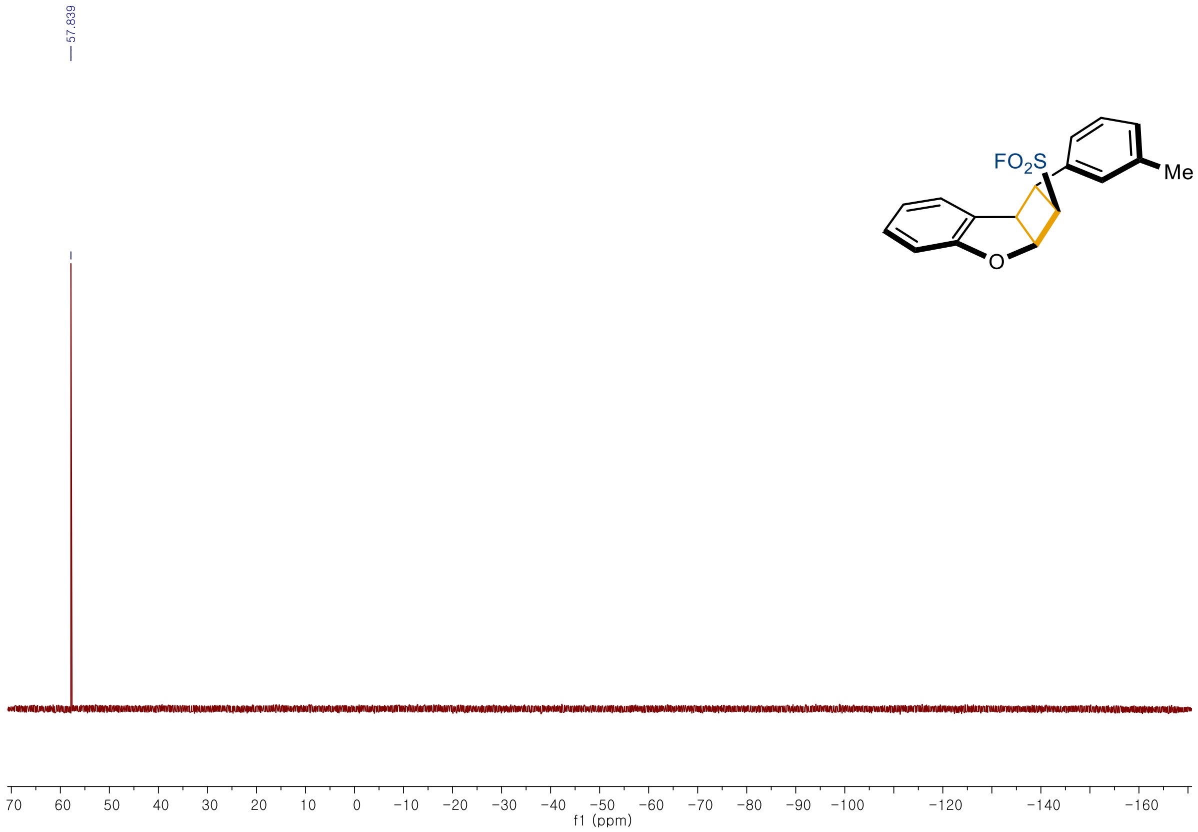

^{19}F NMR spectrum (**3ca**-*endo*)

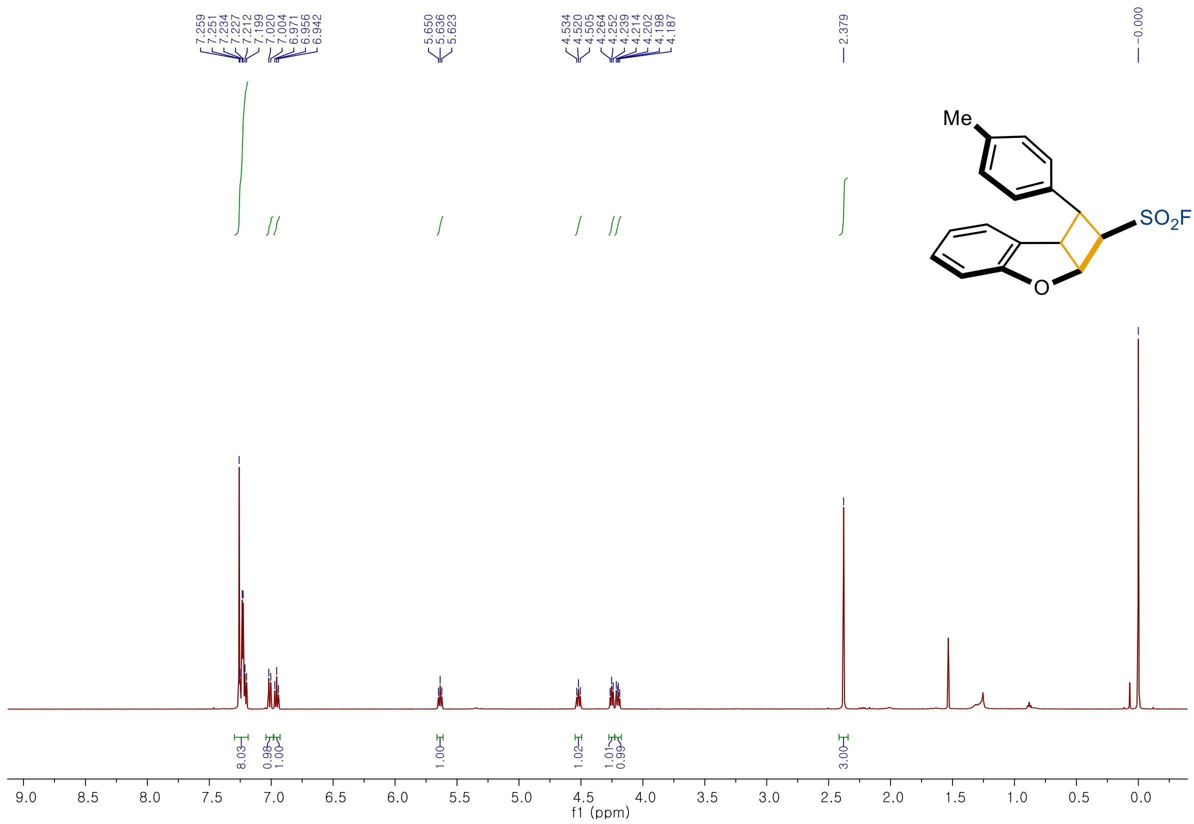

^{1}H NMR spectrum (**3da**-*exo*)

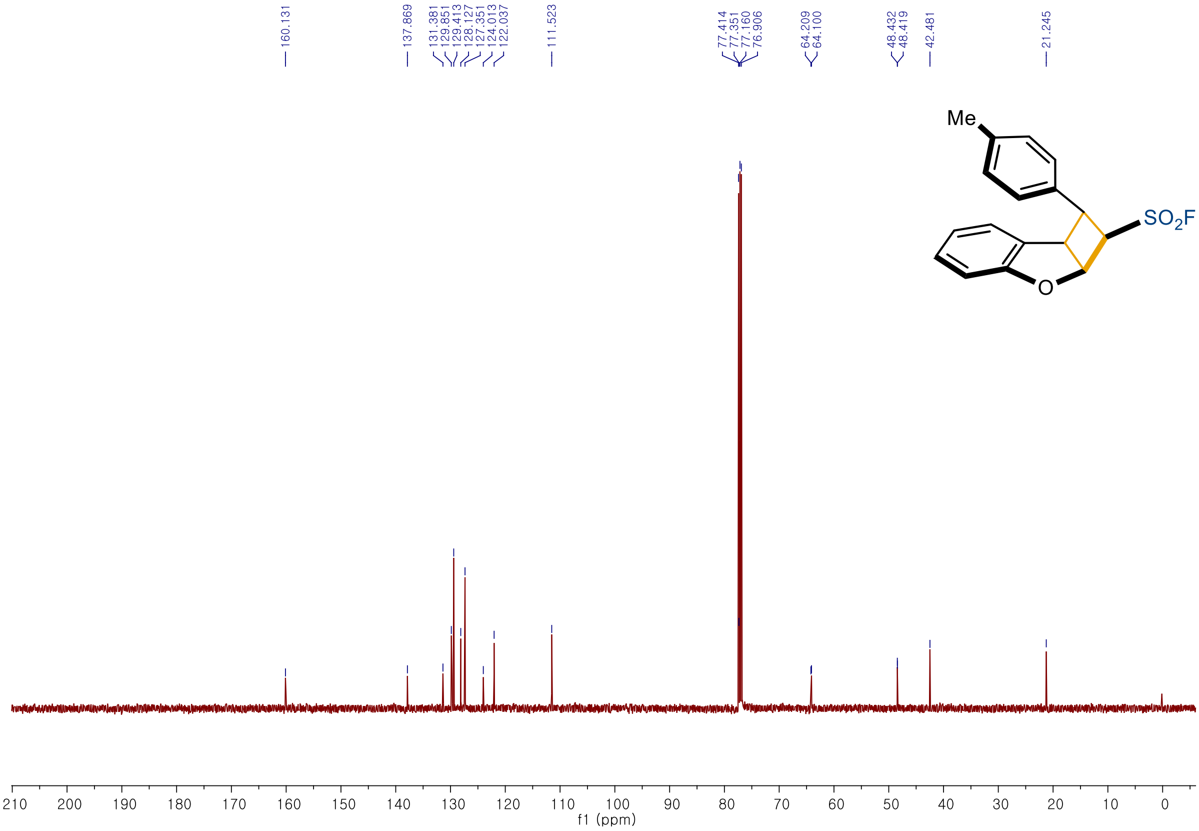

^{13}C NMR spectrum (**3da**-*exo*)

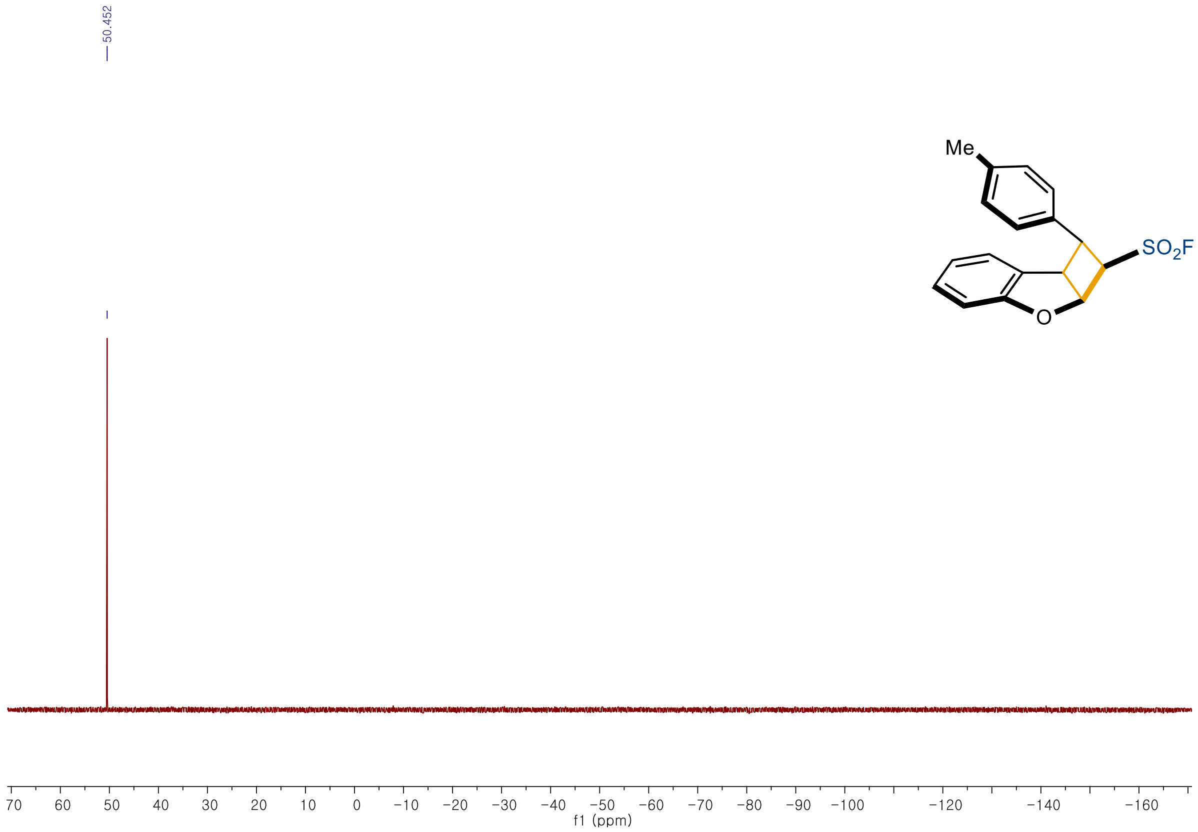

^{19}F NMR spectrum (**3da**-*exo*)

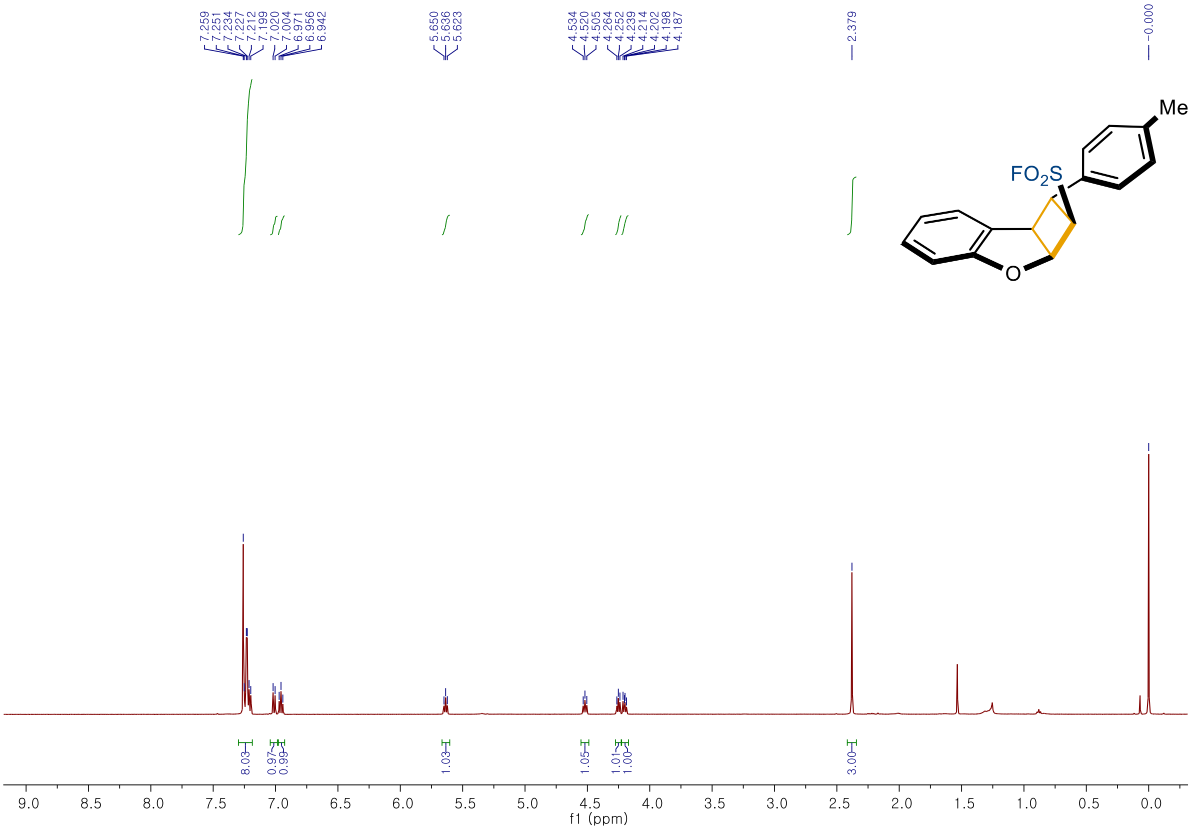

^{1}H NMR spectrum (**3da**-*endo*)

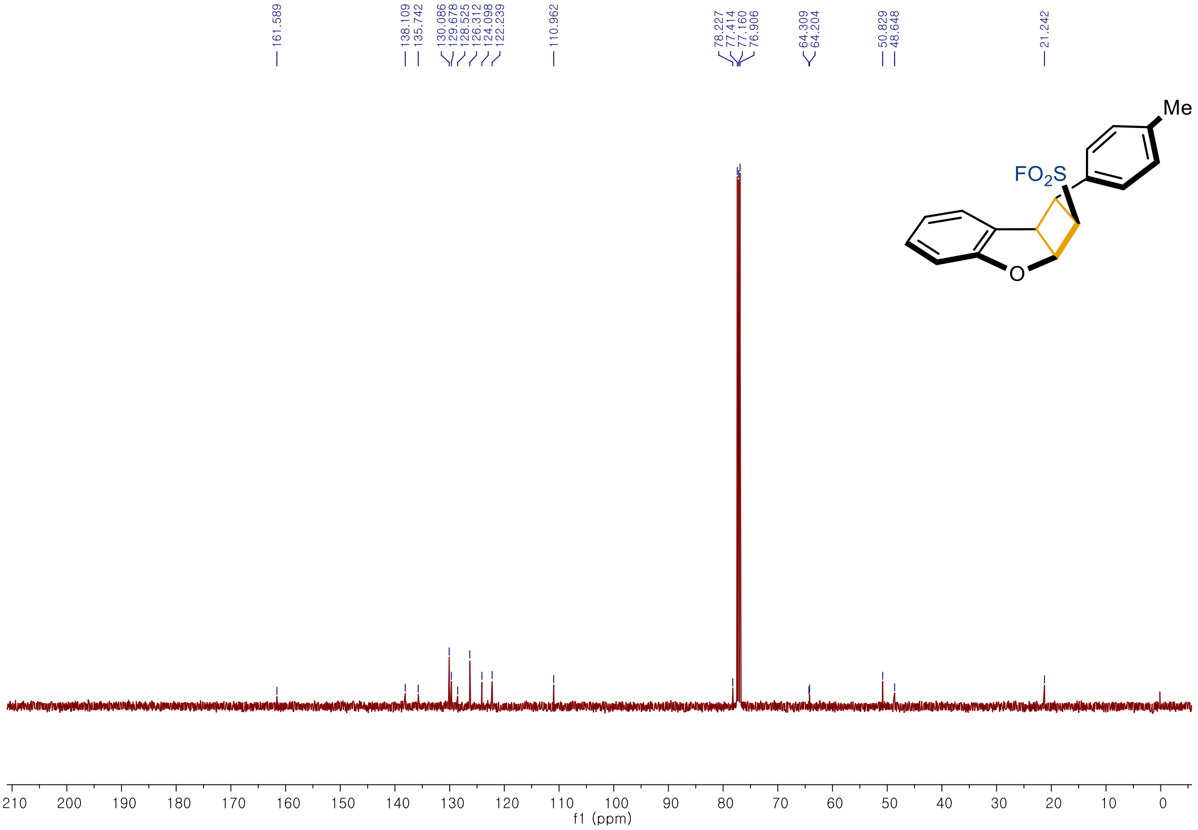

^{13}C NMR spectrum (**3da**-*endo*)

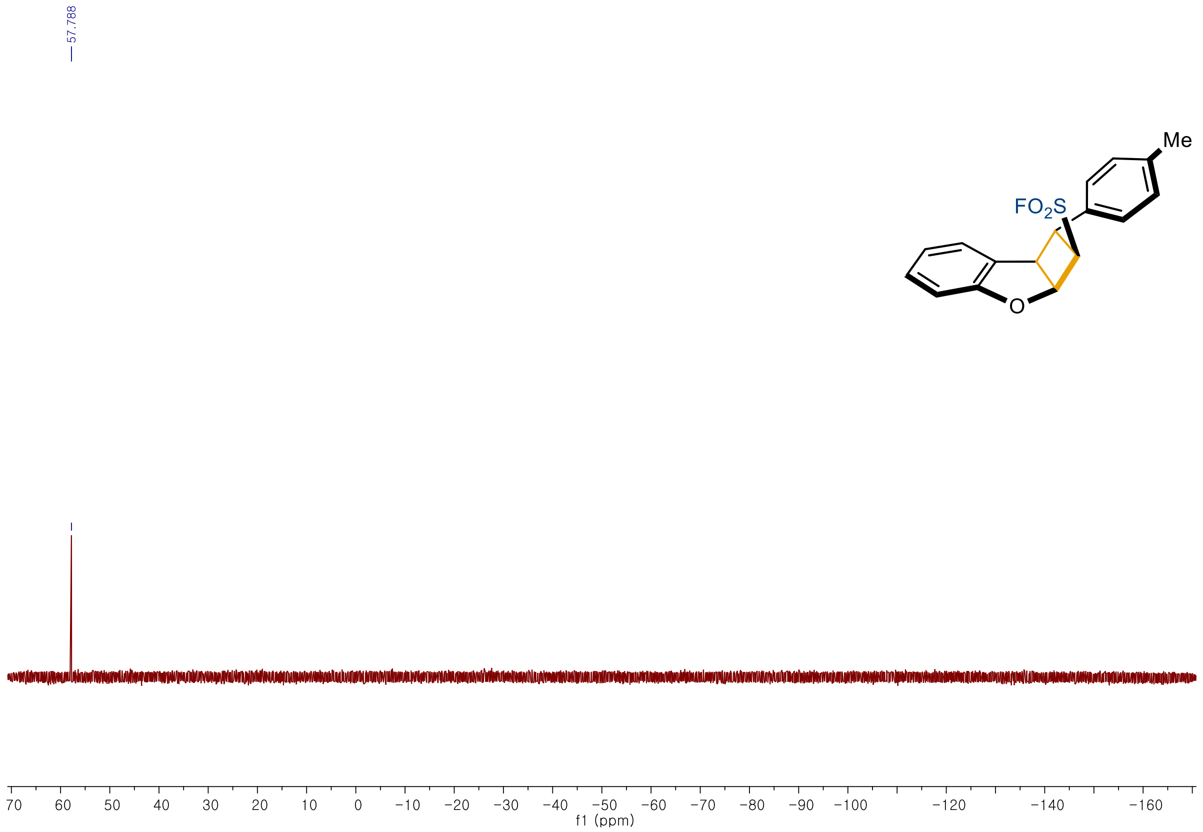

^{19}F NMR spectrum (**3da**-*endo*)

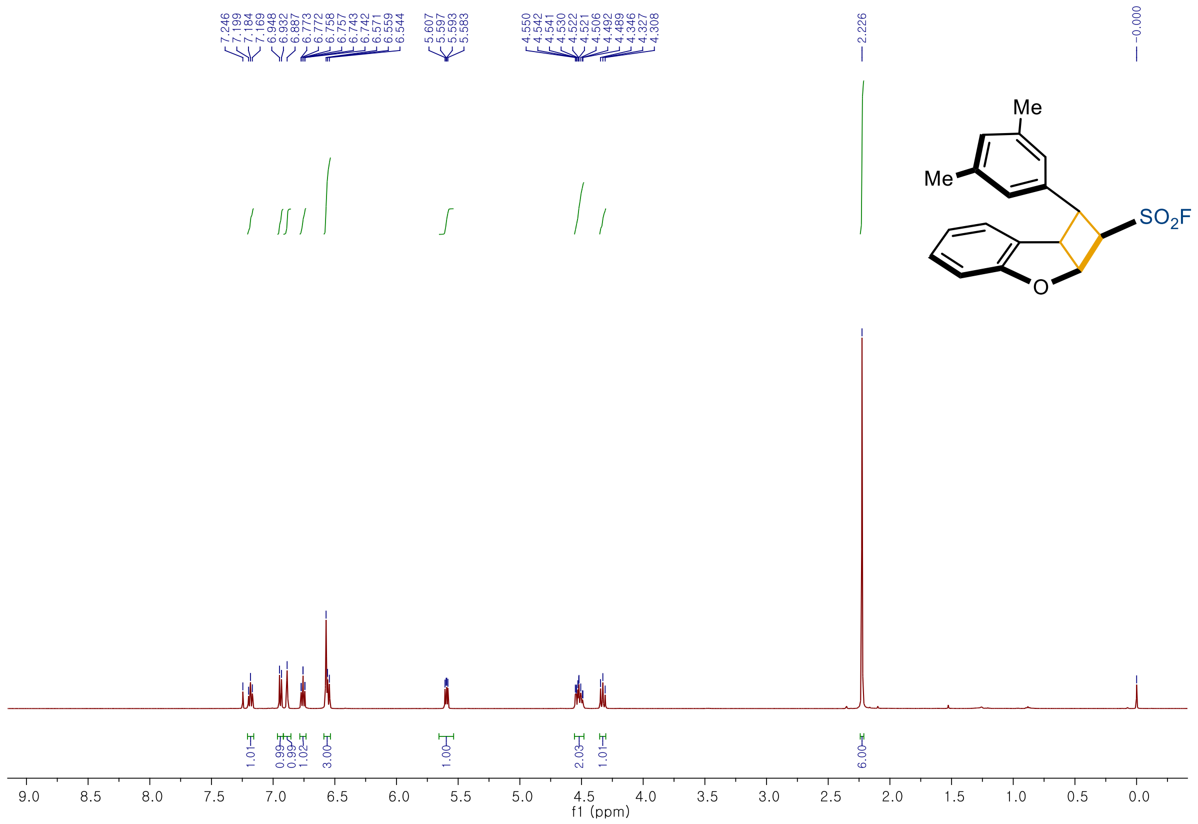

^{1}H NMR spectrum (**3ea**-*exo*)

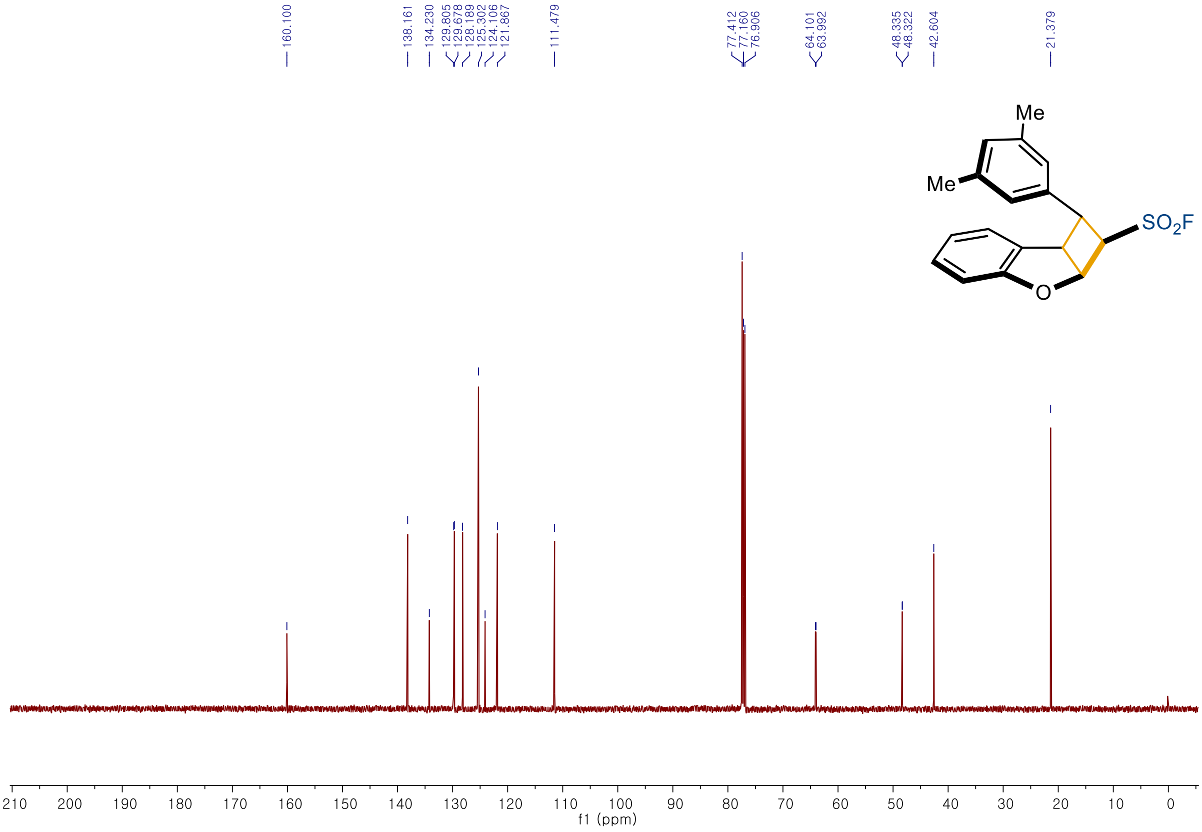

^{13}C NMR spectrum (**3ea**-*exo*)

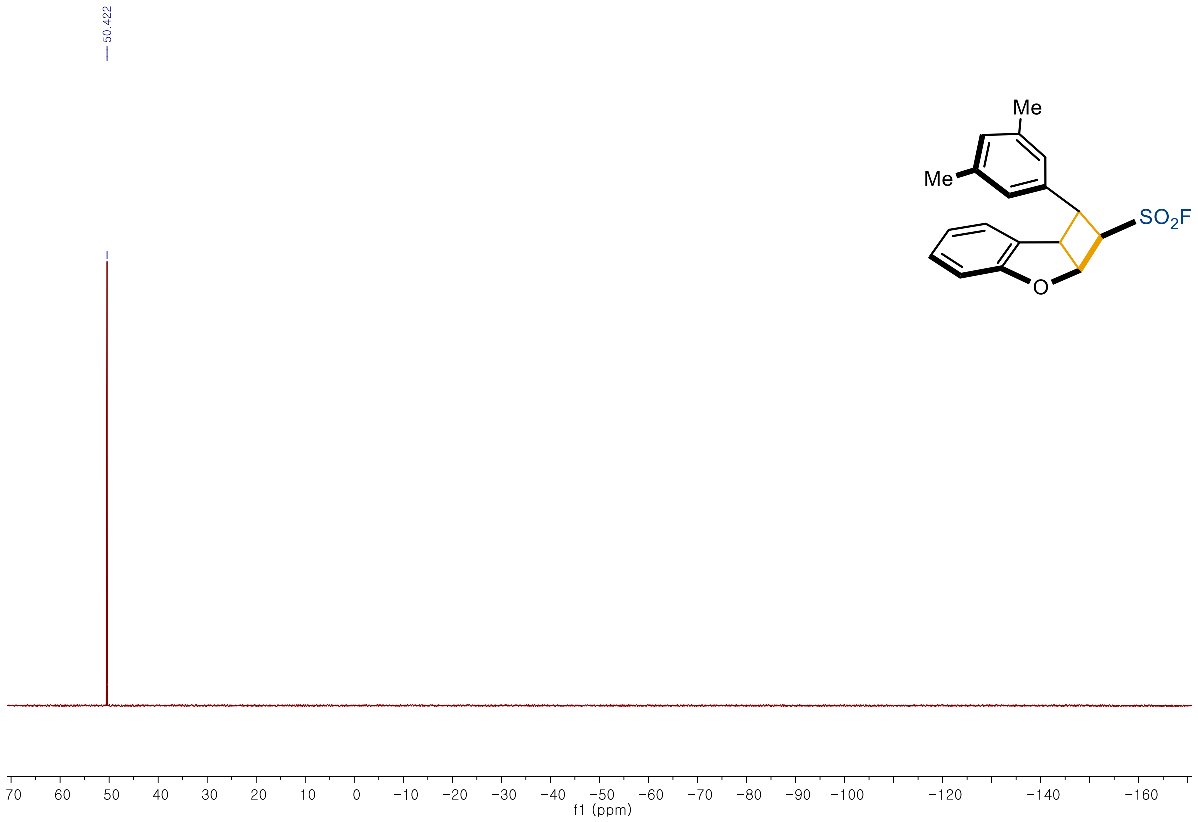

^{19}F NMR spectrum (**3ea**-*exo*)

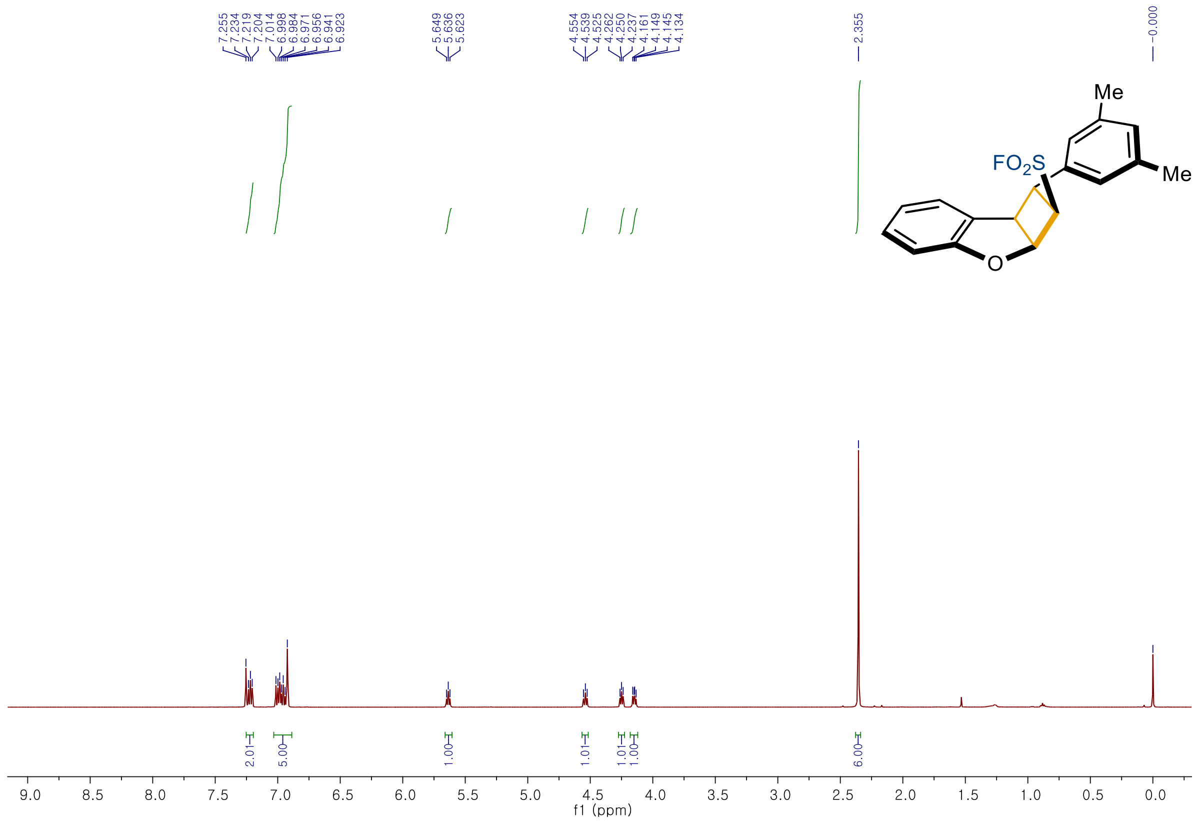

^{1}H NMR spectrum (**3ea**-*endo*)

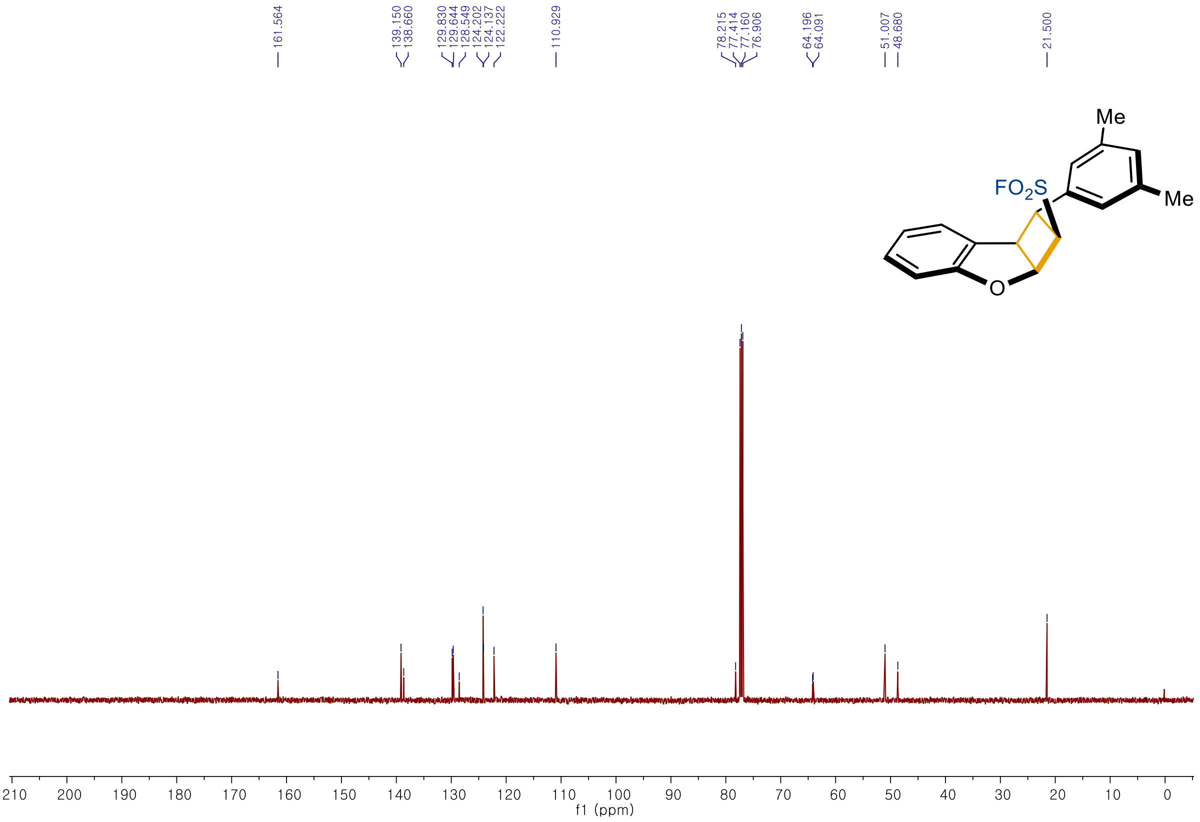

^{13}C NMR spectrum (**3ea**-*endo*)

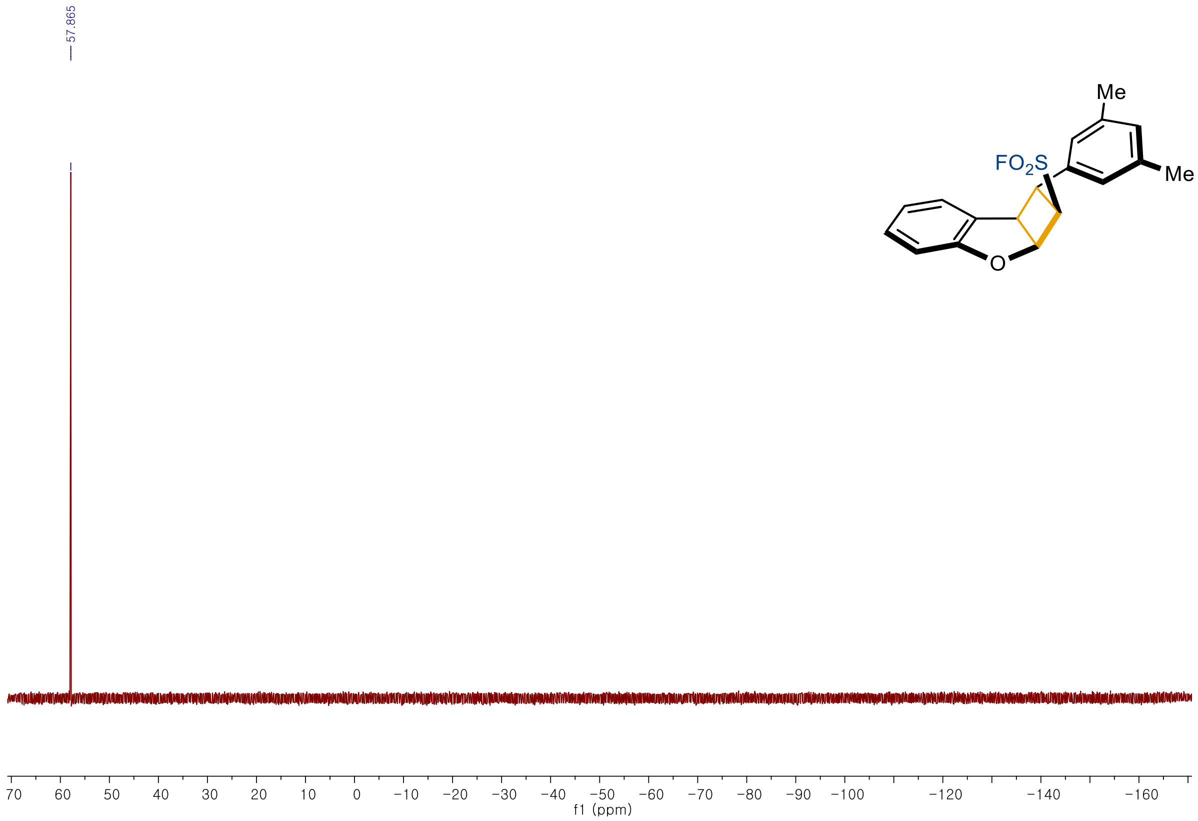

^{19}F NMR spectrum (**3ea**-*endo*)

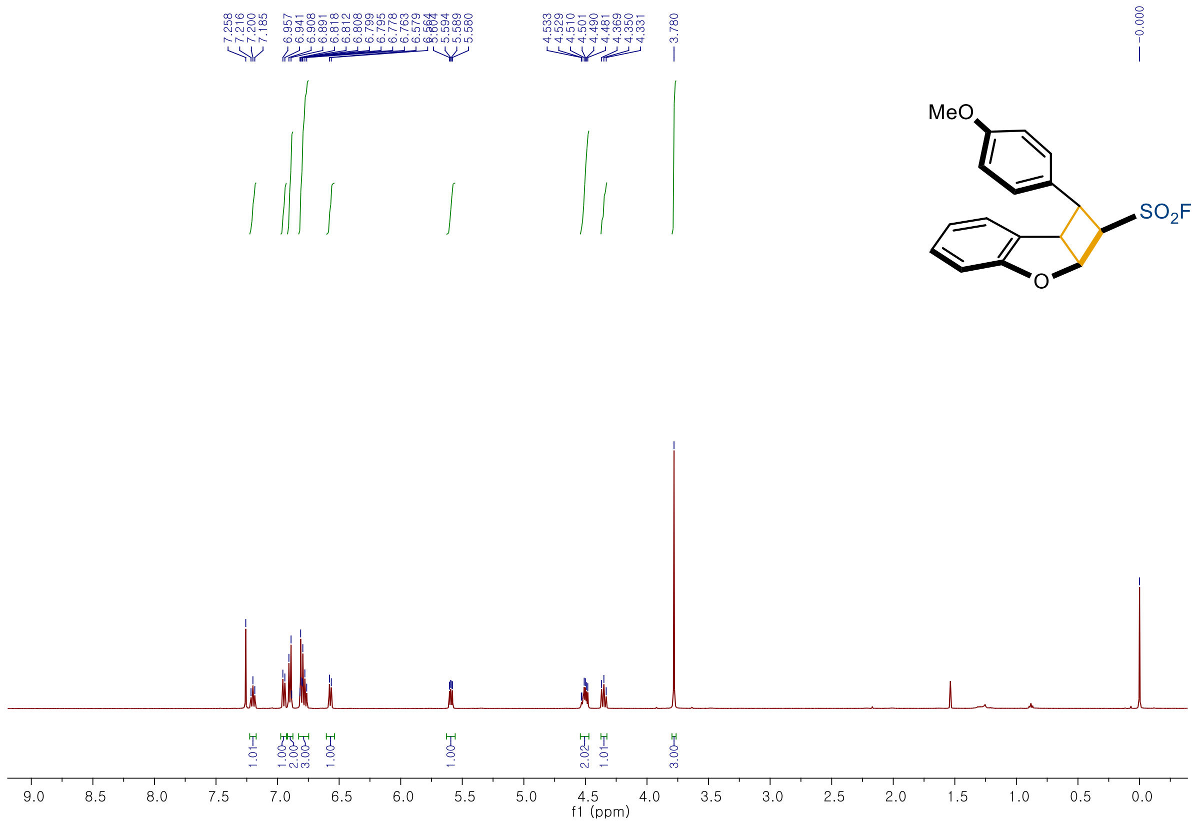

^{1}H NMR spectrum (**3fa**-*exo*)

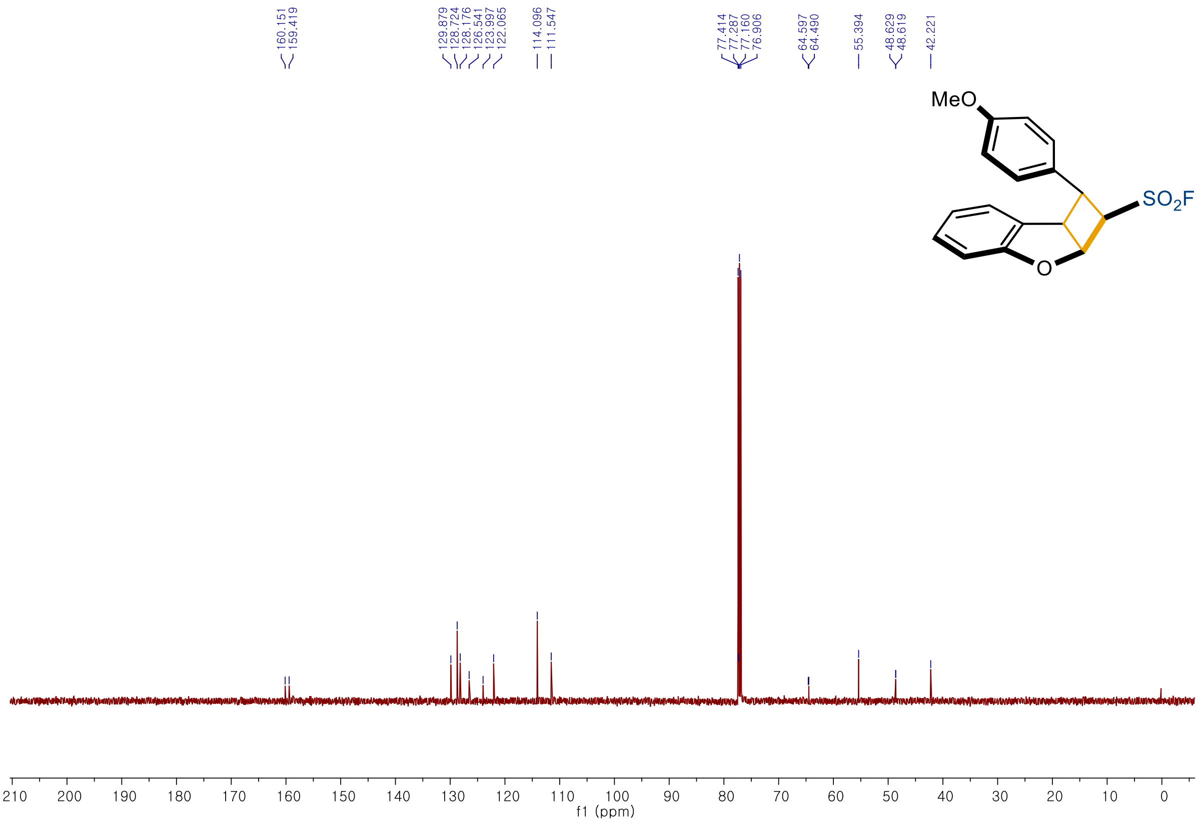

^{13}C NMR spectrum (**3fa**-*exo*)

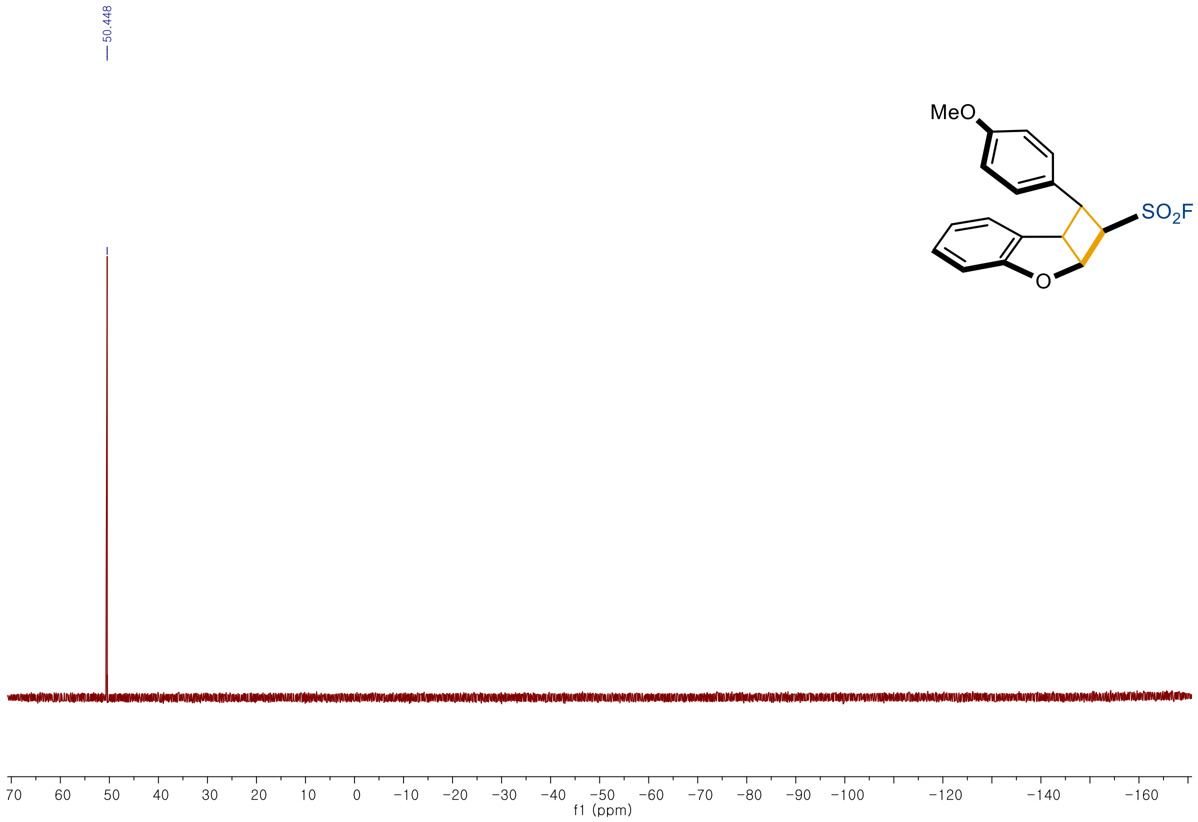

^{19}F NMR spectrum (**3fa**-*exo*)

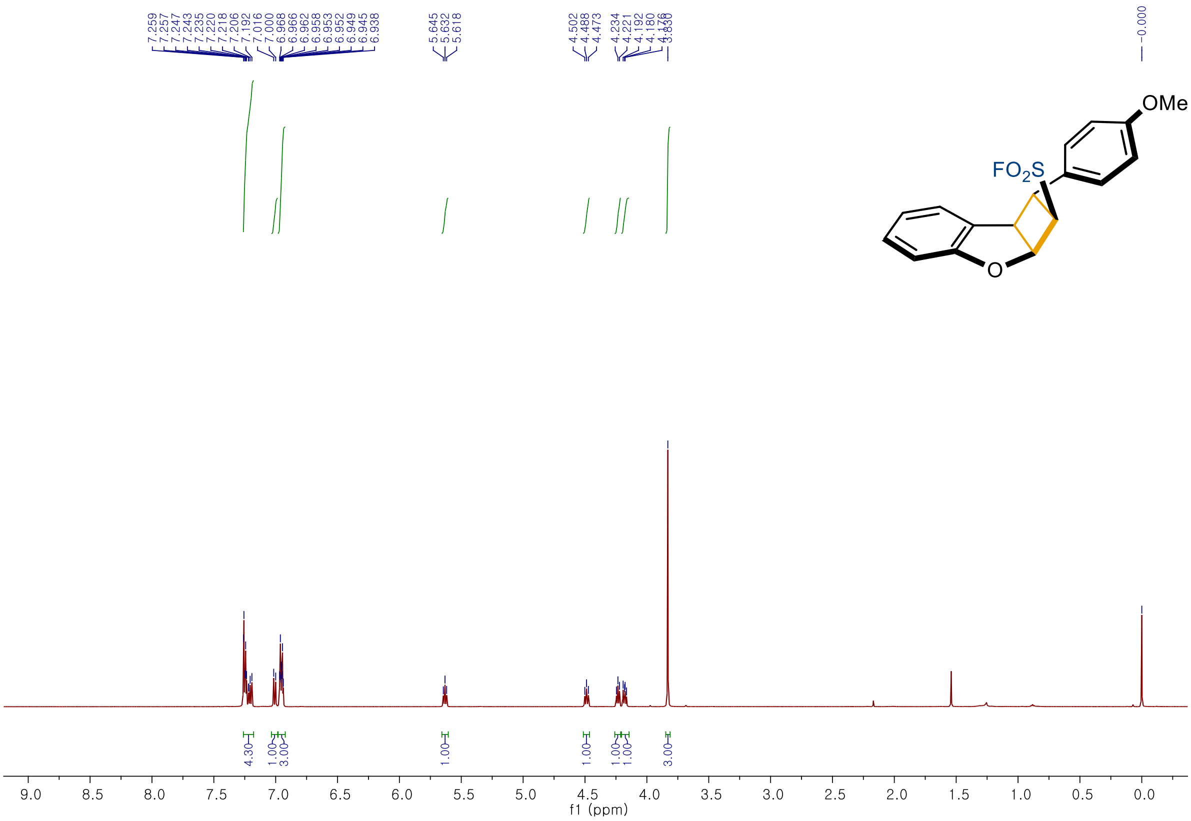

^{1}H NMR spectrum (**3fa**-*endo*)

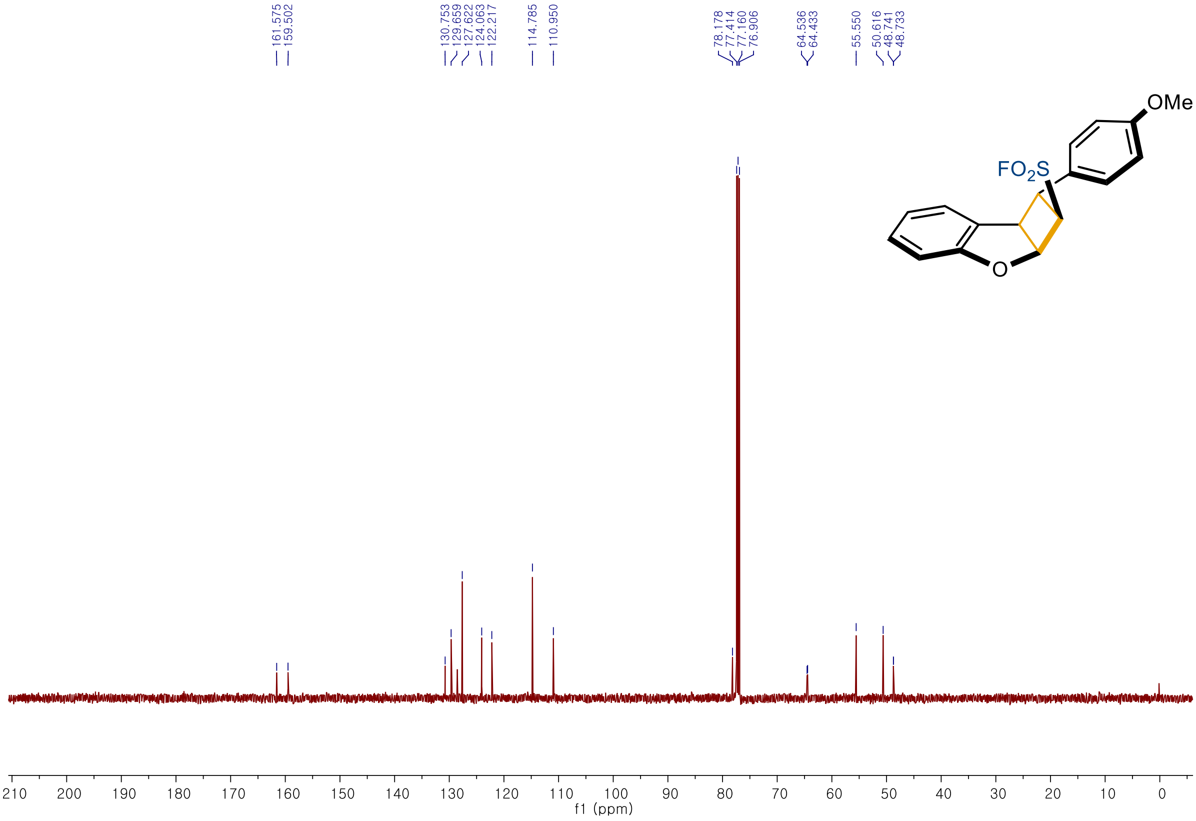

^{13}C NMR spectrum (**3fa**-*endo*)

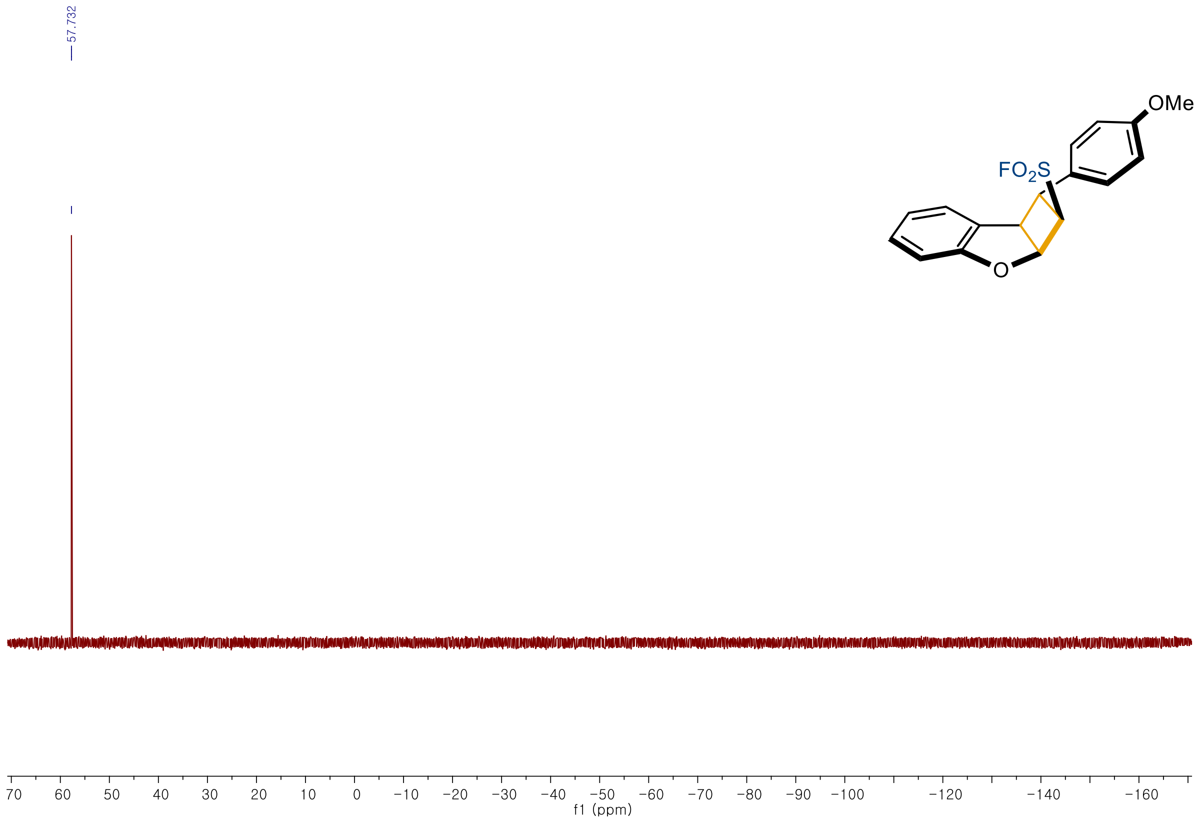

^{19}F NMR spectrum (**3fa**-*endo*)

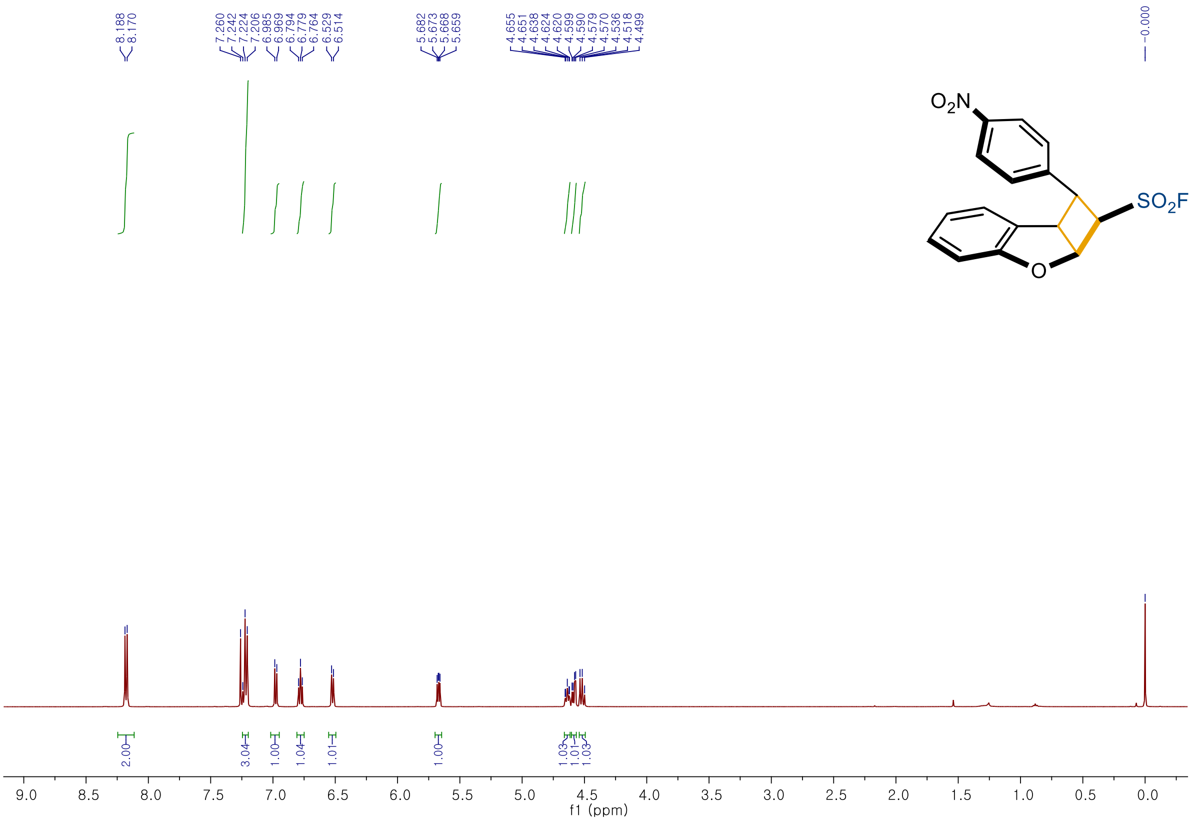

^{1}H NMR spectrum (**3ga**-*exo*)

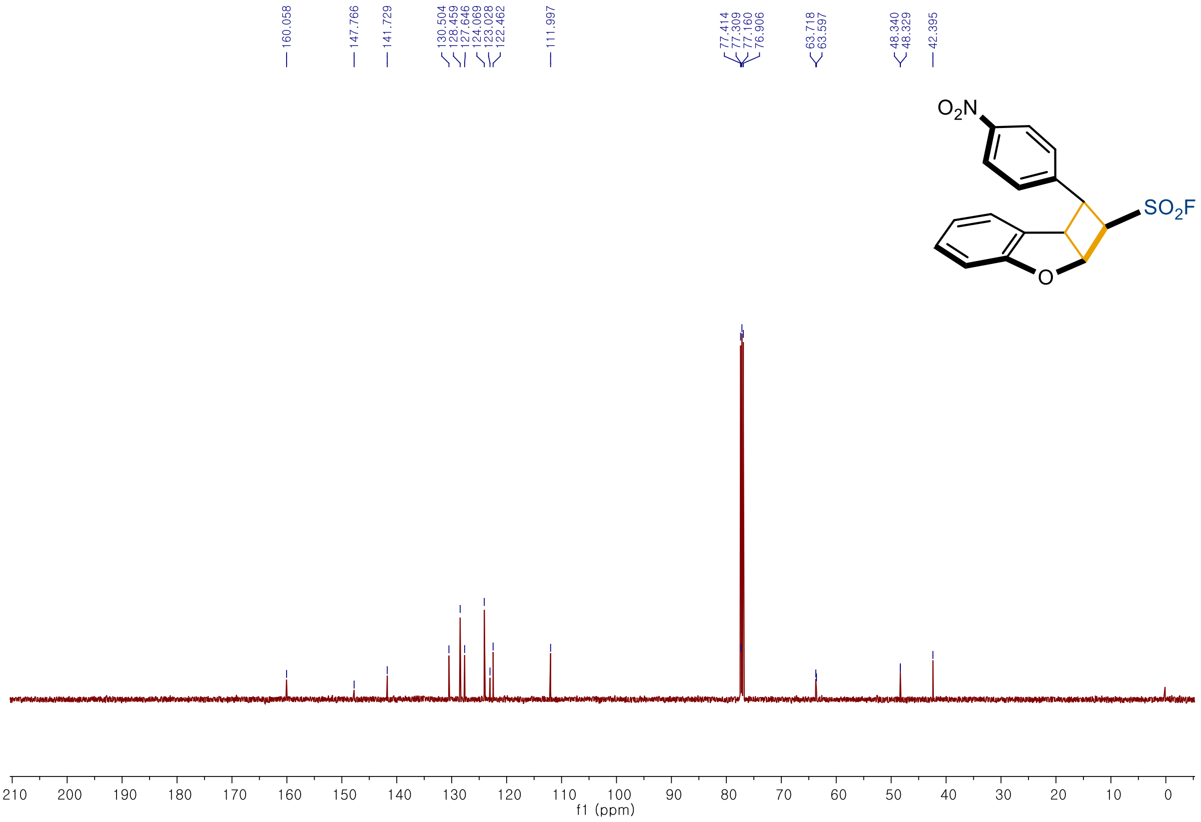

^{13}C NMR spectrum (**3ga**-*exo*)

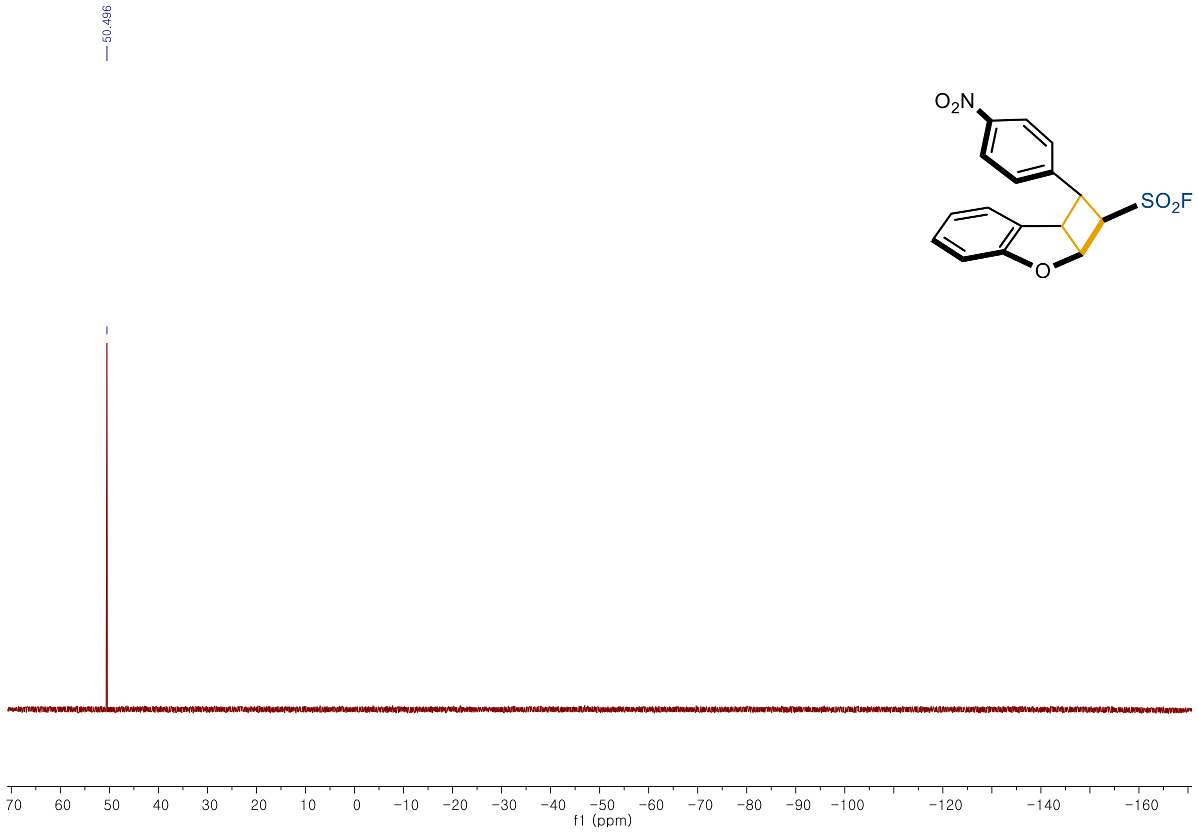

^{19}F NMR spectrum (**3ga**-*exo*)

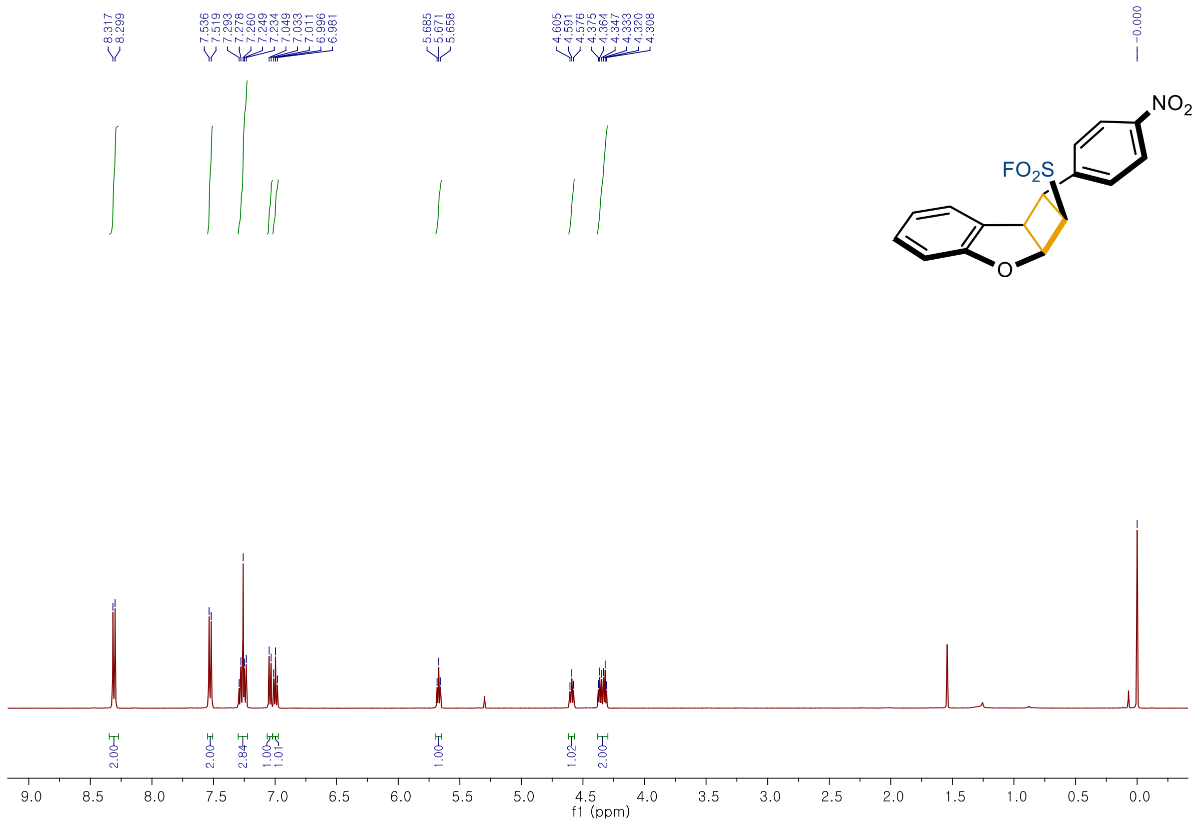

^{1}H NMR spectrum (**3ga**-*endo*)

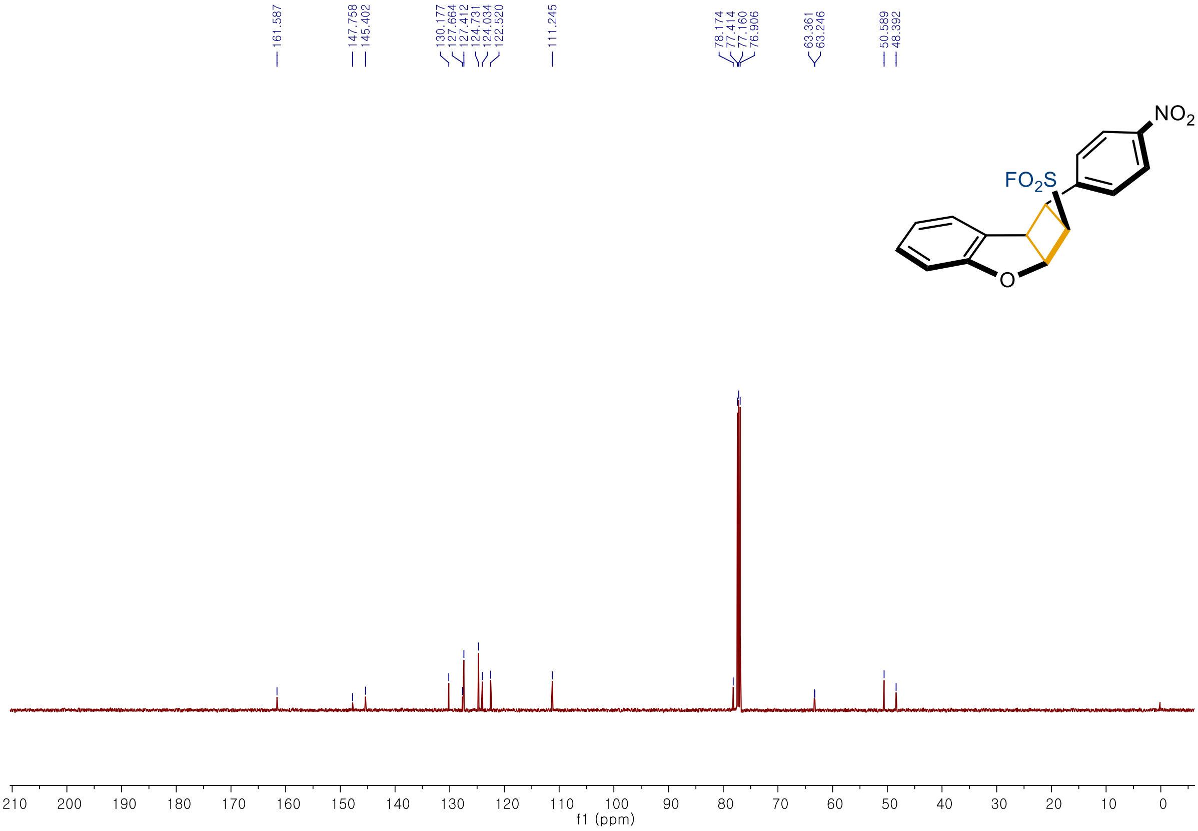

^{13}C NMR spectrum (**3ga**-*endo*)

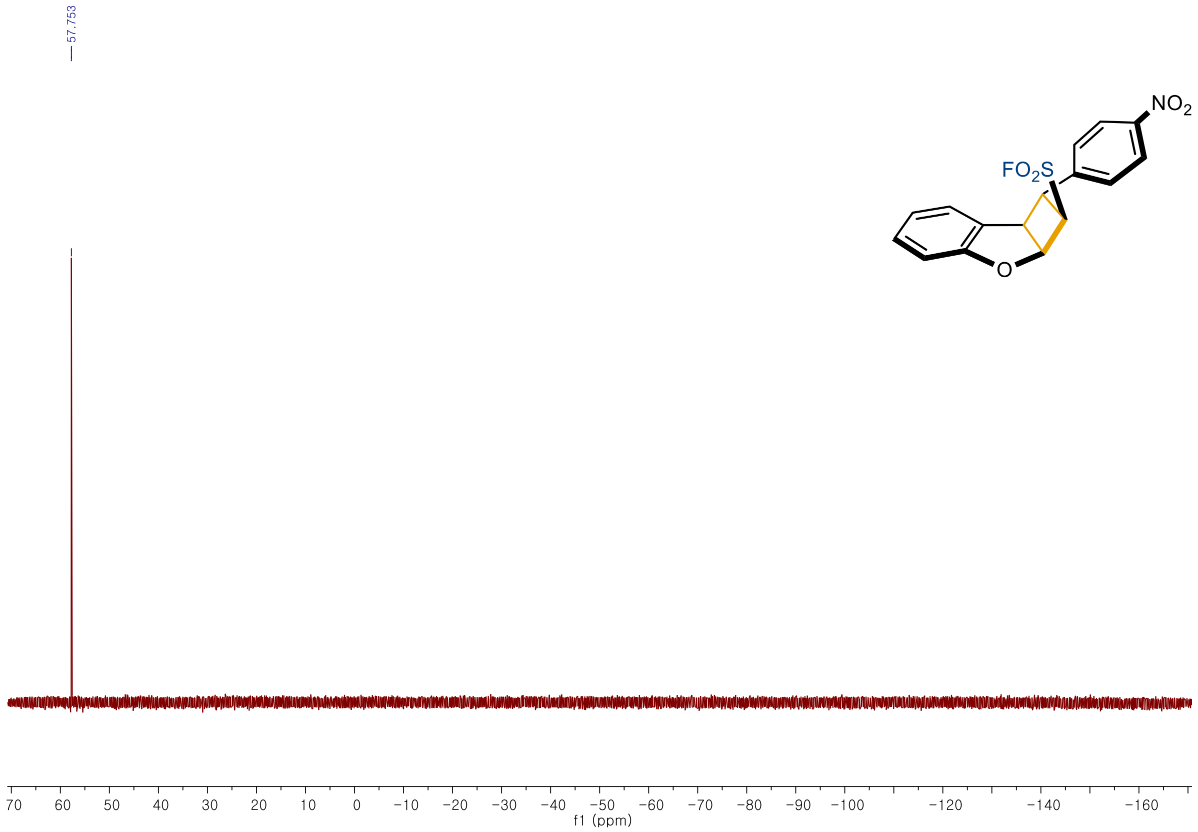

^{19}F NMR spectrum (**3ga**-*endo*)

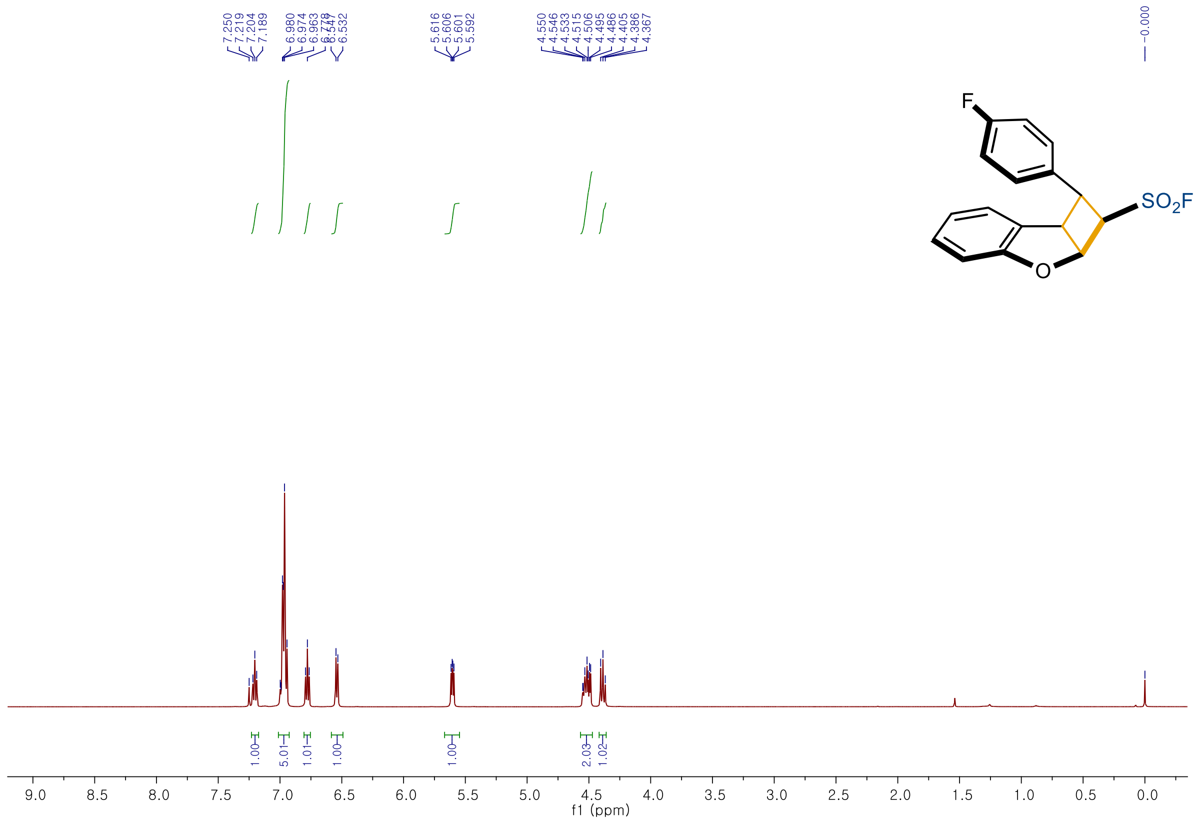

^{1}H NMR spectrum (**3ha**-*exo*)

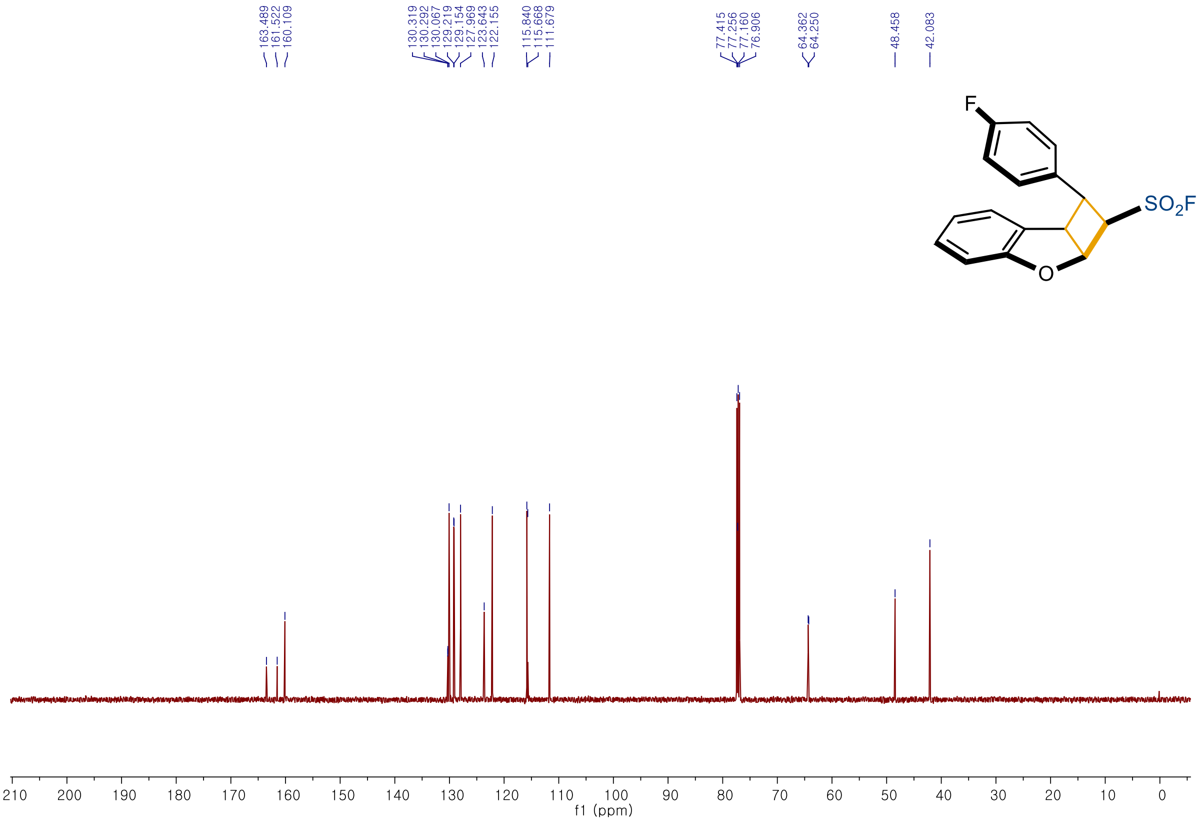

^{13}C NMR spectrum (**3ha**-*exo*)

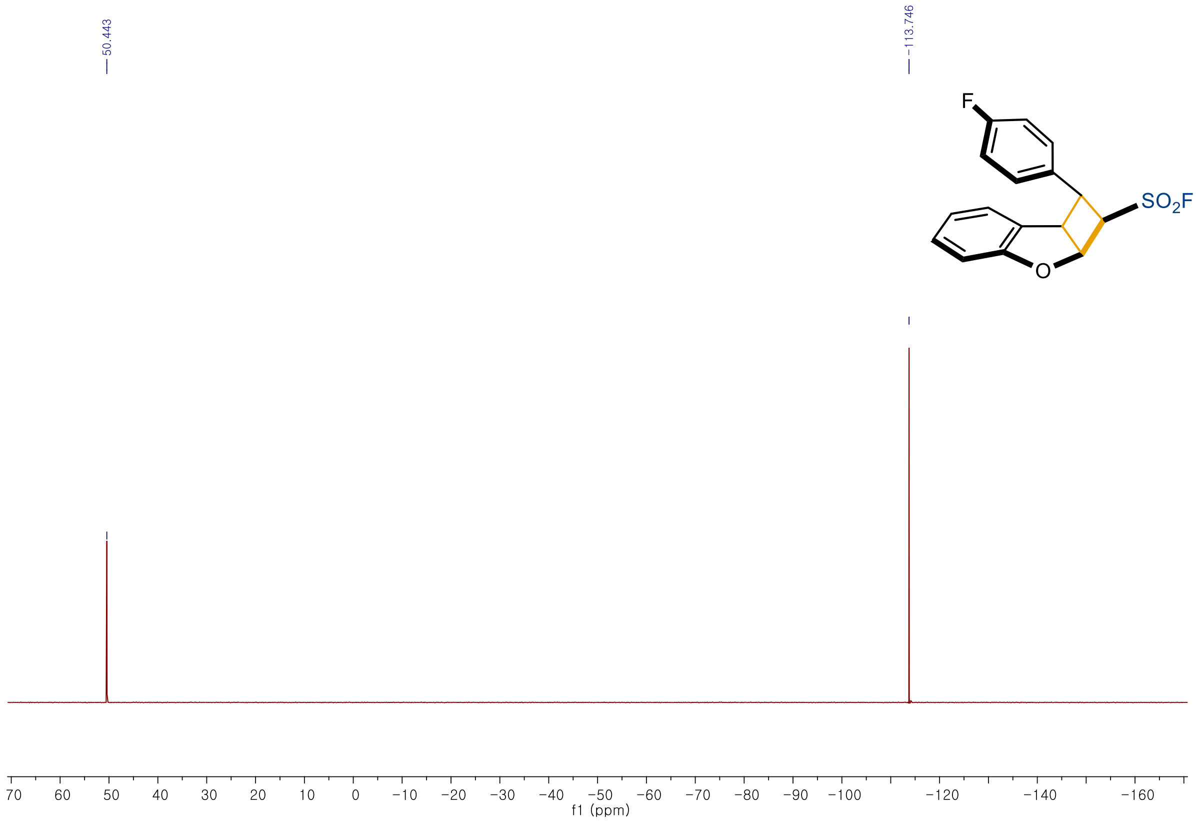

^{19}F NMR spectrum (**3ha**-*exo*)

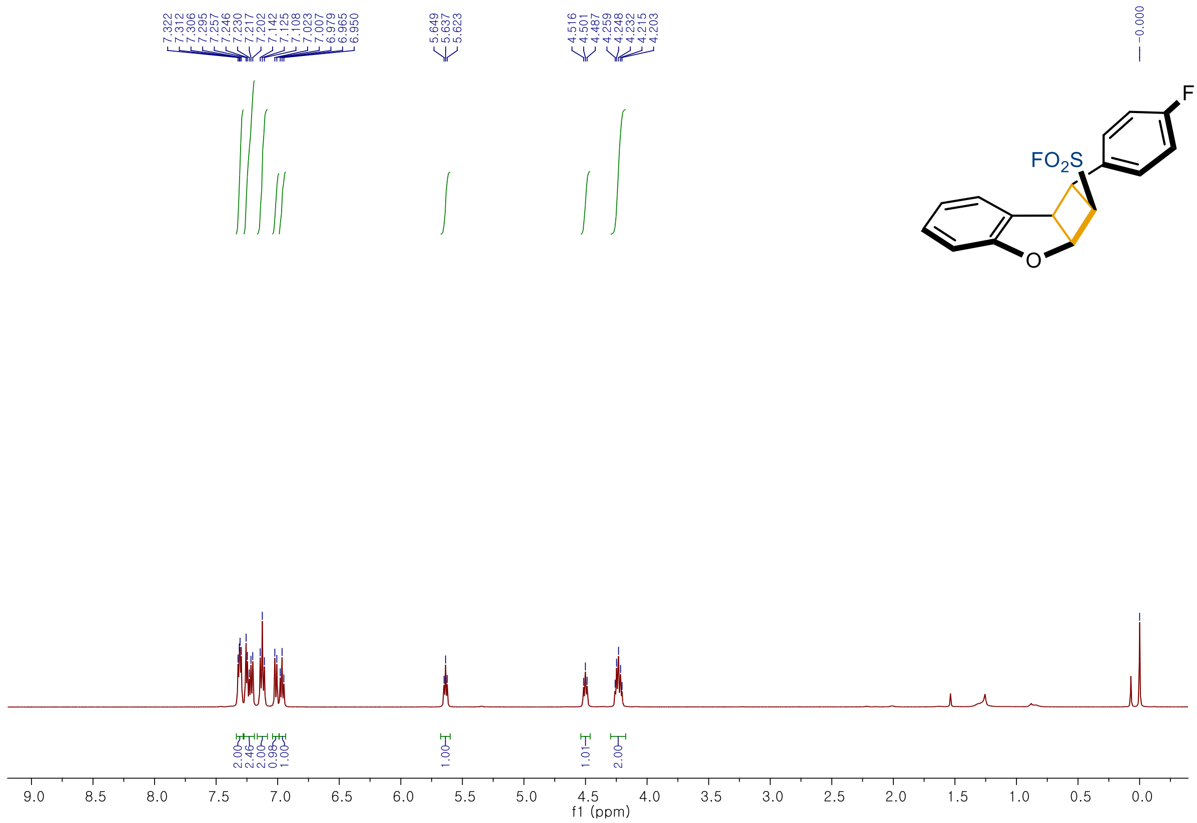

^{1}H NMR spectrum (**3ha**-*endo*)

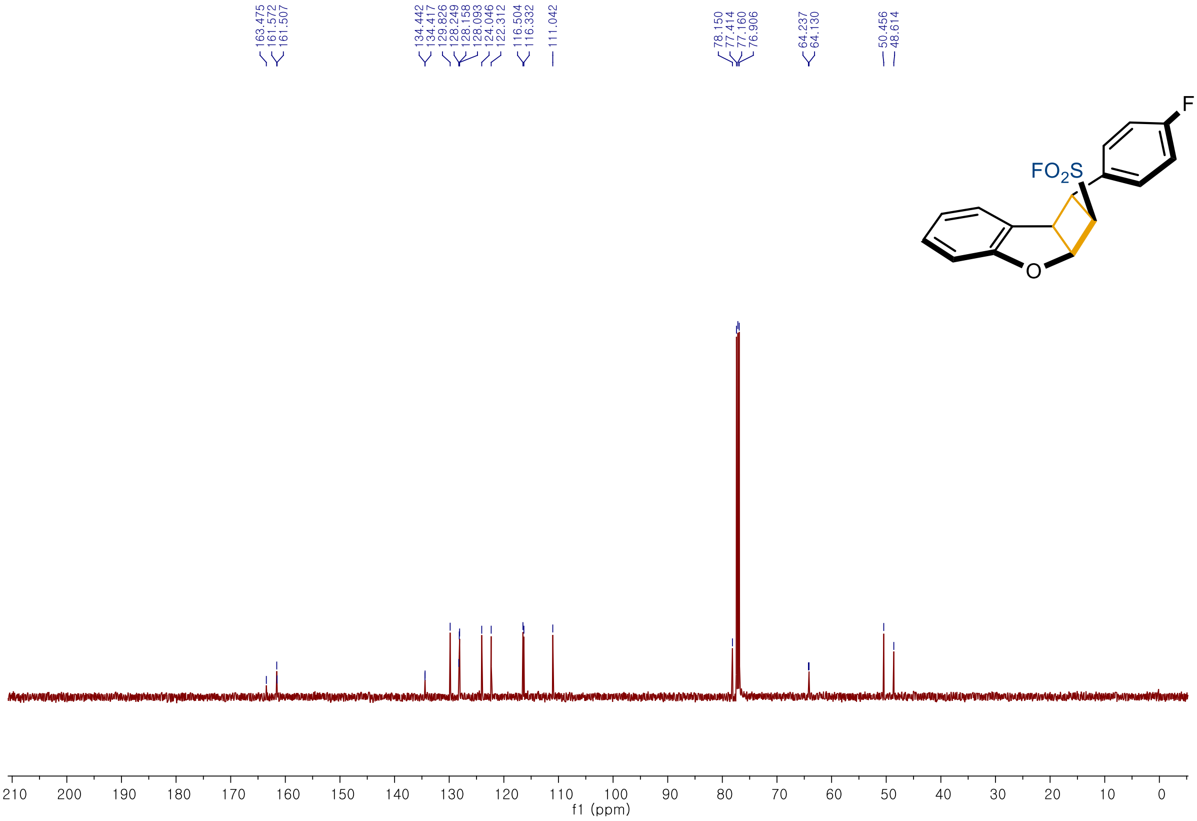

^{13}C NMR spectrum (**3ha**-*endo*)

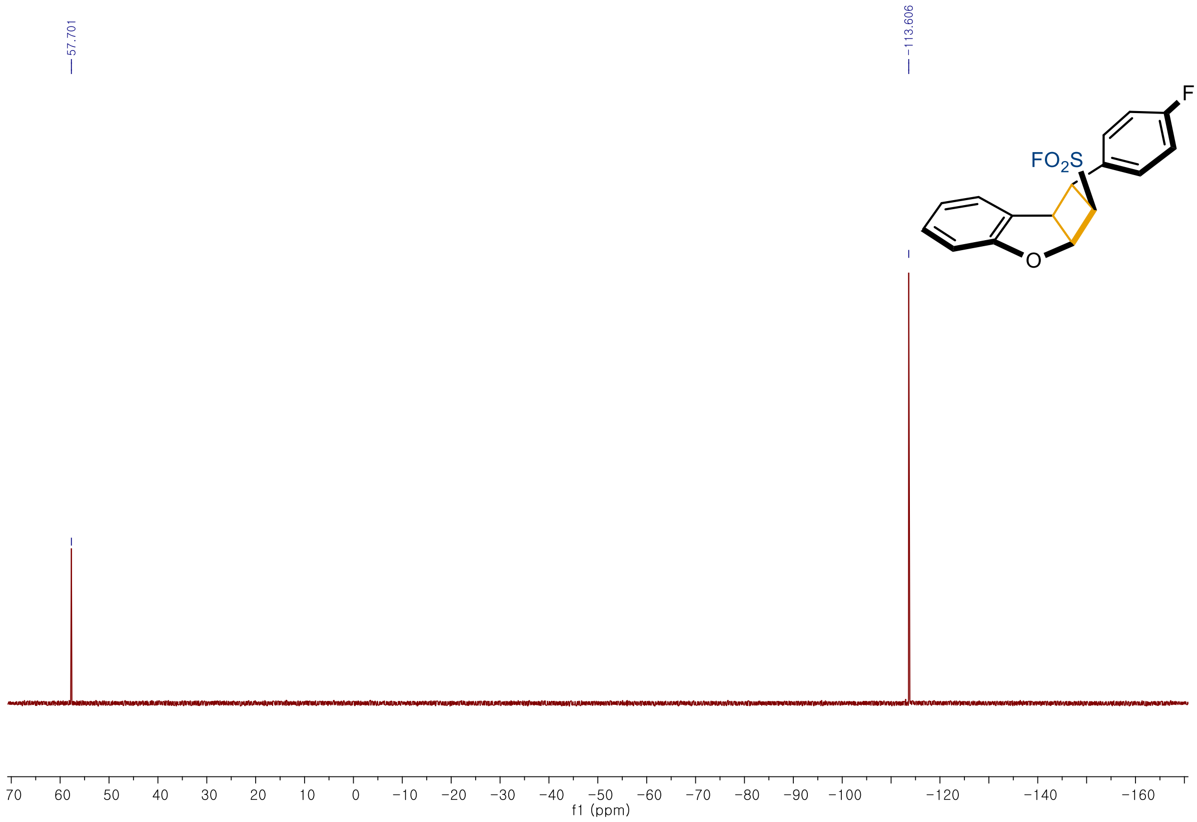

^{19}F NMR spectrum (**3ha**-*endo*)

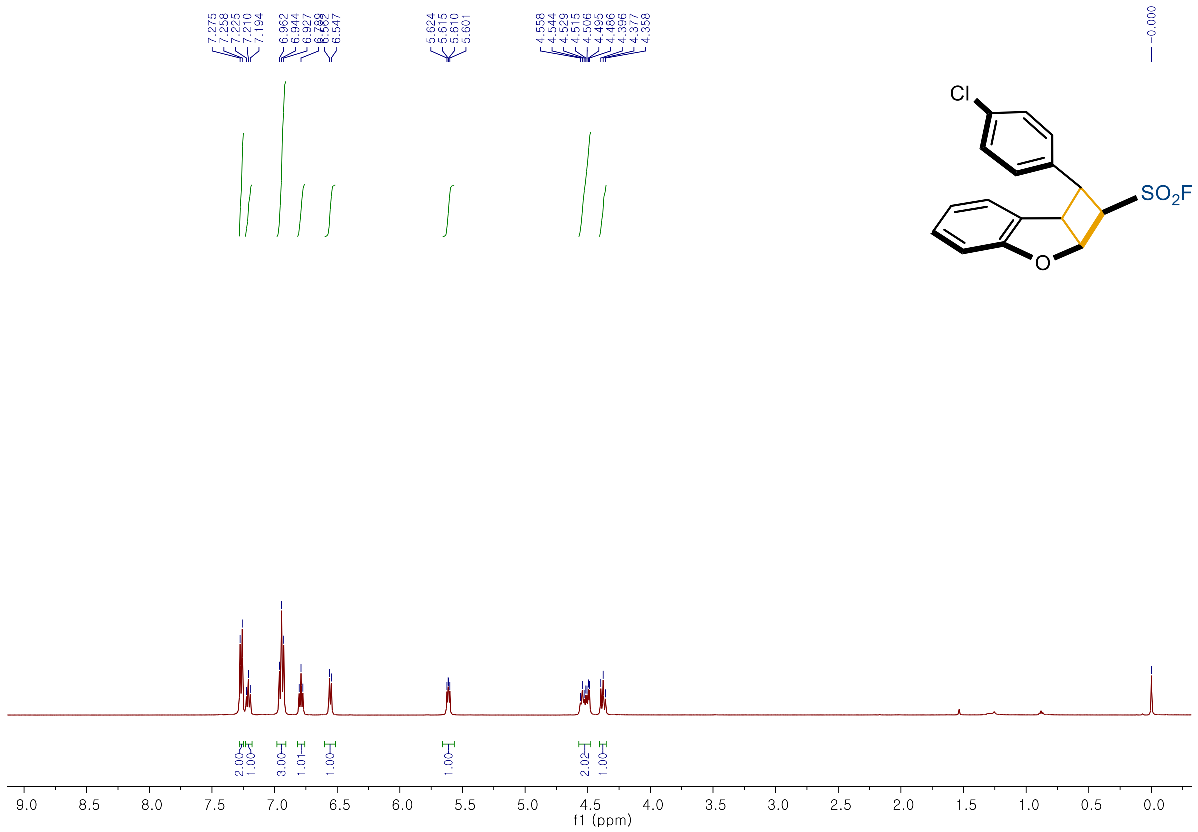

^{1}H NMR spectrum (**3ia**-*exo*)

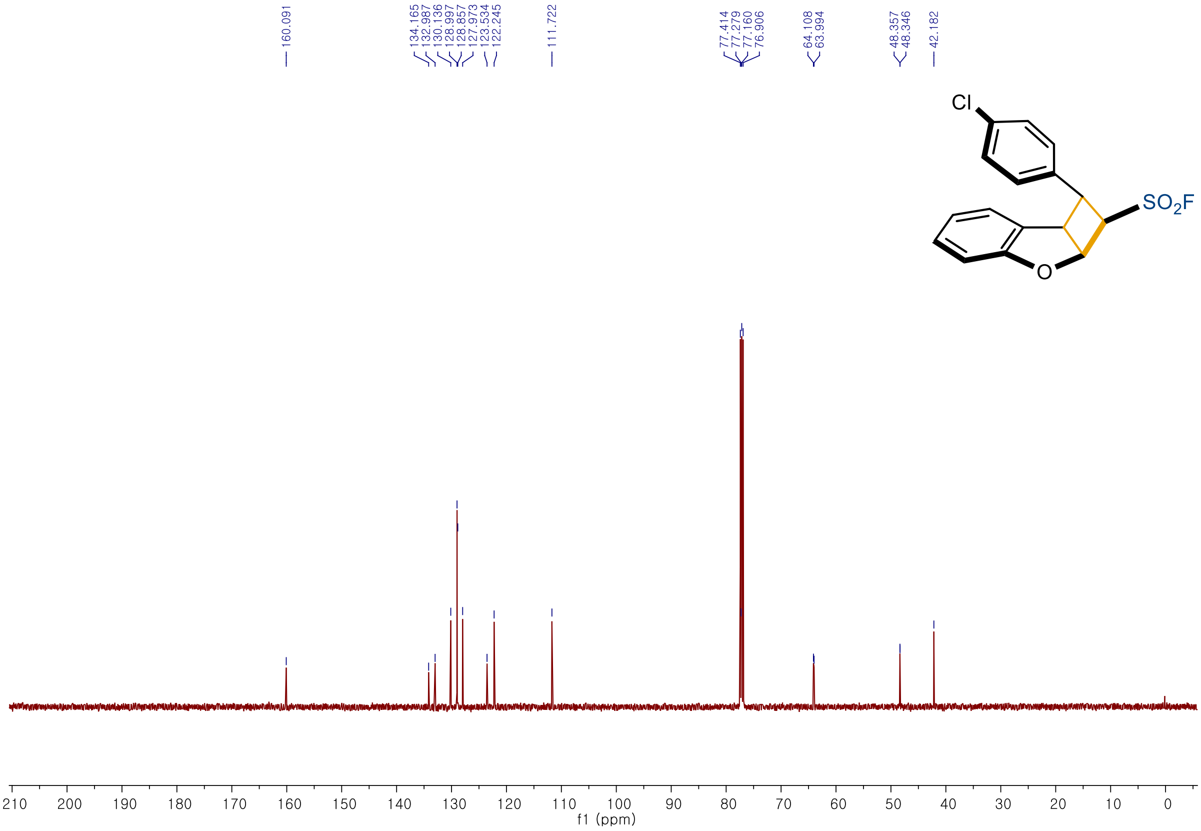

^{13}C NMR spectrum (**3ia**-*exo*)

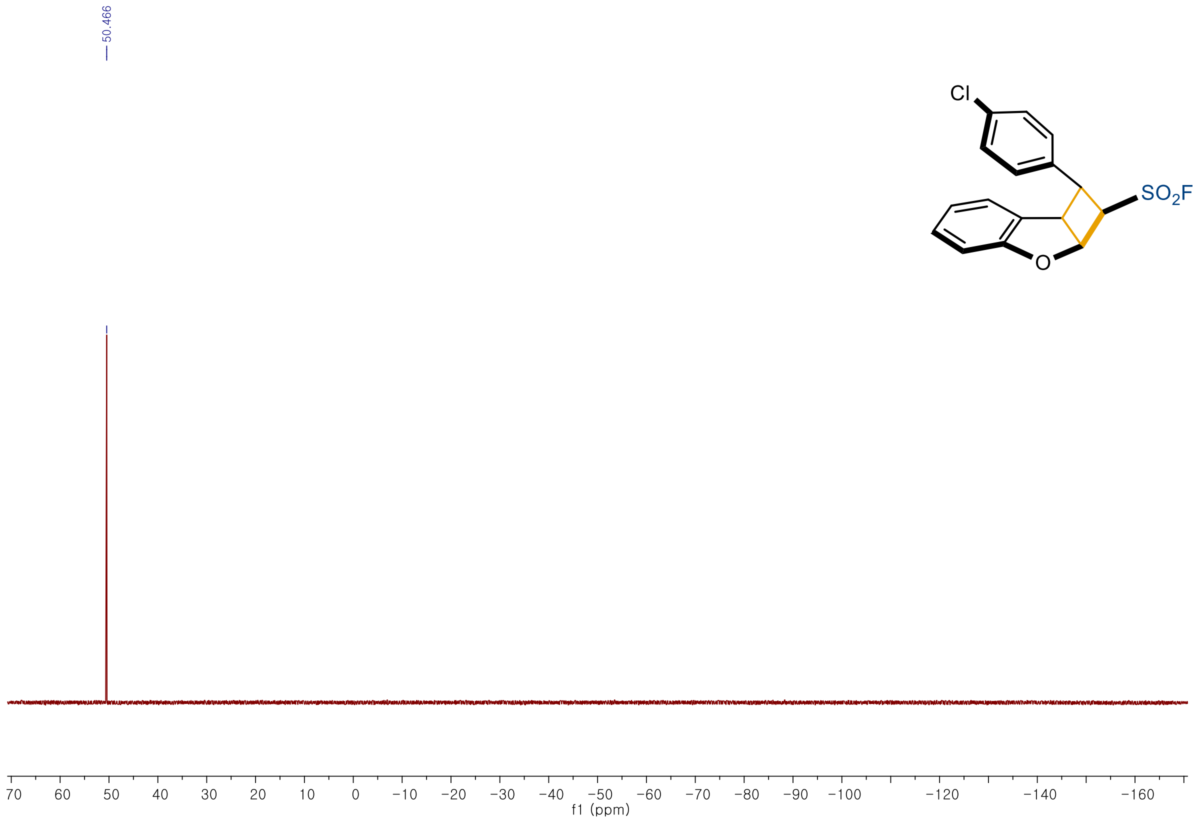

^{19}F NMR spectrum (**3ia**-*exo*)

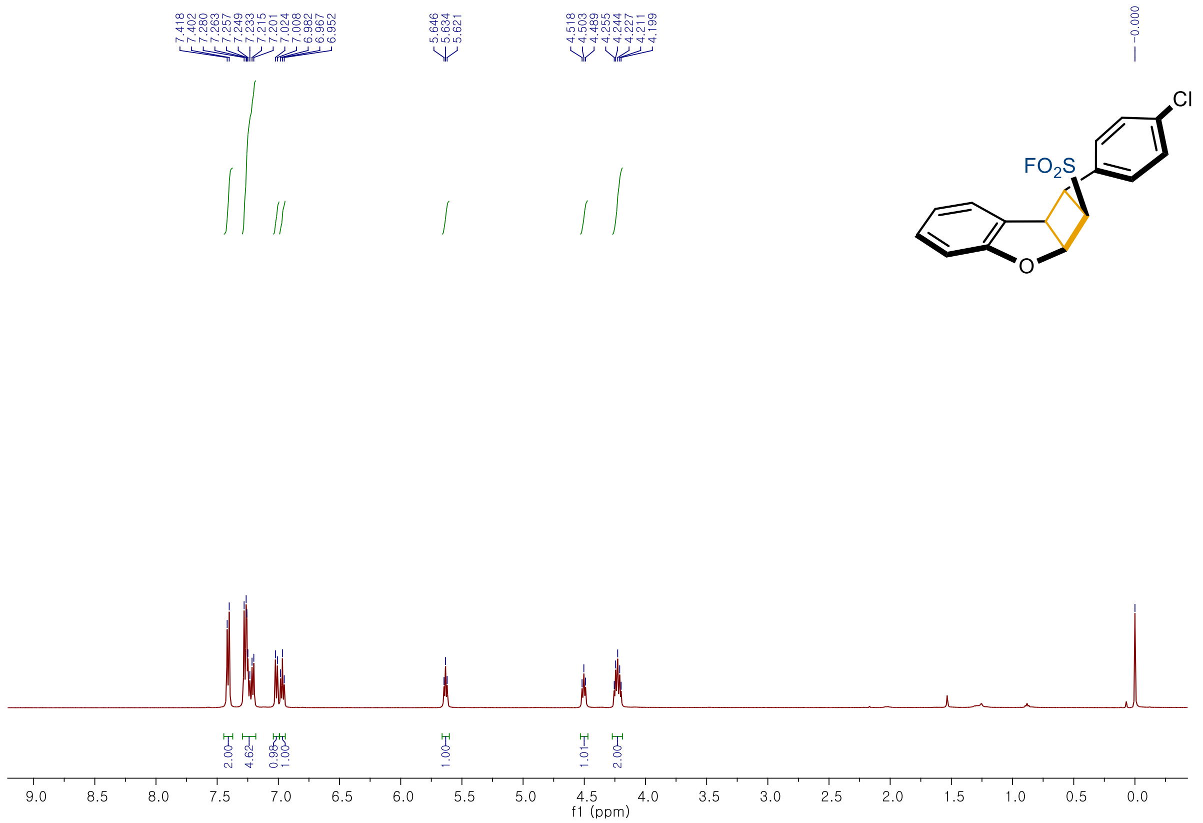

^{1}H NMR spectrum (**3ia**-*endo*)

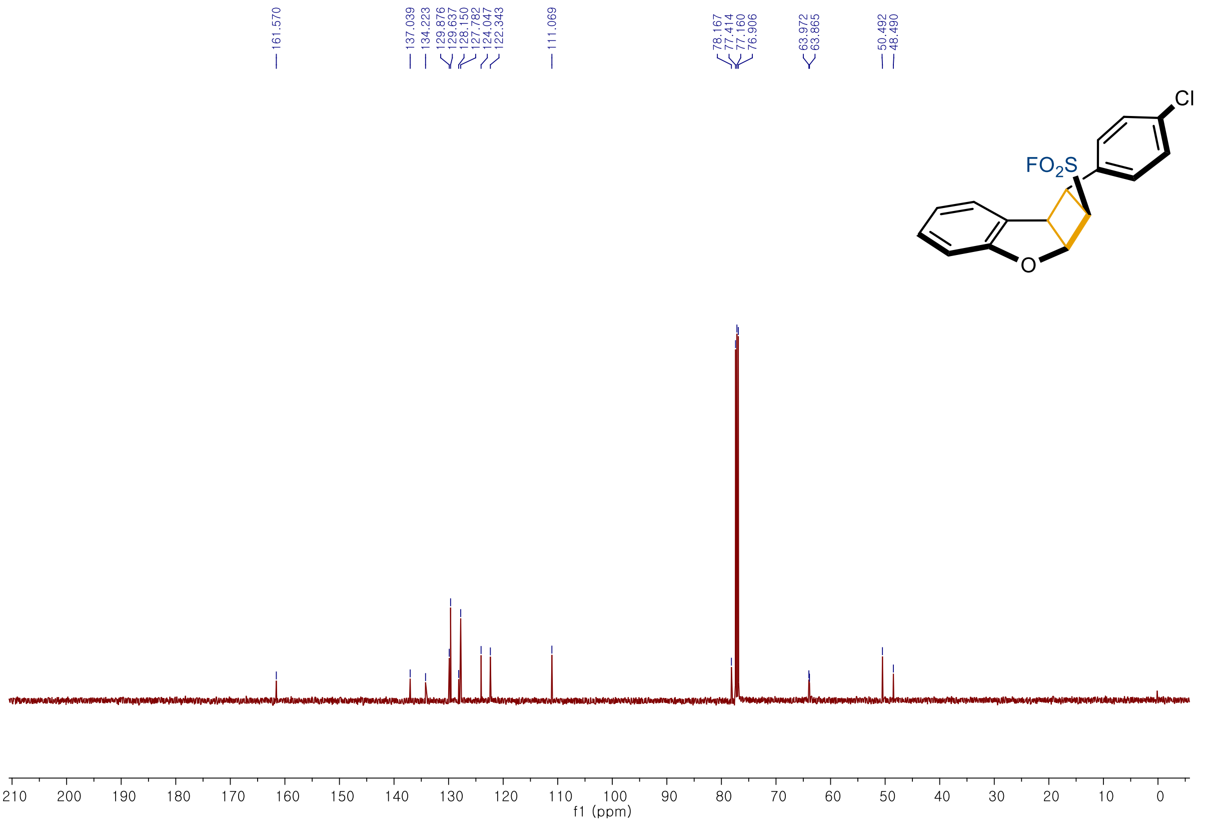

^{13}C NMR spectrum (**3ia**-*endo*)

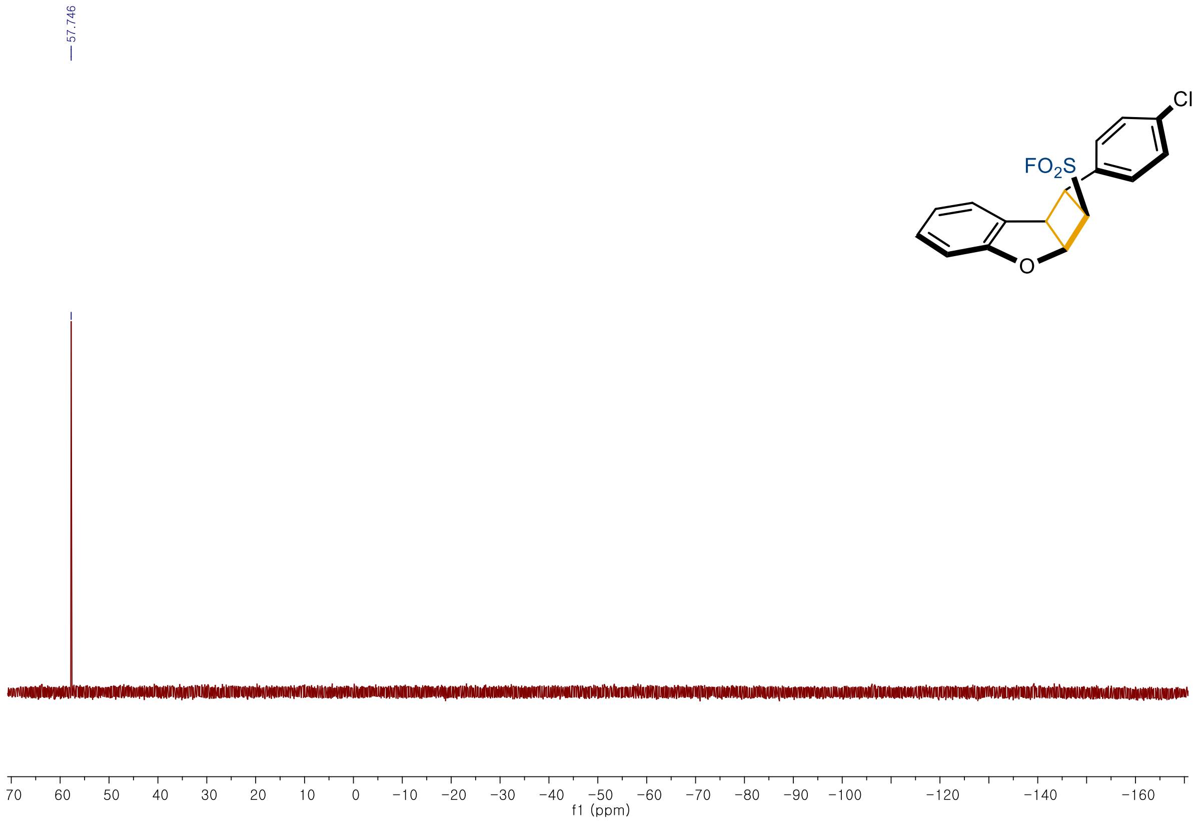

^{19}F NMR spectrum (**3ia**-*endo*)

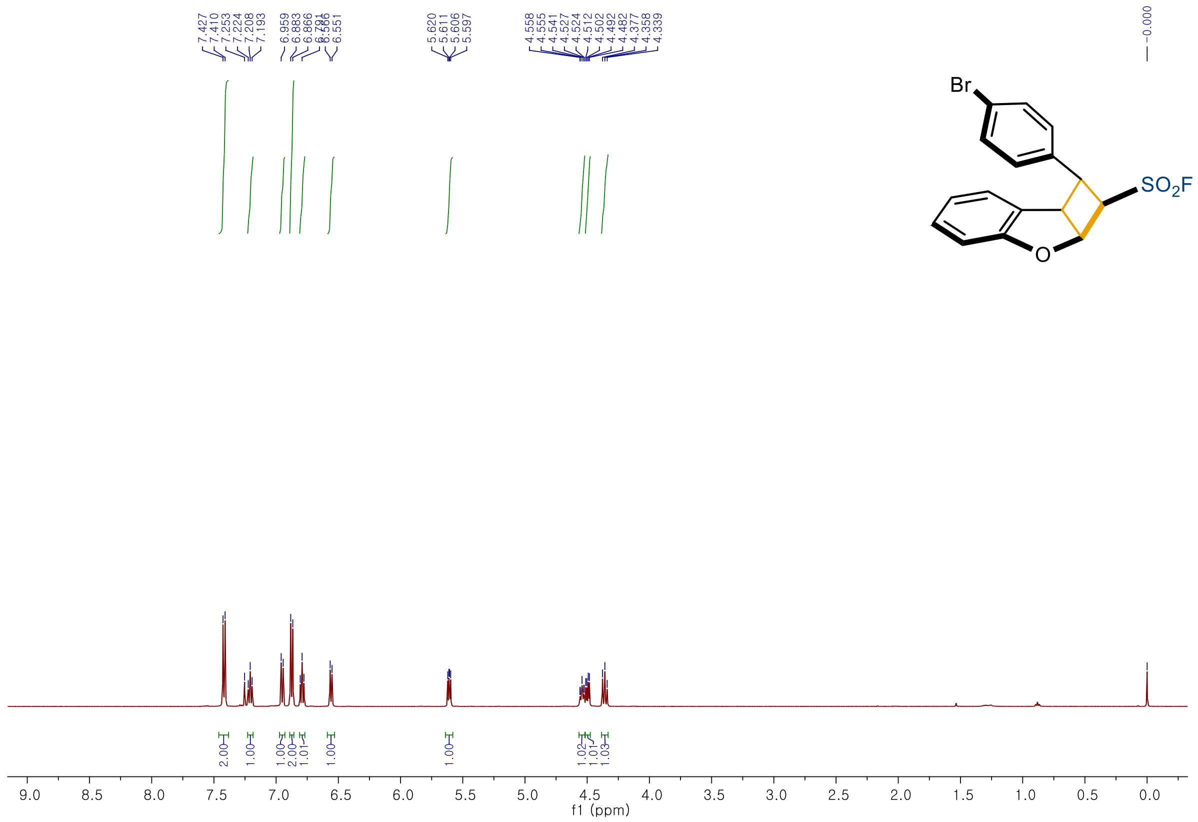

^{1}H NMR spectrum (**3ja**-*exo*)

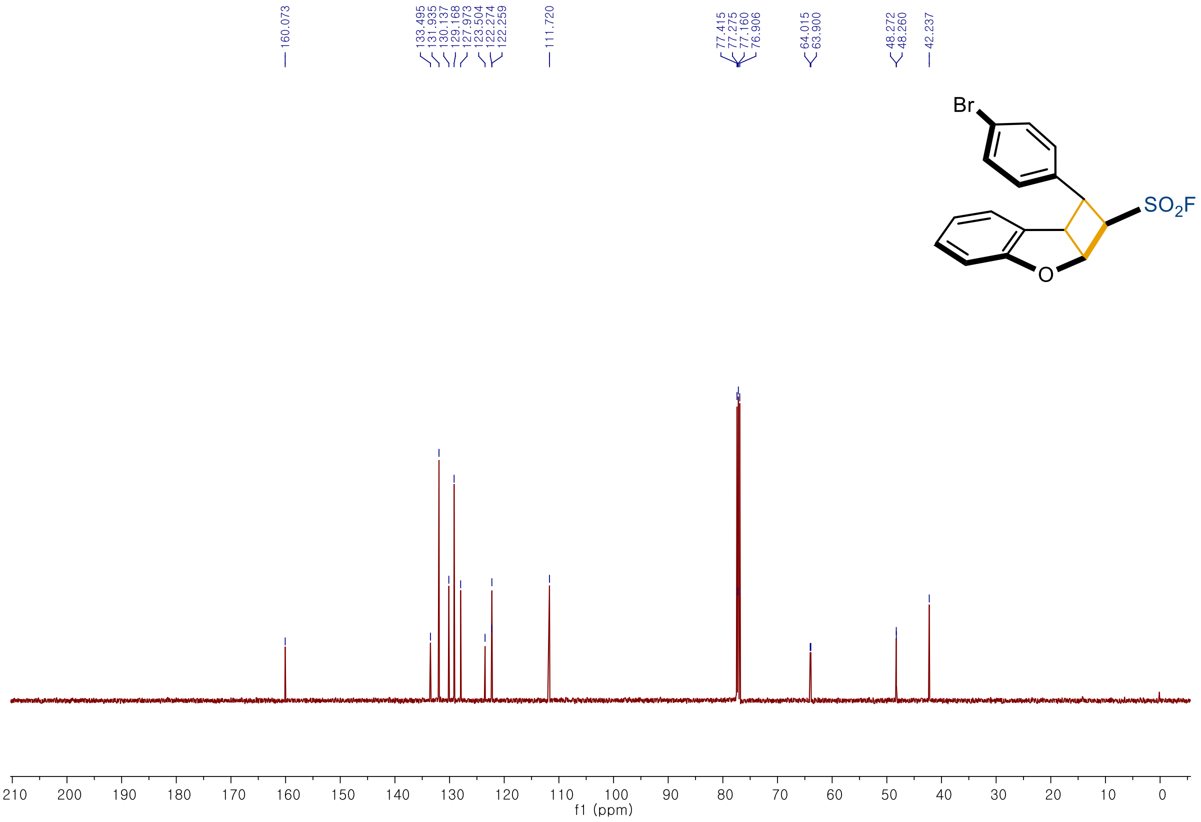

^{13}C NMR spectrum (**3ja**-*exo*)

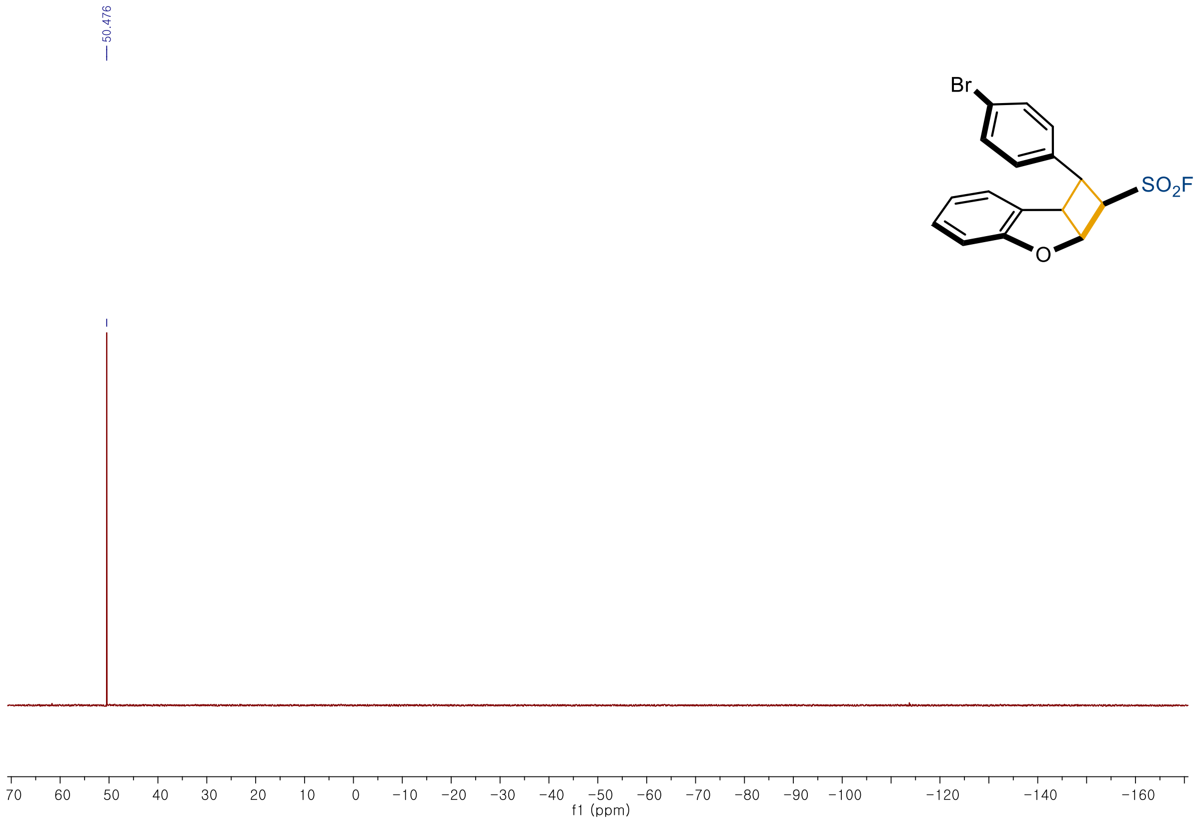

^{19}F NMR spectrum (**3ja**-*exo*)

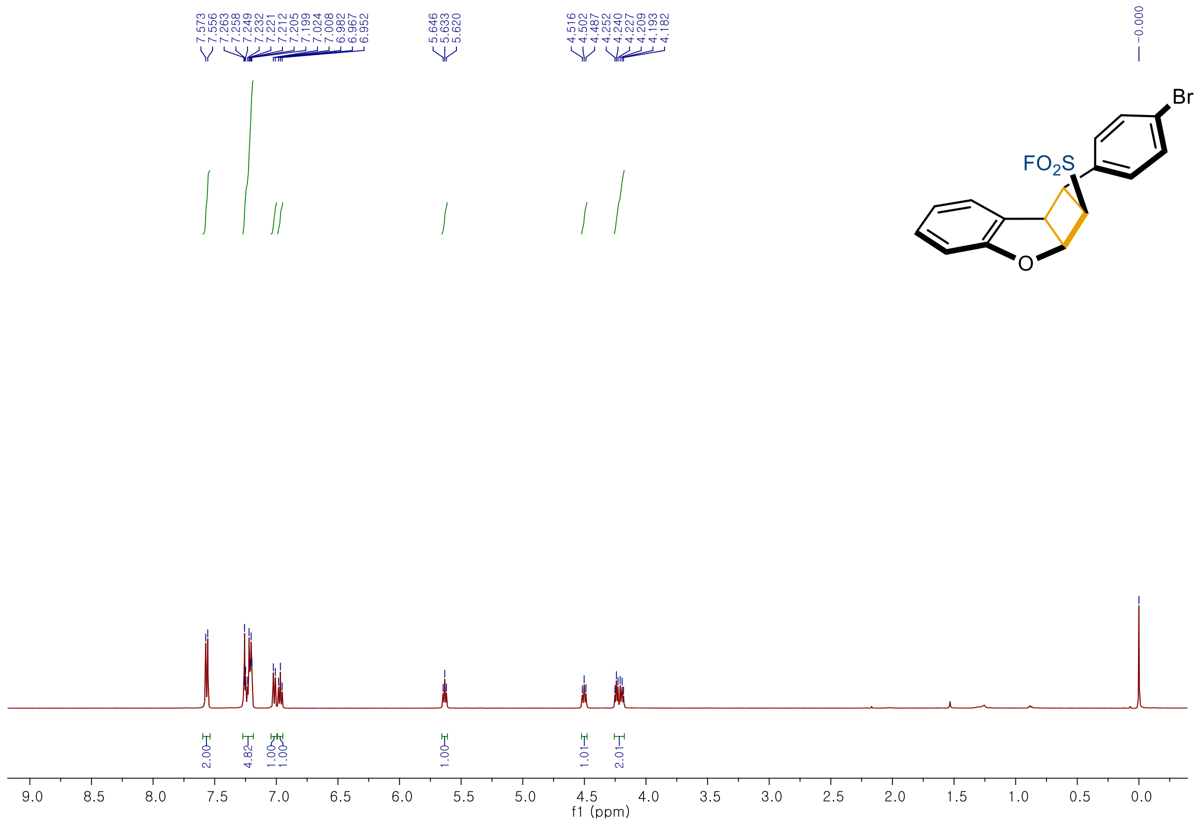

^{1}H NMR spectrum (**3ja**-*endo*)

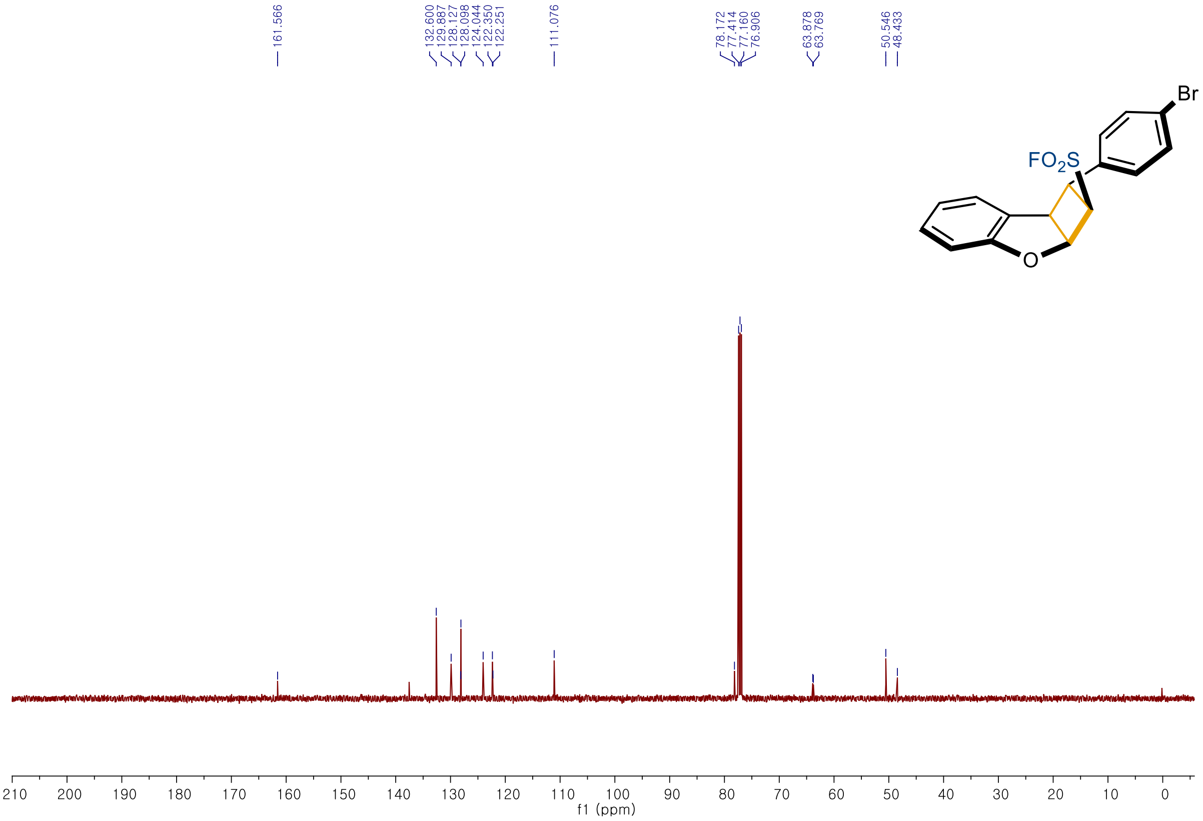

^{13}C NMR spectrum (**3ja**-*endo*)

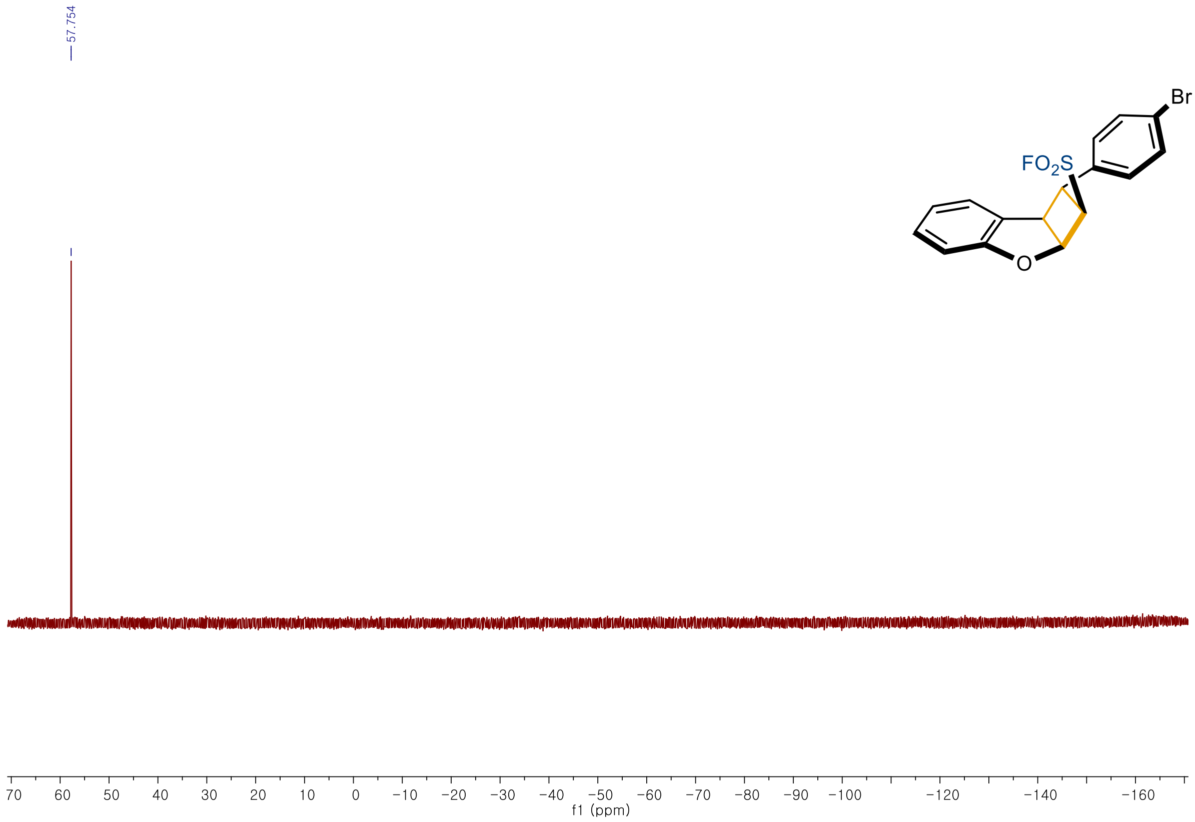

^{19}F NMR spectrum (**3ja**-*endo*)

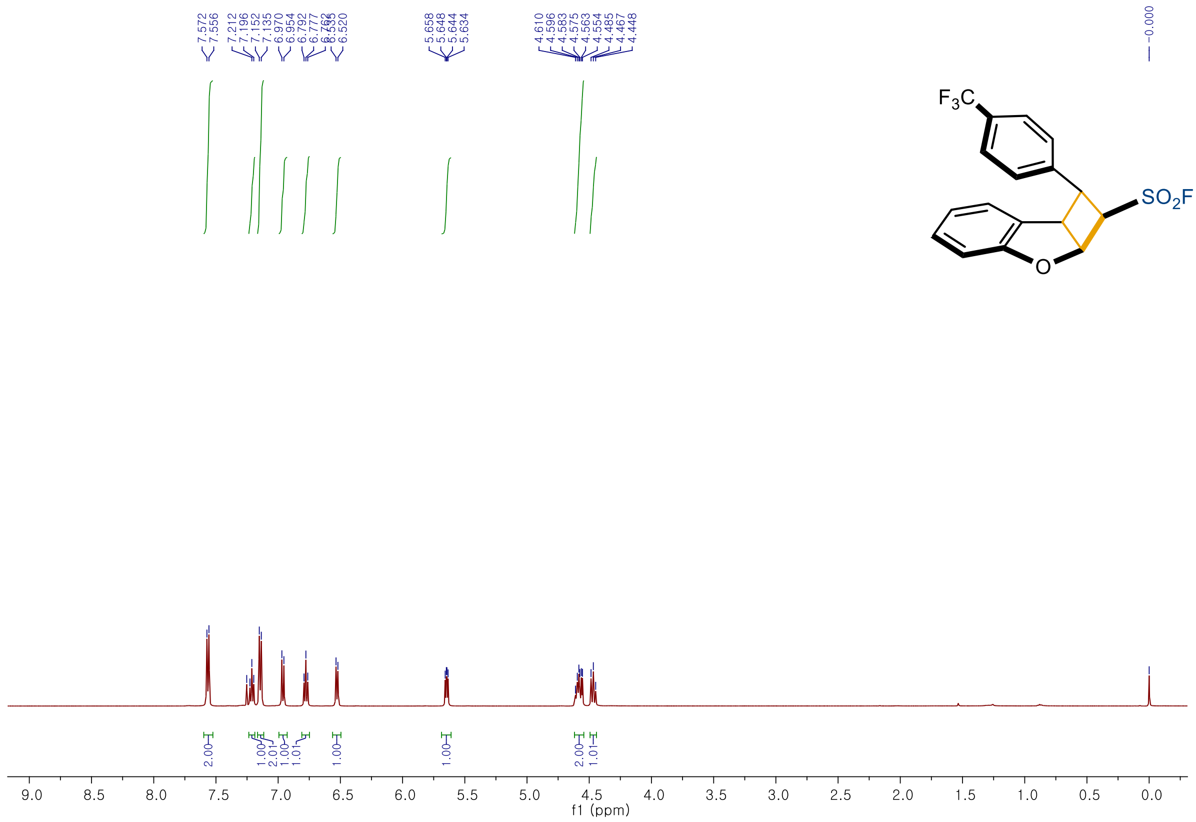

^{1}H NMR spectrum (**3ka**-*exo*)

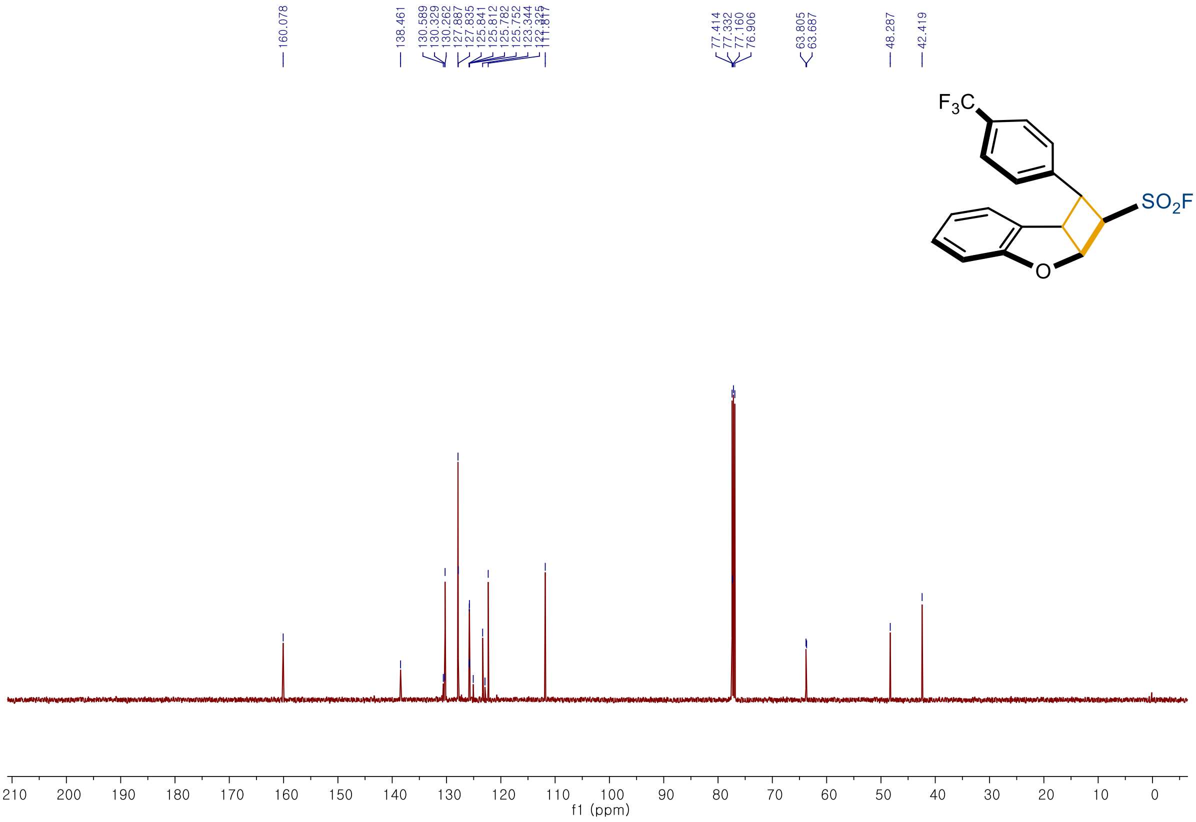

^{13}C NMR spectrum (**3ka**-*exo*)

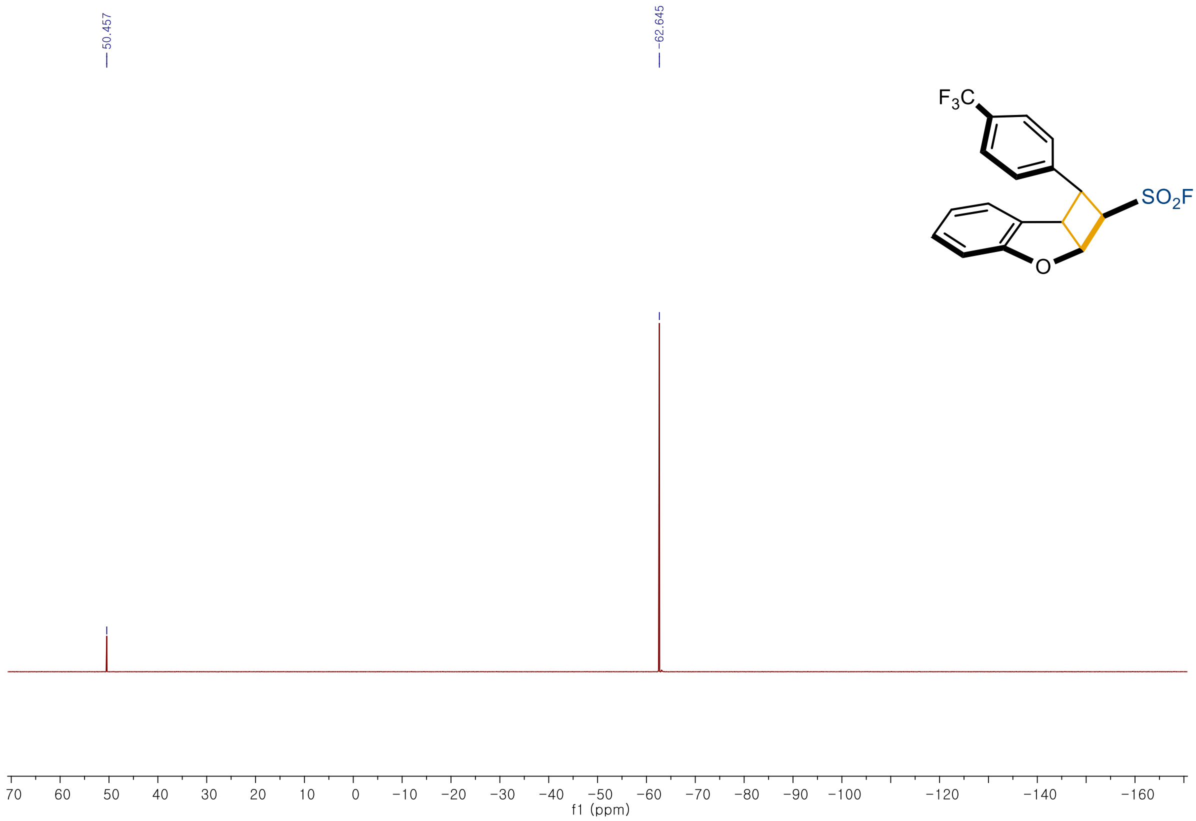

^{19}F NMR spectrum (**3ka**-*exo*)

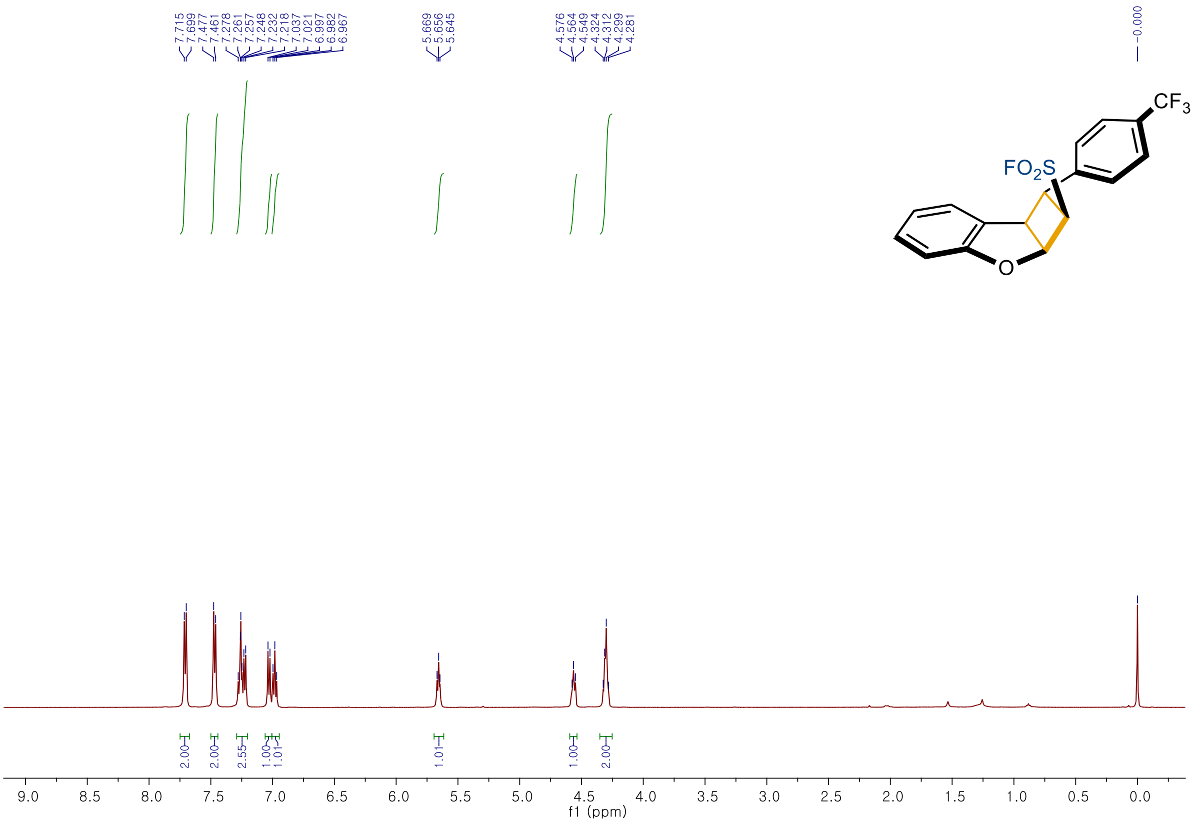

^{1}H NMR spectrum (**3ka**-*endo*)

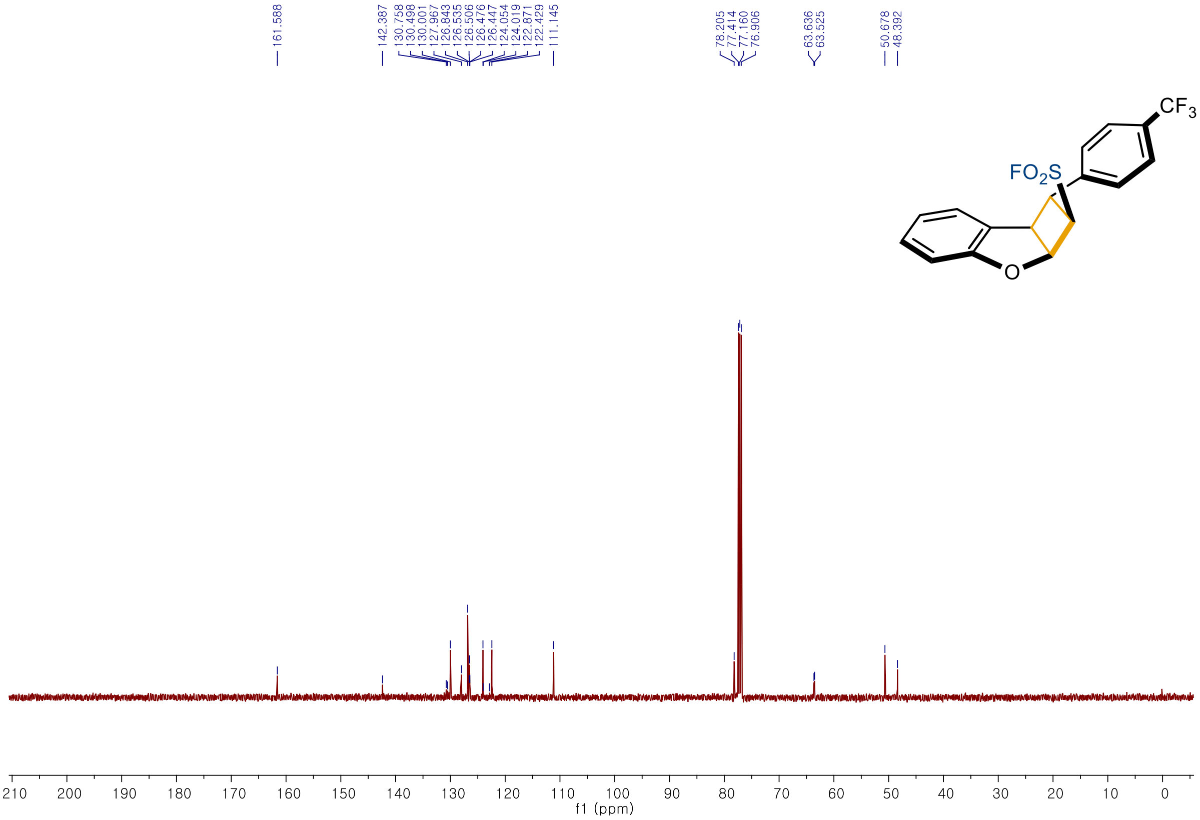

^{13}C NMR spectrum (**3ka**-*endo*)

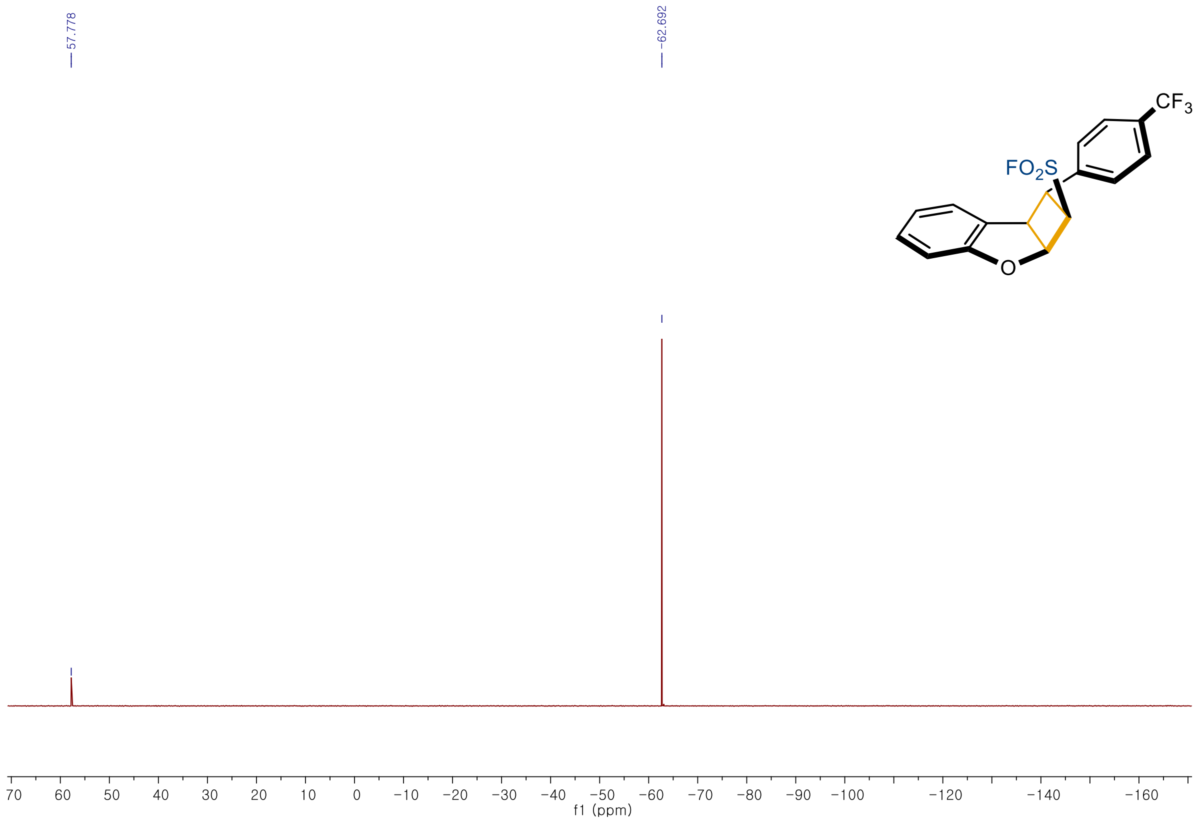

^{19}F NMR spectrum (**3ka**-*endo*)

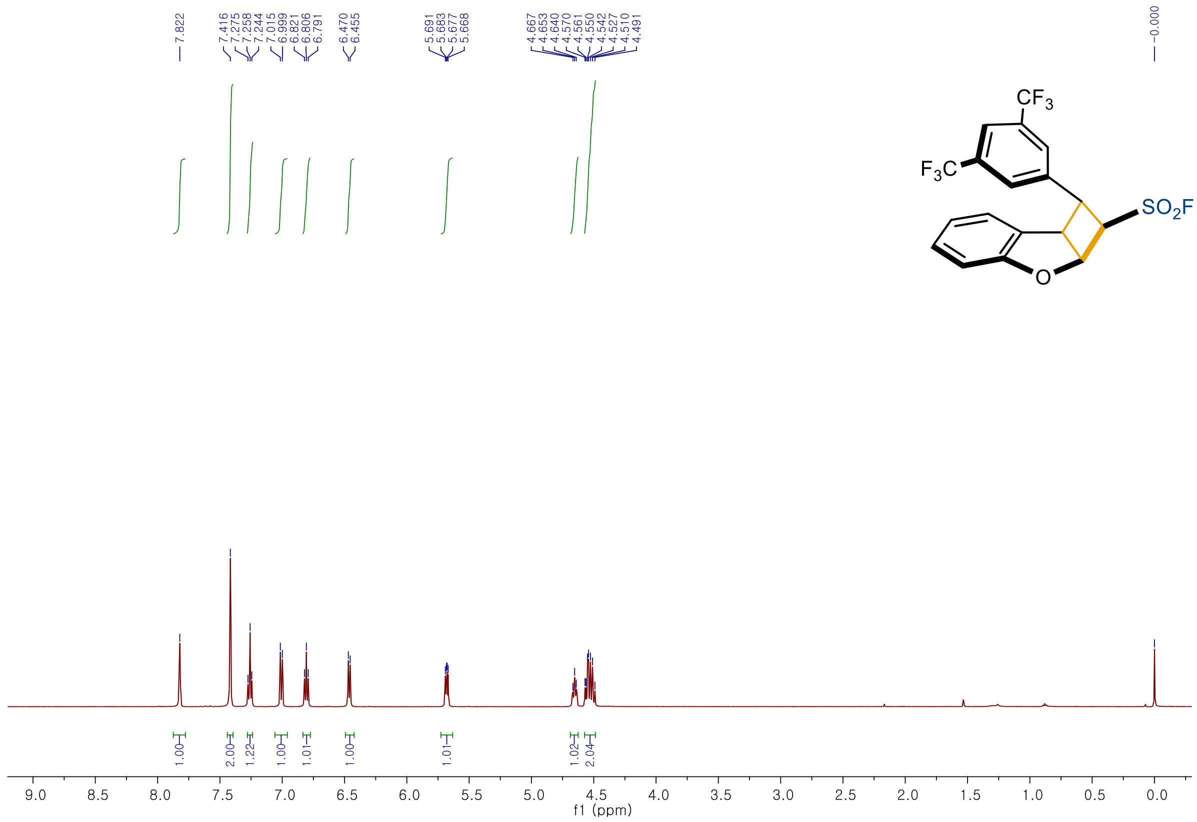

^{1}H NMR spectrum (**3la**-*exo*)

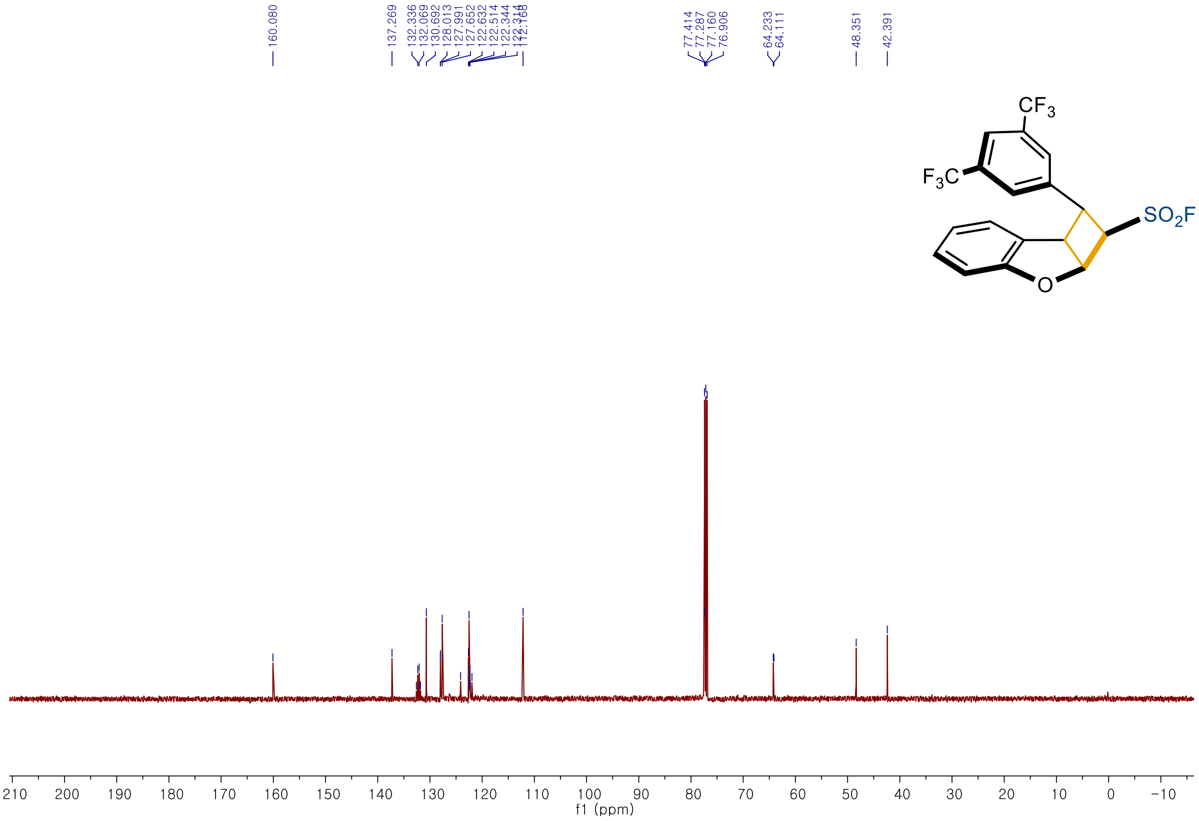

^{13}C NMR spectrum (**3la**-*exo*)

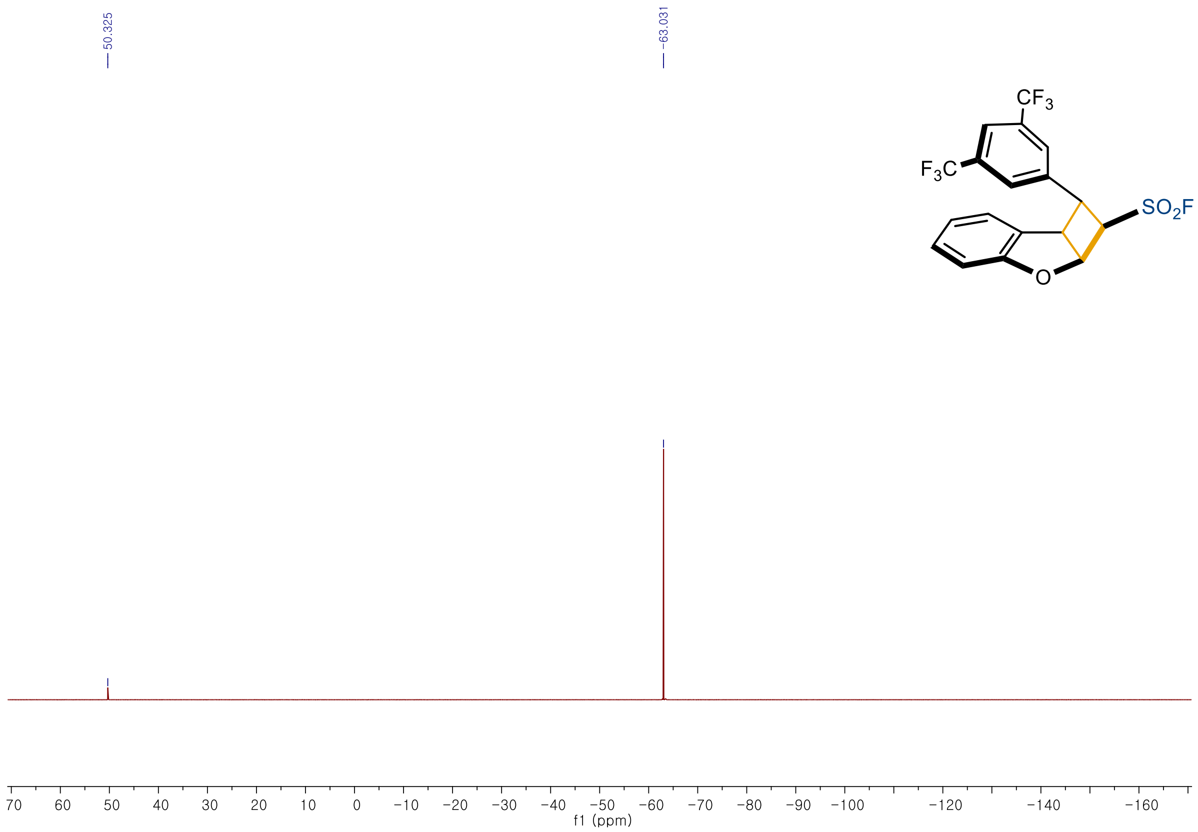

^{19}F NMR spectrum (**3la**-*exo*)

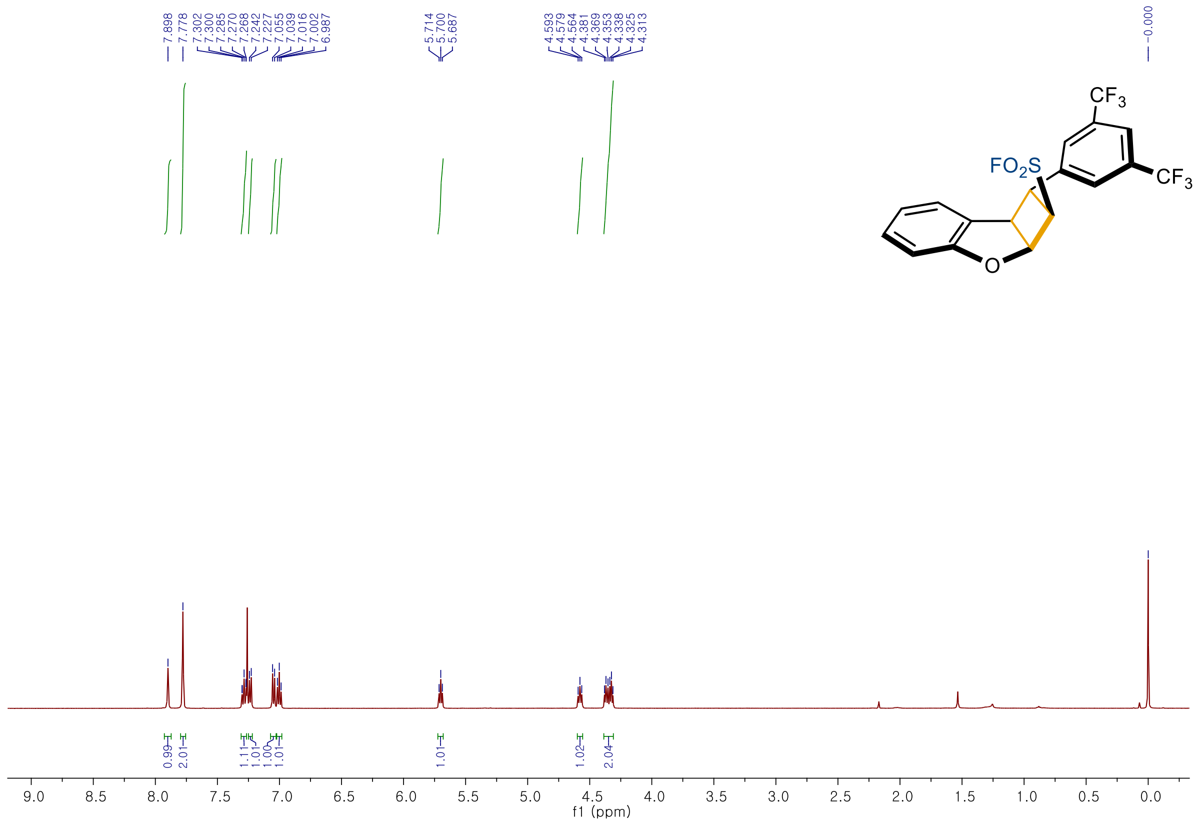

^{1}H NMR spectrum (**3la**-*endo*)

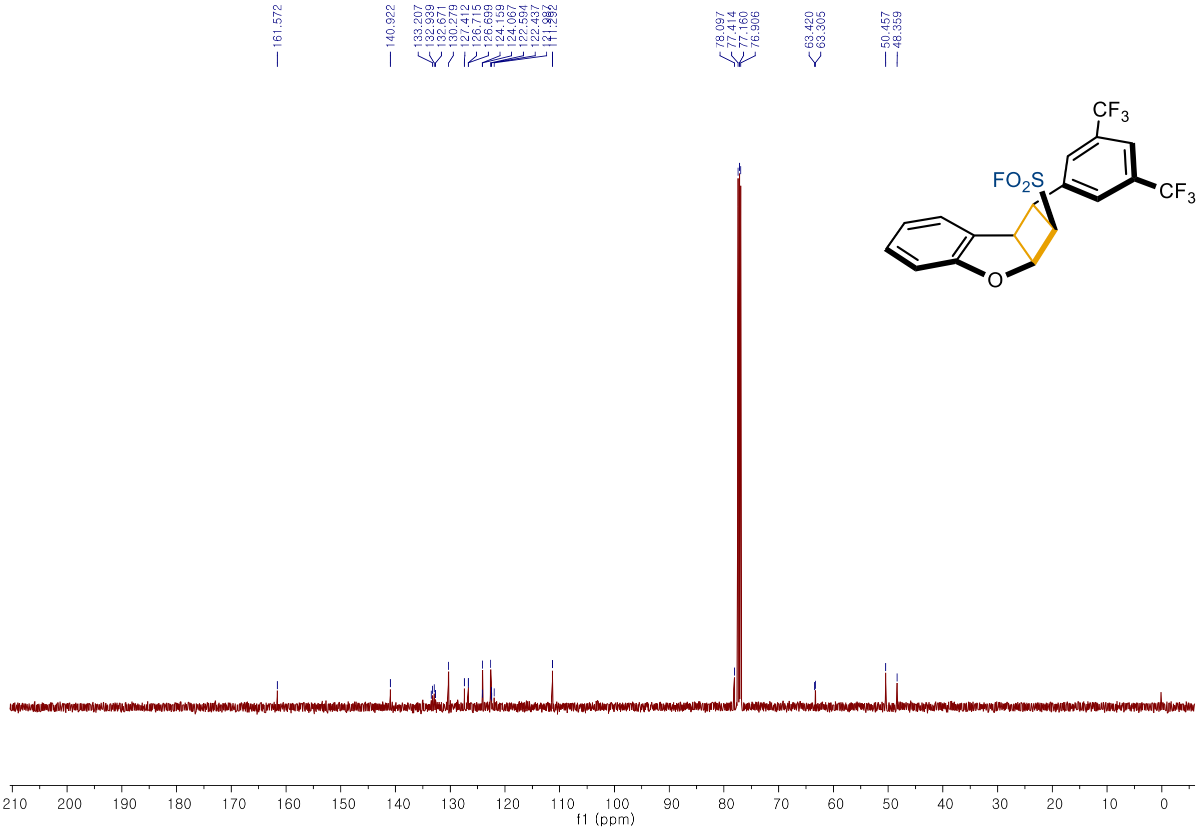

^{13}C NMR spectrum (**3la**-*endo*)

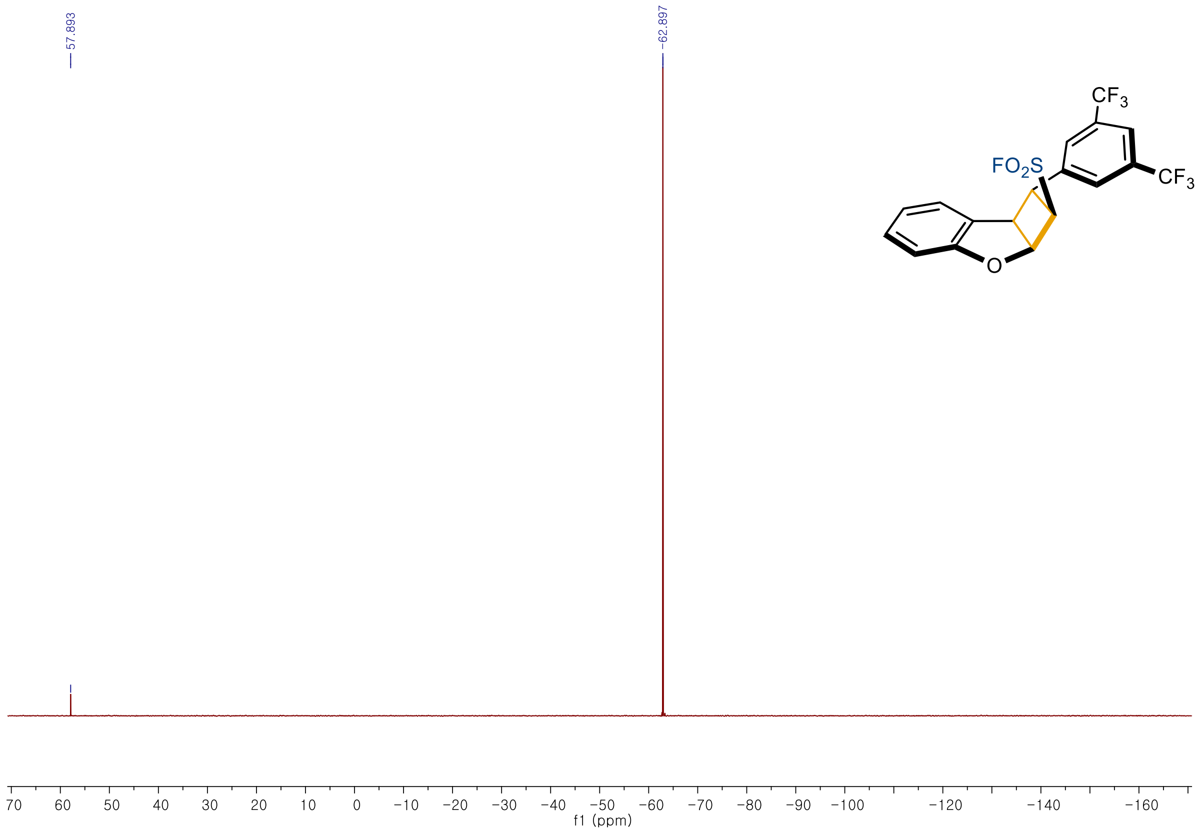

^{19}F NMR spectrum (**3la**-*endo*)

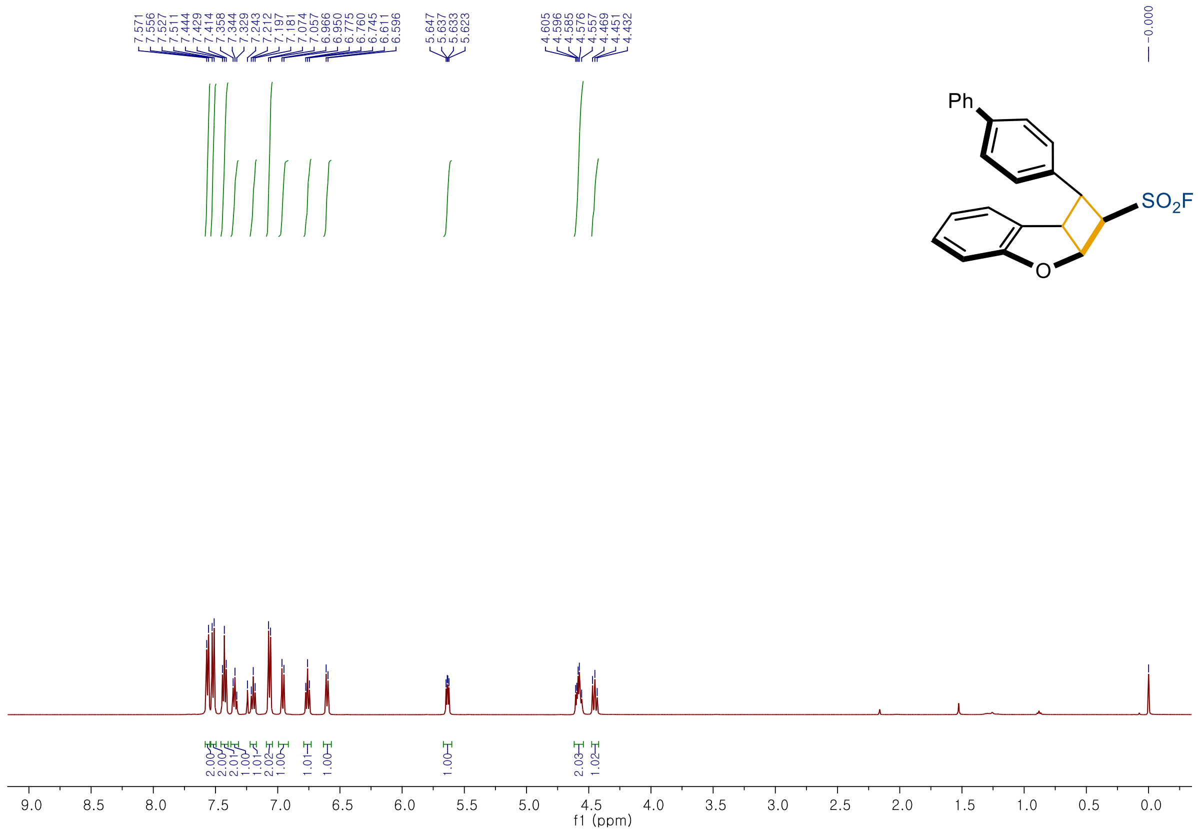

^{1}H NMR spectrum (**3ma**-*exo*)

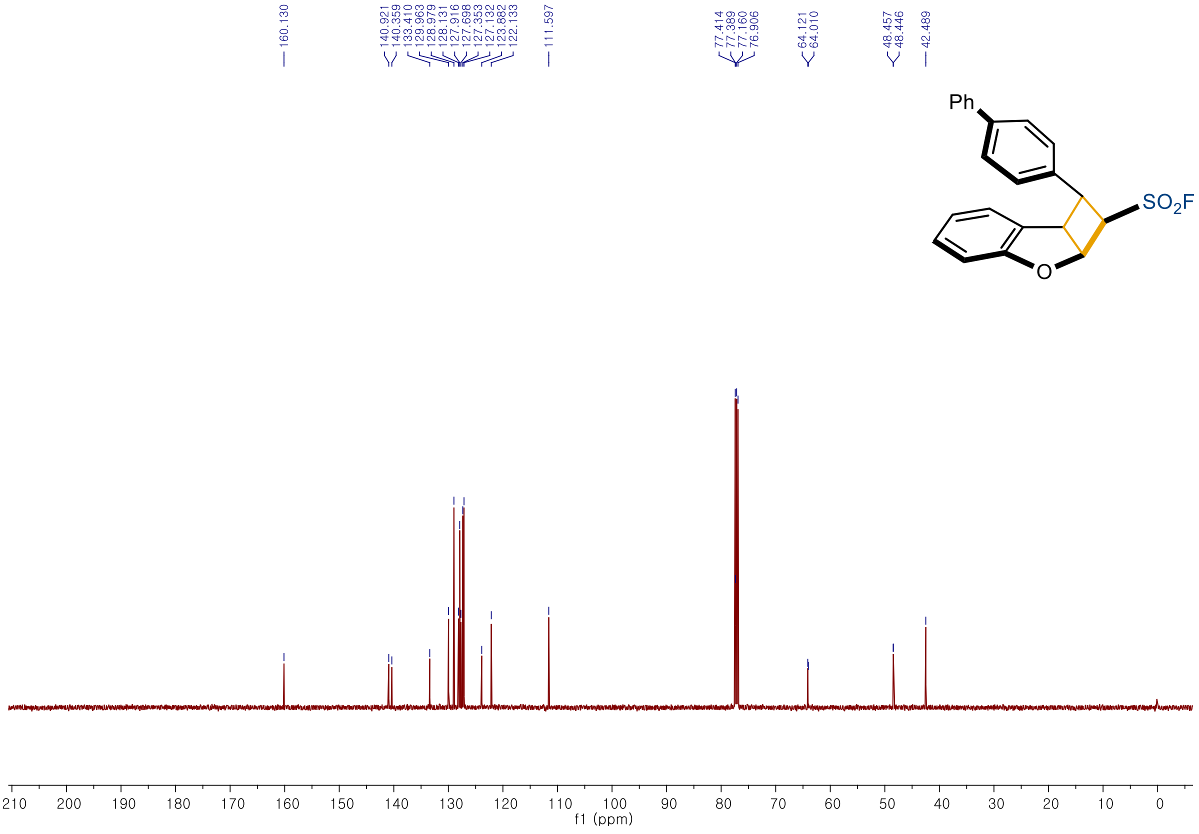

^{13}C NMR spectrum (**3ma**-*exo*)

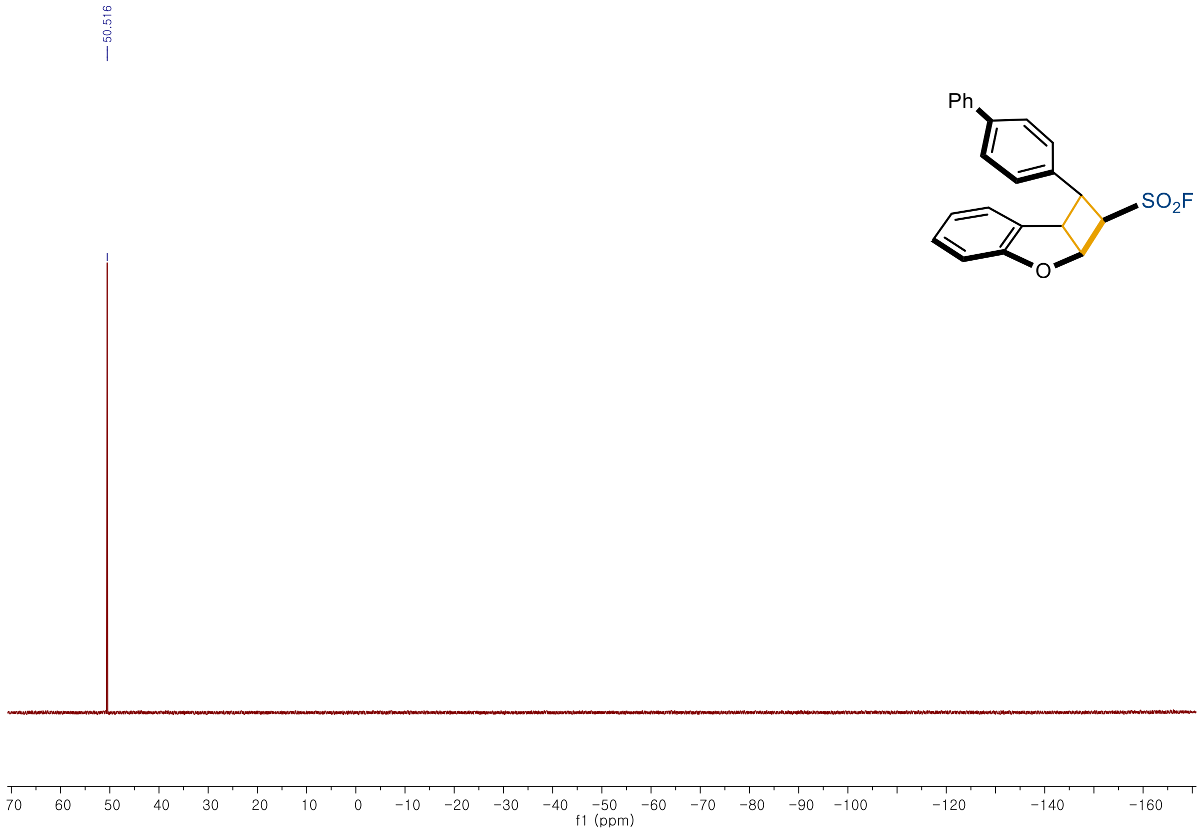

^{19}F NMR spectrum (**3ma**-*exo*)

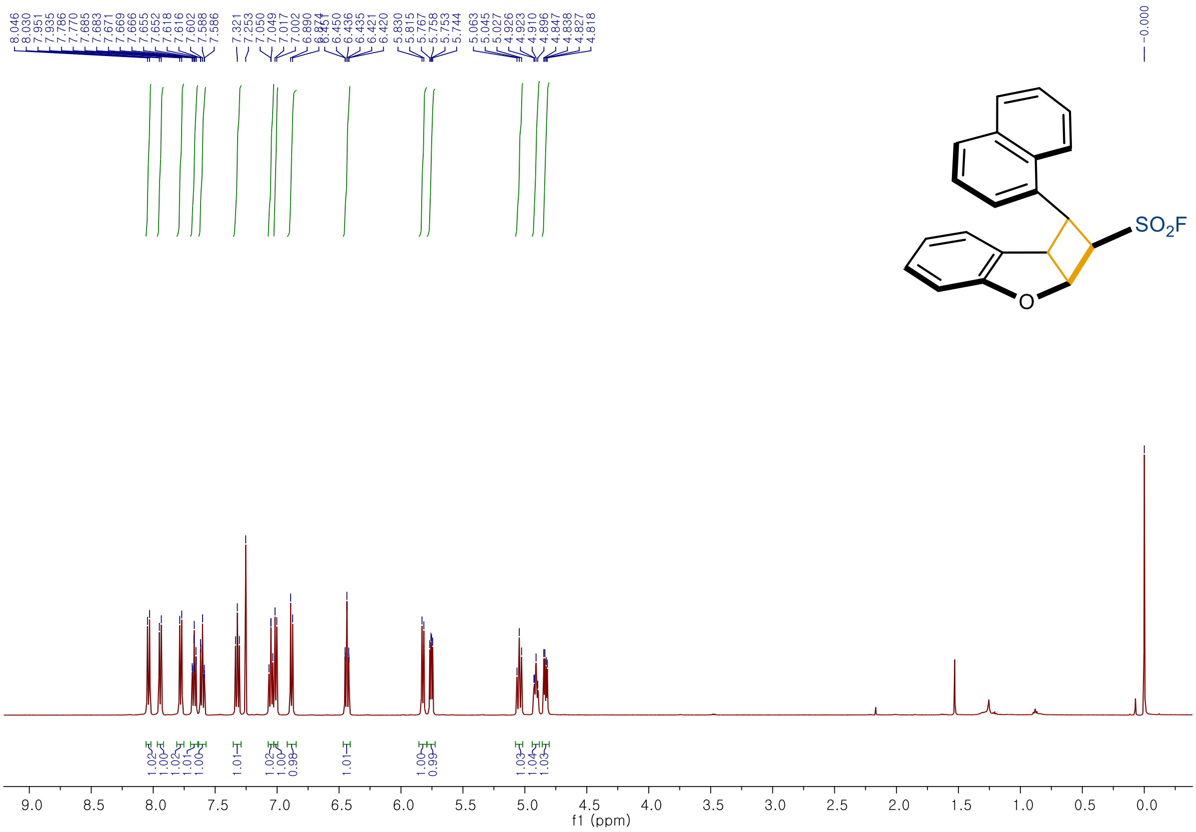

^{1}H NMR spectrum (**3na**-*exo*)

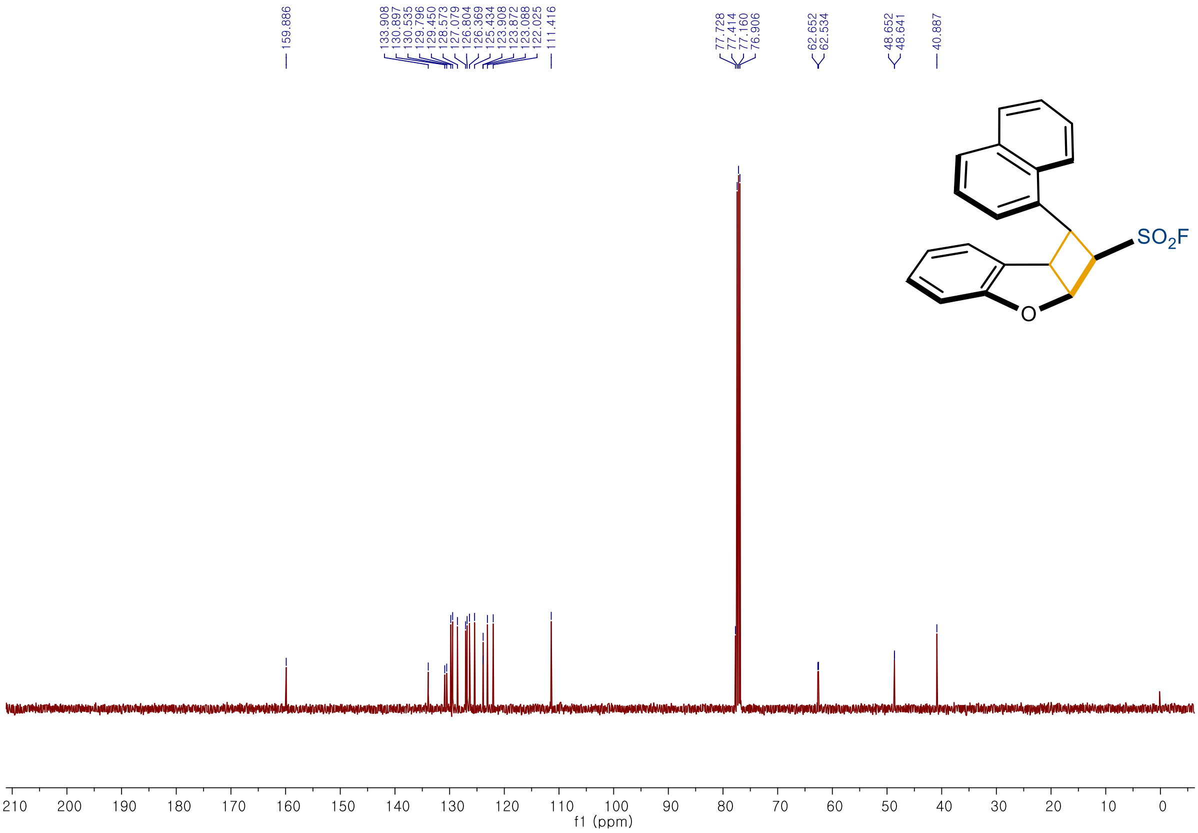

^{13}C NMR spectrum (**3na**-*exo*)

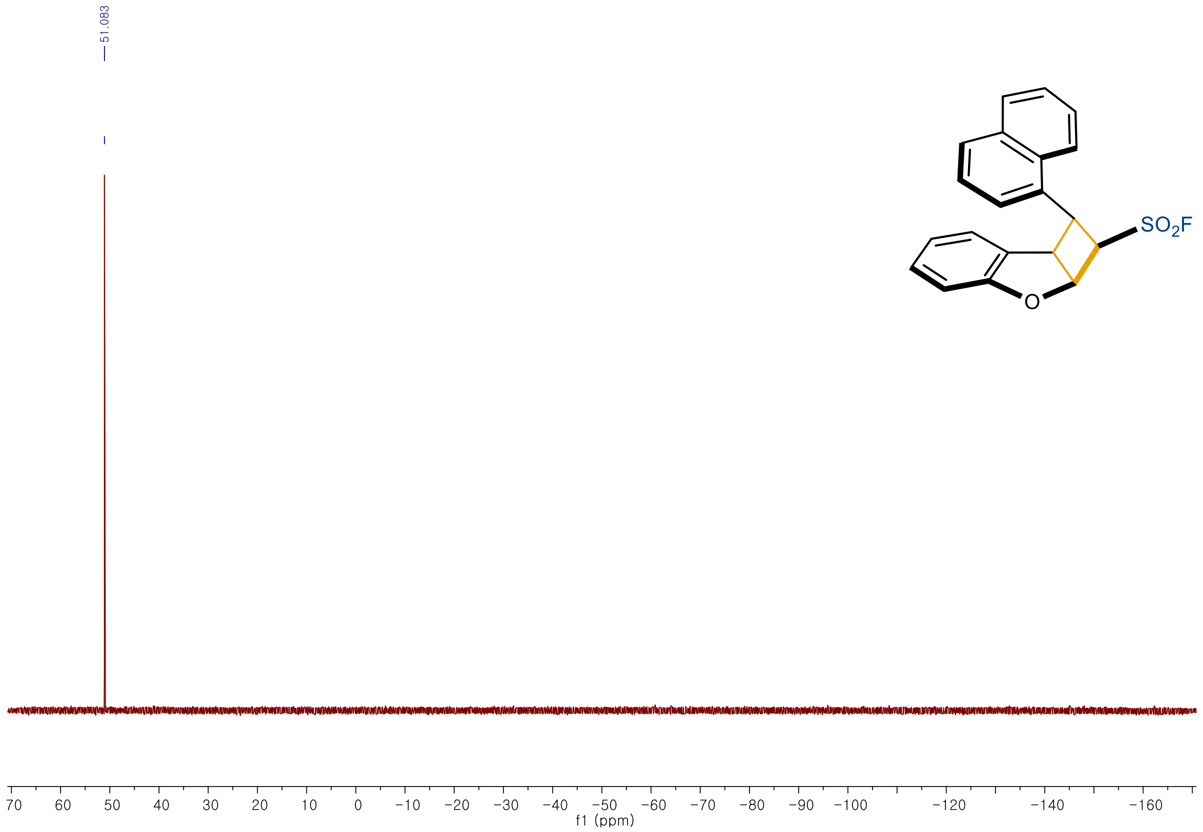

^{19}F NMR spectrum (**3na**-*exo*)

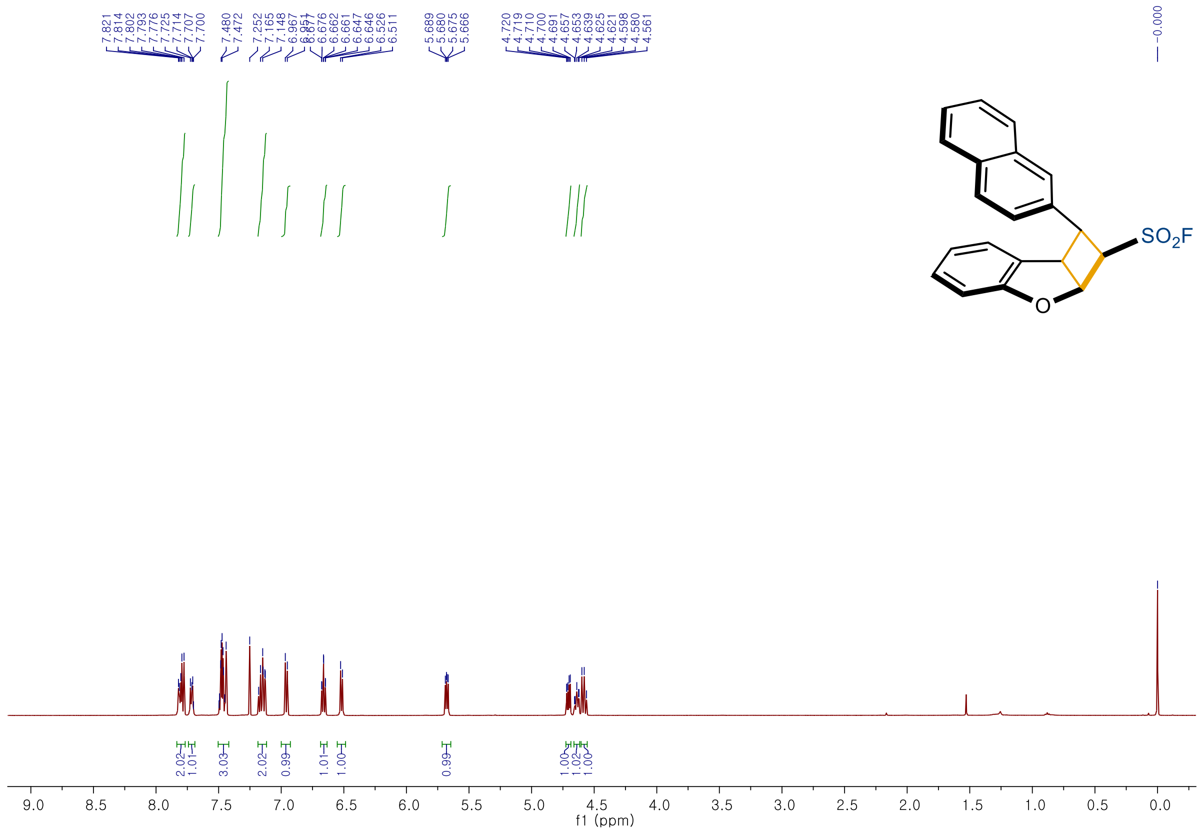

^{1}H NMR spectrum (**3oa**-*exo*)

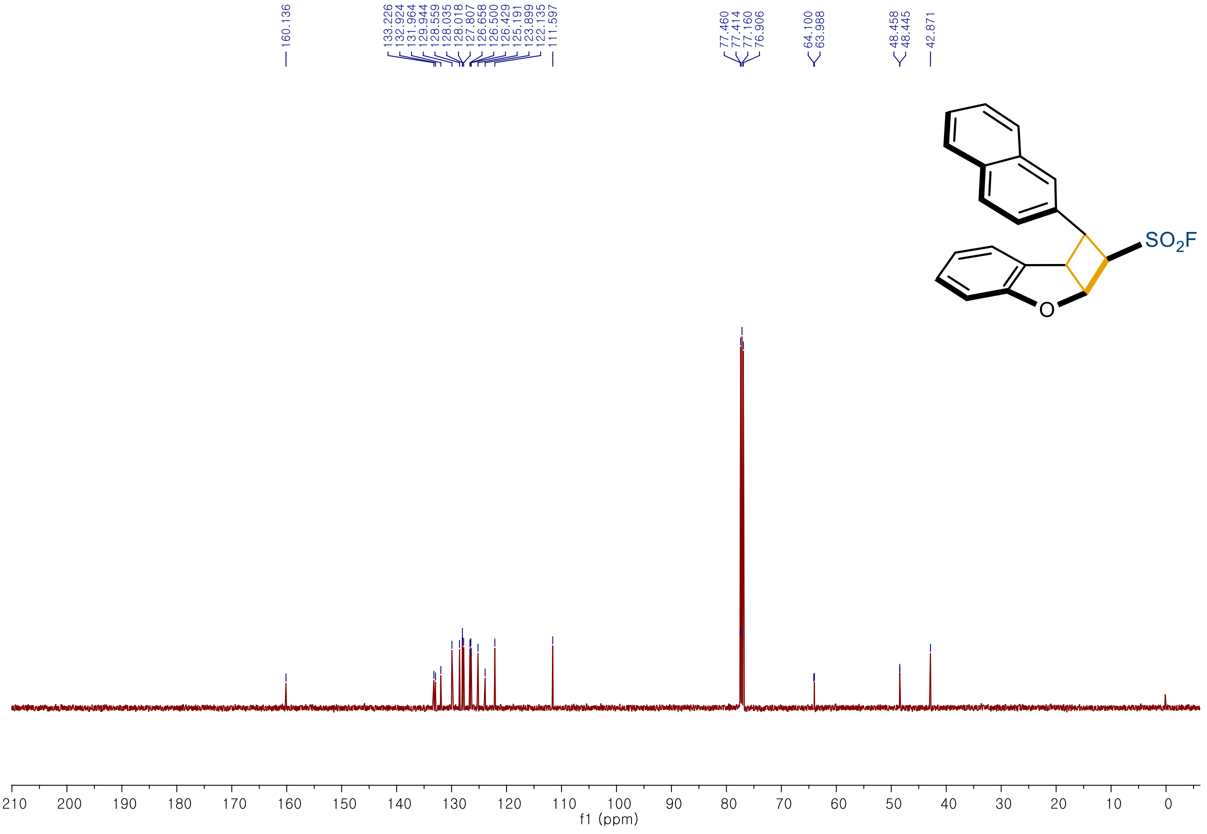

^{13}C NMR spectrum (**3oa**-*exo*)

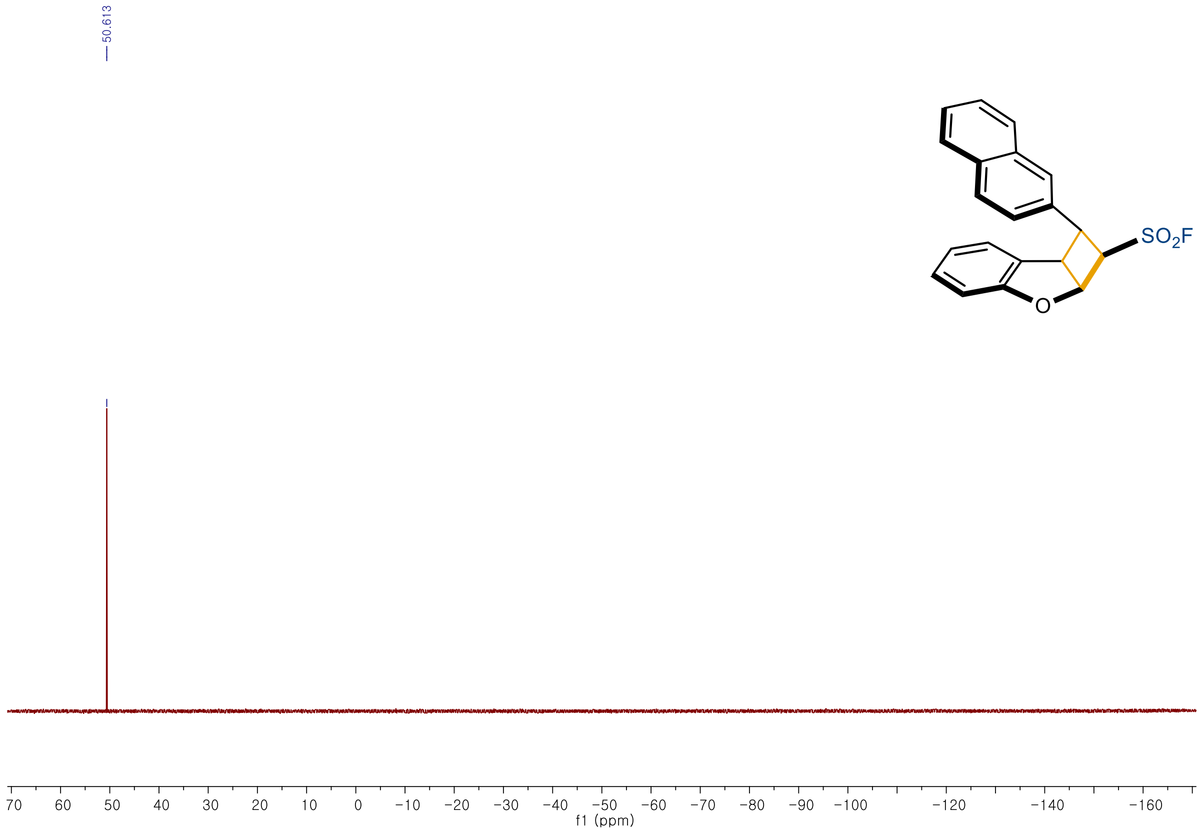

^{19}F NMR spectrum (**3oa**-*exo*)

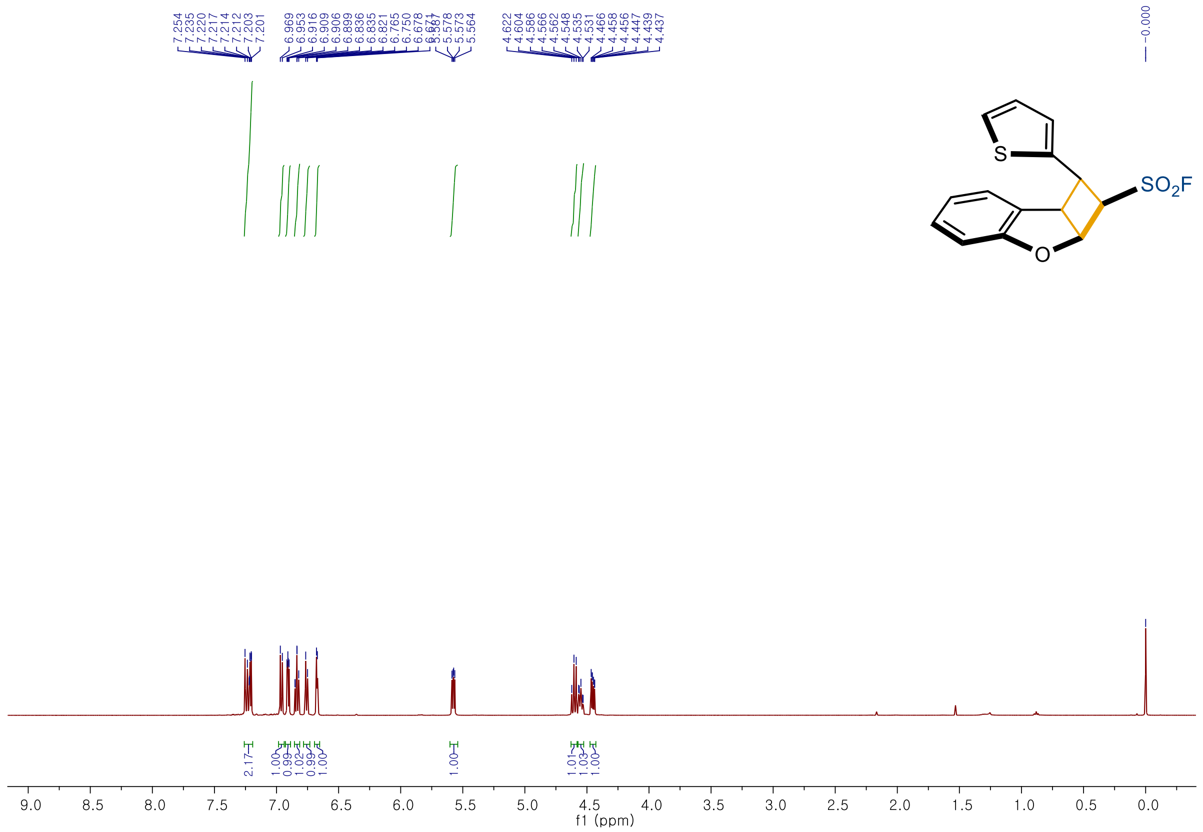

^{1}H NMR spectrum (**3pa**-*exo*)

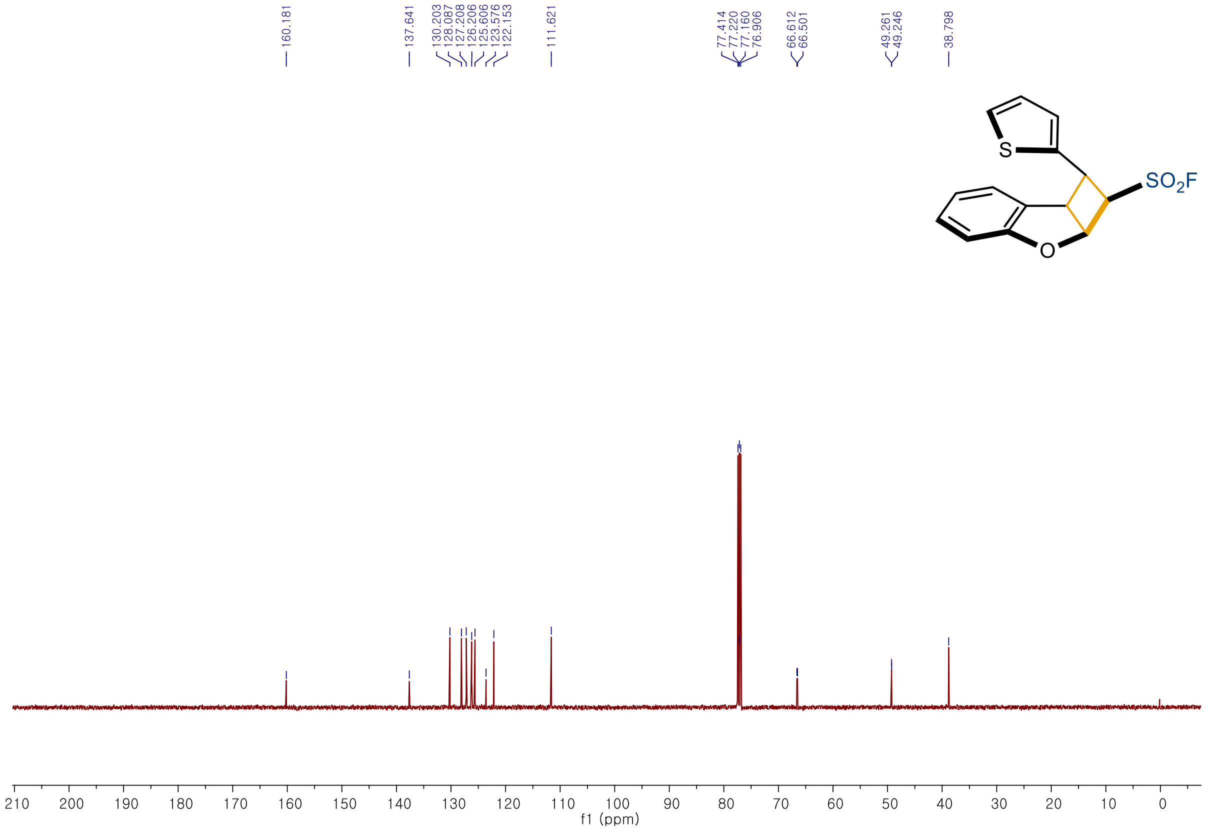

^{13}C NMR spectrum (**3pa**-*exo*)

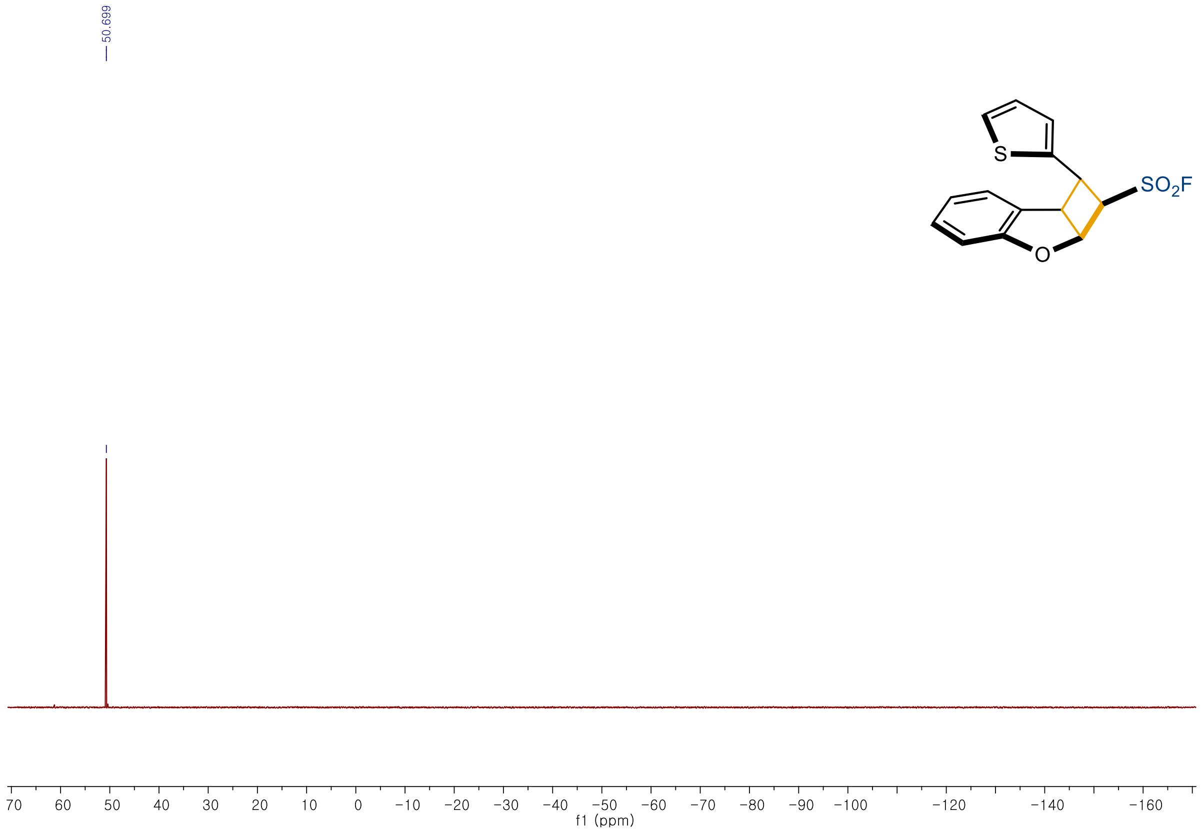

^{19}F NMR spectrum (**3pa**-*exo*)

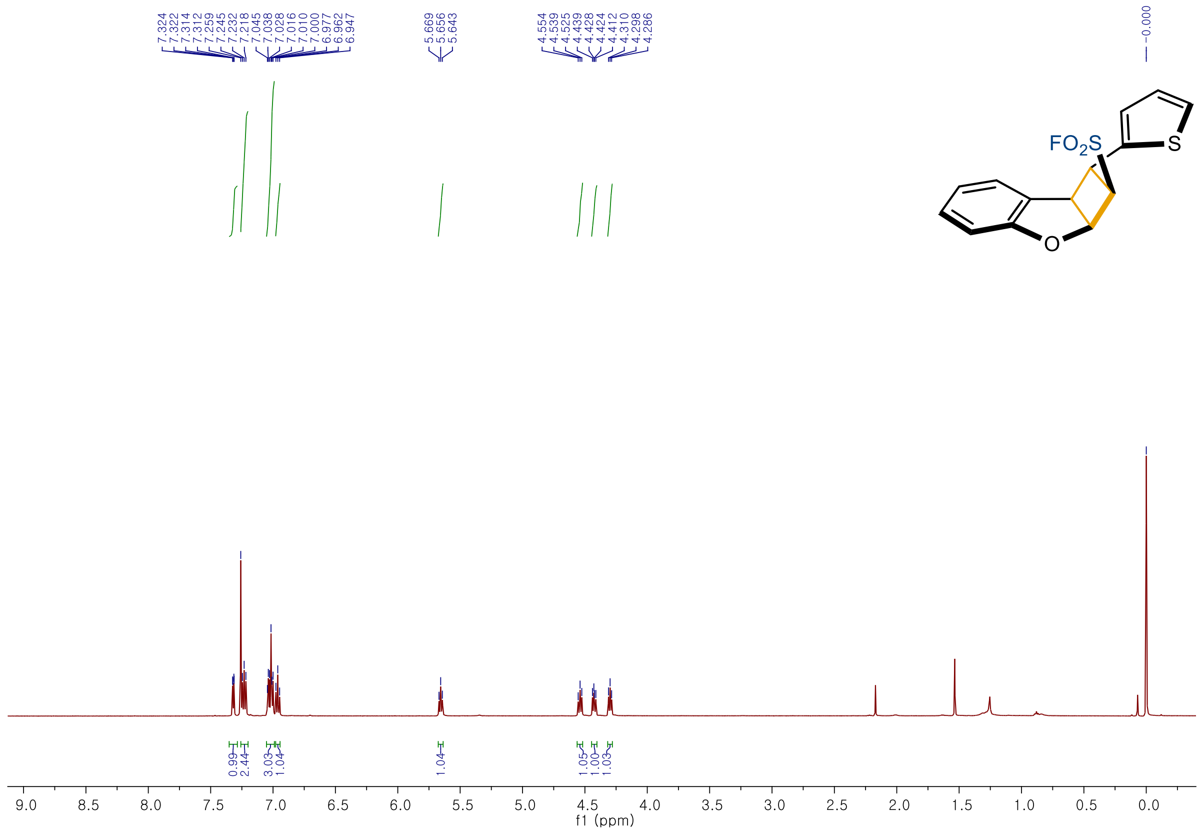

^{1}H NMR spectrum (**3pa**-*endo*)

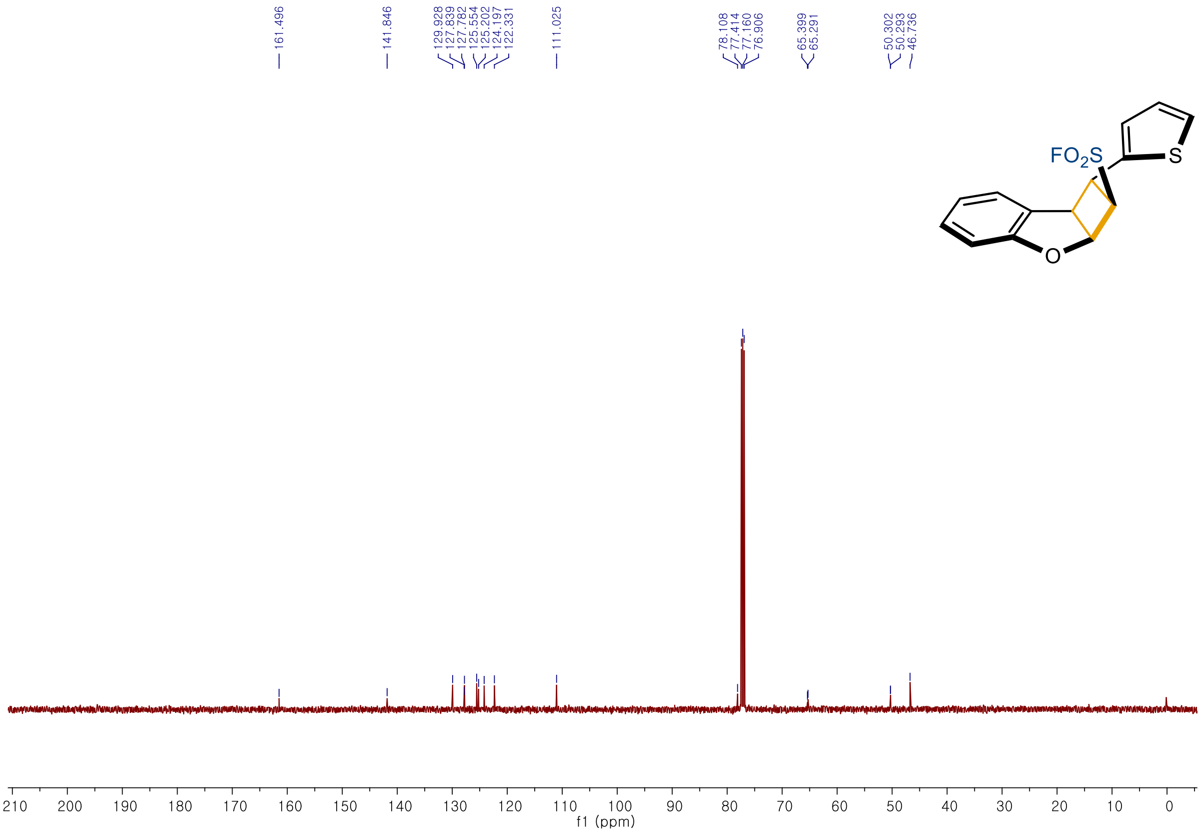

^{13}C NMR spectrum (**3pa**-*endo*)

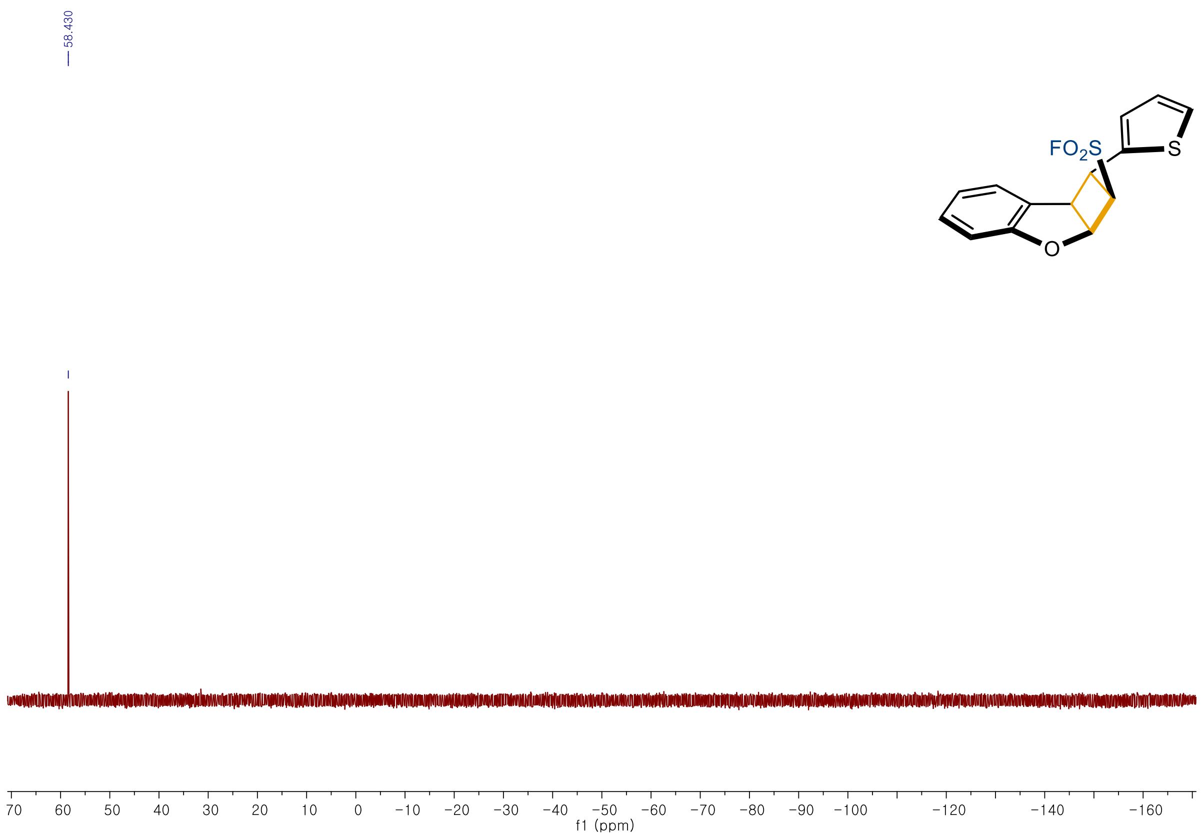

^{19}F NMR spectrum (**3pa**-*endo*)

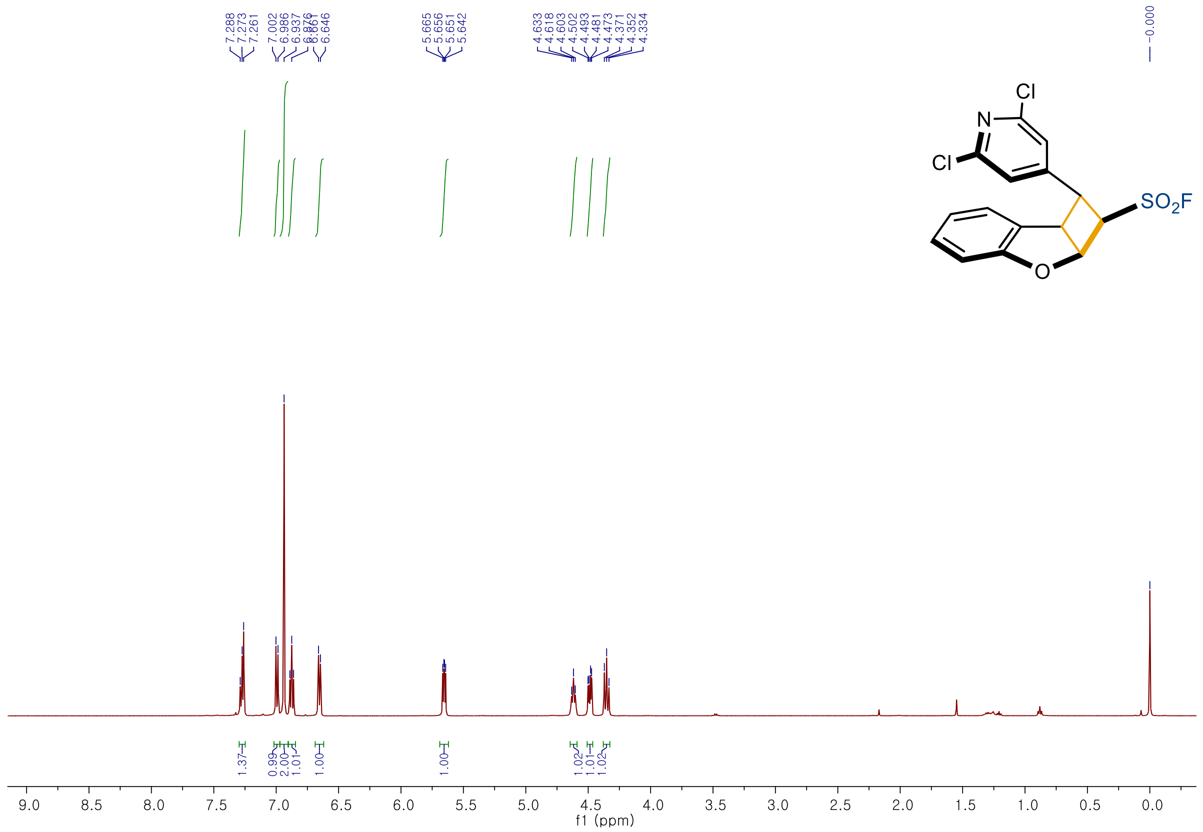

^{1}H NMR spectrum (**3qa**-*exo*)

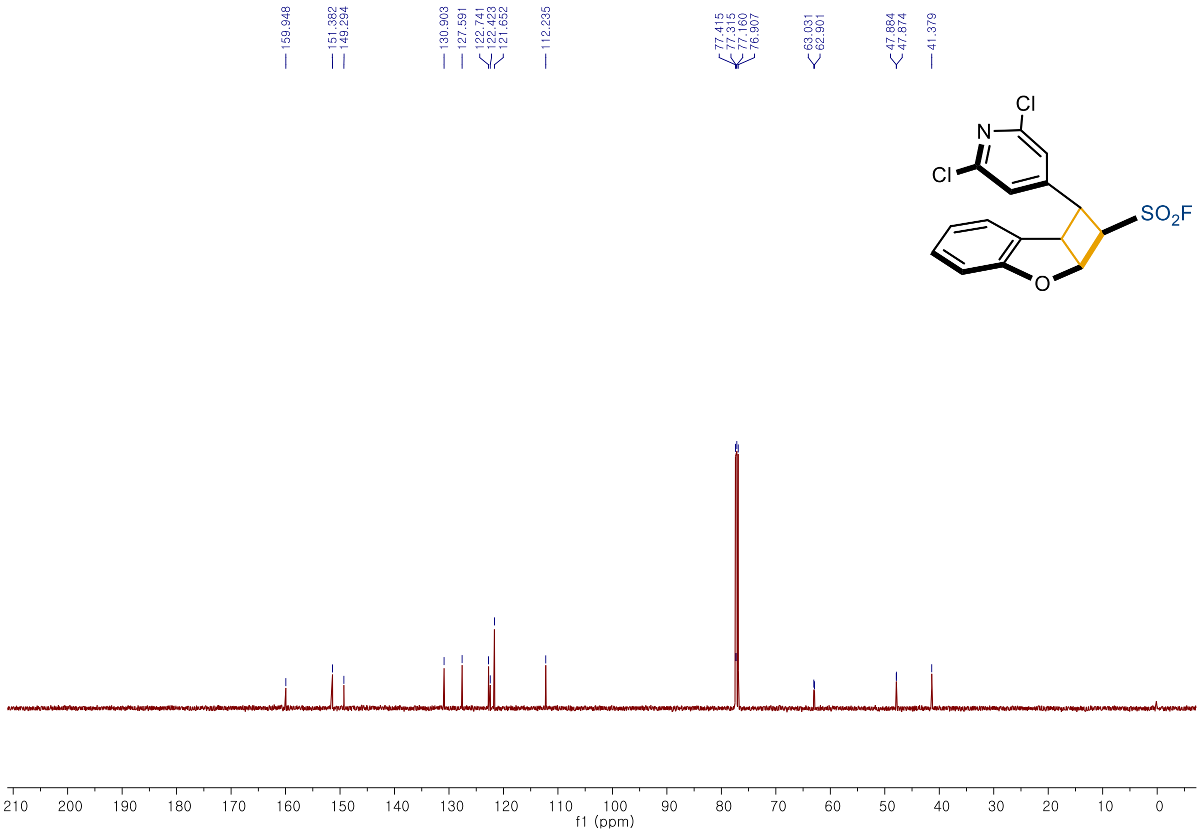

^{13}C NMR spectrum (**3qa**-*exo*))

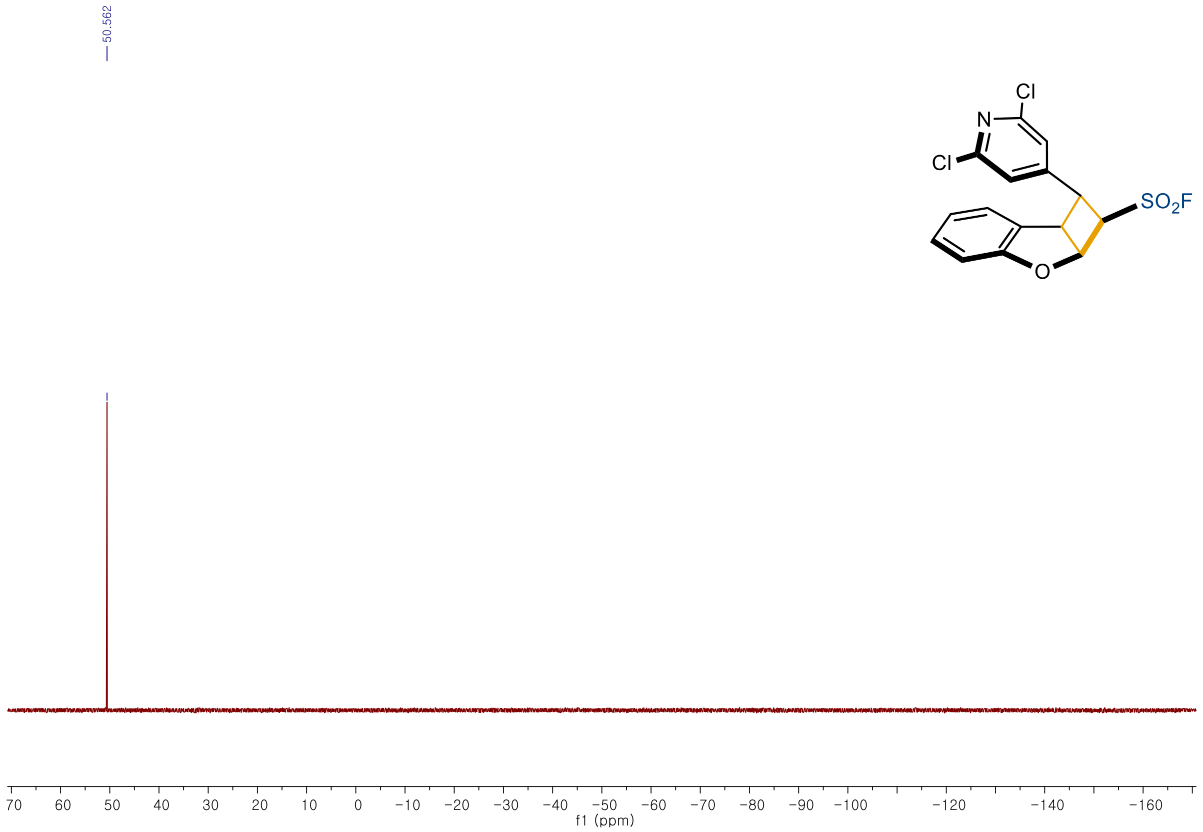

^{19}F NMR spectrum (**3qa**-*exo*)

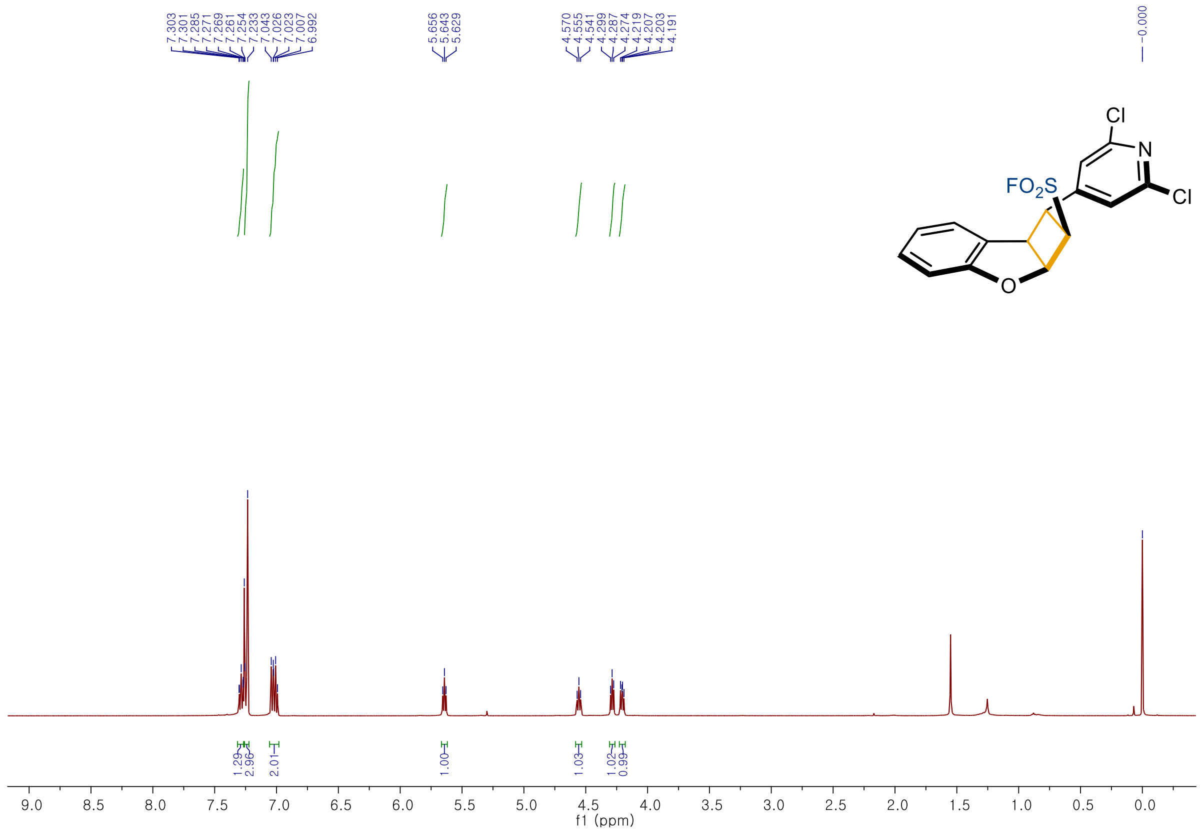

^{1}H NMR spectrum (**3qa**-*endo*)

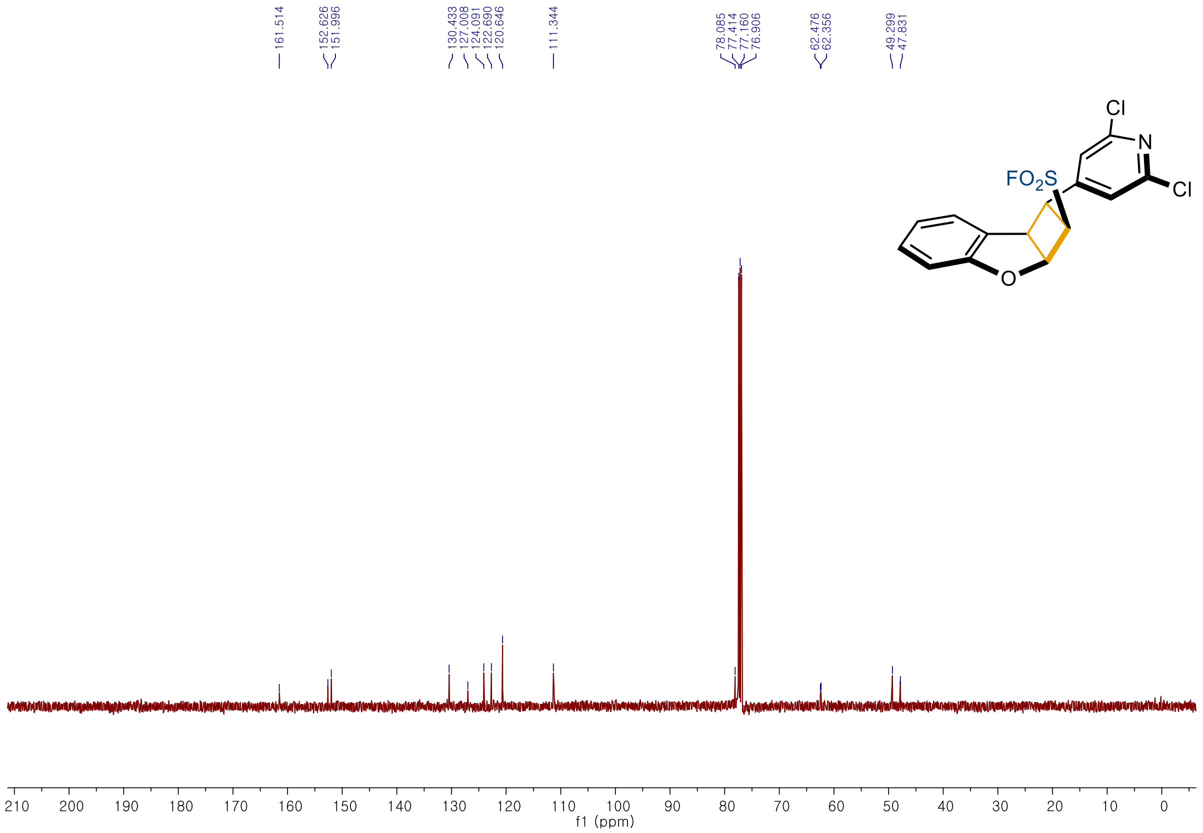

^{13}C NMR spectrum (**3qa**-*endo*)

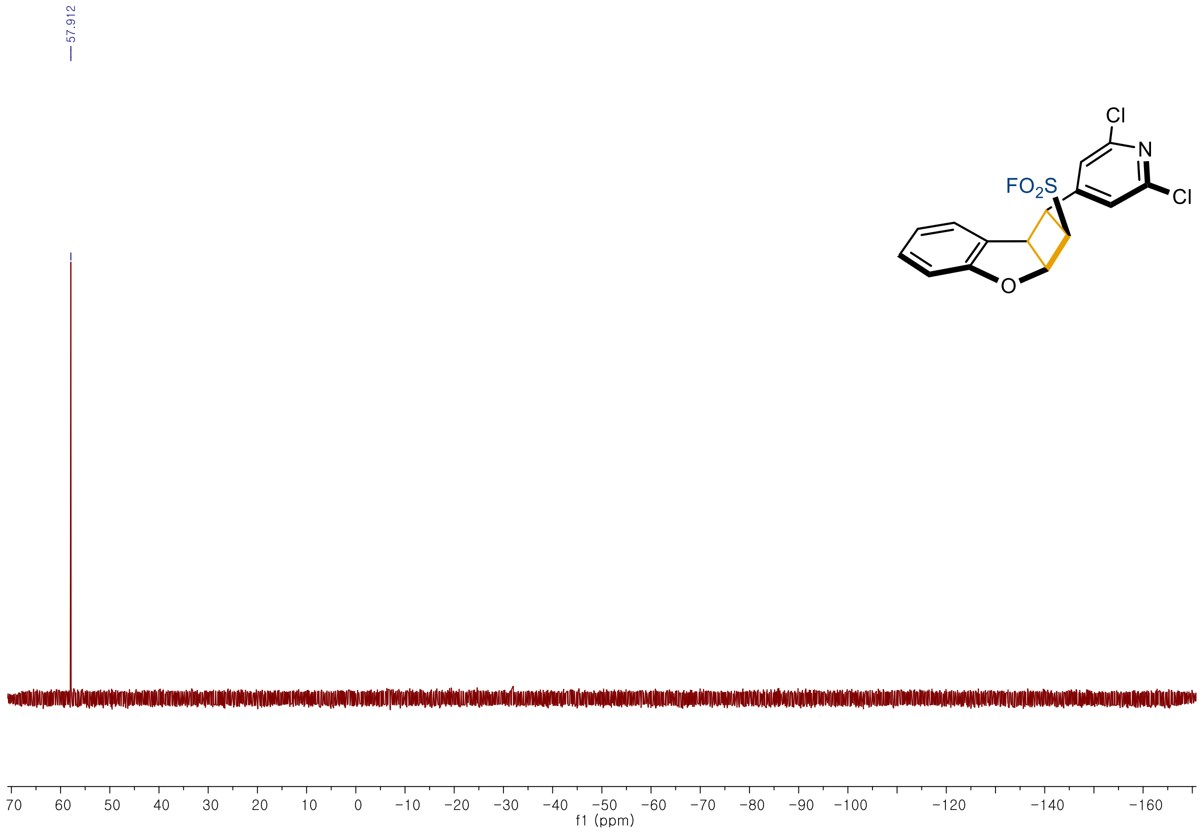

^{19}F NMR spectrum (**3qa**-*endo*)

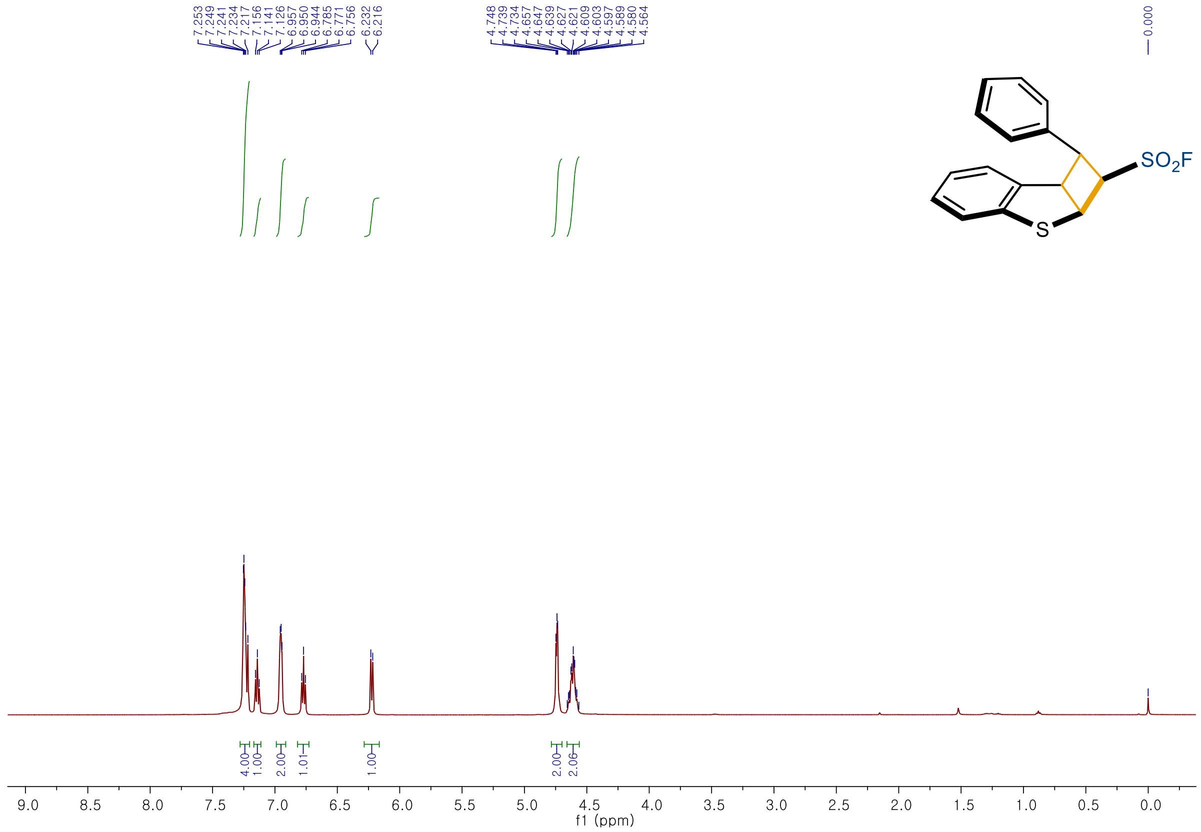

^{1}H NMR spectrum (**3ab**-*exo*)

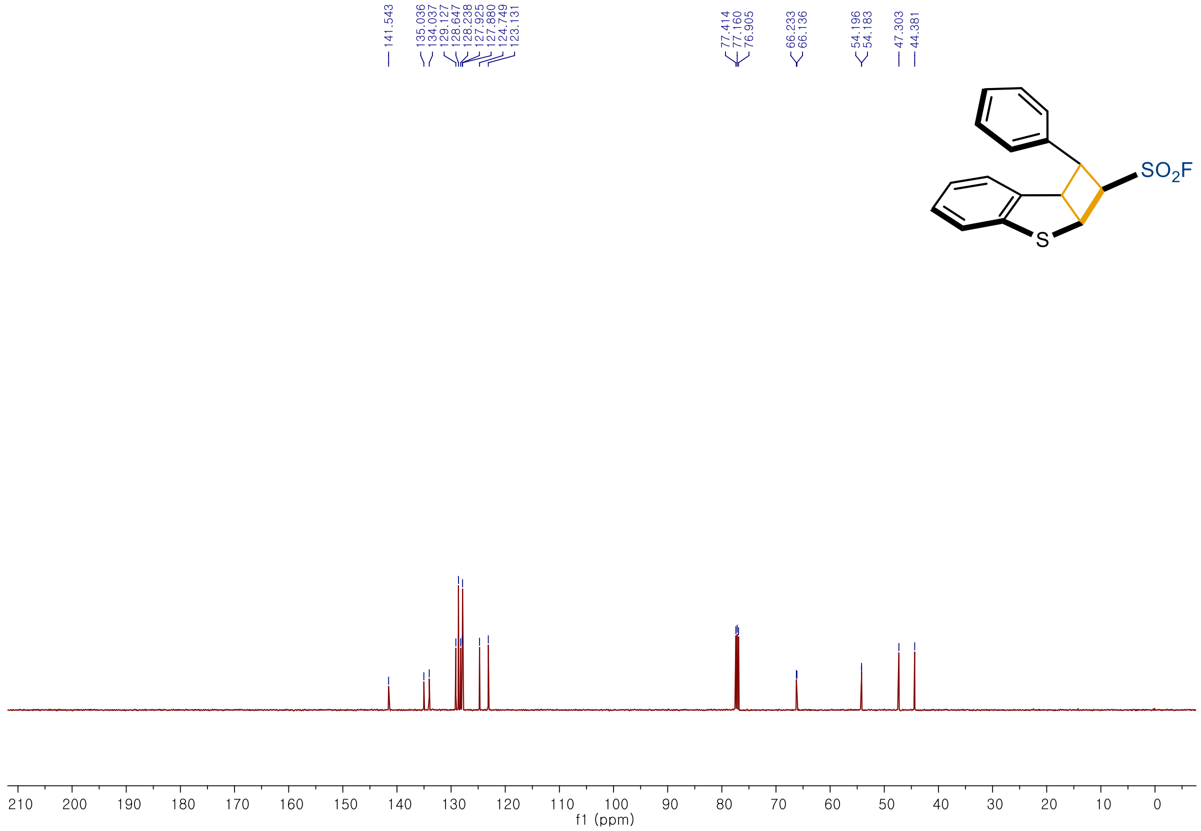

^{13}C NMR spectrum (**3ab**-*exo*)

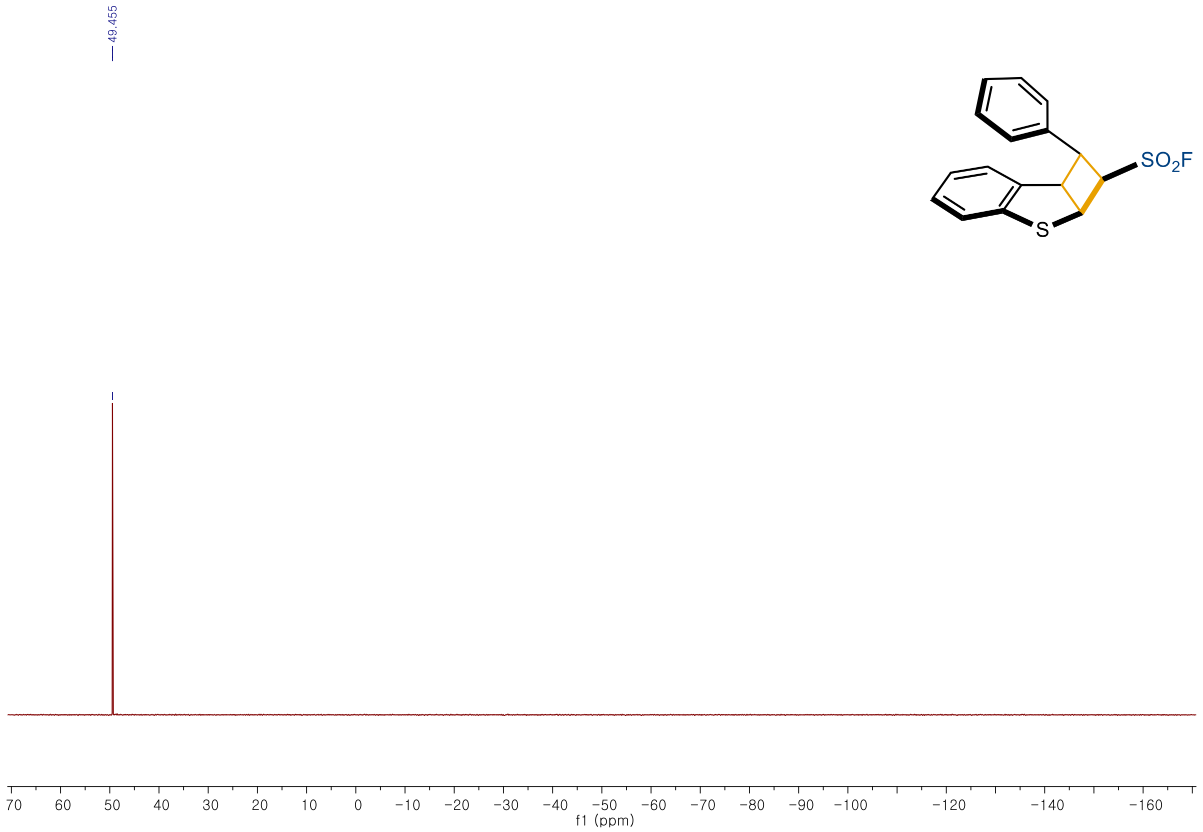

^{19}F NMR spectrum (**3ab**-*exo*)

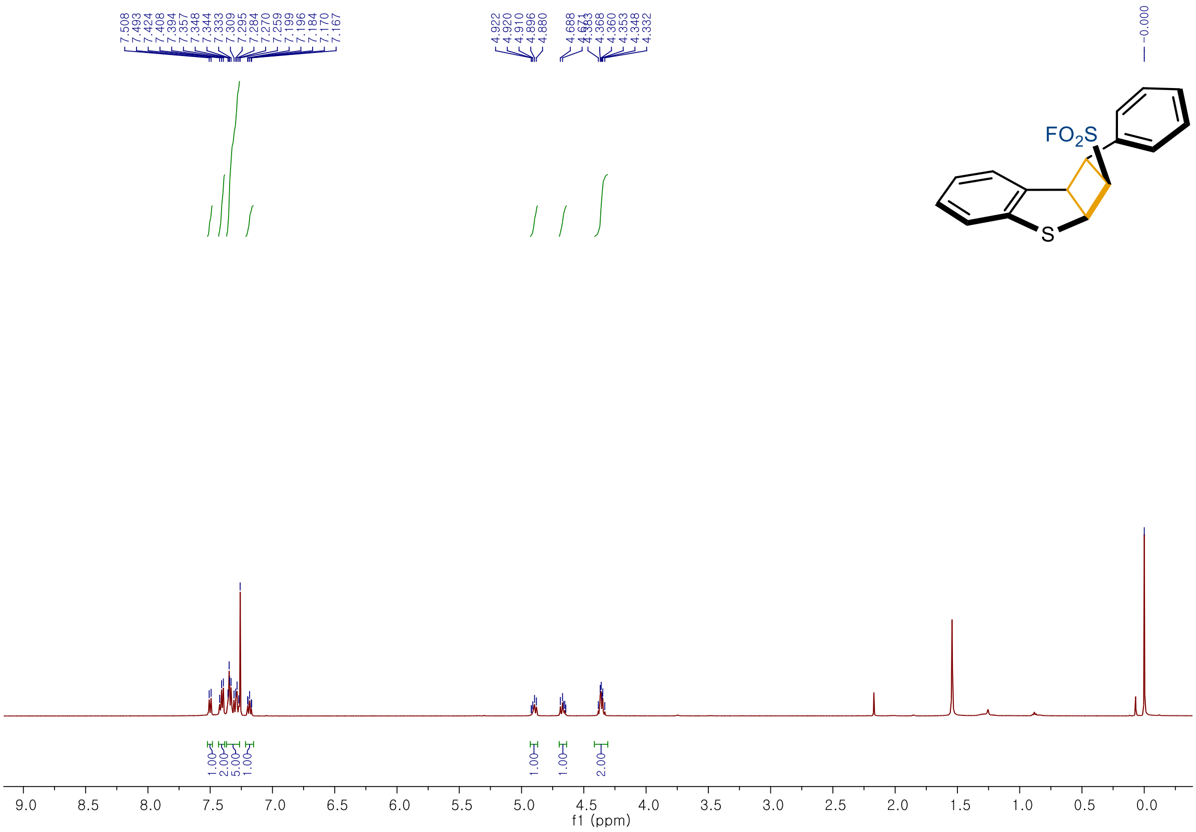

^{1}H NMR spectrum (**3ab**-*endo*)

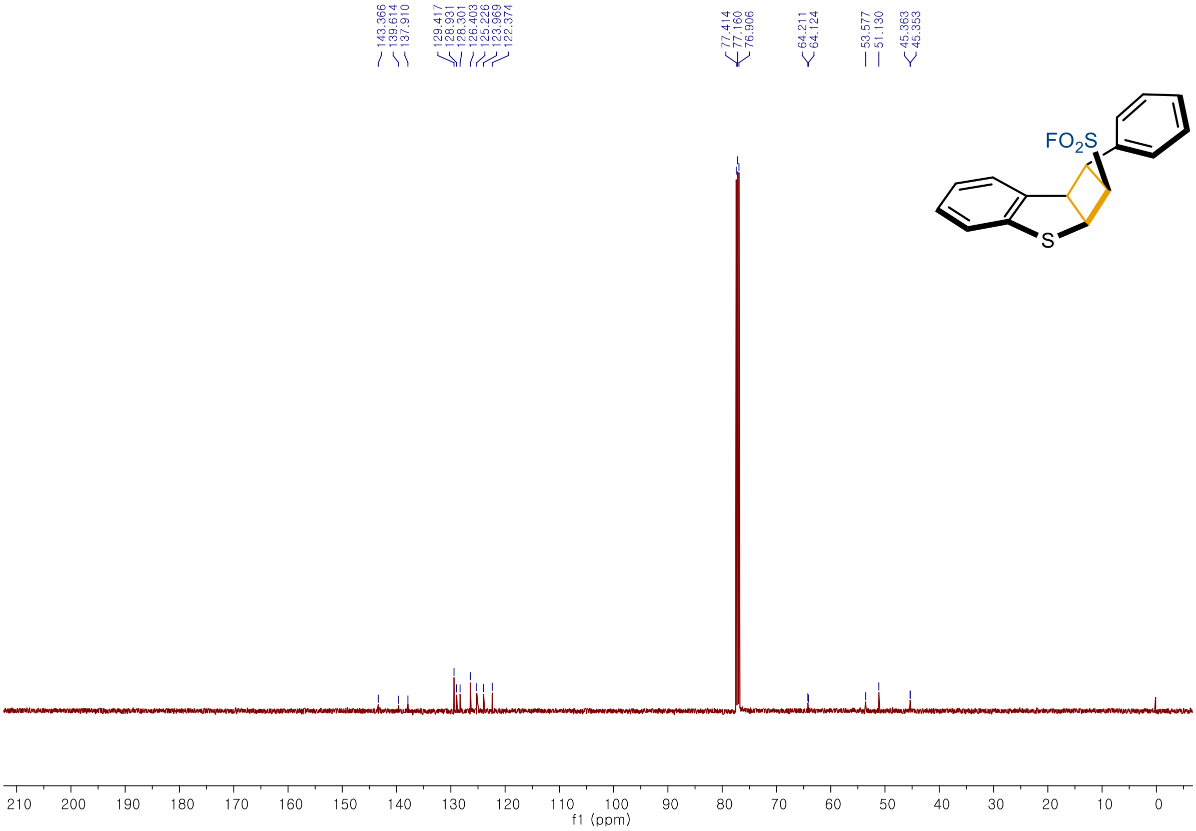

^{13}C NMR spectrum (**3ab**-*endo*)

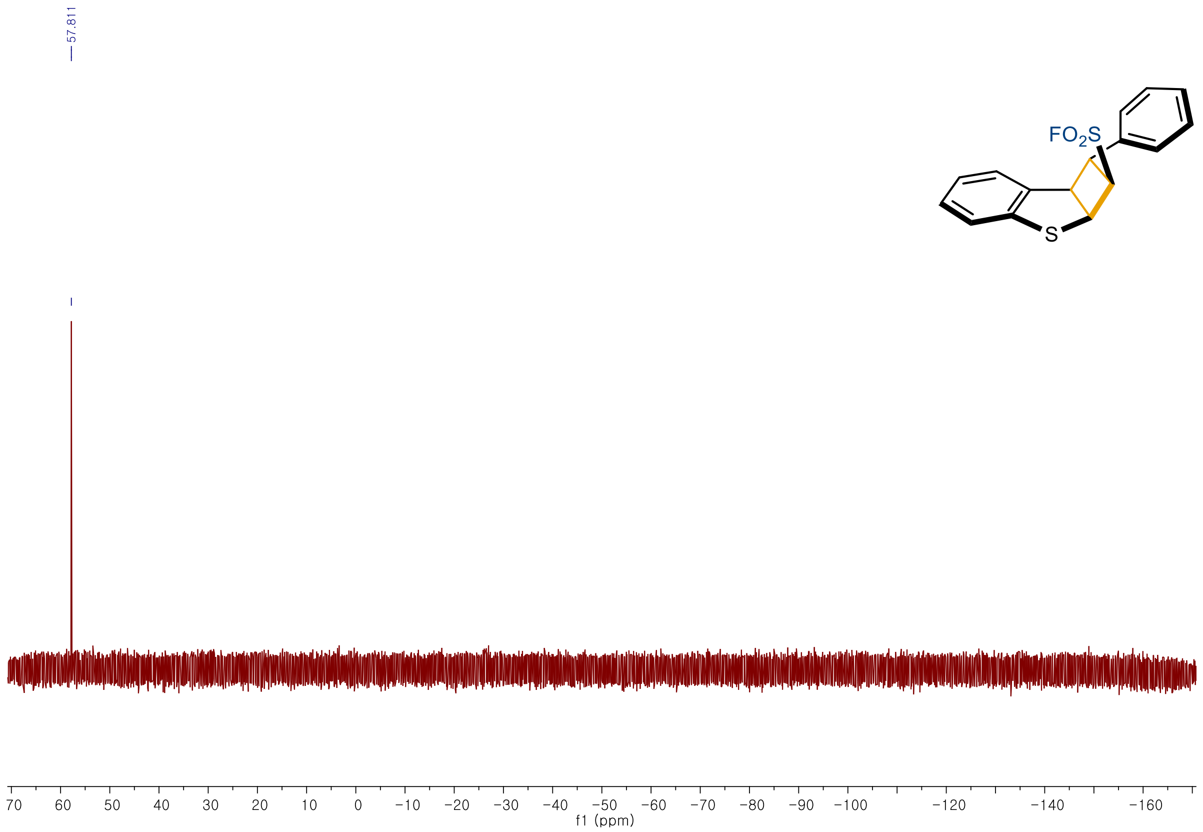

^{19}F NMR spectrum (**3ab**-*endo*)

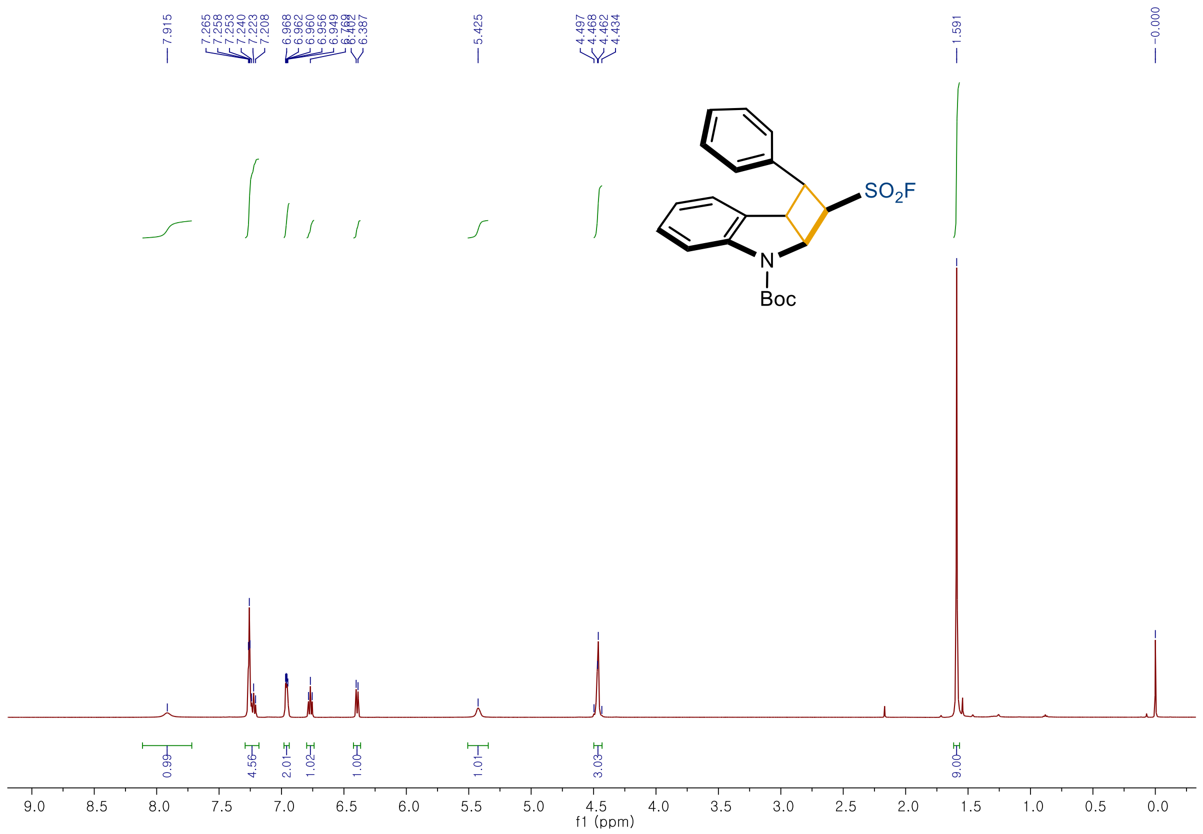

^{1}H NMR spectrum (**3ac**-*exo*)

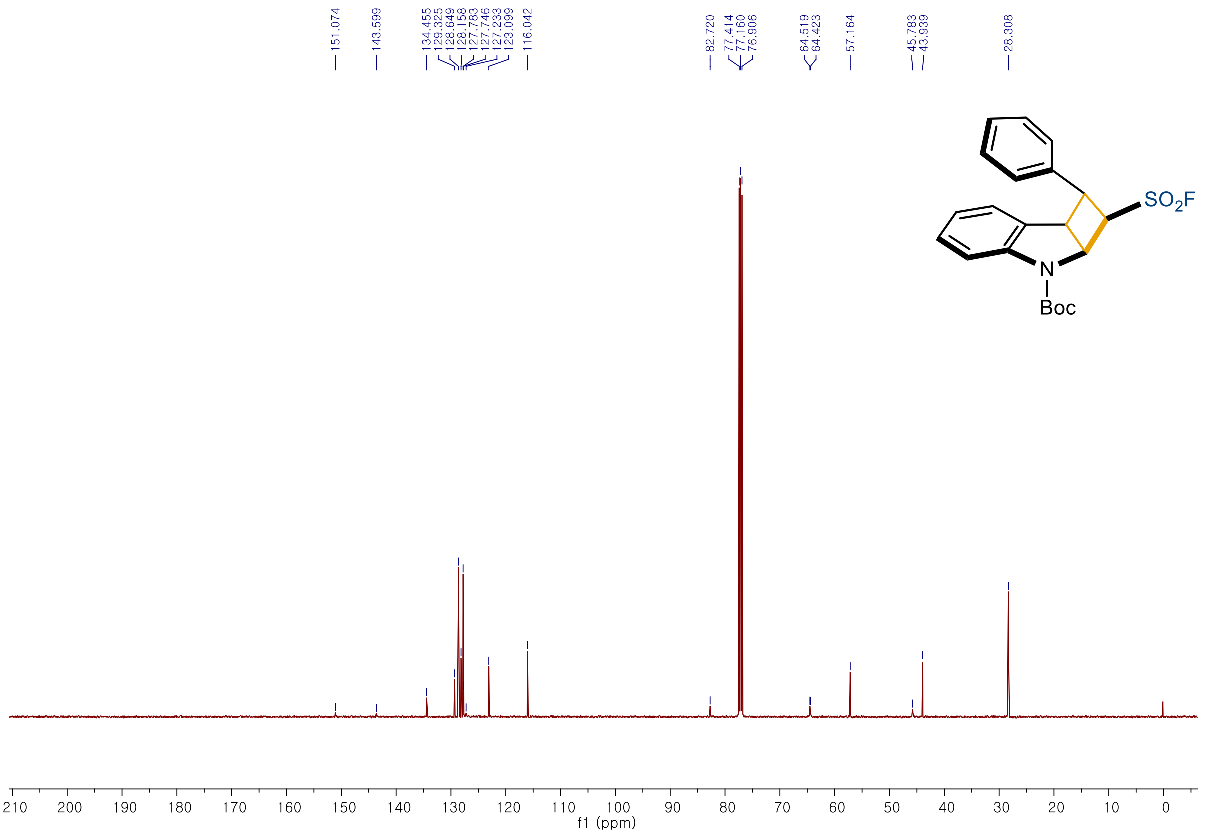

^{13}C NMR spectrum (**3ac**-*exo*)

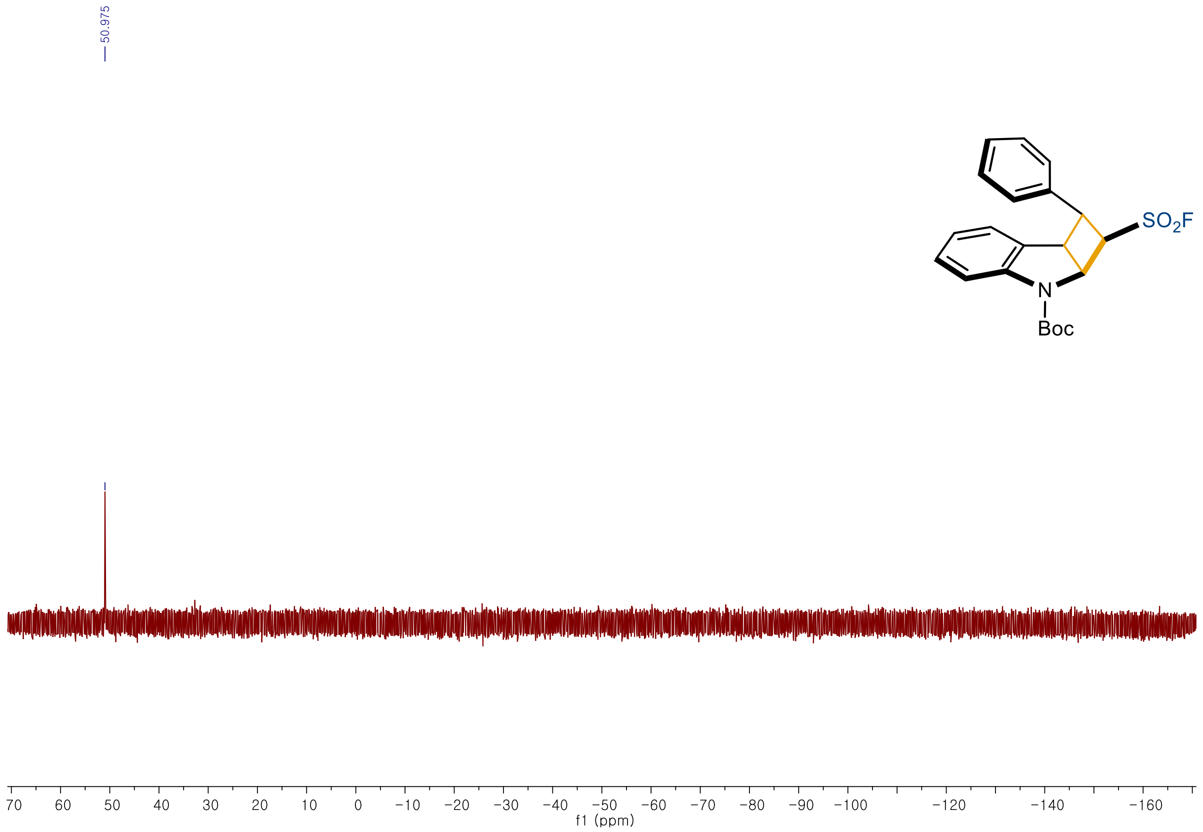

^{19}F NMR spectrum (**3ac**-*exo*)

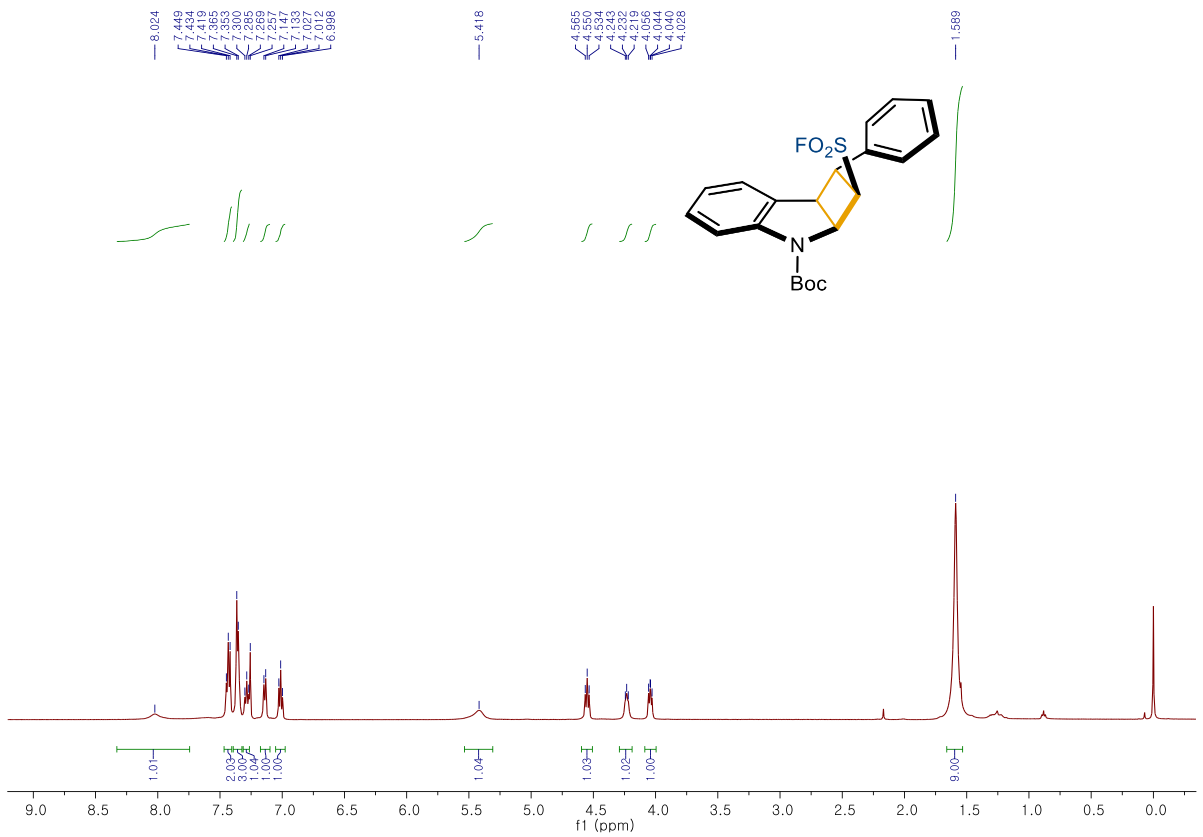

^{1}H NMR spectrum (**3ac**-*endo*)

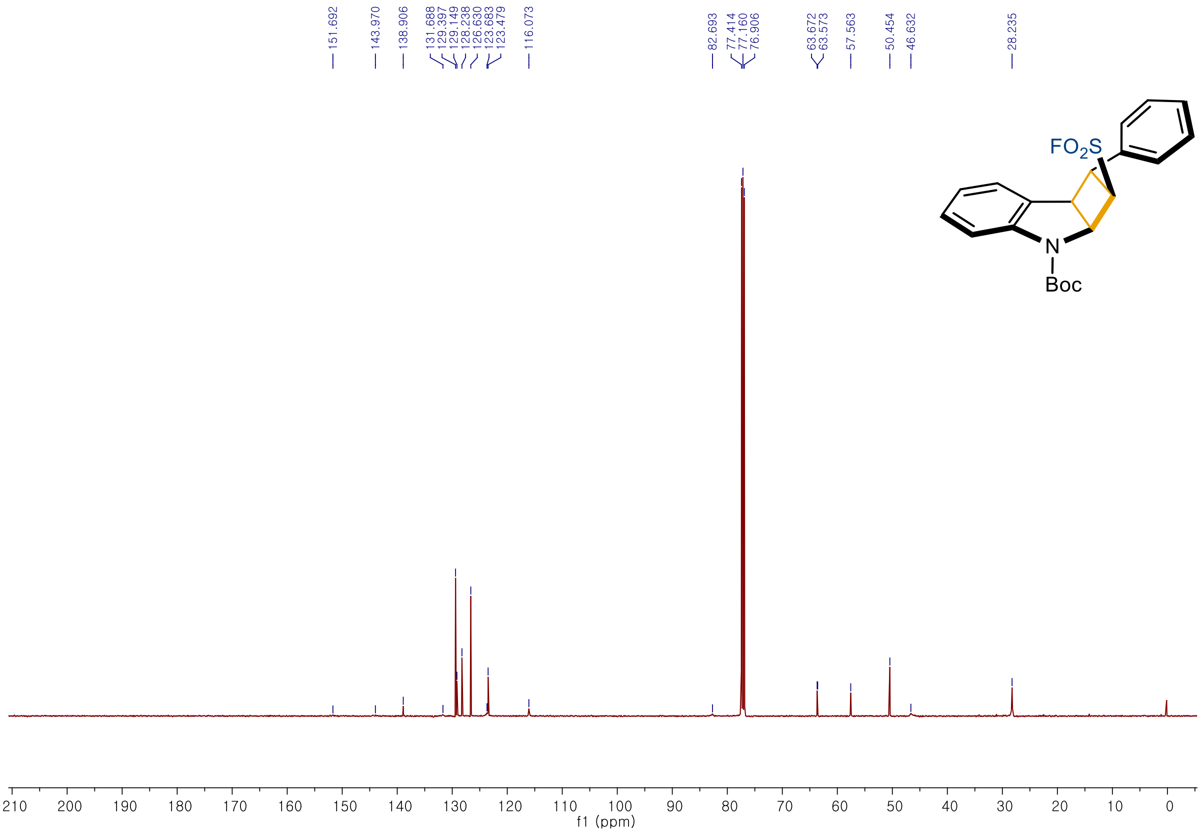

^{13}C NMR spectrum (**3ac**-*endo*)

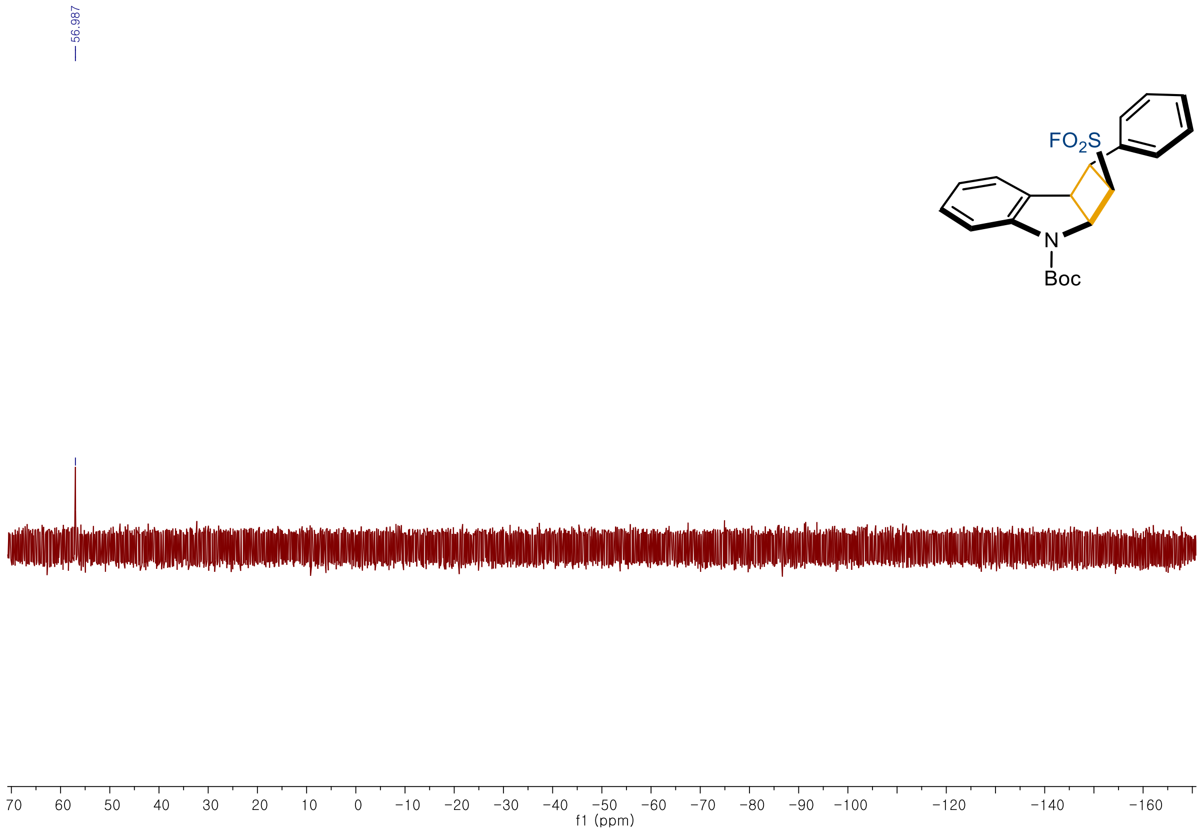

^{19}F NMR spectrum (**3ac**-*endo*)

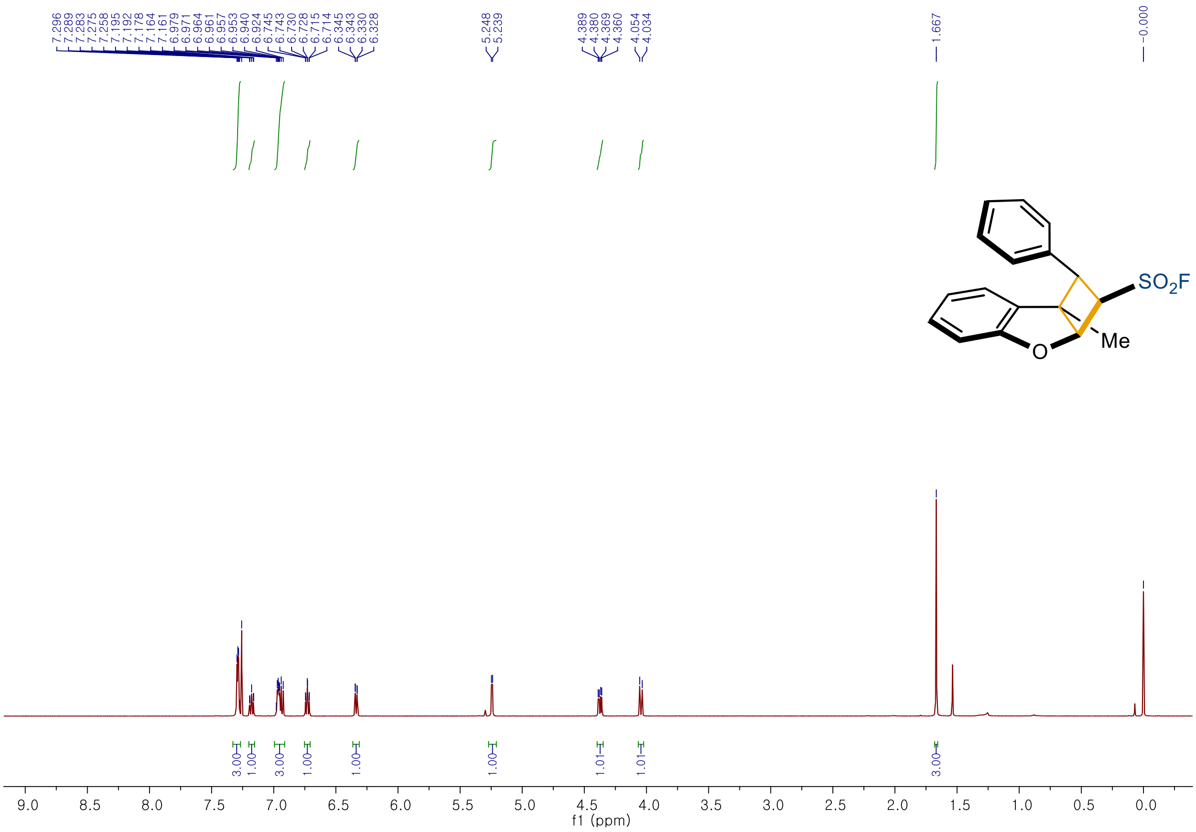

^{1}H NMR spectrum (**3ad**-*exo*)

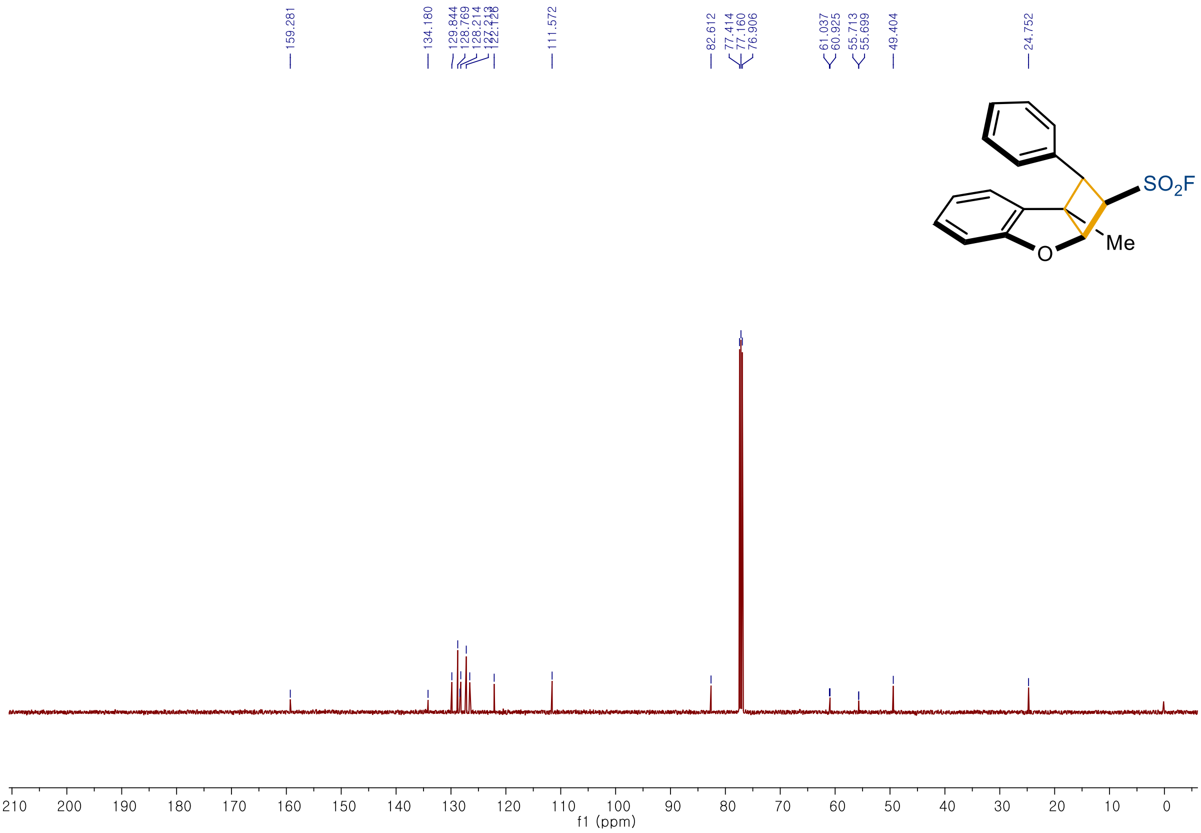

^{13}C NMR spectrum (**3ad**-*exo*)

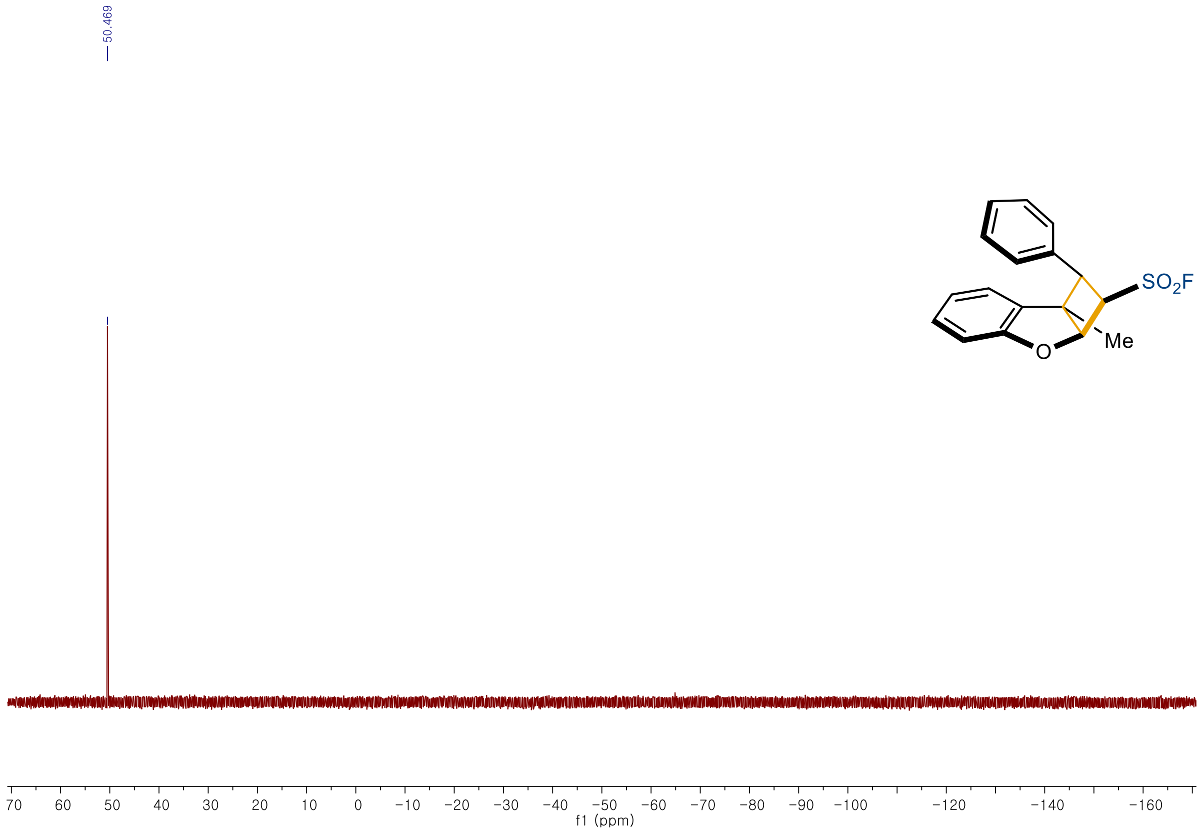

^{19}F NMR spectrum (**3ad**-*exo*)

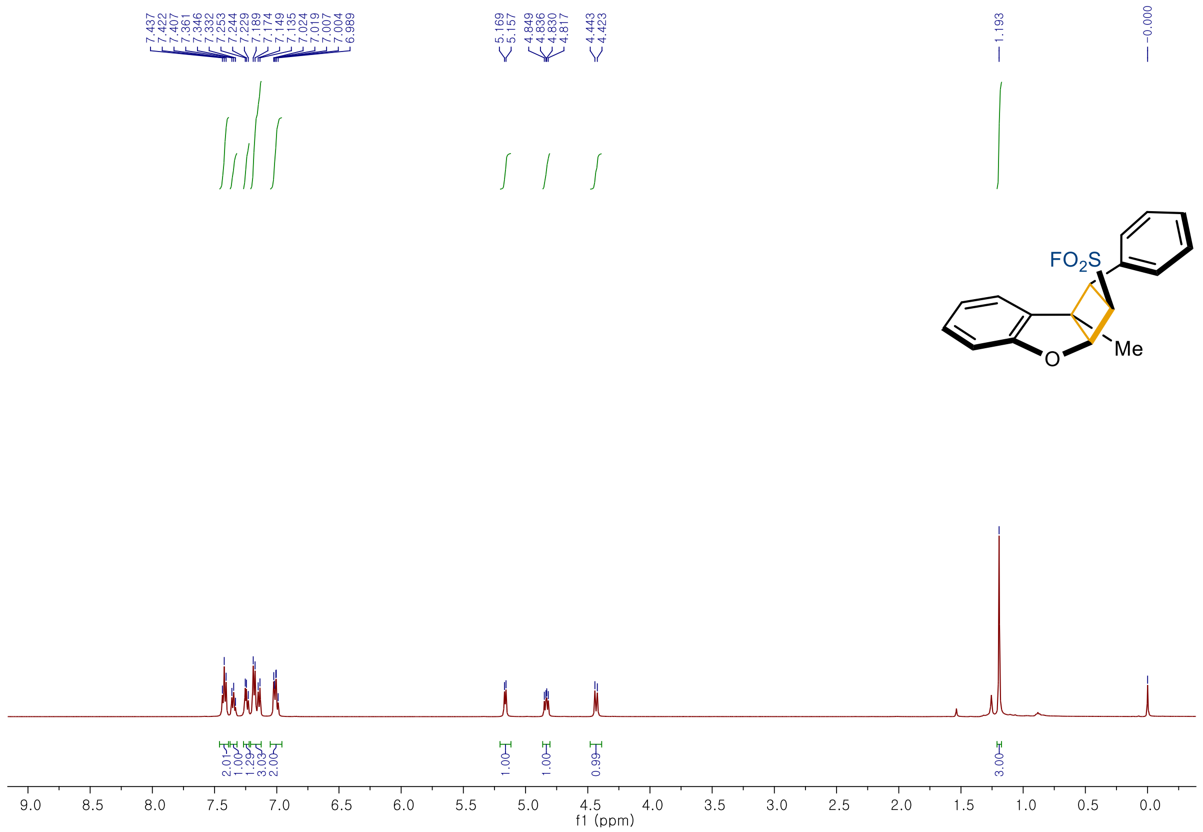

^{1}H NMR spectrum (**3ad**-*endo*)

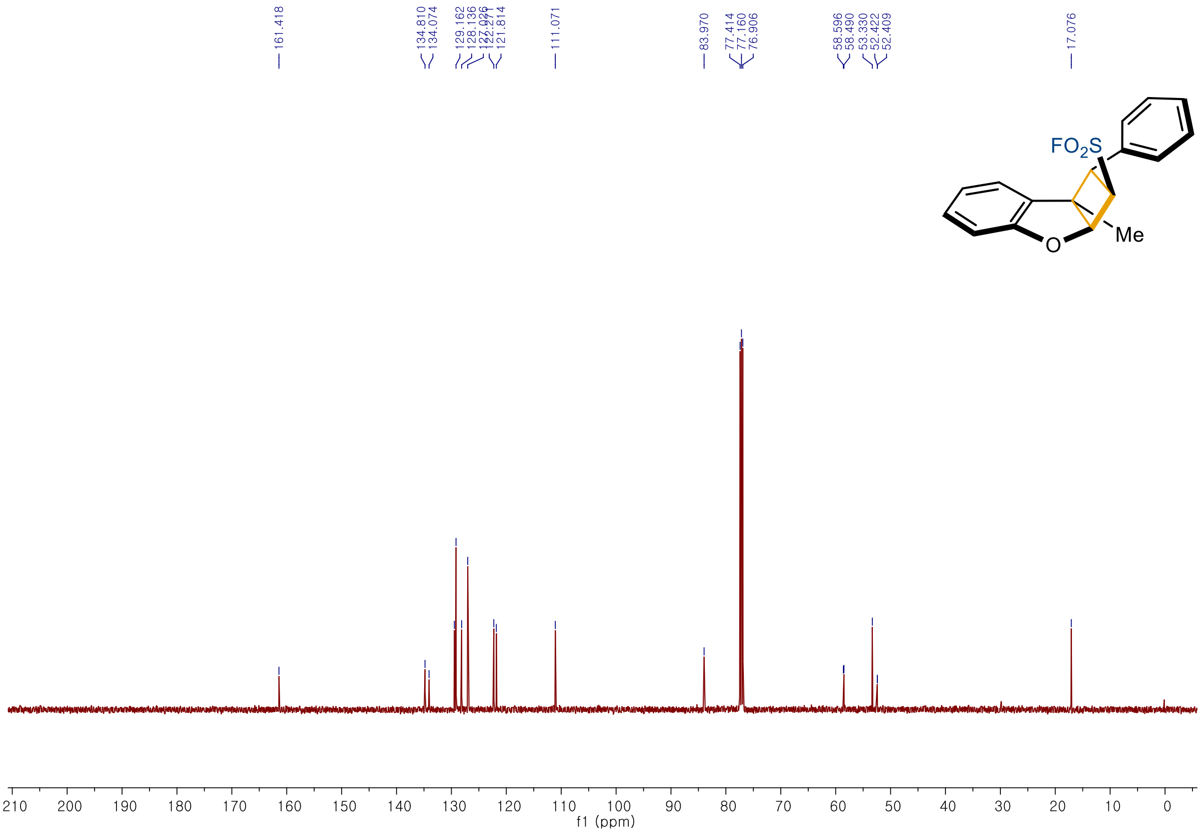

b

^{13}C NMR spectrum (**3ad**-*endo*)

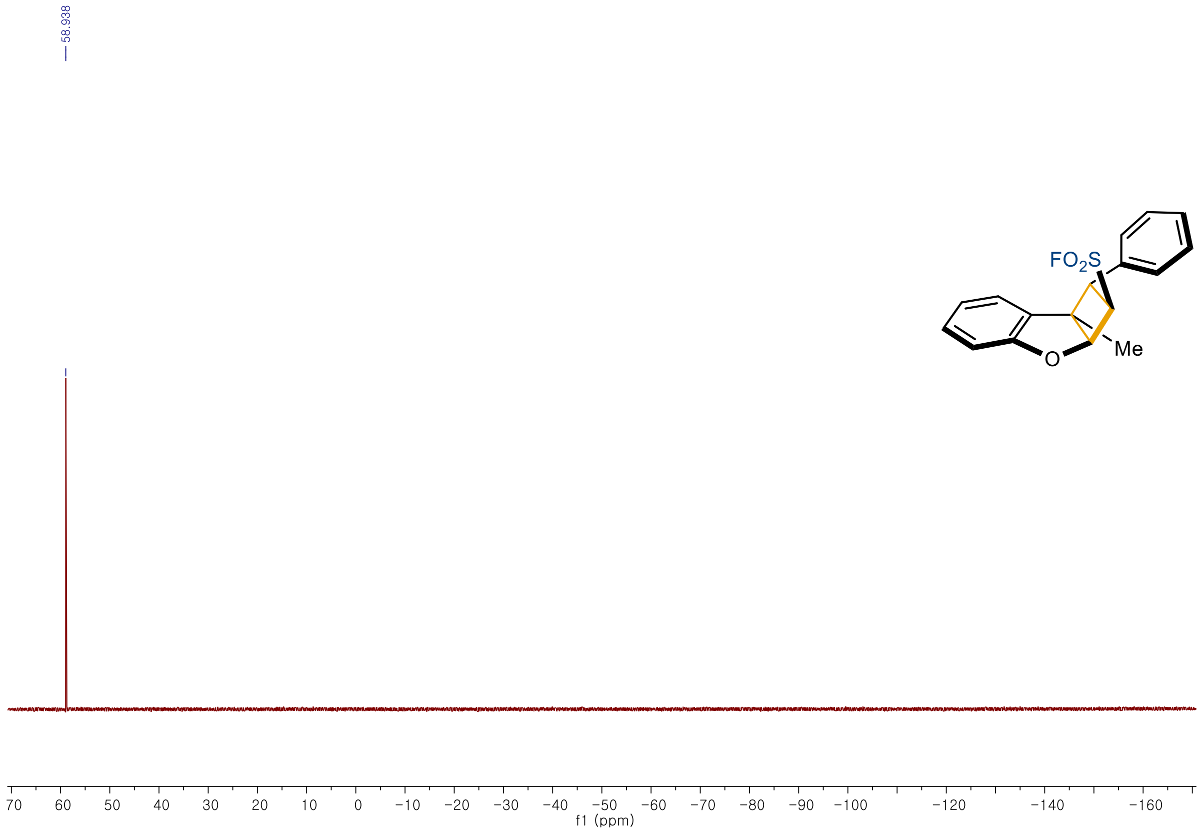

^{19}F NMR spectrum (**3ad**-*endo*)

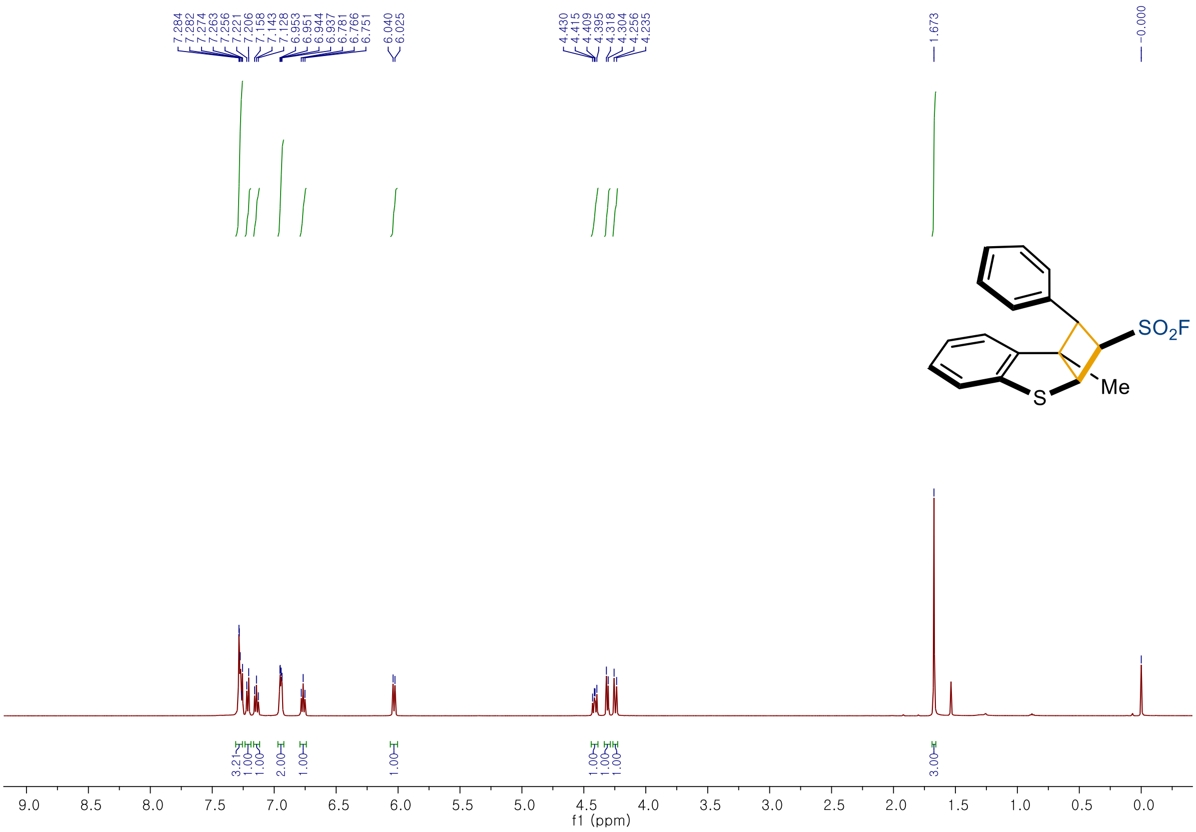

^{1}H NMR spectrum (**3ae**-*exo*)

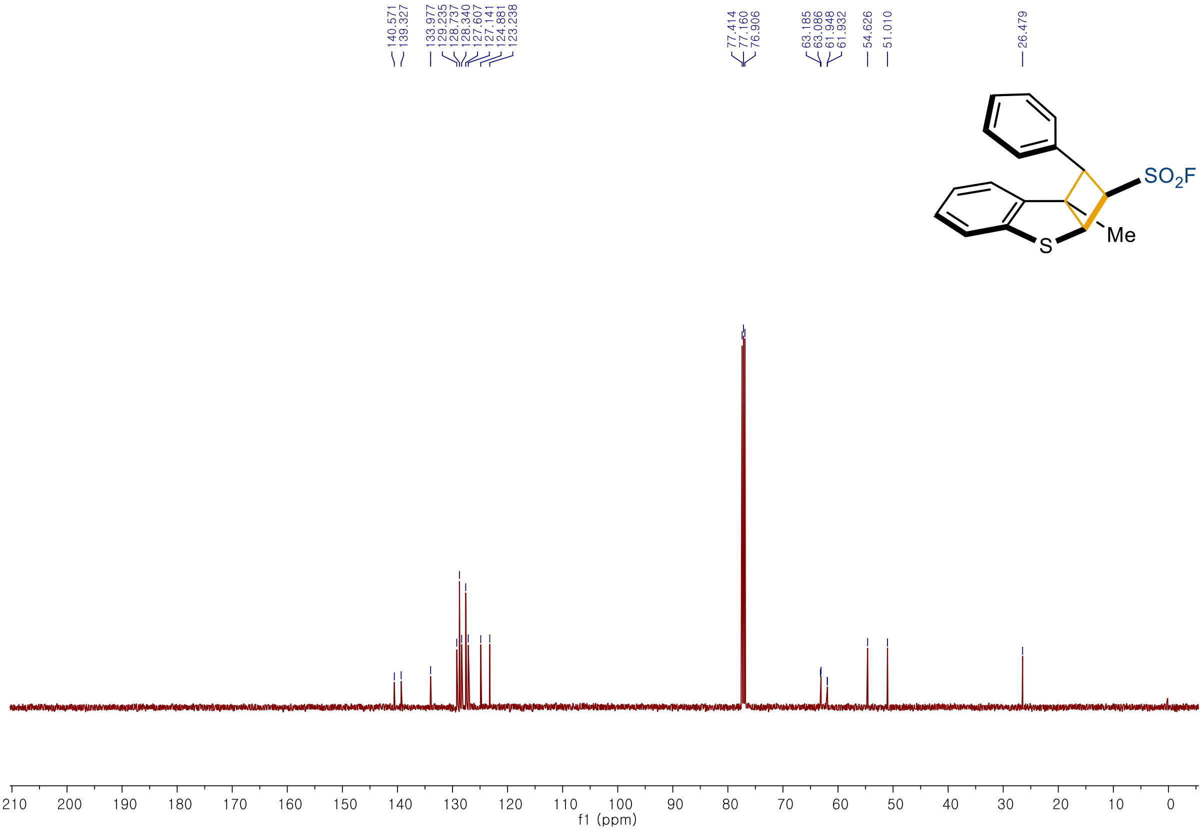

^{13}C NMR spectrum (**3ae**-*exo*)

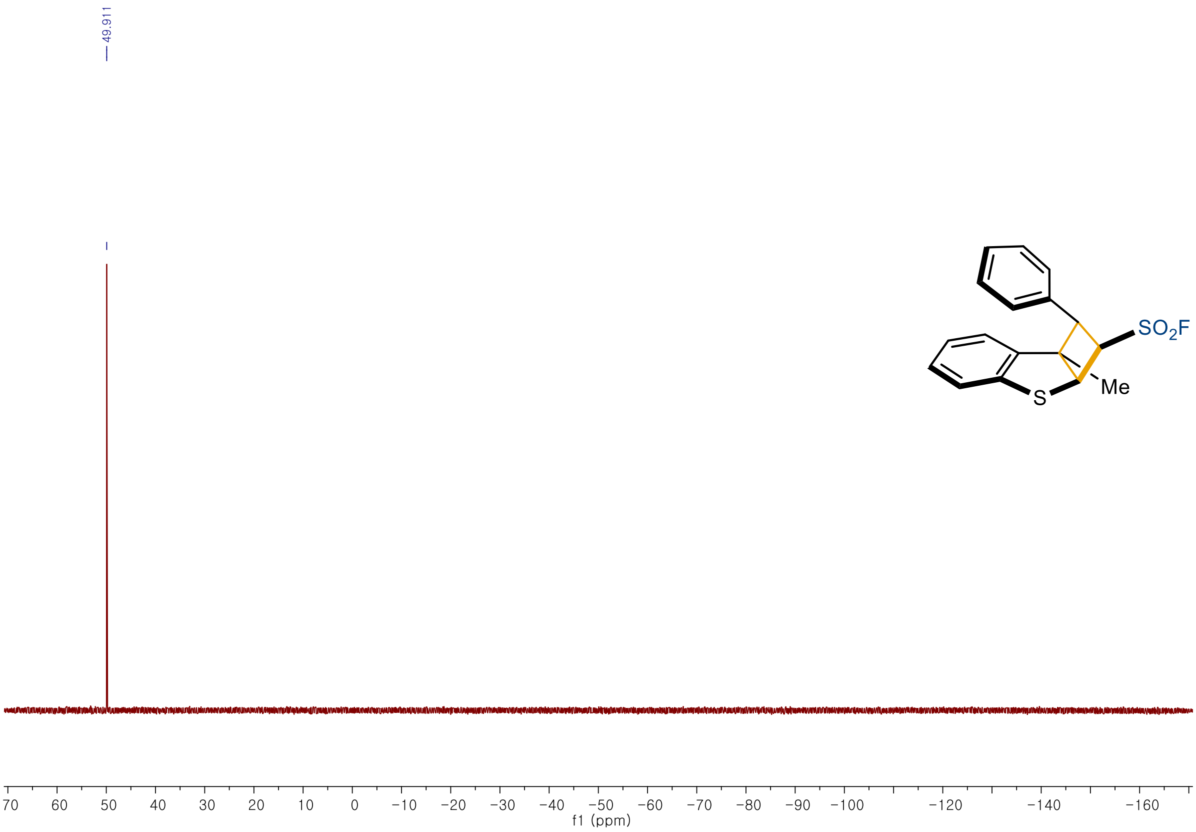

^{19}F NMR spectrum (**3ae**-*exo*)

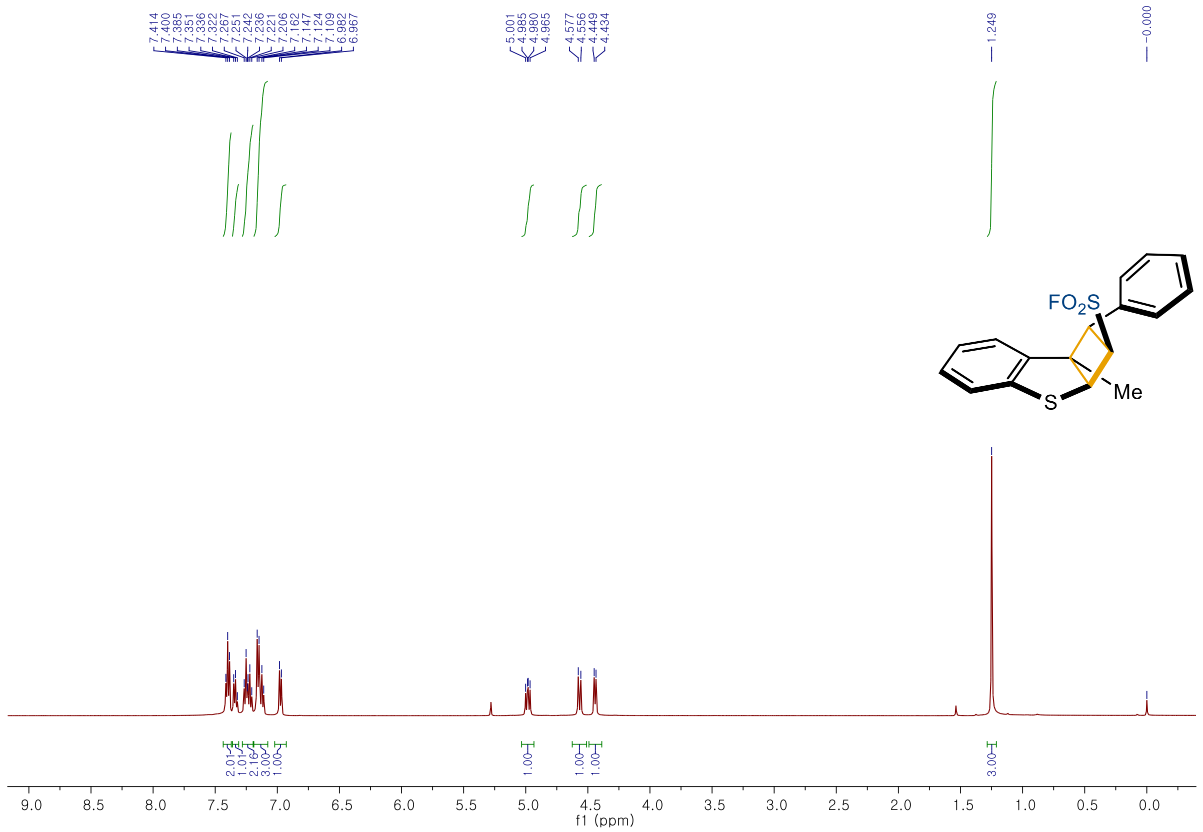

^{1}H NMR spectrum (**3ae**-*endo*)

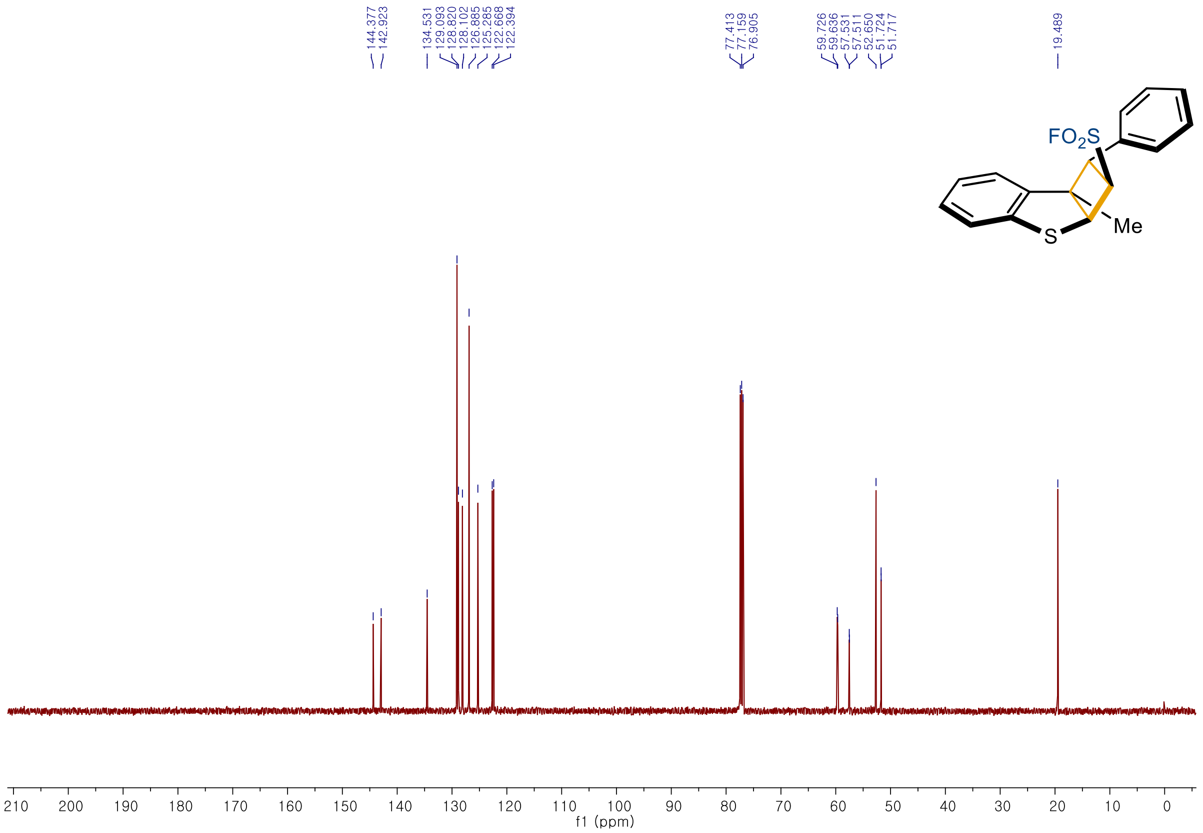

^{13}C NMR spectrum (**3ae**-*endo*)

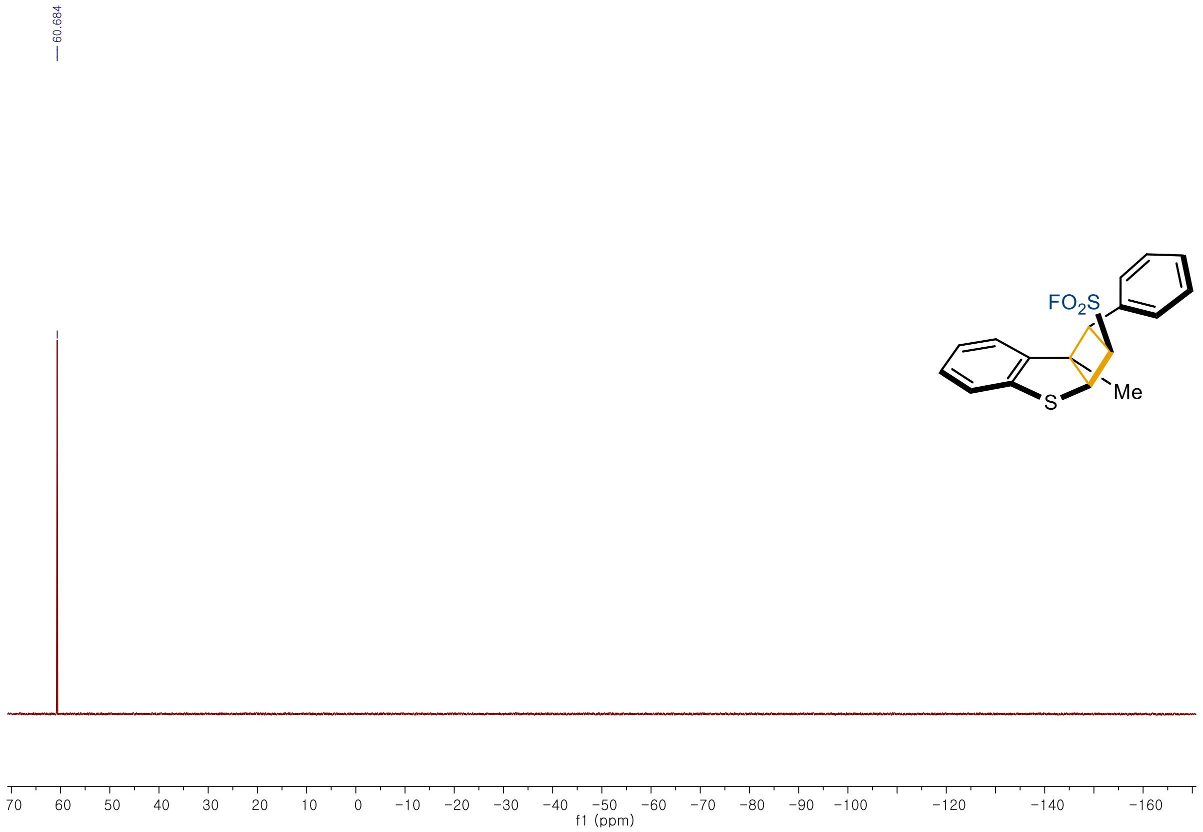

^{19}F NMR spectrum (**3ae**-*endo*)

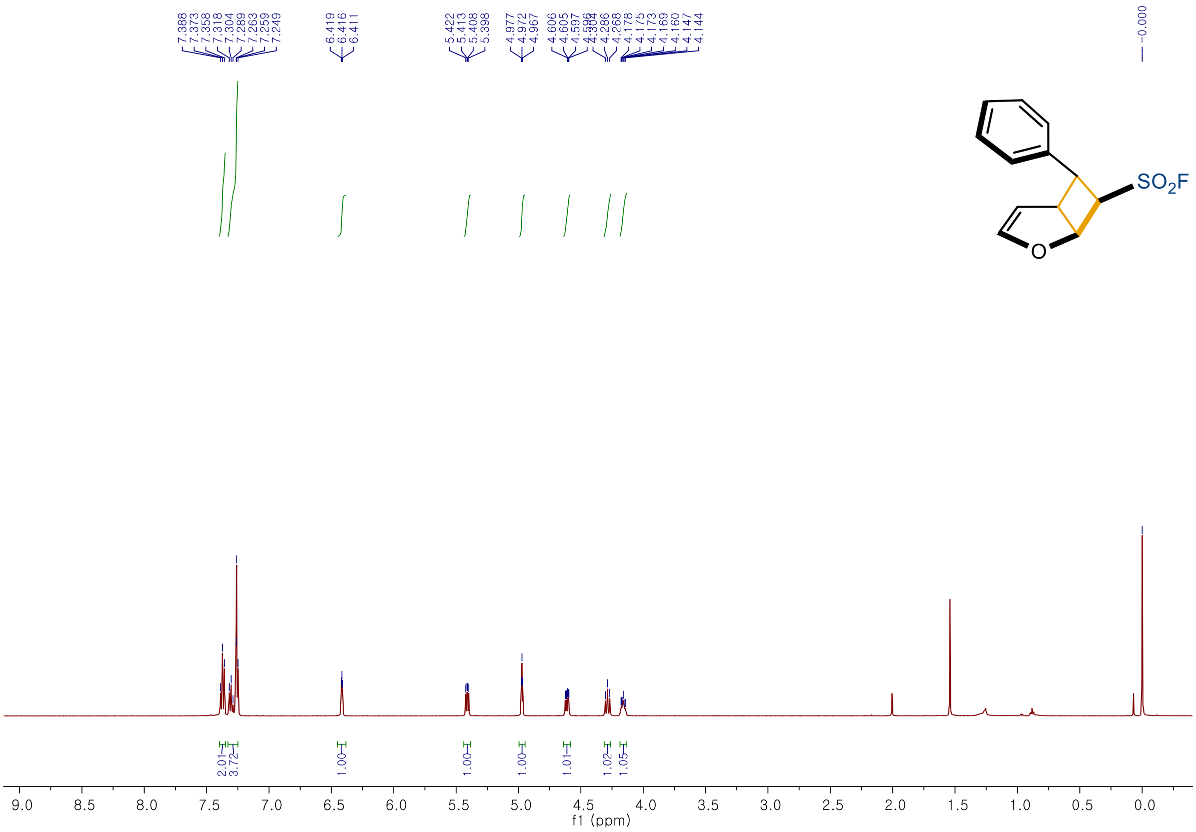

^{1}H NMR spectrum (**3af**-*exo*)

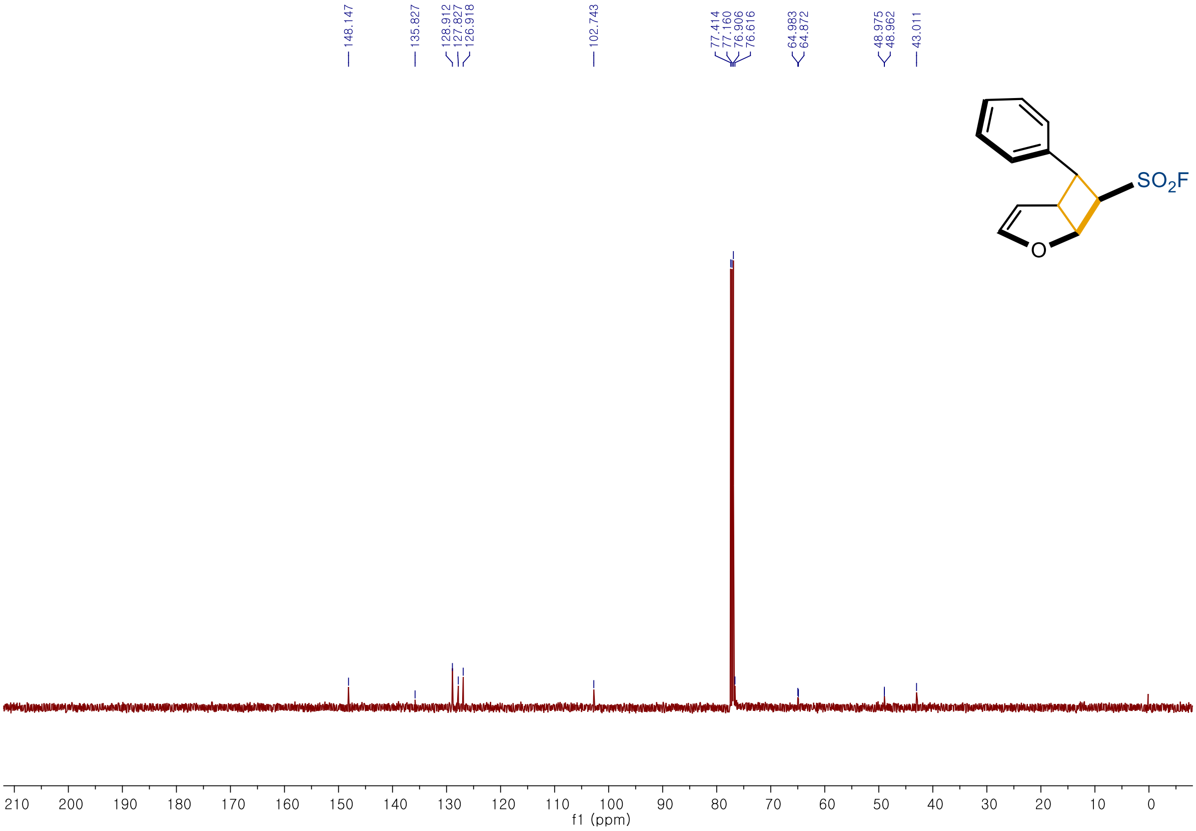

^{13}C NMR spectrum (**3af**-*exo*)

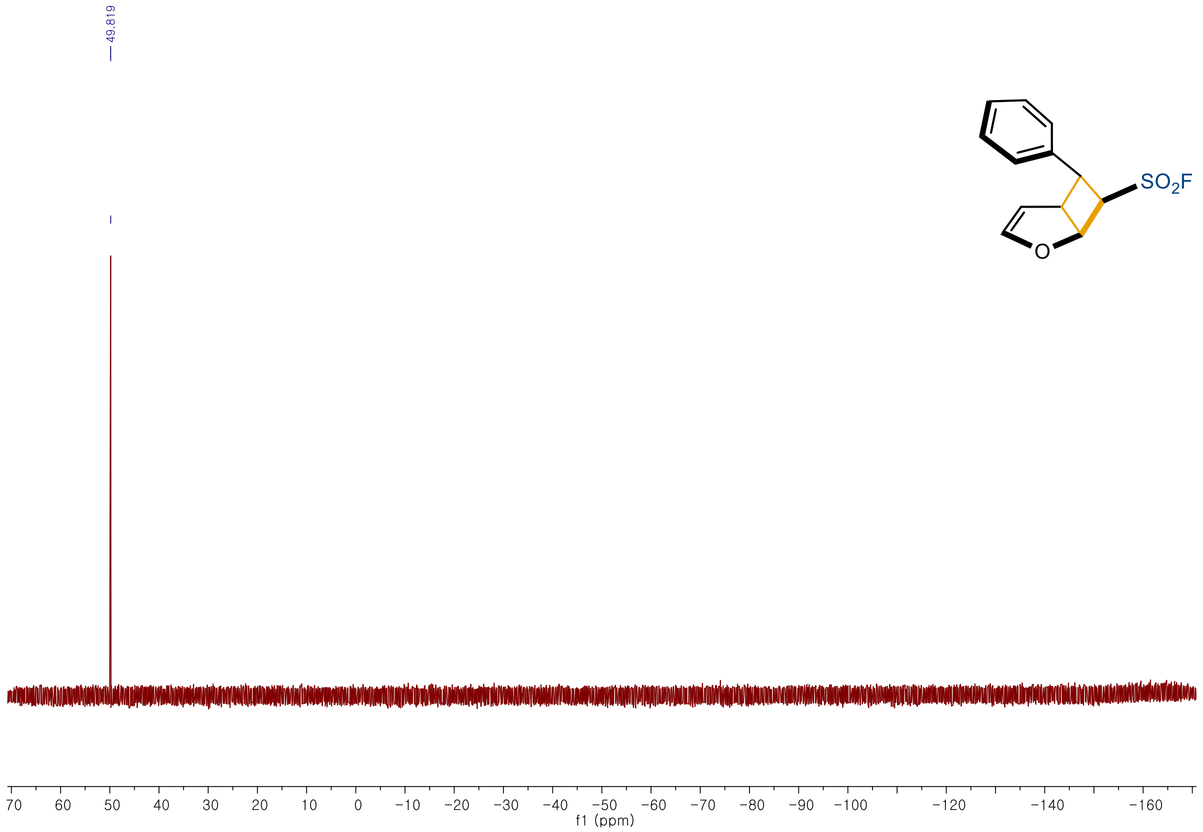

^{19}F NMR spectrum (**3af**-*exo*)

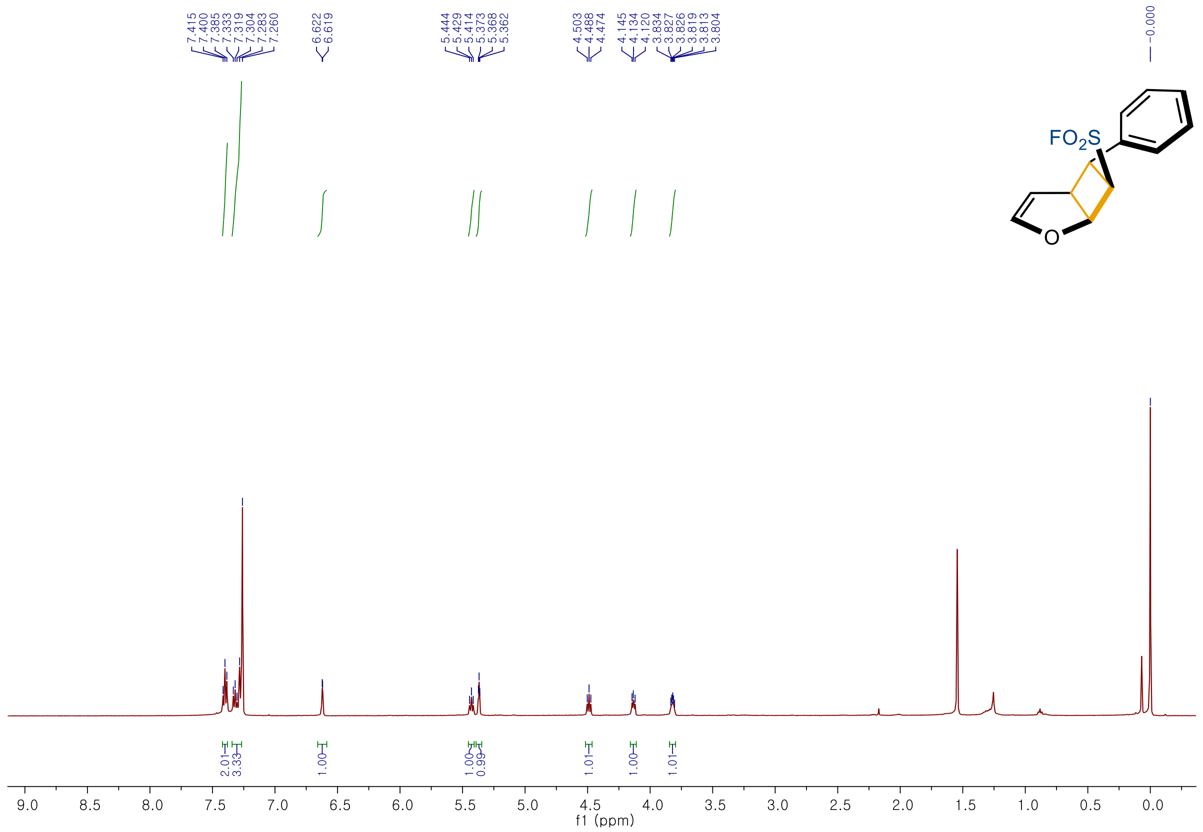

^{1}H NMR spectrum (**3af**-*endo*)

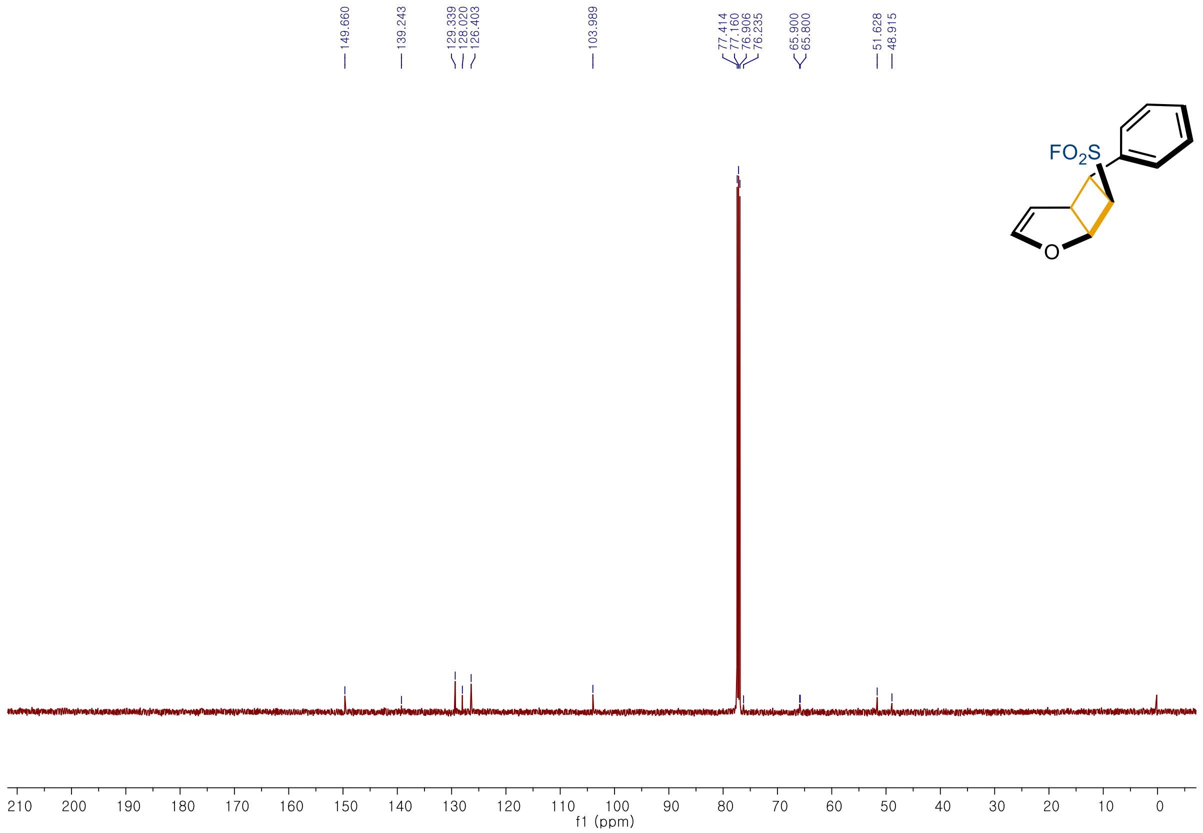

^{13}C NMR spectrum (**3af**-*endo*)

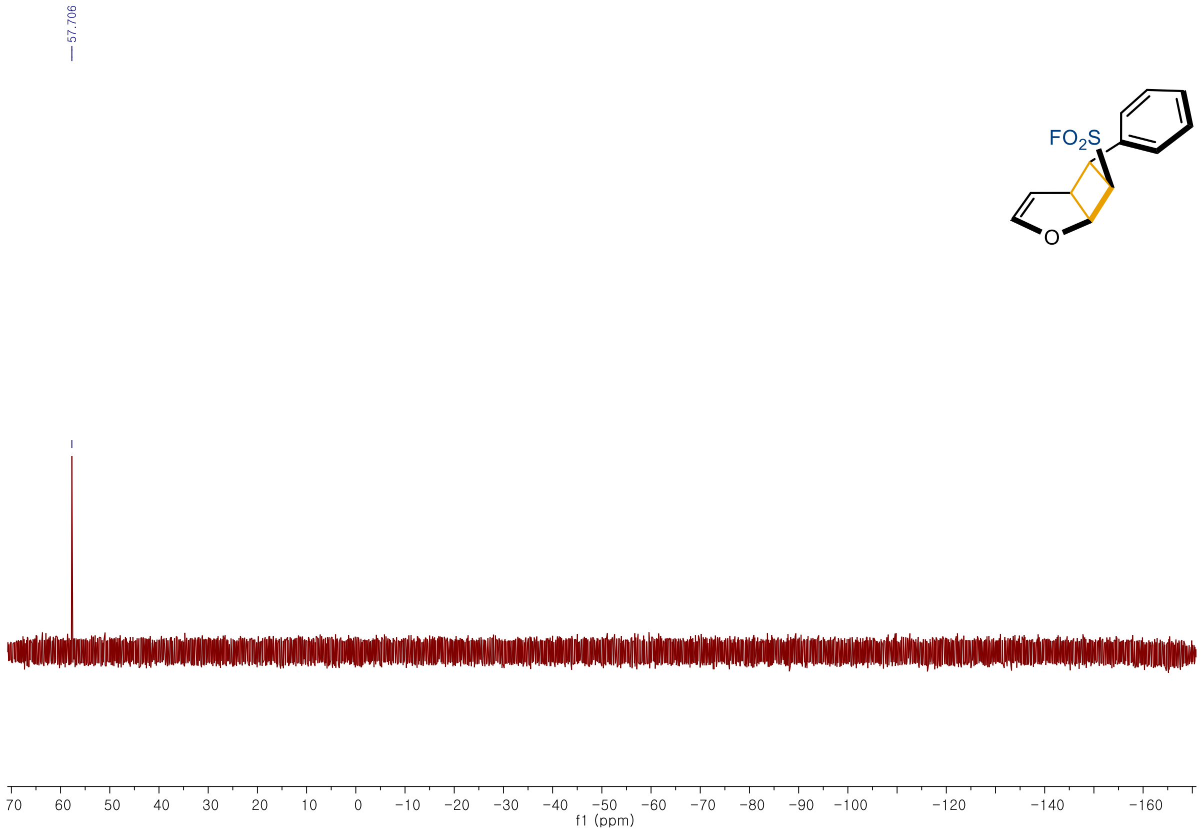

^{19}F NMR spectrum (**3af**-*endo*)

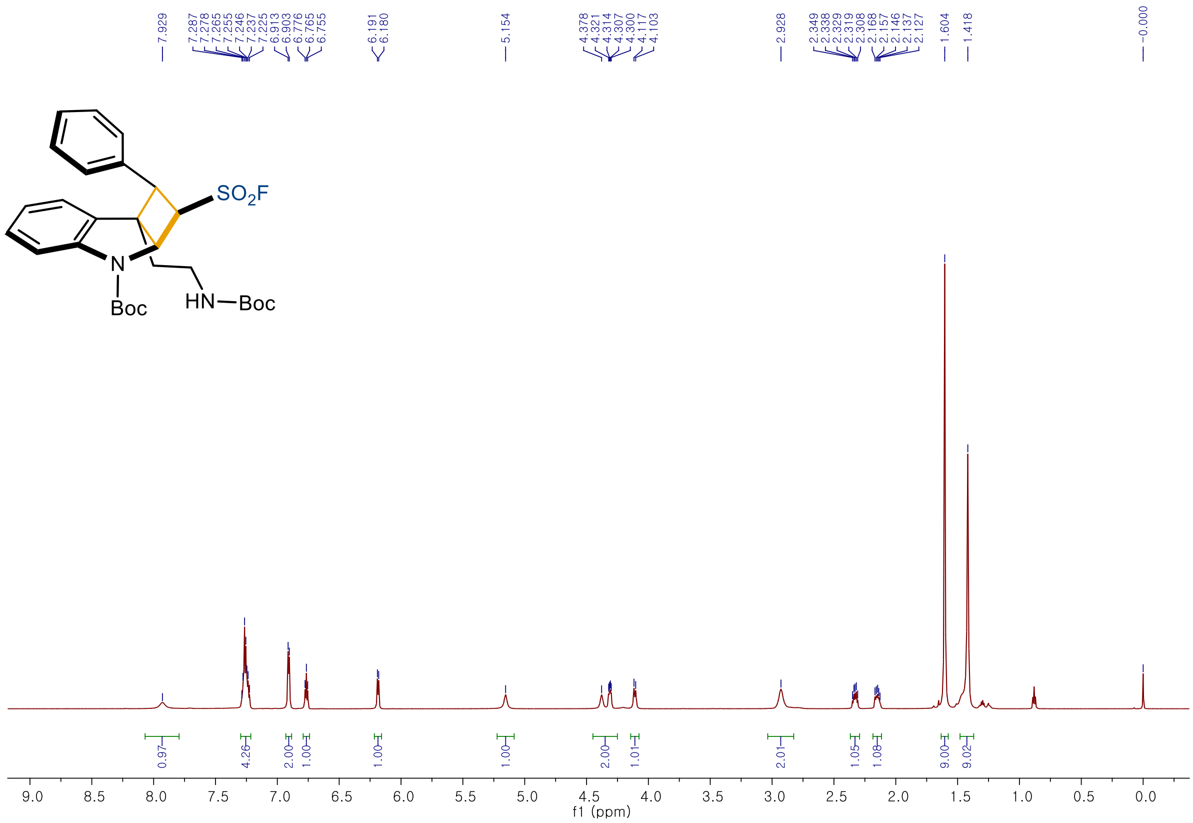

^{1}H NMR spectrum (**3ag**-*exo*)

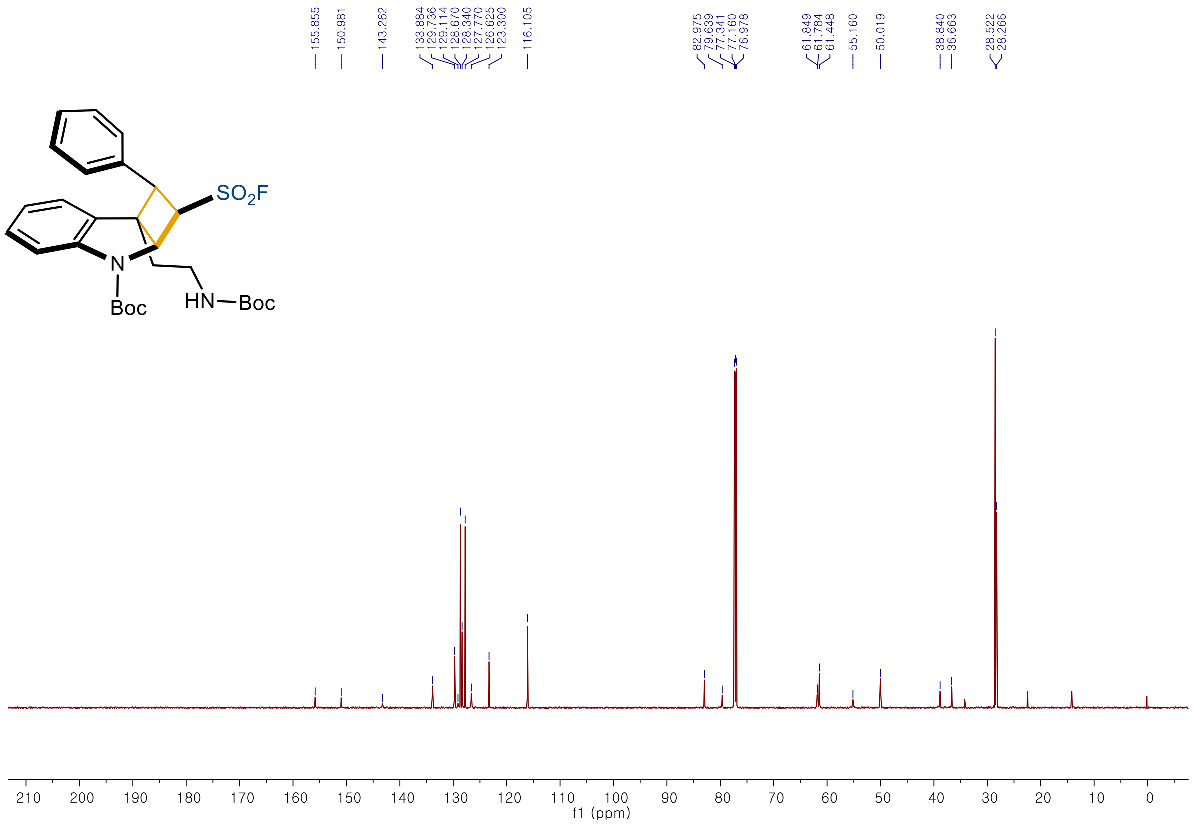

^{13}C NMR spectrum (**3ag**-*exo*)

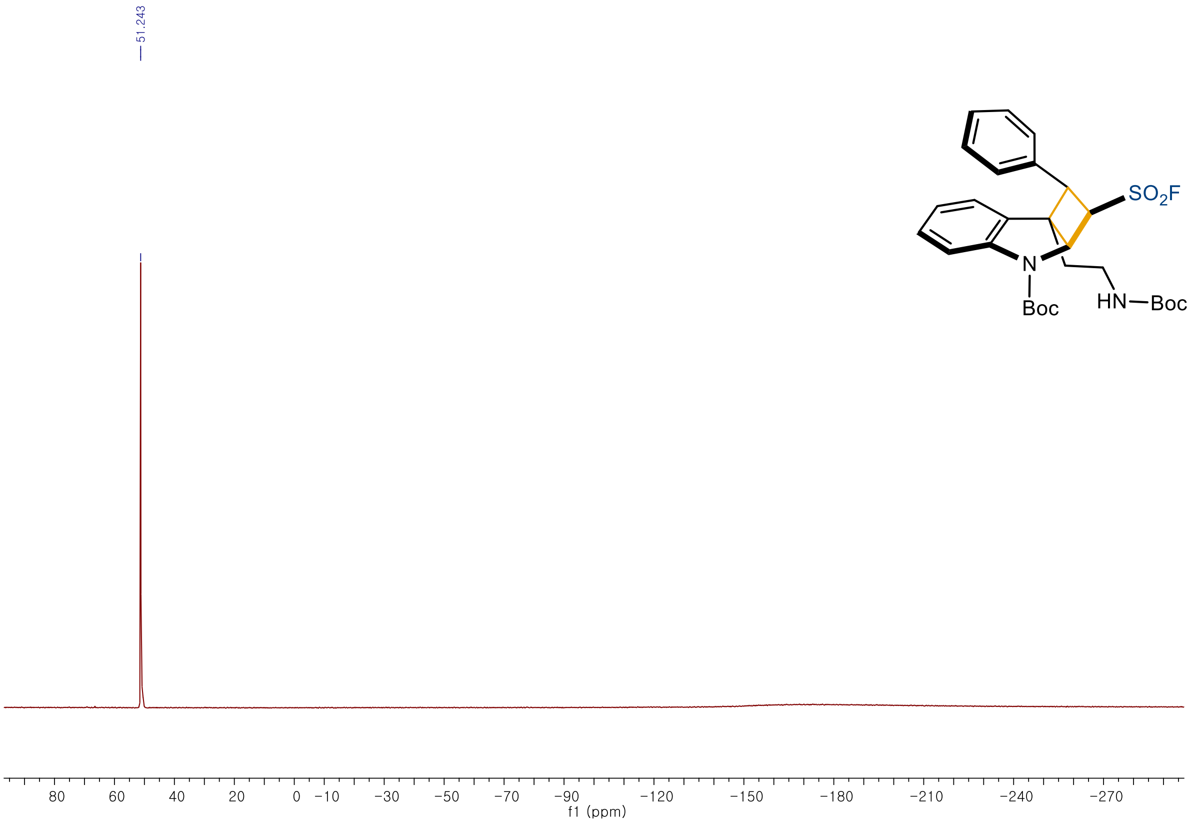

^{19}F NMR spectrum (**3ag**-*exo*)

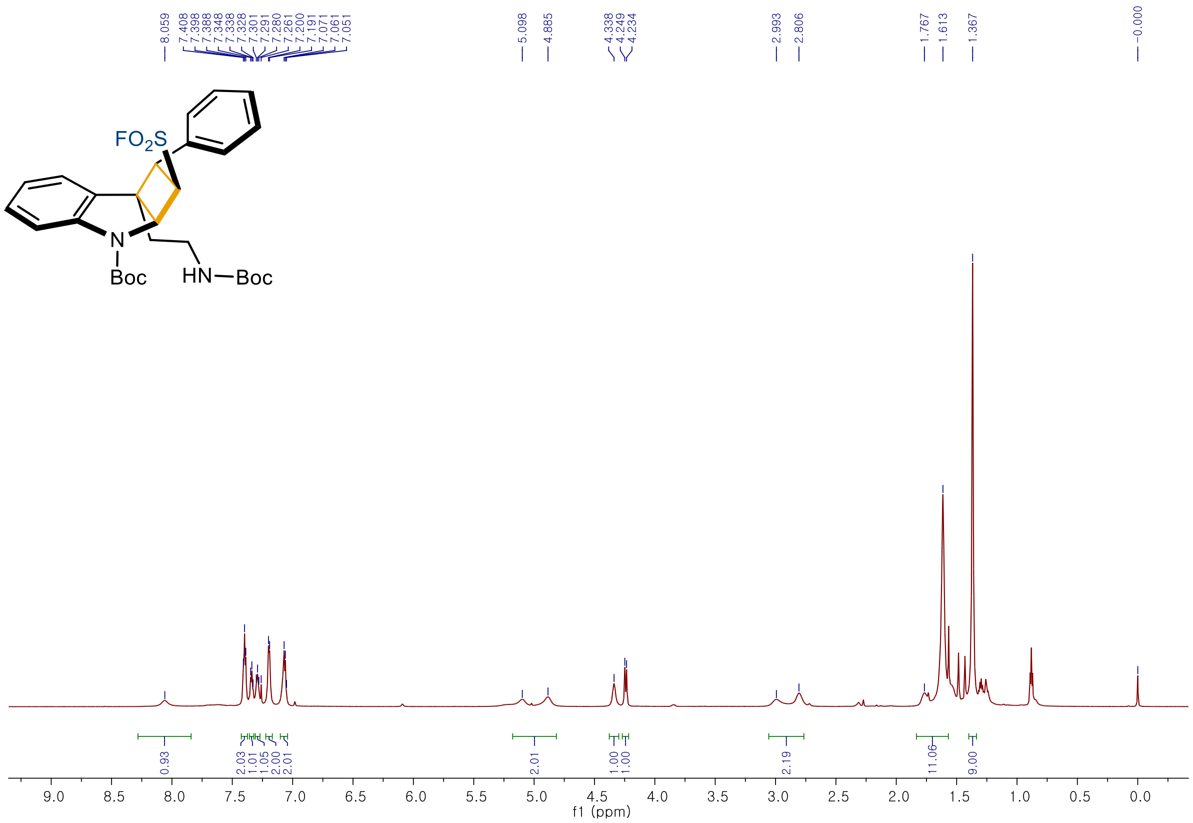

^{1}H NMR spectrum (**3ag**-*endo*)

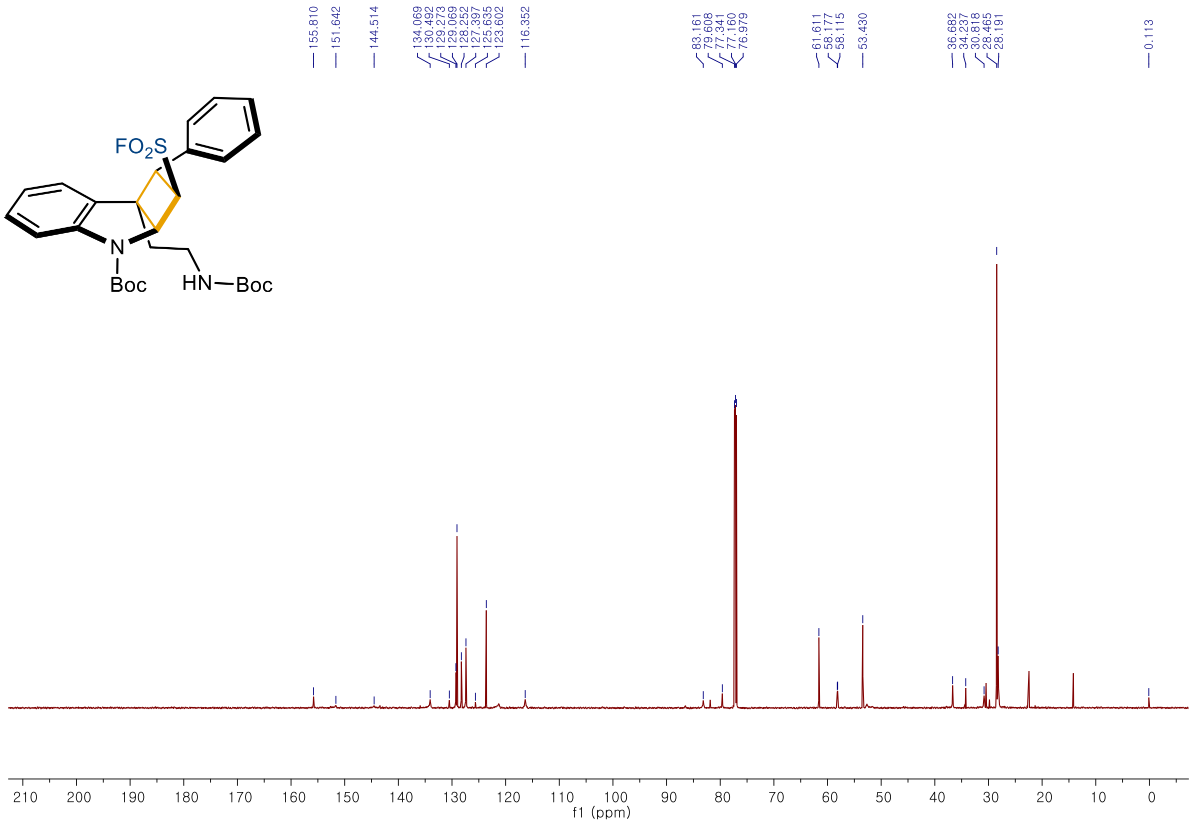

^{13}C NMR spectrum (**3ag**-*endo*)

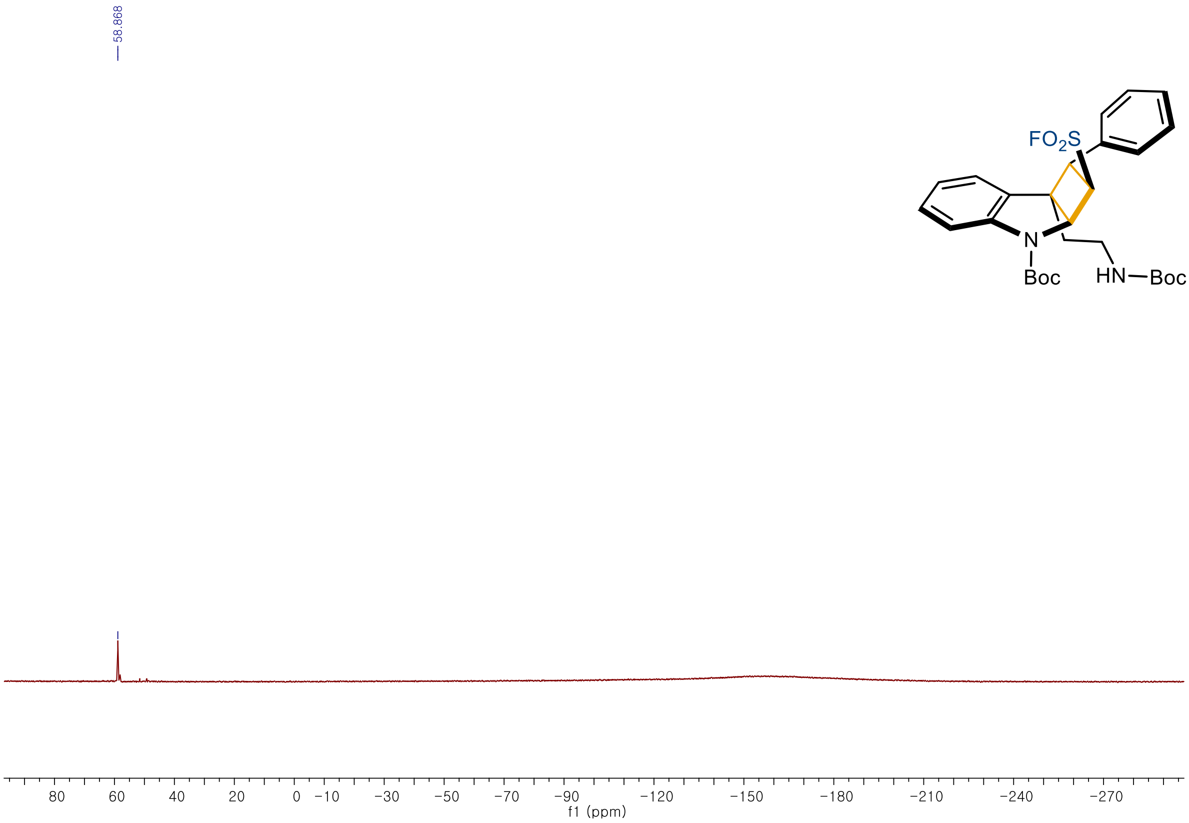

^{19}F NMR spectrum (**3ag**-*endo*)

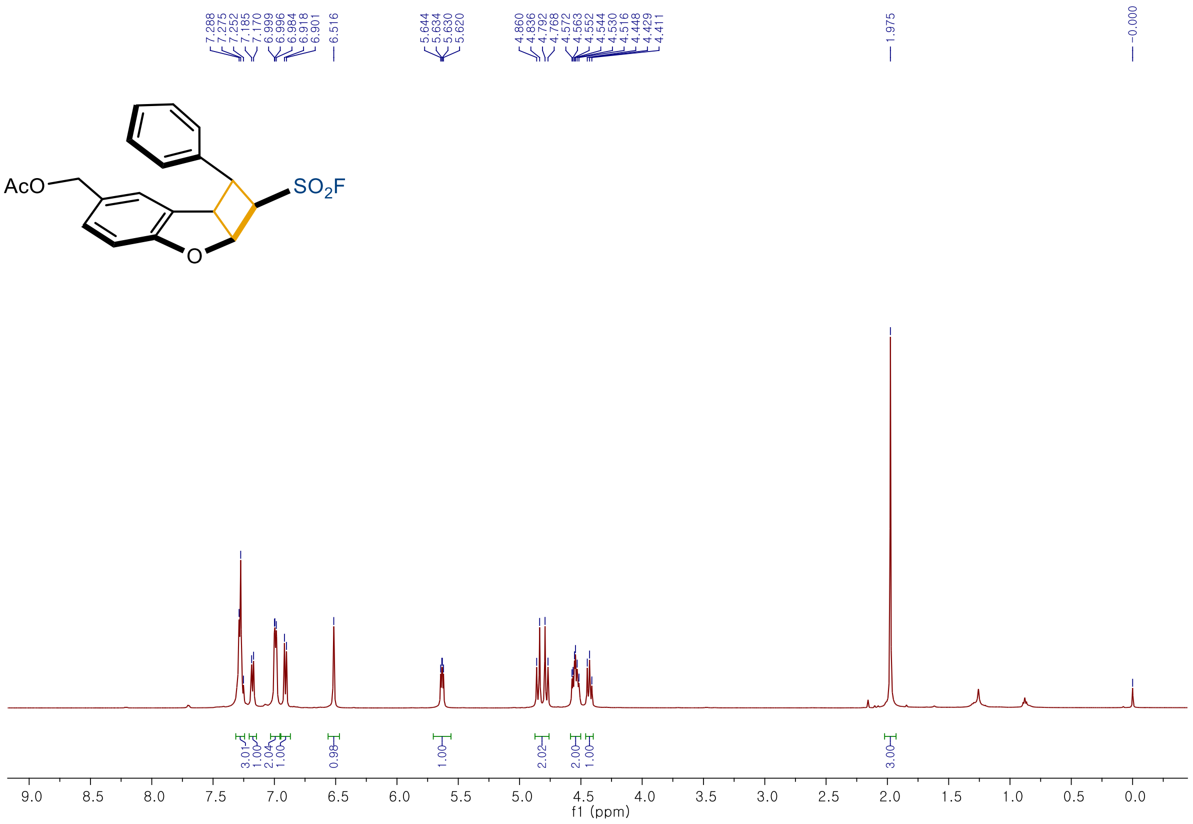

^{1}H NMR spectrum (**3ah**-*exo*)

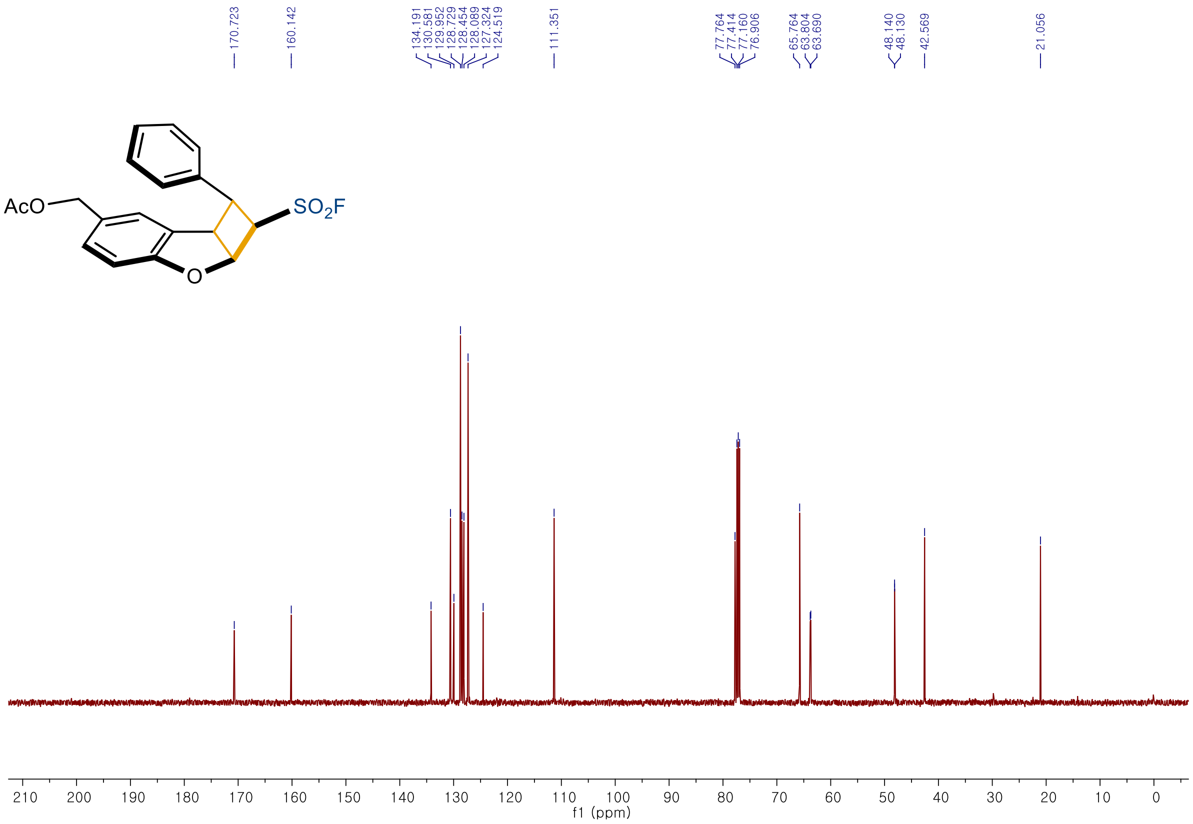

^{13}C NMR spectrum (**3ah**-*exo*)

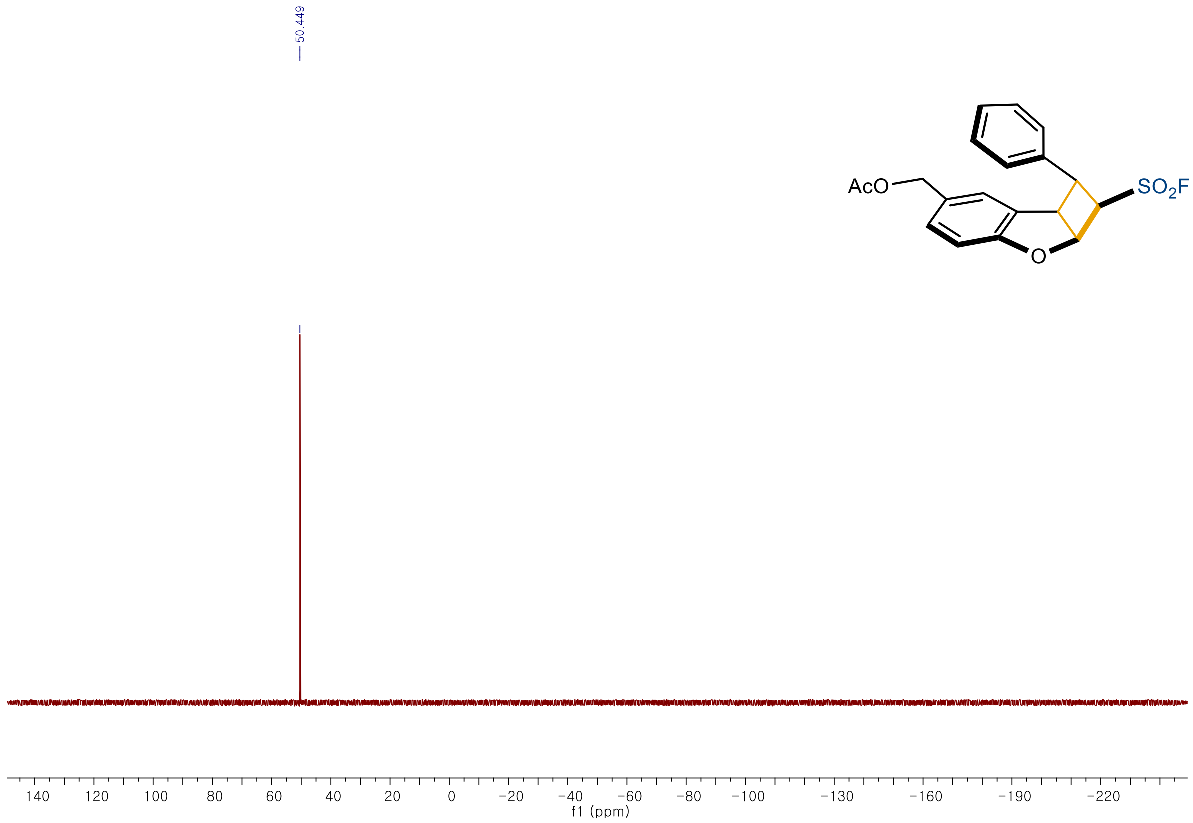

^{19}F NMR spectrum (**3ah**-*exo*)

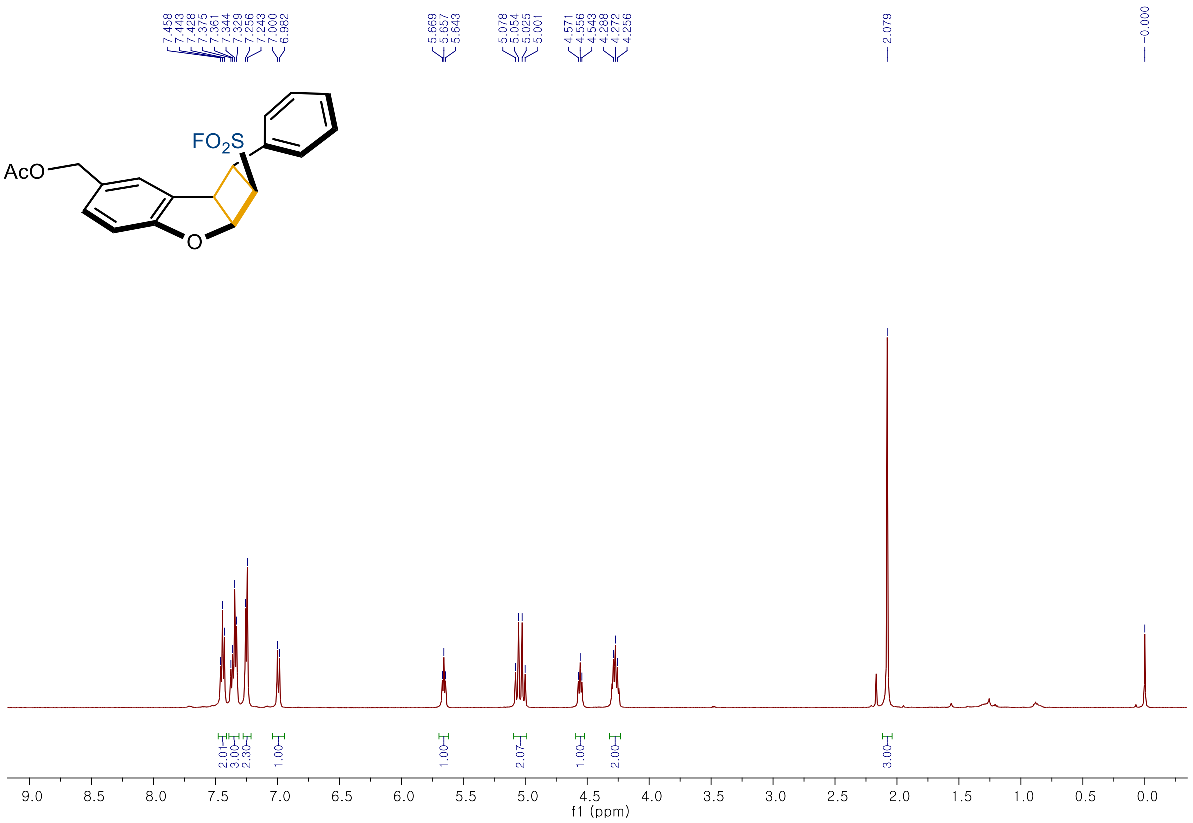

^{1}H NMR spectrum (**3ah**-*endo*)

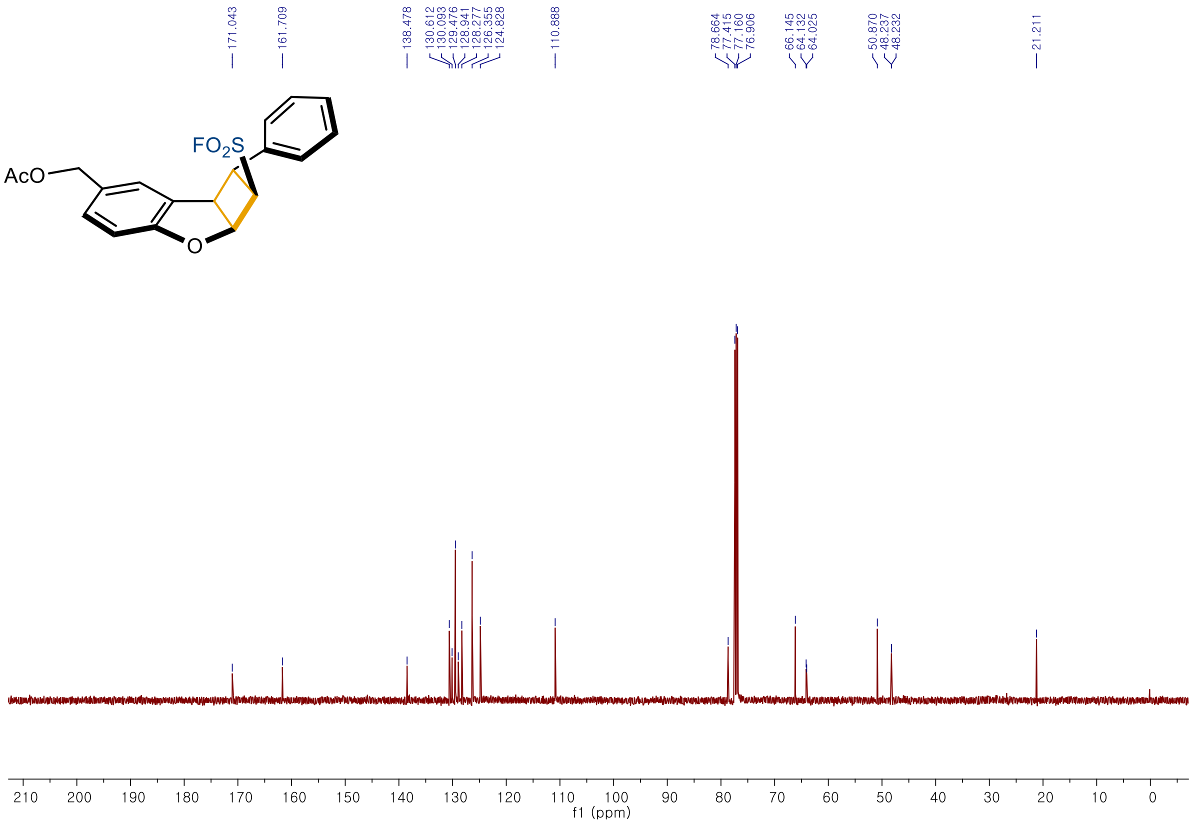

^{13}C NMR spectrum (**3ah**-*endo*)

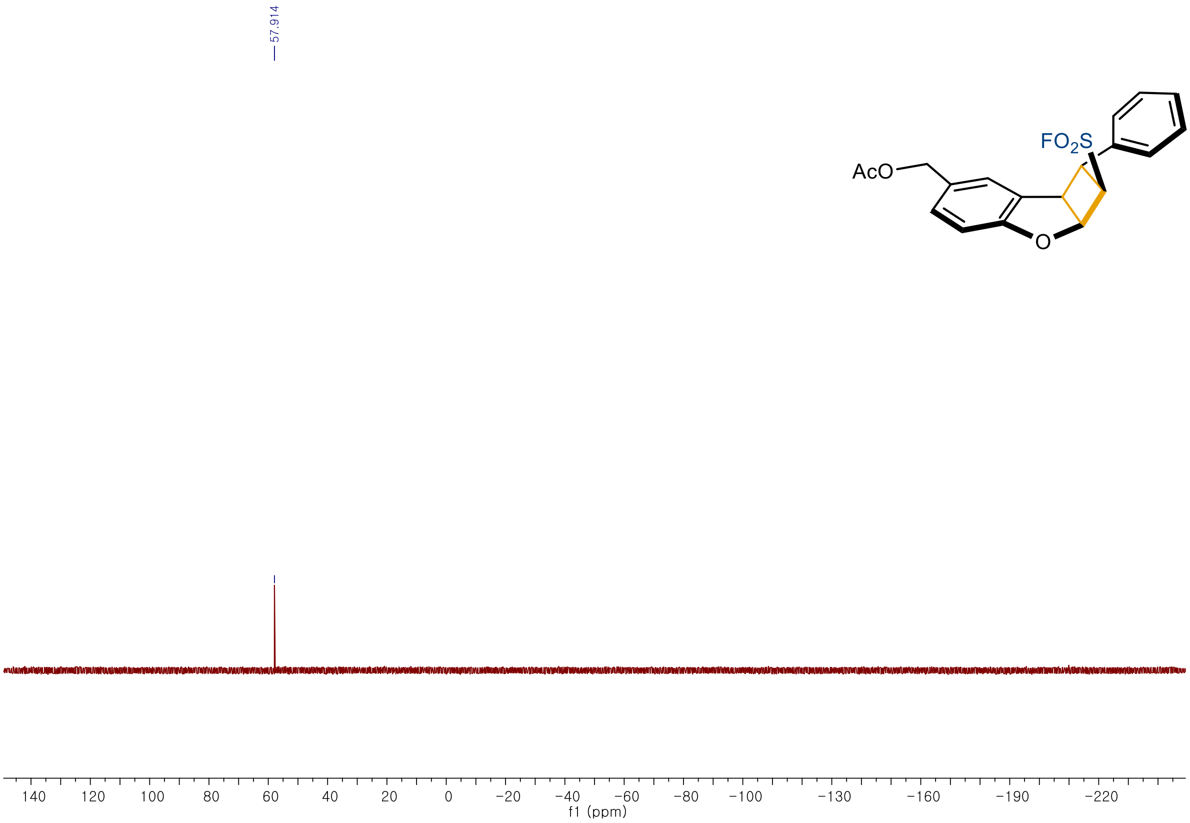

^{19}F NMR spectrum (**3ah**-*endo*)

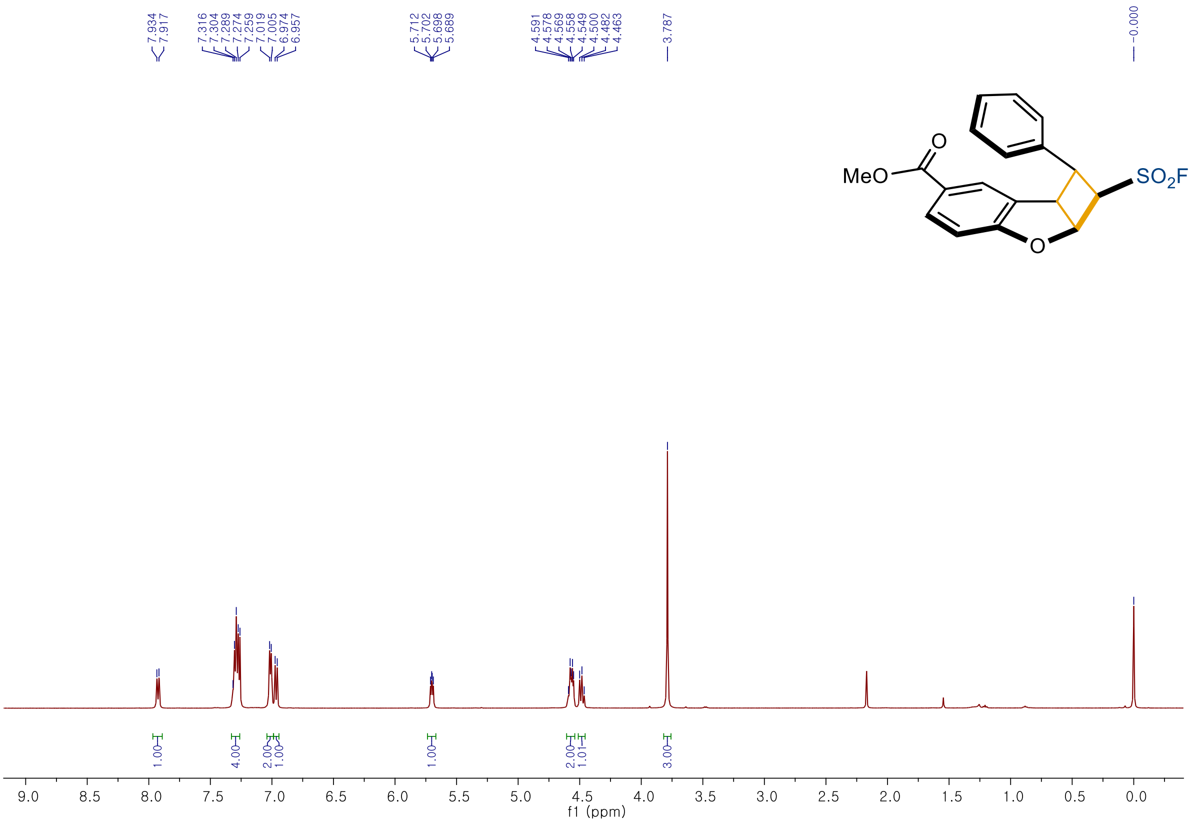

^{1}H NMR spectrum (**3ai**-*exo*)

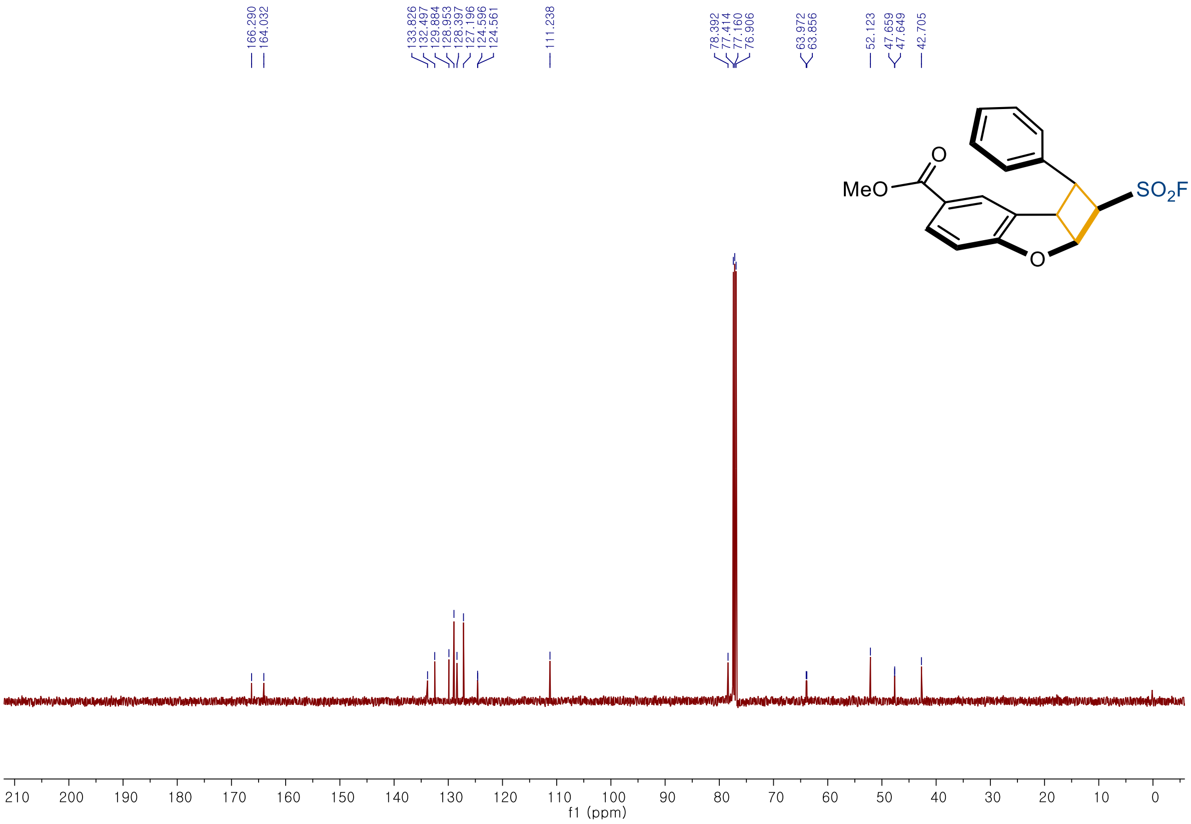

^{13}C NMR spectrum (**3ai**-*exo*)

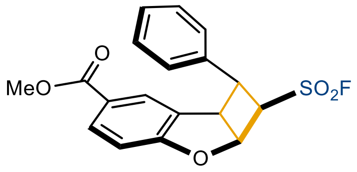

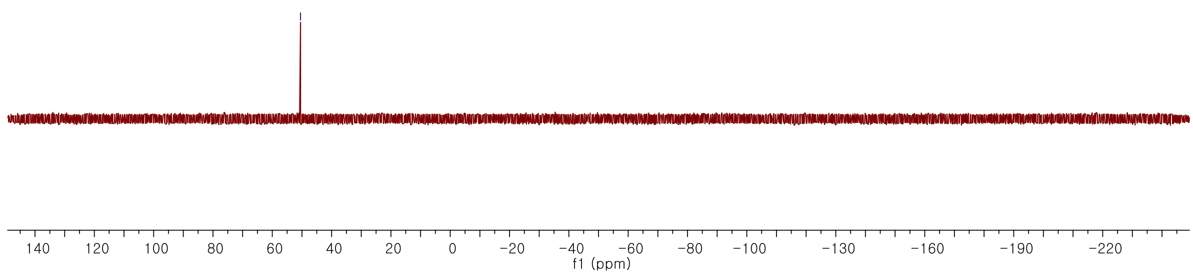

^{19}F NMR spectrum (**3ai**-*exo*)

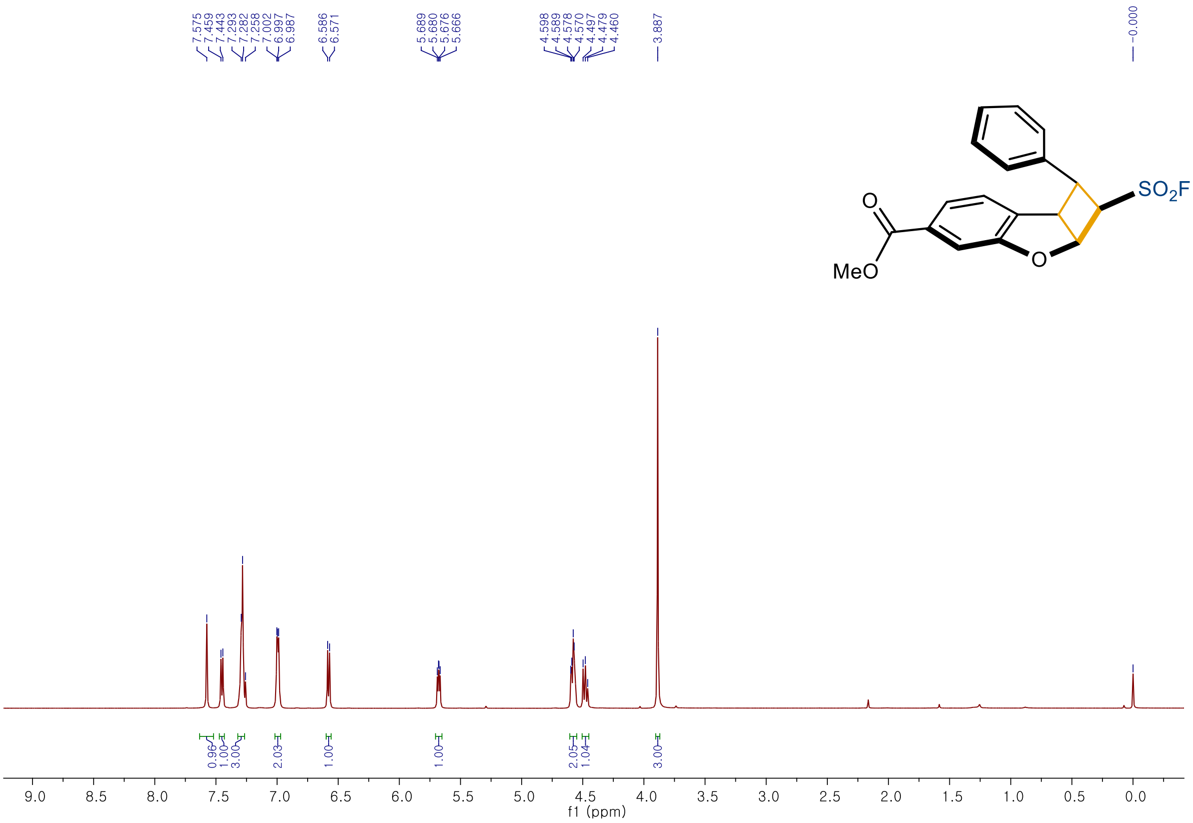

^{1}H NMR spectrum (**3aj**-*exo*)

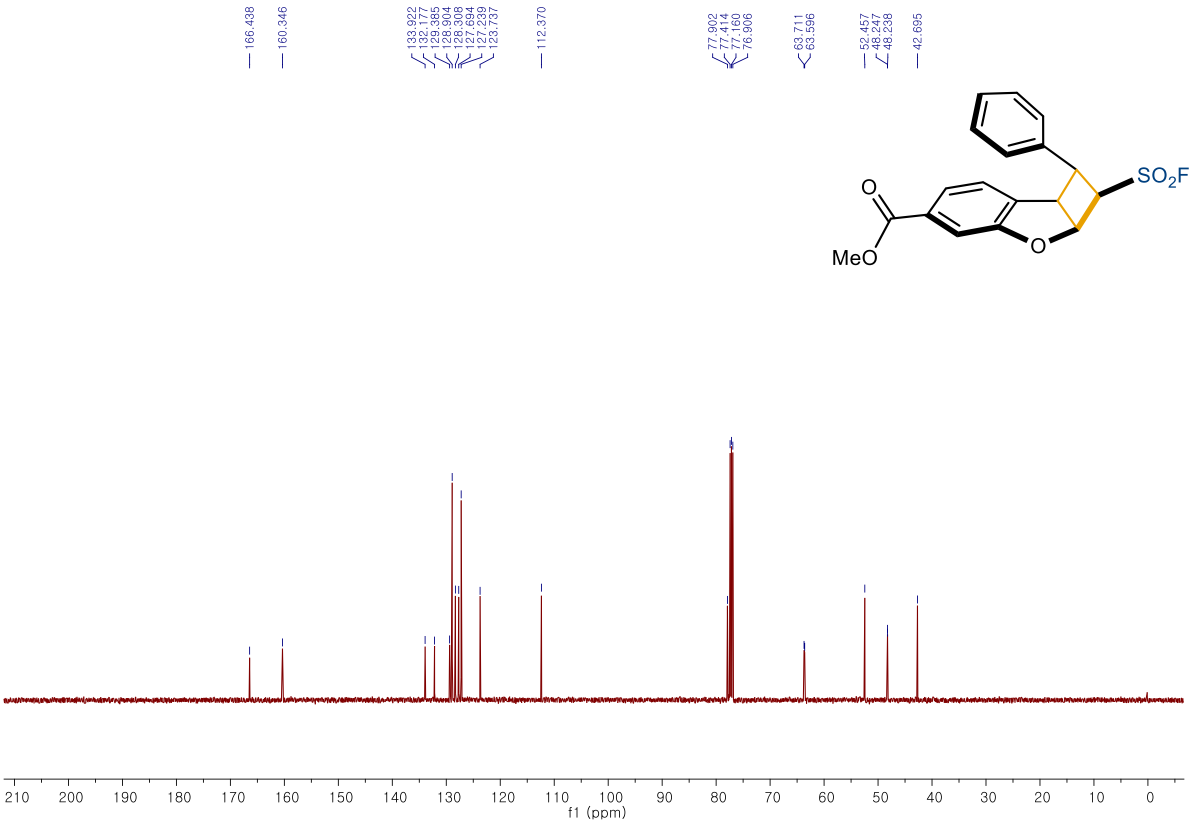

^{13}C NMR spectrum (**3aj**-*exo*)

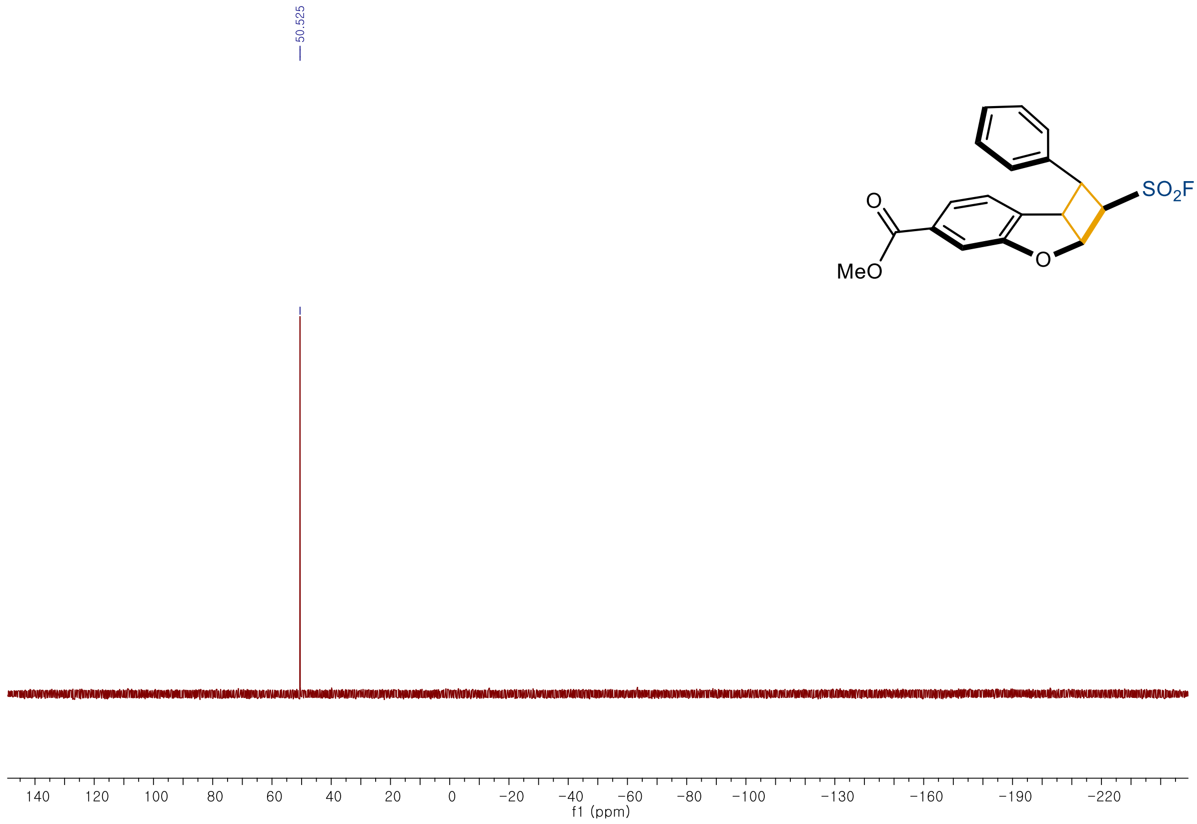

^{19}F NMR spectrum (**3aj**-*exo*)

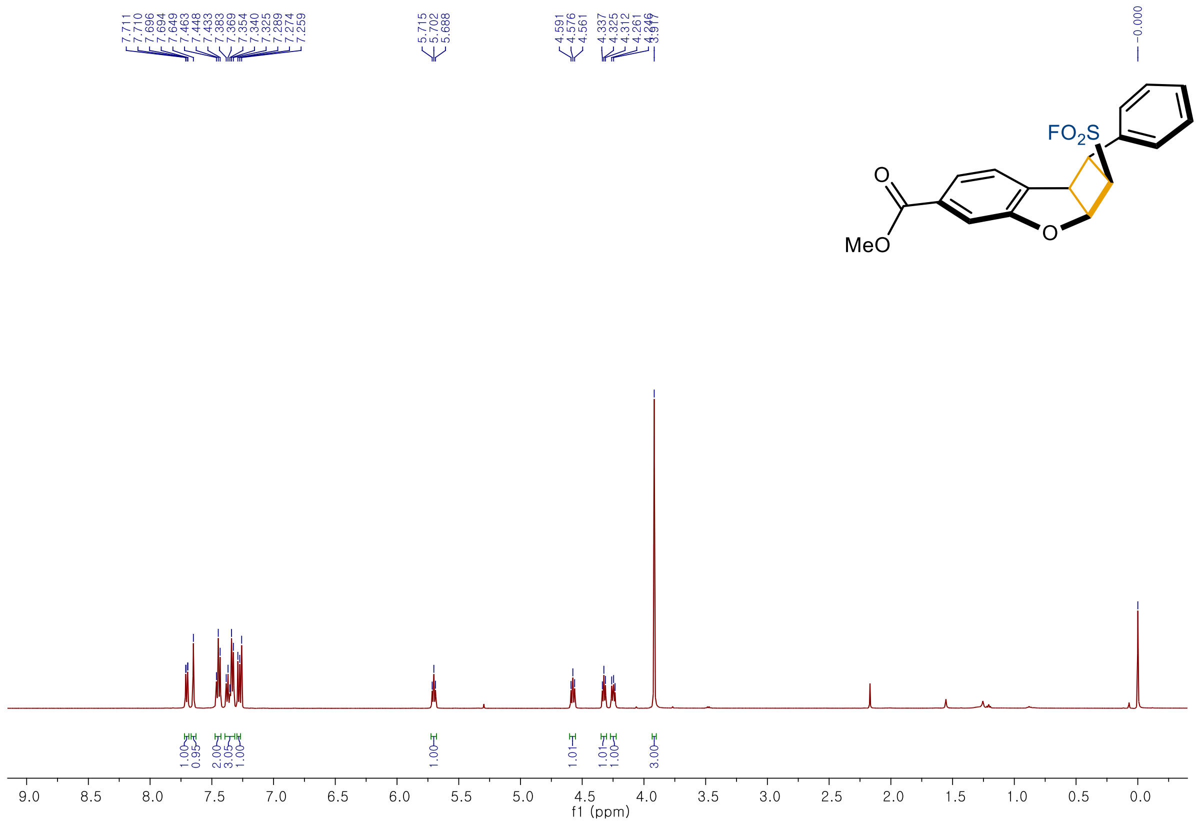

^{1}H NMR spectrum (**3aj**-*endo*)

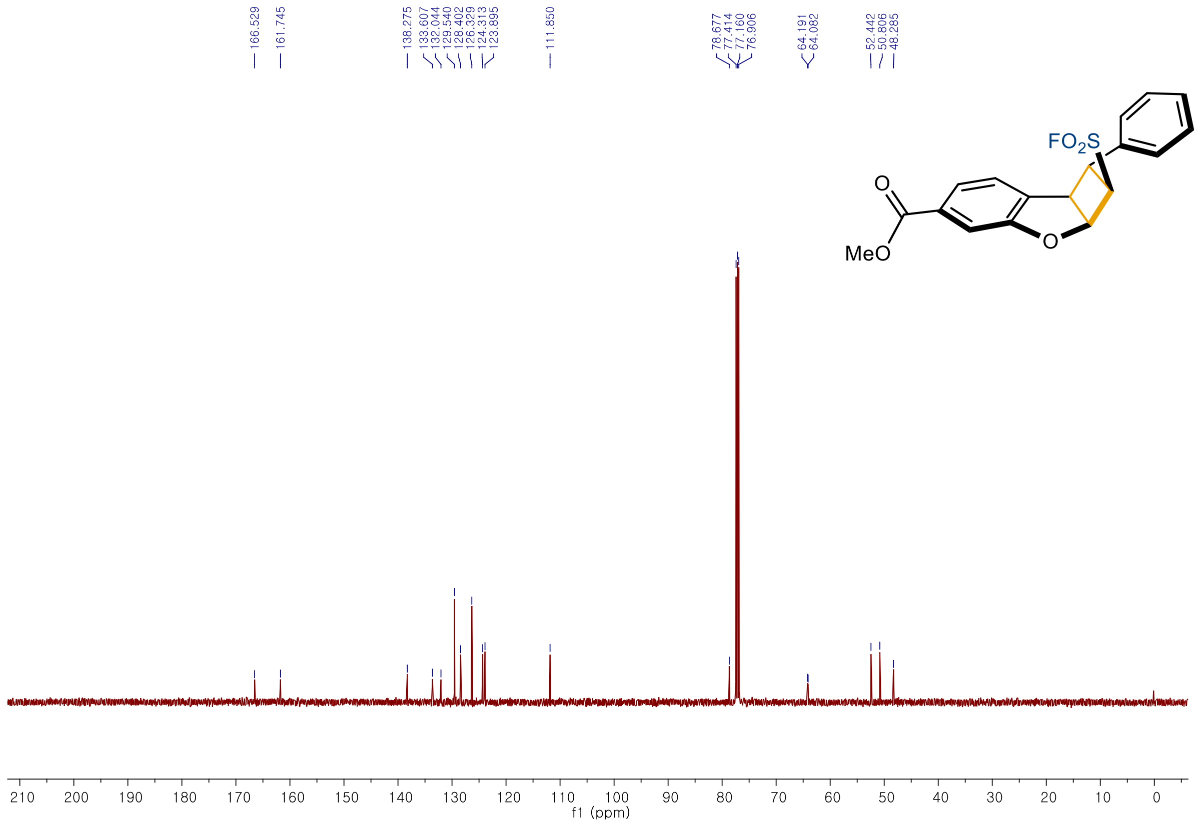

^{13}C NMR spectrum (**3aj**-*endo*)

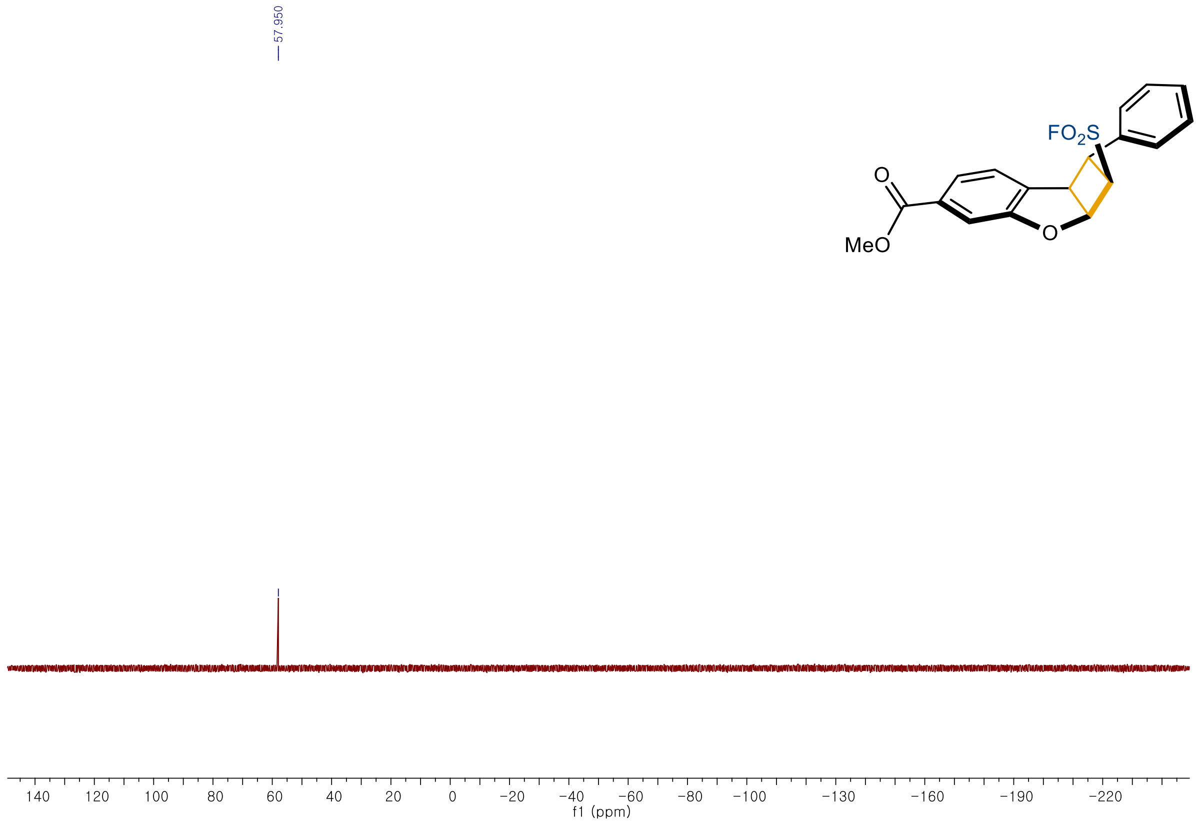

^{19}F NMR spectrum (**3aj**-*endo*)

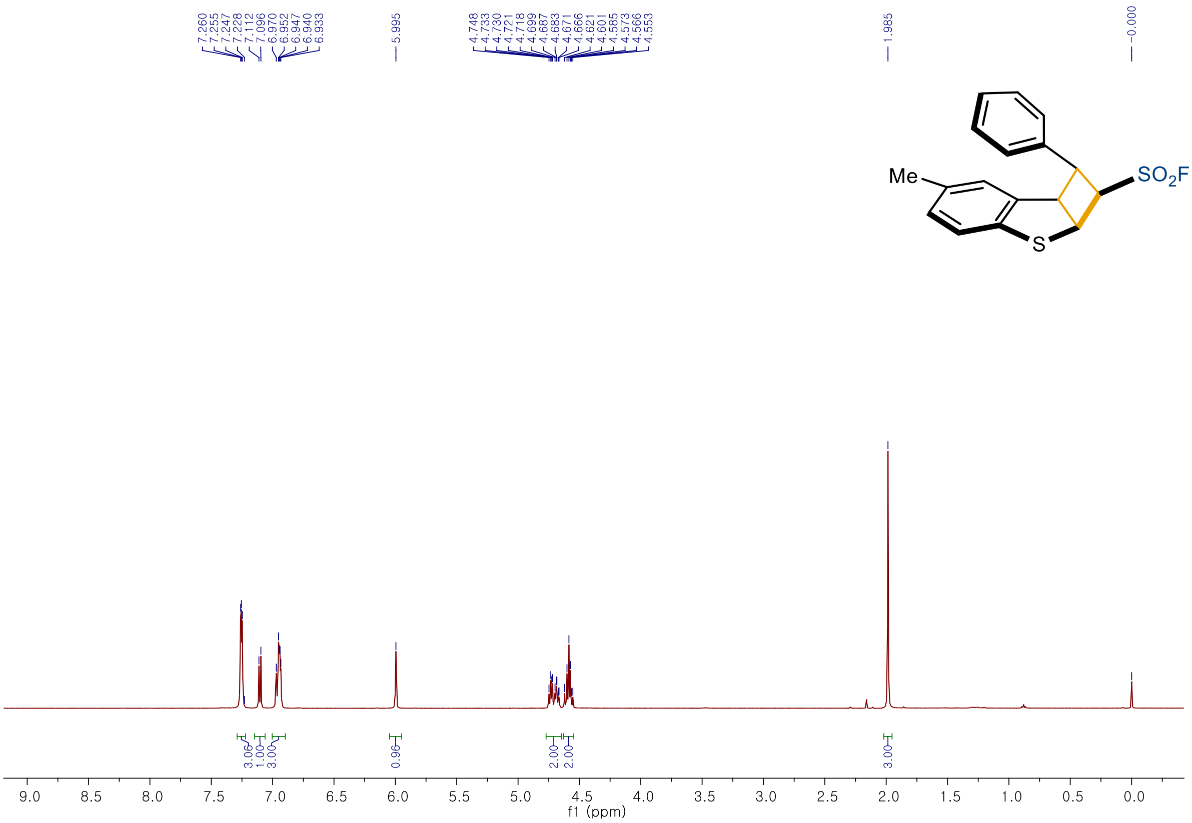

^{1}H NMR spectrum (**3ak**-*exo*)

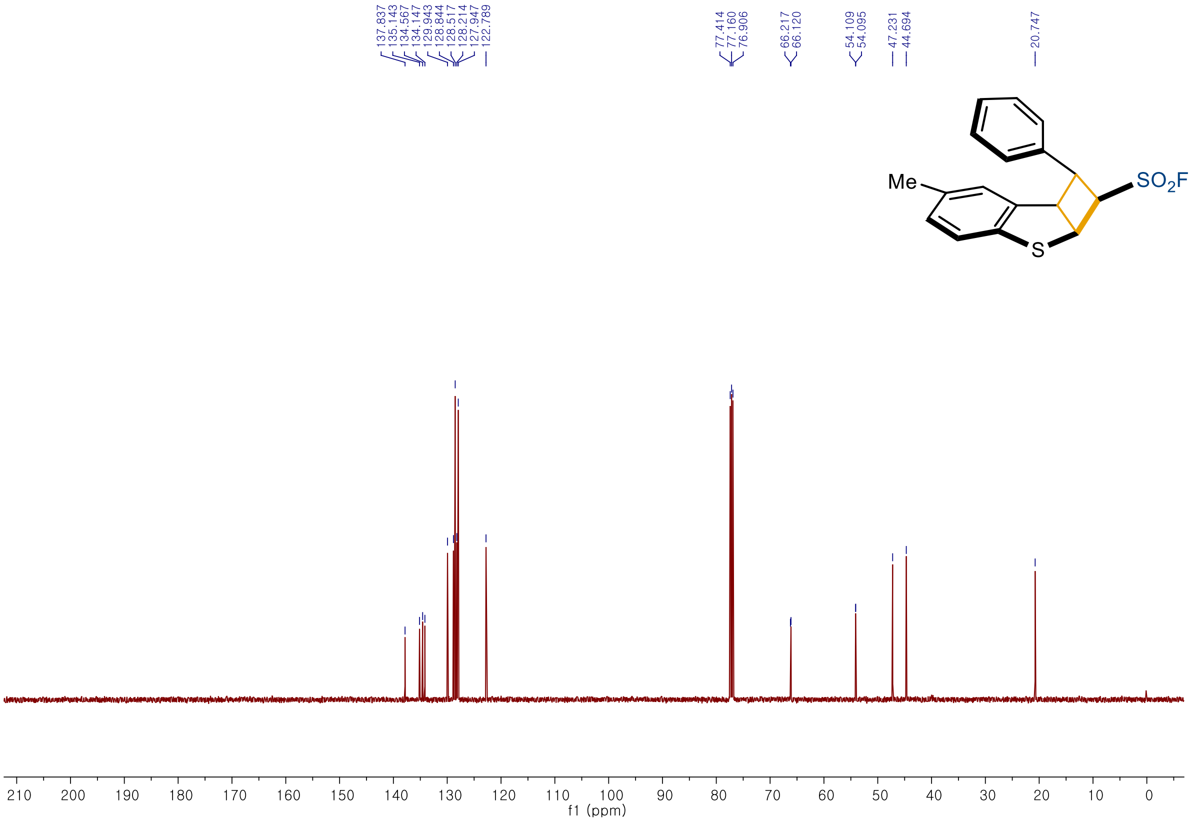

^{13}C NMR spectrum (**3ak**-*exo*)

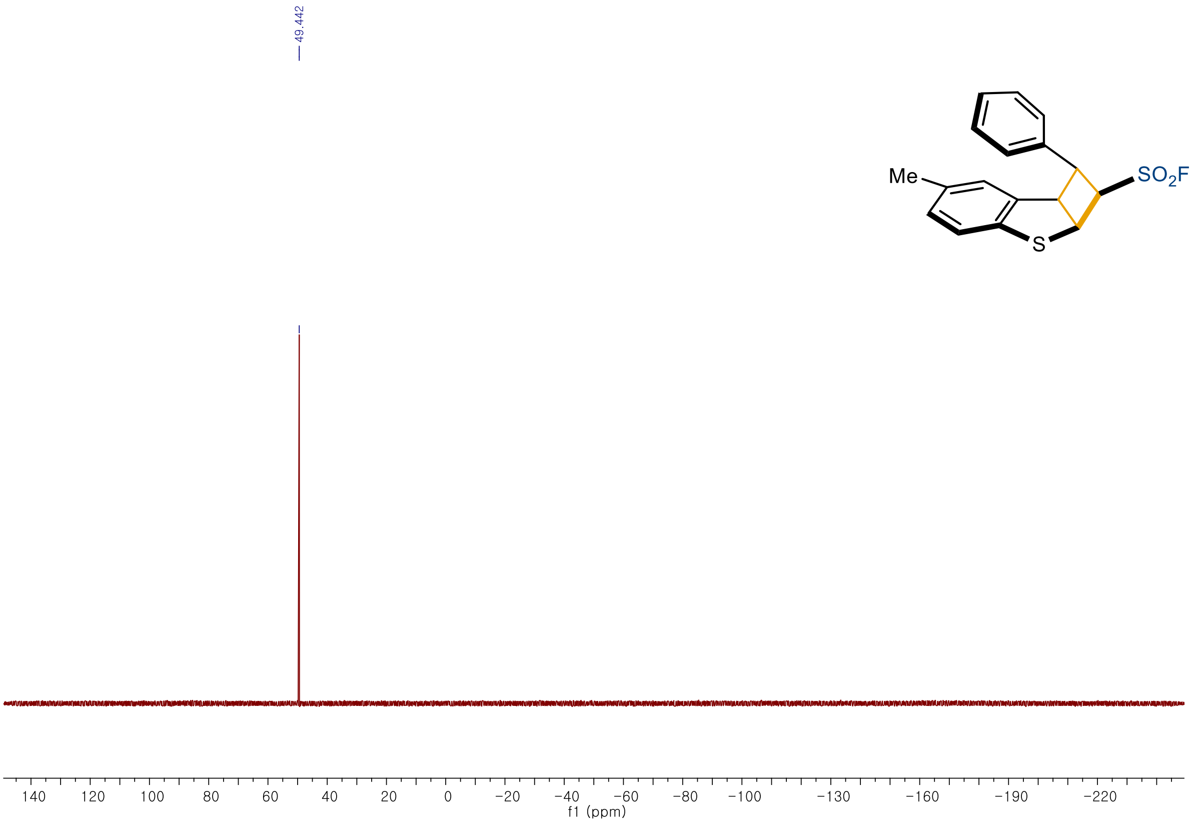

^{19}F NMR spectrum (**3ak**-*exo*)

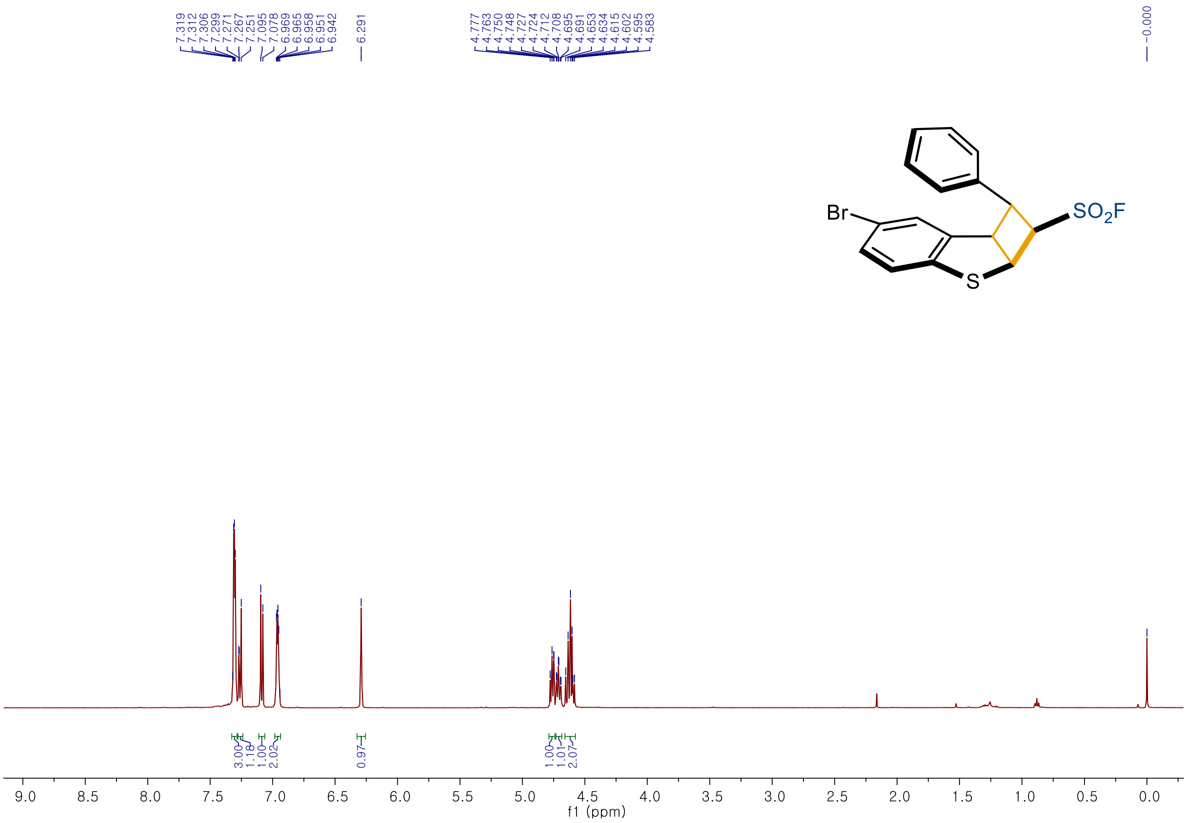

^{1}H NMR spectrum (**3al**-*exo*)

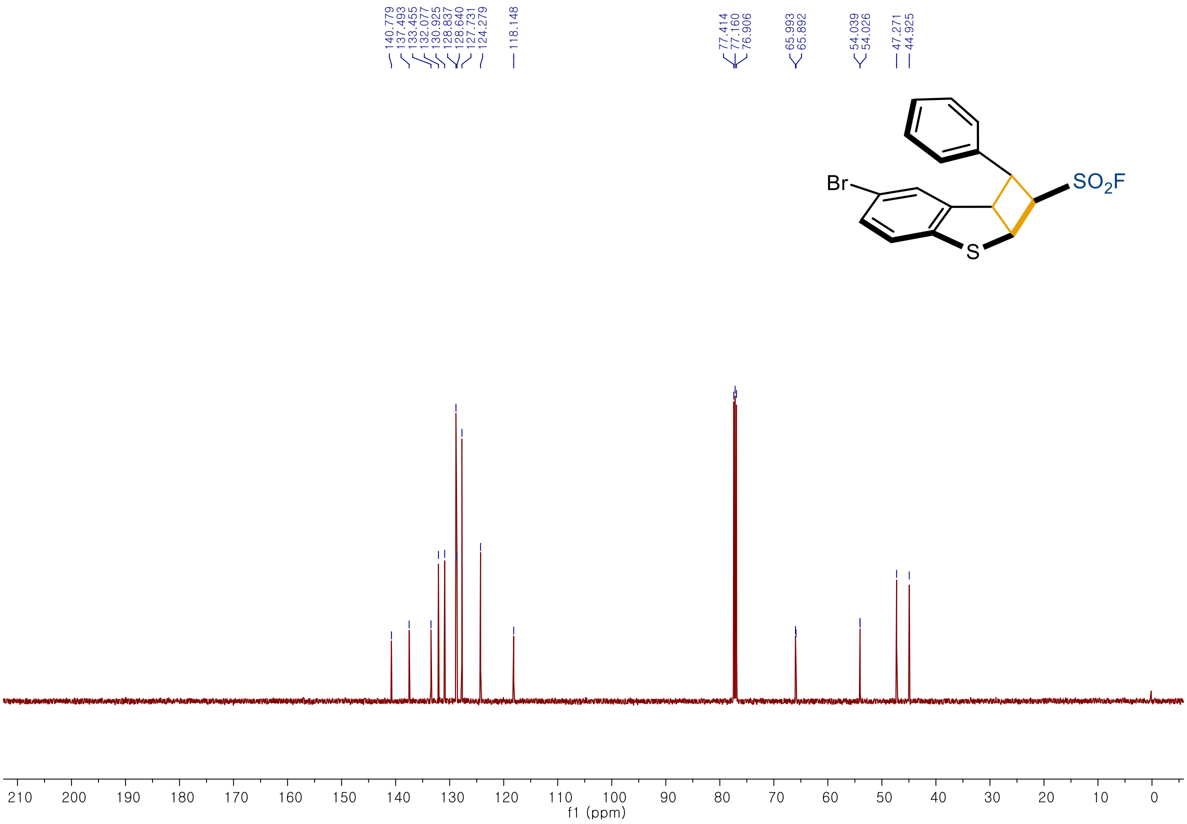

^{13}C NMR spectrum (**3al-***exo*)

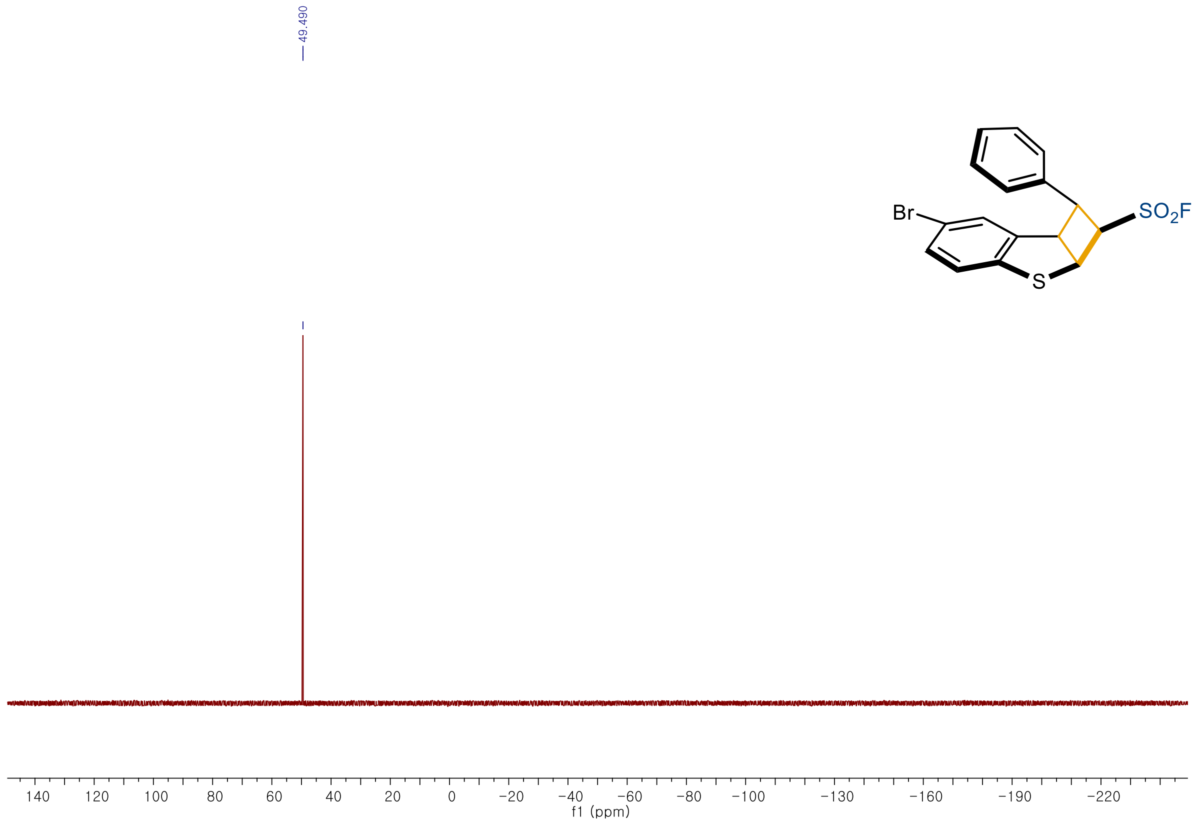

^{19}F NMR spectrum (**3al**-*exo*)

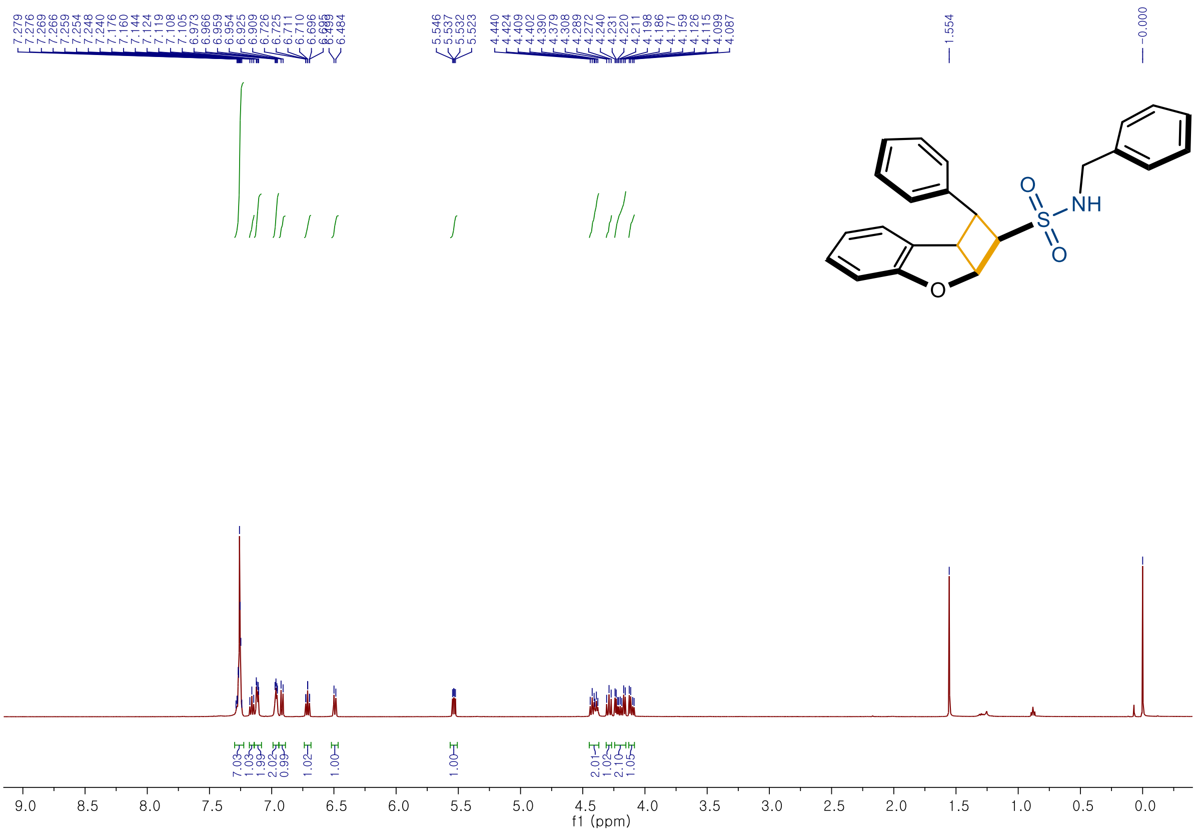

^{1}H NMR spectrum (**4**)

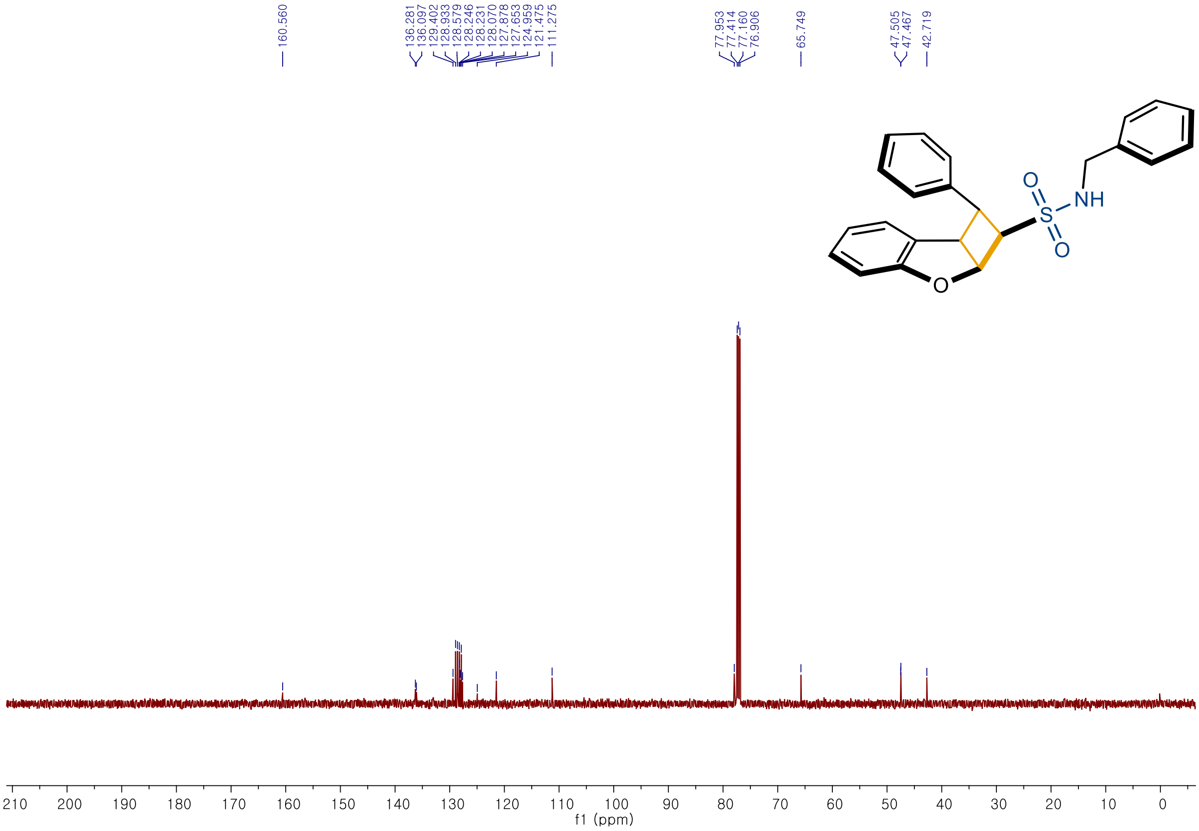

^{13}C NMR spectrum (**4**)

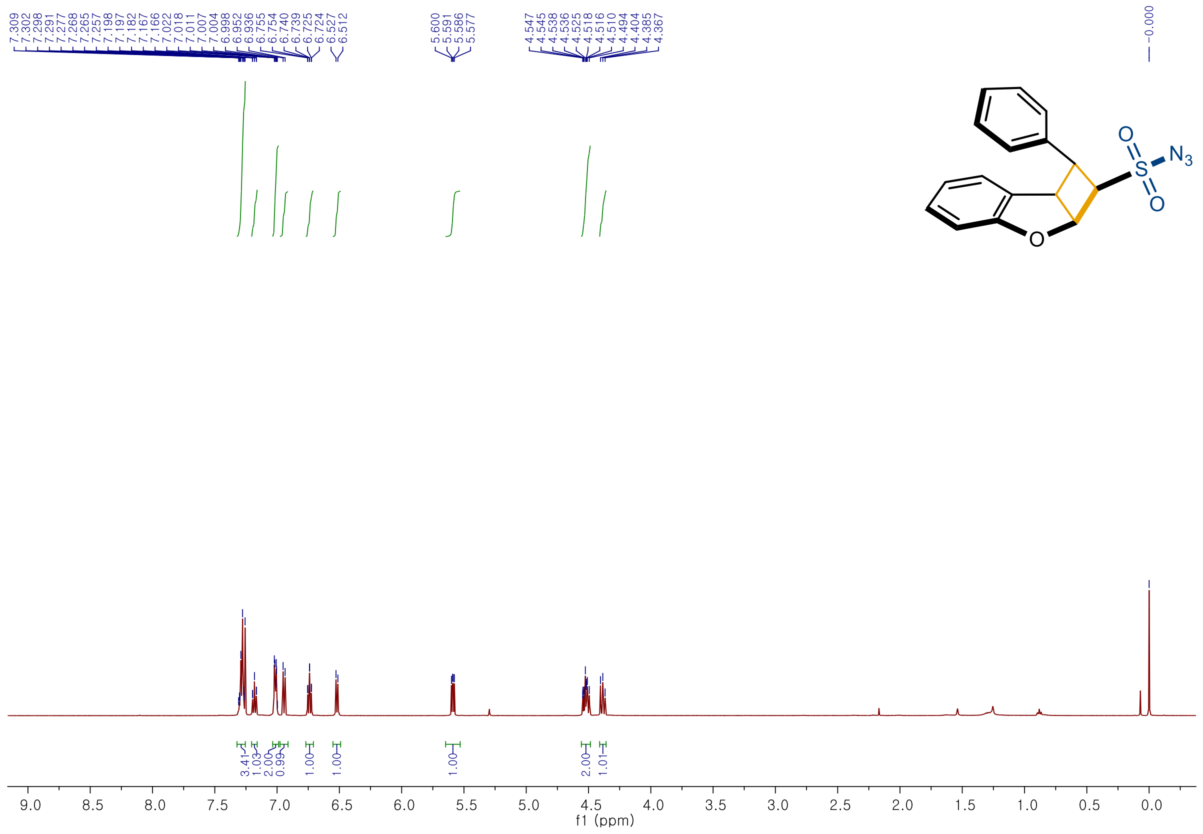

^{1}H NMR spectrum (**5**)

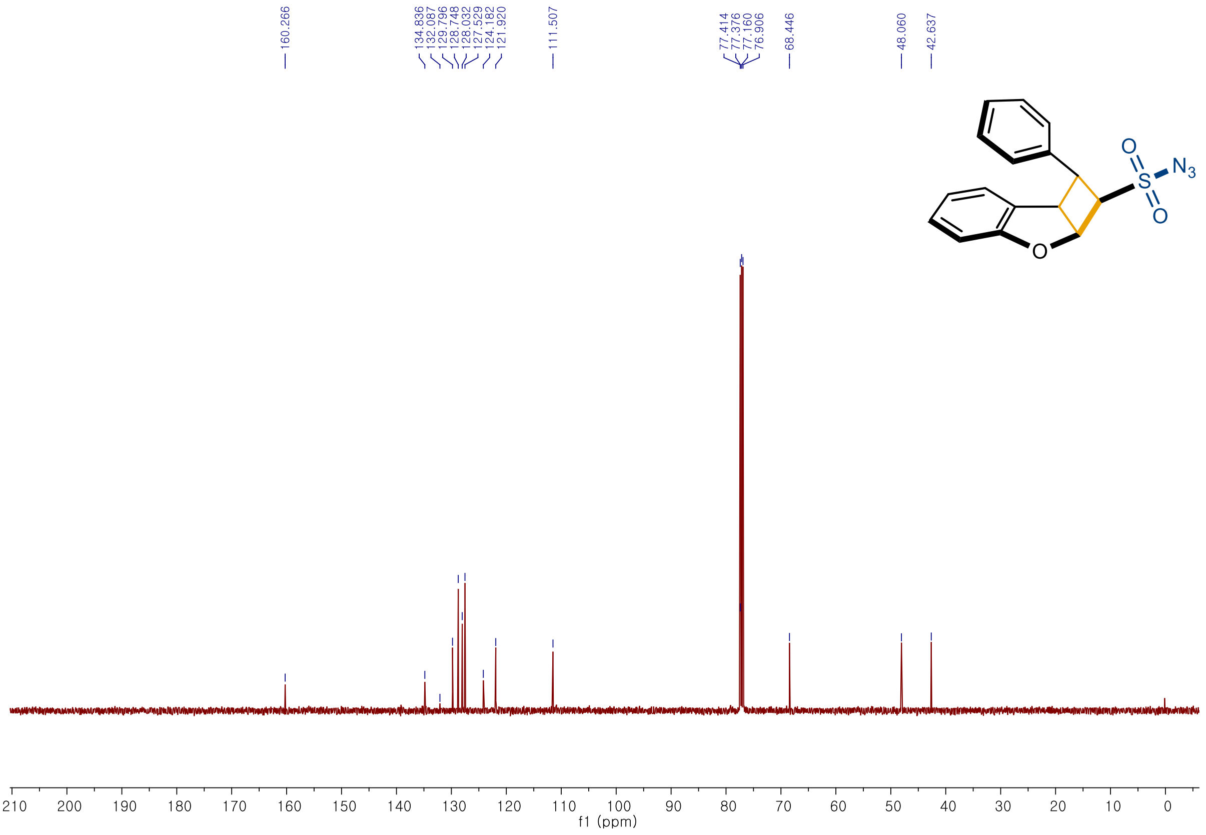

^{13}C NMR spectrum (**5**)

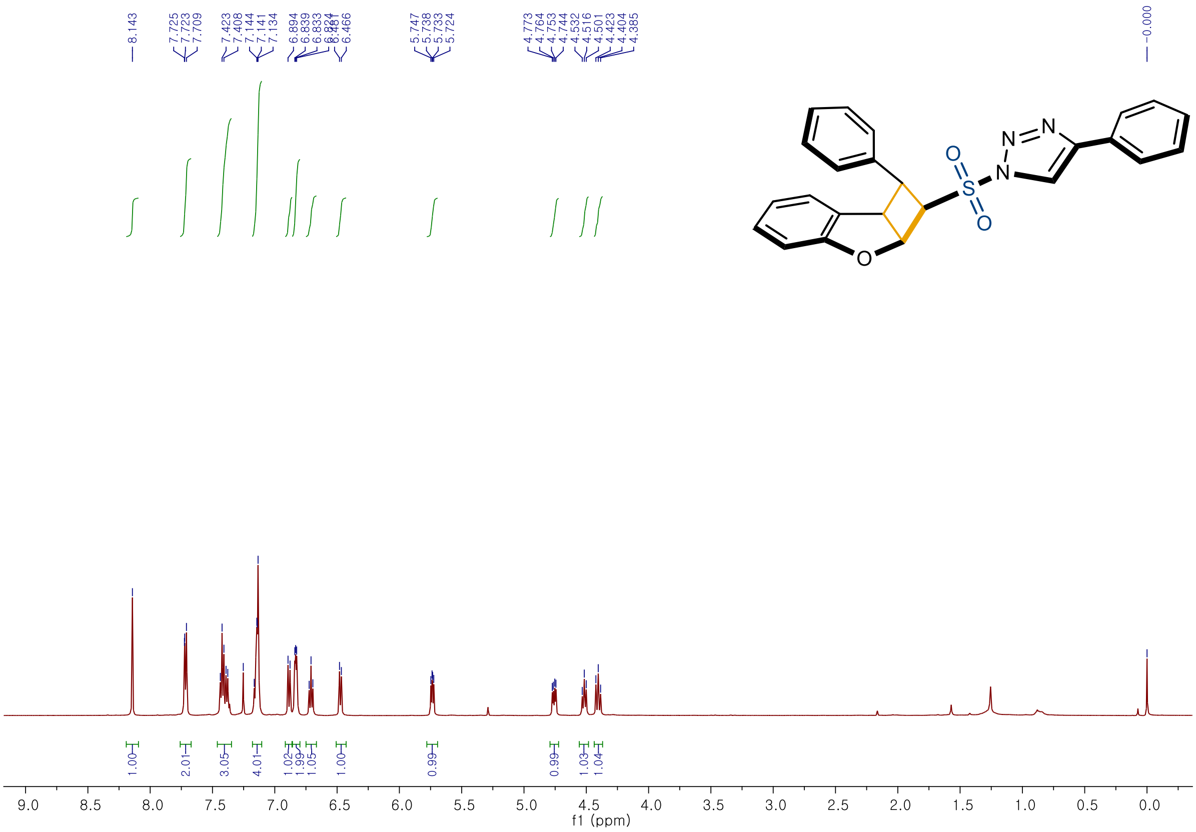

^{1}H NMR spectrum (**6**)

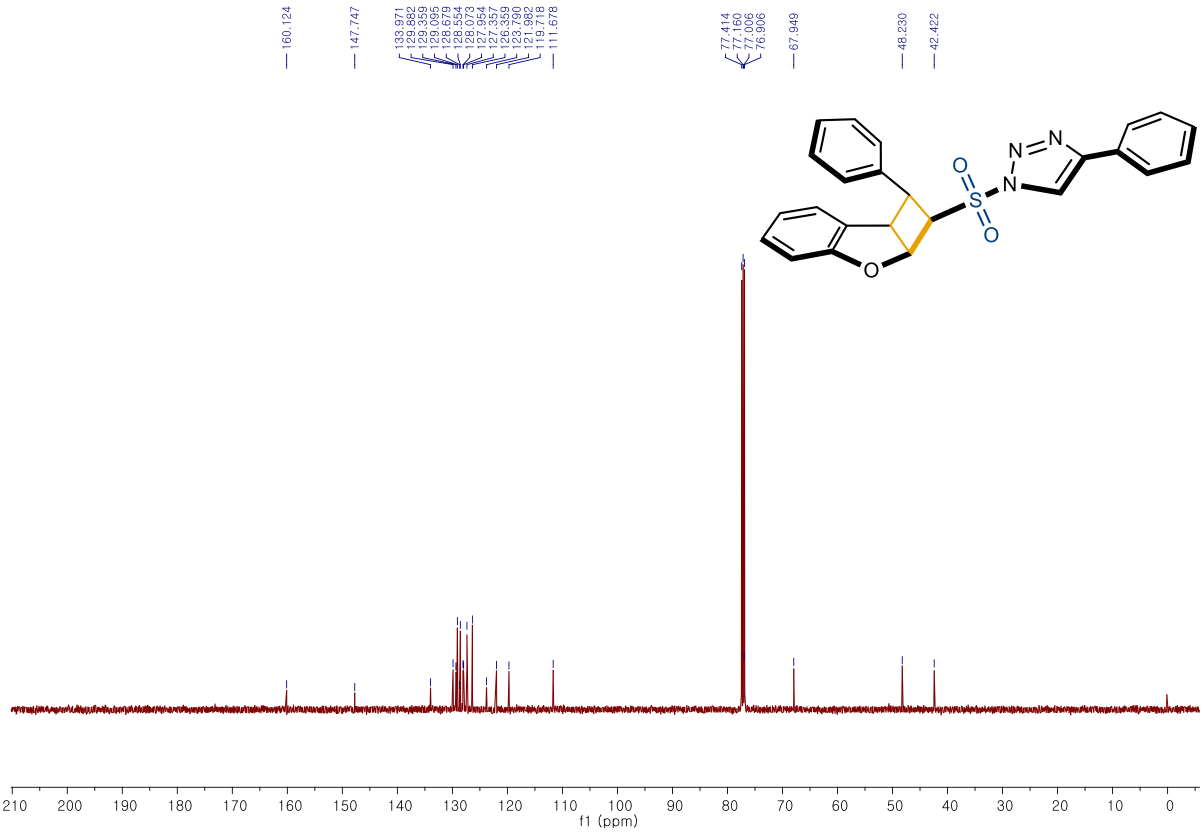

^{13}C NMR spectrum (**6**)

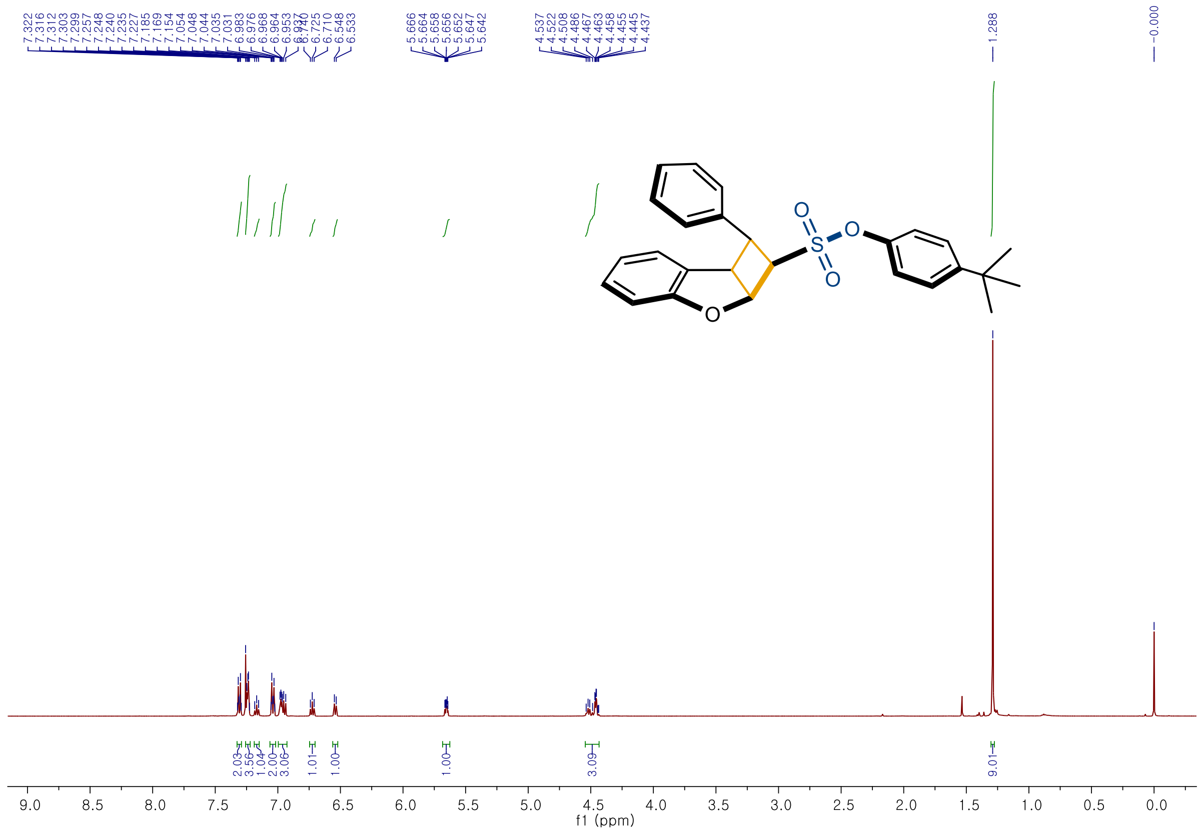

^{1}H NMR spectrum (**7**)

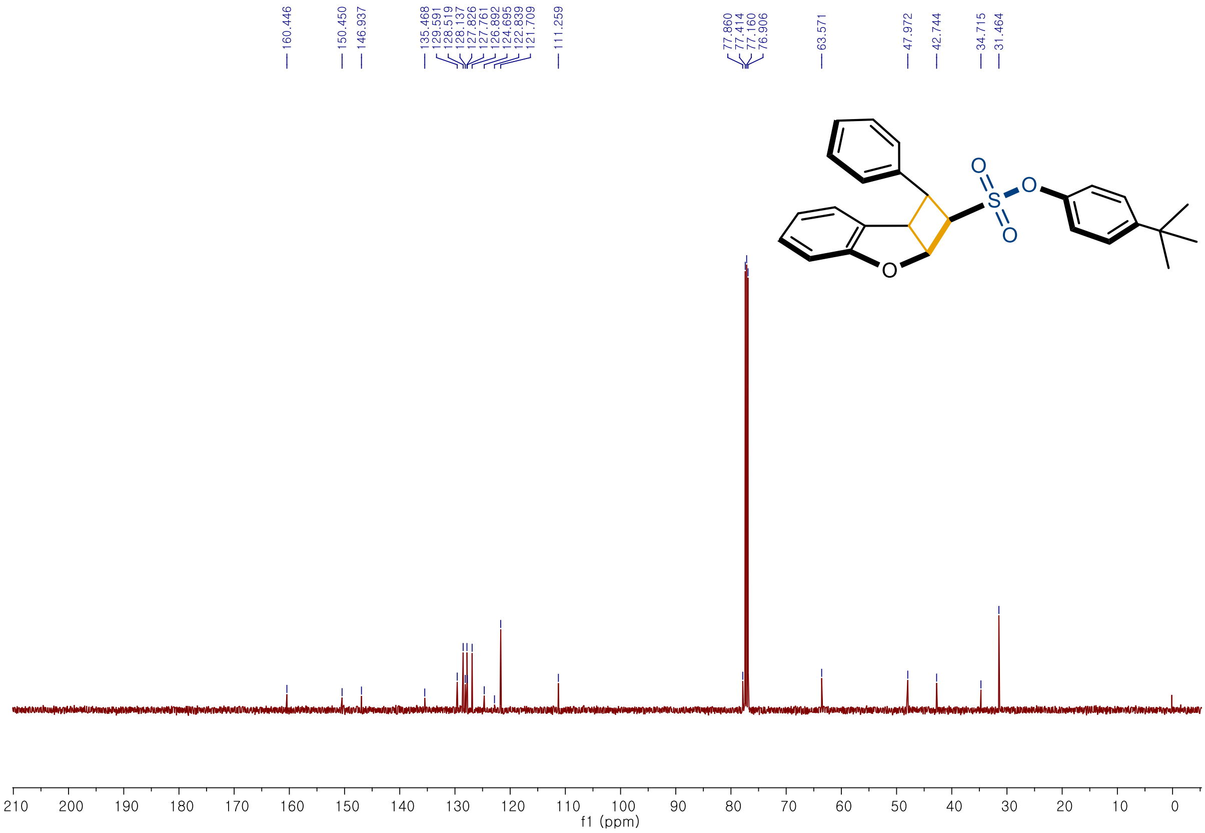

^{13}C NMR spectrum (**7**)

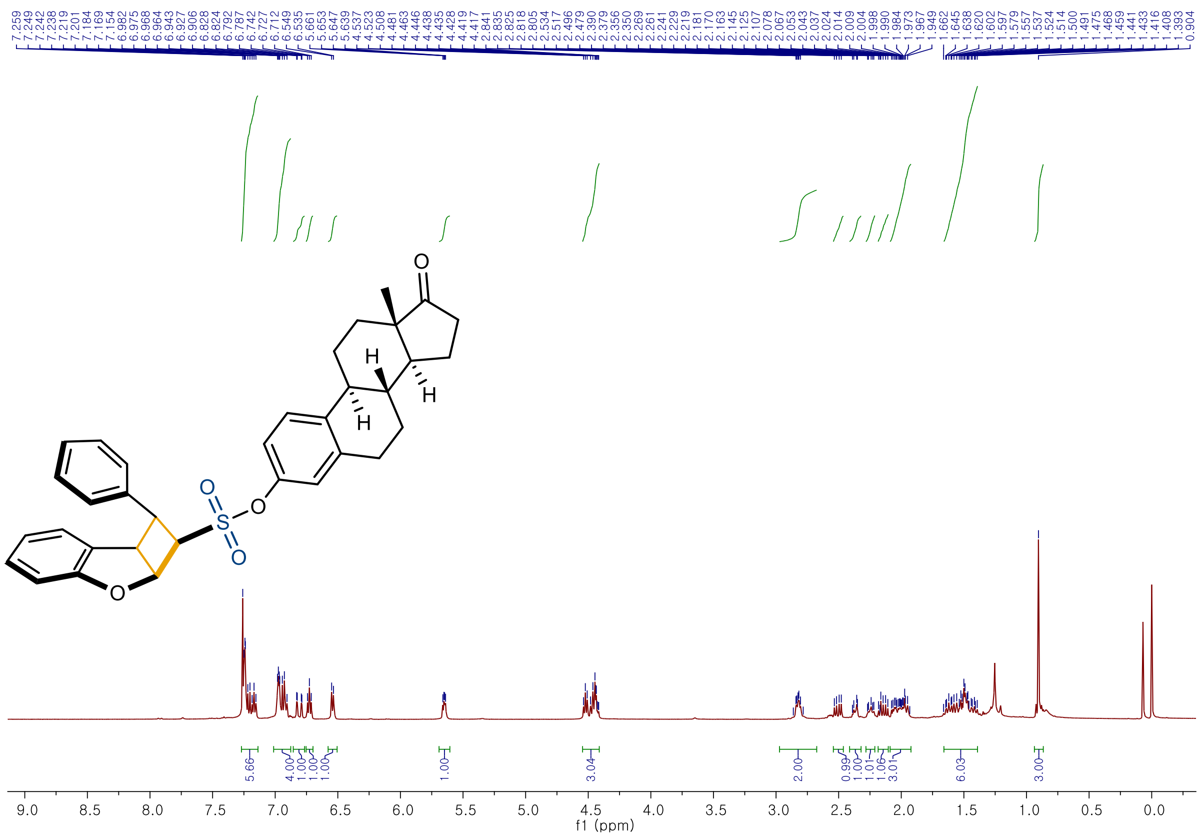

^{1}H NMR spectrum (**8**)

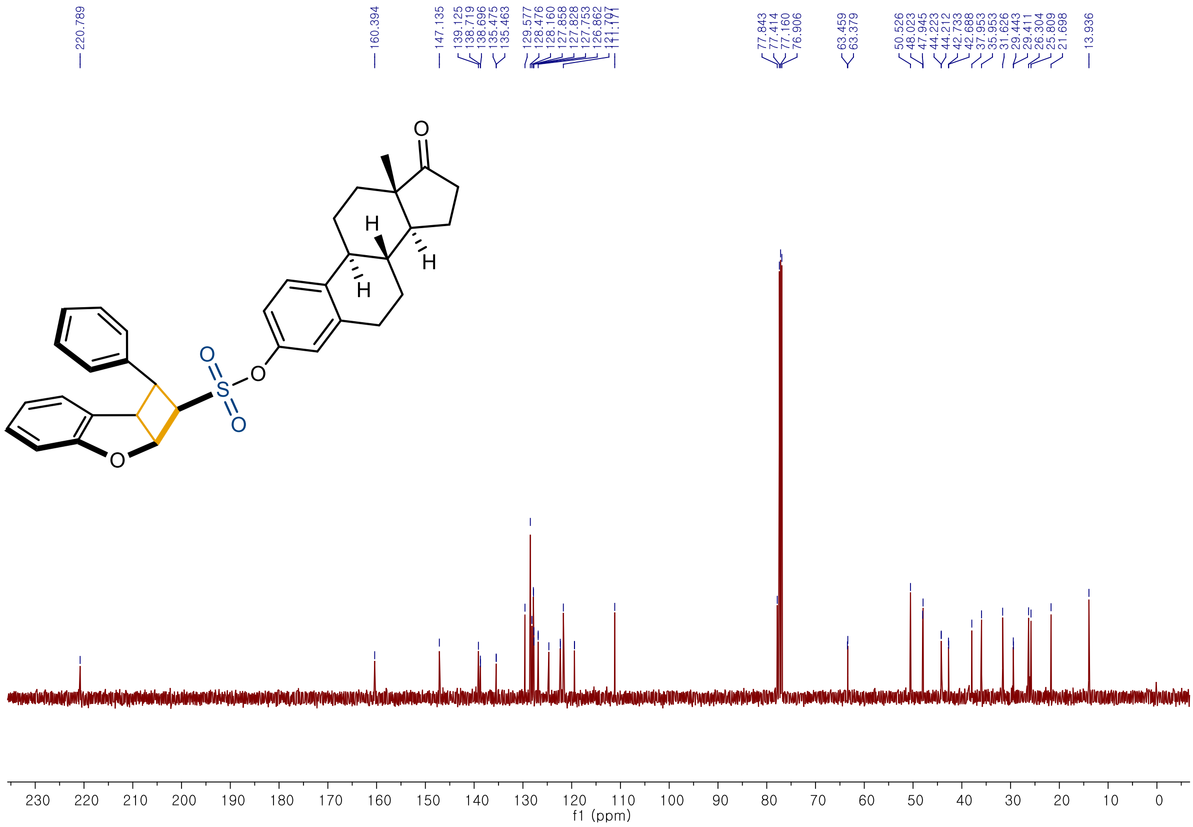

^{13}C NMR spectrum (**8**)

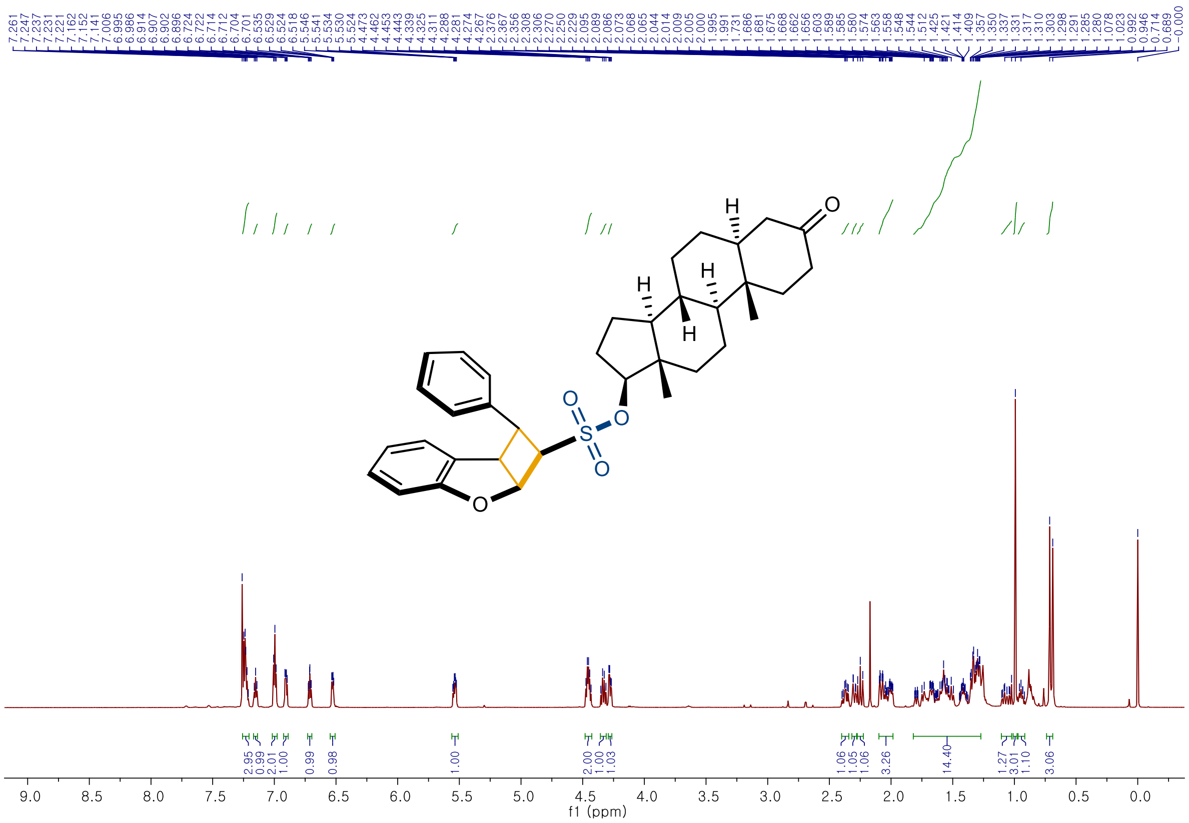

^{1}H NMR spectrum (**9**)

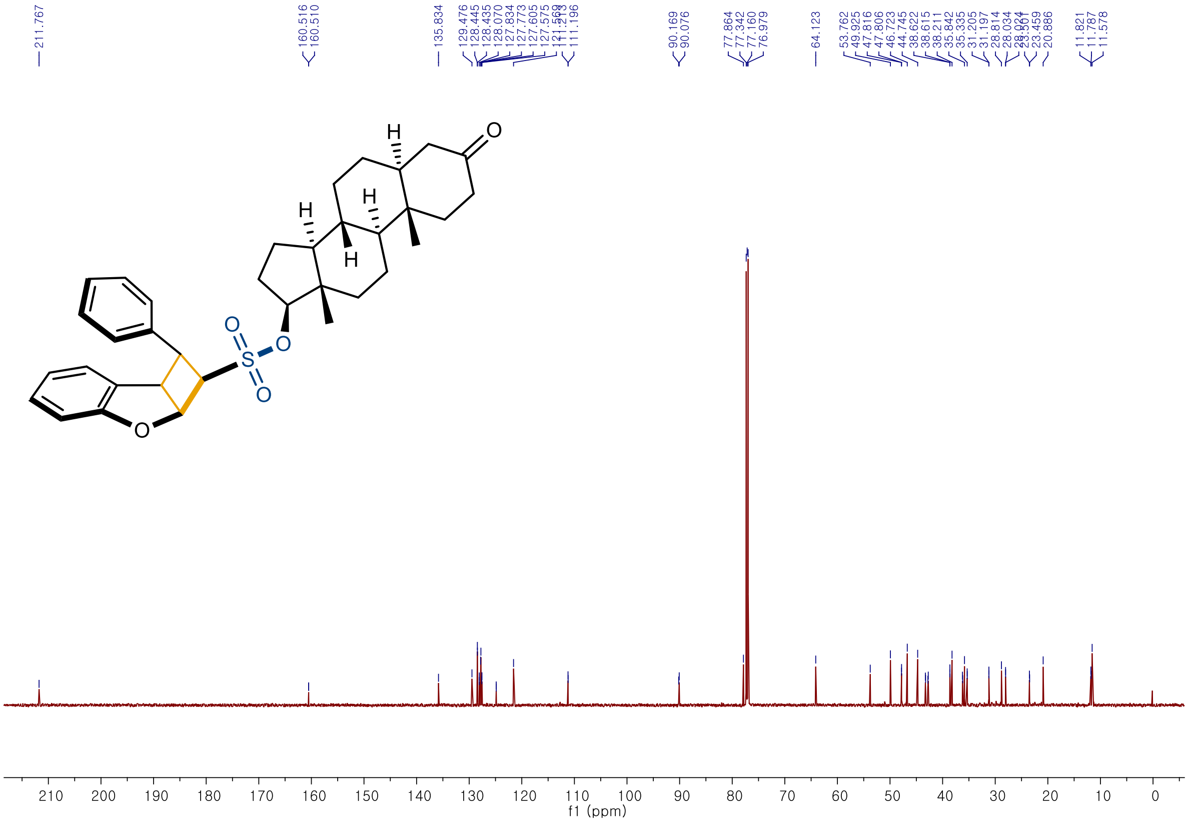

^{13}C NMR spectrum (**9**)

Supplementary References

1. Truce, W. E. & Hoerger, F. D. Diels-alder reactions with arylethenesulfonyl fluorides. *J. Am. Chem. Soc.* **76**, 3230–3232 (1954).

2. Zheng, Q., Dong, J. & Sharpless, K. B. Ethenesulfonyl fluoride (ESF): an on-water procedure for the kilogram-scale preparation. *J. Org. Chem.* **81**, 11360–11362 (2016).

3. Zha, G. -F., Zheng, Q., Leng, J., Wu, P., Qin, H. -L. & Sharpless, K. B. Palladium-catalyzed fluorosulfonylvinylation of organic iodides. *Angew. Chem. Int. Ed*. **56**, 4849–4852 (2017).

4. Bhuyana, M. & Koenig, B. Temperature responsive phosphorescent small unilamellar vesicles. *Chem. Commun*. **48**, 7489–7491 (2012).

5. Tan, G. et al. Photochemical single-step synthesis of β-amino acid derivatives from alkenes and (hetero)arenes. *Nat. Chem*. **14**, 1174–1184 (2022).

6. Yu, X., Meng, Q. -Y., Daniliuc, G. C. & Stude, A. Aroyl fluorides as bifunctional reagents for dearomatizing fluoroaroylation of benzofurans. *J. Am. Chem. Soc.* **114**, 7072–7079 (2022).

7. Kwon, Y. et al. Formation and degradation of strongly reducing cyanoarene-based radical anions towards efficient radical anion-mediated photoredox catalysis. *Nat. Commun*. **14**, 92 (2023).

8. Xu, R., Xu, T., Yang, M., Cao, T. & Liao, S. A rapid access to aliphatic sulfonyl fluorides. *Nat. Commun*. **10**, 3752 (2019).

9. Smedley, C. J. et al. Accelerated SuFEx click chemistry for modular synthesis. *Angew. Chem. Int. Ed*. **61**, e202112375 (2022).

10. Chapman, S. J., et al. Cooperative stereoinduction in asymmetric photocatalysis. *J. Am. Chem. Soc.* **144**, 4206–4213 (2022).

11. Speckmeier, E., Fischer, T. G. & Zeitler, K. (2018). A toolbox approach to construct broadly applicable metal-free catalysts for photoredox chemistry: deliberate tuning of redox potentials and importance of halogens in donor−acceptor cyanoarenes. *J. Am. Chem. Soc*. **140**, 15353–15365 (2018).
